# Detection of copy number variation and selection signatures on the X chromosome in Chinese indigenous sheep with different types of tail

**DOI:** 10.5713/ajas.18.0661

**Published:** 2019-07-01

**Authors:** Caiye Zhu, Mingna Li, Shizhen Qin, Fuping Zhao, Suli Fang

**Affiliations:** 1 College of Animal Science and Technology, Gansu Agricultural University, Lanzhou 730070, China; 2 National Center for Molecular Genetics and Breeding of Animal, Institute of Animal Sciences, Chinese Academy of Agricultural Sciences, Beijing 100193, China

**Keywords:** Copy Number Variation (CNV), Selection Signature, X Chromosome, Sheep

## Abstract

**Objective:**

Chinese indigenous sheep breeds can be classified into the following three categories by their tail morphology: fat-tailed, fat-rumped and thin-tailed sheep. The typical sheep breeds corresponding to fat-tailed, fat-rumped, and thin-tailed sheep are large-tailed Han, Altay, and Tibetan sheep, respectively. Detection of copy number variation (CNV) and selection signatures provides information on the genetic mechanisms underlying the phenotypic differences of the different sheep types.

**Methods:**

In this study, PennCNV software and F-statistics (F_ST_) were implemented to detect CNV and selection signatures, respectively, on the X chromosome in three Chinese indigenous sheep breeds using ovine high-density 600K single nucleotide polymorphism arrays.

**Results:**

In large-tailed Han, Altay, and Tibetan sheep, respectively, a total of six, four and 22 CNV regions (CNVRs) with lengths of 1.23, 0.93, and 7.02 Mb were identified on the X chromosome. In addition, 49, 34, and 55 candidate selection regions with respective lengths of 27.49, 16.47, and 25.42 Mb were identified in large-tailed Han, Altay, and Tibetan sheep, respectively. The bioinformatics analysis results indicated several genes in these regions were associated with fat, including dehydrogenase/reductase X-linked, calcium voltage-gated channel subunit alpha1 F, and patatin like phospholipase domain containing 4. In addition, three other genes were identified from this analysis: the family with sequence similarity 58 member A gene was associated with energy metabolism, the serine/arginine-rich protein specific kinase 3 gene was associated with skeletal muscle development, and the interleukin 2 receptor subunit gamma gene was associated with the immune system.

**Conclusion:**

The results of this study indicated CNVRs and selection regions on the X chromosome of Chinese indigenous sheep contained several genes associated with various heritable traits.

## INTRODUCTION

Chinese indigenous sheep breeds can be classified by their tail morphology into the following categories: fat-tailed, fat-rumped and thin-tailed sheep breeds.

Large-tailed Han sheep are primarily found throughout the hinterland of the North Plain of China. This region has a typical temperate continental monsoon climate with obvious seasonal changes for each of its four seasons. The hinterland is cold and dry in the winter and hot and rainy in the summer. Large-tailed Han sheep in this region have large, long, fan-shaped fat tails that hang down to their hocks. Their peach-shaped tail tips are upturned and hang near their tail grooves.

Altay sheep are primarily distributed throughout the Fuhai and Fuyun counties in the Altay region of the Xinjiang Uygur Autonomous Region. The central production area of these sheep has a typical continental climate, with an annual average temperature of 4.0°C, an extreme minimum temperature of −42.7°C, an annual snow cover of 200 to 250 days and a snow thickness of 15 to 20 cm. The fat deposited on the buttocks of Altay sheep causes the sheep to have fat, rounded hips, which are wide, straight and large. In the middle of the lower edges of these hips, shallow grooves divide the hips into two symmetrical halves.

Tibetan sheep are native to the Qinghai-Tibet Plateau and are primarily distributed throughout the Tibet Autonomous Region and Qinghai. The central production area of these sheep is located at 26°50′-36°53′ north latitude and 78°25′-99°06′ east longitude, which is in the southwestern part of the Qinghai-Tibet Plateau, and has an average elevation of over 4,000 m. The climate is characterized by a long sunshine duration, strong radiation, low temperatures, a large temperature difference, clear and wet sky conditions, long night rains, a dry winter and spring, a high wind pressure, a low air pressure and a low oxygen content. The tails of Tibetan sheep are short and are shaped like flat cones.

Worldwide, more than 25% of sheep breeds are fat-tailed or fat-rumped and accumulate a significant amount of fat in their tails [1]. Fat-tailed sheep are the result of breeding by humans and natural selection and appeared approximately 5,000 years ago [2]. The fat in the tails of fat-tailed and fat-rumped sheep has been used by humans as a high-energy food source during periods of drought and famine. In addition, fat-tailed and fat-rumped sheep are breeds that have adapted to extreme environments. Their fat serves as an energy reserve to support their migration and survival during cold winters.

Copy number variations (CNVs) are DNA segments with sizes ranging from 1 kilobase (Kb) to several megabases (Mb) in which duplication or deletion events have occurred [3]. CNV is a major source of phenotypic diversity and genetic variation [4]. For example, CNV in the KIT proto-oncogene, receptor tyrosine kinase gene leads to a white coat color in pigs [4]. In addition, the phenotype of a white and gray coat in goats is influenced by a CNV in the agouti signaling protein gene [5]. Zhu et al [6] detected CNV in sheep with different tail types, which included genes associated with fat deposition.

Artificial selection has played a significant role in the domestication of livestock. Domestication has reshaped the behavior, morphology and genetics of many livestock species. Selection has various effects on the genome itself. Positive selection can increase advantageous allele frequencies and fix them within a population [7]. Consequently, polymorphism at a selection site is then reduced in the population. Selection signatures can be detected through variation in the allele frequency and the decay of linkage disequilibrium [6]. With the development of high-density single nucleotide polymorphism (SNP) chips and high-throughput genotyping technology, a number of statistical methods have been used to explore the selection signatures in genes and the genome. Several selection signatures in sheep are associated with regions showing evidence of introgression from Asian breeds. A comparison of European sheep breeds and wild boars showed genomic regions with high levels of differentiation in both animals that were found to harbor genes related to bone formation, growth and fat deposition [8]. F-statistics (F_ST_) [9] are extensively used in identifying selection signatures, which have been used primarily to determine differences in the selection signatures between populations.

The X chromosome undergoes more drift than an autosome, as its effective population size is three-quarters that of an autosome [10]. The X chromosome is more specialized than an autosome and plays an important role in the evolution of humans and animals. The X chromosome has a high gene density and thus may be a good target for detecting CNV and selection signatures. Several studies reported the presence of selection footprints on the X chromosome in pigs and sheep and determined that genes relevant to meat quality, reproduction and the immune system were found in potential selection regions [11,12]. In addition, Zhu et al [6] detected selection signatures on the X chromosome in several sheep genes correlated with reproduction. Rubin et al [13] noted the X chromosome should be analyzed only for the identification of selection signatures. Furthermore, they suggested only sows should be used in selection signature studies because sex chromosomes and autosomes for different genders are subjected to different selective pressures and have different effective population sizes.

The purpose of this study was to identify CNV and selection signatures on the X chromosome in Chinese indigenous sheep breeds with different tail types. PennCNV software and a between-population method (F_ST_) were employed to analyze high-density 600K SNP genotype data. Subsequently, a stream of analysis was performed to explain fat deposition in the tail, including gene search and functional enrichment analysis methods.

## MATERIALS AND METHODS

### Ethics statement

All animal experiments were approved by Gansu Agricultural University (Lanzhou, China). All procedures for animals were performed in strict accordance with the guidelines proposed by the China Council on Animal Care and the Ministry of Agriculture of the People’s Republic of China.

### DNA sample collection

In total, 120 individuals from three breeds, including 40 large-tailed Han sheep (20 rams and 20 ewes), 40 Altay sheep (20 rams and 20 ewes), and 40 Tibetan sheep (20 rams and 20 ewes), were collected from Liaocheng in Shandong Province, Altay in Xinjiang Province and Tianzhu in Gansu Province, respectively. All specimens were randomly selected. After a principal component analysis (PCA) was performed to identify population structure and the relatedness of animals, 60 females, comprising 20 large-tailed Han, 20 Altay, and 20 Tibetan sheep, were chosen for detecting CNV in and identifying selection signatures on the X chromosome.

Genomic DNA samples were extracted from blood using the TIANamp Blood DNA Kit (Tiangen Biotech Co. Ltd., Beijing, China). The purity and concentration of genomic DNA were measured using a NanoVue spectrophotometer.

### Genotyping and quality control

The genomic DNA of each specimen was genotyped using an Illumina Ovine SNP 600 BeadChip, which contained 604,715 SNPs that spanned the ovine genome with an average distance of 4.28 kb.

PLINK software (v1.07; http://pngu.mgh.harvard.edu/purcell/plink) was used to control the quality of the X chromosome genotype data. An individual was removed if the call rate was less than 90% or if the sample was a duplicate. An SNP locus was excluded if i) its SNP call rate was less than 90%; ii) its minor allele frequency was less than 0.05; or iii) it did not obey Hardy-Weinberg equilibrium (p value<10^−6^). After quality control, BEAGLE software [14] was used to impute the missing genotypes and infer haplotypes.

To increase the accuracy of CNV inference, the following stringent quality control criteria were applied: i) an individual call rate greater than 95% and a call frequency greater than 90%; ii) a log R ratio (LRR) standard deviation less than 0.30; iii) a B allele frequency (BAF) drift less than 0.01; and iv) a default waviness factor.

### Detection of copy number variations

PennCNV software [15] was employed to detect CNV in only the X chromosome. The PennCNV algorithm incorporated multiple information sources, including the total signal intensity of the LRR, BAF, and population frequency of the B allele (PFB). The LRR and BAF of each SNP for every sample were exported from Illumina GenomeStudio software. The PFB was generated based on the BAF of each SNP marker. Genomic waves were adjusted with the sheep GCmodel file, which was generated by calculating the GC content of 1-Mb genomic regions surrounding each marker (500 kb on each side), after the program argument ‘gcmodel’ was used to adjust the results. According to previous research using high-density SNP chips to detect CNV in humans [16,17], the CNV filter was based on three criteria: i) the CNV must contain 10 or more consecutive SNPs; ii) the length of the CNV must be at least 100K; and iii) the CNV must be present in at least one animal.

Finally, after following the method reported by Redon et al [18], CNVRs were obtained by merging CNVs with overlapping regions that had been identified in all samples.

### Global F_ST_

To better understand the population genetic differentiation among the three breeds studied herein, F_ST_ was used to detect signatures of diversifying selection based on genetic polymorphism data. F_ST_ was calculated as described by MacEachern et al [19]:

(1) $$F_{ST}=\frac{H_{T}-H_{S}}{H_{T}}$$

where *H_T_* represents the expected heterozygosity for the overall total population such that

(2) $$H_{T}=1-\sum \left(p^{2}-q^{2}\right)$$

where *p* and *q* denote the frequencies of alleles A1 and A2 over the total population.

In Equation 2,*H_S_* represents the expected heterozygosities in subpopulations and is calculated as follows:

(3) $$H_{s}=\frac{\sum_{i=1}^{n}H_{{exp}_{i}\times n_{i}}}{N_{Total}}$$

where H *_exp_*_*_i_*_ denotes the expected heterozygosity and *n_i_* denotes the sample size in subpopulation *i*.

### Identifying selection regions on the X chromosome

A boxplot strategy was used to determine the upper and lower threshold values to confirm the F_ST_ outlier values for each SNP locus.

First, the interquartile range (Q) of the F_ST_ empirical distribution on the X chromosome was calculated as follows:

(4) $$\text{Q}=F^{U}+F^{L}$$

where *F^U^* and *F^L^* represent the upper and lower interquartile ranges, respectively. Second, the upper (UL) and lower (LL) threshold values were calculated as follows:

(5) $$\text{UL}=F^{U}+1.5Q$$

(6) $$\text{LL}=F^{L}-1.5Q$$

All values greater than the UL threshold value or less than the LL threshold value were defined as outliers.

### Gene detection and functional analysis

Genes harbored in the previously identified CNVRs and selection regions were obtained from the Ensembl Genes 64 Database using BioMart software based on the *Ovis aries* gene sequence assembly (Oar_v3.1). In the selection region, the outlier or selection footprint was extended approximately 100 kb in the upstream and downstream directions. The Database for Annotation, Visualization and Integrated Discovery (DAVID) (http://david.abcc.ncifcrf.gov/) was used to perform gene ontology (GO) [20] enrichment analysis and Kyoto encyclopedia of gene and genome (KEGG) [21] pathway analysis.

To better understand the functions of genes within the detected CNVRs and selection regions, the *Ovis aries* Ensembl gene IDs were converted to human ortholog Ensembl gene IDs using BioMart because annotation of the sheep genome was limited.

## RESULTS

### Marker information

**Figure 1:**
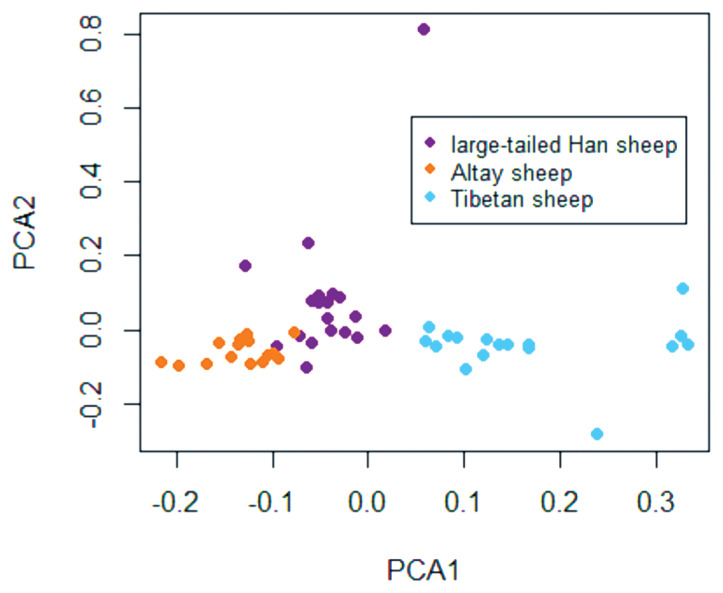
Animal clusters based on a principal component analysis (PCA) using individuals. Plots for the first (PCA1) and second (PCA2) components revealed the clustering of 57 animals, which included large-tailed Han, Altay, and Tibetan sheep.

After genotyping using the Illumina Ovine SNP600K BeadChip, samples with low-quality signal intensities were excluded based on the CNV quality control filter criteria. After quality control and PCA (Figure 1), 20 large-tailed Han ewes, 15 Altay ewes and 17 Tibetan ewes were used in the final analyses. A total of 23295 SNPs was obtained per breed. The average distance between two SNPs was 57.95 kb.

### Genome-wide detection of CNV in sheep

PennCNV software was used to identify the initial numbers of the CNVs and CNVRs in the three selected breeds (Table 1). After merging overlapping CNVs, a total of six, four and 22 CNVRs with lengths of 1.23, 0.93, and 7.02 Mb, respectively, were detected on X chromosomes in large-tailed Han, Altay and Tibetan sheep (Supplementary File: Table S1).

**Figure 2:**
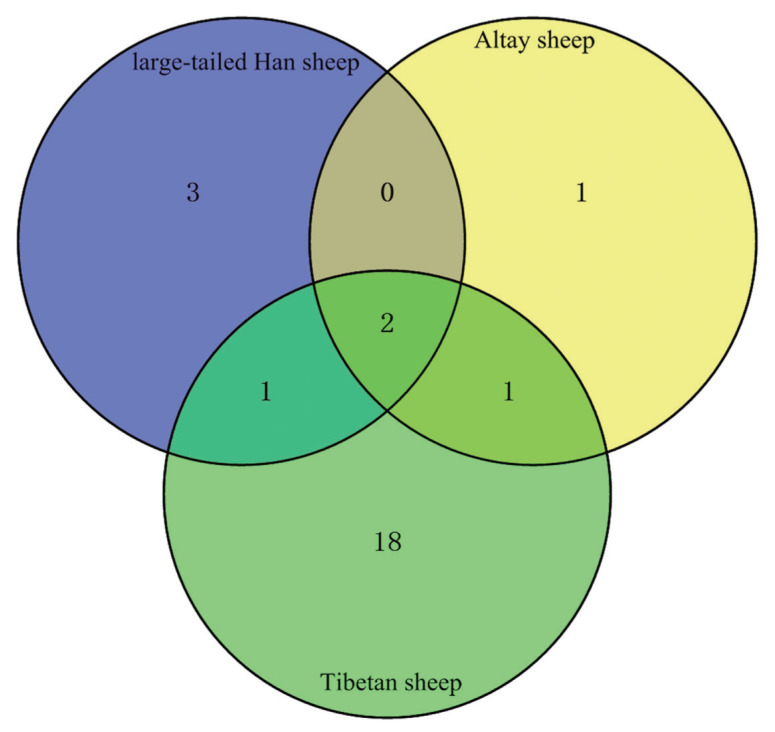
The number of copy number variation regions (CNVRs) identified by PennCNV in three sheep breeds and the number of CNVRs shared among different breeds. Two CNVRs were shared by all three sheep breeds.

Multiple differences were observed in the number of CNVRs among the three breeds. The Tibetan sheep X chromosome contained the most CNVRs, 22, followed by the large-tailed Han sheep X chromosome with six CNVRs and the Altay sheep X chromosome with four CNVRs. Two CNVRs were shared by large-tailed Han sheep and Altay sheep (Figure 2).

**Figure 3:**
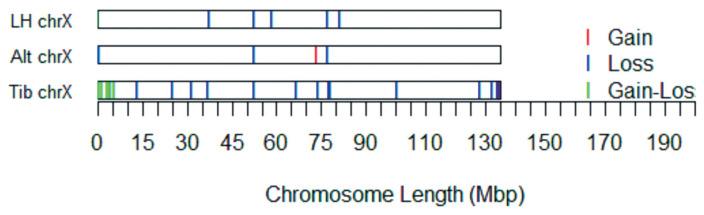
The distribution and status of detected copy number variation regions (CNVRs) in the X chromosome of the three different breeds. For a total of 32 CNVRs, two regions contained gains, 22 regions contained losses, and eight regions contained gains and losses within the same region.

A CNV map of the X chromosome of sheep with different tail types was constructed by removing repetitive CNV regions (Figure 3).

Among these 32 CNVRs, two were gains, 22 were losses, and eight were losses and gains within the same region. The length of the CNVRs ranged from 114.66 to 1,334.87 kb, with an average length of 287.03 kb and a median length of 183.30 kb.

### Empirical distribution of F_ST_ statistics

**Figure 4:**
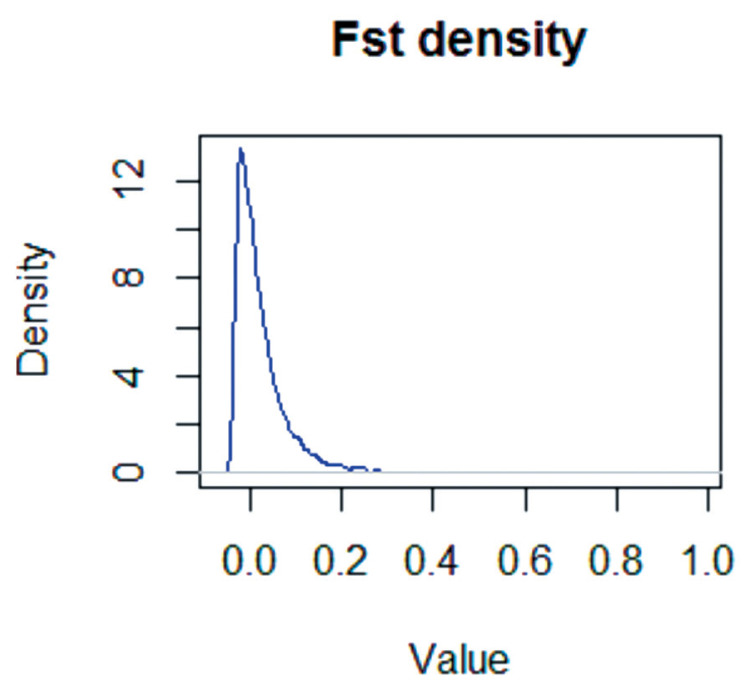
The empirical distribution of F_ST_ test statistical values for the X chromosome in large-tailed Han, Altay and Tibetan ewe populations. The F_ST_ values are normally distributed.

The empirical distributions of the global F_ST_ statistics were determined for the X chromosomes of large-tailed Han, Altay, and Tibetan sheep (Figure 4). The standardized F_ST_ values generally followed a standard normal distribution.

### Selection signature and regions detected using global F_ST_

**Figure 5:**
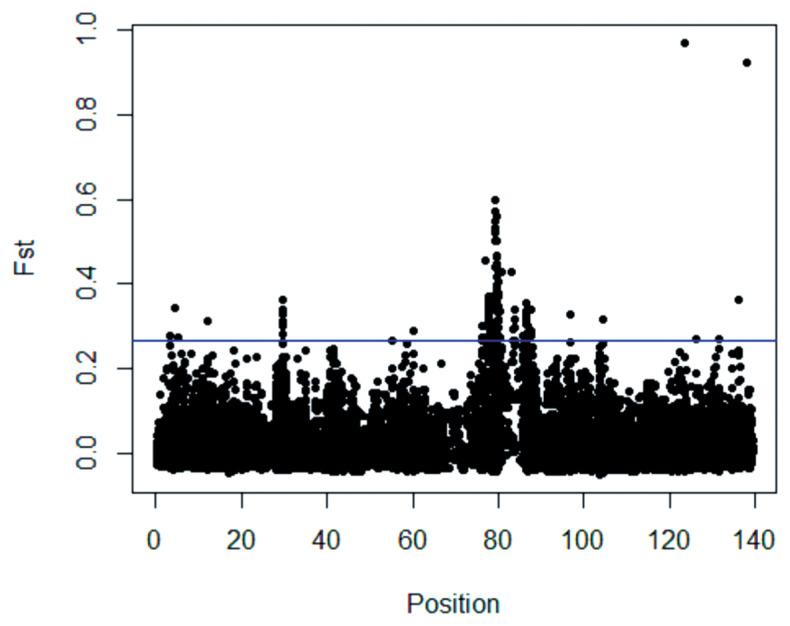
The X chromosome distribution of F_ST_ values. The blue line represents the threshold, and values above the blue line are significantly different from those below the line.

Scanning of the X chromosome for selection signatures in the three sheep breeds was conducted using global F_ST_ to detect the population genetic differentiation at each genetic marker. (Supplementary File: Table S2) reports the true values for each SNP. The boxplot method was used to identify outliers in the genome-wide selection signal because the F_ST_ empirical distribution of a single site was similar to a chi-squared (*χ*^2^) distribution with two or three degrees of freedom [22]. Several obvious genomic regions with high F_ST_ values were detected (Figure 5). Using the F_ST_ test, 36 candidate selection regions with lengths of 14.11 Mb were identified in large Han, Altay and Tibetan sheep (Supplementary File: Table S2).

### Gene content in CNVRs and selection regions

In large-tailed Han sheep, a total of 65 annotated genes within four CNVRs (66.67%) were identified using the Ensembl Genes 64 Database and the BioMart data management system, while the remaining two CNVRs (33.33%) lacked annotated genes. Within the four CNVRs detected in Altay sheep, four CNVRs (100%) harbored 49 annotated genes. In Tibetan sheep, 51 annotated genes were contained within 15 CNVRs (68.18%), whereas the remaining seven CNVRs (31.81%) lacked annotated genes. In total, 82 common genes were shared among large-tailed Han, Altay and Tibetan sheep. Therefore, a total of 83 genes were identified within the detected CNVRs.

The genes in the selection regions were also identified using the Ensembl Genes 64 Database and the BioMart data management system. Twelve other SNPs with significant F_ST_ values were identified within the selection regions. The genes harbored in the selection regions were identified (Table 2), and, in total, 20 common genes were shared by CNVRs and the selection regions.

To provide insight into the functions of genes in the identified CNVRs and selection regions, GO and KEGG pathway analyses were performed using DAVID. “Human” was selected as the background, which resulted in the identification of 159 corresponding human gene IDs in DAVID. After Bonferroni correction, one GO term was identified as statistically significant (Supplementary File: Tables S3–S4). This significant GO term was primarily involved in sulfuric ester hydrolase activity. KEGG pathway analysis revealed the genes in the identified CNVRs and selection regions were significantly enriched in the following two types of pathways: the Janus kinase-signal transducer and activator of transcription (JAK-STAT) signaling pathway and cytokine-cytokine receptor interaction pathways.

## DISCUSSION

In livestock, CNV is one of the primary contributors to phenotypic variation [23]. Several algorithms for inferring CNVs and identifying selection regions in the genome based on SNP chip data have been developed. The selection signature is an imprint left on the genome. To-date, a series of methods have been developed to identify selection signatures on the genome, including the F_ST_ and integrated haplotype score, among others.

In this study, one algorithm was employed to detect CNV in the X chromosome because a number of studies have indicated the reliability of the PennCNV software for detecting CNV is higher than that of several other algorithms [24]. Although only one algorithm was used to detect CNVs in this study, the following two strict criteria were adopted to reduce the risk of false-positive results: an LRR standard deviation of less than 0.30 and a BAF of 0.01. Jiang identified nine CNVRs in the X chromosome in Holsteins using an Illumina High-Density Bovine SNP BeadChip, which contained 777,692 SNPs [25]. In this study, an Illumina Ovine SNP600 BeadChip was used to identify six, four and 22 CNVRs in fat-tailed, fat-rumped and thin-tailed sheep, respectively. These results could be explained by the higher density of the microarray itself, which might have allowed for the detection of a larger number of CNVRs. A general existing belief is the first wild sheep were thin-tailed sheep, and fat-tailed sheep evolved from these thin-tailed sheep via selection by humans and by nature [26]. Thin-tailed sheep had a larger number of CNVRs in their X chromosomes than either fat-tailed or fat-rumped sheep. Altogether, these results indicated natural and artificial selection for fat deposition in sheep tails could lead to alterations in CNV.

In this study, F_ST_ was used to identify selection signatures on the X chromosome in three different breeds of sheep. Previously, Zhu et al [27] used F_ST_ to identify 49, 34, and 55 candidate selection regions respectively associated with reproduction, the immune system and biosynthetic related pathways in German Mutton, Dorper, and Sunit sheep.

In this study, the BioMart data management system was employed to identify overlapping genes or genes within CNVRs, which resulted in the identification of 3,130 genes. Furthermore, the DAVID Bioinformatics Resources System 6.7 was implemented for GO and KEGG pathway analyses. The GO and KEGG pathway enrichment analyses showed the functions of the genes in the identified CNVRs were involved in biological processes, molecular functions and cellular components, including the regulation of transcription; DNA-templated, cytoplasmic translation; rRNA processing; protein serine/threonine kinase activity; viral nucleocapsids; cytosolic large ribosomal subunits; positive regulation of transcription from the RNA polymerase II promoter; Herpes simplex infection; viral carcinogenesis; and the Ras signaling pathway.

Qiu et al [28] used cDNA microarrays to demonstrate the calcium voltage-gated channel subunit alpha1 F gene was expressed in rat adipose tissue in a well-characterized rat model of high-fat diet- (HFD-) induced obesity. Xu et al [29] reported the serine/arginine-rich specific kinase 3 gene might be an important gene for skeletal muscle development and provided basic molecular information useful for further studies on its roles in porcine skeletal muscle. Xiong et al [30] generated liver-specific *FoxO1/3/4*-knockout mice. After being fed a high-fat diet, wild-type mice developed type 2 diabetes; however, lymphotoxin-knockout-mice remained euglycemic and insulin-sensitive.

## Figures and Tables

**Table 1:** X chromosome characteristics of CNVs and CNVRs in sheep

Breed	Number	Total length (Mb)	Average length (Mb)	Gain	Loss	Both	Percentage (%)
CNVs							
Large-tailed Han sheep	78	27.57	0.35	4	58	16	20.42
Altay sheep	45	9.98	0.22	9	36	0	7.39
Tibetan sheep	28	9.96	0.36	2	19	7	7.37
CNVRs							
Large-tailed Han sheep	6	1.23	0.21	0	5	1	0.91
Altay sheep	4	0.93	0.23	1	3	0	0.69
Tibetan sheep	22	7.02	0.31	1	14	7	5.20

CNVs, copy number variation; CNVRs, CNV regions.

**Table 2:** Selection and candidate genes detected in the three sheep breeds using F_ST_

Item	Position (Mb)	F_ST_ value	Gene name
LH-Alt-Tib	1.09–1.29	0.28	*ZBED1, CD99, GYG2*
	1.49–1.69	0.34	*PRKX*
	4.45–4.65	0.31	*HDHD1, STS*
	15.18–15.79	0.32	*NHS, SCML1, RAI2, MKRN1, BEND2, SCML2*
	35.23–35.43	0.27	*HTA2, SYTL5*
	38.42–38.62	0.29	*DDX3X, NYX*
	51.18–51.56	0.28	*SHROOM4*
	52.97–53.17	0.46	*PCSK1N, ERAS, HDAC6, SUV39H1, WAS*
	53.31–54.17	0.32	*SLC38A5, C1ORF21, ZNF81, ZNF182, SPACA5, CSNK1B, CSNK1A1, UXT, ELK1, CFP, SYN1, TIMP1, ARAF, ZNF41, USP11, CDK16*
	54.74–55.90	0.52	*SLC9A7, CHST7, GMCL1, EDA2R*
	56.02–56.36	0.53	*HNRNPK, MT-ND4L, MT-CO3, MT-CO1, MT-ND1*
	58.43–58.76	0.32	*PJA1*
	59.83–63.07	0.38	*KIF4A, DLG3, TEX11, SLC7A3, RPL35A, FOXO4, IL2RG, MED12, NLGN3, GJB1, ZMYM3, ITGB1BP2, RHOG, TAF1, CFDP2, OGT, POL, CNBP SNX12*
	63.09–63.46	0.36	*SLC16A2, RLIM, RBBP4, KIAA2022*
	63.55–63.86	0.36	*ABCB7*
	64.53–65.24	0.30	*SNRPG, COX7B, MAGT1, ATRX, FGF16*
	65.46–65.66	0.43	*NFU1, CXORF26*
	68.14–68.34	0.43	*HMGN5*
	73.44–73.94	0.28	*BTF3, CHM, DACH2*
	76.77–77.12	0.33	*RPL6, PRDM9, IKBKG, G6PD*
	77.71–78.01	0.32	*SLC6A8, PNCK, FAM58A*
	77.83–78.04	0.32	*ATP2B3, BGN, HAUS7, TREX2, ZNF275*
	79.75–79.99	0.29	*GPR50, HMGB3, CD99L2*
	94.88–95.08	0.33	*MOSPD1*
	108.11–108.31	0.32	*UTP14A, BCORL1, ELF4*
	121.75–121.95	0.97	*NXF3, NGFRAP1*
	123.31–123.51	0.27	*ARHGAP12*
	132.86–133.06	0.36	*POL*
	133.84–134.04	0.92	*NUP133*

LH represents large-tailed Han sheep.

Alt represents Altay sheep, and Tib represents Tibetan sheep.
