# Supplementary material for: Detection of copy number variation and selection signatures on the X chromosome in Chinese indigenous sheep with different types of tail
Source: Asian-Australas J Anim Sci. 2019 Jul 1;33(9):1378–86. doi: 10.5713/ajas.18.0661 (PMC7468164; doi:10.5713/ajas.18.0661)
Supplement: Supplementary file 1 [file ajas-18-0661-suppl.pdf]

Table S1: the detailed features of CNVRs on X chromosome of three breeds

CNVR ID	Chr	Start	End	Numsnp	Length	Cn
1	chrX	9495	1405689	456	202,498	2
2	chrX	37292520	37432302	29	139,783	1
3	chrX	52580137	52779630	34	194,312	1
4	chrX	58617657	58734703	32	117,047	1
5	chrX	77076715	78044138	167	375,183	1
6	chrX	81123573	81325067	58	201,495	1
7	chrX	533388	664073	46	130,686	1
8	chrX	52585319	52779630	33	194,312	1
9	chrX	73666316	74169993	64	493,525	3
10	chrX	77076715	77967079	138	115,178	1
11	chrX	90904	545073	107	114,659	2
12	chrX	577400	1305269	197	627,878	2
13	chrX	1338161	3325159	664	205,578	2
14	chrX	3371455	3804198	151	432,744	2
15	chrX	3943268	4156134	78	212,867	2
16	chrX	4215682	5550549	456	1,334,868	2
17	chrX	5633830	6938524	450	1,304,695	2
18	chrX	13215477	13392472	30	176,996	1
19	chrX	24925306	25050185	34	124,880	1
20	chrX	31305692	31672444	68	366,753	1
21	chrX	37138199	37323944	45	185,746	1
22	chrX	52617262	52779630	26	162,369	1
23	chrX	66837417	67089819	26	252,403	1
24	chrX	73989703	74293892	27	183,295	1
25	chrX	77447214	77663518	37	172,770	1
26	chrX	77818214	78031801	53	213,588	1
27	chrX	1E+08	1.01E+08	26	137,035	1
28	chrX	1.28E+08	1.28E+08	18	121,927	1
29	chrX	1.32E+08	1.33E+08	23	115,204	1
30	chrX	1.34E+08	1.34E+08	30	177,790	1
31	chrX	1.34E+08	1.35E+08	41	180,719	3
32	chrX	1.35E+08	1.35E+08	39	216,048	1

Table S3 Significant GO terms associated with genes in CNVRs and selection re

Molecular Function	GO name	Count	P value	Benjamini
GO:0008484	**sulfuric ester hydrol**	5	1.60E-05	5.50E-03

Table S4 KEGG pathway associated with genes in CNVRs and selection regions

Catagory	Term	Count	P-Value	Benjamin
KEGG_ATHWAY	Jak-STAT signaling pathway	4	1.90E-01	9.90E-01
KEGG_PATHWAY	Cytokine-cytokine receptor interacti	5	2.60E-01	9.50E-01

Tbale S2 each SNP Fst value

chr	SNP name	pos	Fst value
27	oar3_OAR)	9495	0.000878
27	oar3_OAR)	18543	-0.02133
27	oar3_OAR)	22190	0.027226

27	oar3_OAR〉	27700	0.027226
27	oar3_OAR〉	27795	-0.0141
27	oar3_OAR〉	40288	0.040509
27	oar3_OAR〉	40458	0.027226
27	oar3_OAR〉	44708	0.011082
27	oar3_OAR〉	51142	0.006016
27	oar3_OAR〉	53429	-0.01171
27	oar3_OAR〉	55118	-0.01193
27	oar3_OAR〉	62778	0.008666
27	oar3_OAR〉	66419	-0.00979
27	oar3_OAR〉	66526	0.015856
27	oar3_OAR〉	79940	0.005184
27	oar3_OAR〉	82020	0.000889
27	oar3_OAR〉	82133	-0.02674
27	oar3_OAR〉	90904	-0.02254
27	oar3_OAR〉	90997	0.027248
27	oar3_OAR〉	92194	-0.02834
27	oar3_OAR〉	92384	-0.02674
27	oar3_OAR〉	93356	-0.0179
27	oar3_OAR〉	99221	-0.02681
27	oar3_OAR〉	100788	-0.02254
27	oar3_OAR〉	101981	0.013682
27	oar3_OAR〉	102023	-0.00295
27	oar3_OAR〉	105290	0.025222
27	oar3_OAR〉	110724	-0.03012
27	s05401.1	112763	-0.02732
27	oar3_OAR〉	114707	-0.02358
27	oar3_OAR〉	117239	0.039131
27	oar3_OAR〉	120002	0.021766
27	oar3_OAR〉	120336	0.010407
27	oar3_OAR〉	122486	-0.0106
27	oar3_OAR〉	125834	-0.01715
27	oar3_OAR〉	127691	-0.00252
27	oar3_OAR〉	132085	-0.02664
27	oar3_OAR〉	133333	-0.02664
27	oar3_OAR〉	133395	-0.02664
27	oar3_OAR〉	137962	-0.00928
27	oar3_OAR〉	138718	0.023067
27	oar3_OAR〉	147971	-0.00252
27	oar3_OAR〉	148901	0.052671
27	oar3_OAR〉	149291	-0.00252
27	oar3_OAR〉	156473	-0.02839
27	oar3_OAR〉	157160	0.055673
27	oar3_OAR〉	162377	-0.01869
27	oar3_OAR〉	168280	0.056205
27	oar3_OAR〉	171119	0.048966
27	oar3_OAR〉	173227	-0.00169
27	oar3_OAR〉	173798	0.009622
27	oar3_OAR〉	176317	-0.02697
27	oar3_OAR〉	192198	-0.003
27	oar3_OAR〉	192772	-0.003
27	oar3_OAR〉	196395	-0.00217
27	oar3_OAR〉	198721	0.071872
27	oar3_OAR〉	200632	0.005255

27	oar3_OAR›	205264	-0.01673
27	oar3_OAR›	209201	-0.02324
27	oar3_OAR›	217035	0.016916
27	oar3_OAR›	223170	0.006173
27	oar3_OAR›	227092	-0.01574
27	oar3_OAR›	228184	0.041643
27	oar3_OAR›	234061	-0.02184
27	oar3_OAR›	240773	-0.02333
27	oar3_OAR›	241626	0.002868
27	oar3_OAR›	247130	0.021632
27	oar3_OAR›	247827	-0.01617
27	oar3_OAR›	253727	0.046998
27	oar3_OAR›	259261	-0.01617
27	oar3_OAR›	259715	-0.02481
27	oar3_OAR›	260479	-0.01255
27	oar3_OAR›	265192	0.013414
27	oar3_OAR›	270137	0.00983
27	oar3_OAR›	271583	0.013414
27	oar3_OAR›	272828	0.02268
27	oar3_OAR›	276521	-0.01567
27	oar3_OAR›	284559	-0.00057
27	oar3_OAR›	285672	-0.02162
27	oar3_OAR›	287738	-0.02055
27	oar3_OAR›	288214	-0.02055
27	oar3_OAR›	298187	0.000704
27	oar3_OAR›	298807	0.042842
27	oar3_OAR›	299352	0.028976
27	oar3_OAR›	301722	-0.00817
27	oar3_OAR›	319455	-0.01942
27	oar3_OAR›	320732	0.015732
27	oar3_OAR›	320746	-0.03059
27	oar3_OAR›	323377	-0.00383
27	oar3_OAR›	325565	-0.00319
27	oar3_OAR›	328200	0.005727
27	oar3_OAR›	331050	-0.01942
27	oar3_OAR›	337378	0.026817
27	oar3_OAR›	339220	0.138792
27	oar3_OAR›	345594	-0.02009
27	oar3_OAR›	346241	-0.01793
27	oar3_OAR›	352101	-0.02797
27	oar3_OAR›	352407	0.001648
27	oar3_OAR›	357737	-0.027
27	oar3_OAR›	360548	7.84E-05
27	oar3_OAR›	364123	-0.02234
27	oar3_OAR›	367133	0.006109
27	oar3_OAR›	373169	0.00609
27	oar3_OAR›	385354	0.01464
27	oar3_OAR›	386039	0.018804
27	oar3_OAR›	386407	-0.03114
27	oar3_OAR›	393371	-0.02716
27	oar3_OAR›	394103	0.037224
27	oar3_OAR›	398082	-0.01423
27	oar3_OAR›	398146	0.015142
27	oar3_OAR›	399003	0.008362

27	oar3_OAR›	399808	-0.01758
27	oar3_OAR›	400074	0.055489
27	oar3_OAR›	405702	0.025383
27	oar3_OAR›	409398	-0.00421
27	oar3_OAR›	412845	-0.00421
27	oar3_OAR›	416769	-0.00606
27	oar3_OAR›	417324	-0.01126
27	oar3_OAR›	421986	-0.02363
27	oar3_OAR›	425547	0.034177
27	oar3_OAR›	427510	0.021004
27	oar3_OAR›	434008	0.072706
27	oar3_OAR›	438339	-0.01253
27	oar3_OAR›	445350	0.008296
27	oar3_OAR›	451168	-0.02485
27	oar3_OAR›	456331	0.020878
27	oar3_OAR›	458031	0.029263
27	oar3_OAR›	465980	0.002868
27	oar3_OAR›	471840	-0.00105
27	oar3_OAR›	476888	-0.0133
27	oar3_OAR›	478042	0.065533
27	oar3_OAR›	480921	0.065533
27	oar3_OAR›	481088	0.067526
27	oar3_OAR›	487866	0.007726
27	oar3_OAR›	490381	-0.02236
27	oar3_OAR›	494334	-0.01291
27	oar3_OAR›	494393	-0.02744
27	oar3_OAR›	510187	-0.01226
27	oar3_OAR›	524600	-0.03109
27	oar3_OAR›	527858	-0.02184
27	oar3_OAR›	528386	-0.0185
27	oar3_OAR›	528554	-0.00325
27	oar3_OAR›	534720	########
27	oar3_OAR›	536456	0.011067
27	oar3_OAR›	536604	0.001049
27	oar3_OAR›	545018	-0.01536
27	oar3_OAR›	545073	########
27	oar3_OAR›	549782	0.025222
27	oar3_OAR›	553482	-0.03351
27	oar3_OAR›	558881	-0.02044
27	oar3_OAR›	560739	0.042263
27	oar3_OAR›	565691	-0.02184
27	oar3_OAR›	569793	0.02374
27	oar3_OAR›	572250	-0.00229
27	oar3_OAR›	572839	0.052227
27	oar3_OAR›	577400	0.022804
27	oar3_OAR›	583825	-0.01464
27	oar3_OAR›	596790	0.018473
27	oar3_OAR›	599084	0.062852
27	oar3_OAR›	602997	-0.02256
27	oar3_OAR›	604874	-0.02256
27	oar3_OAR›	609045	0.023651
27	oar3_OAR›	610532	-0.00712
27	oar3_OAR›	613106	0.176679
27	oar3_OAR›	619549	0.108292

27	oar3_OAR〉	627823	0.048256
27	oar3_OAR〉	627888	-0.0168
27	oar3_OAR〉	633259	-0.01193
27	oar3_OAR〉	634702	-0.027
27	oar3_OAR〉	649448	0.044847
27	oar3_OAR〉	656209	0.005869
27	s45612.1	660724	-0.02165
27	oar3_OAR〉	661758	0.053792
27	oar3_OAR〉	664073	-0.0206
27	oar3_OAR〉	671036	0.07481
27	oar3_OAR〉	672635	0.015158
27	oar3_OAR〉	677392	-0.0186
27	oar3_OAR〉	677525	-0.0186
27	oar3_OAR〉	677540	-0.0186
27	oar3_OAR〉	677717	-0.0186
27	oar3_OAR〉	682930	0.080788
27	oar3_OAR〉	683312	-0.01567
27	oar3_OAR〉	685871	0.039485
27	oar3_OAR〉	686845	0.004609
27	oar3_OAR〉	689904	-0.0299
27	oar3_OAR〉	689942	-0.02448
27	oar3_OAR〉	694861	-0.01567
27	oar3_OAR〉	698471	0.101476
27	oar3_OAR〉	701543	-0.01731
27	oar3_OAR〉	708639	-0.01673
27	oar3_OAR〉	714859	0.007711
27	oar3_OAR〉	729156	-0.0135
27	oar3_OAR〉	729213	-0.0014
27	oar3_OAR〉	738228	-0.0135
27	oar3_OAR〉	746436	0.035683
27	oar3_OAR〉	748423	0.043755
27	oar3_OAR〉	748814	0.046456
27	oar3_OAR〉	749064	0.099485
27	oar3_OAR〉	749332	0.047015
27	oar3_OAR〉	768170	-0.02759
27	oar3_OAR〉	771341	-0.00517
27	oar3_OAR〉	771400	-0.00517
27	s60795.1	776080	-0.01745
27	oar3_OAR〉	781990	0.019974
27	oar3_OAR〉	785343	0.027365
27	oar3_OAR〉	786922	0.050808
27	oar3_OAR〉	792007	-0.02426
27	oar3_OAR〉	793570	-0.01202
27	oar3_OAR〉	801085	-0.02348
27	oar3_OAR〉	805701	0.022519
27	oar3_OAR〉	806739	0.027721
27	oar3_OAR〉	809013	-0.03221
27	oar3_OAR〉	812848	-0.00941
27	oar3_OAR〉	820844	-0.00958
27	oar3_OAR〉	820925	-0.00958
27	oar3_OAR〉	825941	-0.00605
27	oar3_OAR〉	829902	-0.02766
27	oar3_OAR〉	832709	-0.01304
27	oar3_OAR〉	841501	-0.02254

27	oar3_OAR〉	847201	-0.00428
27	oar3_OAR〉	848369	0.03586
27	oar3_OAR〉	863950	-0.02323
27	oar3_OAR〉	865596	-0.01799
27	oar3_OAR〉	878542	0.043822
27	oar3_OAR〉	879285	0.025177
27	oar3_OAR〉	881128	0.012458
27	oar3_OAR〉	882015	-0.01106
27	oar3_OAR〉	887911	-0.02205
27	oar3_OAR〉	892544	-0.02597
27	oar3_OAR〉	893874	-0.01127
27	oar3_OAR〉	900645	0.013007
27	oar3_OAR〉	904665	0.03735
27	oar3_OAR〉	911550	0.000363
27	oar3_OAR〉	915250	-0.03267
27	oar3_OAR〉	918319	-0.00707
27	oar3_OAR〉	922101	0.002868
27	oar3_OAR〉	928415	0.008291
27	oar3_OAR〉	928480	-0.00597
27	oar3_OAR〉	928593	0.198601
27	oar3_OAR〉	928645	0.196812
27	oar3_OAR〉	935573	0.011067
27	oar3_OAR〉	937381	0.009213
27	oar3_OAR〉	942116	0.039481
27	oar3_OAR〉	945200	-0.02416
27	oar3_OAR〉	950438	-0.00195
27	oar3_OAR〉	954695	-0.01565
27	oar3_OAR〉	954746	0.013577
27	oar3_OAR〉	955399	0.07223
27	oar3_OAR〉	965599	0.044155
27	oar3_OAR〉	977978	-0.00033
27	oar3_OAR〉	978755	0.000215
27	oar3_OAR〉	979501	0.057936
27	oar3_OAR〉	982369	0.057936
27	oar3_OAR〉	987289	0.016028
27	oar3_OAR〉	990783	0.029244
27	oar3_OAR〉	992849	-0.00047
27	oar3_OAR〉	993071	0.034323
27	oar3_OAR〉	995768	0.16496
27	oar3_OAR〉	1001760	-0.00148
27	oar3_OAR〉	1001994	0.041586
27	oar3_OAR〉	1006815	0.056789
27	oar3_OAR〉	1007196	0.04816
27	oar3_OAR〉	1011284	-0.02535
27	oar3_OAR〉	1012962	-0.00423
27	oar3_OAR〉	1013016	0.023934
27	oar3_OAR〉	1023508	-0.03142
27	oar3_OAR〉	1023863	-0.02916
27	oar3_OAR〉	1027257	0.014031
27	oar3_OAR〉	1029943	-0.03168
27	oar3_OAR〉	1036963	-0.01356
27	oar3_OAR〉	1044116	-0.00462
27	oar3_OAR〉	1044415	0.027805
27	oar3_OAR〉	1046899	0.006637

27	oar3_OAR〉	1050337	0.041494
27	oar3_OAR〉	1061961	0.004461
27	oar3_OAR〉	1068752	0.010252
27	oar3_OAR〉	1072498	-0.02578
27	oar3_OAR〉	1077207	-0.01871
27	oar3_OAR〉	1078326	0.037451
27	oar3_OAR〉	1083434	0.047282
27	oar3_OAR〉	1085890	0.015168
27	oar3_OAR〉	1086358	-0.02926
27	oar3_OAR〉	1089047	0.046207
27	oar3_OAR〉	1094387	-0.03074
27	oar3_OAR〉	1100466	0.083601
27	oar3_OAR〉	1100778	-0.01994
27	oar3_OAR〉	1111430	-0.01284
27	oar3_OAR〉	1113032	0.009084
27	oar3_OAR〉	1116128	0.017461
27	oar3_OAR〉	1124388	-0.00606
27	oar3_OAR〉	1132276	0.084285
27	oar3_OAR〉	1132382	-0.03504
27	oar3_OAR〉	1133456	-0.02453
27	oar3_OAR〉	1140764	0.043941
27	oar3_OAR〉	1143953	-0.02358
27	oar3_OAR〉	1146926	0.000889
27	oar3_OAR〉	1148611	-0.01317
27	oar3_OAR〉	1150451	-0.03031
27	oar3_OAR〉	1154628	0.0105
27	oar3_OAR〉	1157103	-0.0113
27	oar3_OAR〉	1161041	0.075568
27	oar3_OAR〉	1166491	0.155955
27	oar3_OAR〉	1173509	0.017152
27	oar3_OAR〉	1179064	-0.01737
27	oar3_OAR〉	1181266	-0.00288
27	oar3_OAR〉	1194876	0.276161
27	oar3_OAR〉	1195393	0.113535
27	oar3_OAR〉	1200477	0.255605
27	oar3_OAR〉	1211320	0.015137
27	oar3_OAR〉	1212317	0.034945
27	oar3_OAR〉	1213319	-0.02701
27	oar3_OAR〉	1219380	0.059918
27	oar3_OAR〉	1221664	-0.00271
27	oar3_OAR〉	1222880	-0.03485
27	oar3_OAR〉	1224402	-0.0244
27	oar3_OAR〉	1229602	-0.0246
27	oar3_OAR〉	1234392	-0.03591
27	oar3_OAR〉	1235503	-0.02759
27	oar3_OAR〉	1235767	-0.02491
27	oar3_OAR〉	1238668	-0.01489
27	oar3_OAR〉	1241508	-0.02036
27	oar3_OAR〉	1254911	0.007434
27	oar3_OAR〉	1255052	0.015243
27	oar3_OAR〉	1260040	0.08161
27	oar3_OAR〉	1261992	0.122322
27	oar3_OAR〉	1263802	0.122322
27	oar3_OAR〉	1267237	-0.00308

27	oar3_OAR〉	1267694	0.014386
27	oar3_OAR〉	1272048	-0.00501
27	oar3_OAR〉	1273409	0.230051
27	oar3_OAR〉	1276122	0.01236
27	oar3_OAR〉	1281864	0.029278
27	oar3_OAR〉	1283005	0.032551
27	oar3_OAR〉	1285019	0.038703
27	oar3_OAR〉	1285855	0.078248
27	oar3_OAR〉	1290480	0.092269
27	oar3_OAR〉	1305269	0.003949
27	oar3_OAR〉	1308297	########
27	oar3_OAR〉	1308901	0.101098
27	oar3_OAR〉	1311780	0.082283
27	oar3_OAR〉	1318778	0.03675
27	oar3_OAR〉	1318840	0.016711
27	oar3_OAR〉	1322772	0.046641
27	oar3_OAR〉	1323458	0.066139
27	oar3_OAR〉	1323596	-0.0065
27	oar3_OAR〉	1324671	0.096913
27	oar3_OAR〉	1333708	-0.00965
27	oar3_OAR〉	1335211	-0.02086
27	oar3_OAR〉	1338161	0.127662
27	oar3_OAR〉	1338993	0.047416
27	oar3_OAR〉	1342413	-0.00987
27	oar3_OAR〉	1347554	0.035932
27	oar3_OAR〉	1349305	0.050776
27	oar3_OAR〉	1352039	0.005869
27	oar3_OAR〉	1352935	-0.0197
27	oar3_OAR〉	1356800	########
27	oar3_OAR〉	1361211	0.052511
27	oar3_OAR〉	1361270	0.046507
27	oar3_OAR〉	1363219	0.069984
27	oar3_OAR〉	1365162	0.024562
27	oar3_OAR〉	1372396	0.043599
27	oar3_OAR〉	1372627	-0.00105
27	oar3_OAR〉	1373713	0.028857
27	oar3_OAR〉	1377334	0.161516
27	oar3_OAR〉	1379609	0.017753
27	oar3_OAR〉	1384050	0.02459
27	oar3_OAR〉	1384058	0.0629
27	oar3_OAR〉	1385947	-0.01127
27	oar3_OAR〉	1391923	0.07373
27	oar3_OAR〉	1394772	0.075764
27	oar3_OAR〉	1396000	0.041796
27	oar3_OAR〉	1398430	0.076679
27	oar3_OAR〉	1399730	0.20869
27	oar3_OAR〉	1405689	0.011345
27	oar3_OAR〉	1406758	NA
27	oar3_OAR〉	1406867	-0.02345
27	oar3_OAR〉	1413440	-0.00349
27	oar3_OAR〉	1418138	-0.03024
27	oar3_OAR〉	1418214	-0.0381
27	oar3_OAR〉	1420558	0.038843
27	oar3_OAR〉	1424137	0.001514

27	oar3_OAR〉	1429707	-0.02139
27	oar3_OAR〉	1430999	-0.02662
27	oar3_OAR〉	1431722	0.012371
27	oar3_OAR〉	1436921	0.005349
27	oar3_OAR〉	1443113	-0.00502
27	oar3_OAR〉	1446032	-0.03189
27	oar3_OAR〉	1447451	NA
27	oar3_OAR〉	1447690	0.004105
27	oar3_OAR〉	1451528	-0.01097
27	oar3_OAR〉	1460273	-0.00064
27	oar3_OAR〉	1462050	0.021081
27	oar3_OAR〉	1463020	0.105061
27	oar3_OAR〉	1463720	0.123358
27	oar3_OAR〉	1463792	0.123358
27	oar3_OAR〉	1464489	0.105061
27	oar3_OAR〉	1468157	0.13913
27	oar3_OAR〉	1474239	0.197148
27	oar3_OAR〉	1474820	0.039013
27	oar3_OAR〉	1479000	0.010783
27	oar3_OAR〉	1480357	0.018804
27	oar3_OAR〉	1486702	0.00462
27	oar3_OAR〉	1493742	-0.00508
27	oar3_OAR〉	1500196	0.114012
27	oar3_OAR〉	1501650	0.004368
27	oar3_OAR〉	1502031	-0.00516
27	oar3_OAR〉	1502120	-0.02133
27	oar3_OAR〉	1505786	0.029531
27	oar3_OAR〉	1508648	0.047897
27	oar3_OAR〉	1510536	0.08546
27	oar3_OAR〉	1513779	0.06582
27	oar3_OAR〉	1514389	0.161195
27	oar3_OAR〉	1519090	0.016797
27	oar3_OAR〉	1525690	-0.02393
27	oar3_OAR〉	1529930	0.039745
27	oar3_OAR〉	1535146	-0.01868
27	oar3_OAR〉	1542576	0.01939
27	oar3_OAR〉	1544530	-0.01069
27	oar3_OAR〉	1547738	-0.01069
27	oar3_OAR〉	1561311	-0.02593
27	oar3_OAR〉	1562498	0.01889
27	oar3_OAR〉	1572522	0.049957
27	oar3_OAR〉	1577371	0.120868
27	oar3_OAR〉	1583560	-0.01512
27	oar3_OAR〉	1584581	0.079352
27	oar3_OAR〉	1585581	0.341626
27	oar3_OAR〉	1585724	0.151731
27	oar3_OAR〉	1591292	0.11962
27	oar3_OAR〉	1598239	0.12112
27	oar3_OAR〉	1598249	0.043647
27	oar3_OAR〉	1598322	0.096188
27	oar3_OAR〉	1604783	0.172025
27	oar3_OAR〉	1610556	0.071634
27	oar3_OAR〉	1611438	0.032494
27	oar3_OAR〉	1615639	0.007039

27	oar3_OAR〉	1616215	-0.01271
27	oar3_OAR〉	1620847	-0.02057
27	oar3_OAR〉	1624794	0.056752
27	oar3_OAR〉	1625049	0.007039
27	oar3_OAR〉	1631873	-0.01356
27	oar3_OAR〉	1631980	0.006183
27	oar3_OAR〉	1633165	0.044545
27	oar3_OAR〉	1634879	-0.02866
27	oar3_OAR〉	1639610	-0.01007
27	oar3_OAR〉	1642360	-0.02666
27	oar3_OAR〉	1645560	0.004356
27	oar3_OAR〉	1649983	0.06176
27	oar3_OAR〉	1653588	0.066132
27	oar3_OAR〉	1654715	0.046641
27	oar3_OAR〉	1658036	0.074647
27	oar3_OAR〉	1662878	-0.00623
27	oar3_OAR〉	1665410	0.084506
27	oar3_OAR〉	1669827	0.021556
27	oar3_OAR〉	1670546	-0.02672
27	oar3_OAR〉	1676285	-0.02223
27	oar3_OAR〉	1677365	-0.02786
27	oar3_OAR〉	1677573	-0.0148
27	oar3_OAR〉	1680391	-0.01314
27	oar3_OAR〉	1693477	-0.01666
27	oar3_OAR〉	1695351	-0.00929
27	oar3_OAR〉	1698474	0.009985
27	oar3_OAR〉	1698530	0.018457
27	oar3_OAR〉	1705549	-0.02072
27	oar3_OAR〉	1708799	0.015729
27	oar3_OAR〉	1709975	0.04287
27	oar3_OAR〉	1713814	0.038982
27	oar3_OAR〉	1720064	0.006819
27	oar3_OAR〉	1720170	0.061355
27	oar3_OAR〉	1723312	0.090649
27	oar3_OAR〉	1728334	0.01861
27	oar3_OAR〉	1728526	0.006628
27	oar3_OAR〉	1734312	0.003164
27	oar3_OAR〉	1740553	0.000214
27	oar3_OAR〉	1745328	0.091203
27	oar3_OAR〉	1747600	0.082222
27	oar3_OAR〉	1749241	-0.00894
27	oar3_OAR〉	1753287	-0.02131
27	oar3_OAR〉	1754903	0.042816
27	oar3_OAR〉	1759556	0.000889
27	oar3_OAR〉	1762561	0.019245
27	oar3_OAR〉	1766966	-0.0078
27	oar3_OAR〉	1767024	0.001611
27	oar3_OAR〉	1779771	-0.01538
27	oar3_OAR〉	1781881	-0.02943
27	oar3_OAR〉	1786620	0.035652
27	oar3_OAR〉	1791346	0.056528
27	oar3_OAR〉	1791451	0.056528
27	oar3_OAR〉	1793652	0.016406
27	oar3_OAR〉	1796813	0.008723

27	oar3_OAR›	1796884	0.010734
27	oar3_OAR›	1802554	0.040956
27	oar3_OAR›	1805435	-0.02411
27	oar3_OAR›	1816038	0.01024
27	oar3_OAR›	1821121	0.049372
27	oar3_OAR›	1824605	0.007218
27	oar3_OAR›	1824669	-0.02759
27	oar3_OAR›	1834708	0.046159
27	oar3_OAR›	1834882	-0.00034
27	oar3_OAR›	1837800	0.003335
27	oar3_OAR›	1842007	-0.01611
27	oar3_OAR›	1844220	-0.02699
27	oar3_OAR›	1845610	-0.02213
27	oar3_OAR›	1847445	-0.02447
27	oar3_OAR›	1854870	0.083355
27	oar3_OAR›	1856754	0.045327
27	oar3_OAR›	1859957	-0.00673
27	oar3_OAR›	1860230	0.029081
27	oar3_OAR›	1863592	0.000738
27	oar3_OAR›	1865567	0.018202
27	oar3_OAR›	1867022	-0.03382
27	oar3_OAR›	1871619	-0.00065
27	oar3_OAR›	1872276	-0.00698
27	oar3_OAR›	1874559	0.050043
27	oar3_OAR›	1880432	-0.00916
27	oar3_OAR›	1880494	0.0082
27	oar3_OAR›	1887009	0.066977
27	oar3_OAR›	1887021	-0.0074
27	oar3_OAR›	1892373	0.066977
27	oar3_OAR›	1892402	0.076064
27	oar3_OAR›	1899587	0.009129
27	oar3_OAR›	1899846	-0.02036
27	oar3_OAR›	1899990	0.061513
27	oar3_OAR›	1905100	0.036462
27	oar3_OAR›	1905239	0.064341
27	oar3_OAR›	1910054	0.165497
27	oar3_OAR›	1910250	0.044645
27	oar3_OAR›	1915851	-0.00973
27	oar3_OAR›	1915905	0.097328
27	oar3_OAR›	1916160	0.272325
27	oar3_OAR›	1921042	0.02374
27	oar3_OAR›	1925641	0.03995
27	oar3_OAR›	1927253	0.037217
27	oar3_OAR›	1928569	0.151249
27	oar3_OAR›	1933987	0.090115
27	oar3_OAR›	1934158	-0.01953
27	oar3_OAR›	1941050	0.039481
27	oar3_OAR›	1941583	0.182112
27	oar3_OAR›	1947356	0.053034
27	oar3_OAR›	1947566	0.053034
27	oar3_OAR›	1949808	0.139981
27	oar3_OAR›	1955017	-0.00606
27	oar3_OAR›	1958293	-0.02353
27	oar3_OAR›	1960355	0.127611

27	oar3_OAR〉	1965989	-0.01637
27	oar3_OAR〉	1971900	-0.0085
27	oar3_OAR〉	1974412	-0.01637
27	oar3_OAR〉	1977988	-0.02662
27	oar3_OAR〉	1980996	-0.01605
27	oar3_OAR〉	1983830	0.018532
27	oar3_OAR〉	1986241	-0.01327
27	oar3_OAR〉	1988392	-0.02324
27	oar3_OAR〉	1990501	0.021533
27	oar3_OAR〉	1995345	-0.00122
27	oar3_OAR〉	1999244	0.018406
27	oar3_OAR〉	2000079	0.015497
27	oar3_OAR〉	2000138	-0.03158
27	oar3_OAR〉	2005428	-0.03158
27	oar3_OAR〉	2006289	0.062909
27	oar3_OAR〉	2010061	0.009892
27	oar3_OAR〉	2015059	0.039026
27	oar3_OAR〉	2017115	-0.01232
27	oar3_OAR〉	2018116	-0.00673
27	oar3_OAR〉	2021912	-0.01921
27	oar3_OAR〉	2026231	0.082222
27	oar3_OAR〉	2029969	0.104729
27	oar3_OAR〉	2030767	0.033433
27	oar3_OAR〉	2032722	0.001047
27	oar3_OAR〉	2039460	-0.00448
27	oar3_OAR〉	2041284	0.053462
27	oar3_OAR〉	2042880	-0.03668
27	oar3_OAR〉	2044957	-0.01851
27	oar3_OAR〉	2052947	0.071313
27	oar3_OAR〉	2053299	0.058687
27	oar3_OAR〉	2061154	0.069779
27	oar3_OAR〉	2064662	-0.01695
27	oar3_OAR〉	2101632	0.04762
27	oar3_OAR〉	2104469	0.104461
27	oar3_OAR〉	2106950	0.011328
27	oar3_OAR〉	2109068	0.011643
27	oar3_OAR〉	2114016	0.235215
27	oar3_OAR〉	2116334	-0.01197
27	oar3_OAR〉	2116439	-0.00032
27	oar3_OAR〉	2120173	0.073246
27	oar3_OAR〉	2124760	0.013414
27	oar3_OAR〉	2130241	-0.01423
27	oar3_OAR〉	2130507	-0.00592
27	oar3_OAR〉	2134821	0.023023
27	oar3_OAR〉	2138258	0.046758
27	oar3_OAR〉	2139724	-0.00797
27	oar3_OAR〉	2140715	0.043672
27	oar3_OAR〉	2146625	0.054229
27	oar3_OAR〉	2147105	0.033465
27	oar3_OAR〉	2149268	0.080099
27	oar3_OAR〉	2152607	0.132794
27	oar3_OAR〉	2157689	-0.03679
27	oar3_OAR〉	2157701	0.028803
27	oar3_OAR〉	2159007	0.140969

27	oar3_OAR〉	2161154	0.045535
27	oar3_OAR〉	2163812	-0.03679
27	oar3_OAR〉	2169704	0.088041
27	oar3_OAR〉	2170758	0.029607
27	oar3_OAR〉	2171259	0.003362
27	oar3_OAR〉	2178249	-0.02209
27	oar3_OAR〉	2180176	0.029534
27	oar3_OAR〉	2185334	-0.00712
27	oar3_OAR〉	2191283	0.19697
27	oar3_OAR〉	2192394	-0.00717
27	oar3_OAR〉	2193756	-0.00814
27	oar3_OAR〉	2196699	-0.01375
27	oar3_OAR〉	2197230	0.043041
27	oar3_OAR〉	2204183	-0.02961
27	oar3_OAR〉	2204688	-0.02351
27	oar3_OAR〉	2209332	0.092576
27	oar3_OAR〉	2209652	-0.02902
27	oar3_OAR〉	2209874	0.08014
27	oar3_OAR〉	2215643	0.082057
27	oar3_OAR〉	2215757	-0.03152
27	oar3_OAR〉	2222860	-0.01915
27	oar3_OAR〉	2222899	-0.0014
27	oar3_OAR〉	2223751	0.01826
27	oar3_OAR〉	2226917	-0.01275
27	oar3_OAR〉	2232386	-0.0229
27	oar3_OAR〉	2232410	-0.0313
27	oar3_OAR〉	2233755	-0.01098
27	oar3_OAR〉	2234845	0.015096
27	oar3_OAR〉	2237998	-0.01758
27	oar3_OAR〉	2243183	-0.01491
27	oar3_OAR〉	2243698	0.079455
27	oar3_OAR〉	2246533	-0.00091
27	oar3_OAR〉	2251033	0.078711
27	oar3_OAR〉	2251668	0.002728
27	oar3_OAR〉	2257203	0.073654
27	oar3_OAR〉	2260489	0.122089
27	oar3_OAR〉	2264008	-0.01977
27	oar3_OAR〉	2267763	0.191944
27	oar3_OAR〉	2271887	0.096034
27	oar3_OAR〉	2272060	0.026133
27	oar3_OAR〉	2275902	0.028246
27	oar3_OAR〉	2277511	-0.01967
27	oar3_OAR〉	2281108	0.005086
27	oar3_OAR〉	2283296	0.065153
27	oar3_OAR〉	2288476	-0.03042
27	oar3_OAR〉	2288886	0.000606
27	oar3_OAR〉	2290480	0.174366
27	oar3_OAR〉	2299195	0.039051
27	oar3_OAR〉	2299596	0.019617
27	oar3_OAR〉	2302076	-0.00019
27	oar3_OAR〉	2305143	0.029668
27	oar3_OAR〉	2310672	-0.0079
27	oar3_OAR〉	2313282	-0.00673
27	oar3_OAR〉	2314846	-0.00126

27	oar3_OAR›	2315623	0.081906
27	oar3_OAR›	2323999	0.043637
27	oar3_OAR›	2327700	0.015631
27	oar3_OAR›	2329017	0.016773
27	oar3_OAR›	2330128	0.03419
27	oar3_OAR›	2332990	0.033465
27	oar3_OAR›	2339613	0.046899
27	oar3_OAR›	2342386	0.005381
27	oar3_OAR›	2345802	0.042344
27	oar3_OAR›	2350994	0.042344
27	oar3_OAR›	2352285	-0.02164
27	oar3_OAR›	2360335	0.046852
27	oar3_OAR›	2361040	0.066908
27	oar3_OAR›	2365853	-0.01906
27	oar3_OAR›	2373876	-0.01737
27	oar3_OAR›	2375795	0.010181
27	oar3_OAR›	2377530	-0.01215
27	oar3_OAR›	2377785	0.044232
27	oar3_OAR›	2379767	0.037425
27	oar3_OAR›	2384737	0.121819
27	oar3_OAR›	2389769	0.01345
27	oar3_OAR›	2395946	-0.00462
27	oar3_OAR›	2397175	-0.02104
27	oar3_OAR›	2398691	-0.00932
27	oar3_OAR›	2403233	-0.00383
27	oar3_OAR›	2403307	-0.00383
27	oar3_OAR›	2407634	-0.0269
27	oar3_OAR›	2408959	0.034543
27	oar3_OAR›	2413350	0.033827
27	oar3_OAR›	2414327	0.210784
27	oar3_OAR›	2420136	0.163372
27	oar3_OAR›	2425222	0.163372
27	oar3_OAR›	2439475	0.215584
27	oar3_OAR›	2439821	0.074647
27	oar3_OAR›	2441233	-0.0009
27	oar3_OAR›	2444902	0.054445
27	oar3_OAR›	2451604	-0.00606
27	oar3_OAR›	2452147	0.077863
27	oar3_OAR›	2454280	0.143609
27	oar3_OAR›	2467012	0.102288
27	oar3_OAR›	2468289	0.026045
27	oar3_OAR›	2473864	0.018084
27	oar3_OAR›	2475003	0.002019
27	oar3_OAR›	2477172	0.041937
27	oar3_OAR›	2488577	-0.01082
27	oar3_OAR›	2493178	0.015455
27	oar3_OAR›	2493940	0.08231
27	oar3_OAR›	2503442	0.032739
27	oar3_OAR›	2504518	0.057304
27	oar3_OAR›	2508487	-0.02242
27	oar3_OAR›	2512764	0.002557
27	oar3_OAR›	2517424	0.036226
27	oar3_OAR›	2518900	-0.02209
27	oar3_OAR›	2524388	-0.02588

27	oar3_OAR〉	2524493	-0.02325
27	oar3_OAR〉	2530080	0.022695
27	oar3_OAR〉	2534405	-0.00045
27	oar3_OAR〉	2535955	0.058681
27	oar3_OAR〉	2551192	-0.0126
27	oar3_OAR〉	2554998	0.02501
27	oar3_OAR〉	2565828	-0.02593
27	oar3_OAR〉	2574196	0.010879
27	oar3_OAR〉	2581528	0.008723
27	oar3_OAR〉	2584289	0.014491
27	oar3_OAR〉	2587454	-0.00664
27	oar3_OAR〉	2588810	0.009985
27	oar3_OAR〉	2589374	0.044721
27	oar3_OAR〉	2590357	-0.03152
27	oar3_OAR〉	2592080	0.04569
27	oar3_OAR〉	2594473	-0.00682
27	oar3_OAR〉	2600163	0.055318
27	oar3_OAR〉	2608862	-0.00976
27	oar3_OAR〉	2623945	0.082283
27	oar3_OAR〉	2626996	0.054528
27	oar3_OAR〉	2632237	0.158595
27	oar3_OAR〉	2644459	-0.02208
27	oar3_OAR〉	2648407	0.036047
27	oar3_OAR〉	2649197	0.092269
27	oar3_OAR〉	2654864	-0.02198
27	oar3_OAR〉	2657347	0.009771
27	oar3_OAR〉	2664903	0.009771
27	oar3_OAR〉	2671029	0.175084
27	oar3_OAR〉	2673845	-0.01193
27	oar3_OAR〉	2677411	-0.01995
27	oar3_OAR〉	2678671	0.008008
27	oar3_OAR〉	2685018	0.008411
27	oar3_OAR〉	2685608	-0.01378
27	oar3_OAR〉	2693185	-0.01639
27	oar3_OAR〉	2693996	0.004763
27	oar3_OAR〉	2701662	0.025614
27	oar3_OAR〉	2704322	0.008813
27	oar3_OAR〉	2704686	-0.01061
27	oar3_OAR〉	2721949	0.070593
27	oar3_OAR〉	2730891	-0.00751
27	oar3_OAR〉	2755669	-0.01038
27	oar3_OAR〉	2755678	-0.01897
27	oar3_OAR〉	2761850	-0.00615
27	oar3_OAR〉	2763243	-0.02897
27	oar3_OAR〉	2770050	-0.02395
27	oar3_OAR〉	2770858	-0.02269
27	oar3_OAR〉	2771807	-0.02395
27	oar3_OAR〉	2777511	-0.01356
27	oar3_OAR〉	2782591	-0.0177
27	oar3_OAR〉	2783133	0.003178
27	oar3_OAR〉	2788842	0.009572
27	oar3_OAR〉	2791700	0.021858
27	oar3_OAR〉	2799050	-0.01717
27	oar3_OAR〉	2800197	0.047343

27	oar3_OAR〉	2800315	-0.00213
27	oar3_OAR〉	2808148	0.019413
27	oar3_OAR〉	2808267	-0.00795
27	oar3_OAR〉	2808443	-0.02351
27	oar3_OAR〉	2822154	-0.02545
27	oar3_OAR〉	2822366	-0.02098
27	oar3_OAR〉	2822478	-0.0057
27	oar3_OAR〉	2827378	-0.02788
27	oar3_OAR〉	2827882	-0.01621
27	oar3_OAR〉	2834312	0.000452
27	oar3_OAR〉	2837925	-0.0041
27	oar3_OAR〉	2843293	0.023023
27	oar3_OAR〉	2843485	0.028467
27	oar3_OAR〉	2849628	-0.02308
27	oar3_OAR〉	2849974	0.044706
27	oar3_OAR〉	2854887	-0.01907
27	oar3_OAR〉	2855231	-0.0156
27	oar3_OAR〉	2855579	-0.01649
27	oar3_OAR〉	2856572	0.000363
27	oar3_OAR〉	2861040	-0.02916
27	oar3_OAR〉	2865215	0.00822
27	oar3_OAR〉	2865284	-0.02897
27	oar3_OAR〉	2865374	-0.00589
27	oar3_OAR〉	2876473	-0.01371
27	oar3_OAR〉	2876590	0.038705
27	oar3_OAR〉	2879197	0.099206
27	oar3_OAR〉	2886794	0.007058
27	oar3_OAR〉	2889705	0.048082
27	oar3_OAR〉	2891511	-0.00603
27	oar3_OAR〉	2893819	-0.00961
27	oar3_OAR〉	2895586	0.002868
27	oar3_OAR〉	2903483	0.028445
27	oar3_OAR〉	2903620	0.061587
27	oar3_OAR〉	2905401	0.025934
27	oar3_OAR〉	2910655	0.016012
27	oar3_OAR〉	2912009	-0.00524
27	oar3_OAR〉	2912197	0.046527
27	oar3_OAR〉	2917185	0.067526
27	oar3_OAR〉	2918225	-0.02721
27	oar3_OAR〉	2920568	0.006078
27	oar3_OAR〉	2934918	0.014944
27	oar3_OAR〉	2935964	-0.00043
27	oar3_OAR〉	2936348	-0.0135
27	oar3_OAR〉	2941392	-0.01317
27	oar3_OAR〉	2944204	-0.02549
27	oar3_OAR〉	2945941	0.055255
27	oar3_OAR〉	2945994	0.070091
27	oar3_OAR〉	2947915	-0.0206
27	oar3_OAR〉	2951890	0.100474
27	oar3_OAR〉	2957779	0.055273
27	oar3_OAR〉	2960802	0.031494
27	oar3_OAR〉	2963619	0.004105
27	oar3_OAR〉	2965271	0.062765
27	oar3_OAR〉	2965712	0.049515

27	oar3_OAR〉	2975062	0.022266
27	oar3_OAR〉	2976671	0.009277
27	oar3_OAR〉	2984816	0.004868
27	oar3_OAR〉	2990849	0.235152
27	oar3_OAR〉	2990921	-0.00512
27	oar3_OAR〉	2991042	NA
27	oar3_OAR〉	2993149	0.001424
27	oar3_OAR〉	2996741	-0.02049
27	oar3_OAR〉	2999481	0.04709
27	oar3_OAR〉	3001748	0.102139
27	oar3_OAR〉	3004877	0.042599
27	oar3_OAR〉	3017038	-0.01367
27	oar3_OAR〉	3017056	-0.02883
27	oar3_OAR〉	3022034	-0.01996
27	oar3_OAR〉	3022185	0.02501
27	oar3_OAR〉	3029659	0.018466
27	oar3_OAR〉	3035391	0.023197
27	oar3_OAR〉	3035890	-0.00767
27	oar3_OAR〉	3052915	0.016575
27	oar3_OAR〉	3052969	0.008444
27	oar3_OAR〉	3054016	0.015729
27	oar3_OAR〉	3056242	0.0054
27	oar3_OAR〉	3062276	0.106541
27	oar3_OAR〉	3065601	-0.0172
27	oar3_OAR〉	3068344	0.074465
27	oar3_OAR〉	3069203	0.041494
27	oar3_OAR〉	3072932	-0.01739
27	oar3_OAR〉	3079008	0.02744
27	oar3_OAR〉	3081415	0.040066
27	oar3_OAR〉	3081478	-0.01869
27	oar3_OAR〉	3093229	0.082832
27	oar3_OAR〉	3093326	0.09111
27	oar3_OAR〉	3096396	0.004917
27	oar3_OAR〉	3099312	0.079148
27	oar3_OAR〉	3100096	0.004917
27	oar3_OAR〉	3103962	0.121188
27	oar3_OAR〉	3105983	-0.0347
27	oar3_OAR〉	3108552	-0.0177
27	oar3_OAR〉	3115282	0.02062
27	oar3_OAR〉	3117066	-0.00751
27	oar3_OAR〉	3126151	-0.0284
27	oar3_OAR〉	3126349	0.00222
27	oar3_OAR〉	3131217	0.000134
27	oar3_OAR〉	3136687	0.087044
27	oar3_OAR〉	3137080	0.010252
27	oar3_OAR〉	3143947	-0.03392
27	oar3_OAR〉	3144002	-0.0018
27	oar3_OAR〉	3145877	0.148172
27	oar3_OAR〉	3149892	0.10028
27	oar3_OAR〉	3153213	0.011402
27	oar3_OAR〉	3157992	0.011083
27	oar3_OAR〉	3164365	0.059703
27	oar3_OAR〉	3164750	0.132463
27	oar3_OAR〉	3170186	0.053235

27	oar3_OAR⟩	3173518	-0.02084
27	oar3_OAR⟩	3173704	-0.01203
27	oar3_OAR⟩	3179401	0.014259
27	oar3_OAR⟩	3184601	-0.01872
27	oar3_OAR⟩	3186654	0.01861
27	oar3_OAR⟩	3186822	0.028864
27	oar3_OAR⟩	3190382	0.020697
27	oar3_OAR⟩	3192771	-0.02012
27	oar3_OAR⟩	3195886	0.151221
27	oar3_OAR⟩	3199518	-0.01145
27	oar3_OAR⟩	3206080	0.058521
27	oar3_OAR⟩	3206489	0.008411
27	oar3_OAR⟩	3207890	0.058521
27	oar3_OAR⟩	3212334	0.018027
27	oar3_OAR⟩	3215991	0.013892
27	oar3_OAR⟩	3216801	0.003385
27	oar3_OAR⟩	3217523	0.003385
27	oar3_OAR⟩	3225660	-0.01605
27	oar3_OAR⟩	3240355	-0.03293
27	oar3_OAR⟩	3242089	-0.028
27	oar3_OAR⟩	3247744	0.000343
27	oar3_OAR⟩	3249149	0.038908
27	oar3_OAR⟩	3254069	0.008723
27	oar3_OAR⟩	3263326	-0.02744
27	oar3_OAR⟩	3264391	-0.0129
27	oar3_OAR⟩	3267700	0.115064
27	oar3_OAR⟩	3268936	0.100375
27	oar3_OAR⟩	3275400	0.005379
27	oar3_OAR⟩	3278596	0.061207
27	oar3_OAR⟩	3281046	0.088043
27	oar3_OAR⟩	3283002	0.019058
27	oar3_OAR⟩	3286008	-0.02853
27	oar3_OAR⟩	3288423	0.057757
27	oar3_OAR⟩	3293539	0.086354
27	oar3_OAR⟩	3296822	0.011291
27	oar3_OAR⟩	3306254	0.035322
27	oar3_OAR⟩	3308158	0.011698
27	oar3_OAR⟩	3310736	0.083298
27	oar3_OAR⟩	3318115	0.111576
27	oar3_OAR⟩	3321038	-0.01453
27	oar3_OAR⟩	3323781	0.035613
27	oar3_OAR⟩	3330411	0.012044
27	oar3_OAR⟩	3334267	-0.01915
27	oar3_OAR⟩	3334655	-0.03509
27	oar3_OAR⟩	3335947	0.034261
27	oar3_OAR⟩	3340971	0.009006
27	oar3_OAR⟩	3347545	0.091713
27	oar3_OAR⟩	3348588	0.038594
27	oar3_OAR⟩	3350575	0.024422
27	oar3_OAR⟩	3359220	0.070783
27	oar3_OAR⟩	3360682	-0.01935
27	oar3_OAR⟩	3360997	-0.02597
27	oar3_OAR⟩	3364320	-0.00901
27	oar3_OAR⟩	3371455	0.094362

27	oar3_OAR〉	3373112	0.099484
27	oar3_OAR〉	3378429	-0.02348
27	oar3_OAR〉	3384300	-0.02337
27	oar3_OAR〉	3387212	0.043851
27	oar3_OAR〉	3387911	-0.00102
27	oar3_OAR〉	3395883	0.027118
27	oar3_OAR〉	3401362	0.060764
27	oar3_OAR〉	3404127	-0.02872
27	oar3_OAR〉	3407651	0.020257
27	oar3_OAR〉	3414727	-0.01132
27	oar3_OAR〉	3414800	0.018645
27	oar3_OAR〉	3416712	0.165195
27	oar3_OAR〉	3418161	0.011257
27	oar3_OAR〉	3423964	0.015055
27	oar3_OAR〉	3427821	-0.02374
27	oar3_OAR〉	3429305	0.026346
27	oar3_OAR〉	3431339	-0.00776
27	oar3_OAR〉	3432619	0.028775
27	oar3_OAR〉	3437329	0.032665
27	oar3_OAR〉	3442418	NA
27	oar3_OAR〉	3443567	NA
27	oar3_OAR〉	3443618	0.037873
27	oar3_OAR〉	3443736	0.02459
27	oar3_OAR〉	3443834	0.025367
27	oar3_OAR〉	3447542	-0.00152
27	oar3_OAR〉	3452963	-0.02153
27	oar3_OAR〉	3453949	0.050179
27	oar3_OAR〉	3458406	0.067504
27	oar3_OAR〉	3459891	0.018564
27	oar3_OAR〉	3463792	0.189014
27	oar3_OAR〉	3466707	0.058332
27	oar3_OAR〉	3469191	0.062026
27	oar3_OAR〉	3473201	0.027255
27	oar3_OAR〉	3477507	-0.01546
27	oar3_OAR〉	3482749	0.005635
27	oar3_OAR〉	3482863	0.008176
27	oar3_OAR〉	3485586	-0.01049
27	oar3_OAR〉	3487989	0.02379
27	oar3_OAR〉	3493110	0.063543
27	oar3_OAR〉	3495232	0.001402
27	oar3_OAR〉	3496214	-0.00677
27	oar3_OAR〉	3500471	-0.03126
27	oar3_OAR〉	3503839	-0.03126
27	oar3_OAR〉	3508562	0.006014
27	oar3_OAR〉	3510903	0.011067
27	oar3_OAR〉	3511563	0.035121
27	oar3_OAR〉	3512820	0.041221
27	oar3_OAR〉	3516090	0.047343
27	oar3_OAR〉	3521488	0.022722
27	oar3_OAR〉	3523191	0.127203
27	oar3_OAR〉	3523562	0.03733
27	oar3_OAR〉	3529025	0.01879
27	oar3_OAR〉	3532107	0.01879
27	oar3_OAR〉	3534193	-0.02374

27	oar3_OAR〉	3540106	0.000889
27	oar3_OAR〉	3540605	0.024061
27	oar3_OAR〉	3546410	0.017718
27	oar3_OAR〉	3546745	0.028704
27	oar3_OAR〉	3550025	-0.02879
27	oar3_OAR〉	3551839	0.000363
27	oar3_OAR〉	3552606	0.031225
27	oar3_OAR〉	3560354	-0.00606
27	oar3_OAR〉	3562848	0.025803
27	oar3_OAR〉	3564488	-0.00996
27	oar3_OAR〉	3574345	-0.00928
27	oar3_OAR〉	3575013	0.020358
27	oar3_OAR〉	3576782	0.018388
27	oar3_OAR〉	3577095	0.025714
27	oar3_OAR〉	3586948	-0.00284
27	oar3_OAR〉	3587312	0.015836
27	oar3_OAR〉	3587318	0.057005
27	oar3_OAR〉	3590278	0.003164
27	oar3_OAR〉	3592403	-0.01956
27	oar3_OAR〉	3597136	-0.00349
27	oar3_OAR〉	3597402	0.055303
27	oar3_OAR〉	3602869	0.038838
27	oar3_OAR〉	3602948	-0.03688
27	oar3_OAR〉	3609037	0.016028
27	oar3_OAR〉	3609587	0.011365
27	oar3_OAR〉	3613657	0.009215
27	oar3_OAR〉	3615140	0.017279
27	oar3_OAR〉	3618040	-0.0078
27	oar3_OAR〉	3620311	-0.00395
27	oar3_OAR〉	3626267	0.004517
27	oar3_OAR〉	3629159	0.022385
27	oar3_OAR〉	3634065	0.073
27	oar3_OAR〉	3634127	0.074294
27	oar3_OAR〉	3634498	-0.00401
27	oar3_OAR〉	3636500	-0.0123
27	oar3_OAR〉	3642694	-0.02144
27	oar3_OAR〉	3649767	-0.015
27	oar3_OAR〉	3652240	-0.00939
27	oar3_OAR〉	3652563	0.021755
27	oar3_OAR〉	3654746	0.003446
27	oar3_OAR〉	3654842	-0.01828
27	oar3_OAR〉	3660463	0.07868
27	oar3_OAR〉	3667314	0.066489
27	oar3_OAR〉	3667797	-0.02721
27	oar3_OAR〉	3672772	-0.00463
27	oar3_OAR〉	3679506	-0.02582
27	oar3_OAR〉	3684970	0.027174
27	oar3_OAR〉	3688905	0.002013
27	oar3_OAR〉	3692219	-0.01423
27	oar3_OAR〉	3695194	-0.01593
27	oar3_OAR〉	3695349	-0.02973
27	oar3_OAR〉	3698505	-0.00836
27	oar3_OAR〉	3705270	-0.01702
27	oar3_OAR〉	3705482	-0.01863

27	oar3_OAR〉	3710641	0.097359
27	oar3_OAR〉	3711194	0.007039
27	oar3_OAR〉	3718797	-0.00421
27	oar3_OAR〉	3719495	0.100646
27	oar3_OAR〉	3722012	0.022809
27	oar3_OAR〉	3724823	-0.01527
27	oar3_OAR〉	3725771	-0.00731
27	oar3_OAR〉	3727793	-0.00731
27	oar3_OAR〉	3730844	0.023922
27	oar3_OAR〉	3732036	0.0597
27	oar3_OAR〉	3736442	-0.00097
27	oar3_OAR〉	3736516	0.009572
27	oar3_OAR〉	3740623	-0.01003
27	oar3_OAR〉	3743314	0.011653
27	oar3_OAR〉	3743934	0.028665
27	oar3_OAR〉	3747483	0.093535
27	oar3_OAR〉	3750631	0.006898
27	oar3_OAR〉	3756309	0.028738
27	oar3_OAR〉	3762654	0.040509
27	oar3_OAR〉	3764113	-0.01131
27	oar3_OAR〉	3765140	-0.02184
27	oar3_OAR〉	3777163	0.133317
27	oar3_OAR〉	3777222	0.148871
27	oar3_OAR〉	3780404	0.054341
27	oar3_OAR〉	3780431	0.046534
27	oar3_OAR〉	3783922	0.071155
27	oar3_OAR〉	3783987	0.078139
27	oar3_OAR〉	3785131	0.03833
27	oar3_OAR〉	3785368	0.071155
27	oar3_OAR〉	3792624	0.020822
27	oar3_OAR〉	3793875	0.08968
27	oar3_OAR〉	3793963	0.056205
27	oar3_OAR〉	3798926	0.005553
27	oar3_OAR〉	3806780	0.039538
27	oar3_OAR〉	3809418	0.067989
27	oar3_OAR〉	3813600	0.114012
27	oar3_OAR〉	3817924	0.105526
27	oar3_OAR〉	3824840	0.140003
27	oar3_OAR〉	3831645	-0.01829
27	oar3_OAR〉	3831764	0.10112
27	oar3_OAR〉	3833640	0.001959
27	oar3_OAR〉	3834091	-0.00389
27	oar3_OAR〉	3844982	-0.01275
27	oar3_OAR〉	3845042	-0.01628
27	oar3_OAR〉	3850641	-0.01854
27	oar3_OAR〉	3853102	-0.02512
27	oar3_OAR〉	3856401	-0.03059
27	oar3_OAR〉	3858295	-0.03059
27	oar3_OAR〉	3863493	-0.0014
27	oar3_OAR〉	3869897	0.030402
27	oar3_OAR〉	3870672	-0.02286
27	oar3_OAR〉	3876163	0.03967
27	oar3_OAR〉	3880409	0.001047
27	oar3_OAR〉	3884856	-0.02766

27	oar3_OAR〉	3891334	0.002228
27	oar3_OAR〉	3891384	0.002228
27	oar3_OAR〉	3896785	0.028133
27	oar3_OAR〉	3897337	-0.02603
27	oar3_OAR〉	3901901	-0.02603
27	oar3_OAR〉	3902061	-0.01212
27	oar3_OAR〉	3902248	-0.0003
27	oar3_OAR〉	3907831	-0.0003
27	oar3_OAR〉	3913208	-0.00511
27	oar3_OAR〉	3914462	-0.01856
27	oar3_OAR〉	3915403	0.009127
27	oar3_OAR〉	3915613	0.009127
27	oar3_OAR〉	3924082	-0.01745
27	oar3_OAR〉	3933286	0.004461
27	oar3_OAR〉	3933442	-0.02545
27	oar3_OAR〉	3934705	-0.01251
27	oar3_OAR〉	3936191	0.058806
27	oar3_OAR〉	3943268	-0.02397
27	oar3_OAR〉	3945421	-0.02633
27	oar3_OAR〉	3952480	-0.0006
27	oar3_OAR〉	3956544	-0.02144
27	oar3_OAR〉	3957965	-0.00457
27	oar3_OAR〉	3964241	-0.03648
27	oar3_OAR〉	3966655	-0.02575
27	oar3_OAR〉	3970712	-0.00194
27	oar3_OAR〉	3977374	0.169057
27	oar3_OAR〉	3980747	-0.02702
27	oar3_OAR〉	3981159	0.161533
27	oar3_OAR〉	3986078	0.127762
27	oar3_OAR〉	3987337	-0.01777
27	oar3_OAR〉	3988858	0.176285
27	oar3_OAR〉	3992741	0.007237
27	oar3_OAR〉	3993808	0.051444
27	oar3_OAR〉	3996390	0.044673
27	oar3_OAR〉	4001249	0.096515
27	oar3_OAR〉	4006714	-0.0304
27	oar3_OAR〉	4006983	-0.00589
27	oar3_OAR〉	4009853	0.008772
27	oar3_OAR〉	4016958	-0.02404
27	oar3_OAR〉	4018095	0.023405
27	oar3_OAR〉	4018341	0.023405
27	oar3_OAR〉	4025571	-0.01255
27	oar3_OAR〉	4030644	0.107092
27	oar3_OAR〉	4030704	0.161297
27	oar3_OAR〉	4031695	0.064919
27	oar3_OAR〉	4036637	0.200159
27	oar3_OAR〉	4040324	-0.0071
27	oar3_OAR〉	4040448	-0.00448
27	oar3_OAR〉	4045505	-0.02017
27	oar3_OAR〉	4045556	-0.01587
27	oar3_OAR〉	4048552	-0.02759
27	oar3_OAR〉	4053047	0.016032
27	oar3_OAR〉	4054153	0.027777
27	oar3_OAR〉	4058295	0.066905

27	oar3_OAR〉	4060900	0.079655
27	oar3_OAR〉	4067113	0.0526
27	oar3_OAR〉	4068540	0.011365
27	oar3_OAR〉	4071436	0.035405
27	oar3_OAR〉	4075251	-0.01822
27	oar3_OAR〉	4077762	-0.00307
27	oar3_OAR〉	4079570	0.006583
27	oar3_OAR〉	4080441	-0.01684
27	oar3_OAR〉	4086168	0.144002
27	oar3_OAR〉	4088329	-0.01967
27	oar3_OAR〉	4089412	-0.01183
27	oar3_OAR〉	4092182	-0.01098
27	oar3_OAR〉	4093550	0.003348
27	oar3_OAR〉	4099376	0.056068
27	oar3_OAR〉	4099458	-0.02028
27	oar3_OAR〉	4100026	-0.01556
27	oar3_OAR〉	4104738	-0.01846
27	oar3_OAR〉	4104807	-0.01234
27	oar3_OAR〉	4110403	0.03311
27	oar3_OAR〉	4110472	-0.01916
27	oar3_OAR〉	4112518	-0.00433
27	oar3_OAR〉	4116201	0.00834
27	oar3_OAR〉	4123256	-0.02228
27	oar3_OAR〉	4125253	0.026304
27	oar3_OAR〉	4125747	0.081175
27	oar3_OAR〉	4130792	-0.00049
27	oar3_OAR〉	4133034	-0.01871
27	oar3_OAR〉	4133325	-0.02838
27	oar3_OAR〉	4137437	0.004693
27	oar3_OAR〉	4140417	0.001957
27	oar3_OAR〉	4141223	0.040514
27	oar3_OAR〉	4149800	-0.01294
27	oar3_OAR〉	4151022	-0.0236
27	oar3_OAR〉	4153016	-0.00866
27	oar3_OAR〉	4153449	0.015429
27	oar3_OAR〉	4156056	0.059429
27	oar3_OAR〉	4156134	0.036752
27	oar3_OAR〉	4166944	-0.02351
27	oar3_OAR〉	4168308	-0.00606
27	oar3_OAR〉	4171031	-0.00199
27	oar3_OAR〉	4176846	0.008522
27	oar3_OAR〉	4180794	0.01839
27	oar3_OAR〉	4182055	-0.00335
27	oar3_OAR〉	4190663	-0.01567
27	oar3_OAR〉	4192143	-0.01783
27	oar3_OAR〉	4192588	0.041333
27	oar3_OAR〉	4199400	0.034634
27	oar3_OAR〉	4205816	0.021407
27	oar3_OAR〉	4207524	0.050888
27	oar3_OAR〉	4212466	0.066982
27	oar3_OAR〉	4215682	0.08642
27	oar3_OAR〉	4217264	0.025571
27	oar3_OAR〉	4225531	-0.01264
27	oar3_OAR〉	4225972	-0.01264

27	oar3_OAR〉	4226910	0.021818
27	oar3_OAR〉	4232167	-0.02414
27	oar3_OAR〉	4232685	-0.02709
27	oar3_OAR〉	4234389	-0.03096
27	oar3_OAR〉	4240115	0.002651
27	oar3_OAR〉	4244605	0.030243
27	oar3_OAR〉	4245233	0.030243
27	oar3_OAR〉	4248030	-0.00575
27	oar3_OAR〉	4252469	0.092417
27	oar3_OAR〉	4253380	-0.00575
27	oar3_OAR〉	4256780	0.010341
27	oar3_OAR〉	4258745	0.049721
27	oar3_OAR〉	4262460	-0.02492
27	oar3_OAR〉	4264453	-0.01717
27	oar3_OAR〉	4267800	-0.01866
27	oar3_OAR〉	4271064	0.017792
27	oar3_OAR〉	4271481	-0.02169
27	oar3_OAR〉	4276606	-0.02184
27	oar3_OAR〉	4278332	-0.00352
27	oar3_OAR〉	4280393	0.01898
27	oar3_OAR〉	4282213	-0.01472
27	oar3_OAR〉	4289971	0.031408
27	oar3_OAR〉	4290599	0.049289
27	oar3_OAR〉	4290798	0.067101
27	oar3_OAR〉	4299116	0.100474
27	oar3_OAR〉	4300720	0.004311
27	oar3_OAR〉	4301130	0.015806
27	oar3_OAR〉	4310390	0.079352
27	oar3_OAR〉	4312742	0.011887
27	oar3_OAR〉	4315694	0.002868
27	oar3_OAR〉	4320982	0.014747
27	oar3_OAR〉	4322915	0.02759
27	oar3_OAR〉	4323162	0.022271
27	oar3_OAR〉	4326870	0.044724
27	oar3_OAR〉	4329000	0.086028
27	oar3_OAR〉	4332675	-0.00161
27	oar3_OAR〉	4334973	-0.00825
27	oar3_OAR〉	4338572	-0.01617
27	oar3_OAR〉	4342707	0.036792
27	oar3_OAR〉	4344770	0.02374
27	oar3_OAR〉	4345565	0.008723
27	oar3_OAR〉	4346302	0.063224
27	oar3_OAR〉	4352484	-0.01617
27	oar3_OAR〉	4357874	0.037535
27	oar3_OAR〉	4362238	0.078313
27	oar3_OAR〉	4362301	0.097043
27	oar3_OAR〉	4364940	0.007901
27	oar3_OAR〉	4373995	0.036193
27	oar3_OAR〉	4375885	0.064573
27	oar3_OAR〉	4386070	-0.03022
27	oar3_OAR〉	4386126	0.016709
27	oar3_OAR〉	4386233	0.221421
27	oar3_OAR〉	4399805	0.026685
27	oar3_OAR〉	4400015	-0.01581

27	oar3_OAR〉	4400445	-0.0054
27	oar3_OAR〉	4405787	-0.02169
27	oar3_OAR〉	4408287	0.054445
27	oar3_OAR〉	4408410	0.054445
27	oar3_OAR〉	4411059	0.033905
27	oar3_OAR〉	4413610	0.043533
27	oar3_OAR〉	4417647	0.042883
27	oar3_OAR〉	4426765	0.024891
27	oar3_OAR〉	4427652	-0.02054
27	oar3_OAR〉	4427920	-0.00039
27	oar3_OAR〉	4436226	-0.00026
27	oar3_OAR〉	4436286	0.126968
27	oar3_OAR〉	4441952	0.007181
27	oar3_OAR〉	4445164	0.027176
27	oar3_OAR〉	4458626	-0.01872
27	oar3_OAR〉	4461796	0.08524
27	oar3_OAR〉	4464139	0.002325
27	oar3_OAR〉	4486782	-0.00039
27	oar3_OAR〉	4490377	NA
27	oar3_OAR〉	4491600	-0.02133
27	oar3_OAR〉	4496938	0.012715
27	oar3_OAR〉	4500980	0.015298
27	oar3_OAR〉	4501245	-0.0196
27	oar3_OAR〉	4508829	0.015383
27	oar3_OAR〉	4512464	-0.02036
27	oar3_OAR〉	4520549	0.049642
27	oar3_OAR〉	4524138	-0.00623
27	oar3_OAR〉	4525407	-0.00067
27	oar3_OAR〉	4525677	0.053574
27	oar3_OAR〉	4532179	0.074647
27	oar3_OAR〉	4536018	0.020432
27	oar3_OAR〉	4536065	0.040904
27	oar3_OAR〉	4536187	0.040904
27	oar3_OAR〉	4540732	-0.00309
27	oar3_OAR〉	4549764	0.013267
27	oar3_OAR〉	4552528	0.21064
27	oar3_OAR〉	4553751	0.314109
27	oar3_OAR〉	4558018	0.019974
27	oar3_OAR〉	4561527	0.05869
27	oar3_OAR〉	4562833	0.05869
27	oar3_OAR〉	4572315	-0.03479
27	oar3_OAR〉	4574470	-0.01994
27	oar3_OAR〉	4574631	0.092417
27	oar3_OAR〉	4588139	0.090668
27	oar3_OAR〉	4590060	0.098049
27	oar3_OAR〉	4593564	0.136531
27	oar3_OAR〉	4593577	0.048862
27	oar3_OAR〉	4598900	0.081798
27	oar3_OAR〉	4600741	0.031482
27	oar3_OAR〉	4603640	-0.00271
27	oar3_OAR〉	4609633	0.006791
27	oar3_OAR〉	4613731	0.037131
27	oar3_OAR〉	4614795	0.073957
27	oar3_OAR〉	4619793	0.059771

27	oar3_OAR〉	4620633	-0.02258
27	oar3_OAR〉	4621801	0.02149
27	oar3_OAR〉	4626689	-0.02112
27	oar3_OAR〉	4630478	0.029397
27	oar3_OAR〉	4632250	0.015085
27	oar3_OAR〉	4637818	0.08407
27	oar3_OAR〉	4637890	0.131104
27	oar3_OAR〉	4640618	0.007178
27	oar3_OAR〉	4640935	-0.02049
27	oar3_OAR〉	4647771	-0.01185
27	oar3_OAR〉	4652161	-0.02504
27	oar3_OAR〉	4652252	-0.02504
27	oar3_OAR〉	4655366	0.013417
27	oar3_OAR〉	4662878	-0.00057
27	oar3_OAR〉	4666768	-0.03298
27	oar3_OAR〉	4666978	0.041346
27	oar3_OAR〉	4669098	-0.01679
27	oar3_OAR〉	4670899	0.060324
27	oar3_OAR〉	4675272	-0.0164
27	oar3_OAR〉	4675843	0.038703
27	oar3_OAR〉	4679140	0.058806
27	oar3_OAR〉	4679206	-0.00175
27	oar3_OAR〉	4683665	-0.02756
27	oar3_OAR〉	4684788	-0.00996
27	oar3_OAR〉	4690117	-0.00996
27	oar3_OAR〉	4694278	-0.02063
27	oar3_OAR〉	4698323	-0.04136
27	oar3_OAR〉	4699534	-0.01847
27	oar3_OAR〉	4701517	-0.00707
27	oar3_OAR〉	4705914	-0.02737
27	oar3_OAR〉	4707439	-0.01585
27	oar3_OAR〉	4711744	-0.03193
27	oar3_OAR〉	4717508	-0.02471
27	oar3_OAR〉	4717572	-0.02378
27	oar3_OAR〉	4717930	0.043755
27	oar3_OAR〉	4723652	-0.0065
27	oar3_OAR〉	4728659	-0.02358
27	oar3_OAR〉	4729361	-0.02777
27	oar3_OAR〉	4730129	-0.01621
27	oar3_OAR〉	4735781	-0.00462
27	oar3_OAR〉	4742079	0.040066
27	oar3_OAR〉	4743541	0.013417
27	oar3_OAR〉	4747273	-0.01592
27	oar3_OAR〉	4751822	-0.00464
27	oar3_OAR〉	4755474	-0.01126
27	oar3_OAR〉	4759945	0.117144
27	oar3_OAR〉	4765578	-0.0193
27	oar3_OAR〉	4765752	0.010197
27	oar3_OAR〉	4767866	0.020466
27	oar3_OAR〉	4770726	-0.00673
27	oar3_OAR〉	4776179	0.007181
27	oar3_OAR〉	4776306	0.131493
27	oar3_OAR〉	4779940	0.06167
27	oar3_OAR〉	4782412	-0.011

27	oar3_OAR〉	4783083	0.114486
27	oar3_OAR〉	4790792	0.064598
27	oar3_OAR〉	4794234	0.103967
27	oar3_OAR〉	4794345	0.055773
27	oar3_OAR〉	4797443	0.03863
27	oar3_OAR〉	4804506	0.018033
27	oar3_OAR〉	4805039	-0.00932
27	oar3_OAR〉	4811386	0.014516
27	oar3_OAR〉	4812386	0.014516
27	oar3_OAR〉	4813507	0.090709
27	oar3_OAR〉	4816284	0.055567
27	oar3_OAR〉	4820155	-0.02242
27	oar3_OAR〉	4826260	-0.01193
27	oar3_OAR〉	4831396	0.017279
27	oar3_OAR〉	4831451	0.011638
27	oar3_OAR〉	4832404	0.011638
27	oar3_OAR〉	4836614	-0.01229
27	oar3_OAR〉	4842115	-0.00731
27	oar3_OAR〉	4847257	-0.02303
27	oar3_OAR〉	4848024	0.028803
27	oar3_OAR〉	4851580	-0.02039
27	oar3_OAR〉	4855111	0.023167
27	oar3_OAR〉	4857944	-0.02393
27	oar3_OAR〉	4869168	-0.0132
27	oar3_OAR〉	4872143	0.06063
27	oar3_OAR〉	4876041	0.021496
27	oar3_OAR〉	4883662	-0.00334
27	oar3_OAR〉	4886048	-0.00961
27	oar3_OAR〉	4886232	0.046852
27	oar3_OAR〉	4886357	0.032691
27	oar3_OAR〉	4894826	0.01982
27	oar3_OAR〉	4894840	-0.02295
27	oar3_OAR〉	4895268	-0.01038
27	oar3_OAR〉	4896608	-0.00825
27	oar3_OAR〉	4904929	0.011019
27	oar3_OAR〉	4907648	-0.02591
27	oar3_OAR〉	4912894	-0.02292
27	oar3_OAR〉	4913761	0.010307
27	oar3_OAR〉	4920665	-0.01628
27	oar3_OAR〉	4922813	-0.02893
27	oar3_OAR〉	4925931	-0.00837
27	oar3_OAR〉	4926731	-0.02621
27	oar3_OAR〉	4931212	0.025781
27	oar3_OAR〉	4931217	0.144951
27	oar3_OAR〉	4931662	-0.0285
27	oar3_OAR〉	4937187	-0.02593
27	oar3_OAR〉	4937371	-0.02965
27	oar3_OAR〉	4941423	-0.01255
27	oar3_OAR〉	4942522	0.080539
27	oar3_OAR〉	4942577	-0.02206
27	oar3_OAR〉	4947728	-0.02159
27	oar3_OAR〉	4951365	0.046877
27	oar3_OAR〉	4956520	-0.0275
27	oar3_OAR〉	4961920	-0.00879

27	oar3_OAR〉	4970925	0.162028
27	oar3_OAR〉	4971018	0.023861
27	oar3_OAR〉	4973143	0.072742
27	oar3_OAR〉	4976926	0.031803
27	oar3_OAR〉	4981436	0.04275
27	oar3_OAR〉	4983040	-0.00836
27	oar3_OAR〉	4985145	0.19027
27	oar3_OAR〉	4989423	0.101469
27	oar3_OAR〉	4991065	0.006284
27	oar3_OAR〉	4992903	0.027864
27	oar3_OAR〉	4998893	-0.02342
27	oar3_OAR〉	5003720	0.016328
27	oar3_OAR〉	5004064	0.136425
27	oar3_OAR〉	5007230	0.162871
27	oar3_OAR〉	5009018	-0.02158
27	oar3_OAR〉	5013474	0.0103
27	oar3_OAR〉	5016451	-0.02049
27	oar3_OAR〉	5017409	-0.01879
27	oar3_OAR〉	5018127	0.021773
27	oar3_OAR〉	5018259	0.000968
27	oar3_OAR〉	5022216	-0.01737
27	oar3_OAR〉	5025939	0.004777
27	oar3_OAR〉	5027610	0.096193
27	oar3_OAR〉	5029095	0.043637
27	oar3_OAR〉	5030656	0.031474
27	oar3_OAR〉	5039296	0.044157
27	oar3_OAR〉	5039354	-0.03218
27	oar3_OAR〉	5041552	-0.00623
27	oar3_OAR〉	5049068	-0.03293
27	oar3_OAR〉	5049391	0.010585
27	oar3_OAR〉	5052829	0.008892
27	oar3_OAR〉	5054971	0.009985
27	oar3_OAR〉	5055565	0.190874
27	oar3_OAR〉	5061108	-0.00901
27	oar3_OAR〉	5064562	0.021788
27	oar3_OAR〉	5066879	-0.00344
27	oar3_OAR〉	5067017	-0.01518
27	oar3_OAR〉	5076367	-0.02622
27	oar3_OAR〉	5078228	0.003447
27	oar3_OAR〉	5078503	0.033535
27	oar3_OAR〉	5080606	0.001262
27	oar3_OAR〉	5082269	0.001262
27	oar3_OAR〉	5082326	0.049128
27	oar3_OAR〉	5086919	-0.00042
27	oar3_OAR〉	5090788	-0.0256
27	oar3_OAR〉	5092725	-0.02229
27	oar3_OAR〉	5093457	0.011164
27	oar3_OAR〉	5098453	-0.02531
27	oar3_OAR〉	5106780	-0.0188
27	oar3_OAR〉	5108786	0.104241
27	oar3_OAR〉	5113577	0.010026
27	oar3_OAR〉	5113911	-0.00774
27	oar3_OAR〉	5117755	0.003874
27	oar3_OAR〉	5120139	-0.03248

27	oar3_OAR〉	5123920	-0.00759
27	oar3_OAR〉	5124147	0.011785
27	oar3_OAR〉	5127570	0.056969
27	oar3_OAR〉	5131439	0.05706
27	oar3_OAR〉	5135238	0.017154
27	oar3_OAR〉	5138218	0.008723
27	oar3_OAR〉	5146694	-0.03075
27	oar3_OAR〉	5147047	-0.00993
27	oar3_OAR〉	5152405	0.013599
27	oar3_OAR〉	5152608	0.013599
27	oar3_OAR〉	5158624	-0.02054
27	oar3_OAR〉	5160891	-0.02012
27	oar3_OAR〉	5164110	-0.02534
27	oar3_OAR〉	5164311	-0.01636
27	oar3_OAR〉	5165082	-0.02419
27	oar3_OAR〉	5175162	-0.01455
27	oar3_OAR〉	5183077	-0.02152
27	oar3_OAR〉	5183170	-0.02152
27	oar3_OAR〉	5200878	0.047726
27	oar3_OAR〉	5203244	-0.00465
27	oar3_OAR〉	5203414	-0.02642
27	oar3_OAR〉	5206808	-0.00034
27	oar3_OAR〉	5208054	0.00834
27	oar3_OAR〉	5213007	-0.00273
27	oar3_OAR〉	5218455	0.009322
27	oar3_OAR〉	5218675	0.055815
27	oar3_OAR〉	5219577	0.066206
27	oar3_OAR〉	5220821	0.008296
27	oar3_OAR〉	5226098	-0.00864
27	oar3_OAR〉	5230088	0.02717
27	oar3_OAR〉	5233598	0.11776
27	oar3_OAR〉	5236381	0.062319
27	oar3_OAR〉	5239012	-0.00271
27	oar3_OAR〉	5239550	0.012941
27	oar3_OAR〉	5244825	0.052782
27	oar3_OAR〉	5246419	-0.02086
27	oar3_OAR〉	5252598	0.000605
27	oar3_OAR〉	5252730	0.027354
27	oar3_OAR〉	5256386	0.059109
27	oar3_OAR〉	5263645	0.064748
27	oar3_OAR〉	5264427	-0.03258
27	oar3_OAR〉	5265526	0.08836
27	oar3_OAR〉	5269716	-0.03114
27	oar3_OAR〉	5271468	0.005844
27	oar3_OAR〉	5274465	0.007986
27	oar3_OAR〉	5277519	0.054946
27	oar3_OAR〉	5283572	-0.01789
27	oar3_OAR〉	5289069	-0.0177
27	oar3_OAR〉	5289310	0.069376
27	oar3_OAR〉	5295840	-0.02252
27	oar3_OAR〉	5300622	0.046171
27	oar3_OAR〉	5301180	0.011653
27	oar3_OAR〉	5303032	-0.0026
27	oar3_OAR〉	5308376	-0.01737

27	oar3_OAR〉	5309329	-0.01453
27	oar3_OAR〉	5312100	-0.02446
27	oar3_OAR〉	5314395	0.034228
27	oar3_OAR〉	5314494	0.034228
27	oar3_OAR〉	5317851	0.020666
27	oar3_OAR〉	5319650	0.001875
27	oar3_OAR〉	5325013	0.026654
27	oar3_OAR〉	5325622	-0.00248
27	oar3_OAR〉	5330816	0.04082
27	oar3_OAR〉	5330915	0.01024
27	oar3_OAR〉	5331797	0.0257
27	oar3_OAR〉	5339183	0.040901
27	oar3_OAR〉	5347462	0.031135
27	oar3_OAR〉	5348340	0.008296
27	oar3_OAR〉	5354538	-0.03041
27	oar3_OAR〉	5356271	0.034314
27	oar3_OAR〉	5358433	-0.01098
27	oar3_OAR〉	5360470	0.017678
27	oar3_OAR〉	5361146	-0.02593
27	oar3_OAR〉	5365519	0.069127
27	oar3_OAR〉	5369546	-0.02926
27	oar3_OAR〉	5369889	0.005349
27	oar3_OAR〉	5375590	-0.00364
27	oar3_OAR〉	5375676	-0.01525
27	oar3_OAR〉	5376878	0.003537
27	oar3_OAR〉	5385571	-0.01081
27	oar3_OAR〉	5387373	-0.00456
27	oar3_OAR〉	5390797	-0.01331
27	oar3_OAR〉	5393865	-0.02053
27	oar3_OAR〉	5396739	-0.01801
27	oar3_OAR〉	5400133	0.008739
27	oar3_OAR〉	5401765	0.006014
27	oar3_OAR〉	5402273	0.032056
27	oar3_OAR〉	5408006	0.032056
27	oar3_OAR〉	5410203	-0.00408
27	oar3_OAR〉	5421125	-0.00508
27	oar3_OAR〉	5424623	-0.02744
27	oar3_OAR〉	5429705	0.005553
27	oar3_OAR〉	5429921	0.044948
27	oar3_OAR〉	5432289	-0.01311
27	oar3_OAR〉	5433834	0.012749
27	oar3_OAR〉	5437568	0.005553
27	oar3_OAR〉	5437730	0.005553
27	oar3_OAR〉	5439848	-0.00922
27	oar3_OAR〉	5444820	-0.01489
27	oar3_OAR〉	5445734	0.027463
27	oar3_OAR〉	5445798	-0.01489
27	oar3_OAR〉	5450316	0.003763
27	oar3_OAR〉	5453685	-0.00456
27	oar3_OAR〉	5456524	-0.00208
27	oar3_OAR〉	5460829	0.002221
27	oar3_OAR〉	5462232	0.053231
27	oar3_OAR〉	5467106	-0.01926
27	oar3_OAR〉	5467434	-0.03193

27	oar3_OAR〉	5469650	-0.01902
27	oar3_OAR〉	5474424	-0.00691
27	oar3_OAR〉	5476643	-0.01993
27	oar3_OAR〉	5479367	-0.02152
27	oar3_OAR〉	5480994	0.028621
27	oar3_OAR〉	5483236	-0.02392
27	oar3_OAR〉	5487962	-0.00928
27	oar3_OAR〉	5490751	-0.01902
27	oar3_OAR〉	5491999	0.045957
27	oar3_OAR〉	5498994	-0.03011
27	oar3_OAR〉	5504200	0.00704
27	oar3_OAR〉	5506919	0.057413
27	oar3_OAR〉	5508371	-0.02324
27	oar3_OAR〉	5512130	0.010985
27	oar3_OAR〉	5513134	0.053115
27	oar3_OAR〉	5516328	-0.03441
27	oar3_OAR〉	5518490	0.102069
27	oar3_OAR〉	5525564	-0.02531
27	oar3_OAR〉	5526458	0.018399
27	oar3_OAR〉	5529980	0.086732
27	oar3_OAR〉	5536484	0.10324
27	oar3_OAR〉	5537932	0.038056
27	oar3_OAR〉	5538156	0.050547
27	oar3_OAR〉	5543056	0.093025
27	oar3_OAR〉	5545203	-0.01536
27	oar3_OAR〉	5548173	-0.00751
27	oar3_OAR〉	5548945	0.065991
27	oar3_OAR〉	5550549	-0.0125
27	oar3_OAR〉	5554480	-0.01662
27	oar3_OAR〉	5555479	-0.01368
27	oar3_OAR〉	5567989	0.061223
27	oar3_OAR〉	5568499	0.089927
27	oar3_OAR〉	5569076	0.037425
27	oar3_OAR〉	5574269	0.065556
27	oar3_OAR〉	5584880	-0.01932
27	oar3_OAR〉	5590364	-0.02051
27	oar3_OAR〉	5593179	-0.01716
27	oar3_OAR〉	5593290	-0.0236
27	oar3_OAR〉	5599841	0.005824
27	oar3_OAR〉	5600733	-0.02345
27	oar3_OAR〉	5601319	-0.01955
27	oar3_OAR〉	5606868	-0.02607
27	oar3_OAR〉	5610415	-0.02083
27	oar3_OAR〉	5610614	0.032454
27	oar3_OAR〉	5611970	0.032454
27	oar3_OAR〉	5616330	0.113127
27	oar3_OAR〉	5621871	0.019729
27	oar3_OAR〉	5621959	0.192147
27	oar3_OAR〉	5622055	0.15549
27	oar3_OAR〉	5627584	-0.02884
27	oar3_OAR〉	5629958	0.007986
27	oar3_OAR〉	5633830	0.032787
27	oar3_OAR〉	5633983	0.015319
27	oar3_OAR〉	5637944	-0.00462

27	oar3_OAR〉	5646383	0.020407
27	oar3_OAR〉	5648445	0.111416
27	oar3_OAR〉	5656430	0.107332
27	oar3_OAR〉	5658289	0.02583
27	oar3_OAR〉	5659639	-0.0038
27	oar3_OAR〉	5662514	0.024274
27	oar3_OAR〉	5663650	-0.01615
27	oar3_OAR〉	5668671	-0.00026
27	oar3_OAR〉	5680122	0.056786
27	oar3_OAR〉	5681042	0.019023
27	oar3_OAR〉	5686028	0.008005
27	oar3_OAR〉	5690003	0.032691
27	oar3_OAR〉	5691064	0.02432
27	oar3_OAR〉	5691366	0.039383
27	oar3_OAR〉	5696633	0.008759
27	oar3_OAR〉	5698238	-0.00433
27	oar3_OAR〉	5705939	0.033773
27	oar3_OAR〉	5706092	-0.00149
27	oar3_OAR〉	5706653	-0.00149
27	oar3_OAR〉	5711857	-0.00396
27	oar3_OAR〉	5719318	-0.00654
27	oar3_OAR〉	5722891	0.055258
27	oar3_OAR〉	5726269	-0.01948
27	oar3_OAR〉	5731717	-0.01392
27	oar3_OAR〉	5735425	-0.01392
27	oar3_OAR〉	5735921	0.001167
27	oar3_OAR〉	5741963	0.016301
27	oar3_OAR〉	5744607	0.008594
27	oar3_OAR〉	5745406	0.050205
27	oar3_OAR〉	5754335	0.053964
27	oar3_OAR〉	5754737	-0.02013
27	oar3_OAR〉	5758561	-0.01629
27	oar3_OAR〉	5760297	0.004526
27	oar3_OAR〉	5762231	0.038091
27	oar3_OAR〉	5763820	0.012392
27	oar3_OAR〉	5769051	0.051595
27	oar3_OAR〉	5772232	-0.01529
27	oar3_OAR〉	5773559	0.032932
27	oar3_OAR〉	5779546	0.080958
27	oar3_OAR〉	5783154	0.06415
27	oar3_OAR〉	5785770	-0.02572
27	oar3_OAR〉	5794796	-0.01156
27	oar3_OAR〉	5796562	-0.02638
27	oar3_OAR〉	5796708	-0.02351
27	oar3_OAR〉	5800419	-0.0139
27	oar3_OAR〉	5807202	0.011365
27	oar3_OAR〉	5807706	-0.01967
27	oar3_OAR〉	5807822	-0.01733
27	oar3_OAR〉	5816319	-0.0194
27	oar3_OAR〉	5816991	-0.00875
27	oar3_OAR〉	5820288	-0.01356
27	oar3_OAR〉	5825155	-0.0106
27	oar3_OAR〉	5825259	0.069491
27	oar3_OAR〉	5825627	0.098658

27	oar3_OAR〉	5830294	0.062129
27	oar3_OAR〉	5835881	0.008496
27	oar3_OAR〉	5836804	-0.00961
27	oar3_OAR〉	5837066	0.013241
27	oar3_OAR〉	5843179	0.074956
27	oar3_OAR〉	5845104	0.123969
27	oar3_OAR〉	5853480	0.01552
27	oar3_OAR〉	5854448	0.039569
27	oar3_OAR〉	5855852	0.033905
27	oar3_OAR〉	5855920	-0.02835
27	oar3_OAR〉	5874033	0.009704
27	oar3_OAR〉	5875518	0.07333
27	oar3_OAR〉	5876011	0.08539
27	oar3_OAR〉	5878382	-0.01445
27	oar3_OAR〉	5884147	0.002439
27	oar3_OAR〉	5888283	-0.01904
27	oar3_OAR〉	5889177	-0.01674
27	oar3_OAR〉	5890974	-0.0287
27	oar3_OAR〉	5891148	-0.02816
27	oar3_OAR〉	5895446	-0.03351
27	oar3_OAR〉	5895765	0.007039
27	oar3_OAR〉	5900391	-0.01611
27	oar3_OAR〉	5900736	0.00579
27	oar3_OAR〉	5901303	-0.00556
27	oar3_OAR〉	5901700	-0.01591
27	oar3_OAR〉	5906670	-0.00777
27	oar3_OAR〉	5908597	0.02172
27	oar3_OAR〉	5911801	-0.0276
27	oar3_OAR〉	5927887	0.012393
27	oar3_OAR〉	5927970	-0.01113
27	oar3_OAR〉	5931284	-0.02893
27	oar3_OAR〉	5938597	0.018537
27	oar3_OAR〉	5941282	0.02829
27	oar3_OAR〉	5944276	-0.03508
27	oar3_OAR〉	5946173	0.064547
27	oar3_OAR〉	5946373	0.000342
27	oar3_OAR〉	5951561	0.010227
27	oar3_OAR〉	5952959	0.01464
27	oar3_OAR〉	5954637	-0.03218
27	oar3_OAR〉	5962594	0.091681
27	oar3_OAR〉	5965565	0.029056
27	oar3_OAR〉	5965682	0.02829
27	oar3_OAR〉	5971352	-0.02336
27	oar3_OAR〉	5972894	-0.03262
27	oar3_OAR〉	5983511	0.071029
27	oar3_OAR〉	5984284	0.071029
27	oar3_OAR〉	5984352	0.012052
27	oar3_OAR〉	5991440	0.066871
27	oar3_OAR〉	5992350	########
27	oar3_OAR〉	5998565	-0.00911
27	oar3_OAR〉	6001451	0.012241
27	oar3_OAR〉	6001813	0.072149
27	oar3_OAR〉	6001914	0.072149
27	oar3_OAR〉	6003644	0.060748

27	oar3_OAR〉	6009338	0.042211
27	oar3_OAR〉	6010424	-0.01202
27	oar3_OAR〉	6012287	0.039233
27	oar3_OAR〉	6012401	0.043361
27	oar3_OAR〉	6017196	-0.01224
27	oar3_OAR〉	6017435	-0.01483
27	oar3_OAR〉	6023061	0.007771
27	oar3_OAR〉	6023658	-0.00094
27	oar3_OAR〉	6025897	-0.01079
27	oar3_OAR〉	6028689	0.052216
27	oar3_OAR〉	6035558	0.014009
27	oar3_OAR〉	6036444	-0.02262
27	oar3_OAR〉	6037503	0.032366
27	oar3_OAR〉	6039317	-0.0333
27	oar3_OAR〉	6040736	0.000893
27	oar3_OAR〉	6047295	0.002775
27	oar3_OAR〉	6049309	0.010207
27	oar3_OAR〉	6052003	-0.01565
27	oar3_OAR〉	6055291	0.06354
27	oar3_OAR〉	6055394	-0.03075
27	oar3_OAR〉	6058165	-0.03024
27	oar3_OAR〉	6061429	-0.02063
27	oar3_OAR〉	6066980	0.0824
27	oar3_OAR〉	6067813	0.0824
27	oar3_OAR〉	6069595	0.092614
27	oar3_OAR〉	6078068	-0.00363
27	oar3_OAR〉	6082606	0.037317
27	oar3_OAR〉	6083949	-0.01738
27	oar3_OAR〉	6084948	0.003806
27	oar3_OAR〉	6092531	0.004928
27	oar3_OAR〉	6092605	-0.01538
27	oar3_OAR〉	6096317	0.006976
27	oar3_OAR〉	6097899	0.056295
27	oar3_OAR〉	6101808	-0.00363
27	oar3_OAR〉	6103410	0.070327
27	oar3_OAR〉	6104153	0.018564
27	oar3_OAR〉	6107077	-0.01053
27	oar3_OAR〉	6114794	-0.02477
27	oar3_OAR〉	6115665	-0.02049
27	oar3_OAR〉	6121366	-0.02766
27	oar3_OAR〉	6122777	-0.02049
27	oar3_OAR〉	6125625	-0.0168
27	oar3_OAR〉	6127130	-0.01609
27	oar3_OAR〉	6131406	0.008461
27	oar3_OAR〉	6135306	-0.03332
27	oar3_OAR〉	6136300	-0.00022
27	oar3_OAR〉	6140096	-0.00479
27	oar3_OAR〉	6140244	-0.01735
27	oar3_OAR〉	6140434	-0.00918
27	oar3_OAR〉	6146730	-0.02744
27	oar3_OAR〉	6149146	0.009639
27	oar3_OAR〉	6149326	0.013285
27	oar3_OAR〉	6153010	-0.01543
27	oar3_OAR〉	6158462	0.0073

27	oar3_OAR〉	6160305	-0.01953
27	oar3_OAR〉	6163842	-0.01171
27	oar3_OAR〉	6164214	0.063627
27	oar3_OAR〉	6167426	0.1012
27	oar3_OAR〉	6169268	-0.01757
27	oar3_OAR〉	6171108	-0.0151
27	oar3_OAR〉	6174312	-0.0245
27	oar3_OAR〉	6175678	0.054693
27	oar3_OAR〉	6180111	0.070714
27	oar3_OAR〉	6186039	-0.01508
27	oar3_OAR〉	6187753	-0.00298
27	oar3_OAR〉	6190174	0.022343
27	oar3_OAR〉	6192519	-0.01874
27	oar3_OAR〉	6200368	0.045331
27	oar3_OAR〉	6200603	-0.00354
27	oar3_OAR〉	6209195	-0.02555
27	oar3_OAR〉	6212110	-0.01003
27	oar3_OAR〉	6212993	-0.00134
27	oar3_OAR〉	6217529	-0.00928
27	oar3_OAR〉	6222671	-0.03296
27	oar3_OAR〉	6223785	-0.034
27	oar3_OAR〉	6224864	-0.03117
27	oar3_OAR〉	6225118	-0.02103
27	oar3_OAR〉	6236678	-0.00395
27	oar3_OAR〉	6241531	-0.00933
27	oar3_OAR〉	6242251	-0.0245
27	oar3_OAR〉	6242318	0.069072
27	oar3_OAR〉	6249404	0.018641
27	oar3_OAR〉	6253604	0.096154
27	oar3_OAR〉	6259155	0.025488
27	oar3_OAR〉	6261828	0.050992
27	oar3_OAR〉	6261920	0.072533
27	oar3_OAR〉	6268160	0.102702
27	oar3_OAR〉	6269124	-0.03036
27	oar3_OAR〉	6272519	0.102702
27	oar3_OAR〉	6274079	0.081269
27	oar3_OAR〉	6280294	-0.02197
27	oar3_OAR〉	6283426	-0.02567
27	oar3_OAR〉	6283513	-0.01745
27	oar3_OAR〉	6284914	-0.02523
27	oar3_OAR〉	6290600	0.074824
27	oar3_OAR〉	6301544	-0.00348
27	oar3_OAR〉	6303586	0.012148
27	oar3_OAR〉	6306918	-0.01846
27	oar3_OAR〉	6310293	-0.01737
27	oar3_OAR〉	6310405	0.061695
27	oar3_OAR〉	6316489	-0.02926
27	oar3_OAR〉	6320049	-0.01726
27	oar3_OAR〉	6322997	0.055718
27	oar3_OAR〉	6323076	0.055291
27	oar3_OAR〉	6324645	0.086539
27	oar3_OAR〉	6333520	-0.00148
27	oar3_OAR〉	6336746	0.037402
27	oar3_OAR〉	6339343	-0.01856

27	oar3_OAR›	6344755	0.103909
27	oar3_OAR›	6344824	-0.03414
27	oar3_OAR›	6348556	0.232277
27	oar3_OAR›	6357819	0.017143
27	oar3_OAR›	6357833	0.028993
27	oar3_OAR›	6361607	0.232277
27	oar3_OAR›	6368453	-0.0267
27	oar3_OAR›	6369130	0.001228
27	oar3_OAR›	6369287	0.015728
27	oar3_OAR›	6374170	3.84E-05
27	oar3_OAR›	6375169	-0.01731
27	oar3_OAR›	6381187	0.035041
27	oar3_OAR›	6386041	0.099525
27	oar3_OAR›	6386809	0.022647
27	oar3_OAR›	6391029	0.021188
27	oar3_OAR›	6393565	0.027281
27	oar3_OAR›	6398319	0.003446
27	oar3_OAR›	6401120	-0.02576
27	oar3_OAR›	6401186	-0.02576
27	oar3_OAR›	6405242	-0.01356
27	oar3_OAR›	6410486	0.017727
27	oar3_OAR›	6410598	0.017727
27	oar3_OAR›	6411510	0.037474
27	oar3_OAR›	6412051	-0.01358
27	oar3_OAR›	6415454	-0.00871
27	oar3_OAR›	6425636	-0.0176
27	oar3_OAR›	6425661	-0.00258
27	oar3_OAR›	6428506	-0.00775
27	oar3_OAR›	6428699	-0.00775
27	oar3_OAR›	6431411	-0.02922
27	oar3_OAR›	6435950	0.002019
27	oar3_OAR›	6438848	-0.02671
27	oar3_OAR›	6438945	-0.01567
27	oar3_OAR›	6441541	-0.0334
27	oar3_OAR›	6447027	-0.01731
27	oar3_OAR›	6448653	-0.02753
27	oar3_OAR›	6457439	0.008296
27	oar3_OAR›	6464222	0.088877
27	oar3_OAR›	6465734	0.00613
27	oar3_OAR›	6472892	0.158649
27	oar3_OAR›	6473174	0.140008
27	oar3_OAR›	6475782	0.031361
27	oar3_OAR›	6481664	0.008296
27	oar3_OAR›	6482039	0.11557
27	oar3_OAR›	6482433	0.073003
27	oar3_OAR›	6488075	-0.00363
27	oar3_OAR›	6493552	-0.01574
27	oar3_OAR›	6493901	-0.01574
27	oar3_OAR›	6501383	0.028918
27	oar3_OAR›	6503635	0.059842
27	oar3_OAR›	6505425	0.081932
27	oar3_OAR›	6507177	0.001957
27	oar3_OAR›	6512613	0.043997
27	oar3_OAR›	6514801	0.077448

27	oar3_OAR〉	6520134	0.008635
27	oar3_OAR〉	6525125	0.057272
27	oar3_OAR〉	6528096	0.005261
27	oar3_OAR〉	6530195	0.038001
27	oar3_OAR〉	6531037	-0.02449
27	oar3_OAR〉	6535679	0.00834
27	oar3_OAR〉	6536300	0.057905
27	oar3_OAR〉	6539781	0.012634
27	oar3_OAR〉	6540899	0.075889
27	oar3_OAR〉	6545120	-0.00354
27	oar3_OAR〉	6545901	0.022083
27	oar3_OAR〉	6545992	0.022083
27	oar3_OAR〉	6556502	0.011278
27	oar3_OAR〉	6564532	0.06703
27	oar3_OAR〉	6566338	0.014222
27	oar3_OAR〉	6566536	-0.03273
27	oar3_OAR〉	6569512	-0.02488
27	oar3_OAR〉	6577124	-0.00592
27	oar3_OAR〉	6577189	-0.00592
27	oar3_OAR〉	6585520	0.056068
27	oar3_OAR〉	6586018	-0.01737
27	oar3_OAR〉	6594207	-0.00344
27	oar3_OAR〉	6596837	-0.01936
27	oar3_OAR〉	6600650	-0.02078
27	oar3_OAR〉	6602878	0.010792
27	oar3_OAR〉	6603883	0.025629
27	oar3_OAR〉	6611181	0.065694
27	oar3_OAR〉	6611716	-0.01512
27	oar3_OAR〉	6616199	0.039326
27	oar3_OAR〉	6616309	0.052945
27	oar3_OAR〉	6623825	-0.01184
27	oar3_OAR〉	6625066	0.037404
27	oar3_OAR〉	6627610	-0.02844
27	oar3_OAR〉	6628925	-0.02588
27	oar3_OAR〉	6632742	0.18733
27	oar3_OAR〉	6633203	0.18733
27	oar3_OAR〉	6634853	0.003806
27	oar3_OAR〉	6641303	0.044136
27	oar3_OAR〉	6644740	0.05029
27	oar3_OAR〉	6646765	-0.00187
27	oar3_OAR〉	6646925	0.01293
27	oar3_OAR〉	6649104	0.004368
27	oar3_OAR〉	6652715	0.044752
27	oar3_OAR〉	6654147	-0.02982
27	oar3_OAR〉	6655589	-0.03204
27	oar3_OAR〉	6659443	0.073272
27	oar3_OAR〉	6661425	-0.02492
27	oar3_OAR〉	6662913	0.033869
27	oar3_OAR〉	6668045	0.029926
27	oar3_OAR〉	6669171	0.08039
27	oar3_OAR〉	6677003	-0.01131
27	oar3_OAR〉	6683354	0.038352
27	oar3_OAR〉	6683646	-0.02087
27	oar3_OAR〉	6685085	-0.02306

27	oar3_OAR〉	6688178	-0.02004
27	oar3_OAR〉	6690385	-0.0181
27	oar3_OAR〉	6692034	-0.01065
27	oar3_OAR〉	6695451	0.066872
27	oar3_OAR〉	6700459	0.073272
27	oar3_OAR〉	6700511	0.106066
27	oar3_OAR〉	6702408	0.025289
27	oar3_OAR〉	6703372	0.043925
27	oar3_OAR〉	6709152	-0.02593
27	oar3_OAR〉	6711194	-0.00645
27	oar3_OAR〉	6719254	0.009704
27	oar3_OAR〉	6719438	0.129102
27	oar3_OAR〉	6719456	0.013013
27	oar3_OAR〉	6723443	-0.01212
27	oar3_OAR〉	6725163	0.043086
27	oar3_OAR〉	6727313	0.128977
27	oar3_OAR〉	6731352	-0.01026
27	oar3_OAR〉	6731412	0.026126
27	oar3_OAR〉	6733720	-0.01692
27	oar3_OAR〉	6736220	-0.02847
27	oar3_OAR〉	6736801	-0.02184
27	oar3_OAR〉	6746071	-0.0298
27	oar3_OAR〉	6747051	0.000889
27	oar3_OAR〉	6747309	-0.01731
27	oar3_OAR〉	6749366	-0.01692
27	oar3_OAR〉	6751937	0.011067
27	oar3_OAR〉	6755585	0.026045
27	oar3_OAR〉	6758135	-0.01614
27	oar3_OAR〉	6758817	-0.00435
27	oar3_OAR〉	6763285	-0.02958
27	oar3_OAR〉	6763765	-0.02316
27	oar3_OAR〉	6764119	-0.01705
27	oar3_OAR〉	6773614	-0.02187
27	oar3_OAR〉	6775267	-0.01872
27	oar3_OAR〉	6775874	-0.01872
27	oar3_OAR〉	6781460	-0.007
27	oar3_OAR〉	6788811	-0.02447
27	oar3_OAR〉	6789013	-0.02481
27	oar3_OAR〉	6793103	0.005692
27	oar3_OAR〉	6798113	-0.0262
27	oar3_OAR〉	6805872	-0.01546
27	oar3_OAR〉	6806972	0.046469
27	oar3_OAR〉	6808021	0.034939
27	oar3_OAR〉	6817338	-0.01193
27	oar3_OAR〉	6817801	0.011893
27	oar3_OAR〉	6819908	0.01975
27	oar3_OAR〉	6820029	0.015732
27	oar3_OAR〉	6830219	0.002672
27	oar3_OAR〉	6834575	-0.02805
27	oar3_OAR〉	6837101	-0.02577
27	oar3_OAR〉	6840694	0.002764
27	oar3_OAR〉	6841124	-0.02592
27	oar3_OAR〉	6842692	0.002764
27	oar3_OAR〉	6847151	-0.01097

27	oar3_OAR〉	6848987	-0.00174
27	oar3_OAR〉	6849060	-0.03192
27	oar3_OAR〉	6853200	0.00834
27	oar3_OAR〉	6854473	0.01024
27	oar3_OAR〉	6860215	-0.01931
27	oar3_OAR〉	6860305	0.015773
27	oar3_OAR〉	6863429	-0.02242
27	oar3_OAR〉	6865698	0.022899
27	oar3_OAR〉	6874873	0.004461
27	oar3_OAR〉	6882167	0.004461
27	oar3_OAR〉	6899054	0.004461
27	oar3_OAR〉	6899714	0.021527
27	oar3_OAR〉	6904763	0.004461
27	oar3_OAR〉	6910599	-0.00317
27	oar3_OAR〉	6911775	0.0033
27	oar3_OAR〉	6912253	-0.00575
27	oar3_OAR〉	6917371	0.004461
27	oar3_OAR〉	6921709	0.004461
27	oar3_OAR〉	6922365	0.004461
27	oar3_OAR〉	6927706	0.004461
27	oar3_OAR〉	6930789	0.002267
27	oar3_OAR〉	6933374	0.004461
27	oar3_OAR〉	6934420	0.004461
27	oar3_OAR〉	6937444	0.003998
27	oar3_OAR〉	6943490	0.004461
27	oar3_OAR〉	6947007	0.056578
27	oar3_OAR〉	6951605	0.004461
27	oar3_OAR〉	6957211	0.028387
27	oar3_OAR〉	6957505	0.004461
27	oar3_OAR〉	6962954	0.004461
27	oar3_OAR〉	6965563	0.004461
27	oar3_OAR〉	6968859	0.004461
27	oar3_OAR〉	6974699	-0.0206
27	oar3_OAR〉	6985105	0.004461
27	oar3_OAR〉	6985400	0.044747
27	oar3_OAR〉	6990118	0.004461
27	oar3_OAR〉	6990793	0.050801
27	oar3_OAR〉	6991417	0.004461
27	oar3_OAR〉	6996733	-0.00918
27	oar3_OAR〉	6997590	-0.01731
27	oar3_OAR〉	7004045	0.005166
27	oar3_OAR〉	7005579	0.073039
27	oar3_OAR〉	7015381	-0.00918
27	oar3_OAR〉	7022495	0.004461
27	oar3_OAR〉	7024987	0.004461
27	oar3_OAR〉	7027948	0.004461
27	oar3_OAR〉	7033316	0.011566
27	oar3_OAR〉	7034318	0.004461
27	oar3_OAR〉	7040232	-0.02674
27	oar3_OAR〉	7045743	-0.00935
27	oar3_OAR〉	7055540	-0.00575
27	oar3_OAR〉	7065447	-0.02674
27	oar3_OAR〉	7092963	0.010327
27	oar3_OAR〉	7093048	0.073704

27	oar3_OAR〉	7093156	-0.03101
27	oar3_OAR〉	7098572	0.073704
27	oar3_OAR〉	7104522	-0.0253
27	oar3_OAR〉	7105436	-0.03167
27	oar3_OAR〉	7108693	-0.03167
27	oar3_OAR〉	7110030	0.022876
27	oar3_OAR〉	7113496	0.022876
27	oar3_OAR〉	7119986	-0.01426
27	oar3_OAR〉	7120756	0.103899
27	oar3_OAR〉	7124355	-0.00882
27	oar3_OAR〉	7128167	0.007039
27	oar3_OAR〉	7131095	-0.00882
27	oar3_OAR〉	7133434	-0.00838
27	oar3_OAR〉	7134428	-0.03554
27	oar3_OAR〉	7140595	-0.01157
27	oar3_OAR〉	7142134	-0.01157
27	oar3_OAR〉	7145161	0.067968
27	oar3_OAR〉	7146284	0.03569
27	oar3_OAR〉	7152613	0.007039
27	oar3_OAR〉	7153449	0.085695
27	oar3_OAR〉	7156657	-0.01836
27	oar3_OAR〉	7163516	-0.01367
27	oar3_OAR〉	7166165	0.00549
27	oar3_OAR〉	7172560	-0.00269
27	oar3_OAR〉	7177043	-0.02481
27	oar3_OAR〉	7179729	-0.02481
27	oar3_OAR〉	7182471	-0.03221
27	oar3_OAR〉	7189726	0.007039
27	oar3_OAR〉	7197865	0.007039
27	oar3_OAR〉	7199653	-0.03099
27	oar3_OAR〉	7207354	-0.00688
27	oar3_OAR〉	7212534	0.048281
27	oar3_OAR〉	7213011	0.017374
27	oar3_OAR〉	7220009	0.012941
27	oar3_OAR〉	7229122	0.016807
27	oar3_OAR〉	7233625	-0.00617
27	oar3_OAR〉	7239707	0.016807
27	oar3_OAR〉	7246972	0.016807
27	oar3_OAR〉	7250971	0.048281
27	oar3_OAR〉	7261688	0.007039
27	oar3_OAR〉	7266714	0.013023
27	oar3_OAR〉	7269775	0.071029
27	oar3_OAR〉	7273361	0.108463
27	oar3_OAR〉	7273852	0.025488
27	oar3_OAR〉	7282105	0.104845
27	oar3_OAR〉	7282720	0.104845
27	oar3_OAR〉	7284710	0.104845
27	oar3_OAR〉	7292605	0.104845
27	oar3_OAR〉	7294189	0.013023
27	oar3_OAR〉	7294652	0.107989
27	oar3_OAR〉	7299089	0.052381
27	oar3_OAR〉	7308402	0.04803
27	oar3_OAR〉	7318605	0.14726
27	oar3_OAR〉	7330025	0.07625

27	oar3_OAR〉	7338156	0.079942
27	oar3_OAR〉	7339676	-0.00935
27	oar3_OAR〉	7340076	0.133416
27	oar3_OAR〉	7355451	-0.00011
27	oar3_OAR〉	7400218	-0.01851
27	oar3_OAR〉	7410375	-0.01851
27	oar3_OAR〉	7415503	0.01388
27	oar3_OAR〉	7428282	-0.01851
27	oar3_OAR〉	7432814	0.01388
27	oar3_OAR〉	7439133	0.01388
27	oar3_OAR〉	7439745	-0.00735
27	oar3_OAR〉	7441151	-0.01851
27	oar3_OAR〉	7443470	-0.01851
27	oar3_OAR〉	7444638	0.01388
27	oar3_OAR〉	7448322	0.03967
27	oar3_OAR〉	7452146	0.01388
27	oar3_OAR〉	7453449	-0.01851
27	oar3_OAR〉	7472426	0.01388
27	oar3_OAR〉	7496692	-0.01418
27	oar3_OAR〉	7550105	0.04675
27	oar3_OAR〉	7550199	-0.02327
27	oar3_OAR〉	7569127	-0.00935
27	oar3_OAR〉	7569873	0.049642
27	oar3_OAR〉	7585592	-0.02674
27	oar3_OAR〉	7585950	-0.00935
27	oar3_OAR〉	7603570	0.049642
27	oar3_OAR〉	7606501	0.049642
27	oar3_OAR〉	7614599	0.049642
27	oar3_OAR〉	7616620	-0.00935
27	oar3_OAR〉	7621243	-0.02674
27	oar3_OAR〉	7651998	-0.02327
27	oar3_OAR〉	7663252	0.049642
27	oar3_OAR〉	7664405	-0.00935
27	oar3_OAR〉	7743566	0.007039
27	oar3_OAR〉	7749957	0.007039
27	oar3_OAR〉	7755105	0.044724
27	oar3_OAR〉	7755863	0.022876
27	oar3_OAR〉	7776502	0.022876
27	oar3_OAR〉	7780872	0.044724
27	oar3_OAR〉	7785988	-0.0253
27	oar3_OAR〉	7791130	0.007039
27	oar3_OAR〉	7791335	0.022876
27	oar3_OAR〉	7792867	-0.03145
27	oar3_OAR〉	7803883	0.007039
27	oar3_OAR〉	7812218	-0.0014
27	oar3_OAR〉	7817011	-0.03531
27	oar3_OAR〉	7819129	0.044724
27	oar3_OAR〉	7825798	-0.00918
27	oar3_OAR〉	7827851	0.043437
27	oar3_OAR〉	7838087	0.007931
27	oar3_OAR〉	7838823	-0.02562
27	oar3_OAR〉	7840204	0.005909
27	oar3_OAR〉	7852606	0.007039
27	oar3_OAR〉	7853787	0.02101

27	oar3_OAR>	7855129	0.069547
27	oar3_OAR>	7860231	-0.01192
27	oar3_OAR>	7860393	0.007039
27	oar3_OAR>	7867138	0.007039
27	oar3_OAR>	7867802	0.007039
27	oar3_OAR>	7868137	0.007039
27	oar3_OAR>	7873576	0.007039
27	oar3_OAR>	7878296	0.007039
27	oar3_OAR>	7880122	0.018013
27	oar3_OAR>	7882087	0.021865
27	oar3_OAR>	7888446	0.044724
27	oar3_OAR>	7897209	0.021865
27	oar3_OAR>	7897341	-0.00626
27	oar3_OAR>	7899468	0.021865
27	oar3_OAR>	7903520	0.103251
27	oar3_OAR>	7905719	-0.00723
27	oar3_OAR>	7912880	0.0275
27	oar3_OAR>	7914957	-0.02597
27	oar3_OAR>	7916244	-0.02402
27	oar3_OAR>	7929814	0.007587
27	oar3_OAR>	7933851	0.007587
27	oar3_OAR>	7934363	0.044919
27	oar3_OAR>	7937027	-0.0148
27	oar3_OAR>	7942744	0.007587
27	oar3_OAR>	7943958	0.007587
27	oar3_OAR>	7944506	0.007587
27	oar3_OAR>	7949938	-0.01473
27	oar3_OAR>	7951444	-0.01473
27	oar3_OAR>	7954964	0.011116
27	oar3_OAR>	7955606	0.031119
27	oar3_OAR>	7959954	-0.02607
27	oar3_OAR>	7961714	0.052914
27	oar3_OAR>	7968805	-0.01859
27	oar3_OAR>	7970584	0.014394
27	oar3_OAR>	7972348	0.077226
27	oar3_OAR>	7978669	0.077226
27	oar3_OAR>	7984209	-0.03296
27	oar3_OAR>	7984828	-0.03296
27	oar3_OAR>	7997412	0.053348
27	oar3_OAR>	8001833	0.053348
27	oar3_OAR>	8010499	0.025215
27	oar3_OAR>	8010501	0.041488
27	oar3_OAR>	8019345	-0.0313
27	oar3_OAR>	8020023	0.009694
27	oar3_OAR>	8024886	0.007039
27	oar3_OAR>	8026683	-0.00574
27	oar3_OAR>	8028983	0.007039
27	oar3_OAR>	8030690	0.025215
27	oar3_OAR>	8034098	-0.03081
27	oar3_OAR>	8039296	0.045085
27	oar3_OAR>	8040774	0.015509
27	oar3_OAR>	8046282	-0.00574
27	oar3_OAR>	8049154	0.00661
27	oar3_OAR>	8049556	0.021709

27	oar3_OAR〉	8053448	0.00661
27	oar3_OAR〉	8064138	-0.00178
27	oar3_OAR〉	8066810	0.00661
27	oar3_OAR〉	8069880	0.010874
27	oar3_OAR〉	8078760	0.087043
27	oar3_OAR〉	8081967	0.016214
27	oar3_OAR〉	8082748	0.122295
27	oar3_OAR〉	8087330	0.07333
27	oar3_OAR〉	8088959	0.048284
27	oar3_OAR〉	8097458	0.012749
27	oar3_OAR〉	8099864	-0.01212
27	oar3_OAR〉	8099972	0.027738
27	oar3_OAR〉	8107933	-0.00874
27	oar3_OAR〉	8110443	-0.00874
27	oar3_OAR〉	8119057	-0.02778
27	oar3_OAR〉	8124693	0.025656
27	oar3_OAR〉	8124942	-0.01205
27	oar3_OAR〉	8131627	0.015023
27	oar3_OAR〉	8137720	0.007039
27	oar3_OAR〉	8145854	0.015023
27	oar3_OAR〉	8146861	-0.01998
27	oar3_OAR〉	8150180	0.004601
27	oar3_OAR〉	8157435	-0.01363
27	oar3_OAR〉	8166426	0.065685
27	oar3_OAR〉	8172288	0.154215
27	oar3_OAR〉	8175631	0.03988
27	oar3_OAR〉	8181938	0.002439
27	oar3_OAR〉	8182039	-0.02402
27	oar3_OAR〉	8186064	0.020467
27	oar3_OAR〉	8190255	-0.03171
27	oar3_OAR〉	8192367	0.020467
27	oar3_OAR〉	8195600	-0.02223
27	oar3_OAR〉	8202912	0.007497
27	oar3_OAR〉	8209453	0.055993
27	oar3_OAR〉	8209666	-0.01484
27	oar3_OAR〉	8215145	-0.01766
27	oar3_OAR〉	8220046	0.028951
27	oar3_OAR〉	8229348	-0.02974
27	oar3_OAR〉	8234140	-0.01731
27	oar3_OAR〉	8234291	-0.00674
27	oar3_OAR〉	8234954	0.078236
27	oar3_OAR〉	8241586	0.05901
27	oar3_OAR〉	8246999	-0.01308
27	oar3_OAR〉	8255863	-0.03091
27	oar3_OAR〉	8256022	-0.02957
27	oar3_OAR〉	8262336	-0.00228
27	oar3_OAR〉	8265147	-0.00228
27	oar3_OAR〉	8273618	0.011884
27	oar3_OAR〉	8300199	0.02374
27	oar3_OAR〉	8305900	0.038636
27	oar3_OAR〉	8314585	-0.0143
27	oar3_OAR〉	8315389	-0.02082
27	oar3_OAR〉	8315465	0.10849
27	oar3_OAR〉	8327912	0.08464

27	oar3_OAR〉	8333273	0.10849
27	oar3_OAR〉	8334356	0.014978
27	oar3_OAR〉	8339282	0.061399
27	oar3_OAR〉	8345943	0.075335
27	oar3_OAR〉	8346608	-0.01787
27	oar3_OAR〉	8347517	0.075335
27	oar3_OAR〉	8349684	0.099038
27	oar3_OAR〉	8352634	-0.0278
27	oar3_OAR〉	8357793	0.071826
27	oar3_OAR〉	8358010	0.007973
27	oar3_OAR〉	8370218	0.072793
27	oar3_OAR〉	8371219	0.002868
27	oar3_OAR〉	8371712	0.005595
27	oar3_OAR〉	8377053	0.086518
27	oar3_OAR〉	8378483	0.137068
27	oar3_OAR〉	8383963	0.033052
27	oar3_OAR〉	8387345	0.021977
27	oar3_OAR〉	8388267	0.021977
27	oar3_OAR〉	8396368	0.00822
27	oar3_OAR〉	8411549	-0.01222
27	oar3_OAR〉	8413123	0.006902
27	oar3_OAR〉	8419981	-0.02168
27	oar3_OAR〉	8421563	-0.00395
27	oar3_OAR〉	8426073	-0.01674
27	oar3_OAR〉	8436500	-0.00837
27	oar3_OAR〉	8437961	0.028803
27	oar3_OAR〉	8438029	-0.01439
27	oar3_OAR〉	8448852	-0.01547
27	oar3_OAR〉	8451607	-0.01126
27	oar3_OAR〉	8454118	0.029875
27	oar3_OAR〉	8460936	0.040551
27	oar3_OAR〉	8463576	-0.02922
27	oar3_OAR〉	8463659	0.029875
27	oar3_OAR〉	8468569	0.040551
27	oar3_OAR〉	8471452	-0.00971
27	oar3_OAR〉	8480263	0.002754
27	oar3_OAR〉	8484119	-0.0149
27	oar3_OAR〉	8485971	0.003301
27	oar3_OAR〉	8487606	0.034228
27	oar3_OAR〉	8500374	0.063308
27	oar3_OAR〉	8503127	-0.02341
27	oar3_OAR〉	8510877	-0.00716
27	oar3_OAR〉	8511511	0.00766
27	oar3_OAR〉	8512533	0.00766
27	oar3_OAR〉	8514072	-0.02444
27	oar3_OAR〉	8522856	0.038645
27	oar3_OAR〉	8523111	0.037044
27	oar3_OAR〉	8524486	0.003215
27	oar3_OAR〉	8537580	-0.00511
27	oar3_OAR〉	8538081	0.045143
27	oar3_OAR〉	8542251	0.00686
27	oar3_OAR〉	8564842	0.00686
27	oar3_OAR〉	8565637	0.019023
27	oar3_OAR〉	8575621	0.0188

27	oar3_OAR〉	8575862	0.034468
27	oar3_OAR〉	8579929	0.077748
27	oar3_OAR〉	8580570	0.077748
27	oar3_OAR〉	8583961	0.036702
27	oar3_OAR〉	8589210	-0.0014
27	oar3_OAR〉	8596942	-0.02217
27	oar3_OAR〉	8597048	0.054567
27	oar3_OAR〉	8598864	0.07148
27	oar3_OAR〉	8609391	0.050381
27	oar3_OAR〉	8609625	0.054567
27	oar3_OAR〉	8620009	0.053273
27	oar3_OAR〉	8620110	-0.0291
27	oar3_OAR〉	8622001	0.024876
27	oar3_OAR〉	8629242	0.004761
27	oar3_OAR〉	8629775	0.141716
27	oar3_OAR〉	8630750	0.060591
27	oar3_OAR〉	8633216	0.060591
27	oar3_OAR〉	8637346	0.046787
27	oar3_OAR〉	8642909	-0.0112
27	oar3_OAR〉	8647926	-0.017
27	oar3_OAR〉	8647980	-0.017
27	oar3_OAR〉	8652338	0.002944
27	oar3_OAR〉	8659253	-0.00521
27	oar3_OAR〉	8662291	-0.00606
27	oar3_OAR〉	8663377	0.012045
27	oar3_OAR〉	8665216	-0.03429
27	oar3_OAR〉	8665903	0.007039
27	oar3_OAR〉	8668212	0.066395
27	oar3_OAR〉	8670283	-0.00606
27	oar3_OAR〉	8675703	0.031919
27	oar3_OAR〉	8680556	-0.01014
27	oar3_OAR〉	8680957	0.088417
27	oar3_OAR〉	8684953	0.056295
27	oar3_OAR〉	8693871	-0.02813
27	oar3_OAR〉	8695313	0.006757
27	oar3_OAR〉	8695438	0.006757
27	oar3_OAR〉	8699131	-0.01327
27	oar3_OAR〉	8699467	0.110003
27	oar3_OAR〉	8705997	0.180831
27	oar3_OAR〉	8707171	-0.00294
27	oar3_OAR〉	8712465	-0.02977
27	oar3_OAR〉	8713806	-0.00294
27	oar3_OAR〉	8714573	0.056295
27	oar3_OAR〉	8719391	0.070321
27	oar3_OAR〉	8722693	0.022147
27	oar3_OAR〉	8724735	0.134079
27	oar3_OAR〉	8727836	0.1593
27	oar3_OAR〉	8734578	-0.03553
27	oar3_OAR〉	8735108	-0.01423
27	oar3_OAR〉	8741222	-0.02449
27	oar3_OAR〉	8741391	0.069801
27	oar3_OAR〉	8746351	-0.01801
27	oar3_OAR〉	8746394	0.009452
27	oar3_OAR〉	8751484	0.069801

27	oar3_OAR〉	8757440	-0.00776
27	oar3_OAR〉	8759265	-0.00776
27	oar3_OAR〉	8760014	-0.01423
27	oar3_OAR〉	8769578	-0.00297
27	oar3_OAR〉	8776615	0.022096
27	oar3_OAR〉	8779093	-0.03175
27	oar3_OAR〉	8790415	-0.02778
27	oar3_OAR〉	8806956	0.075962
27	oar3_OAR〉	8809444	0.06659
27	oar3_OAR〉	8810643	0.005967
27	oar3_OAR〉	8812588	-0.01014
27	oar3_OAR〉	8814852	0.082562
27	oar3_OAR〉	8820357	0.018466
27	oar3_OAR〉	8821689	-0.02916
27	oar3_OAR〉	8826248	0.024935
27	oar3_OAR〉	8828971	0.018466
27	oar3_OAR〉	8834190	0.041221
27	oar3_OAR〉	8835082	0.000954
27	oar3_OAR〉	8835989	0.000954
27	oar3_OAR〉	8837297	-0.027
27	oar3_OAR〉	8839735	0.041221
27	oar3_OAR〉	8846667	0.061554
27	oar3_OAR〉	8847502	0.01544
27	oar3_OAR〉	8851428	0.029401
27	oar3_OAR〉	8859758	0.049642
27	oar3_OAR〉	8862066	0.017279
27	oar3_OAR〉	8864407	0.084783
27	oar3_OAR〉	8868071	0.042137
27	oar3_OAR〉	8874049	0.049642
27	oar3_OAR〉	8877117	0.049642
27	oar3_OAR〉	8879493	0.049642
27	oar3_OAR〉	8886397	0.097836
27	oar3_OAR〉	8887919	0.008723
27	oar3_OAR〉	8888339	-0.0168
27	oar3_OAR〉	8892380	NA
27	oar3_OAR〉	8894093	NA
27	oar3_OAR〉	8896947	NA
27	oar3_OAR〉	8899982	0.026651
27	oar3_OAR〉	8900539	0.007973
27	oar3_OAR〉	8906780	0.060748
27	oar3_OAR〉	8907533	0.060748
27	oar3_OAR〉	8909879	-0.01461
27	oar3_OAR〉	8912502	0.051419
27	oar3_OAR〉	8912568	-0.03676
27	oar3_OAR〉	8915405	0.060748
27	oar3_OAR〉	8919939	-0.02176
27	oar3_OAR〉	8924297	0.044003
27	oar3_OAR〉	8925334	0.041504
27	oar3_OAR〉	8925724	0.060748
27	oar3_OAR〉	8932728	0.060748
27	oar3_OAR〉	8933479	0.162741
27	oar3_OAR〉	8938721	-0.02049
27	oar3_OAR〉	8944083	0.022281
27	oar3_OAR〉	8948815	0.050218

27	oar3_OAR〉	8949209	-0.00181
27	oar3_OAR〉	8954327	-0.03178
27	oar3_OAR〉	8960267	0.055777
27	oar3_OAR〉	8960560	0.021415
27	oar3_OAR〉	8962465	0.019294
27	oar3_OAR〉	8982758	0.067884
27	oar3_OAR〉	8983430	-0.00395
27	oar3_OAR〉	8988992	-0.00395
27	oar3_OAR〉	8991052	0.01417
27	oar3_OAR〉	8994818	-0.00395
27	oar3_OAR〉	8997841	0.013791
27	oar3_OAR〉	9002575	-0.01224
27	oar3_OAR〉	9003208	0.02306
27	oar3_OAR〉	9014183	-0.01224
27	oar3_OAR〉	9015427	0.037898
27	oar3_OAR〉	9022942	-0.01224
27	oar3_OAR〉	9031119	-0.00181
27	oar3_OAR〉	9035030	-0.00585
27	oar3_OAR〉	9036324	-0.00181
27	oar3_OAR〉	9047508	0.012172
27	oar3_OAR〉	9048266	-0.0236
27	oar3_OAR〉	9053461	-0.01059
27	oar3_OAR〉	9054477	-0.00585
27	oar3_OAR〉	9058399	0.082366
27	oar3_OAR〉	9058461	0.038332
27	oar3_OAR〉	9060413	0.031225
27	oar3_OAR〉	9062297	0.009692
27	oar3_OAR〉	9065401	0.031225
27	oar3_OAR〉	9074059	-0.02184
27	oar3_OAR〉	9074133	-0.00585
27	oar3_OAR〉	9082188	0.069768
27	oar3_OAR〉	9096083	0.011389
27	oar3_OAR〉	9097295	-0.00725
27	oar3_OAR〉	9100218	0.012406
27	oar3_OAR〉	9100392	0.011116
27	oar3_OAR〉	9102175	-0.00585
27	oar3_OAR〉	9113261	-0.01251
27	oar3_OAR〉	9114977	-0.02806
27	oar3_OAR〉	9121581	-0.01386
27	oar3_OAR〉	9122352	-0.02184
27	oar3_OAR〉	9123286	-0.04633
27	oar3_OAR〉	9128412	-0.00287
27	oar3_OAR〉	9128608	-0.00287
27	oar3_OAR〉	9134727	-0.00585
27	oar3_OAR〉	9138596	-0.02545
27	oar3_OAR〉	9149480	-0.0194
27	oar3_OAR〉	9153348	0.000605
27	oar3_OAR〉	9161931	0.003894
27	oar3_OAR〉	9163926	0.044724
27	oar3_OAR〉	9175223	-0.02184
27	oar3_OAR〉	9190228	0.012291
27	oar3_OAR〉	9190521	0.012291
27	oar3_OAR〉	9199549	-0.02184
27	oar3_OAR〉	9208169	-0.02582

27	oar3_OAR〉	9214850	0.017104
27	oar3_OAR〉	9222659	0.088825
27	oar3_OAR〉	9223791	0.098649
27	oar3_OAR〉	9229053	0.004105
27	oar3_OAR〉	9234020	0.004105
27	oar3_OAR〉	9248108	0.054445
27	oar3_OAR〉	9251710	0.050776
27	oar3_OAR〉	9252861	0.054445
27	oar3_OAR〉	9260778	0.044724
27	oar3_OAR〉	9261822	-0.00931
27	oar3_OAR〉	9276586	0.054281
27	oar3_OAR〉	9276678	0.010876
27	oar3_OAR〉	9281486	0.0204
27	oar3_OAR〉	9288474	NA
27	oar3_OAR〉	9289025	0.002309
27	oar3_OAR〉	9289276	0.021518
27	oar3_OAR〉	9294868	0.188378
27	oar3_OAR〉	9295617	-0.00517
27	oar3_OAR〉	9305290	0.188378
27	oar3_OAR〉	9309599	0.045744
27	oar3_OAR〉	9314912	-0.01069
27	oar3_OAR〉	9315247	0.060364
27	oar3_OAR〉	9319811	-0.01069
27	oar3_OAR〉	9323257	0.051111
27	oar3_OAR〉	9323626	-0.00153
27	oar3_OAR〉	9331486	0.026834
27	oar3_OAR〉	9333629	-0.02267
27	oar3_OAR〉	9337533	-0.02267
27	oar3_OAR〉	9343711	-0.02267
27	oar3_OAR〉	9354267	-0.02719
27	oar3_OAR〉	9367296	-0.02065
27	oar3_OAR〉	9380720	-0.01747
27	oar3_OAR〉	9380861	-0.0245
27	oar3_OAR〉	9392101	0.023997
27	oar3_OAR〉	9392164	0.023997
27	oar3_OAR〉	9403053	-0.01958
27	oar3_OAR〉	9424134	-0.02883
27	oar3_OAR〉	9430303	-0.02502
27	oar3_OAR〉	9439451	0.000889
27	oar3_OAR〉	9447696	0.000889
27	oar3_OAR〉	9452240	-0.02419
27	oar3_OAR〉	9467072	-0.03098
27	oar3_OAR〉	9469176	0.024688
27	oar3_OAR〉	9477832	0.027249
27	oar3_OAR〉	9478478	-0.00057
27	oar3_OAR〉	9491701	0.000889
27	oar3_OAR〉	9493603	-0.01611
27	oar3_OAR〉	9494014	-0.02295
27	oar3_OAR〉	9502213	-0.02295
27	oar3_OAR〉	9507909	0.015023
27	oar3_OAR〉	9508142	-0.01611
27	oar3_OAR〉	9512284	0.051588
27	oar3_OAR〉	9521048	0.051588
27	oar3_OAR〉	9525720	-0.02838

27	oar3_OAR〉	9533241	0.011067
27	oar3_OAR〉	9537378	0.072163
27	oar3_OAR〉	9545365	-0.00166
27	oar3_OAR〉	9546426	0.072163
27	oar3_OAR〉	9550045	0.03112
27	oar3_OAR〉	9557484	-0.01762
27	oar3_OAR〉	9557547	-0.02374
27	oar3_OAR〉	9557741	-0.00016
27	oar3_OAR〉	9565283	0.080072
27	oar3_OAR〉	9570697	0.080072
27	oar3_OAR〉	9571051	-0.00462
27	oar3_OAR〉	9579609	0.031833
27	oar3_OAR〉	9584928	0.014046
27	oar3_OAR〉	9589492	0.054446
27	oar3_OAR〉	9592144	0.08344
27	oar3_OAR〉	9592146	-0.04195
27	oar3_OAR〉	9603497	0.119507
27	oar3_OAR〉	9622269	0.169618
27	oar3_OAR〉	9632680	0.169618
27	oar3_OAR〉	9633852	0.12394
27	oar3_OAR〉	9634563	0.075125
27	oar3_OAR〉	9642334	0.12394
27	oar3_OAR〉	9653040	-0.01496
27	oar3_OAR〉	9656905	0.075125
27	oar3_OAR〉	9659227	-0.01496
27	oar3_OAR〉	9661194	-0.02348
27	oar3_OAR〉	9663872	-0.0281
27	oar3_OAR〉	9665279	0.008296
27	oar3_OAR〉	9675374	0.008296
27	oar3_OAR〉	9677420	0.241379
27	oar3_OAR〉	9686428	-0.02035
27	oar3_OAR〉	9687452	-0.02488
27	oar3_OAR〉	9694820	-0.01737
27	oar3_OAR〉	9698152	0.087331
27	oar3_OAR〉	9708108	-0.00731
27	oar3_OAR〉	9712840	0.081175
27	oar3_OAR〉	9716512	-0.02242
27	oar3_OAR〉	9716923	0.03057
27	oar3_OAR〉	9721483	0.077592
27	oar3_OAR〉	9722167	0.03057
27	oar3_OAR〉	9728912	-0.01851
27	oar3_OAR〉	9730762	0.106306
27	oar3_OAR〉	9739704	0.063106
27	oar3_OAR〉	9740443	-0.02242
27	oar3_OAR〉	9743850	-0.02231
27	oar3_OAR〉	9749428	-0.01953
27	oar3_OAR〉	9756491	-0.02533
27	oar3_OAR〉	9757867	-0.0219
27	oar3_OAR〉	9759960	-0.01673
27	oar3_OAR〉	9782099	-0.01673
27	oar3_OAR〉	9786286	0.016379
27	oar3_OAR〉	9787711	0.011872
27	oar3_OAR〉	9791680	-0.01873
27	oar3_OAR〉	9793757	-0.00471

27	oar3_OAR〉	9796482	-0.00716
27	oar3_OAR〉	9806004	0.034221
27	oar3_OAR〉	9817151	0.034221
27	oar3_OAR〉	9824449	-0.00325
27	oar3_OAR〉	9827165	-0.00705
27	oar3_OAR〉	9829043	-0.02061
27	oar3_OAR〉	9840158	0.044019
27	oar3_OAR〉	9841330	0.044019
27	oar3_OAR〉	9842340	0.080754
27	oar3_OAR〉	9842826	0.026045
27	oar3_OAR〉	9848800	-0.02662
27	oar3_OAR〉	9860188	-0.0054
27	oar3_OAR〉	9863044	-0.01069
27	oar3_OAR〉	9864802	-0.02209
27	oar3_OAR〉	9867526	0.041221
27	oar3_OAR〉	9870501	-0.01069
27	oar3_OAR〉	9878047	-0.00325
27	oar3_OAR〉	9882896	0.046389
27	oar3_OAR〉	9884036	0.021134
27	oar3_OAR〉	9884162	0.062973
27	oar3_OAR〉	9886386	0.043831
27	oar3_OAR〉	9889216	0.11979
27	oar3_OAR〉	9894470	-0.02049
27	oar3_OAR〉	9895822	0.013573
27	oar3_OAR〉	9903896	0.008032
27	oar3_OAR〉	9917072	0.148276
27	oar3_OAR〉	9917342	0.210133
27	oar3_OAR〉	9927900	-0.00817
27	oar3_OAR〉	9928232	-0.00817
27	oar3_OAR〉	9931903	0.082377
27	oar3_OAR〉	9938677	-0.00856
27	oar3_OAR〉	9938916	-0.0104
27	oar3_OAR〉	9941487	-0.03388
27	oar3_OAR〉	9944608	-0.00035
27	oar3_OAR〉	9950123	0.010572
27	oar3_OAR〉	9950485	-0.03878
27	oar3_OAR〉	9958088	-0.02926
27	oar3_OAR〉	9958948	0.006706
27	oar3_OAR〉	9959570	-0.00866
27	oar3_OAR〉	9966415	0.099057
27	oar3_OAR〉	9969998	0.008296
27	oar3_OAR〉	9971338	0.005111
27	oar3_OAR〉	9972716	-0.01745
27	oar3_OAR〉	9973471	-0.00822
27	oar3_OAR〉	9981829	-0.00657
27	oar3_OAR〉	9983454	0.029261
27	oar3_OAR〉	9985603	-0.01585
27	oar3_OAR〉	9987485	-0.02027
27	oar3_OAR〉	9991455	0.013336
27	oar3_OAR〉	9995231	-0.01588
27	oar3_OAR〉	9996758	-0.02588
27	oar3_OAR〉	9997532	-0.02027
27	oar3_OAR〉	10001657	0.017279
27	oar3_OAR〉	10008883	-0.02895

27	oar3_OAR〉	10021953	0.006682
27	oar3_OAR〉	10024436	-0.00689
27	oar3_OAR〉	10030040	-0.00117
27	oar3_OAR〉	10032168	-0.00117
27	oar3_OAR〉	10032350	-0.00117
27	oar3_OAR〉	10034772	-0.03427
27	oar3_OAR〉	10041862	-0.03427
27	oar3_OAR〉	10045464	-0.03427
27	oar3_OAR〉	10056606	-0.00689
27	oar3_OAR〉	10057600	0.004924
27	oar3_OAR〉	10058253	-0.01128
27	oar3_OAR〉	10063698	-0.01117
27	oar3_OAR〉	10066044	-0.01117
27	oar3_OAR〉	10073018	-0.02647
27	oar3_OAR〉	10077228	-0.01117
27	oar3_OAR〉	10081497	-0.00639
27	oar3_OAR〉	10091280	0.012034
27	oar3_OAR〉	10099641	-0.00204
27	oar3_OAR〉	10105594	-0.01366
27	oar3_OAR〉	10106740	0.038705
27	oar3_OAR〉	10122516	-0.01366
27	oar3_OAR〉	10124656	-0.02353
27	oar3_OAR〉	10132332	-0.02576
27	oar3_OAR〉	10133673	-0.01129
27	oar3_OAR〉	10136089	0.011696
27	oar3_OAR〉	10146352	0.014442
27	oar3_OAR〉	10150930	-0.01872
27	oar3_OAR〉	10151837	-0.01434
27	oar3_OAR〉	10157275	0.008723
27	oar3_OAR〉	10158574	-0.00606
27	oar3_OAR〉	10170549	0.082222
27	oar3_OAR〉	10178566	0.007446
27	oar3_OAR〉	10178887	-0.03175
27	oar3_OAR〉	10185776	0.041221
27	oar3_OAR〉	10191731	-0.02308
27	oar3_OAR〉	10201682	0.007446
27	oar3_OAR〉	10208454	0.119533
27	oar3_OAR〉	10211864	NA
27	oar3_OAR〉	10214955	0.092117
27	oar3_OAR〉	10217274	0.041776
27	oar3_OAR〉	10221560	0.007446
27	oar3_OAR〉	10223142	-0.02951
27	oar3_OAR〉	10231575	0.088043
27	oar3_OAR〉	10232573	0.088043
27	oar3_OAR〉	10236564	-0.02957
27	oar3_OAR〉	10237043	0.01742
27	oar3_OAR〉	10241743	-0.02827
27	oar3_OAR〉	10242302	0.01742
27	oar3_OAR〉	10246980	0.088043
27	oar3_OAR〉	10247323	0.01742
27	oar3_OAR〉	10259960	0.055085
27	oar3_OAR〉	10260445	-0.02292
27	oar3_OAR〉	10260560	-0.02292
27	oar3_OAR〉	10267271	-0.02292

27	oar3_OAR›	10269959	0.065575
27	oar3_OAR›	10270958	0.087039
27	oar3_OAR›	10274634	-0.00571
27	oar3_OAR›	10278372	0.009127
27	oar3_OAR›	10280751	-0.01731
27	oar3_OAR›	10284168	-0.0085
27	oar3_OAR›	10286559	-0.01731
27	oar3_OAR›	10287706	-0.00556
27	oar3_OAR›	10291883	-0.01731
27	oar3_OAR›	10300378	-0.02013
27	oar3_OAR›	10301509	-0.00462
27	oar3_OAR›	10302482	-0.01121
27	oar3_OAR›	10310681	-0.00462
27	oar3_OAR›	10312652	0.104241
27	oar3_OAR›	10313315	-0.00462
27	oar3_OAR›	10319068	-0.00462
27	oar3_OAR›	10323120	0.08864
27	oar3_OAR›	10328794	-0.02995
27	oar3_OAR›	10329744	-0.00462
27	oar3_OAR›	10333961	0.05869
27	oar3_OAR›	10340057	0.024294
27	oar3_OAR›	10340552	-0.02345
27	oar3_OAR›	10343654	-0.00462
27	oar3_OAR›	10345881	-0.02395
27	oar3_OAR›	10347136	-0.0245
27	oar3_OAR›	10350678	-0.02395
27	oar3_OAR›	10352691	-0.00462
27	oar3_OAR›	10357342	0.003762
27	oar3_OAR›	10360855	-0.00083
27	oar3_OAR›	10363090	0.002868
27	oar3_OAR›	10363265	0.004823
27	oar3_OAR›	10373046	-0.00609
27	oar3_OAR›	10377693	0.002868
27	oar3_OAR›	10379898	-0.0216
27	oar3_OAR›	10385242	-0.0216
27	oar3_OAR›	10389421	-0.0216
27	oar3_OAR›	10391448	-0.00462
27	oar3_OAR›	10395648	0.014309
27	oar3_OAR›	10396445	0.046868
27	oar3_OAR›	10398449	0.053104
27	oar3_OAR›	10404930	0.019729
27	oar3_OAR›	10406022	0.029915
27	oar3_OAR›	10418044	-0.02082
27	oar3_OAR›	10418091	-0.01188
27	oar3_OAR›	10429659	-0.01779
27	oar3_OAR›	10431820	-0.02182
27	oar3_OAR›	10438901	-0.01779
27	oar3_OAR›	10445210	-0.02182
27	oar3_OAR›	10450414	0.016959
27	oar3_OAR›	10458065	-0.02004
27	oar3_OAR›	10468229	-0.0151
27	oar3_OAR›	10469147	0.013911
27	oar3_OAR›	10484476	-0.0229
27	oar3_OAR›	10508327	0.013743

27	oar3_OAR›	10508502	-0.02565
27	oar3_OAR›	10510262	0.013743
27	oar3_OAR›	10515385	0.066643
27	oar3_OAR›	10518806	-0.00535
27	oar3_OAR›	10524910	0.066643
27	oar3_OAR›	10525030	0.066643
27	oar3_OAR›	10530377	-0.00435
27	oar3_OAR›	10533035	-0.0126
27	oar3_OAR›	10535890	0.04569
27	oar3_OAR›	10540715	-0.0268
27	oar3_OAR›	10544856	0.00193
27	oar3_OAR›	10548642	0.013952
27	oar3_OAR›	10554637	0.04569
27	oar3_OAR›	10565093	0.04569
27	oar3_OAR›	10566583	0.04569
27	oar3_OAR›	10569040	0.031551
27	oar3_OAR›	10578479	-0.02687
27	oar3_OAR›	10581002	0.04569
27	oar3_OAR›	10591133	0.04569
27	oar3_OAR›	10591227	0.04569
27	oar3_OAR›	10593257	-0.03202
27	oar3_OAR›	10596754	0.04569
27	oar3_OAR›	10605860	-0.03183
27	oar3_OAR›	10613135	-0.02627
27	oar3_OAR›	10614173	0.013773
27	oar3_OAR›	10619265	-0.02707
27	oar3_OAR›	10622531	-0.03145
27	oar3_OAR›	10625145	-0.03145
27	oar3_OAR›	10629801	0.001835
27	oar3_OAR›	10633128	0.021839
27	oar3_OAR›	10639089	0.001657
27	oar3_OAR›	10644314	-0.00232
27	oar3_OAR›	10656083	0.021839
27	oar3_OAR›	10656706	0.066678
27	oar3_OAR›	10664232	-0.00327
27	oar3_OAR›	10666292	0.001835
27	oar3_OAR›	10670086	0.04569
27	oar3_OAR›	10672670	-0.02049
27	oar3_OAR›	10674010	0.01985
27	oar3_OAR›	10679194	0.003661
27	oar3_OAR›	10680657	-0.02856
27	oar3_OAR›	10683080	-0.02926
27	oar3_OAR›	10688483	0.07548
27	oar3_OAR›	10691345	-0.01567
27	oar3_OAR›	10699362	-0.00188
27	oar3_OAR›	10702406	-0.00188
27	oar3_OAR›	10702861	0.078631
27	oar3_OAR›	10709438	-0.03189
27	oar3_OAR›	10711136	-0.00188
27	oar3_OAR›	10722809	-0.02543
27	oar3_OAR›	10723029	-0.02543
27	oar3_OAR›	10723828	-0.02323
27	oar3_OAR›	10732171	0.001015
27	oar3_OAR›	10740184	0.050547

27	oar3_OAR›	10742383	-0.02941
27	oar3_OAR›	10744265	-0.00144
27	oar3_OAR›	10748200	0.007039
27	oar3_OAR›	10748271	0.049673
27	oar3_OAR›	10751745	-0.00933
27	oar3_OAR›	10754019	0.055942
27	oar3_OAR›	10755427	0.055942
27	oar3_OAR›	10756526	0.055942
27	oar3_OAR›	10758352	0.060256
27	oar3_OAR›	10766415	0.109557
27	oar3_OAR›	10768828	0.000605
27	oar3_OAR›	10770762	-0.01273
27	oar3_OAR›	10780651	0.034546
27	oar3_OAR›	10784052	-0.03946
27	oar3_OAR›	10784622	0.02374
27	oar3_OAR›	10792847	-0.02759
27	oar3_OAR›	10799009	-0.00032
27	oar3_OAR›	10799383	0.046064
27	oar3_OAR›	10805354	-0.02759
27	oar3_OAR›	10805629	-0.02759
27	oar3_OAR›	10809431	-0.01595
27	oar3_OAR›	10810305	-0.00508
27	oar3_OAR›	10819532	-0.01519
27	oar3_OAR›	10824506	-0.0059
27	oar3_OAR›	10826780	NA
27	oar3_OAR›	10829862	-0.02351
27	oar3_OAR›	10832310	-0.01415
27	oar3_OAR›	10833496	0.005869
27	oar3_OAR›	10837248	-0.01241
27	oar3_OAR›	10850284	-0.00334
27	oar3_OAR›	10850560	-0.00334
27	oar3_OAR›	10913347	0.057858
27	oar3_OAR›	10914330	0.003214
27	oar3_OAR›	10922478	0.019309
27	oar3_OAR›	10922695	0.019309
27	oar3_OAR›	10926158	-0.03423
27	oar3_OAR›	10928322	-0.02377
27	oar3_OAR›	10934284	-0.02035
27	oar3_OAR›	10941435	-0.02099
27	oar3_OAR›	10942052	-0.02099
27	oar3_OAR›	10947673	-0.02773
27	oar3_OAR›	10950186	-0.0223
27	oar3_OAR›	10950962	-0.02617
27	oar3_OAR›	10963889	-0.02078
27	oar3_OAR›	10966156	-0.0268
27	oar3_OAR›	10971600	0.033773
27	oar3_OAR›	10984960	-0.02292
27	oar3_OAR›	10985015	0.011887
27	oar3_OAR›	10985181	0.038968
27	oar3_OAR›	10994968	-0.01185
27	oar3_OAR›	10995584	0.027445
27	oar3_OAR›	11009152	0.00462
27	oar3_OAR›	11012529	0.00462
27	oar3_OAR›	11041843	0.039481

27	oar3_OAR›	11046607	0.009706
27	oar3_OAR›	11049701	0.002868
27	oar3_OAR›	11055027	-0.0061
27	oar3_OAR›	11057332	0.009706
27	oar3_OAR›	11065033	-0.02603
27	oar3_OAR›	11069747	-0.01755
27	oar3_OAR›	11072378	-0.02603
27	oar3_OAR›	11072801	-0.02603
27	oar3_OAR›	11104357	0.025367
27	oar3_OAR›	11104417	0.091169
27	oar3_OAR›	11107382	0.061193
27	oar3_OAR›	11108189	0.053123
27	oar3_OAR›	11113407	0.029155
27	oar3_OAR›	11116166	0.104349
27	oar3_OAR›	11118336	0.067504
27	oar3_OAR›	11118454	-0.01262
27	oar3_OAR›	11121686	0.035181
27	oar3_OAR›	11125017	0.004325
27	oar3_OAR›	11128886	0.078934
27	oar3_OAR›	11129981	0.024014
27	oar3_OAR›	11130361	0.005395
27	oar3_OAR›	11137604	0.018459
27	oar3_OAR›	11143825	0.032217
27	oar3_OAR›	11143896	-0.01717
27	oar3_OAR›	11146672	0.030424
27	oar3_OAR›	11153466	-0.00287
27	oar3_OAR›	11156327	0.032217
27	oar3_OAR›	11157901	-0.00287
27	oar3_OAR›	11172620	-0.00817
27	oar3_OAR›	11176941	-0.02856
27	oar3_OAR›	11177678	-0.00322
27	oar3_OAR›	11179754	-0.00322
27	oar3_OAR›	11188318	-0.02205
27	oar3_OAR›	11192174	0.013063
27	oar3_OAR›	11199323	0.030998
27	oar3_OAR›	11199396	0.013063
27	oar3_OAR›	11214292	0.026757
27	oar3_OAR›	11218724	-0.02446
27	oar3_OAR›	11227468	0.0139
27	oar3_OAR›	11229292	0.000157
27	oar3_OAR›	11231369	0.000157
27	oar3_OAR›	11232435	0.000157
27	oar3_OAR›	11237479	0.073654
27	oar3_OAR›	11242139	0.023167
27	oar3_OAR›	11248944	0.006697
27	oar3_OAR›	11249548	0.046758
27	oar3_OAR›	11255309	-0.0299
27	oar3_OAR›	11255500	0.002242
27	oar3_OAR›	11256861	-0.0346
27	oar3_OAR›	11260982	0.034165
27	oar3_OAR›	11264933	-0.02984
27	oar3_OAR›	11265301	-0.02408
27	oar3_OAR›	11266366	-0.02984
27	oar3_OAR›	11267535	-0.01106

27	oar3_OAR〉	11272298	0.034165
27	oar3_OAR〉	11276461	-0.02374
27	oar3_OAR〉	11277869	0.034165
27	oar3_OAR〉	11278117	-0.01216
27	oar3_OAR〉	11283922	0.034165
27	oar3_OAR〉	11288886	0.034165
27	oar3_OAR〉	11289302	0.020885
27	oar3_OAR〉	11292000	-0.01527
27	oar3_OAR〉	11298123	-0.01396
27	oar3_OAR〉	11304069	-0.01567
27	oar3_OAR〉	11306221	-0.02533
27	oar3_OAR〉	11309306	-0.00395
27	oar3_OAR〉	11311207	-0.01611
27	oar3_OAR〉	11311426	-0.00603
27	oar3_OAR〉	11316049	-0.01543
27	oar3_OAR〉	11316758	-0.01493
27	oar3_OAR〉	11320693	-0.00152
27	oar3_OAR〉	11321540	0.004281
27	oar3_OAR〉	11327991	0.006706
27	oar3_OAR〉	11328534	5.87E-06
27	oar3_OAR〉	11336406	-0.01493
27	oar3_OAR〉	11337320	-0.00718
27	oar3_OAR〉	11341759	-0.0051
27	oar3_OAR〉	11347766	0.007863
27	oar3_OAR〉	11351641	0.007863
27	oar3_OAR〉	11357049	0.134299
27	oar3_OAR〉	11359949	0.166482
27	oar3_OAR〉	11363991	0.156251
27	oar3_OAR〉	11365604	0.109331
27	oar3_OAR〉	11370731	0.140063
27	oar3_OAR〉	11378599	0.073459
27	oar3_OAR〉	11380514	0.064924
27	oar3_OAR〉	11381368	0.064924
27	oar3_OAR〉	11388636	0.224073
27	oar3_OAR〉	11389485	0.224073
27	oar3_OAR〉	11393107	0.224073
27	oar3_OAR〉	11395855	0.061323
27	oar3_OAR〉	11396584	0.061323
27	oar3_OAR〉	11406765	0.000295
27	oar3_OAR〉	11406850	0.000295
27	oar3_OAR〉	11411784	0.040241
27	oar3_OAR〉	11419730	0.023389
27	oar3_OAR〉	11429669	0.082222
27	oar3_OAR〉	11430479	0.038594
27	oar3_OAR〉	11434173	0.057401
27	oar3_OAR〉	11440296	0.057401
27	oar3_OAR〉	11440612	0.057401
27	oar3_OAR〉	11446648	0.038594
27	oar3_OAR〉	11451609	0.03648
27	oar3_OAR〉	11455674	0.008723
27	oar3_OAR〉	11461368	0.033773
27	oar3_OAR〉	11471660	0.039481
27	oar3_OAR〉	11472692	-0.01931
27	oar3_OAR〉	11473959	-0.0225

27	oar3_OAR›	11480208	-0.01846
27	oar3_OAR›	11481226	-0.01931
27	oar3_OAR›	11486666	0.082222
27	oar3_OAR›	11496285	-0.016
27	oar3_OAR›	11500334	-0.00267
27	oar3_OAR›	11505355	-0.0096
27	oar3_OAR›	11505754	-0.01062
27	oar3_OAR›	11523754	-0.0096
27	oar3_OAR›	11525156	NA
27	oar3_OAR›	11536913	-0.00486
27	oar3_OAR›	11554158	-0.02529
27	oar3_OAR›	11565000	-0.0246
27	oar3_OAR›	11565531	-0.0246
27	oar3_OAR›	11565810	NA
27	oar3_OAR›	11567218	-0.01583
27	oar3_OAR›	11572404	-0.0246
27	oar3_OAR›	11578510	-0.00581
27	oar3_OAR›	11579331	-0.02765
27	oar3_OAR›	11581160	0.020343
27	oar3_OAR›	11590466	-0.01227
27	oar3_OAR›	11600939	0.017279
27	oar3_OAR›	11607083	-0.0219
27	oar3_OAR›	11611251	-0.00075
27	oar3_OAR›	11619276	-0.01525
27	oar3_OAR›	11619419	-0.01525
27	oar3_OAR›	11628251	-0.00075
27	oar3_OAR›	11634589	0.041221
27	oar3_OAR›	11647475	-0.01614
27	oar3_OAR›	11649600	-0.00252
27	oar3_OAR›	11660094	0.044724
27	oar3_OAR›	11678693	0.011638
27	oar3_OAR›	11682148	-0.0298
27	oar3_OAR›	11687225	0.026045
27	oar3_OAR›	11763840	-0.00357
27	oar3_OAR›	11780947	-0.00788
27	oar3_OAR›	11814027	0.058602
27	oar3_OAR›	11826639	0.067309
27	oar3_OAR›	11827712	0.067309
27	oar3_OAR›	11827835	0.067309
27	oar3_OAR›	11835673	0.056679
27	oar3_OAR›	11837103	0.056679
27	oar3_OAR›	11838783	0.025205
27	oar3_OAR›	11845767	0.058702
27	oar3_OAR›	11847307	-0.01511
27	oar3_OAR›	11848791	0.005807
27	oar3_OAR›	11853575	-0.00575
27	oar3_OAR›	11853649	-0.01628
27	oar3_OAR›	11860321	-0.0055
27	oar3_OAR›	11860442	-0.0021
27	oar3_OAR›	11860774	-0.01069
27	oar3_OAR›	11861129	-0.01008
27	oar3_OAR›	11866552	-0.01735
27	oar3_OAR›	11868517	-0.01351
27	oar3_OAR›	11876081	-0.00691

27	oar3_OAR〉	11883496	0.013414
27	oar3_OAR〉	11885135	-0.00201
27	oar3_OAR〉	11885254	-0.00201
27	oar3_OAR〉	11890073	-0.02486
27	oar3_OAR〉	11894728	0.059856
27	oar3_OAR〉	11897608	0.065557
27	oar3_OAR〉	11898583	0.065557
27	oar3_OAR〉	11902824	-0.01206
27	oar3_OAR〉	11905520	0.005349
27	oar3_OAR〉	11909900	-0.01525
27	oar3_OAR〉	11910231	0.05171
27	oar3_OAR〉	11910880	-0.01525
27	oar3_OAR〉	11911422	0.065557
27	oar3_OAR〉	11921648	0.084595
27	oar3_OAR〉	11922925	0.052943
27	oar3_OAR〉	11936154	-0.00181
27	oar3_OAR〉	11937778	0.043015
27	oar3_OAR〉	11941146	-0.00928
27	oar3_OAR〉	11942438	0.104982
27	oar3_OAR〉	11944866	0.084729
27	oar3_OAR〉	11948537	-0.02242
27	oar3_OAR〉	11955584	0.061306
27	oar3_OAR〉	11955912	0.070068
27	oar3_OAR〉	11964511	0.015525
27	oar3_OAR〉	11964589	0.039722
27	oar3_OAR〉	11966677	-0.02242
27	oar3_OAR〉	11974415	0.025709
27	oar3_OAR〉	11974623	0.025709
27	oar3_OAR〉	11978299	-0.00381
27	oar3_OAR〉	11980912	0.041837
27	oar3_OAR〉	11986901	-0.00928
27	oar3_OAR〉	11995325	0.012941
27	oar3_OAR〉	12010340	0.009572
27	oar3_OAR〉	12013193	0.094701
27	oar3_OAR〉	12014836	0.05391
27	oar3_OAR〉	12021715	0.06501
27	oar3_OAR〉	12029517	0.004606
27	oar3_OAR〉	12030717	0.009639
27	oar3_OAR〉	12031726	-0.00787
27	oar3_OAR〉	12037422	-0.01093
27	oar3_OAR〉	12045484	0.011208
27	oar3_OAR〉	12048573	-0.00928
27	oar3_OAR〉	12052798	0.044058
27	oar3_OAR〉	12065802	-0.01657
27	oar3_OAR〉	12070692	-0.01776
27	oar3_OAR〉	12075901	-0.01776
27	oar3_OAR〉	12081780	0.062765
27	oar3_OAR〉	12083615	-0.01776
27	oar3_OAR〉	12084734	-0.01776
27	oar3_OAR〉	12090804	0.017281
27	oar3_OAR〉	12098087	-0.00287
27	oar3_OAR〉	12100359	0.060548
27	oar3_OAR〉	12105866	0.025529
27	oar3_OAR〉	12108917	-0.00287

27	oar3_OAR›	12115330	-0.00287
27	oar3_OAR›	12115833	0.042883
27	oar3_OAR›	12116202	-0.00287
27	oar3_OAR›	12121454	0.039638
27	oar3_OAR›	12127295	0.04359
27	oar3_OAR›	12128352	-0.00287
27	oar3_OAR›	12129273	0.041235
27	oar3_OAR›	12131061	-0.00209
27	oar3_OAR›	12138226	0.033491
27	oar3_OAR›	12146169	0.063977
27	oar3_OAR›	12146314	-0.00287
27	oar3_OAR›	12148547	0.005534
27	oar3_OAR›	12149790	0.008723
27	oar3_OAR›	12155247	0.00481
27	oar3_OAR›	12158137	-0.00169
27	oar3_OAR›	12158310	0.021129
27	oar3_OAR›	12163570	-0.01594
27	oar3_OAR›	12168063	0.097358
27	oar3_OAR›	12168519	-0.00751
27	oar3_OAR›	12169234	0.007853
27	oar3_OAR›	12172206	0.025097
27	oar3_OAR›	12177614	-0.00784
27	oar3_OAR›	12179292	-0.00784
27	oar3_OAR›	12182388	0.002898
27	oar3_OAR›	12186512	0.002898
27	oar3_OAR›	12188536	-0.02759
27	oar3_OAR›	12189388	-0.02772
27	oar3_OAR›	12193197	-0.01166
27	oar3_OAR›	12193841	-0.00147
27	oar3_OAR›	12200734	0.010341
27	oar3_OAR›	12203815	-0.01166
27	oar3_OAR›	12206059	-0.01356
27	oar3_OAR›	12220432	0.00406
27	oar3_OAR›	12221673	0.025112
27	oar3_OAR›	12230950	-0.00748
27	oar3_OAR›	12232895	-0.01416
27	oar3_OAR›	12241691	0.005714
27	oar3_OAR›	12241949	-0.01974
27	oar3_OAR›	12247592	-0.02834
27	oar3_OAR›	12253457	0.049985
27	oar3_OAR›	12253732	-0.0134
27	oar3_OAR›	12258562	-0.02603
27	oar3_OAR›	12261141	0.035788
27	oar3_OAR›	12261899	-0.00895
27	oar3_OAR›	12270997	0.016062
27	oar3_OAR›	12271267	0.018887
27	oar3_OAR›	12276998	-0.03208
27	oar3_OAR›	12286161	-0.02831
27	oar3_OAR›	12289318	-0.02118
27	oar3_OAR›	12292895	0.030966
27	oar3_OAR›	12292946	0.062026
27	oar3_OAR›	12294976	-0.02458
27	oar3_OAR›	12302716	0.128677
27	oar3_OAR›	12310464	0.026805

27	oar3_OAR›	12313129	0.008723
27	oar3_OAR›	12321202	0.008723
27	oar3_OAR›	12323312	0.054933
27	oar3_OAR›	12336155	-0.02926
27	oar3_OAR›	12337792	0.022748
27	oar3_OAR›	12343390	-0.00836
27	oar3_OAR›	12347935	-0.0065
27	oar3_OAR›	12350916	-0.02593
27	oar3_OAR›	12361085	-0.02926
27	oar3_OAR›	12362330	-0.02926
27	oar3_OAR›	12362641	-0.03287
27	oar3_OAR›	12373906	-0.01735
27	oar3_OAR›	12396112	-0.02834
27	oar3_OAR›	12406986	0.009985
27	oar3_OAR›	12407251	0.03603
27	oar3_OAR›	12407334	0.017915
27	oar3_OAR›	12413574	0.052244
27	oar3_OAR›	12414776	0.002668
27	oar3_OAR›	12418900	-0.00014
27	oar3_OAR›	12424465	-0.02547
27	oar3_OAR›	12428202	0.052244
27	oar3_OAR›	12433357	-0.00606
27	oar3_OAR›	12434256	0.020068
27	oar3_OAR›	12439029	-0.02208
27	oar3_OAR›	12452345	0.030422
27	oar3_OAR›	12454805	0.131348
27	oar3_OAR›	12462355	0.127708
27	oar3_OAR›	12463667	0.156077
27	oar3_OAR›	12466380	0.095767
27	oar3_OAR›	12476569	0.155705
27	oar3_OAR›	12476788	0.095361
27	oar3_OAR›	12477467	-0.00606
27	oar3_OAR›	12487362	-0.013
27	oar3_OAR›	12487863	0.077259
27	oar3_OAR›	12492254	0.115258
27	oar3_OAR›	12492327	0.115258
27	oar3_OAR›	12492632	-0.01069
27	oar3_OAR›	12497910	0.226104
27	oar3_OAR›	12504359	-0.0291
27	oar3_OAR›	12507023	0.226104
27	oar3_OAR›	12514758	-0.03183
27	oar3_OAR›	12516239	0.062474
27	oar3_OAR›	12528213	-0.01558
27	oar3_OAR›	12532907	-0.02896
27	oar3_OAR›	12536252	0.161374
27	oar3_OAR›	12540286	-0.0265
27	oar3_OAR›	12541567	-0.0265
27	oar3_OAR›	12541576	0.033517
27	oar3_OAR›	12553856	-0.0265
27	oar3_OAR›	12556640	-0.02514
27	oar3_OAR›	12557946	0.011202
27	oar3_OAR›	12565953	0.056386
27	oar3_OAR›	12575191	0.008491
27	oar3_OAR›	12575399	0.101047

27	oar3_OAR〉	12576879	0.008491
27	oar3_OAR〉	12584123	-0.0346
27	oar3_OAR〉	12592926	-0.0246
27	oar3_OAR〉	12593157	-0.02951
27	oar3_OAR〉	12593961	0.011007
27	oar3_OAR〉	12600917	-0.02159
27	oar3_OAR〉	12603421	0.007513
27	oar3_OAR〉	12603716	0.107041
27	oar3_OAR〉	12603723	0.007513
27	oar3_OAR〉	12614493	0.085695
27	oar3_OAR〉	12614553	0.026045
27	oar3_OAR〉	12621330	-0.0085
27	oar3_OAR〉	12623779	0.080619
27	oar3_OAR〉	12626598	0.04711
27	oar3_OAR〉	12627456	-0.03081
27	oar3_OAR〉	12632377	0.032608
27	oar3_OAR〉	12632753	-0.03518
27	oar3_OAR〉	12640013	0.022201
27	oar3_OAR〉	12640667	0.017238
27	oar3_OAR〉	12648954	0.027215
27	oar3_OAR〉	12649020	-0.01265
27	oar3_OAR〉	12650881	0.113583
27	oar3_OAR〉	12659747	0.011643
27	oar3_OAR〉	12660836	-0.01846
27	oar3_OAR〉	12662197	0.005819
27	oar3_OAR〉	12669096	-0.01021
27	oar3_OAR〉	12672846	-0.02498
27	oar3_OAR〉	12680526	0.011643
27	oar3_OAR〉	12681405	-0.02353
27	oar3_OAR〉	12686061	-0.02353
27	oar3_OAR〉	12691206	0.000215
27	oar3_OAR〉	12693659	0.002195
27	oar3_OAR〉	12693869	-0.0275
27	oar3_OAR〉	12694080	-0.02189
27	oar3_OAR〉	12704314	-0.00049
27	oar3_OAR〉	12705833	-0.0236
27	oar3_OAR〉	12712333	-0.02807
27	oar3_OAR〉	12719742	-0.0158
27	oar3_OAR〉	12720867	-0.0236
27	oar3_OAR〉	12721825	0.011643
27	oar3_OAR〉	12722874	0.010288
27	oar3_OAR〉	12727524	0.010288
27	oar3_OAR〉	12733656	0.010288
27	oar3_OAR〉	12734700	0.010288
27	oar3_OAR〉	12735052	-0.01021
27	oar3_OAR〉	12740335	0.001266
27	oar3_OAR〉	12746698	0.031432
27	oar3_OAR〉	12749088	0.031432
27	oar3_OAR〉	12751870	0.007039
27	oar3_OAR〉	12763994	0.059225
27	oar3_OAR〉	12782271	0.059225
27	oar3_OAR〉	12796471	0.016713
27	oar3_OAR〉	12798959	-0.0148
27	oar3_OAR〉	12808113	0.053273

27	oar3_OAR›	12811262	-0.01787
27	oar3_OAR›	12814803	-0.00901
27	oar3_OAR›	12818877	-0.00681
27	oar3_OAR›	12820527	0.018804
27	oar3_OAR›	12821067	0.028602
27	oar3_OAR›	12821134	0.018804
27	oar3_OAR›	12826538	-0.01089
27	oar3_OAR›	12833306	0.018804
27	oar3_OAR›	12838808	0.018459
27	oar3_OAR›	12841359	-0.01982
27	oar3_OAR›	12843837	0.044633
27	oar3_OAR›	12849886	0.003713
27	oar3_OAR›	12850150	0.026045
27	oar3_OAR›	12858973	-0.02973
27	oar3_OAR›	12859192	-0.02973
27	oar3_OAR›	12859890	0.031311
27	oar3_OAR›	12863565	0.039725
27	oar3_OAR›	12871354	0.140528
27	oar3_OAR›	12871949	-0.00289
27	oar3_OAR›	12877862	0.048356
27	oar3_OAR›	12883452	0.056621
27	oar3_OAR›	12884152	0.056621
27	oar3_OAR›	12885088	0.07445
27	oar3_OAR›	12894279	0.056621
27	oar3_OAR›	12894610	0.060833
27	oar3_OAR›	12897041	-0.04015
27	oar3_OAR›	12903234	0.029185
27	oar3_OAR›	12906567	0.139126
27	oar3_OAR›	12906670	0.009927
27	oar3_OAR›	12910083	0.046186
27	oar3_OAR›	12911787	0.004308
27	oar3_OAR›	12913267	0.029185
27	oar3_OAR›	12914453	-0.00534
27	oar3_OAR›	12919618	-0.00314
27	oar3_OAR›	12920811	-0.0127
27	oar3_OAR›	12924867	0.079731
27	oar3_OAR›	12926945	0.05172
27	oar3_OAR›	12927383	0.05172
27	oar3_OAR›	12940993	0.030586
27	oar3_OAR›	12943243	0.077419
27	oar3_OAR›	12954319	0.014442
27	oar3_OAR›	12956469	0.030586
27	oar3_OAR›	12958410	0.035942
27	oar3_OAR›	12962043	0.030586
27	oar3_OAR›	12967082	0.027907
27	oar3_OAR›	12967748	-0.03147
27	oar3_OAR›	12968208	0.027907
27	oar3_OAR›	12969991	-0.02367
27	oar3_OAR›	12974694	0.065346
27	oar3_OAR›	12976987	-0.0156
27	oar3_OAR›	12980029	-0.0137
27	oar3_OAR›	12983765	-0.01032
27	oar3_OAR›	12992076	0.023228
27	oar3_OAR›	13001656	0.011019

27	oar3_OAR›	13006179	0.026886
27	oar3_OAR›	13006690	-0.02344
27	oar3_OAR›	13007233	0.044724
27	oar3_OAR›	13015524	0.044724
27	oar3_OAR›	13016765	0.082222
27	oar3_OAR›	13017054	0.037433
27	oar3_OAR›	13018303	0.000135
27	oar3_OAR›	13025961	0.054442
27	oar3_OAR›	13028518	0.032298
27	oar3_OAR›	13033013	0.005701
27	oar3_OAR›	13033565	0.062765
27	oar3_OAR›	13041069	0.001737
27	oar3_OAR›	13041311	-0.01197
27	oar3_OAR›	13042841	-0.0133
27	oar3_OAR›	13046693	0.001737
27	oar3_OAR›	13054405	-0.02462
27	oar3_OAR›	13055558	-0.02462
27	oar3_OAR›	13056922	0.057765
27	oar3_OAR›	13066153	0.023275
27	oar3_OAR›	13077839	0.003811
27	oar3_OAR›	13080904	0.038703
27	oar3_OAR›	13089390	0.057073
27	oar3_OAR›	13097400	-0.02778
27	oar3_OAR›	13099618	0.057073
27	oar3_OAR›	13107329	-0.01089
27	oar3_OAR›	13107458	0.034656
27	oar3_OAR›	13109466	0.001452
27	oar3_OAR›	13114900	-0.01628
27	oar3_OAR›	13125611	0.010252
27	oar3_OAR›	13125907	0.060883
27	oar3_OAR›	13129235	0.029372
27	oar3_OAR›	13134636	0.029879
27	oar3_OAR›	13135247	0.029879
27	oar3_OAR›	13161222	0.044491
27	oar3_OAR›	13168018	0.029879
27	oar3_OAR›	13171114	0.017106
27	oar3_OAR›	13175378	0.043921
27	oar3_OAR›	13178046	0.049753
27	oar3_OAR›	13192238	0.043921
27	oar3_OAR›	13195725	0.060402
27	oar3_OAR›	13195957	0.043921
27	oar3_OAR›	13200834	0.060402
27	oar3_OAR›	13207201	0.025891
27	oar3_OAR›	13210292	0.025891
27	oar3_OAR›	13215477	-0.03518
27	oar3_OAR›	13217339	-0.03518
27	oar3_OAR›	13227640	0.039207
27	oar3_OAR›	13230492	-0.00734
27	oar3_OAR›	13232085	0.063401
27	oar3_OAR›	13239242	0.043078
27	oar3_OAR›	13240011	0.050868
27	oar3_OAR›	13252640	0.030671
27	oar3_OAR›	13253172	0.01726
27	oar3_OAR›	13263808	-0.00901

27	oar3_OAR⟩	13264656	0.01726
27	oar3_OAR⟩	13266202	0.01726
27	oar3_OAR⟩	13273025	0.01726
27	oar3_OAR⟩	13275877	0.012361
27	oar3_OAR⟩	13301276	-0.01379
27	oar3_OAR⟩	13303078	-0.01379
27	oar3_OAR⟩	13304975	-0.01379
27	oar3_OAR⟩	13310715	-0.01379
27	oar3_OAR⟩	13327547	-0.01379
27	oar3_OAR⟩	13327915	-0.00606
27	oar3_OAR⟩	13330982	-0.01379
27	oar3_OAR⟩	13349223	-0.01379
27	oar3_OAR⟩	13358143	-0.01379
27	oar3_OAR⟩	13369586	-0.01379
27	oar3_OAR⟩	13370478	-0.01379
27	oar3_OAR⟩	13381814	-0.01379
27	oar3_OAR⟩	13385286	-0.0253
27	oar3_OAR⟩	13392472	-0.01379
27	oar3_OAR⟩	13399122	-0.00606
27	oar3_OAR⟩	13401471	-0.02176
27	oar3_OAR⟩	13411608	-0.02176
27	oar3_OAR⟩	13413424	-0.00606
27	oar3_OAR⟩	13416714	-0.00606
27	oar3_OAR⟩	13424783	-0.00606
27	oar3_OAR⟩	13437372	-0.00606
27	oar3_OAR⟩	13437742	-0.01887
27	oar3_OAR⟩	13447553	-0.01254
27	oar3_OAR⟩	13456811	-0.01887
27	oar3_OAR⟩	13466083	-0.00668
27	oar3_OAR⟩	13469874	0.00088
27	oar3_OAR⟩	13471586	-0.00668
27	oar3_OAR⟩	13476616	-0.00668
27	oar3_OAR⟩	13499685	-0.00668
27	oar3_OAR⟩	13514787	-0.00446
27	oar3_OAR⟩	13532612	-0.00606
27	oar3_OAR⟩	13540298	-0.00606
27	oar3_OAR⟩	13540547	0.040524
27	oar3_OAR⟩	13551558	-0.00606
27	oar3_OAR⟩	13558420	0.030858
27	oar3_OAR⟩	13560810	-0.00606
27	oar3_OAR⟩	13569208	0.030858
27	oar3_OAR⟩	13571359	0.030858
27	oar3_OAR⟩	13582156	-0.00606
27	oar3_OAR⟩	13582244	0.030858
27	oar3_OAR⟩	13582383	0.030858
27	oar3_OAR⟩	13590347	0.030858
27	oar3_OAR⟩	13592236	0.035666
27	oar3_OAR⟩	13604927	0.030858
27	oar3_OAR⟩	13614478	-0.01737
27	oar3_OAR⟩	13619091	-0.01699
27	oar3_OAR⟩	13621922	-0.01737
27	oar3_OAR⟩	13622976	0.109905
27	oar3_OAR⟩	13625120	-0.01699
27	oar3_OAR⟩	13649461	-0.02784

27	oar3_OAR›	13658492	-0.02784
27	oar3_OAR›	13658980	-0.02442
27	oar3_OAR›	13665681	-0.01834
27	oar3_OAR›	13669526	0.113139
27	oar3_OAR›	13672148	-0.02784
27	oar3_OAR›	13686618	0.033563
27	oar3_OAR›	13689333	-0.00927
27	oar3_OAR›	13696154	-0.02897
27	oar3_OAR›	13706500	-0.02897
27	oar3_OAR›	13719552	-0.01329
27	oar3_OAR›	13724615	0.025931
27	oar3_OAR›	13725349	0.019294
27	oar3_OAR›	13735400	0.019475
27	oar3_OAR›	13737108	0.019475
27	oar3_OAR›	13749831	-0.02531
27	oar3_OAR›	13750071	0.051643
27	oar3_OAR›	13756164	-0.00045
27	oar3_OAR›	13756853	0.024266
27	oar3_OAR›	13760541	0.068499
27	oar3_OAR›	13787580	0.068499
27	oar3_OAR›	13787767	0.015497
27	oar3_OAR›	13788219	0.051643
27	oar3_OAR›	13798968	0.068499
27	oar3_OAR›	13802787	0.051643
27	oar3_OAR›	13806042	0.037529
27	oar3_OAR›	13807753	0.051643
27	oar3_OAR›	13824101	0.029873
27	oar3_OAR›	13827129	NA
27	oar3_OAR›	13832047	NA
27	oar3_OAR›	13837360	0.029873
27	oar3_OAR›	13839195	0.031994
27	oar3_OAR›	13841711	0.031994
27	oar3_OAR›	13842498	0.047897
27	oar3_OAR›	13845970	0.031994
27	oar3_OAR›	13850398	0.038546
27	oar3_OAR›	13855341	0.036769
27	oar3_OAR›	13865328	0.040141
27	oar3_OAR›	13865406	0.014786
27	oar3_OAR›	13874509	0.026353
27	oar3_OAR›	13881686	-0.02184
27	oar3_OAR›	13884239	-0.03231
27	oar3_OAR›	13905443	-0.0385
27	oar3_OAR›	13913226	-0.02387
27	oar3_OAR›	13914053	0.013417
27	oar3_OAR›	13918009	-0.00884
27	oar3_OAR›	13920128	-0.02448
27	oar3_OAR›	13922901	-0.01735
27	oar3_OAR›	13926472	-0.02448
27	oar3_OAR›	13936831	-0.00998
27	oar3_OAR›	13939256	-0.00011
27	oar3_OAR›	13940089	0.001262
27	oar3_OAR›	13945627	-0.02807
27	oar3_OAR›	13945879	-0.02522
27	oar3_OAR›	13947284	-0.02834

27	oar3_OAR›	13955567	0.004532
27	oar3_OAR›	13957780	0.033804
27	oar3_OAR›	13959391	-0.03769
27	oar3_OAR›	13969582	-0.03769
27	oar3_OAR›	13974904	-0.01311
27	oar3_OAR›	13982844	-0.03769
27	oar3_OAR›	13984742	-0.03769
27	oar3_OAR›	13993195	-0.02683
27	oar3_OAR›	13998961	-0.03672
27	oar3_OAR›	14006135	0.002228
27	oar3_OAR›	14008323	-0.01423
27	oar3_OAR›	14014791	-0.02369
27	oar3_OAR›	14029397	-0.02902
27	oar3_OAR›	14040409	-0.0319
27	oar3_OAR›	14045890	-0.00865
27	oar3_OAR›	14048240	0.034101
27	oar3_OAR›	14059535	0.003425
27	oar3_OAR›	14063495	0.011389
27	oar3_OAR›	14070526	0.058353
27	oar3_OAR›	14076238	0.079324
27	oar3_OAR›	14077530	0.082873
27	oar3_OAR›	14082751	-0.02471
27	oar3_OAR›	14086867	0.037451
27	oar3_OAR›	14096780	-0.02471
27	oar3_OAR›	14097879	0.053574
27	oar3_OAR›	14099217	-0.0308
27	oar3_OAR›	14108618	NA
27	oar3_OAR›	14110169	-0.02531
27	oar3_OAR›	14110434	-0.00425
27	oar3_OAR›	14118665	0.007511
27	oar3_OAR›	14119029	0.007511
27	oar3_OAR›	14120293	0.00481
27	oar3_OAR›	14126002	0.019346
27	oar3_OAR›	14137609	0.085177
27	oar3_OAR›	14137994	0.05528
27	oar3_OAR›	14147567	-0.00267
27	oar3_OAR›	14148164	0.121655
27	oar3_OAR›	14150071	0.007039
27	oar3_OAR›	14155496	0.005915
27	oar3_OAR›	14157631	-0.00441
27	oar3_OAR›	14161208	0.044724
27	oar3_OAR›	14165388	-0.02597
27	oar3_OAR›	14167351	-0.02597
27	oar3_OAR›	14167676	-0.02597
27	oar3_OAR›	14174459	0.011067
27	oar3_OAR›	14183401	-0.01702
27	oar3_OAR›	14183957	0.155149
27	oar3_OAR›	14184268	-0.01702
27	oar3_OAR›	14189235	-0.00037
27	oar3_OAR›	14194705	0.200353
27	oar3_OAR›	14195844	0.080014
27	oar3_OAR›	14197439	0.076683
27	oar3_OAR›	14204398	0.018106
27	oar3_OAR›	14213283	-0.03024

27	oar3_OAR›	14239040	-0.00957
27	oar3_OAR›	14240566	-0.02567
27	oar3_OAR›	14243609	-0.02325
27	oar3_OAR›	14252972	-0.01084
27	oar3_OAR›	14252994	0.007996
27	oar3_OAR›	14261483	-0.00961
27	oar3_OAR›	14267388	-0.01657
27	oar3_OAR›	14270315	-0.01964
27	oar3_OAR›	14274666	-0.02174
27	oar3_OAR›	14277340	-0.02082
27	oar3_OAR›	14282511	-0.00739
27	oar3_OAR›	14285213	-0.0179
27	oar3_OAR›	14289425	-0.03432
27	oar3_OAR›	14295370	0.041202
27	oar3_OAR›	14299768	0.052782
27	oar3_OAR›	14302707	0.081786
27	oar3_OAR›	14304784	0.073272
27	oar3_OAR›	14308571	0.044077
27	oar3_OAR›	14310323	0.037425
27	oar3_OAR›	14315507	0.182074
27	oar3_OAR›	14323371	0.074675
27	oar3_OAR›	14327707	-0.01989
27	oar3_OAR›	14336455	-0.01989
27	oar3_OAR›	14340539	-0.01141
27	oar3_OAR›	14353530	0.069893
27	oar3_OAR›	14362877	0.105104
27	oar3_OAR›	14363353	0.037433
27	oar3_OAR›	14364319	0.060324
27	oar3_OAR›	14372900	0.074839
27	oar3_OAR›	14381009	0.074839
27	oar3_OAR›	14382781	0.023859
27	oar3_OAR›	14384151	-0.02104
27	oar3_OAR›	14407690	0.007178
27	oar3_OAR›	14411803	0.029554
27	oar3_OAR›	14412539	0.029554
27	oar3_OAR›	14414520	-0.03086
27	oar3_OAR›	14414866	0.011067
27	oar3_OAR›	14426321	0.08299
27	oar3_OAR›	14427802	0.088071
27	oar3_OAR›	14439590	0.032812
27	oar3_OAR›	14442639	0.024156
27	oar3_OAR›	14453360	0.012492
27	oar3_OAR›	14453987	0.036506
27	oar3_OAR›	14455200	0.107673
27	oar3_OAR›	14464373	0.10028
27	oar3_OAR›	14464666	0.10028
27	oar3_OAR›	14465950	0.108575
27	oar3_OAR›	14466109	0.139234
27	oar3_OAR›	14475943	0.10028
27	oar3_OAR›	14476030	0.10028
27	oar3_OAR›	14478462	0.10028
27	oar3_OAR›	14482016	0.10028
27	oar3_OAR›	14488514	0.10028
27	oar3_OAR›	14489253	-0.00308

27	oar3_OAR〉	14489264	0.116589
27	oar3_OAR〉	14500294	0.134201
27	oar3_OAR〉	14502192	0.058388
27	oar3_OAR〉	14503194	0.025688
27	oar3_OAR〉	14512312	0.119861
27	oar3_OAR〉	14513085	0.119861
27	oar3_OAR〉	14515734	0.119861
27	oar3_OAR〉	14516095	0.028688
27	oar3_OAR〉	14517722	0.005851
27	oar3_OAR〉	14523263	0.10648
27	oar3_OAR〉	14523646	0.203545
27	oar3_OAR〉	14529824	0.151331
27	oar3_OAR〉	14546699	0.0082
27	oar3_OAR〉	14546882	0.003678
27	oar3_OAR〉	14547409	0.07085
27	oar3_OAR〉	14549054	0.07085
27	oar3_OAR〉	14554255	-0.01665
27	oar3_OAR〉	14556866	0.091911
27	oar3_OAR〉	14559081	0.165959
27	oar3_OAR〉	14559528	0.165959
27	oar3_OAR〉	14559980	0.091911
27	oar3_OAR〉	14564885	0.053574
27	oar3_OAR〉	14566180	0.070004
27	oar3_OAR〉	14567240	-0.02681
27	oar3_OAR〉	14569255	-0.00121
27	oar3_OAR〉	14579893	0.053115
27	oar3_OAR〉	14580184	0.053115
27	oar3_OAR〉	14597993	0.000945
27	oar3_OAR〉	14601402	-0.02031
27	oar3_OAR〉	14602248	-0.01911
27	oar3_OAR〉	14615357	-0.00871
27	oar3_OAR〉	14621139	0.000945
27	oar3_OAR〉	14621329	0.000945
27	oar3_OAR〉	14623777	-0.01214
27	oar3_OAR〉	14626783	0.051303
27	oar3_OAR〉	14631964	-0.02164
27	oar3_OAR〉	14632273	0.000945
27	oar3_OAR〉	14633506	0.051303
27	oar3_OAR〉	14637434	0.040006
27	oar3_OAR〉	14657612	0.115081
27	oar3_OAR〉	14661558	-0.02164
27	oar3_OAR〉	14662129	-0.0298
27	oar3_OAR〉	14663575	0.000945
27	oar3_OAR〉	14666179	0.010604
27	oar3_OAR〉	14671787	-0.00016
27	oar3_OAR〉	14674195	0.021937
27	oar3_OAR〉	14676443	0.041849
27	oar3_OAR〉	14676903	0.014122
27	oar3_OAR〉	14686855	0.092546
27	oar3_OAR〉	14686912	-0.01317
27	oar3_OAR〉	14690144	-0.01317
27	oar3_OAR〉	14693495	0.045503
27	oar3_OAR〉	14698829	0.010026
27	oar3_OAR〉	14704508	0.046264

27	oar3_OAR〉	14706317	0.020686
27	oar3_OAR〉	14711313	0.13268
27	oar3_OAR〉	14712009	0.13268
27	oar3_OAR〉	14717034	0.092685
27	oar3_OAR〉	14718848	0.080156
27	oar3_OAR〉	14721758	0.109761
27	oar3_OAR〉	14727836	0.019587
27	oar3_OAR〉	14729313	0.140853
27	oar3_OAR〉	14736934	0.010883
27	oar3_OAR〉	14737313	-0.01384
27	oar3_OAR〉	14742995	0.016176
27	oar3_OAR〉	14743509	-0.01567
27	oar3_OAR〉	14756208	-0.01567
27	oar3_OAR〉	14757112	0.000214
27	oar3_OAR〉	14759760	0.009985
27	oar3_OAR〉	14759917	-0.02744
27	oar3_OAR〉	14761237	0.05761
27	oar3_OAR〉	14768899	0.05761
27	oar3_OAR〉	14772141	0.040674
27	oar3_OAR〉	14774198	-0.04082
27	oar3_OAR〉	14774521	0.147214
27	oar3_OAR〉	14778646	0.043657
27	oar3_OAR〉	14782707	0.191185
27	oar3_OAR〉	14783487	0.015055
27	oar3_OAR〉	14784560	-0.00382
27	oar3_OAR〉	14788684	0.084485
27	oar3_OAR〉	14796612	-0.02119
27	oar3_OAR〉	14797014	-0.02586
27	oar3_OAR〉	14797762	-0.02159
27	oar3_OAR〉	14798275	-0.02159
27	oar3_OAR〉	14802683	-0.03869
27	oar3_OAR〉	14806331	-0.02088
27	oar3_OAR〉	14809377	-0.02159
27	oar3_OAR〉	14814523	-0.03869
27	oar3_OAR〉	14820118	-0.02562
27	oar3_OAR〉	14823466	-0.02159
27	oar3_OAR〉	14825706	0.105061
27	oar3_OAR〉	14826129	0.003054
27	oar3_OAR〉	14831100	0.079324
27	oar3_OAR〉	14842211	6.15E-05
27	oar3_OAR〉	14842330	0.006016
27	oar3_OAR〉	14851407	0.154482
27	oar3_OAR〉	14859357	0.037636
27	oar3_OAR〉	14859721	0.016499
27	oar3_OAR〉	14871944	-0.00335
27	oar3_OAR〉	14872226	0.013369
27	oar3_OAR〉	14878415	0.026159
27	oar3_OAR〉	14887522	0.014568
27	oar3_OAR〉	14889750	-0.01824
27	oar3_OAR〉	14897523	-0.02785
27	oar3_OAR〉	14902903	-0.01824
27	oar3_OAR〉	14907227	-0.02137
27	oar3_OAR〉	14914377	-0.0302
27	oar3_OAR〉	14917835	-0.00622

27	oar3_OAR›	14917911	-0.0059
27	oar3_OAR›	14919747	-0.00109
27	oar3_OAR›	14927755	-0.01962
27	oar3_OAR›	14930094	-0.02704
27	oar3_OAR›	14930160	-0.02704
27	oar3_OAR›	14934376	0.02438
27	oar3_OAR›	14938109	0.025097
27	oar3_OAR›	14939962	########
27	oar3_OAR›	14940346	-0.00209
27	oar3_OAR›	14946722	-0.03463
27	oar3_OAR›	14949128	-0.01086
27	oar3_OAR›	14953938	-0.00576
27	oar3_OAR›	14961227	0.085437
27	oar3_OAR›	14961627	-0.01017
27	oar3_OAR›	14967258	0.210045
27	oar3_OAR›	14969323	-0.00535
27	oar3_OAR›	14978897	0.047776
27	oar3_OAR›	14980392	0.025097
27	oar3_OAR›	14982498	-0.04011
27	oar3_OAR›	14987353	-0.01913
27	oar3_OAR›	14988717	-0.02512
27	oar3_OAR›	14990741	0.032095
27	oar3_OAR›	14994087	0.05945
27	oar3_OAR›	14999180	0.153538
27	oar3_OAR›	15000557	0.017067
27	oar3_OAR›	15001590	-0.02286
27	oar3_OAR›	15007942	-0.03835
27	oar3_OAR›	15008393	0.010842
27	oar3_OAR›	15008493	-0.01724
27	oar3_OAR›	15020566	-0.01297
27	oar3_OAR›	15030457	-0.02642
27	oar3_OAR›	15046331	-0.0178
27	oar3_OAR›	15047500	0.052203
27	oar3_OAR›	15051079	0.031311
27	oar3_OAR›	15056950	-0.03058
27	oar3_OAR›	15058563	0.087241
27	oar3_OAR›	15068629	-0.00169
27	oar3_OAR›	15072958	-0.03461
27	oar3_OAR›	15078618	0.000889
27	oar3_OAR›	15081857	0.003164
27	oar3_OAR›	15089563	0.070714
27	oar3_OAR›	15102380	0.093614
27	oar3_OAR›	15102959	0.093614
27	oar3_OAR›	15120352	0.094947
27	oar3_OAR›	15132664	0.115932
27	oar3_OAR›	15136610	0.142485
27	oar3_OAR›	15143285	-0.02926
27	oar3_OAR›	15148243	0.196033
27	oar3_OAR›	15148316	0.163377
27	oar3_OAR›	15158159	-0.0155
27	oar3_OAR›	15158550	-0.0155
27	oar3_OAR›	15159762	-0.0155
27	oar3_OAR›	15160671	0.061943
27	oar3_OAR›	15176560	0.042125

27	oar3_OAR›	15176854	0.042125
27	oar3_OAR›	15177626	0.042125
27	oar3_OAR›	15180958	-0.00357
27	oar3_OAR›	15187922	-0.0293
27	oar3_OAR›	15191337	-0.01423
27	oar3_OAR›	15197659	0.125094
27	oar3_OAR›	15198122	0.125094
27	oar3_OAR›	15200488	-0.00606
27	oar3_OAR›	15203838	0.008723
27	oar3_OAR›	15204833	0.057981
27	oar3_OAR›	15215464	-0.00928
27	oar3_OAR›	15222271	0.008723
27	oar3_OAR›	15231690	-0.01243
27	oar3_OAR›	15233580	-0.00758
27	oar3_OAR›	15244557	0.259926
27	oar3_OAR›	15256173	0.121732
27	oar3_OAR›	15256654	0.121732
27	oar3_OAR›	15257259	0.121732
27	oar3_OAR›	15260430	0.077313
27	oar3_OAR›	15276459	0.024879
27	oar3_OAR›	15282564	0.332133
27	oar3_OAR›	15283590	0.032043
27	oar3_OAR›	15288666	0.032043
27	oar3_OAR›	15295874	0.339111
27	oar3_OAR›	15296919	0.051241
27	oar3_OAR›	15297187	0.313201
27	oar3_OAR›	15308192	0.339111
27	oar3_OAR›	15309960	0.302015
27	oar3_OAR›	15310590	0.017742
27	oar3_OAR›	15316124	0.10301
27	oar3_OAR›	15317844	-0.03869
27	oar3_OAR›	15322927	0.193128
27	oar3_OAR›	15328343	-0.00901
27	oar3_OAR›	15338596	0.134494
27	oar3_OAR›	15353907	0.007854
27	oar3_OAR›	15354224	0.071497
27	oar3_OAR›	15356020	0.026075
27	oar3_OAR›	15368156	0.071497
27	oar3_OAR›	15369038	0.115543
27	oar3_OAR›	15369964	0.011815
27	oar3_OAR›	15380794	0.046767
27	oar3_OAR›	15392887	0.115543
27	oar3_OAR›	15393199	0.046767
27	oar3_OAR›	15393856	0.0075
27	oar3_OAR›	15398547	0.115543
27	oar3_OAR›	15401601	0.025277
27	oar3_OAR›	15403501	0.003394
27	oar3_OAR›	15404258	-0.02057
27	oar3_OAR›	15412279	0.052599
27	oar3_OAR›	15413940	0.003394
27	oar3_OAR›	15422475	0.115543
27	oar3_OAR›	15434213	0.009117
27	oar3_OAR›	15434271	0.009117
27	oar3_OAR›	15440195	0.138351

27	oar3_OAR›	15442970	0.128852
27	oar3_OAR›	15447450	0.05319
27	oar3_OAR›	15452712	0.043609
27	oar3_OAR›	15458451	0.106701
27	oar3_OAR›	15460539	0.339136
27	oar3_OAR›	15463486	0.092167
27	oar3_OAR›	15463831	0.011067
27	oar3_OAR›	15464764	0.113381
27	oar3_OAR›	15471841	0.110922
27	oar3_OAR›	15477289	0.013849
27	oar3_OAR›	15477918	0.079336
27	oar3_OAR›	15478410	0.079336
27	oar3_OAR›	15493352	0.058095
27	oar3_OAR›	15493546	0.125023
27	oar3_OAR›	15498950	0.10911
27	oar3_OAR›	15499055	0.139981
27	oar3_OAR›	15508592	0.327819
27	oar3_OAR›	15510634	0.216174
27	oar3_OAR›	15511935	0.327819
27	oar3_OAR›	15512783	0.327819
27	oar3_OAR›	15517791	0.115766
27	oar3_OAR›	15523165	0.000889
27	oar3_OAR›	15523592	0.180723
27	oar3_OAR›	15526987	0.113572
27	oar3_OAR›	15528119	0.037433
27	oar3_OAR›	15541685	-0.02822
27	oar3_OAR›	15570089	0.073272
27	oar3_OAR›	15574803	0.187581
27	oar3_OAR›	15575998	-0.02822
27	oar3_OAR›	15576858	0.186974
27	oar3_OAR›	15582196	0.227046
27	oar3_OAR›	15582298	-0.02683
27	oar3_OAR›	15587353	0.18469
27	oar3_OAR›	15590048	0.16512
27	oar3_OAR›	15592278	0.18469
27	oar3_OAR›	15598444	0.178165
27	oar3_OAR›	15603342	0.260555
27	oar3_OAR›	15603928	0.203525
27	oar3_OAR›	15605148	0.123857
27	oar3_OAR›	15611956	0.000893
27	oar3_OAR›	15612036	0.014754
27	oar3_OAR›	15617627	0.16512
27	oar3_OAR›	15623732	0.149072
27	oar3_OAR›	15625094	0.127493
27	oar3_OAR›	15628783	0.025008
27	oar3_OAR›	15628894	0.136506
27	oar3_OAR›	15630194	0.149828
27	oar3_OAR›	15634920	0.12742
27	oar3_OAR›	15643872	0.133549
27	oar3_OAR›	15647138	0.108193
27	oar3_OAR›	15654451	0.333145
27	oar3_OAR›	15661618	0.304949
27	oar3_OAR›	15665950	0.282823
27	oar3_OAR›	15668082	0.133549

27	oar3_OAR⟩	15678818	0.304949
27	oar3_OAR⟩	15683285	0.097713
27	oar3_OAR⟩	15684974	0.004924
27	oar3_OAR⟩	15688101	-0.01618
27	oar3_OAR⟩	15693822	0.020399
27	oar3_OAR⟩	15694258	0.363249
27	oar3_OAR⟩	15715491	0.130458
27	oar3_OAR⟩	15728432	-0.01633
27	oar3_OAR⟩	15773264	0.103749
27	oar3_OAR⟩	15796046	0.103749
27	oar3_OAR⟩	15809631	0.103749
27	oar3_OAR⟩	15819522	0.012138
27	oar3_OAR⟩	15836633	-0.0038
27	oar3_OAR⟩	15838934	0.012138
27	oar3_OAR⟩	15840100	-0.0038
27	oar3_OAR⟩	15844315	0.148874
27	oar3_OAR⟩	15846045	-0.0038
27	oar3_OAR⟩	15856421	-0.0038
27	oar3_OAR⟩	15871152	-0.02688
27	oar3_OAR⟩	15876593	0.103749
27	oar3_OAR⟩	15886920	0.075175
27	oar3_OAR⟩	15888363	-0.03152
27	oar3_OAR⟩	15892192	-0.03152
27	oar3_OAR⟩	15899821	0.041601
27	oar3_OAR⟩	15901983	-0.01141
27	oar3_OAR⟩	15906651	0.073272
27	oar3_OAR⟩	15907637	-0.01141
27	oar3_OAR⟩	15910391	0.093506
27	oar3_OAR⟩	15912866	0.052226
27	oar3_OAR⟩	15920294	0.061799
27	oar3_OAR⟩	15926018	0.034457
27	oar3_OAR⟩	15931511	0.052226
27	oar3_OAR⟩	15938436	0.052226
27	oar3_OAR⟩	15940276	-0.00765
27	oar3_OAR⟩	15942063	0.06591
27	oar3_OAR⟩	15949174	0.136358
27	oar3_OAR⟩	15958214	0.108203
27	oar3_OAR⟩	15958556	0.136358
27	oar3_OAR⟩	15963179	0.052226
27	oar3_OAR⟩	15964005	0.004036
27	oar3_OAR⟩	15964517	0.136358
27	oar3_OAR⟩	15970290	0.052226
27	oar3_OAR⟩	15971236	0.11361
27	oar3_OAR⟩	15981426	0.09292
27	oar3_OAR⟩	16023123	0.09292
27	oar3_OAR⟩	16023244	0.052226
27	oar3_OAR⟩	16035818	0.052226
27	oar3_OAR⟩	16043359	0.075175
27	oar3_OAR⟩	16044547	-0.026
27	oar3_OAR⟩	16061726	0.019459
27	oar3_OAR⟩	16066726	0.002868
27	oar3_OAR⟩	16076941	0.047646
27	oar3_OAR⟩	16081662	0.047646
27	oar3_OAR⟩	16084687	0.025831

27	oar3_OAR›	16087682	0.002868
27	oar3_OAR›	16090599	-0.00032
27	oar3_OAR›	16098127	0.030008
27	oar3_OAR›	16104539	0.002868
27	oar3_OAR›	16104608	0.022266
27	oar3_OAR›	16107431	0.041488
27	oar3_OAR›	16112315	0.070714
27	oar3_OAR›	16120270	0.002868
27	oar3_OAR›	16126694	0.041532
27	oar3_OAR›	16134014	0.164417
27	oar3_OAR›	16140371	0.129378
27	oar3_OAR›	16143208	0.11726
27	oar3_OAR›	16147516	0.052562
27	oar3_OAR›	16153735	0.138483
27	oar3_OAR›	16165283	0.078248
27	oar3_OAR›	16169579	0.156894
27	oar3_OAR›	16179110	0.190037
27	oar3_OAR›	16180149	0.081793
27	oar3_OAR›	16191247	0.028702
27	oar3_OAR›	16195598	-0.0125
27	oar3_OAR›	16195679	-0.02926
27	oar3_OAR›	16195849	-0.0125
27	oar3_OAR›	16197410	-0.02168
27	oar3_OAR›	16205021	0.041658
27	oar3_OAR›	16205722	-0.01443
27	oar3_OAR›	16208083	0.023929
27	oar3_OAR›	16212303	0.015985
27	oar3_OAR›	16217703	0.027422
27	oar3_OAR›	16225875	-0.0085
27	oar3_OAR›	16239741	0.023793
27	oar3_OAR›	16239981	0.083142
27	oar3_OAR›	16241462	0.038453
27	oar3_OAR›	16243402	-0.02565
27	oar3_OAR›	16245973	-0.02897
27	oar3_OAR›	16252531	0.13159
27	oar3_OAR›	16257769	0.13159
27	oar3_OAR›	16265721	0.13159
27	oar3_OAR›	16273924	0.093195
27	oar3_OAR›	16274131	0.104279
27	oar3_OAR›	16274888	0.04394
27	oar3_OAR›	16291252	0.042977
27	oar3_OAR›	16292507	0.022444
27	oar3_OAR›	16293618	0.039198
27	oar3_OAR›	16299956	0.039198
27	oar3_OAR›	16301159	0.104279
27	oar3_OAR›	16313504	-0.03239
27	oar3_OAR›	16325115	0.025713
27	oar3_OAR›	16329971	0.023091
27	oar3_OAR›	16335116	0.015047
27	oar3_OAR›	16348323	-0.01017
27	oar3_OAR›	16348873	0.016807
27	oar3_OAR›	16373720	-0.00935
27	oar3_OAR›	16380252	0.054621
27	oar3_OAR›	16385512	0.108112

27	oar3_OAR>	16389066	0.127395
27	oar3_OAR>	16406568	0.127395
27	oar3_OAR>	16410379	0.084722
27	oar3_OAR>	16411739	0.055667
27	oar3_OAR>	16422449	0.095259
27	oar3_OAR>	16422646	0.022668
27	oar3_OAR>	16436028	-0.00194
27	oar3_OAR>	16437602	-0.0225
27	oar3_OAR>	16439267	-0.03163
27	oar3_OAR>	16454843	-0.00274
27	oar3_OAR>	16455624	0.062492
27	oar3_OAR>	16463357	0.013225
27	oar3_OAR>	16465999	-0.01704
27	oar3_OAR>	16470905	-0.01971
27	oar3_OAR>	16473205	-0.01132
27	oar3_OAR>	16479094	-0.03185
27	oar3_OAR>	16481408	0.009313
27	oar3_OAR>	16481608	-0.02819
27	oar3_OAR>	16483807	0.039481
27	oar3_OAR>	16490704	0.046507
27	oar3_OAR>	16492929	-0.0125
27	oar3_OAR>	16499417	-0.02413
27	oar3_OAR>	16510006	-0.02381
27	oar3_OAR>	16514977	0.066932
27	oar3_OAR>	16520652	0.038795
27	oar3_OAR>	16527243	0.004976
27	oar3_OAR>	16530812	0.038795
27	oar3_OAR>	16531018	0.024211
27	oar3_OAR>	16547351	0.081777
27	oar3_OAR>	16562065	0.064765
27	oar3_OAR>	16562536	0.019638
27	oar3_OAR>	16562566	0.064765
27	oar3_OAR>	16564716	0.056669
27	oar3_OAR>	16575196	0.064244
27	oar3_OAR>	16580780	-0.01336
27	oar3_OAR>	16582241	-0.02156
27	oar3_OAR>	16588239	0.021381
27	oar3_OAR>	16588640	-0.01177
27	oar3_OAR>	16600945	-0.03217
27	oar3_OAR>	16601126	0.019091
27	oar3_OAR>	16613638	0.040778
27	oar3_OAR>	16634824	0.030185
27	oar3_OAR>	16651985	0.00651
27	oar3_OAR>	16703289	0.022432
27	oar3_OAR>	16703417	0.034348
27	oar3_OAR>	16736389	0.01024
27	oar3_OAR>	16737994	0.010803
27	oar3_OAR>	16745680	-0.01756
27	oar3_OAR>	16747745	0.029879
27	oar3_OAR>	16750774	-0.02049
27	oar3_OAR>	16753011	0.064242
27	oar3_OAR>	16766632	-0.02662
27	oar3_OAR>	16769332	-0.02662
27	oar3_OAR>	16770804	0.052035

27	oar3_OAR›	16775306	-0.02662
27	oar3_OAR›	16783146	-0.02662
27	oar3_OAR›	16784272	-0.02662
27	oar3_OAR›	16786620	0.058702
27	oar3_OAR›	16811261	0.005972
27	oar3_OAR›	16811859	-0.00935
27	oar3_OAR›	16811965	-0.00935
27	oar3_OAR›	16817034	0.005972
27	oar3_OAR›	16829572	-0.0283
27	oar3_OAR›	16829814	-0.01415
27	oar3_OAR›	16830784	0.127467
27	oar3_OAR›	16835359	-0.00067
27	oar3_OAR›	16843021	0.175273
27	oar3_OAR›	16846735	0.175273
27	oar3_OAR›	16847914	0.014864
27	oar3_OAR›	16860220	-0.03024
27	oar3_OAR›	16860322	0.106909
27	oar3_OAR›	16864993	-0.01879
27	oar3_OAR›	16866805	-0.00485
27	oar3_OAR›	16875341	-0.03024
27	oar3_OAR›	16876445	-0.03369
27	oar3_OAR›	16876771	-0.04078
27	oar3_OAR›	16882722	-0.01069
27	oar3_OAR›	16883702	0.144402
27	oar3_OAR›	16887463	-0.02232
27	oar3_OAR›	16898920	-0.00261
27	oar3_OAR›	16902289	-0.02004
27	oar3_OAR›	16905146	0.000707
27	oar3_OAR›	16912129	0.228836
27	oar3_OAR›	16912284	0.044356
27	oar3_OAR›	16912417	0.179657
27	oar3_OAR›	16918654	0.068389
27	oar3_OAR›	16929990	0.04091
27	oar3_OAR›	16935481	0.024422
27	oar3_OAR›	16935550	0.066175
27	oar3_OAR›	16936076	0.024422
27	oar3_OAR›	16939981	0.013321
27	oar3_OAR›	16944878	0.032582
27	oar3_OAR›	16946353	0.002113
27	oar3_OAR›	16952758	0.032582
27	oar3_OAR›	16952941	0.113553
27	oar3_OAR›	16961957	-0.00335
27	oar3_OAR›	16964613	0.060162
27	oar3_OAR›	16965561	0.032312
27	oar3_OAR›	16973429	0.163734
27	oar3_OAR›	16976311	0.127885
27	oar3_OAR›	16977061	0.226287
27	oar3_OAR›	16983175	0.034967
27	oar3_OAR›	16989128	0.109894
27	oar3_OAR›	16994296	0.160666
27	oar3_OAR›	16994714	0.160666
27	oar3_OAR›	16994894	-0.02988
27	oar3_OAR›	16999782	1.82E-05
27	oar3_OAR›	17003427	0.018804

27	oar3_OAR〉	17010836	0.05565
27	oar3_OAR〉	17016207	-0.00512
27	oar3_OAR〉	17016298	-0.01333
27	oar3_OAR〉	17017495	0.07132
27	oar3_OAR〉	17018774	0.07132
27	oar3_OAR〉	17021048	0.07132
27	oar3_OAR〉	17024305	0.018804
27	oar3_OAR〉	17029340	0.090399
27	oar3_OAR〉	17030229	0.161215
27	oar3_OAR〉	17031917	0.030028
27	oar3_OAR〉	17035686	-0.01252
27	oar3_OAR〉	17039312	-0.011
27	oar3_OAR〉	17040041	0.077799
27	oar3_OAR〉	17041620	0.044847
27	oar3_OAR〉	17046791	0.080088
27	oar3_OAR〉	17049559	-0.01652
27	oar3_OAR〉	17056358	0.023707
27	oar3_OAR〉	17057118	0.059985
27	oar3_OAR〉	17057354	0.06516
27	oar3_OAR〉	17059382	0.009985
27	oar3_OAR〉	17064380	-0.00555
27	oar3_OAR〉	17070841	-0.0197
27	oar3_OAR〉	17080036	0.038185
27	oar3_OAR〉	17092845	0.063401
27	oar3_OAR〉	17105886	-0.01879
27	oar3_OAR〉	17105950	0.052265
27	oar3_OAR〉	17107402	0.045114
27	oar3_OAR〉	17107655	0.145226
27	oar3_OAR〉	17111255	-0.01595
27	oar3_OAR〉	17126172	-0.02652
27	oar3_OAR〉	17126578	-0.02087
27	oar3_OAR〉	17131979	0.021766
27	oar3_OAR〉	17133178	-0.00314
27	oar3_OAR〉	17136218	-0.00066
27	oar3_OAR〉	17138238	-0.00314
27	oar3_OAR〉	17141480	0.030243
27	oar3_OAR〉	17145113	0.00372
27	oar3_OAR〉	17147436	-0.00446
27	oar3_OAR〉	17147635	-0.03085
27	oar3_OAR〉	17154904	-0.00606
27	oar3_OAR〉	17156266	-0.02487
27	oar3_OAR〉	17159838	-0.02576
27	oar3_OAR〉	17159903	0.035625
27	oar3_OAR〉	17166035	0.035625
27	oar3_OAR〉	17168736	0.054933
27	oar3_OAR〉	17171596	0.038838
27	oar3_OAR〉	17173671	-0.00409
27	oar3_OAR〉	17178410	0.007996
27	oar3_OAR〉	17180121	0.046852
27	oar3_OAR〉	17184160	0.003593
27	oar3_OAR〉	17192616	0.046852
27	oar3_OAR〉	17204337	-0.0019
27	oar3_OAR〉	17206833	0.046852
27	oar3_OAR〉	17211447	0.022368

27	oar3_OAR›	17211774	-0.01871
27	oar3_OAR›	17217139	0.093803
27	oar3_OAR›	17217458	0.087558
27	oar3_OAR›	17223920	0.093803
27	oar3_OAR›	17230592	0.032487
27	oar3_OAR›	17231516	0.087558
27	oar3_OAR›	17235865	0.027125
27	oar3_OAR›	17241147	0.028803
27	oar3_OAR›	17245122	-0.01275
27	oar3_OAR›	17246367	-0.01275
27	oar3_OAR›	17252460	0.008723
27	oar3_OAR›	17253755	0.008723
27	oar3_OAR›	17254039	0.029928
27	oar3_OAR›	17258912	0.008723
27	oar3_OAR›	17264209	-0.01008
27	oar3_OAR›	17266127	0.008296
27	oar3_OAR›	17266855	-0.00956
27	oar3_OAR›	17267332	-0.00956
27	oar3_OAR›	17270958	0.0049
27	oar3_OAR›	17281785	0.022514
27	oar3_OAR›	17284196	-0.02555
27	oar3_OAR›	17284896	-0.03062
27	oar3_OAR›	17302157	0.040698
27	oar3_OAR›	17304848	-0.01186
27	oar3_OAR›	17305826	-0.01186
27	oar3_OAR›	17307205	0.033491
27	oar3_OAR›	17315221	NA
27	oar3_OAR›	17315598	0.112209
27	oar3_OAR›	17320800	0.010712
27	oar3_OAR›	17323432	-0.01628
27	oar3_OAR›	17326431	-0.00735
27	oar3_OAR›	17331425	-0.01939
27	oar3_OAR›	17331750	-0.00056
27	oar3_OAR›	17334875	-0.03768
27	oar3_OAR›	17345047	0.104672
27	oar3_OAR›	17345158	0.151885
27	oar3_OAR›	17364788	0.03617
27	oar3_OAR›	17376517	0.079258
27	oar3_OAR›	17382365	-0.00791
27	oar3_OAR›	17389277	0.079258
27	oar3_OAR›	17389526	0.079258
27	oar3_OAR›	17397739	0.03617
27	oar3_OAR›	17412033	-0.01995
27	oar3_OAR›	17420573	0.03688
27	oar3_OAR›	17423152	0.107333
27	oar3_OAR›	17430659	0.10191
27	oar3_OAR›	17442723	0.034602
27	oar3_OAR›	17447458	0.039466
27	oar3_OAR›	17449524	0.008681
27	oar3_OAR›	17449843	0.03958
27	oar3_OAR›	17452775	-0.00909
27	oar3_OAR›	17463185	0.022867
27	oar3_OAR›	17466241	0.02221
27	oar3_OAR›	17467426	-0.02209

27	oar3_OAR>	17468333	0.02221
27	oar3_OAR>	17468550	0.023492
27	oar3_OAR>	17474862	0.023492
27	oar3_OAR>	17477081	-0.00353
27	oar3_OAR>	17480083	0.02221
27	oar3_OAR>	17484736	0.023492
27	oar3_OAR>	17486519	0.037735
27	oar3_OAR>	17489942	-0.02174
27	oar3_OAR>	17491285	-0.01875
27	oar3_OAR>	17492114	-0.01511
27	oar3_OAR>	17498632	-0.01234
27	oar3_OAR>	17506302	-0.02772
27	oar3_OAR>	17506693	-0.00952
27	oar3_OAR>	17509061	0.007039
27	oar3_OAR>	17510506	-0.01686
27	oar3_OAR>	17516258	-0.0177
27	oar3_OAR>	17520972	-0.02438
27	oar3_OAR>	17521225	-0.0177
27	oar3_OAR>	17529688	0.02374
27	oar3_OAR>	17535450	0.02374
27	oar3_OAR>	17537653	0.167035
27	oar3_OAR>	17544049	0.181357
27	oar3_OAR>	17549589	0.152574
27	oar3_OAR>	17551680	-0.00923
27	oar3_OAR>	17552879	0.117147
27	oar3_OAR>	17554723	0.095871
27	oar3_OAR>	17560996	0.095663
27	oar3_OAR>	17561428	0.095663
27	oar3_OAR>	17567770	-0.01008
27	oar3_OAR>	17571522	-0.0302
27	oar3_OAR>	17573822	-0.00735
27	oar3_OAR>	17576965	0.04198
27	oar3_OAR>	17579000	0.04198
27	oar3_OAR>	17583562	0.030434
27	oar3_OAR>	17587371	0.119364
27	oar3_OAR>	17590344	0.119364
27	oar3_OAR>	17593307	0.119364
27	oar3_OAR>	17599199	0.119364
27	oar3_OAR>	17600185	0.153362
27	oar3_OAR>	17606062	0.18658
27	oar3_OAR>	17612310	0.18658
27	oar3_OAR>	17623037	0.119364
27	oar3_OAR>	17631272	0.145825
27	oar3_OAR>	17646227	0.046782
27	oar3_OAR>	17653780	0.046782
27	oar3_OAR>	17658969	0.046782
27	oar3_OAR>	17659374	-0.00942
27	oar3_OAR>	17662873	-0.00765
27	oar3_OAR>	17663916	0.024935
27	oar3_OAR>	17671127	-0.02205
27	oar3_OAR>	17673491	-0.02556
27	oar3_OAR>	17678504	0.060992
27	oar3_OAR>	17686500	-0.02648
27	oar3_OAR>	17698018	0.029289

27	oar3_OAR›	17705027	0.025223
27	oar3_OAR›	17708182	-0.02487
27	oar3_OAR›	17720496	0.024368
27	oar3_OAR›	17720763	-0.02947
27	oar3_OAR›	17720981	-0.02947
27	oar3_OAR›	17725539	-0.00999
27	oar3_OAR›	17725648	-0.00428
27	oar3_OAR›	17732015	0.024368
27	oar3_OAR›	17733418	0.059429
27	oar3_OAR›	17735956	-0.00428
27	oar3_OAR›	17741193	-0.00999
27	oar3_OAR›	17745983	-0.00428
27	oar3_OAR›	17749036	0.059429
27	oar3_OAR›	17749204	0.059429
27	oar3_OAR›	17760474	-0.02492
27	oar3_OAR›	17762296	0.019555
27	oar3_OAR›	17772822	0.019555
27	oar3_OAR›	17776367	0.019555
27	oar3_OAR›	17782212	-0.02492
27	oar3_OAR›	17793263	-0.03231
27	oar3_OAR›	17796075	0.00295
27	oar3_OAR›	17802880	-0.0278
27	oar3_OAR›	17810538	-0.00669
27	oar3_OAR›	17810808	-0.00955
27	oar3_OAR›	17812495	-0.03113
27	oar3_OAR›	17821131	-0.00406
27	oar3_OAR›	17822026	-0.03458
27	oar3_OAR›	17822496	-0.03231
27	oar3_OAR›	17826723	0.003054
27	oar3_OAR›	17832692	-0.00275
27	oar3_OAR›	17850698	-0.01416
27	oar3_OAR›	17851532	-0.00861
27	oar3_OAR›	17856500	0.051958
27	oar3_OAR›	17861860	-0.00861
27	oar3_OAR›	17865401	-0.01676
27	oar3_OAR›	17865781	-0.00406
27	oar3_OAR›	17867487	0.005676
27	oar3_OAR›	17873489	-0.0293
27	oar3_OAR›	17878702	-0.00406
27	oar3_OAR›	17885918	0.027898
27	oar3_OAR›	17890638	0.070334
27	oar3_OAR›	17890966	0.070334
27	oar3_OAR›	17904657	-0.0115
27	oar3_OAR›	17904827	-0.0115
27	oar3_OAR›	17909233	0.003939
27	oar3_OAR›	17912321	0.064242
27	oar3_OAR›	17917540	0.034254
27	oar3_OAR›	17920320	-0.00209
27	oar3_OAR›	17940531	0.000415
27	oar3_OAR›	17949113	0.019309
27	oar3_OAR›	17950769	0.053574
27	oar3_OAR›	17951911	-0.03617
27	oar3_OAR›	17963961	-0.01089
27	oar3_OAR›	17969438	0.039198

27	oar3_OAR›	17976723	0.030975
27	oar3_OAR›	17981738	0.019377
27	oar3_OAR›	17990867	-0.03858
27	oar3_OAR›	17995073	0.053574
27	oar3_OAR›	18010575	-0.01817
27	oar3_OAR›	18019457	-0.01817
27	oar3_OAR›	18024500	-0.03497
27	oar3_OAR›	18024823	0.004606
27	oar3_OAR›	18025462	0.004606
27	oar3_OAR›	18032559	-0.02576
27	oar3_OAR›	18044039	0.011067
27	oar3_OAR›	18058045	-0.02205
27	oar3_OAR›	18059840	-0.02205
27	oar3_OAR›	18063230	-0.02205
27	oar3_OAR›	18063393	-0.02205
27	oar3_OAR›	18071285	-0.0325
27	oar3_OAR›	18074668	-0.02222
27	oar3_OAR›	18080560	-0.02773
27	oar3_OAR›	18084883	-0.02773
27	oar3_OAR›	18088528	-0.0318
27	oar3_OAR›	18088798	-0.03282
27	oar3_OAR›	18101787	0.024495
27	oar3_OAR›	18111858	0.008723
27	oar3_OAR›	18112026	0.007039
27	oar3_OAR›	18112704	-0.01917
27	oar3_OAR›	18124470	-0.01679
27	oar3_OAR›	18128117	-0.00585
27	oar3_OAR›	18129142	-0.00164
27	oar3_OAR›	18140370	0.023353
27	oar3_OAR›	18159184	-0.02856
27	oar3_OAR›	18160428	-0.00857
27	oar3_OAR›	18163116	-0.01822
27	oar3_OAR›	18178355	NA
27	oar3_OAR›	18189235	0.097307
27	oar3_OAR›	18196095	0.052486
27	oar3_OAR›	18196688	-0.0178
27	oar3_OAR›	18207154	0.021253
27	oar3_OAR›	18212820	0.039199
27	oar3_OAR›	18213279	0.011067
27	oar3_OAR›	18218088	0.059631
27	oar3_OAR›	18222976	0.028919
27	oar3_OAR›	18226162	-0.02217
27	oar3_OAR›	18240056	-0.03477
27	oar3_OAR›	18240318	-0.03477
27	oar3_OAR›	18250026	0.011067
27	oar3_OAR›	18250938	0.059631
27	oar3_OAR›	18258487	0.059631
27	oar3_OAR›	18261601	0.09203
27	oar3_OAR›	18266322	0.023093
27	oar3_OAR›	18268564	0.059631
27	oar3_OAR›	18274993	0.011067
27	oar3_OAR›	18278778	0.013644
27	oar3_OAR›	18279335	0.013644
27	oar3_OAR›	18279534	-0.04077

27	oar3_OAR›	18285260	-0.00407
27	oar3_OAR›	18291222	-0.02186
27	oar3_OAR›	18295272	-0.01773
27	oar3_OAR›	18298173	-0.02186
27	oar3_OAR›	18301302	-0.01773
27	oar3_OAR›	18302042	-0.02064
27	oar3_OAR›	18306899	-0.00574
27	oar3_OAR›	18308097	-0.00574
27	oar3_OAR›	18312249	-0.01767
27	oar3_OAR›	18326198	-0.00682
27	oar3_OAR›	18332873	0.006819
27	oar3_OAR›	18336286	-0.00682
27	oar3_OAR›	18341837	0.025831
27	oar3_OAR›	18354965	0.025831
27	oar3_OAR›	18355451	-0.03168
27	oar3_OAR›	18366313	0.003256
27	oar3_OAR›	18375700	-0.01206
27	oar3_OAR›	18384447	0.029783
27	oar3_OAR›	18385439	-0.0054
27	oar3_OAR›	18388409	0.026955
27	oar3_OAR›	18402732	0.003256
27	oar3_OAR›	18409842	-0.01203
27	oar3_OAR›	18409926	-0.00362
27	oar3_OAR›	18422395	-0.01203
27	oar3_OAR›	18443439	0.018772
27	oar3_OAR›	18443507	0.005595
27	oar3_OAR›	18445679	-0.03171
27	oar3_OAR›	18449485	-0.01405
27	oar3_OAR›	18449771	0.005595
27	oar3_OAR›	18459902	-0.01255
27	oar3_OAR›	18462779	-0.02466
27	oar3_OAR›	18468334	########
27	oar3_OAR›	18482670	-0.01658
27	oar3_OAR›	18486566	-0.01551
27	oar3_OAR›	18498404	-0.00432
27	oar3_OAR›	18502708	0.017028
27	oar3_OAR›	18536512	0.050165
27	oar3_OAR›	18542532	0.060014
27	oar3_OAR›	18550823	-0.00427
27	oar3_OAR›	18556110	0.05685
27	oar3_OAR›	18558713	-0.00056
27	oar3_OAR›	18577832	0.016173
27	oar3_OAR›	18587692	0.088722
27	oar3_OAR›	18590151	-0.02279
27	oar3_OAR›	18598530	-0.02279
27	oar3_OAR›	18598888	0.027215
27	oar3_OAR›	18599437	0.027215
27	oar3_OAR›	18600274	0.088722
27	oar3_OAR›	18618154	-0.02279
27	oar3_OAR›	18625290	0.016219
27	oar3_OAR›	18631884	0.088722
27	oar3_OAR›	18634362	0.021224
27	oar3_OAR›	18641175	-0.00462
27	oar3_OAR›	18642415	0.009572

27	oar3_OAR›	18642603	0.009572
27	oar3_OAR›	18648175	0.035931
27	oar3_OAR›	18652638	0.028704
27	oar3_OAR›	18653081	0.028704
27	oar3_OAR›	18656767	0.033554
27	oar3_OAR›	18663166	0.033554
27	oar3_OAR›	18663228	0.073408
27	oar3_OAR›	18673768	0.028704
27	oar3_OAR›	18686183	NA
27	oar3_OAR›	18687659	0.058233
27	oar3_OAR›	18692706	0.083796
27	oar3_OAR›	18699040	0.037818
27	oar3_OAR›	18699074	-0.01639
27	oar3_OAR›	18700438	0.037818
27	oar3_OAR›	18704922	NA
27	oar3_OAR›	18707408	0.048056
27	oar3_OAR›	18712738	0.071606
27	oar3_OAR›	18717646	-0.00606
27	oar3_OAR›	18718013	0.071606
27	oar3_OAR›	18733829	0.05272
27	oar3_OAR›	18742692	0.082774
27	oar3_OAR›	18747371	0.043765
27	oar3_OAR›	18757050	-0.03461
27	oar3_OAR›	18762762	-0.02562
27	oar3_OAR›	18765819	-0.00836
27	oar3_OAR›	18777960	-0.00641
27	oar3_OAR›	18784666	-0.0184
27	oar3_OAR›	18785482	0.044084
27	oar3_OAR›	18789636	0.023164
27	oar3_OAR›	18797890	0.115355
27	oar3_OAR›	18798914	0.115355
27	oar3_OAR›	18810297	0.008296
27	oar3_OAR›	18810420	0.096188
27	oar3_OAR›	18818627	0.023763
27	oar3_OAR›	18829465	0.108872
27	oar3_OAR›	18829984	-0.00809
27	oar3_OAR›	18836177	-0.03565
27	oar3_OAR›	18836243	-0.03565
27	oar3_OAR›	18848225	0.045719
27	oar3_OAR›	18856389	-0.01519
27	oar3_OAR›	18856633	0.027457
27	oar3_OAR›	18861918	0.034066
27	oar3_OAR›	18868193	-0.00718
27	oar3_OAR›	18868524	0.052269
27	oar3_OAR›	18868604	0.109621
27	oar3_OAR›	18873190	-0.00099
27	oar3_OAR›	18874993	0.055547
27	oar3_OAR›	18880176	-0.00099
27	oar3_OAR›	18881144	0.022043
27	oar3_OAR›	18883714	0.002868
27	oar3_OAR›	18884451	0.007587
27	oar3_OAR›	18889183	0.022722
27	oar3_OAR›	18893167	0.022722
27	oar3_OAR›	18897907	0.023898

27	oar3_OAR〉	18898385	0.050922
27	oar3_OAR〉	18903868	0.046951
27	oar3_OAR〉	18904819	-0.01257
27	oar3_OAR〉	18910821	-0.01257
27	oar3_OAR〉	18915219	0.057021
27	oar3_OAR〉	18926493	-0.0309
27	oar3_OAR〉	18929810	0.003054
27	oar3_OAR〉	18930186	0.003054
27	oar3_OAR〉	18944566	-0.01605
27	oar3_OAR〉	18952831	-0.01574
27	oar3_OAR〉	18968205	-0.00195
27	oar3_OAR〉	18976058	-0.01789
27	oar3_OAR〉	18976673	-0.00195
27	oar3_OAR〉	18977713	-0.00195
27	oar3_OAR〉	18978393	-0.00195
27	oar3_OAR〉	18985621	0.000363
27	oar3_OAR〉	18988724	-0.00444
27	oar3_OAR〉	18998923	0.018459
27	oar3_OAR〉	19007291	0.000363
27	oar3_OAR〉	19010046	-0.02209
27	oar3_OAR〉	19010748	0.042256
27	oar3_OAR〉	19012111	-0.01735
27	oar3_OAR〉	19017080	0.000605
27	oar3_OAR〉	19017593	0.000363
27	oar3_OAR〉	19022030	-0.02209
27	oar3_OAR〉	19029612	0.000363
27	oar3_OAR〉	19031107	-0.0146
27	oar3_OAR〉	19032109	-0.0054
27	oar3_OAR〉	19038533	-0.00034
27	oar3_OAR〉	19039092	0.071872
27	oar3_OAR〉	19046161	0.047855
27	oar3_OAR〉	19046343	0.077843
27	oar3_OAR〉	19053302	-0.00034
27	oar3_OAR〉	19053306	0.028803
27	oar3_OAR〉	19056541	-0.01995
27	oar3_OAR〉	19057860	0.024934
27	oar3_OAR〉	19059079	0.028803
27	oar3_OAR〉	19065184	0.000363
27	oar3_OAR〉	19067519	-0.02344
27	oar3_OAR〉	19070414	0.021942
27	oar3_OAR〉	19070848	-0.01859
27	oar3_OAR〉	19074719	0.000363
27	oar3_OAR〉	19079833	-0.02913
27	oar3_OAR〉	19083972	-0.03098
27	oar3_OAR〉	19084317	0.016275
27	oar3_OAR〉	19092807	-0.02332
27	oar3_OAR〉	19096582	0.02374
27	oar3_OAR〉	19098162	0.023798
27	oar3_OAR〉	19101314	0.005349
27	oar3_OAR〉	19112422	0.02374
27	oar3_OAR〉	19113147	0.02374
27	oar3_OAR〉	19116698	-0.02049
27	oar3_OAR〉	19118965	-0.00363
27	oar3_OAR〉	19122133	0.02374

27	oar3_OAR〉	19123456	0.02374
27	oar3_OAR〉	19133532	0.001746
27	oar3_OAR〉	19134694	-0.02716
27	oar3_OAR〉	19137295	0.014031
27	oar3_OAR〉	19141727	-0.02176
27	oar3_OAR〉	19154695	-0.01402
27	oar3_OAR〉	19154823	0.040807
27	oar3_OAR〉	19158099	0.051759
27	oar3_OAR〉	19164748	0.030733
27	oar3_OAR〉	19168981	0.086031
27	oar3_OAR〉	19178906	0.057401
27	oar3_OAR〉	19184664	-0.01816
27	oar3_OAR〉	19189790	0.018804
27	oar3_OAR〉	19192769	-0.02254
27	oar3_OAR〉	19195576	0.065149
27	oar3_OAR〉	19196328	0.029401
27	oar3_OAR〉	19204632	0.008723
27	oar3_OAR〉	19205605	0.017067
27	oar3_OAR〉	19210700	0.04976
27	oar3_OAR〉	19214131	-0.01192
27	oar3_OAR〉	19221204	-0.00382
27	oar3_OAR〉	19226093	0.029473
27	oar3_OAR〉	19230042	-0.01486
27	oar3_OAR〉	19234615	-0.00389
27	oar3_OAR〉	19248135	0.018117
27	oar3_OAR〉	19251571	0.066458
27	oar3_OAR〉	19256959	0.072373
27	oar3_OAR〉	19265689	-0.00166
27	oar3_OAR〉	19270184	0.059334
27	oar3_OAR〉	19270477	0.042587
27	oar3_OAR〉	19271756	0.042587
27	oar3_OAR〉	19281988	0.055406
27	oar3_OAR〉	19287181	0.068095
27	oar3_OAR〉	19289385	0.026605
27	oar3_OAR〉	19293363	0.061397
27	oar3_OAR〉	19294511	0.061397
27	oar3_OAR〉	19295904	0.061397
27	oar3_OAR〉	19299415	0.061397
27	oar3_OAR〉	19301112	0.082638
27	oar3_OAR〉	19306645	0.023635
27	oar3_OAR〉	19306774	0.031144
27	oar3_OAR〉	19310109	0.005952
27	oar3_OAR〉	19315283	0.036974
27	oar3_OAR〉	19318539	0.039646
27	oar3_OAR〉	19321045	0.041494
27	oar3_OAR〉	19325137	0.036205
27	oar3_OAR〉	19326604	0.067699
27	oar3_OAR〉	19331190	0.067699
27	oar3_OAR〉	19332091	0.053169
27	oar3_OAR〉	19339813	-0.0218
27	oar3_OAR〉	19344414	0.001207
27	oar3_OAR〉	19344655	-0.01288
27	oar3_OAR〉	19344725	-0.03437
27	oar3_OAR〉	19344860	-0.03437

27	oar3_OAR›	19351807	0.000196
27	oar3_OAR›	19353598	0.002684
27	oar3_OAR›	19354817	-0.00698
27	oar3_OAR›	19362655	0.000196
27	oar3_OAR›	19366995	0.076106
27	oar3_OAR›	19368274	0.093951
27	oar3_OAR›	19373050	0.000196
27	oar3_OAR›	19379057	0.06651
27	oar3_OAR›	19390345	-0.00259
27	oar3_OAR›	19392327	0.003086
27	oar3_OAR›	19401625	0.008431
27	oar3_OAR›	19404073	0.035118
27	oar3_OAR›	19407677	0.056508
27	oar3_OAR›	19412684	-0.01537
27	oar3_OAR›	19412950	0.003509
27	oar3_OAR›	19417421	0.027788
27	oar3_OAR›	19422106	-0.0284
27	oar3_OAR›	19436094	-0.0291
27	oar3_OAR›	19441332	-0.00075
27	oar3_OAR›	19445987	-0.02863
27	oar3_OAR›	19453977	0.056064
27	oar3_OAR›	19454631	0.014267
27	oar3_OAR›	19456043	0.014267
27	oar3_OAR›	19457981	-0.00941
27	oar3_OAR›	19463021	0.014944
27	oar3_OAR›	19465673	0.007351
27	oar3_OAR›	19468400	0.22363
27	oar3_OAR›	19471620	0.013976
27	oar3_OAR›	19476082	0.013976
27	oar3_OAR›	19480304	0.028277
27	oar3_OAR›	19480658	0.013976
27	oar3_OAR›	19487173	0.005379
27	oar3_OAR›	19487238	0.013976
27	oar3_OAR›	19487437	0.103185
27	oar3_OAR›	19492213	0.005379
27	oar3_OAR›	19497791	0.024925
27	oar3_OAR›	19497938	0.008884
27	oar3_OAR›	19499811	0.035888
27	oar3_OAR›	19502950	-0.00761
27	oar3_OAR›	19509792	-0.00342
27	oar3_OAR›	19509910	0.05355
27	oar3_OAR›	19518594	-0.01383
27	oar3_OAR›	19518726	-0.01383
27	oar3_OAR›	19522947	-0.02603
27	oar3_OAR›	19523061	0.02878
27	oar3_OAR›	19533215	0.028315
27	oar3_OAR›	19533246	-0.01092
27	oar3_OAR›	19533435	-0.01674
27	oar3_OAR›	19537078	-0.01569
27	oar3_OAR›	19542057	-0.02577
27	oar3_OAR›	19543317	-0.02375
27	oar3_OAR›	19549288	-0.02216
27	oar3_OAR›	19554425	-0.02822
27	oar3_OAR›	19566832	-0.00516

27	oar3_OAR›	19567242	-0.00516
27	oar3_OAR›	19568320	-0.01456
27	oar3_OAR›	19572461	-0.00953
27	oar3_OAR›	19572605	0.012512
27	oar3_OAR›	19578370	-0.02497
27	oar3_OAR›	19578493	-0.02693
27	oar3_OAR›	19586610	0.104279
27	oar3_OAR›	19588682	-0.0143
27	oar3_OAR›	19588986	-0.01905
27	oar3_OAR›	19594398	0.030434
27	oar3_OAR›	19602143	-0.01959
27	oar3_OAR›	19605405	-0.0109
27	oar3_OAR›	19615771	-0.0109
27	oar3_OAR›	19616431	-0.0109
27	oar3_OAR›	19623041	-0.03099
27	oar3_OAR›	19623867	-0.03228
27	oar3_OAR›	19628449	0.023689
27	oar3_OAR›	19639144	-0.01434
27	oar3_OAR›	19639719	0.082519
27	oar3_OAR›	19639896	0.003071
27	oar3_OAR›	19649055	0.009324
27	oar3_OAR›	19651291	0.124133
27	oar3_OAR›	19657552	0.003628
27	oar3_OAR›	19660092	0.032056
27	oar3_OAR›	19660254	0.110676
27	oar3_OAR›	19662924	-0.01428
27	oar3_OAR›	19664936	0.018601
27	oar3_OAR›	19678727	0.018601
27	oar3_OAR›	19687214	-0.02308
27	oar3_OAR›	19693877	0.018601
27	oar3_OAR›	19694414	0.056503
27	oar3_OAR›	19699083	-0.03151
27	oar3_OAR›	19710218	0.01079
27	oar3_OAR›	19741108	0.00703
27	oar3_OAR›	19747664	0.023793
27	oar3_OAR›	19747769	-0.00026
27	oar3_OAR›	19763983	-0.02151
27	oar3_OAR›	19765490	0.082199
27	oar3_OAR›	19766269	0.022519
27	oar3_OAR›	19766341	0.056503
27	oar3_OAR›	19781394	0.079606
27	oar3_OAR›	19782623	-0.01274
27	oar3_OAR›	19786784	0.029023
27	oar3_OAR›	19787744	0.016819
27	oar3_OAR›	19792417	-0.03768
27	oar3_OAR›	19801349	0.009617
27	oar3_OAR›	19801869	-0.02179
27	oar3_OAR›	19815546	0.050366
27	oar3_OAR›	19822582	0.013963
27	oar3_OAR›	19825570	-0.00606
27	oar3_OAR›	19830239	NA
27	oar3_OAR›	19831714	-0.0294
27	oar3_OAR›	19833400	0.043847
27	oar3_OAR›	19835974	0.043847

27	oar3_OAR>	19836857	-0.02872
27	oar3_OAR>	19842418	0.173709
27	oar3_OAR>	19843304	0.064221
27	oar3_OAR>	19845576	0.064221
27	oar3_OAR>	19848157	0.064221
27	oar3_OAR>	19849182	0.064221
27	oar3_OAR>	19860325	0.011581
27	oar3_OAR>	19864035	0.070246
27	oar3_OAR>	19873594	-0.01313
27	oar3_OAR>	19879722	-0.01443
27	oar3_OAR>	19885597	0.024838
27	oar3_OAR>	19894071	-0.01608
27	oar3_OAR>	19895822	0.016062
27	oar3_OAR>	19899017	0.050888
27	oar3_OAR>	19899096	0.059793
27	oar3_OAR>	19904598	0.003004
27	oar3_OAR>	19906922	0.020767
27	oar3_OAR>	19914708	-0.02739
27	oar3_OAR>	19915422	-0.01636
27	oar3_OAR>	19917117	-0.01636
27	oar3_OAR>	19921520	0.022666
27	oar3_OAR>	19928383	-0.00049
27	oar3_OAR>	19929940	0.005042
27	oar3_OAR>	19939193	-0.01404
27	oar3_OAR>	19939331	0.003214
27	oar3_OAR>	19940204	0.003214
27	oar3_OAR>	19942256	0.086809
27	oar3_OAR>	19947685	0.025535
27	oar3_OAR>	19949729	0.094362
27	oar3_OAR>	19951774	-0.02465
27	oar3_OAR>	19962084	0.120487
27	oar3_OAR>	19963547	0.120487
27	oar3_OAR>	19965778	0.120996
27	oar3_OAR>	19972598	-0.01588
27	oar3_OAR>	19973975	-0.03768
27	oar3_OAR>	19975210	0.065685
27	oar3_OAR>	19978691	0.020267
27	oar3_OAR>	19984138	0.189185
27	oar3_OAR>	19990884	-0.00024
27	oar3_OAR>	19991933	0.050453
27	oar3_OAR>	19996218	0.008484
27	oar3_OAR>	19998728	-0.02415
27	oar3_OAR>	20008493	0.0175
27	oar3_OAR>	20011252	-0.00575
27	oar3_OAR>	20012257	0.007381
27	oar3_OAR>	20021656	-0.02453
27	oar3_OAR>	20034051	-0.00199
27	oar3_OAR>	20044842	-0.01204
27	oar3_OAR>	20046453	-0.01834
27	oar3_OAR>	20049000	0.005042
27	oar3_OAR>	20054951	0.011932
27	oar3_OAR>	20056661	-0.01286
27	oar3_OAR>	20057060	0.011932
27	oar3_OAR>	20057976	-0.00597

27	oar3_OAR›	20066832	-0.02926
27	oar3_OAR›	20067630	0.005111
27	oar3_OAR›	20074461	0.07082
27	oar3_OAR›	20074473	0.04635
27	oar3_OAR›	20088719	0.053745
27	oar3_OAR›	20091579	-0.03151
27	oar3_OAR›	20095173	-0.03151
27	oar3_OAR›	20108480	-0.01657
27	oar3_OAR›	20113683	-0.01657
27	oar3_OAR›	20125291	-0.03305
27	oar3_OAR›	20133630	0.093869
27	oar3_OAR›	20134659	0.11259
27	oar3_OAR›	20136966	0.05186
27	oar3_OAR›	20147027	-0.02759
27	oar3_OAR›	20147659	0.000889
27	oar3_OAR›	20152434	0.036429
27	oar3_OAR›	20155108	0.078896
27	oar3_OAR›	20180233	0.020834
27	oar3_OAR›	20184869	0.03658
27	oar3_OAR›	20197880	0.020834
27	oar3_OAR›	20198851	0.006423
27	oar3_OAR›	20201101	0.062636
27	oar3_OAR›	20207158	0.006423
27	oar3_OAR›	20222544	0.02379
27	oar3_OAR›	20222645	0.020834
27	oar3_OAR›	20227432	0.006981
27	oar3_OAR›	20231406	0.006981
27	oar3_OAR›	20236398	-0.01605
27	oar3_OAR›	20237191	0.032181
27	oar3_OAR›	20243641	0.02379
27	oar3_OAR›	20248394	0.044724
27	oar3_OAR›	20251104	0.006423
27	oar3_OAR›	20259306	0.03648
27	oar3_OAR›	20259440	-0.00881
27	oar3_OAR›	20271411	0.02306
27	oar3_OAR›	20280575	0.018388
27	oar3_OAR›	20284875	0.03648
27	oar3_OAR›	20290604	-0.00693
27	oar3_OAR›	20290677	0.033689
27	oar3_OAR›	20300451	0.019049
27	oar3_OAR›	20307171	-0.00693
27	oar3_OAR›	20308218	-0.00693
27	oar3_OAR›	20308645	-0.00693
27	oar3_OAR›	20313834	0.021081
27	oar3_OAR›	20321128	-0.0324
27	oar3_OAR›	20321992	0.021081
27	oar3_OAR›	20322324	0.03652
27	oar3_OAR›	20334268	0.045627
27	oar3_OAR›	20334430	-0.00462
27	oar3_OAR›	20347251	-0.00392
27	oar3_OAR›	20352284	0.180784
27	oar3_OAR›	20357881	0.180784
27	oar3_OAR›	20360548	0.020295
27	oar3_OAR›	20361934	0.020295

27	oar3_OAR〉	20388465	0.072293
27	oar3_OAR〉	20392713	0.123939
27	oar3_OAR〉	20392791	0.066132
27	oar3_OAR〉	20398282	0.033773
27	oar3_OAR〉	20401135	0.066132
27	oar3_OAR〉	20404185	0.033773
27	oar3_OAR〉	20410342	0.113479
27	oar3_OAR〉	20413414	0.063868
27	oar3_OAR〉	20431572	0.036767
27	oar3_OAR〉	20431841	0.123939
27	oar3_OAR〉	20433055	0.123939
27	oar3_OAR〉	20433614	-0.0294
27	oar3_OAR〉	20445213	0.012115
27	oar3_OAR〉	20448249	0.05528
27	oar3_OAR〉	20453782	-0.00462
27	oar3_OAR〉	20455027	0.005471
27	oar3_OAR〉	20455528	0.05528
27	oar3_OAR〉	20462126	0.043437
27	oar3_OAR〉	20464883	0.025417
27	oar3_OAR〉	20469740	-0.02222
27	oar3_OAR〉	20477379	-0.02176
27	oar3_OAR〉	20478606	-0.00044
27	oar3_OAR〉	20483994	-0.01602
27	oar3_OAR〉	20492623	-0.02188
27	oar3_OAR〉	20492941	-0.02176
27	oar3_OAR〉	20493507	0.071634
27	oar3_OAR〉	20498448	-0.02188
27	oar3_OAR〉	20501644	-0.02534
27	oar3_OAR〉	20504221	-0.0148
27	oar3_OAR〉	20504383	0.013236
27	oar3_OAR〉	20509892	0.013236
27	oar3_OAR〉	20514198	-0.02176
27	oar3_OAR〉	20514434	0.013236
27	oar3_OAR〉	20516002	0.013236
27	oar3_OAR〉	20528948	-0.02749
27	oar3_OAR〉	20538474	-0.00239
27	oar3_OAR〉	20550316	-0.02799
27	oar3_OAR〉	20552563	-0.00044
27	oar3_OAR〉	20561301	0.083626
27	oar3_OAR〉	20564159	-0.02799
27	oar3_OAR〉	20571151	-0.01423
27	oar3_OAR〉	20582163	-0.01423
27	oar3_OAR〉	20583766	0.034256
27	oar3_OAR〉	20585767	-0.00239
27	oar3_OAR〉	20590145	0.050654
27	oar3_OAR〉	20591673	0.050654
27	oar3_OAR〉	20592942	0.029788
27	oar3_OAR〉	20599501	0.034256
27	oar3_OAR〉	20599757	0.034256
27	oar3_OAR〉	20600792	-0.01834
27	oar3_OAR〉	20602937	0.125607
27	oar3_OAR〉	20608166	0.015429
27	oar3_OAR〉	20608920	0.001366
27	oar3_OAR〉	20610172	0.09111

27	oar3_OAR›	20617100	0.036205
27	oar3_OAR›	20618839	0.001366
27	oar3_OAR›	20625798	0.05901
27	oar3_OAR›	20626715	0.013414
27	oar3_OAR›	20627060	0.013414
27	oar3_OAR›	20632808	0.023064
27	oar3_OAR›	20638032	0.029753
27	oar3_OAR›	20642333	-0.00349
27	oar3_OAR›	20647574	0.013908
27	oar3_OAR›	20653729	0.013908
27	oar3_OAR›	20669622	-0.00349
27	oar3_OAR›	20674836	0.009761
27	oar3_OAR›	20678929	0.060339
27	oar3_OAR›	20680651	0.060339
27	oar3_OAR›	20691770	0.00268
27	oar3_OAR›	20697040	0.009761
27	oar3_OAR›	20697287	0.023064
27	oar3_OAR›	20708534	0.013776
27	oar3_OAR›	20709196	0.023064
27	oar3_OAR›	20709629	-0.01777
27	oar3_OAR›	20715217	0.017206
27	oar3_OAR›	20718297	-0.01638
27	oar3_OAR›	20719888	0.017146
27	oar3_OAR›	20723542	0.009761
27	oar3_OAR›	20726417	0.017206
27	oar3_OAR›	20731757	0.060339
27	oar3_OAR›	20733351	0.060339
27	oar3_OAR›	20735168	0.016378
27	oar3_OAR›	20738795	0.026045
27	oar3_OAR›	20740748	0.070463
27	oar3_OAR›	20746803	0.020467
27	oar3_OAR›	20748504	0.020467
27	oar3_OAR›	20751949	0.025831
27	oar3_OAR›	20756248	0.120662
27	oar3_OAR›	20761214	0.12535
27	oar3_OAR›	20762649	0.096817
27	oar3_OAR›	20765437	0.049265
27	oar3_OAR›	20767310	0.023963
27	oar3_OAR›	20772724	0.020467
27	oar3_OAR›	20773927	0.020467
27	oar3_OAR›	20779077	-0.00847
27	oar3_OAR›	20785374	0.12535
27	oar3_OAR›	20785432	0.088785
27	oar3_OAR›	20788807	0.086413
27	oar3_OAR›	20791764	0.020467
27	oar3_OAR›	20794244	0.020467
27	oar3_OAR›	20812274	0.036907
27	oar3_OAR›	20812401	0.021523
27	oar3_OAR›	20812769	0.051954
27	oar3_OAR›	20813348	0.021523
27	oar3_OAR›	20824455	-0.00902
27	oar3_OAR›	20845120	-0.01955
27	oar3_OAR›	20850075	0.015
27	oar3_OAR›	20850818	0.015863

27	oar3_OAR›	20853299	0.050228
27	oar3_OAR›	20870080	-0.01465
27	oar3_OAR›	20873733	-0.00056
27	oar3_OAR›	20882522	-0.00056
27	oar3_OAR›	20883062	0.018227
27	oar3_OAR›	20883189	-0.01143
27	oar3_OAR›	20884897	0.018227
27	oar3_OAR›	20899974	0.018227
27	oar3_OAR›	20901068	-0.00052
27	oar3_OAR›	20902910	-0.01143
27	oar3_OAR›	20903056	0.018227
27	oar3_OAR›	20907065	-0.00052
27	oar3_OAR›	20937069	-0.02142
27	oar3_OAR›	20945585	0.054599
27	oar3_OAR›	20950117	0.011041
27	oar3_OAR›	20969944	-0.00301
27	oar3_OAR›	20970045	-0.00301
27	oar3_OAR›	20972942	0.046122
27	oar3_OAR›	20975974	-0.00301
27	oar3_OAR›	20984077	0.011041
27	oar3_OAR›	20985069	-0.01533
27	oar3_OAR›	20991180	0.023749
27	oar3_OAR›	21011028	-0.01533
27	oar3_OAR›	21018715	0.072901
27	oar3_OAR›	21018804	0.046122
27	oar3_OAR›	21020887	0.045535
27	oar3_OAR›	21026575	0.04151
27	oar3_OAR›	21029261	0.007354
27	oar3_OAR›	21030968	0.052315
27	oar3_OAR›	21038562	-0.02176
27	oar3_OAR›	21040487	-0.03114
27	oar3_OAR›	21041022	-0.02507
27	oar3_OAR›	21043910	-0.01107
27	oar3_OAR›	21051668	0.04778
27	oar3_OAR›	21052973	-0.01127
27	oar3_OAR›	21053479	-0.01127
27	oar3_OAR›	21060696	-0.01694
27	oar3_OAR›	21067083	0.03648
27	oar3_OAR›	21075007	-0.02237
27	oar3_OAR›	21075811	-0.02959
27	oar3_OAR›	21076522	0.001316
27	oar3_OAR›	21086742	-0.02292
27	oar3_OAR›	21093672	-0.02216
27	oar3_OAR›	21099729	0.049327
27	oar3_OAR›	21106681	-0.02044
27	oar3_OAR›	21106963	0.038173
27	oar3_OAR›	21113100	0.030402
27	oar3_OAR›	21120633	0.041871
27	oar3_OAR›	21126035	-0.01547
27	oar3_OAR›	21132561	-0.00064
27	oar3_OAR›	21133145	-0.01255
27	oar3_OAR›	21142721	-0.01908
27	oar3_OAR›	21142895	-0.0262
27	oar3_OAR›	21151933	-0.00731

27	oar3_OAR〉	21156340	-0.00731
27	oar3_OAR〉	21159365	0.011531
27	oar3_OAR〉	21167002	-0.00731
27	oar3_OAR〉	21175931	0.039798
27	oar3_OAR〉	21179887	0.054035
27	oar3_OAR〉	21189852	-0.00668
27	oar3_OAR〉	21190375	0.039227
27	oar3_OAR〉	21190655	-0.01082
27	oar3_OAR〉	21195278	0.018172
27	oar3_OAR〉	21196694	0.164267
27	oar3_OAR〉	21218580	0.007028
27	oar3_OAR〉	21228230	-0.02831
27	oar3_OAR〉	21230942	-0.01132
27	oar3_OAR〉	21233013	-0.00631
27	oar3_OAR〉	21234245	-0.01662
27	oar3_OAR〉	21241976	0.013486
27	oar3_OAR〉	21244617	-0.01121
27	oar3_OAR〉	21246779	0.092888
27	oar3_OAR〉	21251388	-0.01032
27	oar3_OAR〉	21257341	0.149879
27	oar3_OAR〉	21258020	0.038328
27	oar3_OAR〉	21258072	-0.01745
27	oar3_OAR〉	21263779	0.083404
27	oar3_OAR〉	21264162	-0.03565
27	oar3_OAR〉	21273331	-0.03565
27	oar3_OAR〉	21274147	0.038394
27	oar3_OAR〉	21285236	0.145562
27	oar3_OAR〉	21308552	0.005579
27	oar3_OAR〉	21328684	-0.01
27	oar3_OAR〉	21331312	0.108913
27	oar3_OAR〉	21331377	0.048625
27	oar3_OAR〉	21337378	0.183417
27	oar3_OAR〉	21346050	-0.01791
27	oar3_OAR〉	21346178	0.088624
27	oar3_OAR〉	21347591	0.100853
27	oar3_OAR〉	21353140	0.243475
27	oar3_OAR〉	21354891	-0.01381
27	oar3_OAR〉	21358471	0.100853
27	oar3_OAR〉	21359281	0.066022
27	oar3_OAR〉	21383610	-0.00887
27	oar3_OAR〉	21384951	0.036271
27	oar3_OAR〉	21385276	-0.01802
27	oar3_OAR〉	21396608	-0.02199
27	oar3_OAR〉	21397772	0.002541
27	oar3_OAR〉	21400616	0.04559
27	oar3_OAR〉	21405966	0.066891
27	oar3_OAR〉	21427506	-0.01069
27	oar3_OAR〉	21428673	0.023798
27	oar3_OAR〉	21453409	-0.027
27	oar3_OAR〉	21456217	-0.00568
27	oar3_OAR〉	21464551	-0.00568
27	oar3_OAR〉	21466570	0.03488
27	oar3_OAR〉	21470045	-0.0147
27	oar3_OAR〉	21474005	0.004461

27	oar3_OAR)	21475169	-0.00568
27	oar3_OAR)	21476226	0.004183
27	oar3_OAR)	21485080	-0.01356
27	oar3_OAR)	21486496	0.004461
27	oar3_OAR)	21493330	-0.02526
27	oar3_OAR)	21512565	7.25E-05
27	oar3_OAR)	21512704	7.25E-05
27	oar3_OAR)	21512845	7.25E-05
27	oar3_OAR)	21519276	0.056789
27	oar3_OAR)	21521032	-0.00188
27	oar3_OAR)	21523576	-0.01817
27	oar3_OAR)	21523837	-0.02369
27	oar3_OAR)	21531901	0.000195
27	oar3_OAR)	21532454	0.032551
27	oar3_OAR)	21535392	-0.00857
27	oar3_OAR)	21540125	-0.02576
27	oar3_OAR)	21540154	0.001262
27	oar3_OAR)	21546290	-0.03098
27	oar3_OAR)	21547597	-0.01176
27	oar3_OAR)	21554117	-0.01138
27	oar3_OAR)	21554209	-0.01525
27	oar3_OAR)	21566464	-0.02877
27	oar3_OAR)	21568187	-0.02818
27	oar3_OAR)	21570196	-0.00524
27	oar3_OAR)	21571575	0.011513
27	oar3_OAR)	21586528	-0.00037
27	oar3_OAR)	21589440	-0.0029
27	oar3_OAR)	21591056	-0.02398
27	oar3_OAR)	21596337	-0.00121
27	oar3_OAR)	21613403	-0.01093
27	oar3_OAR)	21614115	-0.01705
27	oar3_OAR)	21614368	-0.0029
27	oar3_OAR)	21619720	-0.00462
27	oar3_OAR)	21623840	0.003335
27	oar3_OAR)	21631639	-0.00651
27	oar3_OAR)	21632046	-0.02633
27	oar3_OAR)	21632129	-0.01334
27	oar3_OAR)	21633378	0.095767
27	oar3_OAR)	21638726	-0.01334
27	oar3_OAR)	21645566	0.10269
27	oar3_OAR)	21645938	0.10269
27	oar3_OAR)	21646665	0.095871
27	oar3_OAR)	21649805	-0.02316
27	oar3_OAR)	21656059	-0.0248
27	oar3_OAR)	21657080	-0.00215
27	oar3_OAR)	21661818	-0.00215
27	oar3_OAR)	21666288	0.012151
27	oar3_OAR)	21673196	-0.01356
27	oar3_OAR)	21675017	-0.01356
27	oar3_OAR)	21677719	-0.01618
27	oar3_OAR)	21678203	-0.01618
27	oar3_OAR)	21681543	-0.00357
27	oar3_OAR)	21683327	-0.03549
27	oar3_OAR)	21688959	0.036107

27	oar3_OAR›	21689069	0.016478
27	oar3_OAR›	21689723	0.007294
27	oar3_OAR›	21691534	0.004423
27	oar3_OAR›	21695138	0.028897
27	oar3_OAR›	21699757	0.051444
27	oar3_OAR›	21700862	0.070211
27	oar3_OAR›	21704748	-0.00344
27	oar3_OAR›	21715076	0.055972
27	oar3_OAR›	21719558	0.055972
27	oar3_OAR›	21725311	0.059679
27	oar3_OAR›	21730195	0.014347
27	oar3_OAR›	21731634	0.018169
27	oar3_OAR›	21735126	-0.0031
27	oar3_OAR›	21736649	-0.02087
27	oar3_OAR›	21741284	-0.00853
27	oar3_OAR›	21742063	-0.01822
27	oar3_OAR›	21742220	0.008681
27	oar3_OAR›	21747294	-0.00097
27	oar3_OAR›	21750047	0.017146
27	oar3_OAR›	21751372	0.001274
27	oar3_OAR›	21752929	0.017146
27	oar3_OAR›	21767097	0.049315
27	oar3_OAR›	21767529	-0.02974
27	oar3_OAR›	21771046	0.03574
27	oar3_OAR›	21776027	0.041199
27	oar3_OAR›	21776388	-0.03502
27	oar3_OAR›	21779455	-0.00738
27	oar3_OAR›	21780200	0.002533
27	oar3_OAR›	21784607	-0.03461
27	oar3_OAR›	21787343	0.011849
27	oar3_OAR›	21789221	0.011849
27	oar3_OAR›	21792207	0.020338
27	oar3_OAR›	21805708	-0.01672
27	oar3_OAR›	21805806	0.026763
27	oar3_OAR›	21805975	0.026763
27	oar3_OAR›	21806927	0.039248
27	oar3_OAR›	21815561	-0.02926
27	oar3_OAR›	21819014	-0.0049
27	oar3_OAR›	21822163	-0.0052
27	oar3_OAR›	21827908	0.020371
27	oar3_OAR›	21835461	0.00646
27	oar3_OAR›	21840057	-0.00958
27	oar3_OAR›	21858849	0.011643
27	oar3_OAR›	21859026	-0.00349
27	oar3_OAR›	21859416	-0.01226
27	oar3_OAR›	21870352	-0.01226
27	oar3_OAR›	21874231	-0.02348
27	oar3_OAR›	21874320	-0.03513
27	oar3_OAR›	21875161	-0.01731
27	oar3_OAR›	21875177	-0.01731
27	oar3_OAR›	21882364	0.026955
27	oar3_OAR›	21900253	0.042174
27	oar3_OAR›	21900485	0.068708
27	oar3_OAR›	21900845	0.030214

27	oar3_OAR›	21911057	0.007039
27	oar3_OAR›	21919016	0.041666
27	oar3_OAR›	21924535	-0.03858
27	oar3_OAR›	21930972	0.017152
27	oar3_OAR›	21933421	-0.03858
27	oar3_OAR›	21935402	-0.03858
27	oar3_OAR›	21936799	-0.03679
27	oar3_OAR›	21956173	-0.02223
27	oar3_OAR›	21984445	-0.03093
27	oar3_OAR›	22001626	-0.03093
27	oar3_OAR›	22004633	-0.03093
27	oar3_OAR›	22006624	0.009496
27	oar3_OAR›	22020886	-0.0008
27	oar3_OAR›	22021727	0.013496
27	oar3_OAR›	22059496	-0.0024
27	oar3_OAR›	22076096	-0.03337
27	oar3_OAR›	22078760	-0.01225
27	oar3_OAR›	22091102	-0.00836
27	oar3_OAR›	22094853	-0.03337
27	oar3_OAR›	22100520	-0.00117
27	oar3_OAR›	22100651	0.036843
27	oar3_OAR›	22117537	-0.03337
27	oar3_OAR›	22123086	-0.03337
27	oar3_OAR›	22126339	-0.00064
27	oar3_OAR›	22128622	-0.03287
27	oar3_OAR›	22137616	-0.03287
27	oar3_OAR›	22141164	-0.0264
27	oar3_OAR›	22149408	-0.0264
27	oar3_OAR›	22160989	-0.0264
27	oar3_OAR›	22167380	-0.02993
27	oar3_OAR›	22176689	-0.0264
27	oar3_OAR›	22182938	0.009985
27	oar3_OAR›	22189856	-0.02011
27	oar3_OAR›	22195581	-0.00671
27	oar3_OAR›	22198360	-0.03036
27	oar3_OAR›	22202003	-0.01962
27	oar3_OAR›	22207097	-0.0249
27	oar3_OAR›	22207175	-0.0249
27	oar3_OAR›	22215625	-0.03672
27	oar3_OAR›	22216236	-0.01284
27	oar3_OAR›	22242167	-0.01284
27	oar3_OAR›	22249914	-0.03661
27	oar3_OAR›	22259272	-0.02555
27	oar3_OAR›	22268270	-0.03142
27	oar3_OAR›	22275078	-0.02362
27	oar3_OAR›	22275214	-0.01569
27	oar3_OAR›	22278460	0.057126
27	oar3_OAR›	22279163	0.057126
27	oar3_OAR›	22280363	-0.01673
27	oar3_OAR›	22283257	0.030545
27	oar3_OAR›	22292865	0.007039
27	oar3_OAR›	22299960	-0.0354
27	oar3_OAR›	22302112	-0.00985
27	oar3_OAR›	22302172	0.028743

27	oar3_OAR›	22302346	-0.00985
27	oar3_OAR›	22311538	-0.0141
27	oar3_OAR›	22314341	-0.00884
27	oar3_OAR›	22315355	0.016767
27	oar3_OAR›	22318944	-0.01587
27	oar3_OAR›	22324501	0.120734
27	oar3_OAR›	22326838	-0.00326
27	oar3_OAR›	22332477	0.104285
27	oar3_OAR›	22332631	0.053295
27	oar3_OAR›	22340942	0.076117
27	oar3_OAR›	22342804	0.053691
27	oar3_OAR›	22349331	0.013395
27	oar3_OAR›	22353240	-0.02624
27	oar3_OAR›	22354786	-0.00913
27	oar3_OAR›	22354903	0.007823
27	oar3_OAR›	22358735	-0.00325
27	oar3_OAR›	22360207	-0.02092
27	oar3_OAR›	22369702	0.069982
27	oar3_OAR›	22370279	0.026771
27	oar3_OAR›	22376313	-0.0267
27	oar3_OAR›	22376512	-0.00597
27	oar3_OAR›	22381578	0.024935
27	oar3_OAR›	22381955	-0.03208
27	oar3_OAR›	22386842	-0.01539
27	oar3_OAR›	22388192	-0.01539
27	oar3_OAR›	22393083	0.019458
27	oar3_OAR›	22395458	-0.01978
27	oar3_OAR›	22403996	0.061637
27	oar3_OAR›	22404254	0.122418
27	oar3_OAR›	22405108	0.040066
27	oar3_OAR›	22405686	0.116401
27	oar3_OAR›	22410335	0.026583
27	oar3_OAR›	22416877	0.16925
27	oar3_OAR›	22419692	0.017085
27	oar3_OAR›	22421288	0.076873
27	oar3_OAR›	22432639	0.040066
27	oar3_OAR›	22432856	0.15482
27	oar3_OAR›	22435728	0.043051
27	oar3_OAR›	22450331	0.011164
27	oar3_OAR›	22450621	0.122193
27	oar3_OAR›	22460014	-0.02917
27	oar3_OAR›	22462057	0.017057
27	oar3_OAR›	22467762	-0.02037
27	oar3_OAR›	22476022	0.023689
27	oar3_OAR›	22480125	0.099745
27	oar3_OAR›	22483242	-0.01653
27	oar3_OAR›	22485786	0.050333
27	oar3_OAR›	22489068	-0.01031
27	oar3_OAR›	22493409	-0.01031
27	oar3_OAR›	22500960	-0.02604
27	oar3_OAR›	22505581	-0.02393
27	oar3_OAR›	22506151	-0.03167
27	oar3_OAR›	22510934	-0.01375
27	oar3_OAR›	22511736	-0.02802

27	oar3_OAR›	22519309	-0.00707
27	oar3_OAR›	22520382	-0.02604
27	oar3_OAR›	22526250	-0.00707
27	oar3_OAR›	22543363	0.010971
27	oar3_OAR›	22543958	0.010971
27	oar3_OAR›	22544342	0.010971
27	oar3_OAR›	22561029	0.010971
27	oar3_OAR›	22564835	0.006953
27	oar3_OAR›	22565445	0.006953
27	oar3_OAR›	22569196	0.014708
27	oar3_OAR›	22574882	-0.01028
27	oar3_OAR›	22577153	-0.0299
27	oar3_OAR›	22577278	0.043816
27	oar3_OAR›	22581050	0.006953
27	oar3_OAR›	22585342	0.046484
27	oar3_OAR›	22593388	-0.00673
27	oar3_OAR›	22594529	-0.01992
27	oar3_OAR›	22595932	-0.01242
27	oar3_OAR›	22596495	-0.01992
27	oar3_OAR›	22598344	-0.00864
27	oar3_OAR›	22615720	0.044611
27	oar3_OAR›	22619612	-0.01368
27	oar3_OAR›	22628295	0.027345
27	oar3_OAR›	22635891	-0.01614
27	oar3_OAR›	22639673	0.033773
27	oar3_OAR›	22640822	-0.00057
27	oar3_OAR›	22645996	0.033773
27	oar3_OAR›	22650415	-0.00815
27	oar3_OAR›	22656362	-0.02449
27	oar3_OAR›	22658134	0.009348
27	oar3_OAR›	22665420	-0.02381
27	oar3_OAR›	22668695	0.028704
27	oar3_OAR›	22670901	-0.02381
27	oar3_OAR›	22672130	0.001617
27	oar3_OAR›	22673685	-0.02381
27	oar3_OAR›	22685836	-0.01841
27	oar3_OAR›	22686034	-0.00842
27	oar3_OAR›	22688292	0.075154
27	oar3_OAR›	22696357	0.00481
27	oar3_OAR›	22699457	-0.0236
27	oar3_OAR›	22700359	-0.03099
27	oar3_OAR›	22705993	-0.00735
27	oar3_OAR›	22706319	-0.00735
27	oar3_OAR›	22708252	-0.02788
27	oar3_OAR›	22718796	0.028555
27	oar3_OAR›	22723163	0.072806
27	oar3_OAR›	22725548	0.024857
27	oar3_OAR›	22726574	0.074647
27	oar3_OAR›	22731742	0.119455
27	oar3_OAR›	22748861	-0.02593
27	oar3_OAR›	22750751	-0.02593
27	oar3_OAR›	22753873	-0.02593
27	oar3_OAR›	22763425	0.016958
27	oar3_OAR›	22763488	-0.02833

27	oar3_OAR〉	22775592	-0.01794
27	oar3_OAR〉	22776908	-0.02493
27	oar3_OAR〉	22778636	-0.01954
27	oar3_OAR〉	22779984	-0.02755
27	oar3_OAR〉	22780713	-0.03382
27	oar3_OAR〉	22787360	0.007039
27	oar3_OAR〉	22791320	-0.01128
27	oar3_OAR〉	22798038	-0.02349
27	oar3_OAR〉	22799227	-0.00613
27	oar3_OAR〉	22805031	-0.0107
27	oar3_OAR〉	22807601	0.027363
27	oar3_OAR〉	22812429	-0.01529
27	oar3_OAR〉	22814293	-0.03434
27	oar3_OAR〉	22817377	0.005819
27	oar3_OAR〉	22817473	0.005819
27	oar3_OAR〉	22828033	-0.00446
27	oar3_OAR〉	22829892	0.009791
27	oar3_OAR〉	22831187	0.005993
27	oar3_OAR〉	22836879	-0.03529
27	oar3_OAR〉	22839314	-0.03132
27	oar3_OAR〉	22843101	0.000239
27	oar3_OAR〉	22847534	0.037749
27	oar3_OAR〉	22850053	-0.01716
27	oar3_OAR〉	22853835	0.038109
27	oar3_OAR〉	22859251	-0.01628
27	oar3_OAR〉	22864687	-0.01379
27	oar3_OAR〉	22865060	-0.01069
27	oar3_OAR〉	22872956	0.017714
27	oar3_OAR〉	22876911	0.118398
27	oar3_OAR〉	22880181	-0.00814
27	oar3_OAR〉	22883967	-0.00066
27	oar3_OAR〉	22884237	0.007009
27	oar3_OAR〉	22886115	0.106555
27	oar3_OAR〉	22891316	-0.00996
27	oar3_OAR〉	22894441	0.016149
27	oar3_OAR〉	22902226	-0.00209
27	oar3_OAR〉	22905363	0.02905
27	oar3_OAR〉	22905416	0.003654
27	oar3_OAR〉	22906235	0.00481
27	oar3_OAR〉	22912595	0.02183
27	oar3_OAR〉	22913725	-0.00699
27	oar3_OAR〉	22916049	0.02715
27	oar3_OAR〉	22916102	-0.02154
27	oar3_OAR〉	22922546	-0.03267
27	oar3_OAR〉	22924542	-0.01144
27	oar3_OAR〉	22928704	-0.03267
27	oar3_OAR〉	22929652	-0.00996
27	oar3_OAR〉	22930123	-0.0385
27	oar3_OAR〉	22939705	0.044635
27	oar3_OAR〉	22947144	0.065512
27	oar3_OAR〉	22947645	-0.01794
27	oar3_OAR〉	22948933	-0.00765
27	oar3_OAR〉	22950269	-0.02235
27	oar3_OAR〉	22955100	0.026159

27	oar3_OAR›	22957134	-0.02345
27	oar3_OAR›	22957180	0.036972
27	oar3_OAR›	22960691	0.026583
27	oar3_OAR›	22967783	-0.01456
27	oar3_OAR›	22967868	-0.01456
27	oar3_OAR›	22967881	0.005378
27	oar3_OAR›	22972300	0.065755
27	oar3_OAR›	22976499	0.025394
27	oar3_OAR›	22976698	-0.03382
27	oar3_OAR›	22981232	0.00239
27	oar3_OAR›	22983915	0.013569
27	oar3_OAR›	22993377	-0.02788
27	oar3_OAR›	22993720	0.075535
27	oar3_OAR›	22994969	0.120825
27	oar3_OAR›	22999078	-0.0298
27	oar3_OAR›	23003458	-0.01879
27	oar3_OAR›	23004896	-0.02597
27	oar3_OAR›	23005037	-0.01074
27	oar3_OAR›	23006832	-0.02529
27	oar3_OAR›	23012396	-0.0191
27	oar3_OAR›	23019171	0.044724
27	oar3_OAR›	23019752	-0.02529
27	oar3_OAR›	23020121	-0.0294
27	oar3_OAR›	23027586	0.037003
27	oar3_OAR›	23035182	-0.00883
27	oar3_OAR›	23040902	0.055969
27	oar3_OAR›	23051919	0.078432
27	oar3_OAR›	23054512	0.147015
27	oar3_OAR›	23058228	-0.02546
27	oar3_OAR›	23058894	0.021224
27	oar3_OAR›	23061760	0.095763
27	oar3_OAR›	23063016	0.134073
27	oar3_OAR›	23080848	0.10915
27	oar3_OAR›	23084144	-0.03073
27	oar3_OAR›	23084524	-0.03483
27	oar3_OAR›	23085344	0.0075
27	oar3_OAR›	23088592	0.009692
27	oar3_OAR›	23094134	-0.03073
27	oar3_OAR›	23095954	-0.03073
27	oar3_OAR›	23096176	-0.03073
27	oar3_OAR›	23096426	-0.03073
27	oar3_OAR›	23102946	0.010961
27	oar3_OAR›	23108138	0.05094
27	oar3_OAR›	23108488	0.059318
27	oar3_OAR›	23109048	-0.00465
27	oar3_OAR›	23109138	-0.03858
27	oar3_OAR›	23137075	0.08518
27	oar3_OAR›	23141567	0.056773
27	oar3_OAR›	23141721	0.099668
27	oar3_OAR›	23157788	0.085614
27	oar3_OAR›	23162594	0.157209
27	oar3_OAR›	23168568	0.099555
27	oar3_OAR›	23175544	0.096439
27	oar3_OAR›	23187440	-0.00578

27	oar3_OAR›	23195766	-0.00735
27	oar3_OAR›	23198178	-0.00578
27	oar3_OAR›	23205951	0.079876
27	oar3_OAR›	23206181	0.010961
27	oar3_OAR›	23208400	0.032125
27	oar3_OAR›	23208448	0.135487
27	oar3_OAR›	23213789	0.135487
27	oar3_OAR›	23213915	0.025112
27	oar3_OAR›	23218982	-0.00735
27	oar3_OAR›	23221394	0.025112
27	oar3_OAR›	23222465	0.025112
27	oar3_OAR›	23227346	0.013065
27	oar3_OAR›	23228016	-0.00735
27	oar3_OAR›	23233776	0.02652
27	oar3_OAR›	23242400	0.044086
27	oar3_OAR›	23242651	0.044086
27	oar3_OAR›	23246272	0.000889
27	oar3_OAR›	23248509	0.19078
27	oar3_OAR›	23254345	0.072163
27	oar3_OAR›	23255966	0.038847
27	oar3_OAR›	23258898	0.242377
27	oar3_OAR›	23259151	0.242377
27	oar3_OAR›	23260977	-0.02162
27	oar3_OAR›	23268956	0.22556
27	oar3_OAR›	23272272	0.181891
27	oar3_OAR›	23276701	0.095554
27	oar3_OAR›	23286811	-0.00788
27	oar3_OAR›	23297371	-0.01069
27	oar3_OAR›	23308991	-0.0155
27	oar3_OAR›	23326128	-0.0293
27	oar3_OAR›	23366583	-0.04195
27	oar3_OAR›	23372932	-0.01567
27	oar3_OAR›	23391379	-0.01243
27	oar3_OAR›	23415540	-0.01096
27	oar3_OAR›	23415814	0.00952
27	oar3_OAR›	23416781	-0.01223
27	oar3_OAR›	23433375	-0.01096
27	oar3_OAR›	23446403	-0.02645
27	oar3_OAR›	23449559	0.007994
27	oar3_OAR›	23458143	0.029806
27	oar3_OAR›	23470239	-0.00332
27	oar3_OAR›	23472221	-0.02223
27	oar3_OAR›	23472787	-0.02223
27	oar3_OAR›	23479977	-0.01212
27	oar3_OAR›	23482805	-0.00229
27	oar3_OAR›	23483396	-0.00606
27	oar3_OAR›	23488686	-0.01456
27	oar3_OAR›	23493727	0.019615
27	oar3_OAR›	23495325	0.02833
27	oar3_OAR›	23495461	-0.02575
27	oar3_OAR›	23505413	0.023269
27	oar3_OAR›	23511893	0.071521
27	oar3_OAR›	23514146	0.048781
27	oar3_OAR›	23514406	0.226938

27	oar3_OAR›	23527073	0.067788
27	oar3_OAR›	23532311	0.090065
27	oar3_OAR›	23532675	0.064031
27	oar3_OAR›	23535754	0.044678
27	oar3_OAR›	23548243	-0.00232
27	oar3_OAR›	23548427	-0.00463
27	oar3_OAR›	23554176	-0.00232
27	oar3_OAR›	23557848	-0.01631
27	oar3_OAR›	23559183	-0.00463
27	oar3_OAR›	23568435	-0.00917
27	oar3_OAR›	23574968	-0.03752
27	oar3_OAR›	23577117	0.014586
27	oar3_OAR›	23581144	-0.01606
27	oar3_OAR›	23586431	-0.02358
27	oar3_OAR›	23589286	-0.01349
27	oar3_OAR›	23593816	0.012479
27	oar3_OAR›	23596668	0.009046
27	oar3_OAR›	23601384	-0.02358
27	oar3_OAR›	23601576	-0.01069
27	oar3_OAR›	23601695	-0.02358
27	oar3_OAR›	23610512	0.018725
27	oar3_OAR›	23611728	-0.0036
27	oar3_OAR›	23612683	-0.02556
27	oar3_OAR›	23633955	0.016062
27	oar3_OAR›	23641170	-0.01236
27	oar3_OAR›	23644941	0.040246
27	oar3_OAR›	23652081	-0.01069
27	oar3_OAR›	23655679	0.083467
27	oar3_OAR›	23657339	0.151226
27	oar3_OAR›	23660148	0.026444
27	oar3_OAR›	23660190	0.001648
27	oar3_OAR›	23662501	-0.02351
27	oar3_OAR›	23667025	0.156748
27	oar3_OAR›	23667701	0.107121
27	oar3_OAR›	23672660	0.156748
27	oar3_OAR›	23683651	-0.01069
27	oar3_OAR›	23685014	0.142886
27	oar3_OAR›	23687892	0.162217
27	oar3_OAR›	23688619	0.126876
27	oar3_OAR›	23694729	0.028803
27	oar3_OAR›	23695731	0.095464
27	oar3_OAR›	23699882	0.035124
27	oar3_OAR›	23700131	0.000893
27	oar3_OAR›	23700311	-0.01069
27	oar3_OAR›	23705311	-0.01069
27	oar3_OAR›	23710846	0.003587
27	oar3_OAR›	23716192	0.236165
27	oar3_OAR›	23719334	0.07966
27	oar3_OAR›	23720884	-0.02744
27	oar3_OAR›	23727491	-0.02744
27	oar3_OAR›	23730486	0.189911
27	oar3_OAR›	23733383	0.241166
27	oar3_OAR›	23733529	0.224545
27	oar3_OAR›	23740952	0.086168

27	oar3_OAR›	23744290	0.109575
27	oar3_OAR›	23746364	-0.02159
27	oar3_OAR›	23750817	0.160937
27	oar3_OAR›	23757525	0.142165
27	oar3_OAR›	23759215	0.102559
27	oar3_OAR›	23760560	0.244627
27	oar3_OAR›	23778197	0.031957
27	oar3_OAR›	23781217	0.031957
27	oar3_OAR›	23786093	0.104328
27	oar3_OAR›	23786557	-0.03688
27	oar3_OAR›	23786689	-0.00982
27	oar3_OAR›	23792999	0.080841
27	oar3_OAR›	23797368	0.053442
27	oar3_OAR›	23799300	0.119605
27	oar3_OAR›	23802028	-0.0107
27	oar3_OAR›	23805957	0.019413
27	oar3_OAR›	23810166	0.007338
27	oar3_OAR›	23813131	0.005104
27	oar3_OAR›	23814651	0.219944
27	oar3_OAR›	23821137	0.219944
27	oar3_OAR›	23821553	0.007665
27	oar3_OAR›	23821758	0.068943
27	oar3_OAR›	23830878	-0.02805
27	oar3_OAR›	23835290	-0.0025
27	oar3_OAR›	23842181	0.245815
27	oar3_OAR›	23852030	-0.0065
27	oar3_OAR›	23854797	0.032945
27	oar3_OAR›	23861491	0.056909
27	oar3_OAR›	23861587	0.210378
27	oar3_OAR›	23876866	-0.02645
27	oar3_OAR›	23881469	-0.02561
27	oar3_OAR›	23896905	0.018399
27	oar3_OAR›	23900561	-0.00668
27	oar3_OAR›	23902464	0.086728
27	oar3_OAR›	23902496	0.086728
27	oar3_OAR›	23908651	0.016847
27	oar3_OAR›	23908810	0.068225
27	oar3_OAR›	23915265	0.022167
27	oar3_OAR›	23916297	-0.01879
27	oar3_OAR›	23916450	0.032787
27	oar3_OAR›	23922619	-0.00606
27	oar3_OAR›	23946475	-0.00427
27	oar3_OAR›	23946597	0.01345
27	oar3_OAR›	23949046	0.028467
27	oar3_OAR›	23954275	-0.00942
27	oar3_OAR›	23959237	0.003466
27	oar3_OAR›	23959532	-0.00404
27	oar3_OAR›	23959642	-0.00698
27	oar3_OAR›	23959767	-0.01202
27	oar3_OAR›	23969745	-0.00793
27	oar3_OAR›	23971349	-0.00606
27	oar3_OAR›	23973877	0.038843
27	oar3_OAR›	23984864	-0.0206
27	oar3_OAR›	23993392	-0.01805

27	oar3_OAR›	23995247	-0.01473
27	oar3_OAR›	24005996	-0.02586
27	oar3_OAR›	24007690	0.084285
27	oar3_OAR›	24008640	0.038304
27	oar3_OAR›	24023912	0.060033
27	oar3_OAR›	24029252	-0.01738
27	oar3_OAR›	24032112	-0.02515
27	oar3_OAR›	24038790	-0.03037
27	oar3_OAR›	24043933	-0.03252
27	oar3_OAR›	24047579	-0.00423
27	oar3_OAR›	24050836	0.077702
27	oar3_OAR›	24054144	0.036229
27	oar3_OAR›	24058747	0.054952
27	oar3_OAR›	24059365	-0.00423
27	oar3_OAR›	24067342	0.027215
27	oar3_OAR›	24069976	0.015084
27	oar3_OAR›	24074097	-0.01002
27	oar3_OAR›	24074463	0.091693
27	oar3_OAR›	24074519	-0.01957
27	oar3_OAR›	24080611	-0.02681
27	oar3_OAR›	24092697	-0.02681
27	oar3_OAR›	24094584	-0.02044
27	oar3_OAR›	24099755	-0.00462
27	oar3_OAR›	24106905	-0.00606
27	oar3_OAR›	24112332	0.021636
27	oar3_OAR›	24112389	-0.00931
27	oar3_OAR›	24112894	0.041837
27	oar3_OAR›	24129536	-0.00606
27	oar3_OAR›	24129673	0.066908
27	oar3_OAR›	24130676	-0.0246
27	oar3_OAR›	24133065	0.066908
27	oar3_OAR›	24135736	-0.02845
27	oar3_OAR›	24141090	0.101779
27	oar3_OAR›	24141159	0.07031
27	oar3_OAR›	24141553	-0.00796
27	oar3_OAR›	24141830	-0.028
27	oar3_OAR›	24148926	0.01309
27	oar3_OAR›	24152690	-0.022
27	oar3_OAR›	24153126	-0.022
27	oar3_OAR›	24159200	0.01309
27	oar3_OAR›	24159969	-0.02666
27	oar3_OAR›	24163899	0.00172
27	oar3_OAR›	24164779	0.078398
27	oar3_OAR›	24181225	0.025713
27	oar3_OAR›	24181389	-0.00436
27	oar3_OAR›	24186961	0.025713
27	oar3_OAR›	24191419	0.02221
27	oar3_OAR›	24192712	0.029985
27	oar3_OAR›	24192845	-0.02137
27	oar3_OAR›	24198748	0.025713
27	oar3_OAR›	24204693	-0.00575
27	oar3_OAR›	24207238	0.07572
27	oar3_OAR›	24209399	0.019513
27	oar3_OAR›	24210588	-0.00756

27	oar3_OAR〉	24219741	0.057053
27	oar3_OAR〉	24220281	-0.00337
27	oar3_OAR〉	24222870	-0.02462
27	oar3_OAR〉	24234127	-0.02597
27	oar3_OAR〉	24244908	0.043851
27	oar3_OAR〉	24247830	-0.00768
27	oar3_OAR〉	24253966	0.043851
27	oar3_OAR〉	24261777	0.074839
27	oar3_OAR〉	24263767	-0.00795
27	oar3_OAR〉	24266722	0.016589
27	oar3_OAR〉	24267312	-0.02597
27	oar3_OAR〉	24272556	0.103251
27	oar3_OAR〉	24275027	0.060376
27	oar3_OAR〉	24278323	0.100105
27	oar3_OAR〉	24278698	-0.0179
27	oar3_OAR〉	24288589	-0.01069
27	oar3_OAR〉	24296110	0.103251
27	oar3_OAR〉	24299235	-0.01069
27	oar3_OAR〉	24309468	-0.00381
27	oar3_OAR〉	24310834	-0.02265
27	oar3_OAR〉	24322548	0.068407
27	oar3_OAR〉	24323952	0.068407
27	oar3_OAR〉	24324093	-0.00381
27	oar3_OAR〉	24330962	0.000214
27	oar3_OAR〉	24333131	0.012193
27	oar3_OAR〉	24343491	0.000214
27	oar3_OAR〉	24349560	-0.01537
27	oar3_OAR〉	24352204	0.000214
27	oar3_OAR〉	24360870	0.031762
27	oar3_OAR〉	24366667	0.000889
27	oar3_OAR〉	24367652	0.015455
27	oar3_OAR〉	24368854	0.004113
27	oar3_OAR〉	24388946	-0.01061
27	oar3_OAR〉	24392245	0.009617
27	oar3_OAR〉	24401728	-0.02768
27	oar3_OAR〉	24450730	-0.00146
27	oar3_OAR〉	24459102	-0.03213
27	oar3_OAR〉	24467431	-0.01737
27	oar3_OAR〉	24467659	-0.00382
27	oar3_OAR〉	24467788	-0.02755
27	oar3_OAR〉	24472518	-0.01124
27	oar3_OAR〉	24475950	-0.00928
27	oar3_OAR〉	24485566	-0.00693
27	oar3_OAR〉	24485899	-0.00693
27	oar3_OAR〉	24487847	-0.01133
27	oar3_OAR〉	24492190	-0.01165
27	oar3_OAR〉	24497744	-0.01165
27	oar3_OAR〉	24501842	-0.01587
27	oar3_OAR〉	24516028	0.003409
27	oar3_OAR〉	24516382	-0.00274
27	oar3_OAR〉	24519939	0.003055
27	oar3_OAR〉	24524213	-0.0197
27	oar3_OAR〉	24524371	0.000889
27	oar3_OAR〉	24530783	-0.02097

27	oar3_OAR>	24532410	-0.0076
27	oar3_OAR>	24534849	-0.01252
27	oar3_OAR>	24541342	-0.01252
27	oar3_OAR>	24548962	-0.00274
27	oar3_OAR>	24548997	0.000889
27	oar3_OAR>	24554390	0.021558
27	oar3_OAR>	24554824	-0.0076
27	oar3_OAR>	24563192	-0.0054
27	oar3_OAR>	24569819	-0.00663
27	oar3_OAR>	24576026	-0.02797
27	oar3_OAR>	24579158	-0.02797
27	oar3_OAR>	24584433	-0.02797
27	oar3_OAR>	24590267	0.011966
27	oar3_OAR>	24602887	-0.015
27	oar3_OAR>	24604667	0.029527
27	oar3_OAR>	24612470	0.029527
27	oar3_OAR>	24612617	-0.015
27	oar3_OAR>	24619895	0.050381
27	oar3_OAR>	24625123	0.029527
27	oar3_OAR>	24630524	0.029527
27	oar3_OAR>	24659871	-0.02009
27	oar3_OAR>	24660105	0.000889
27	oar3_OAR>	24667118	0.011871
27	oar3_OAR>	24677040	-0.02009
27	oar3_OAR>	24681845	0.01084
27	oar3_OAR>	24687794	0.017104
27	oar3_OAR>	24691992	0.048539
27	oar3_OAR>	24701341	0.035383
27	oar3_OAR>	24701494	0.058238
27	oar3_OAR>	24710932	0.017714
27	oar3_OAR>	24729286	0.011802
27	oar3_OAR>	24729352	-0.01752
27	oar3_OAR>	24731646	-0.01752
27	oar3_OAR>	24734832	-0.00024
27	oar3_OAR>	24740949	0.001086
27	oar3_OAR>	24747503	-0.0085
27	oar3_OAR>	24752203	0.008723
27	oar3_OAR>	24760645	-0.01349
27	oar3_OAR>	24773741	-0.02709
27	oar3_OAR>	24789382	-0.02709
27	oar3_OAR>	24791396	-0.03047
27	oar3_OAR>	24820496	-0.01752
27	oar3_OAR>	24821218	-0.01128
27	oar3_OAR>	24838662	0.028803
27	oar3_OAR>	24838774	0.028803
27	oar3_OAR>	24839947	-0.02205
27	oar3_OAR>	24843653	NA
27	oar3_OAR>	24847552	-0.02153
27	oar3_OAR>	24870292	-0.00561
27	oar3_OAR>	24870601	-0.0178
27	oar3_OAR>	24870667	-0.00561
27	oar3_OAR>	24885910	-0.03531
27	oar3_OAR>	24887037	-0.00195
27	oar3_OAR>	24893273	0.019123

27	oar3_OAR›	24895291	0.019123
27	oar3_OAR›	24899141	-0.01795
27	oar3_OAR›	24904181	0.028277
27	oar3_OAR›	24904810	0.059098
27	oar3_OAR›	24911642	-0.00941
27	oar3_OAR›	24911971	0.034159
27	oar3_OAR›	24912951	0.080493
27	oar3_OAR›	24913041	-0.04195
27	oar3_OAR›	24915713	0.092287
27	oar3_OAR›	24918017	0.003762
27	oar3_OAR›	24925870	0.026238
27	oar3_OAR›	24926029	0.127971
27	oar3_OAR›	24926818	0.051893
27	oar3_OAR›	24938250	-0.02068
27	oar3_OAR›	24946614	0.002754
27	oar3_OAR›	24952505	0.016173
27	oar3_OAR›	24952657	-0.00857
27	oar3_OAR›	24963248	0.028778
27	oar3_OAR›	24965586	0.055489
27	oar3_OAR›	24974720	0.046329
27	oar3_OAR›	24974848	0.013579
27	oar3_OAR›	24975892	-0.01801
27	oar3_OAR›	24977162	-0.02339
27	oar3_OAR›	24983294	0.000363
27	oar3_OAR›	24986138	0.021796
27	oar3_OAR›	24987034	-0.02159
27	oar3_OAR›	24988770	0.131261
27	oar3_OAR›	24992548	-0.02242
27	oar3_OAR›	24997856	-0.02242
27	oar3_OAR›	25002627	0.041093
27	oar3_OAR›	25002703	0.041093
27	oar3_OAR›	25002909	0.041093
27	oar3_OAR›	25004442	-0.02575
27	oar3_OAR›	25013523	0.049865
27	oar3_OAR›	25030621	0.16797
27	oar3_OAR›	25033805	0.16797
27	oar3_OAR›	25034311	0.069057
27	oar3_OAR›	25041700	0.032365
27	oar3_OAR›	25043425	0.037425
27	oar3_OAR›	25045353	0.14742
27	oar3_OAR›	25050185	0.116965
27	oar3_OAR›	25065067	0.170958
27	oar3_OAR›	25066131	0.016379
27	oar3_OAR›	25068448	0.068311
27	oar3_OAR›	25068516	0.068311
27	oar3_OAR›	25073005	0.048426
27	oar3_OAR›	25078354	0.011115
27	oar3_OAR›	25079315	0.064036
27	oar3_OAR›	25080781	0.010956
27	oar3_OAR›	25097842	0.013268
27	oar3_OAR›	25103567	0.013268
27	oar3_OAR›	25109479	0.01457
27	oar3_OAR›	25121502	0.210162
27	oar3_OAR›	25128024	0.177127

27	oar3_OAR›	25129467	0.06659
27	oar3_OAR›	25134765	-0.02575
27	oar3_OAR›	25137513	0.05072
27	oar3_OAR›	25138483	0.177127
27	oar3_OAR›	25139900	0.018772
27	oar3_OAR›	25145178	-0.01356
27	oar3_OAR›	25145424	0.034492
27	oar3_OAR›	25146086	0.063149
27	oar3_OAR›	25150479	0.177126
27	oar3_OAR›	25154593	0.128756
27	oar3_OAR›	25156955	0.002668
27	oar3_OAR›	25161770	0.051649
27	oar3_OAR›	25163189	0.066176
27	oar3_OAR›	25163342	0.078398
27	oar3_OAR›	25168973	-0.01851
27	oar3_OAR›	25171875	0.048956
27	oar3_OAR›	25175151	0.16401
27	oar3_OAR›	25175431	0.16401
27	oar3_OAR›	25185551	8.73E-05
27	oar3_OAR›	25199952	0.018564
27	oar3_OAR›	25203080	0.076926
27	oar3_OAR›	25207239	0.035888
27	oar3_OAR›	25208928	-0.00434
27	oar3_OAR›	25213590	0.01645
27	oar3_OAR›	25226617	0.01645
27	oar3_OAR›	25236324	-0.02568
27	oar3_OAR›	25238395	0.005457
27	oar3_OAR›	25251682	-0.00995
27	oar3_OAR›	25264204	-0.02693
27	oar3_OAR›	25283828	0.009029
27	oar3_OAR›	25285658	0.017015
27	oar3_OAR›	25305456	0.025089
27	oar3_OAR›	25323008	0.023763
27	oar3_OAR›	25323219	0.023763
27	oar3_OAR›	25331818	-0.0284
27	oar3_OAR›	25332029	-0.0284
27	oar3_OAR›	25344124	-0.0177
27	oar3_OAR›	25348851	0.010721
27	oar3_OAR›	25349314	-0.01567
27	oar3_OAR›	25353810	-0.01089
27	oar3_OAR›	25359439	0.002942
27	oar3_OAR›	25364065	-0.00638
27	oar3_OAR›	25370441	0.009746
27	oar3_OAR›	25388079	-0.028
27	oar3_OAR›	25388314	0.051958
27	oar3_OAR›	25391934	-0.02453
27	oar3_OAR›	25395824	-0.02453
27	oar3_OAR›	25398898	0.101608
27	oar3_OAR›	25401232	0.021823
27	oar3_OAR›	25402252	-0.00396
27	oar3_OAR›	25411240	-0.03031
27	oar3_OAR›	25414717	0.078179
27	oar3_OAR›	25415734	0.104574
27	oar3_OAR›	25420218	-0.03147

27	oar3_OAR〉	25422185	0.119594
27	oar3_OAR〉	25427390	-0.03369
27	oar3_OAR〉	25427754	-0.0085
27	oar3_OAR〉	25444265	-0.01251
27	oar3_OAR〉	25450097	0.020939
27	oar3_OAR〉	25454907	0.007893
27	oar3_OAR〉	25457236	0.053964
27	oar3_OAR〉	25461150	-0.0248
27	oar3_OAR〉	25468291	0.006682
27	oar3_OAR〉	25471618	0.062981
27	oar3_OAR〉	25475380	0.018601
27	oar3_OAR〉	25483347	-0.02473
27	oar3_OAR〉	25483643	-0.02473
27	oar3_OAR〉	25489870	-0.00811
27	oar3_OAR〉	25495462	-0.02883
27	oar3_OAR〉	25496358	0.003071
27	oar3_OAR〉	25496477	-0.02515
27	oar3_OAR〉	25501464	0.042058
27	oar3_OAR〉	25507839	0.003071
27	oar3_OAR〉	25513356	0.029155
27	oar3_OAR〉	25518655	0.010842
27	oar3_OAR〉	25518811	-0.00161
27	oar3_OAR〉	25524874	0.003337
27	oar3_OAR〉	25536198	-0.02505
27	oar3_OAR〉	25551111	0.007039
27	oar3_OAR〉	25552834	0.007039
27	oar3_OAR〉	25556916	0.007039
27	oar3_OAR〉	25560795	0.006638
27	oar3_OAR〉	25564218	0.007039
27	oar3_OAR〉	25567328	-0.02555
27	oar3_OAR〉	25575776	-0.01199
27	oar3_OAR〉	25579792	0.009692
27	oar3_OAR〉	25579876	-0.0057
27	oar3_OAR〉	25587762	-0.03111
27	oar3_OAR〉	25588930	-0.01758
27	oar3_OAR〉	25589633	-0.01046
27	oar3_OAR〉	25592808	0.018271
27	oar3_OAR〉	25599546	0.016364
27	oar3_OAR〉	25603594	-0.03117
27	oar3_OAR〉	25605377	0.010835
27	oar3_OAR〉	25610997	-0.02398
27	oar3_OAR〉	25611277	-0.02398
27	oar3_OAR〉	25612037	-0.02091
27	oar3_OAR〉	25613143	-0.02398
27	oar3_OAR〉	25616486	-0.0234
27	oar3_OAR〉	25622275	-0.03439
27	oar3_OAR〉	25627249	-0.03549
27	oar3_OAR〉	25635699	0.022432
27	oar3_OAR〉	25647052	0.11116
27	oar3_OAR〉	25656225	-0.01844
27	oar3_OAR〉	25657420	-0.01844
27	oar3_OAR〉	25659180	-0.01844
27	oar3_OAR〉	25665879	0.038968
27	oar3_OAR〉	25674953	-0.01257

27	oar3_OAR〉	25676621	-0.0196
27	oar3_OAR〉	25682606	-0.02077
27	oar3_OAR〉	25684655	0.022582
27	oar3_OAR〉	25686437	0.104229
27	oar3_OAR〉	25691402	0.090034
27	oar3_OAR〉	25693397	0.122726
27	oar3_OAR〉	25700838	0.022582
27	oar3_OAR〉	25705761	0.124569
27	oar3_OAR〉	25711165	0.146922
27	oar3_OAR〉	25711272	0.010288
27	oar3_OAR〉	25717267	0.047556
27	oar3_OAR〉	25719682	-0.02177
27	oar3_OAR〉	25722618	0.005053
27	oar3_OAR〉	25724034	-0.01255
27	oar3_OAR〉	25727040	0.01815
27	oar3_OAR〉	25730043	-0.01527
27	oar3_OAR〉	25730468	-0.00726
27	oar3_OAR〉	25736277	-0.03172
27	oar3_OAR〉	25736312	-0.03172
27	oar3_OAR〉	25737607	0.000415
27	oar3_OAR〉	25745331	-0.03117
27	oar3_OAR〉	25746632	0.082521
27	oar3_OAR〉	25754051	0.100807
27	oar3_OAR〉	25760049	0.100807
27	oar3_OAR〉	25760400	0.100807
27	oar3_OAR〉	25761200	0.100807
27	oar3_OAR〉	25766642	0.066207
27	oar3_OAR〉	25771154	0.00219
27	oar3_OAR〉	25771625	0.044246
27	oar3_OAR〉	25773942	0.131472
27	oar3_OAR〉	25774282	0.155549
27	oar3_OAR〉	25780295	-0.00195
27	oar3_OAR〉	25781896	0.021533
27	oar3_OAR〉	25786548	0.083648
27	oar3_OAR〉	25786590	0.004461
27	oar3_OAR〉	25787927	0.15886
27	oar3_OAR〉	25799202	-0.01731
27	oar3_OAR〉	25803542	0.006864
27	oar3_OAR〉	25805619	0.108492
27	oar3_OAR〉	25806536	-0.00524
27	oar3_OAR〉	25813220	-0.02662
27	oar3_OAR〉	25814108	0.01868
27	oar3_OAR〉	25817336	-0.01753
27	oar3_OAR〉	25823350	-0.00524
27	oar3_OAR〉	25824091	0.092065
27	oar3_OAR〉	25833969	-0.02774
27	oar3_OAR〉	25834207	-0.0085
27	oar3_OAR〉	25834291	-0.0337
27	oar3_OAR〉	25834499	-0.02774
27	oar3_OAR〉	25840632	0.068285
27	oar3_OAR〉	25842676	-0.00545
27	oar3_OAR〉	25845180	-0.03375
27	oar3_OAR〉	25846745	-0.01437
27	oar3_OAR〉	25857414	0.012126

27	oar3_OAR>	25857524	0.011083
27	oar3_OAR>	25860306	0.01736
27	oar3_OAR>	25867520	0.011365
27	oar3_OAR>	25867753	0.110469
27	oar3_OAR>	25867926	-0.03679
27	oar3_OAR>	25869059	0.137721
27	oar3_OAR>	25878876	0.110469
27	oar3_OAR>	25879266	0.058453
27	oar3_OAR>	25889904	-0.03542
27	oar3_OAR>	25892303	0.110469
27	oar3_OAR>	25893424	0.065275
27	oar3_OAR>	25895016	-0.01165
27	oar3_OAR>	25895431	0.104464
27	oar3_OAR>	25902531	0.015023
27	oar3_OAR>	25906925	0.028942
27	oar3_OAR>	25907136	0.028942
27	oar3_OAR>	25907473	0.028942
27	oar3_OAR>	25917342	0.044425
27	oar3_OAR>	25921079	-0.01717
27	oar3_OAR>	25922561	-0.02789
27	oar3_OAR>	25927492	-0.03189
27	oar3_OAR>	25932179	-0.01894
27	oar3_OAR>	25937187	-0.02174
27	oar3_OAR>	25937643	-0.02604
27	oar3_OAR>	25942190	-0.03189
27	oar3_OAR>	25943266	-0.03417
27	oar3_OAR>	25946197	-0.01574
27	oar3_OAR>	25951583	0.008378
27	oar3_OAR>	25954077	-0.01484
27	oar3_OAR>	25957036	-0.01884
27	oar3_OAR>	25965019	-0.03062
27	oar3_OAR>	25965239	0.044365
27	oar3_OAR>	25965280	-0.00681
27	oar3_OAR>	25969250	0.003829
27	oar3_OAR>	25970654	0.00613
27	oar3_OAR>	25972906	0.042972
27	oar3_OAR>	25976767	-0.01789
27	oar3_OAR>	25987125	0.026383
27	oar3_OAR>	25987703	0.036878
27	oar3_OAR>	25991416	-0.02609
27	oar3_OAR>	25994293	-0.01202
27	oar3_OAR>	26000691	-0.02721
27	oar3_OAR>	26001573	-0.01854
27	oar3_OAR>	26008918	-0.022
27	oar3_OAR>	26009298	-0.022
27	oar3_OAR>	26017743	-0.02193
27	oar3_OAR>	26019565	-0.02524
27	oar3_OAR>	26021041	-0.02524
27	oar3_OAR>	26024940	-0.01546
27	oar3_OAR>	26028277	0.018084
27	oar3_OAR>	26031442	-0.0009
27	oar3_OAR>	26031814	-0.0364
27	oar3_OAR>	26032694	-0.00248
27	oar3_OAR>	26037184	-0.03739

27	oar3_OAR)	26041850	-0.01851
27	oar3_OAR)	26051381	-0.0325
27	oar3_OAR)	26055537	-0.00868
27	oar3_OAR)	26060783	0.177033
27	oar3_OAR)	26063523	-0.03062
27	oar3_OAR)	26069967	0.015023
27	oar3_OAR)	26071681	0.093614
27	oar3_OAR)	26074454	0.045744
27	oar3_OAR)	26078999	0.015324
27	oar3_OAR)	26079733	0.032258
27	oar3_OAR)	26082234	-0.01451
27	oar3_OAR)	26089530	-0.02926
27	oar3_OAR)	26093957	0.103129
27	oar3_OAR)	26094661	-0.00522
27	oar3_OAR)	26096240	0.137631
27	oar3_OAR)	26099888	-0.01834
27	oar3_OAR)	26115894	0.190196
27	oar3_OAR)	26117422	0.033946
27	oar3_OAR)	26120239	-0.03036
27	oar3_OAR)	26124289	-0.02926
27	oar3_OAR)	26129586	0.00481
27	oar3_OAR)	26130022	0.055289
27	oar3_OAR)	26130627	0.024051
27	oar3_OAR)	26131136	-0.00952
27	oar3_OAR)	26140243	0.011749
27	oar3_OAR)	26140808	0.117817
27	oar3_OAR)	26146123	-0.03869
27	oar3_OAR)	26146456	0.097661
27	oar3_OAR)	26153910	0.119621
27	oar3_OAR)	26161281	0.078974
27	oar3_OAR)	26162101	########
27	oar3_OAR)	26175627	0.024583
27	oar3_OAR)	26178728	-0.01926
27	oar3_OAR)	26180248	-0.02362
27	oar3_OAR)	26180983	0.007201
27	oar3_OAR)	26185460	0.112717
27	oar3_OAR)	26186561	0.042344
27	oar3_OAR)	26193713	0.079498
27	oar3_OAR)	26195350	0.037014
27	oar3_OAR)	26200481	0.100633
27	oar3_OAR)	26207140	0.090116
27	oar3_OAR)	26207630	0.06433
27	oar3_OAR)	26210516	0.080619
27	oar3_OAR)	26212997	0.053501
27	oar3_OAR)	26221552	-0.01667
27	oar3_OAR)	26222086	0.014167
27	oar3_OAR)	26234256	0.037113
27	oar3_OAR)	26234680	0.000889
27	oar3_OAR)	26242143	0.002325
27	oar3_OAR)	26242825	-0.01667
27	oar3_OAR)	26247079	-0.01639
27	oar3_OAR)	26253686	-0.01667
27	oar3_OAR)	26257148	-0.02152
27	oar3_OAR)	26261520	-0.02413

27	oar3_OAR〉	26263316	-0.01021
27	oar3_OAR〉	26265082	0.035837
27	oar3_OAR〉	26269852	0.007039
27	oar3_OAR〉	26273841	0.074204
27	oar3_OAR〉	26278750	-0.01069
27	oar3_OAR〉	26288116	-0.01252
27	oar3_OAR〉	26298605	0.012328
27	oar3_OAR〉	26301398	-0.00096
27	oar3_OAR〉	26304045	0.031875
27	oar3_OAR〉	26310894	-0.00096
27	oar3_OAR〉	26311329	0.058441
27	oar3_OAR〉	26315889	0.02383
27	oar3_OAR〉	26319809	0.040893
27	oar3_OAR〉	26325084	-0.0027
27	oar3_OAR〉	26326905	-0.01461
27	oar3_OAR〉	26338923	-0.01859
27	oar3_OAR〉	26338960	0.044357
27	oar3_OAR〉	26345897	-0.00712
27	oar3_OAR〉	26351411	-0.02797
27	oar3_OAR〉	26351530	0.027046
27	oar3_OAR〉	26351668	0.031894
27	oar3_OAR〉	26354988	0.014944
27	oar3_OAR〉	26356685	-0.02662
27	oar3_OAR〉	26362745	-0.02068
27	oar3_OAR〉	26367716	-0.01799
27	oar3_OAR〉	26370764	-0.0084
27	oar3_OAR〉	26371073	-0.02443
27	oar3_OAR〉	26373406	0.0274
27	oar3_OAR〉	26378574	0.07194
27	oar3_OAR〉	26388989	-0.02995
27	oar3_OAR〉	26393045	-0.01709
27	oar3_OAR〉	26400028	-0.01069
27	oar3_OAR〉	26405843	-0.01886
27	oar3_OAR〉	26406146	0.028803
27	oar3_OAR〉	26412034	-0.0093
27	oar3_OAR〉	26414608	-0.0159
27	oar3_OAR〉	26417156	-0.01459
27	oar3_OAR〉	26417779	-0.0093
27	oar3_OAR〉	26421649	0.000877
27	oar3_OAR〉	26425490	0.021224
27	oar3_OAR〉	26426795	0.007511
27	oar3_OAR〉	26433482	0.021224
27	oar3_OAR〉	26434876	-0.03098
27	oar3_OAR〉	26435095	-0.03098
27	oar3_OAR〉	26446836	-0.00677
27	oar3_OAR〉	26448442	0.021865
27	oar3_OAR〉	26448970	-0.01717
27	oar3_OAR〉	26455854	0.003446
27	oar3_OAR〉	26457106	-0.01845
27	oar3_OAR〉	26474587	-0.01009
27	oar3_OAR〉	26475056	-0.00954
27	oar3_OAR〉	26478081	-0.03266
27	oar3_OAR〉	26483217	0.006981
27	oar3_OAR〉	26490686	-0.03231

27	oar3_OAR›	26494674	-0.03066
27	oar3_OAR›	26506498	0.003894
27	oar3_OAR›	26506592	-0.02526
27	oar3_OAR›	26514029	-0.01139
27	oar3_OAR›	26515617	-0.01191
27	oar3_OAR›	26522292	-0.02238
27	oar3_OAR›	26527292	-0.02077
27	oar3_OAR›	26528851	0.046951
27	oar3_OAR›	26529207	-0.0021
27	oar3_OAR›	26546526	-0.02775
27	oar3_OAR›	26555428	0.011041
27	oar3_OAR›	26556075	-0.0004
27	oar3_OAR›	26561490	-0.01854
27	oar3_OAR›	26563934	-0.0156
27	oar3_OAR›	26584326	-0.01702
27	oar3_OAR›	26585741	0.029652
27	oar3_OAR›	26591219	0.008134
27	oar3_OAR›	26591884	-0.0092
27	oar3_OAR›	26594007	-0.01645
27	oar3_OAR›	26594362	-0.01645
27	oar3_OAR›	26596928	0.013328
27	oar3_OAR›	26603555	-0.0209
27	oar3_OAR›	26614058	0.098799
27	oar3_OAR›	26614137	-0.00463
27	oar3_OAR›	26619543	0.093288
27	oar3_OAR›	26628416	0.053216
27	oar3_OAR›	26630360	0.114816
27	oar3_OAR›	26638673	-0.01885
27	oar3_OAR›	26644906	0.081272
27	oar3_OAR›	26647699	0.041412
27	oar3_OAR›	26649248	-0.01775
27	oar3_OAR›	26655906	-0.03326
27	oar3_OAR›	26655958	-0.0321
27	oar3_OAR›	26657811	-0.02522
27	oar3_OAR›	26658041	-0.02708
27	oar3_OAR›	26666364	0.015597
27	oar3_OAR›	26671532	0.068886
27	oar3_OAR›	26671743	0.018601
27	oar3_OAR›	26671917	-0.00874
27	oar3_OAR›	26679359	0.066154
27	oar3_OAR›	26683450	0.000363
27	oar3_OAR›	26684163	0.000363
27	oar3_OAR›	26692723	-0.00768
27	oar3_OAR›	26697125	-0.02591
27	oar3_OAR›	26706323	-0.00712
27	oar3_OAR›	26711254	-0.0344
27	oar3_OAR›	26711963	-0.0344
27	oar3_OAR›	26712883	0.005718
27	oar3_OAR›	26723489	-0.03258
27	oar3_OAR›	26725764	-0.03114
27	oar3_OAR›	26732531	-0.02351
27	oar3_OAR›	26734075	-0.00416
27	oar3_OAR›	26734252	-0.00416
27	oar3_OAR›	26734556	-0.00383

27	oar3_OAR›	26746510	-0.03705
27	oar3_OAR›	26748584	-0.02658
27	oar3_OAR›	26751681	-0.03382
27	oar3_OAR›	26757467	-0.00098
27	oar3_OAR›	26759669	-0.00417
27	oar3_OAR›	26761733	-0.01818
27	oar3_OAR›	26764262	-0.02683
27	oar3_OAR›	26774878	0.02676
27	oar3_OAR›	26776042	0.070127
27	oar3_OAR›	26804484	-0.00769
27	oar3_OAR›	26808834	7.84E-05
27	oar3_OAR›	26809553	0.011181
27	oar3_OAR›	26809752	0.00783
27	oar3_OAR›	26815734	0.048833
27	oar3_OAR›	26820389	0.000374
27	oar3_OAR›	26821149	0.011067
27	oar3_OAR›	26832732	0.042322
27	oar3_OAR›	26834246	0.06015
27	oar3_OAR›	26835851	0.001507
27	oar3_OAR›	26836290	-0.02132
27	oar3_OAR›	26844986	0.05523
27	oar3_OAR›	26850122	0.039538
27	oar3_OAR›	26851560	-0.02158
27	oar3_OAR›	26857288	-0.02229
27	oar3_OAR›	26864413	-0.0056
27	oar3_OAR›	26865568	-0.01295
27	oar3_OAR›	26868453	0.051359
27	oar3_OAR›	26868615	0.046743
27	oar3_OAR›	26874106	0.062797
27	oar3_OAR›	26880050	0.062797
27	oar3_OAR›	26880317	0.062797
27	oar3_OAR›	26880560	0.062797
27	oar3_OAR›	26883409	0.062797
27	oar3_OAR›	26887223	-0.01737
27	oar3_OAR›	26892694	-0.03641
27	oar3_OAR›	26892862	0.0317
27	oar3_OAR›	26893276	0.023228
27	oar3_OAR›	26895287	0.03863
27	oar3_OAR›	26902548	0.079637
27	oar3_OAR›	26906947	0.05351
27	oar3_OAR›	26908924	0.025714
27	oar3_OAR›	26920350	0.031886
27	oar3_OAR›	26926782	-0.01295
27	oar3_OAR›	26931753	0.018071
27	oar3_OAR›	26933341	0.117738
27	oar3_OAR›	26937324	0.051246
27	oar3_OAR›	26944434	0.142971
27	oar3_OAR›	26947068	0.146409
27	oar3_OAR›	26958478	0.02306
27	oar3_OAR›	26959481	0.02306
27	oar3_OAR›	26960926	-0.03169
27	oar3_OAR›	26964503	0.065419
27	oar3_OAR›	26970039	0.035666
27	oar3_OAR›	26977461	-0.01717

27	oar3_OAR〉	26978404	0.103477
27	oar3_OAR〉	26979580	-0.0347
27	oar3_OAR〉	26991058	-0.00577
27	oar3_OAR〉	26992813	-0.02708
27	oar3_OAR〉	26994425	-0.01519
27	oar3_OAR〉	26999256	0.016983
27	oar3_OAR〉	27006110	0.034769
27	oar3_OAR〉	27007066	0.018953
27	oar3_OAR〉	27008918	0.010581
27	oar3_OAR〉	27009960	0.00293
27	oar3_OAR〉	27013666	-0.00574
27	oar3_OAR〉	27027625	-0.02159
27	oar3_OAR〉	27031016	-0.02851
27	oar3_OAR〉	27031575	0.034769
27	oar3_OAR〉	27036127	0.100947
27	oar3_OAR〉	27043785	-0.00574
27	oar3_OAR〉	27045677	-0.02382
27	oar3_OAR〉	27045847	-0.00498
27	oar3_OAR〉	27049307	0.015023
27	oar3_OAR〉	27051790	-0.03024
27	oar3_OAR〉	27055320	0.001984
27	oar3_OAR〉	27055811	0.067322
27	oar3_OAR〉	27063715	0.026851
27	oar3_OAR〉	27064621	-0.01737
27	oar3_OAR〉	27072428	-0.03497
27	oar3_OAR〉	27072435	0.011503
27	oar3_OAR〉	27080311	-0.00379
27	oar3_OAR〉	27082201	-0.01143
27	oar3_OAR〉	27082887	-0.01143
27	oar3_OAR〉	27089943	-0.03062
27	oar3_OAR〉	27092961	-0.0185
27	oar3_OAR〉	27098169	0.042679
27	oar3_OAR〉	27099032	-0.03504
27	oar3_OAR〉	27103533	-0.03497
27	oar3_OAR〉	27117763	-0.01045
27	oar3_OAR〉	27123800	-0.01117
27	oar3_OAR〉	27127287	0.015492
27	oar3_OAR〉	27129932	-0.01651
27	oar3_OAR〉	27134057	-0.02926
27	oar3_OAR〉	27139405	-0.02926
27	oar3_OAR〉	27141187	0.035713
27	oar3_OAR〉	27154950	0.049338
27	oar3_OAR〉	27155852	-0.02159
27	oar3_OAR〉	27155987	0.035646
27	oar3_OAR〉	27160762	-0.01567
27	oar3_OAR〉	27166959	-0.01043
27	oar3_OAR〉	27168122	0.052739
27	oar3_OAR〉	27168547	0.038117
27	oar3_OAR〉	27174240	0.040355
27	oar3_OAR〉	27177340	0.025066
27	oar3_OAR〉	27180088	-0.00406
27	oar3_OAR〉	27180179	0.026177
27	oar3_OAR〉	27180192	0.034785
27	oar3_OAR〉	27185344	0.005452

27	oar3_OAR>	27186013	-0.03022
27	oar3_OAR>	27191774	0.024368
27	oar3_OAR>	27198012	-0.02237
27	oar3_OAR>	27199438	0.053453
27	oar3_OAR>	27201241	0.060545
27	oar3_OAR>	27209079	0.083142
27	oar3_OAR>	27209631	0.052709
27	oar3_OAR>	27214033	0.060033
27	oar3_OAR>	27219956	0.05061
27	oar3_OAR>	27223270	0.02441
27	oar3_OAR>	27227926	0.045334
27	oar3_OAR>	27229423	0.045334
27	oar3_OAR>	27229688	0.045334
27	oar3_OAR>	27239189	0.00481
27	oar3_OAR>	27239223	0.012261
27	oar3_OAR>	27244314	0.005579
27	oar3_OAR>	27245189	-0.02548
27	oar3_OAR>	27249611	0.010877
27	oar3_OAR>	27252396	-0.01463
27	oar3_OAR>	27257488	0.027062
27	oar3_OAR>	27258045	-0.02744
27	oar3_OAR>	27271272	0.018044
27	oar3_OAR>	27276967	0.004594
27	oar3_OAR>	27284577	-0.01525
27	oar3_OAR>	27289581	0.024124
27	oar3_OAR>	27295512	0.00043
27	oar3_OAR>	27295691	0.000399
27	oar3_OAR>	27299702	0.023073
27	oar3_OAR>	27300288	0.023073
27	oar3_OAR>	27302324	0.021891
27	oar3_OAR>	27310193	0.029783
27	oar3_OAR>	27317224	-0.01338
27	oar3_OAR>	27317610	-0.00598
27	oar3_OAR>	27320476	-0.02371
27	oar3_OAR>	27328671	-0.03371
27	oar3_OAR>	27331681	-0.00606
27	oar3_OAR>	27347791	0.022902
27	oar3_OAR>	27354509	-0.00674
27	oar3_OAR>	27360843	-0.00606
27	oar3_OAR>	27374804	0.032459
27	oar3_OAR>	27384470	-0.01356
27	oar3_OAR>	27385772	0.061768
27	oar3_OAR>	27397344	0.060943
27	oar3_OAR>	27452409	0.05527
27	oar3_OAR>	27471196	-0.00037
27	oar3_OAR>	27494830	-0.02525
27	oar3_OAR>	27499641	0.022432
27	oar3_OAR>	27500648	0.031225
27	oar3_OAR>	27500713	0.047576
27	oar3_OAR>	27506271	0.043603
27	oar3_OAR>	27514688	0.004924
27	oar3_OAR>	27521009	0.040509
27	oar3_OAR>	27537842	-0.02174
27	oar3_OAR>	27546617	-0.00933

27	oar3_OAR	27548301	0.059793
27	oar3_OAR	27549739	0.017568
27	oar3_OAR	27552512	-0.03175
27	oar3_OAR	27555397	0.060681
27	oar3_OAR	27560127	-0.03458
27	oar3_OAR	27564910	-0.00682
27	oar3_OAR	27564947	-0.00928
27	oar3_OAR	27566798	-0.00209
27	oar3_OAR	27575490	-0.01032
27	oar3_OAR	27580878	0.035529
27	oar3_OAR	27583652	0.018106
27	oar3_OAR	27588722	0.038456
27	oar3_OAR	27590775	0.018106
27	oar3_OAR	27591036	-0.00195
27	oar3_OAR	27593547	0.006708
27	oar3_OAR	27596152	0.002898
27	oar3_OAR	27599336	-0.0229
27	oar3_OAR	27603697	-0.00622
27	oar3_OAR	27610938	0.013555
27	oar3_OAR	27611294	-0.00986
27	oar3_OAR	27611327	-0.02498
27	oar3_OAR	27619425	0.013555
27	oar3_OAR	27620115	-0.00929
27	oar3_OAR	27622561	-0.02498
27	oar3_OAR	27628924	0.013555
27	oar3_OAR	27635469	-0.02498
27	oar3_OAR	27639467	-0.01162
27	oar3_OAR	27643897	-0.02498
27	oar3_OAR	27643996	-0.02498
27	oar3_OAR	27650782	-0.00707
27	oar3_OAR	27652023	0.001583
27	oar3_OAR	27653058	0.001554
27	oar3_OAR	27659390	0.016892
27	oar3_OAR	27660357	-0.01628
27	oar3_OAR	27663457	-0.01628
27	oar3_OAR	27672199	0.057108
27	oar3_OAR	27681184	0.050904
27	oar3_OAR	27686728	0.093322
27	oar3_OAR	27691523	-0.0287
27	oar3_OAR	27692597	-0.0287
27	oar3_OAR	27698445	-0.01611
27	oar3_OAR	27702621	-0.00189
27	oar3_OAR	27703568	-0.0031
27	oar3_OAR	27706096	-0.00275
27	oar3_OAR	27711970	0.048438
27	oar3_OAR	27712257	0.122298
27	oar3_OAR	27713689	0.09465
27	oar3_OAR	27724215	0.001963
27	oar3_OAR	27724954	0.01636
27	oar3_OAR	27725168	-0.00993
27	oar3_OAR	27737010	0.069301
27	oar3_OAR	27739413	-0.01117
27	oar3_OAR	27739689	0.066613
27	oar3_OAR	27745553	0.080158

27	oar3_OAR›	27746094	0.117048
27	oar3_OAR›	27749515	0.129787
27	oar3_OAR›	27751179	0.035002
27	oar3_OAR›	27757706	0.123773
27	oar3_OAR›	27761448	0.160504
27	oar3_OAR›	27761753	0.19185
27	oar3_OAR›	27768272	-0.01012
27	oar3_OAR›	27771792	0.042137
27	oar3_OAR›	27774855	-0.01172
27	oar3_OAR›	27783066	-0.01875
27	oar3_OAR›	27784534	-0.01734
27	oar3_OAR›	27795108	0.002836
27	oar3_OAR›	27800326	0.021496
27	oar3_OAR›	27808416	-0.02782
27	oar3_OAR›	27810208	0.072367
27	oar3_OAR›	27811099	0.026827
27	oar3_OAR›	27818082	0.01451
27	oar3_OAR›	27818519	-0.00102
27	oar3_OAR›	27823105	0.006205
27	oar3_OAR›	27823995	-0.02255
27	oar3_OAR›	27825986	0.016767
27	oar3_OAR›	27836682	-0.01748
27	oar3_OAR›	27837060	-0.01356
27	oar3_OAR›	27838030	-0.01748
27	oar3_OAR›	27848328	0.061554
27	oar3_OAR›	27848393	-0.02666
27	oar3_OAR›	27852085	-0.0132
27	oar3_OAR›	27858610	-0.0132
27	oar3_OAR›	27861215	-0.01489
27	oar3_OAR›	27861476	-0.03062
27	oar3_OAR›	27868175	-0.0188
27	oar3_OAR›	27873545	-0.01338
27	oar3_OAR›	27875395	-0.01338
27	oar3_OAR›	27875653	-0.02947
27	oar3_OAR›	27880665	-0.01193
27	oar3_OAR›	27883852	-0.02515
27	oar3_OAR›	27889746	-0.028
27	oar3_OAR›	27890480	0.008032
27	oar3_OAR›	27894917	0.009799
27	oar3_OAR›	27904130	-0.03673
27	oar3_OAR›	27912639	0.009737
27	oar3_OAR›	27913182	-0.03673
27	oar3_OAR›	27919489	-0.03673
27	oar3_OAR›	27925268	-0.03673
27	oar3_OAR›	27926676	0.017792
27	oar3_OAR›	27929370	-0.01053
27	oar3_OAR›	27932437	0.069468
27	oar3_OAR›	27947744	0.016028
27	oar3_OAR›	27961181	0.044633
27	oar3_OAR›	27963318	0.022402
27	oar3_OAR›	27966605	0.037113
27	oar3_OAR›	27973941	0.04151
27	oar3_OAR›	27976295	0.04151
27	oar3_OAR›	27983292	0.022402

27	oar3_OAR›	27989768	-0.02507
27	oar3_OAR›	27991575	-0.00276
27	oar3_OAR›	28002861	0.012855
27	oar3_OAR›	28007351	-0.02926
27	oar3_OAR›	28008756	0.02374
27	oar3_OAR›	28015609	-0.01069
27	oar3_OAR›	28046044	0.122778
27	oar3_OAR›	28051928	0.122778
27	oar3_OAR›	28062973	0.092823
27	oar3_OAR›	28076852	0.007934
27	oar3_OAR›	28077824	-0.00352
27	oar3_OAR›	28085298	0.030586
27	oar3_OAR›	28108116	-0.01043
27	oar3_OAR›	28129378	-0.03075
27	oar3_OAR›	28151165	0.034274
27	oar3_OAR›	28153651	0.023763
27	oar3_OAR›	28156206	0.050888
27	oar3_OAR›	28167579	0.009746
27	oar3_OAR›	28168620	0.016149
27	oar3_OAR›	28178260	-0.01288
27	oar3_OAR›	28187452	-0.02546
27	oar3_OAR›	28187685	-0.02546
27	oar3_OAR›	28194771	-0.02622
27	oar3_OAR›	28201673	-0.01957
27	oar3_OAR›	28202151	0.041346
27	oar3_OAR›	28202358	-0.01201
27	oar3_OAR›	28202471	-0.00589
27	oar3_OAR›	28212670	-0.031
27	oar3_OAR›	28214245	0.033414
27	oar3_OAR›	28227470	-0.02652
27	oar3_OAR›	28232484	0.019555
27	oar3_OAR›	28237755	0.013189
27	oar3_OAR›	28240337	0.013189
27	oar3_OAR›	28245524	-0.00689
27	oar3_OAR›	28245583	-0.00223
27	oar3_OAR›	28248459	0.015203
27	oar3_OAR›	28255980	0.015203
27	oar3_OAR›	28256789	0.008542
27	oar3_OAR›	28266165	0.008542
27	oar3_OAR›	28266831	0.001258
27	oar3_OAR›	28266914	-0.02368
27	oar3_OAR›	28269922	0.000427
27	oar3_OAR›	28272732	-0.0236
27	oar3_OAR›	28277513	-0.02267
27	oar3_OAR›	28278324	-0.01527
27	oar3_OAR›	28279548	0.022632
27	oar3_OAR›	28284672	-0.03786
27	oar3_OAR›	28291335	-0.03206
27	oar3_OAR›	28299271	0.008134
27	oar3_OAR›	28301059	-0.01558
27	oar3_OAR›	28307941	-0.03786
27	oar3_OAR›	28311222	-0.03786
27	oar3_OAR›	28312911	-0.02945
27	oar3_OAR›	28316205	-0.03354

27	oar3_OAR〉	28325256	0.004188
27	oar3_OAR〉	28327521	-0.00701
27	oar3_OAR〉	28327974	-0.03295
27	oar3_OAR〉	28334415	0.016466
27	oar3_OAR〉	28338155	-0.01596
27	oar3_OAR〉	28345133	-0.00113
27	oar3_OAR〉	28347809	-0.03093
27	oar3_OAR〉	28351355	-0.03093
27	oar3_OAR〉	28351596	0.02024
27	oar3_OAR〉	28356287	0.02024
27	oar3_OAR〉	28358762	-0.02441
27	oar3_OAR〉	28363281	-0.02948
27	oar3_OAR〉	28378888	-0.02112
27	oar3_OAR〉	28380642	-0.01922
27	oar3_OAR〉	28384337	0.018564
27	oar3_OAR〉	28391049	-0.01967
27	oar3_OAR〉	28394049	0.016028
27	oar3_OAR〉	28400707	-0.02448
27	oar3_OAR〉	28403092	-0.00957
27	oar3_OAR〉	28403229	-0.00957
27	oar3_OAR〉	28403521	-0.03662
27	oar3_OAR〉	28409785	0.016364
27	oar3_OAR〉	28417100	0.035647
27	oar3_OAR〉	28418392	-0.01618
27	oar3_OAR〉	28418650	0.035647
27	oar3_OAR〉	28419345	-0.01373
27	oar3_OAR〉	28429596	0.031879
27	oar3_OAR〉	28435925	-0.00502
27	oar3_OAR〉	28439267	0.048281
27	oar3_OAR〉	28442754	-0.03332
27	oar3_OAR〉	28444177	-0.00396
27	oar3_OAR〉	28446307	-0.02015
27	oar3_OAR〉	28447585	-0.00396
27	oar3_OAR〉	28454227	-0.00936
27	oar3_OAR〉	28458626	-0.03332
27	oar3_OAR〉	28458750	-0.02577
27	oar3_OAR〉	28465353	-0.00996
27	oar3_OAR〉	28467809	0.013595
27	oar3_OAR〉	28468858	0.016046
27	oar3_OAR〉	28478417	-0.01445
27	oar3_OAR〉	28480967	-0.02672
27	oar3_OAR〉	28486245	-0.02162
27	oar3_OAR〉	28488588	0.009529
27	oar3_OAR〉	28491345	-0.02769
27	oar3_OAR〉	28493015	-0.0013
27	oar3_OAR〉	28493821	-0.02744
27	oar3_OAR〉	28500786	0.069268
27	oar3_OAR〉	28501023	0.069268
27	oar3_OAR〉	28502587	0.043608
27	oar3_OAR〉	28506694	-0.02184
27	oar3_OAR〉	28508014	-0.014
27	oar3_OAR〉	28508686	0.037131
27	oar3_OAR〉	28511828	-0.0168
27	oar3_OAR〉	28513208	-0.01893

27	oar3_OAR}	28521132	-0.00019
27	oar3_OAR}	28522925	0.033005
27	oar3_OAR}	28523382	-0.01871
27	oar3_OAR}	28526342	0.032882
27	oar3_OAR}	28531938	-0.01368
27	oar3_OAR}	28534192	-0.00997
27	oar3_OAR}	28535629	-0.02413
27	oar3_OAR}	28539764	0.014501
27	oar3_OAR}	28540078	-0.00075
27	oar3_OAR}	28542731	-0.00673
27	oar3_OAR}	28544369	0.019456
27	oar3_OAR}	28552644	-0.014
27	oar3_OAR}	28558718	0.00395
27	oar3_OAR}	28565977	0.008296
27	oar3_OAR}	28574219	0.08121
27	oar3_OAR}	28574440	-0.01224
27	oar3_OAR}	28577739	-0.00735
27	oar3_OAR}	28578799	-0.01069
27	oar3_OAR}	28583624	0.037641
27	oar3_OAR}	28583977	-0.01069
27	oar3_OAR}	28585135	0.026936
27	oar3_OAR}	28598968	-0.03148
27	oar3_OAR}	28602648	0.029146
27	oar3_OAR}	28603958	-0.02603
27	oar3_OAR}	28609629	-0.00996
27	oar3_OAR}	28613380	-0.01385
27	oar3_OAR}	28617787	0.062996
27	oar3_OAR}	28617899	0.031241
27	oar3_OAR}	28619402	-0.0425
27	oar3_OAR}	28624368	0.007039
27	oar3_OAR}	28628811	0.050043
27	oar3_OAR}	28629575	0.023093
27	oar3_OAR}	28633914	-0.00508
27	oar3_OAR}	28634618	0.021081
27	oar3_OAR}	28635088	-0.01567
27	oar3_OAR}	28639725	-0.00287
27	oar3_OAR}	28640157	-0.00531
27	oar3_OAR}	28648274	0.023093
27	oar3_OAR}	28652102	0.004113
27	oar3_OAR}	28655293	0.021937
27	oar3_OAR}	28655911	-0.02736
27	oar3_OAR}	28661007	-0.00188
27	oar3_OAR}	28662920	-0.00068
27	oar3_OAR}	28668272	0.016214
27	oar3_OAR}	28672109	0.07187
27	oar3_OAR}	28674007	-0.03191
27	oar3_OAR}	28674573	NA
27	oar3_OAR}	28679017	-0.01828
27	oar3_OAR}	28685834	-0.01893
27	oar3_OAR}	28685956	0.011067
27	oar3_OAR}	28689522	-0.02564
27	oar3_OAR}	28691275	0.145398
27	oar3_OAR}	28692310	0.020087
27	oar3_OAR}	28698434	-0.02242

27	oar3_OAR›	28700038	-0.0225
27	oar3_OAR›	28700482	-0.02197
27	oar3_OAR›	28700693	-0.01779
27	oar3_OAR›	28703536	-0.0313
27	oar3_OAR›	28708561	0.005718
27	oar3_OAR›	28712031	-0.02
27	oar3_OAR›	28713536	-0.02593
27	oar3_OAR›	28717641	0.024365
27	oar3_OAR›	28722412	-0.03298
27	oar3_OAR›	28723349	0.009734
27	oar3_OAR›	28726251	-0.01414
27	oar3_OAR›	28728900	-0.03475
27	oar3_OAR›	28739763	0.013268
27	oar3_OAR›	28740805	0.001349
27	oar3_OAR›	28745183	-0.0405
27	oar3_OAR›	28756355	0.019138
27	oar3_OAR›	28757668	0.137732
27	oar3_OAR›	28764752	-0.01126
27	oar3_OAR›	28766768	NA
27	oar3_OAR›	28769228	0.057527
27	oar3_OAR›	28770652	-0.01916
27	oar3_OAR›	28771505	0.00712
27	oar3_OAR›	28781883	-0.01141
27	oar3_OAR›	28782757	0.011808
27	oar3_OAR›	28782965	0.046441
27	oar3_OAR›	28783045	0.058555
27	oar3_OAR›	28788213	-0.028
27	oar3_OAR›	28791762	0.000147
27	oar3_OAR›	28793469	-0.03558
27	oar3_OAR›	28793737	0.011808
27	oar3_OAR›	28793881	-0.03558
27	oar3_OAR›	28800091	0.029742
27	oar3_OAR›	28801156	-0.00205
27	oar3_OAR›	28812590	-0.02069
27	oar3_OAR›	28819715	0.013296
27	oar3_OAR›	28826998	0.071798
27	oar3_OAR›	28832308	0.042323
27	oar3_OAR›	28835079	0.013296
27	oar3_OAR›	28835903	-0.02683
27	oar3_OAR›	28839122	-0.00229
27	oar3_OAR›	28842916	-0.0253
27	oar3_OAR›	28845919	-0.02052
27	oar3_OAR›	28847894	-0.02891
27	oar3_OAR›	28847975	-0.02891
27	oar3_OAR›	28855072	-0.03081
27	oar3_OAR›	28855471	-0.03114
27	oar3_OAR›	28859727	-0.03099
27	oar3_OAR›	28861317	-0.02839
27	oar3_OAR›	28869226	-0.01378
27	oar3_OAR›	28871309	-0.02174
27	oar3_OAR›	28872174	-0.01119
27	oar3_OAR›	28874871	-0.02719
27	oar3_OAR›	28883902	-0.02375
27	oar3_OAR›	28885159	-0.01846

27	oar3_OAR›	28887151	-0.01809
27	oar3_OAR›	28888972	-0.03458
27	oar3_OAR›	28894578	0.004594
27	oar3_OAR›	28897783	0.036688
27	oar3_OAR›	28898056	-0.01922
27	oar3_OAR›	28912301	-0.01086
27	oar3_OAR›	28917086	0.02676
27	oar3_OAR›	28919254	0.008296
27	oar3_OAR›	28920561	-0.02382
27	oar3_OAR›	28925060	-0.00134
27	oar3_OAR›	28930933	-0.00032
27	oar3_OAR›	28931846	-0.00032
27	oar3_OAR›	28935816	-0.01423
27	oar3_OAR›	28940362	0.01925
27	oar3_OAR›	28941416	-0.0156
27	oar3_OAR›	28942973	0.000889
27	oar3_OAR›	28948304	0.022514
27	oar3_OAR›	28953712	0.0275
27	oar3_OAR›	28955983	-0.01567
27	oar3_OAR›	28958683	-0.01356
27	oar3_OAR›	28965383	0.026392
27	oar3_OAR›	28968619	0.074839
27	oar3_OAR›	28970952	0.013417
27	oar3_OAR›	28971789	0.145371
27	oar3_OAR›	28977605	0.026392
27	oar3_OAR›	28980629	0.032431
27	oar3_OAR›	28982544	-0.03075
27	oar3_OAR›	28992640	0.046532
27	oar3_OAR›	28992828	-0.01636
27	oar3_OAR›	28993318	0.027176
27	oar3_OAR›	28993499	-0.01567
27	oar3_OAR›	28999038	0.031038
27	oar3_OAR›	29004146	0.031432
27	oar3_OAR›	29004645	-0.0081
27	oar3_OAR›	29013442	0.003987
27	oar3_OAR›	29014618	0.015319
27	oar3_OAR›	29017927	-0.01153
27	oar3_OAR›	29022048	-0.01567
27	oar3_OAR›	29023109	0.050888
27	oar3_OAR›	29024149	-0.01749
27	oar3_OAR›	29029486	-0.01567
27	oar3_OAR›	29044863	-0.00874
27	oar3_OAR›	29045323	-0.00428
27	oar3_OAR›	29046682	0.035413
27	oar3_OAR›	29052691	0.021533
27	oar3_OAR›	29053443	0.002868
27	oar3_OAR›	29058080	0.064242
27	oar3_OAR›	29059471	0.017625
27	oar3_OAR›	29063414	0.064242
27	oar3_OAR›	29065081	-0.01717
27	oar3_OAR›	29070847	-0.01338
27	oar3_OAR›	29071117	0.024024
27	oar3_OAR›	29073591	-0.01225
27	oar3_OAR›	29076137	0.024024

27	oar3_OAR›	29076354	0.040785
27	oar3_OAR›	29084906	0.050165
27	oar3_OAR›	29086586	0.036091
27	oar3_OAR›	29086648	0.060748
27	oar3_OAR›	29090897	0.068499
27	oar3_OAR›	29093176	0.062642
27	oar3_OAR›	29098200	0.012705
27	oar3_OAR›	29098667	-0.0139
27	oar3_OAR›	29101159	0.011849
27	oar3_OAR›	29110015	-0.03464
27	oar3_OAR›	29113958	-0.02906
27	oar3_OAR›	29117692	-0.02292
27	oar3_OAR›	29128048	-0.01866
27	oar3_OAR›	29128882	-0.02645
27	oar3_OAR›	29135467	-0.034
27	oar3_OAR›	29135729	0.046963
27	oar3_OAR›	29138686	0.034692
27	oar3_OAR›	29141027	-0.03337
27	oar3_OAR›	29141711	-0.01611
27	oar3_OAR›	29146188	-0.02571
27	oar3_OAR›	29148524	-0.02297
27	oar3_OAR›	29151414	-0.00848
27	oar3_OAR›	29151591	-0.0185
27	oar3_OAR›	29156565	-0.01925
27	oar3_OAR›	29162109	-0.02356
27	oar3_OAR›	29164553	-0.02428
27	oar3_OAR›	29169778	-0.01101
27	oar3_OAR›	29177894	-0.01664
27	oar3_OAR›	29179097	-0.00058
27	oar3_OAR›	29180026	0.045573
27	oar3_OAR›	29185222	0.024815
27	oar3_OAR›	29187113	0.001778
27	oar3_OAR›	29193212	0.01603
27	oar3_OAR›	29196011	-0.03226
27	oar3_OAR›	29196501	0.001778
27	oar3_OAR›	29208629	0.011116
27	oar3_OAR›	29209336	0.011116
27	oar3_OAR›	29209425	0.011116
27	oar3_OAR›	29222181	0.002191
27	oar3_OAR›	29222831	-0.0054
27	oar3_OAR›	29224689	-0.01887
27	oar3_OAR›	29225499	-0.01963
27	oar3_OAR›	29227888	-0.00114
27	oar3_OAR›	29231885	0.012341
27	oar3_OAR›	29238963	-0.02844
27	oar3_OAR›	29240981	0.01084
27	oar3_OAR›	29254928	7.25E-05
27	oar3_OAR›	29257730	-0.03509
27	oar3_OAR›	29262211	-0.02645
27	oar3_OAR›	29267953	-0.01628
27	oar3_OAR›	29278760	-0.01628
27	oar3_OAR›	29285480	-0.0264
27	oar3_OAR›	29285708	-0.03171
27	oar3_OAR›	29290056	-0.00932

27	oar3_OAR›	29291229	-0.01186
27	oar3_OAR›	29300403	-0.01804
27	oar3_OAR›	29301686	-0.00363
27	oar3_OAR›	29302125	-0.03054
27	oar3_OAR›	29311211	0.009746
27	oar3_OAR›	29311597	-0.00121
27	oar3_OAR›	29312711	-0.01687
27	oar3_OAR›	29316730	-0.03031
27	oar3_OAR›	29318735	-0.00927
27	oar3_OAR›	29320737	0.015985
27	oar3_OAR›	29322305	-0.03509
27	oar3_OAR›	29323244	-0.0158
27	oar3_OAR›	29327853	0.012006
27	oar3_OAR›	29336139	-0.02638
27	oar3_OAR›	29336916	0.016767
27	oar3_OAR›	29337187	-0.00788
27	oar3_OAR›	29343772	0.004822
27	oar3_OAR›	29347170	-0.03679
27	oar3_OAR›	29351956	0.026703
27	oar3_OAR›	29355423	-0.0294
27	oar3_OAR›	29356209	-0.03679
27	oar3_OAR›	29359277	-0.0053
27	oar3_OAR›	29360864	-0.03679
27	oar3_OAR›	29366354	-0.02933
27	oar3_OAR›	29366408	-0.02593
27	oar3_OAR›	29371424	0.022695
27	oar3_OAR›	29373931	0.008431
27	oar3_OAR›	29376653	0.008431
27	oar3_OAR›	29376959	-0.00232
27	oar3_OAR›	29377192	0.00481
27	oar3_OAR›	29384727	0.001442
27	oar3_OAR›	29387492	-0.02049
27	oar3_OAR›	29387600	0.001442
27	oar3_OAR›	29388231	-0.00677
27	oar3_OAR›	29393803	-0.00733
27	oar3_OAR›	29393828	-0.02184
27	oar3_OAR›	29398661	-0.00657
27	oar3_OAR›	29398830	-0.02593
27	oar3_OAR›	29401899	0.000889
27	oar3_OAR›	29410422	-0.0347
27	oar3_OAR›	29418891	0.000944
27	oar3_OAR›	29419714	0.000944
27	oar3_OAR›	29422359	-0.01338
27	oar3_OAR›	29425570	0.000944
27	oar3_OAR›	29431215	0.018804
27	oar3_OAR›	29432356	0.058996
27	oar3_OAR›	29440445	0.000944
27	oar3_OAR›	29440570	0.058996
27	oar3_OAR›	29444325	-0.00936
27	oar3_OAR›	29447160	0.031918
27	oar3_OAR›	29448616	NA
27	oar3_OAR›	29452780	-0.00137
27	oar3_OAR›	29453807	-0.00411
27	oar3_OAR›	29457307	-0.03135

27	oar3_OAR〉	29462922	0.004034
27	oar3_OAR〉	29469508	-0.02926
27	oar3_OAR〉	29474469	-0.01418
27	oar3_OAR〉	29477313	-0.00963
27	oar3_OAR〉	29478453	-0.00963
27	oar3_OAR〉	29480456	NA
27	oar3_OAR〉	29486066	0.001957
27	oar3_OAR〉	29488950	0.001957
27	oar3_OAR〉	29498286	-0.0264
27	oar3_OAR〉	29501821	-0.00666
27	oar3_OAR〉	29504564	-0.01311
27	oar3_OAR〉	29506692	-0.0264
27	oar3_OAR〉	29511870	NA
27	oar3_OAR〉	29512970	0.039826
27	oar3_OAR〉	29514876	0.01868
27	oar3_OAR〉	29515595	-0.00673
27	oar3_OAR〉	29525701	0.024613
27	oar3_OAR〉	29528249	0.032258
27	oar3_OAR〉	29534481	-0.01357
27	oar3_OAR〉	29534581	0.063275
27	oar3_OAR〉	29537980	0.049695
27	oar3_OAR〉	29541781	-0.01357
27	oar3_OAR〉	29548560	0.011365
27	oar3_OAR〉	29553105	-0.02683
27	oar3_OAR〉	29566455	0.028803
27	oar3_OAR〉	29575838	-0.02424
27	oar3_OAR〉	29578514	-0.01144
27	oar3_OAR〉	29585806	0.027002
27	oar3_OAR〉	29586074	-0.0014
27	oar3_OAR〉	29591737	0.009891
27	oar3_OAR〉	29600999	-0.01069
27	oar3_OAR〉	29605011	0.025396
27	oar3_OAR〉	29608981	0.092997
27	oar3_OAR〉	29610267	-0.0092
27	oar3_OAR〉	29618156	-0.01423
27	oar3_OAR〉	29624977	0.065874
27	oar3_OAR〉	29629658	0.012045
27	oar3_OAR〉	29634263	0.012045
27	oar3_OAR〉	29640069	0.020136
27	oar3_OAR〉	29640778	-0.02577
27	oar3_OAR〉	29648118	0.014872
27	oar3_OAR〉	29649745	-0.00384
27	oar3_OAR〉	29652349	-0.03152
27	oar3_OAR〉	29653572	0.014872
27	oar3_OAR〉	29658922	0.037649
27	oar3_OAR〉	29660646	0.066592
27	oar3_OAR〉	29660848	0.066592
27	oar3_OAR〉	29660899	0.035242
27	oar3_OAR〉	29667826	-0.02621
27	oar3_OAR〉	29677632	-0.00115
27	oar3_OAR〉	29680769	0.023961
27	oar3_OAR〉	29681868	NA
27	oar3_OAR〉	29687613	0.056205
27	oar3_OAR〉	29689461	0.005432

27	oar3_OAR	29692647	0.009298
27	oar3_OAR	29695558	-0.01873
27	oar3_OAR	29698324	-0.00863
27	oar3_OAR	29703882	-0.01737
27	oar3_OAR	29705604	-0.01902
27	oar3_OAR	29707943	0.04841
27	oar3_OAR	29710678	0.026152
27	oar3_OAR	29716135	0.024378
27	oar3_OAR	29719718	0.062308
27	oar3_OAR	29722150	-0.02381
27	oar3_OAR	29726325	0.005807
27	oar3_OAR	29731353	-0.02381
27	oar3_OAR	29732308	0.023064
27	oar3_OAR	29734928	0.023064
27	oar3_OAR	29738260	-0.02375
27	oar3_OAR	29739245	-0.02719
27	oar3_OAR	29746252	-0.01964
27	oar3_OAR	29746601	0.019698
27	oar3_OAR	29748763	-0.02209
27	oar3_OAR	29751651	-0.03075
27	oar3_OAR	29758189	-0.02381
27	oar3_OAR	29759245	0.136491
27	oar3_OAR	29760572	0.012984
27	oar3_OAR	29763711	-0.02381
27	oar3_OAR	29765119	0.011067
27	oar3_OAR	29771381	-0.0234
27	oar3_OAR	29773043	0.039131
27	oar3_OAR	29773111	0.039131
27	oar3_OAR	29779277	0.025091
27	oar3_OAR	29784218	-0.00192
27	oar3_OAR	29785579	-0.02561
27	oar3_OAR	29791851	-0.01411
27	oar3_OAR	29799749	-0.01931
27	oar3_OAR	29805324	-0.00451
27	oar3_OAR	29805711	-0.03769
27	oar3_OAR	29811134	0.121655
27	oar3_OAR	29811801	-0.02727
27	oar3_OAR	29816451	0.043648
27	oar3_OAR	29816513	0.043648
27	oar3_OAR	29825132	-0.00765
27	oar3_OAR	29827840	0.043451
27	oar3_OAR	29828105	0.083232
27	oar3_OAR	29830212	-0.02645
27	oar3_OAR	29835238	0.014104
27	oar3_OAR	29839220	0.026698
27	oar3_OAR	29840463	-0.02926
27	oar3_OAR	29840625	-0.02053
27	oar3_OAR	29850567	-0.02851
27	oar3_OAR	29850942	0.047261
27	oar3_OAR	29852332	-0.01743
27	oar3_OAR	29857336	0.17638
27	oar3_OAR	29862234	0.005166
27	oar3_OAR	29862494	0.005166
27	oar3_OAR	29865964	########

27	oar3_OAR›	29868931	0.003659
27	oar3_OAR›	29874538	-0.01731
27	oar3_OAR›	29877195	-0.00448
27	oar3_OAR›	29879078	-0.02807
27	oar3_OAR›	29882629	0.067884
27	oar3_OAR›	29886115	0.013553
27	oar3_OAR›	29888296	0.004113
27	oar3_OAR›	29893682	-0.02582
27	oar3_OAR›	29901894	-0.01497
27	oar3_OAR›	29903534	0.017807
27	oar3_OAR›	29903707	-0.01024
27	oar3_OAR›	29903949	-0.01963
27	oar3_OAR›	29908902	0.00115
27	oar3_OAR›	29914253	0.002019
27	oar3_OAR›	29914419	0.02829
27	oar3_OAR›	29915697	-0.01053
27	oar3_OAR›	29916387	-0.01497
27	oar3_OAR›	29921404	-0.01303
27	oar3_OAR›	29926338	-0.02159
27	oar3_OAR›	29929500	0.033563
27	oar3_OAR›	29939070	-0.02038
27	oar3_OAR›	29941026	0.034383
27	oar3_OAR›	29941814	0.005534
27	oar3_OAR›	29958180	-0.02922
27	oar3_OAR›	29959007	-0.0236
27	oar3_OAR›	29968517	0.007994
27	oar3_OAR›	29971929	-0.00284
27	oar3_OAR›	29974075	-0.0286
27	oar3_OAR›	29985795	-0.02217
27	oar3_OAR›	29985862	-0.03186
27	oar3_OAR›	29988784	0.026126
27	oar3_OAR›	29989184	0.103248
27	oar3_OAR›	29996244	0.103248
27	oar3_OAR›	30000770	0.103248
27	oar3_OAR›	30002569	0.032756
27	oar3_OAR›	30009261	-0.03248
27	oar3_OAR›	30009989	-0.01935
27	oar3_OAR›	30024766	0.046207
27	oar3_OAR›	30030373	0.025477
27	oar3_OAR›	30031742	0.005844
27	oar3_OAR›	30036485	-0.03114
27	oar3_OAR›	30036791	0.025477
27	oar3_OAR›	30037655	0.02235
27	oar3_OAR›	30056001	-0.00255
27	oar3_OAR›	30061024	0.12303
27	oar3_OAR›	30063781	-0.02544
27	oar3_OAR›	30066726	0.005166
27	oar3_OAR›	30069448	-0.01698
27	oar3_OAR›	30076296	0.065074
27	oar3_OAR›	30081869	-0.02861
27	oar3_OAR›	30083359	-0.02861
27	oar3_OAR›	30083450	0.065074
27	oar3_OAR›	30084941	-0.02557
27	oar3_OAR›	30090071	-0.0114

27	oar3_OAR	30099298	0.041488
27	oar3_OAR	30102626	-0.01121
27	oar3_OAR	30109012	-0.01771
27	oar3_OAR	30113528	0.022432
27	oar3_OAR	30119255	0.017194
27	oar3_OAR	30121553	-0.01717
27	oar3_OAR	30123165	0.0087
27	oar3_OAR	30127215	-0.00898
27	oar3_OAR	30129171	-0.00898
27	oar3_OAR	30132105	0.024368
27	oar3_OAR	30132385	-0.02184
27	oar3_OAR	30138164	-0.01241
27	oar3_OAR	30141543	0.113431
27	oar3_OAR	30143392	-0.01493
27	oar3_OAR	30143668	-0.01493
27	oar3_OAR	30149167	-0.01196
27	oar3_OAR	30153084	0.000889
27	oar3_OAR	30153724	-0.01493
27	oar3_OAR	30158134	-0.02875
27	oar3_OAR	30158571	7.25E-05
27	oar3_OAR	30176174	-0.02393
27	oar3_OAR	30178393	-0.00957
27	oar3_OAR	30185610	0.02001
27	oar3_OAR	30187652	-0.01758
27	oar3_OAR	30197180	-0.00723
27	oar3_OAR	30207180	-0.01993
27	oar3_OAR	30225118	0.003214
27	oar3_OAR	30233408	-0.00036
27	oar3_OAR	30237321	-0.00448
27	oar3_OAR	30238678	-0.02529
27	oar3_OAR	30239474	0.005819
27	oar3_OAR	30251612	-0.00898
27	oar3_OAR	30256587	-0.01483
27	oar3_OAR	30267546	-0.00535
27	oar3_OAR	30267630	-0.01581
27	oar3_OAR	30268565	-0.00187
27	oar3_OAR	30270599	-0.01483
27	oar3_OAR	30278434	-0.01483
27	oar3_OAR	30279528	-0.02153
27	oar3_OAR	30280897	-0.00535
27	oar3_OAR	30291474	0.0321
27	oar3_OAR	30297206	0.019766
27	oar3_OAR	30302571	-0.00933
27	oar3_OAR	30305026	-0.0012
27	oar3_OAR	30306819	0.039466
27	oar3_OAR	30317752	0.122116
27	oar3_OAR	30318348	0.028481
27	oar3_OAR	30319871	-0.02279
27	oar3_OAR	30325759	-0.04195
27	oar3_OAR	30326235	0.06488
27	oar3_OAR	30330773	-0.03645
27	oar3_OAR	30333502	0.055372
27	oar3_OAR	30334229	-0.01278
27	oar3_OAR	30339842	0.011798

27	oar3_OAR›	30342910	0.045441
27	oar3_OAR›	30349143	0.030359
27	oar3_OAR›	30352552	0.014851
27	oar3_OAR›	30353364	-0.03688
27	oar3_OAR›	30354242	-0.00676
27	oar3_OAR›	30359826	0.058278
27	oar3_OAR›	30359944	0.019484
27	oar3_OAR›	30371691	-0.02693
27	oar3_OAR›	30377350	-0.0267
27	oar3_OAR›	30381357	0.012242
27	oar3_OAR›	30383072	-0.03805
27	oar3_OAR›	30387533	0.003028
27	oar3_OAR›	30388747	0.086726
27	oar3_OAR›	30389801	-0.01835
27	oar3_OAR›	30407095	0.043014
27	oar3_OAR›	30409461	-0.00462
27	oar3_OAR›	30410584	0.11363
27	oar3_OAR›	30421129	0.020467
27	oar3_OAR›	30422455	-0.00462
27	oar3_OAR›	30424367	0.018013
27	oar3_OAR›	30428581	0.07728
27	oar3_OAR›	30440209	-0.00605
27	oar3_OAR›	30442659	0.009277
27	oar3_OAR›	30455530	0.05016
27	oar3_OAR›	30455622	-0.00195
27	oar3_OAR›	30460070	0.014442
27	oar3_OAR›	30462798	0.070915
27	oar3_OAR›	30463668	0.071783
27	oar3_OAR›	30463828	0.023353
27	oar3_OAR›	30471425	0.017106
27	oar3_OAR›	30473653	0.028173
27	oar3_OAR›	30474783	-0.02025
27	oar3_OAR›	30474931	0.021369
27	oar3_OAR›	30484681	0.013414
27	oar3_OAR›	30485578	0.02374
27	oar3_OAR›	30486726	0.019099
27	oar3_OAR›	30490458	0.000889
27	oar3_OAR›	30495213	-0.01186
27	oar3_OAR›	30497735	-0.02546
27	oar3_OAR›	30497939	-0.02879
27	oar3_OAR›	30498142	-0.03349
27	oar3_OAR›	30508779	0.016045
27	oar3_OAR›	30528256	0.020353
27	oar3_OAR›	30538710	-0.03733
27	oar3_OAR›	30539127	0.002868
27	oar3_OAR›	30544946	-0.0245
27	oar3_OAR›	30549660	0.024536
27	oar3_OAR›	30554431	0.022793
27	oar3_OAR›	30554829	-0.01523
27	oar3_OAR›	30556654	0.074553
27	oar3_OAR›	30560812	0.074553
27	oar3_OAR›	30564107	0.074553
27	oar3_OAR›	30567877	-0.00013
27	oar3_OAR›	30568716	-0.01992

27	oar3_OAR〉	30569131	-0.00758
27	oar3_OAR〉	30572888	0.006902
27	oar3_OAR〉	30574476	0.005993
27	oar3_OAR〉	30580157	-0.01737
27	oar3_OAR〉	30581018	-0.02564
27	oar3_OAR〉	30586568	-0.01871
27	oar3_OAR〉	30588199	-0.02694
27	oar3_OAR〉	30591685	-0.02575
27	oar3_OAR〉	30594757	-0.02251
27	oar3_OAR〉	30599498	-0.03384
27	oar3_OAR〉	30599715	-0.04121
27	oar3_OAR〉	30601355	0.010803
27	oar3_OAR〉	30606560	-0.02707
27	oar3_OAR〉	30609679	-0.00349
27	oar3_OAR〉	30610209	0.009815
27	oar3_OAR〉	30610929	0.002019
27	oar3_OAR〉	30613832	0.124703
27	oar3_OAR〉	30620553	-0.02546
27	oar3_OAR〉	30621032	-0.0113
27	oar3_OAR〉	30621500	-0.01489
27	oar3_OAR〉	30627139	0.005771
27	oar3_OAR〉	30635958	0.008571
27	oar3_OAR〉	30636838	0.014267
27	oar3_OAR〉	30640158	-0.01705
27	oar3_OAR〉	30650972	-0.02286
27	oar3_OAR〉	30655931	-0.01141
27	oar3_OAR〉	30660244	-0.00927
27	oar3_OAR〉	30660743	-0.01594
27	oar3_OAR〉	30679908	-0.01887
27	oar3_OAR〉	30680058	-0.01887
27	oar3_OAR〉	30681575	-0.01837
27	oar3_OAR〉	30686352	-0.0036
27	oar3_OAR〉	30694638	-0.00735
27	oar3_OAR〉	30694694	-0.02645
27	oar3_OAR〉	30699686	-0.00606
27	oar3_OAR〉	30700563	-0.00229
27	oar3_OAR〉	30702162	-0.01224
27	oar3_OAR〉	30710896	0.016537
27	oar3_OAR〉	30711818	-0.03351
27	oar3_OAR〉	30715948	-0.01731
27	oar3_OAR〉	30721785	-0.02744
27	oar3_OAR〉	30727389	-0.0385
27	oar3_OAR〉	30733732	-0.02381
27	oar3_OAR〉	30733790	0.012997
27	oar3_OAR〉	30741413	0.012997
27	oar3_OAR〉	30746343	0.000134
27	oar3_OAR〉	30748151	0.000134
27	oar3_OAR〉	30750457	0.012997
27	oar3_OAR〉	30755783	0.007039
27	oar3_OAR〉	30755827	-0.013
27	oar3_OAR〉	30756851	-0.02005
27	oar3_OAR〉	30761407	-0.03477
27	oar3_OAR〉	30764599	-0.01818
27	oar3_OAR〉	30769201	-0.02693

27	oar3_OAR›	30783791	0.070124
27	oar3_OAR›	30786590	0.070124
27	oar3_OAR›	30793019	0.051142
27	oar3_OAR›	30796742	0.042256
27	oar3_OAR›	30798287	0.025934
27	oar3_OAR›	30803279	-0.02825
27	oar3_OAR›	30803561	0.01985
27	oar3_OAR›	30811127	-0.01104
27	oar3_OAR›	30811364	-0.03369
27	oar3_OAR›	30818199	-0.02725
27	oar3_OAR›	30818516	-0.01437
27	oar3_OAR›	30819020	-0.02725
27	oar3_OAR›	30826362	-0.03369
27	oar3_OAR›	30828138	0.004757
27	oar3_OAR›	30829267	0.004757
27	oar3_OAR›	30831326	0.006769
27	oar3_OAR›	30833289	0.066891
27	oar3_OAR›	30839651	-0.01014
27	oar3_OAR›	30840148	0.01024
27	oar3_OAR›	30843418	-0.014
27	oar3_OAR›	30854325	0.042921
27	oar3_OAR›	30854382	-0.01455
27	oar3_OAR›	30858581	-0.02744
27	oar3_OAR›	30867045	-0.00243
27	oar3_OAR›	30867203	-0.02744
27	oar3_OAR›	30869587	0.007224
27	oar3_OAR›	30874578	0.080827
27	oar3_OAR›	30882166	-0.02744
27	oar3_OAR›	30882266	-0.02004
27	oar3_OAR›	30885588	-0.02004
27	oar3_OAR›	30888012	-0.02744
27	oar3_OAR›	30898985	-0.0184
27	oar3_OAR›	30902400	-0.02224
27	oar3_OAR›	30907097	-0.02242
27	oar3_OAR›	30907939	-0.01456
27	oar3_OAR›	30915361	0.042169
27	oar3_OAR›	30925130	0.004473
27	oar3_OAR›	30925589	0.012511
27	oar3_OAR›	30950213	0.171219
27	oar3_OAR›	30950713	0.141875
27	oar3_OAR›	30956305	-0.02565
27	oar3_OAR›	30959093	-0.03582
27	oar3_OAR›	30976524	-0.02078
27	oar3_OAR›	30978217	-0.0293
27	oar3_OAR›	30981099	-0.02977
27	oar3_OAR›	30985363	-0.02611
27	oar3_OAR›	30992266	-0.01416
27	oar3_OAR›	30997644	-0.01992
27	oar3_OAR›	31000434	-0.01992
27	oar3_OAR›	31002778	0.022147
27	oar3_OAR›	31003288	-0.02251
27	oar3_OAR›	31004117	-0.02891
27	oar3_OAR›	31016322	0.01236
27	oar3_OAR›	31021564	-0.01

27	oar3_OAR〉	31023377	-0.01856
27	oar3_OAR〉	31024375	-0.02252
27	oar3_OAR〉	31026570	0.013768
27	oar3_OAR〉	31027807	-0.01418
27	oar3_OAR〉	31032254	-0.01
27	oar3_OAR〉	31034734	-0.01418
27	oar3_OAR〉	31035760	-0.01069
27	oar3_OAR〉	31037335	-0.02662
27	oar3_OAR〉	31045013	-0.02778
27	oar3_OAR〉	31046141	0.014923
27	oar3_OAR〉	31049485	-0.01617
27	oar3_OAR〉	31051325	-0.00884
27	oar3_OAR〉	31056619	0.005166
27	oar3_OAR〉	31058554	-0.02216
27	oar3_OAR〉	31062672	0.008806
27	oar3_OAR〉	31067078	-0.02182
27	oar3_OAR〉	31083372	-0.02292
27	oar3_OAR〉	31086173	-0.02307
27	oar3_OAR〉	31096518	-0.02143
27	oar3_OAR〉	31099038	-0.02797
27	oar3_OAR〉	31099701	-0.01739
27	oar3_OAR〉	31107501	-0.032
27	oar3_OAR〉	31107831	-0.032
27	oar3_OAR〉	31108096	-0.02143
27	oar3_OAR〉	31118203	-0.02796
27	oar3_OAR〉	31118430	-0.02782
27	oar3_OAR〉	31132969	-0.01828
27	oar3_OAR〉	31145164	-0.01532
27	oar3_OAR〉	31159011	-0.02375
27	oar3_OAR〉	31163170	0.003987
27	oar3_OAR〉	31171525	-0.02622
27	oar3_OAR〉	31172148	-0.02622
27	oar3_OAR〉	31184512	-0.02432
27	oar3_OAR〉	31190460	-0.0299
27	oar3_OAR〉	31195965	-0.01532
27	oar3_OAR〉	31196397	-0.02551
27	oar3_OAR〉	31198962	-0.00791
27	oar3_OAR〉	31212443	-0.02261
27	oar3_OAR〉	31212469	-0.01581
27	oar3_OAR〉	31221570	-0.0275
27	oar3_OAR〉	31224540	-0.02551
27	oar3_OAR〉	31225478	-0.02861
27	oar3_OAR〉	31262962	0.017556
27	oar3_OAR〉	31273136	-0.02827
27	oar3_OAR〉	31274616	0.0275
27	oar3_OAR〉	31276435	0.076975
27	oar3_OAR〉	31279765	0.019419
27	oar3_OAR〉	31282962	0.076975
27	oar3_OAR〉	31285676	0.031119
27	oar3_OAR〉	31293297	0.057462
27	oar3_OAR〉	31305692	0.161314
27	oar3_OAR〉	31323956	0.03329
27	oar3_OAR〉	31324072	0.032613
27	oar3_OAR〉	31337722	0.067581

27	oar3_OAR›	31378631	0.077637
27	oar3_OAR›	31384984	0.077637
27	oar3_OAR›	31390856	0.077637
27	oar3_OAR›	31393262	0.159328
27	oar3_OAR›	31412863	-0.01486
27	oar3_OAR›	31418319	0.088579
27	oar3_OAR›	31418849	0.105123
27	oar3_OAR›	31434028	-0.01673
27	oar3_OAR›	31435479	0.071358
27	oar3_OAR›	31437315	0.012044
27	oar3_OAR›	31448122	-0.01992
27	oar3_OAR›	31457843	-0.03171
27	oar3_OAR›	31458044	-0.03171
27	oar3_OAR›	31465436	-0.01871
27	oar3_OAR›	31469270	-0.01202
27	oar3_OAR›	31477748	-0.01871
27	oar3_OAR›	31486085	-0.02793
27	oar3_OAR›	31486445	-0.02897
27	oar3_OAR›	31494237	-0.02897
27	oar3_OAR›	31510032	-0.02897
27	oar3_OAR›	31514239	-0.04332
27	oar3_OAR›	31514922	-0.02575
27	oar3_OAR›	31517188	-0.04332
27	oar3_OAR›	31521073	-0.04332
27	oar3_OAR›	31524627	-0.02773
27	oar3_OAR›	31531675	-0.03609
27	oar3_OAR›	31533354	0.030952
27	oar3_OAR›	31538875	0.007039
27	oar3_OAR›	31546810	-0.01673
27	oar3_OAR›	31549108	-0.03679
27	oar3_OAR›	31554988	0.000135
27	oar3_OAR›	31556653	-0.02773
27	oar3_OAR›	31561041	0.008296
27	oar3_OAR›	31564515	0.005679
27	oar3_OAR›	31565726	0.005679
27	oar3_OAR›	31566414	-0.03152
27	oar3_OAR›	31573115	-0.01121
27	oar3_OAR›	31577208	0.008082
27	oar3_OAR›	31579958	0.096142
27	oar3_OAR›	31585870	0.051052
27	oar3_OAR›	31601156	0.051052
27	oar3_OAR›	31608086	0.001263
27	oar3_OAR›	31609071	-0.02645
27	oar3_OAR›	31614706	0.008296
27	oar3_OAR›	31620340	-0.01643
27	oar3_OAR›	31620402	-0.03287
27	oar3_OAR›	31625633	0.008296
27	oar3_OAR›	31629644	-0.03852
27	oar3_OAR›	31633156	-0.02849
27	oar3_OAR›	31635399	-0.00534
27	oar3_OAR›	31640363	-0.02752
27	oar3_OAR›	31649189	0.008296
27	oar3_OAR›	31654776	0.018071
27	oar3_OAR›	31662317	-0.02788

27	oar3_OAR›	31662877	-0.02545
27	oar3_OAR›	31672444	-0.00501
27	oar3_OAR›	31679592	0.045387
27	oar3_OAR›	31682064	-0.01743
27	oar3_OAR›	31682800	0.034967
27	oar3_OAR›	31695957	0.049865
27	oar3_OAR›	31699401	-0.01166
27	oar3_OAR›	31700356	0.0257
27	oar3_OAR›	31705711	0.018942
27	oar3_OAR›	31714885	0.007414
27	oar3_OAR›	31722988	-0.00485
27	oar3_OAR›	31729096	0.00481
27	oar3_OAR›	31732531	-0.03885
27	oar3_OAR›	31733685	-0.04007
27	oar3_OAR›	31735952	-0.04127
27	oar3_OAR›	31762905	NA
27	oar3_OAR›	31763455	-0.02797
27	oar3_OAR›	31773967	-0.02525
27	oar3_OAR›	31779160	-0.02525
27	oar3_OAR›	31780685	0.011653
27	oar3_OAR›	31783643	-0.0203
27	oar3_OAR›	31785382	-0.02525
27	oar3_OAR›	31786933	-0.01607
27	oar3_OAR›	31790470	0.011653
27	oar3_OAR›	31799635	-0.0203
27	oar3_OAR›	31807332	-0.02773
27	oar3_OAR›	31813615	-0.00674
27	oar3_OAR›	31816084	-0.03892
27	oar3_OAR›	31829196	-0.02057
27	oar3_OAR›	31831905	-0.04382
27	oar3_OAR›	31833341	0.030402
27	oar3_OAR›	31856175	-0.02672
27	oar3_OAR›	31861941	-0.02679
27	oar3_OAR›	31866906	-0.03447
27	oar3_OAR›	31867180	-0.03672
27	oar3_OAR›	31868487	-0.03672
27	oar3_OAR›	31877495	0.028803
27	oar3_OAR›	31882309	-0.00984
27	oar3_OAR›	31883113	-0.02486
27	oar3_OAR›	31884008	-0.01527
27	oar3_OAR›	31889164	0.028803
27	oar3_OAR›	31897696	0.028803
27	oar3_OAR›	31898312	-0.025
27	oar3_OAR›	31900980	-0.00252
27	oar3_OAR›	31903732	-0.01655
27	oar3_OAR›	31904266	0.045503
27	oar3_OAR›	31912476	0.016242
27	oar3_OAR›	31918293	0.003466
27	oar3_OAR›	31924910	-0.02505
27	oar3_OAR›	31948778	-0.02545
27	oar3_OAR›	31951685	-0.02545
27	oar3_OAR›	31955337	-0.02545
27	oar3_OAR›	31959631	-0.02545
27	oar3_OAR›	31962028	-0.0219

27	oar3_OAR)	31968992	-0.0219
27	oar3_OAR)	31971937	-0.02236
27	oar3_OAR)	31973688	-0.0286
27	oar3_OAR)	31978616	-0.00148
27	oar3_OAR)	31978934	-0.00148
27	oar3_OAR)	31979242	0.031085
27	oar3_OAR)	31989861	0.007726
27	oar3_OAR)	31998711	0.021891
27	oar3_OAR)	31999230	0.086473
27	oar3_OAR)	32007588	-0.01538
27	oar3_OAR)	32014356	-0.02774
27	oar3_OAR)	32017018	0.053295
27	oar3_OAR)	32017328	-0.02774
27	oar3_OAR)	32018523	0.053295
27	oar3_OAR)	32028151	-0.01815
27	oar3_OAR)	32030847	0.011871
27	oar3_OAR)	32031920	0.05584
27	oar3_OAR)	32033502	0.004461
27	oar3_OAR)	32039387	0.004461
27	oar3_OAR)	32043351	0.022172
27	oar3_OAR)	32046626	-0.02688
27	oar3_OAR)	32051933	-0.01538
27	oar3_OAR)	32052334	-0.01585
27	oar3_OAR)	32055931	-0.01538
27	oar3_OAR)	32063251	-0.01585
27	oar3_OAR)	32065151	-0.01585
27	oar3_OAR)	32070477	-0.03193
27	oar3_OAR)	32076283	-0.01527
27	oar3_OAR)	32078239	-0.0338
27	oar3_OAR)	32079016	-0.03143
27	oar3_OAR)	32082374	-0.00974
27	oar3_OAR)	32083286	-0.01538
27	oar3_OAR)	32088177	0.032582
27	oar3_OAR)	32093952	-0.02746
27	oar3_OAR)	32102249	0.009992
27	oar3_OAR)	32102351	0.009992
27	oar3_OAR)	32102743	0.009992
27	oar3_OAR)	32125089	0.009825
27	oar3_OAR)	32133263	0.026827
27	oar3_OAR)	32133928	NA
27	oar3_OAR)	32136032	-0.00444
27	oar3_OAR)	32148144	-0.00444
27	oar3_OAR)	32151308	-0.00949
27	oar3_OAR)	32162876	-0.01439
27	oar3_OAR)	32170241	-0.01762
27	oar3_OAR)	32178998	-0.02174
27	oar3_OAR)	32186686	-0.0168
27	oar3_OAR)	32189729	8.73E-05
27	oar3_OAR)	32191485	-0.03147
27	oar3_OAR)	32196790	-0.03147
27	oar3_OAR)	32209452	-0.0321
27	oar3_OAR)	32214311	-0.03147
27	oar3_OAR)	32218318	-0.01869
27	oar3_OAR)	32220593	-0.0321

27	oar3_OAR〉	32229188	0.009985
27	oar3_OAR〉	32234061	0.00481
27	oar3_OAR〉	32235222	0.000215
27	oar3_OAR〉	32235833	0.023922
27	oar3_OAR〉	32266278	-0.02757
27	oar3_OAR〉	32266532	-0.03175
27	oar3_OAR〉	32277867	-0.00452
27	oar3_OAR〉	32284591	-0.02974
27	oar3_OAR〉	32292344	0.026097
27	oar3_OAR〉	32294568	0.007039
27	oar3_OAR〉	32298833	0.007039
27	oar3_OAR〉	32307264	-0.01704
27	oar3_OAR〉	32309172	-0.00173
27	oar3_OAR〉	32315849	-0.01303
27	oar3_OAR〉	32318041	-0.00462
27	oar3_OAR〉	32320562	-0.01303
27	oar3_OAR〉	32323871	-0.01303
27	oar3_OAR〉	32351290	0.0069
27	oar3_OAR〉	32354848	-0.02778
27	oar3_OAR〉	32365738	-0.01735
27	oar3_OAR〉	32375948	-0.00575
27	oar3_OAR〉	32393496	-0.02916
27	oar3_OAR〉	32399586	-0.0262
27	oar3_OAR〉	32412758	-0.02778
27	oar3_OAR〉	32430833	-0.02778
27	oar3_OAR〉	32434966	-0.02778
27	oar3_OAR〉	32440297	-0.01735
27	oar3_OAR〉	32443437	-0.02607
27	oar3_OAR〉	32451350	-0.02009
27	oar3_OAR〉	32451747	-0.02778
27	oar3_OAR〉	32456465	0.007497
27	oar3_OAR〉	32461931	-0.01741
27	oar3_OAR〉	32468001	-0.02488
27	oar3_OAR〉	32481433	-0.02664
27	oar3_OAR〉	32483921	-0.00751
27	oar3_OAR〉	32495602	-0.01158
27	oar3_OAR〉	32507314	-0.03142
27	oar3_OAR〉	32507472	-0.03142
27	oar3_OAR〉	32515814	-0.02915
27	oar3_OAR〉	32519796	-0.02712
27	oar3_OAR〉	32527435	-0.0177
27	oar3_OAR〉	32546923	-0.02822
27	oar3_OAR〉	32552985	-0.02512
27	oar3_OAR〉	32557229	-0.01881
27	oar3_OAR〉	32561300	-0.01192
27	oar3_OAR〉	32571824	-0.01423
27	oar3_OAR〉	32578708	-0.02565
27	oar3_OAR〉	32581493	0.008694
27	oar3_OAR〉	32593241	-0.0233
27	oar3_OAR〉	32613495	0.000363
27	oar3_OAR〉	32613858	-0.02458
27	oar3_OAR〉	32620274	0.009617
27	oar3_OAR〉	32621007	-0.00606
27	oar3_OAR〉	32637912	-0.00606

27	oar3_OAR>	32648842	-0.00682
27	oar3_OAR>	32651910	-0.01689
27	oar3_OAR>	32661520	-0.01532
27	oar3_OAR>	32665951	-0.0071
27	oar3_OAR>	32681443	0.000889
27	oar3_OAR>	32684844	-0.0071
27	oar3_OAR>	32687256	-0.0071
27	oar3_OAR>	32693782	-0.00931
27	oar3_OAR>	32698350	0.018804
27	oar3_OAR>	32706635	0.08233
27	oar3_OAR>	32710395	0.023064
27	oar3_OAR>	32726436	0.06208
27	oar3_OAR>	32727767	0.044724
27	oar3_OAR>	32747525	0.044633
27	oar3_OAR>	32749316	0.082222
27	oar3_OAR>	32755063	0.074839
27	oar3_OAR>	32761043	0.020467
27	oar3_OAR>	32765161	0.024247
27	oar3_OAR>	32765630	0.057272
27	oar3_OAR>	32771292	0.020467
27	oar3_OAR>	32772776	NA
27	oar3_OAR>	32775702	0.008806
27	oar3_OAR>	32777799	-0.00349
27	oar3_OAR>	32784857	0.043995
27	oar3_OAR>	32785009	0.043995
27	oar3_OAR>	32788330	0.006793
27	oar3_OAR>	32794687	0.006793
27	oar3_OAR>	32794961	0.018804
27	oar3_OAR>	32798851	-0.01538
27	oar3_OAR>	32807867	0.006793
27	oar3_OAR>	32814583	0.006793
27	oar3_OAR>	32820956	0.020467
27	oar3_OAR>	32821285	0.020467
27	oar3_OAR>	32825874	0.006793
27	oar3_OAR>	32826387	0.020467
27	oar3_OAR>	32841782	0.030434
27	oar3_OAR>	32853806	0.020467
27	oar3_OAR>	32854074	0.081485
27	oar3_OAR>	32854143	0.009572
27	oar3_OAR>	32867530	0.030434
27	oar3_OAR>	32882599	-0.01538
27	oar3_OAR>	32895957	-0.01605
27	oar3_OAR>	32896024	-0.00673
27	oar3_OAR>	32896072	-0.00673
27	oar3_OAR>	32897225	-0.00185
27	oar3_OAR>	32904009	NA
27	oar3_OAR>	32904509	0.030434
27	oar3_OAR>	32907787	NA
27	oar3_OAR>	32918523	0.004917
27	oar3_OAR>	32924896	0.044586
27	oar3_OAR>	32930218	-0.00462
27	oar3_OAR>	32932840	-0.01069
27	oar3_OAR>	32942433	-0.00462
27	oar3_OAR>	32943083	0.093272

27	oar3_OAR›	32943137	0.093272
27	oar3_OAR›	32951693	0.00481
27	oar3_OAR›	32961088	0.08119
27	oar3_OAR›	32965273	-0.00462
27	oar3_OAR›	32980795	0.002868
27	oar3_OAR›	32999833	0.032582
27	oar3_OAR›	33000772	0.00481
27	oar3_OAR›	33004689	-0.02575
27	oar3_OAR›	33009407	0.044403
27	oar3_OAR›	33010480	0.000889
27	oar3_OAR›	33011103	-0.00395
27	oar3_OAR›	33019417	-0.00325
27	oar3_OAR›	33022815	0.141065
27	oar3_OAR›	33024495	0.079352
27	oar3_OAR›	33025710	0.100253
27	oar3_OAR›	33029794	0.042868
27	oar3_OAR›	33038398	-0.02083
27	oar3_OAR›	33039299	-0.01176
27	oar3_OAR›	33055995	0.006079
27	oar3_OAR›	33056377	0.006079
27	oar3_OAR›	33060102	0.096103
27	oar3_OAR›	33066680	0.02963
27	oar3_OAR›	33066743	0.02963
27	oar3_OAR›	33072063	0.042517
27	oar3_OAR›	33081518	0.007039
27	oar3_OAR›	33088197	-0.02921
27	oar3_OAR›	33097649	0.072901
27	oar3_OAR›	33098072	-0.02921
27	oar3_OAR›	33103359	-0.02921
27	oar3_OAR›	33108974	-0.01426
27	oar3_OAR›	33120739	-0.02921
27	oar3_OAR›	33124659	-0.01426
27	oar3_OAR›	33125365	-0.01426
27	oar3_OAR›	33125752	0.072751
27	oar3_OAR›	33130772	-0.00901
27	oar3_OAR›	33131597	0.031577
27	oar3_OAR›	33136612	0.060033
27	oar3_OAR›	33138521	0.043729
27	oar3_OAR›	33142185	0.00481
27	oar3_OAR›	33143618	0.034707
27	oar3_OAR›	33144293	0.067717
27	oar3_OAR›	33148322	0.021215
27	oar3_OAR›	33154142	0.127807
27	oar3_OAR›	33154485	0.127807
27	oar3_OAR›	33160418	0.13002
27	oar3_OAR›	33160989	0.042659
27	oar3_OAR›	33164063	0.111655
27	oar3_OAR›	33169436	0.045498
27	oar3_OAR›	33169507	0.111655
27	oar3_OAR›	33178333	-0.01309
27	oar3_OAR›	33187871	-0.00195
27	oar3_OAR›	33206957	-0.01074
27	oar3_OAR›	33208789	-0.00935
27	oar3_OAR›	33212242	-0.01567

27	oar3_OAR›	33224118	0.021496
27	oar3_OAR›	33224457	0.021496
27	oar3_OAR›	33224622	-0.01074
27	oar3_OAR›	33225123	-0.01074
27	oar3_OAR›	33231101	-0.01074
27	oar3_OAR›	33234051	0.0505
27	oar3_OAR›	33234831	-0.01074
27	oar3_OAR›	33236806	-0.00195
27	oar3_OAR›	33237101	0.105625
27	oar3_OAR›	33248834	-0.02488
27	oar3_OAR›	33249096	-0.02926
27	oar3_OAR›	33249233	-0.00195
27	oar3_OAR›	33258831	0.032223
27	oar3_OAR›	33260028	0.04151
27	oar3_OAR›	33261149	-0.01351
27	oar3_OAR›	33261368	0.043437
27	oar3_OAR›	33268194	0.081173
27	oar3_OAR›	33272569	-0.00882
27	oar3_OAR›	33273146	-0.01373
27	oar3_OAR›	33280463	-0.01737
27	oar3_OAR›	33283093	NA
27	oar3_OAR›	33284280	-0.01737
27	oar3_OAR›	33285524	-0.01737
27	oar3_OAR›	33285671	NA
27	oar3_OAR›	33295659	-0.03248
27	oar3_OAR›	33301576	-0.02055
27	oar3_OAR›	33313485	NA
27	oar3_OAR›	33317087	0.015985
27	oar3_OAR›	33327341	-0.01737
27	oar3_OAR›	33327503	NA
27	oar3_OAR›	33333189	-0.02453
27	oar3_OAR›	33335024	-0.02198
27	oar3_OAR›	33347343	-0.02236
27	oar3_OAR›	33348089	-0.02462
27	oar3_OAR›	33352559	0.021558
27	oar3_OAR›	33359776	0.025955
27	oar3_OAR›	33365288	-0.02049
27	oar3_OAR›	33365916	-0.00395
27	oar3_OAR›	33368715	0.000577
27	oar3_OAR›	33370916	0.088289
27	oar3_OAR›	33371214	0.007181
27	oar3_OAR›	33375826	-0.02049
27	oar3_OAR›	33381410	-0.02049
27	oar3_OAR›	33383315	0.039638
27	oar3_OAR›	33392996	0.125097
27	oar3_OAR›	33397821	-0.00395
27	oar3_OAR›	33405627	-0.02049
27	oar3_OAR›	33415541	0.095404
27	oar3_OAR›	33416875	0.045915
27	oar3_OAR›	33420833	0.0672
27	oar3_OAR›	33424561	0.102808
27	oar3_OAR›	33428933	0.036226
27	oar3_OAR›	33432704	0.103129
27	oar3_OAR›	33438681	0.000889

27	oar3_OAR›	33441277	0.036226
27	oar3_OAR›	33441794	0.036226
27	oar3_OAR›	33454264	0.000889
27	oar3_OAR›	33462864	0.033282
27	oar3_OAR›	33462934	0.103129
27	oar3_OAR›	33464569	0.036226
27	oar3_OAR›	33469109	0.000889
27	oar3_OAR›	33474758	0.088731
27	oar3_OAR›	33482705	-0.01069
27	oar3_OAR›	33485334	-0.01069
27	oar3_OAR›	33493285	0.088731
27	oar3_OAR›	33497175	-0.01069
27	oar3_OAR›	33497285	0.036226
27	oar3_OAR›	33501124	-0.01069
27	oar3_OAR›	33503396	-0.01069
27	oar3_OAR›	33506334	0.141096
27	oar3_OAR›	33510156	0.066666
27	oar3_OAR›	33516125	-0.01069
27	oar3_OAR›	33524515	0.141096
27	oar3_OAR›	33532343	0.141096
27	oar3_OAR›	33535486	0.122761
27	oar3_OAR›	33540605	-0.01069
27	oar3_OAR›	33543991	0.036226
27	oar3_OAR›	33548123	0.000889
27	oar3_OAR›	33549705	0.141096
27	oar3_OAR›	33558873	0.16768
27	oar3_OAR›	33562610	-0.03332
27	oar3_OAR›	33563668	0.028519
27	oar3_OAR›	33566703	0.036271
27	oar3_OAR›	33574128	5.87E-06
27	oar3_OAR›	33575806	0.015702
27	oar3_OAR›	33578471	0.026199
27	oar3_OAR›	33595245	0.093774
27	oar3_OAR›	33596226	0.071421
27	oar3_OAR›	33596877	0.071421
27	oar3_OAR›	33596948	0.071421
27	oar3_OAR›	33602021	-0.03688
27	oar3_OAR›	33631559	0.006407
27	oar3_OAR›	33637436	########
27	oar3_OAR›	33646278	0.006407
27	oar3_OAR›	33647463	########
27	oar3_OAR›	33650287	0.045521
27	oar3_OAR›	33656407	-0.0178
27	oar3_OAR›	33660486	-0.00017
27	oar3_OAR›	33662007	-0.02184
27	oar3_OAR›	33662324	-0.00017
27	oar3_OAR›	33686482	-0.02759
27	oar3_OAR›	33686679	0.067699
27	oar3_OAR›	33690216	-0.02637
27	oar3_OAR›	33695481	0.027516
27	oar3_OAR›	33696342	0.027516
27	oar3_OAR›	33699805	-0.02184
27	oar3_OAR›	33705179	-0.02759
27	oar3_OAR›	33710718	-0.02759

27	oar3_OAR〉	33713137	-0.02759
27	oar3_OAR〉	33714214	-0.02497
27	oar3_OAR〉	33720887	-0.02497
27	oar3_OAR〉	33721644	-0.00571
27	oar3_OAR〉	33725853	0.000377
27	oar3_OAR〉	33726311	-0.02184
27	oar3_OAR〉	33746827	0.053465
27	oar3_OAR〉	33747568	0.053465
27	oar3_OAR〉	33750332	0.053465
27	oar3_OAR〉	33756947	-0.0344
27	oar3_OAR〉	33757732	-0.0344
27	oar3_OAR〉	33758540	0.053465
27	oar3_OAR〉	33768987	-0.0264
27	oar3_OAR〉	33771994	-0.00502
27	oar3_OAR〉	33787567	0.013119
27	oar3_OAR〉	33795721	-0.00325
27	oar3_OAR〉	33795873	0.052755
27	oar3_OAR〉	33815894	0.005534
27	oar3_OAR〉	33816909	0.015525
27	oar3_OAR〉	33817082	0.014143
27	oar3_OAR〉	33822276	-0.02375
27	oar3_OAR〉	33822853	0.015525
27	oar3_OAR〉	33823886	0.005534
27	oar3_OAR〉	33825282	0.015525
27	oar3_OAR〉	33829188	0.015525
27	oar3_OAR〉	33831141	0.076187
27	oar3_OAR〉	33833196	-0.02375
27	oar3_OAR〉	33834966	0.027467
27	oar3_OAR〉	33876108	0.00623
27	oar3_OAR〉	33876180	-0.02375
27	oar3_OAR〉	33876273	-0.02375
27	oar3_OAR〉	33884148	0.030975
27	oar3_OAR〉	33890706	-0.00398
27	oar3_OAR〉	33896097	0.049959
27	oar3_OAR〉	33904825	0.06803
27	oar3_OAR〉	33905336	-0.02375
27	oar3_OAR〉	33910668	-0.00127
27	oar3_OAR〉	33911148	0.061695
27	oar3_OAR〉	33916500	0.061695
27	oar3_OAR〉	33931717	0.024041
27	oar3_OAR〉	33931935	0.03187
27	oar3_OAR〉	33932086	0.024041
27	oar3_OAR〉	33938463	0.032012
27	oar3_OAR〉	33938464	0.028027
27	oar3_OAR〉	33944879	0.011389
27	oar3_OAR〉	33945401	0.032012
27	oar3_OAR〉	33958885	0.028027
27	oar3_OAR〉	33960165	0.0379
27	oar3_OAR〉	33964064	-0.0049
27	oar3_OAR〉	33965880	0.072812
27	oar3_OAR〉	33972142	0.000889
27	oar3_OAR〉	33983323	0.012756
27	oar3_OAR〉	33983980	0.035537
27	oar3_OAR〉	34007102	0.01584

27	oar3_OAR3	34007565	-0.0168
27	oar3_OAR3	34007811	0.022167
27	oar3_OAR3	34016148	-0.01416
27	oar3_OAR3	34020511	-0.01416
27	oar3_OAR3	34021470	-0.00606
27	oar3_OAR3	34021958	-0.02217
27	oar3_OAR3	34034330	-0.02162
27	oar3_OAR3	34045854	-0.02162
27	oar3_OAR3	34047593	-0.01726
27	oar3_OAR3	34053025	-0.0006
27	oar3_OAR3	34056025	-0.02182
27	oar3_OAR3	34056301	0.001274
27	oar3_OAR3	34063217	-0.02162
27	oar3_OAR3	34066066	-0.00915
27	oar3_OAR3	34077637	0.003806
27	oar3_OAR3	34078567	0.003806
27	oar3_OAR3	34078596	-0.01815
27	oar3_OAR3	34079332	-0.01069
27	oar3_OAR3	34084613	0.00563
27	oar3_OAR3	34088072	-0.00865
27	oar3_OAR3	34089242	-0.00865
27	oar3_OAR3	34089601	-0.01069
27	oar3_OAR3	34094976	-0.01069
27	oar3_OAR3	34098345	0.006079
27	oar3_OAR3	34100307	0.020906
27	oar3_OAR3	34105482	0.000889
27	oar3_OAR3	34108596	-0.0206
27	oar3_OAR3	34110683	-0.0206
27	oar3_OAR3	34118288	0.000889
27	oar3_OAR3	34118751	-0.0014
27	oar3_OAR3	34122377	-0.02539
27	oar3_OAR3	34122752	-0.02539
27	oar3_OAR3	34123881	0.000889
27	oar3_OAR3	34129988	-0.0029
27	oar3_OAR3	34130041	-0.0029
27	oar3_OAR3	34133564	-0.01373
27	oar3_OAR3	34136248	0.008694
27	oar3_OAR3	34142709	0.026055
27	oar3_OAR3	34145777	0.063224
27	oar3_OAR3	34151343	0.063224
27	oar3_OAR3	34157056	0.096319
27	oar3_OAR3	34159273	-0.01588
27	oar3_OAR3	34162720	0.004601
27	oar3_OAR3	34164255	0.000135
27	oar3_OAR3	34164844	0.088607
27	oar3_OAR3	34169670	-0.01121
27	oar3_OAR3	34173484	-0.00693
27	oar3_OAR3	34173894	0.000889
27	oar3_OAR3	34185249	-0.01341
27	oar3_OAR3	34189655	-0.02582
27	oar3_OAR3	34189693	0.007263
27	oar3_OAR3	34195787	-0.02582
27	oar3_OAR3	34197375	-0.02292
27	oar3_OAR3	34197491	-0.01587

27	oar3_OAR›	34202200	0.056948
27	oar3_OAR›	34210084	-0.00933
27	oar3_OAR›	34215440	-0.00933
27	oar3_OAR›	34216290	0.030998
27	oar3_OAR›	34221406	0.006708
27	oar3_OAR›	34228010	0.006708
27	oar3_OAR›	34228493	-0.0262
27	oar3_OAR›	34234382	-0.00933
27	oar3_OAR›	34234589	0.029883
27	oar3_OAR›	34234836	-0.03166
27	oar3_OAR›	34235984	-0.02818
27	oar3_OAR›	34239966	0.014843
27	oar3_OAR›	34245909	-0.02947
27	oar3_OAR›	34250049	0.007039
27	oar3_OAR›	34251722	0.027516
27	oar3_OAR›	34251847	0.136861
27	oar3_OAR›	34260819	NA
27	oar3_OAR›	34261037	0.023823
27	oar3_OAR›	34269890	-0.00457
27	oar3_OAR›	34271202	0.044806
27	oar3_OAR›	34273889	-0.00758
27	oar3_OAR›	34279627	0.003466
27	oar3_OAR›	34304524	0.032976
27	oar3_OAR›	34318720	0.003466
27	oar3_OAR›	34318746	0.048875
27	oar3_OAR›	34325334	0.048875
27	oar3_OAR›	34343386	-0.01445
27	oar3_OAR›	34360465	0.030053
27	oar3_OAR›	34363403	0.048875
27	oar3_OAR›	34364483	0.003466
27	oar3_OAR›	34379715	-0.01762
27	oar3_OAR›	34380035	-0.01762
27	oar3_OAR›	34391248	0.011402
27	oar3_OAR›	34404448	-0.01762
27	oar3_OAR›	34404530	-0.01762
27	oar3_OAR›	34418291	-0.01762
27	oar3_OAR›	34418550	-0.01762
27	oar3_OAR›	34419567	-0.01762
27	oar3_OAR›	34427823	-0.01762
27	oar3_OAR›	34431518	0.023698
27	oar3_OAR›	34432970	-0.01762
27	oar3_OAR›	34435866	0.048875
27	oar3_OAR›	34444103	-0.01762
27	oar3_OAR›	34445998	-0.01762
27	oar3_OAR›	34447903	-0.01762
27	oar3_OAR›	34475403	0.003466
27	oar3_OAR›	34504386	0.037433
27	oar3_OAR›	34509823	-0.02575
27	oar3_OAR›	34520886	0.138138
27	oar3_OAR›	34531933	-0.02575
27	oar3_OAR›	34537746	-0.02575
27	oar3_OAR›	34550664	-0.02807
27	oar3_OAR›	34552855	-0.02555
27	oar3_OAR›	34553352	-0.02388

27	oar3_OAR›	34554233	-0.01854
27	oar3_OAR›	34556279	-0.02388
27	oar3_OAR›	34556454	-0.03113
27	oar3_OAR›	34556581	-0.02575
27	oar3_OAR›	34564812	-0.02899
27	oar3_OAR›	34569061	-0.0262
27	oar3_OAR›	34569195	-0.02367
27	oar3_OAR›	34569466	0.029067
27	oar3_OAR›	34587559	-0.02989
27	oar3_OAR›	34594583	0.127748
27	oar3_OAR›	34600240	0.108198
27	oar3_OAR›	34600691	0.108198
27	oar3_OAR›	34607099	-0.02395
27	oar3_OAR›	34610171	0.006684
27	oar3_OAR›	34612181	-0.0169
27	oar3_OAR›	34612680	-0.02236
27	oar3_OAR›	34619629	-0.02413
27	oar3_OAR›	34623794	0.047247
27	oar3_OAR›	34630925	-0.01859
27	oar3_OAR›	34632773	-0.01758
27	oar3_OAR›	34640681	-0.02405
27	oar3_OAR›	34645592	0.018326
27	oar3_OAR›	34656572	0.016767
27	oar3_OAR›	34674649	-0.02324
27	oar3_OAR›	34674766	-0.02324
27	oar3_OAR›	34676575	0.029964
27	oar3_OAR›	34682927	0.006556
27	oar3_OAR›	34695775	0.006556
27	oar3_OAR›	34695837	0.003095
27	oar3_OAR›	34697539	0.006556
27	oar3_OAR›	34707288	0.000655
27	oar3_OAR›	34708562	0.006662
27	oar3_OAR›	34720759	0.011345
27	oar3_OAR›	34739558	-0.00161
27	oar3_OAR›	34740557	-0.00926
27	oar3_OAR›	34742352	-0.00118
27	oar3_OAR›	34776937	-0.007
27	oar3_OAR›	34832752	0.082222
27	oar3_OAR›	34845905	0.174262
27	oar3_OAR›	34853643	0.043718
27	oar3_OAR›	34854223	0.061653
27	oar3_OAR›	34855555	0.061653
27	oar3_OAR›	34857252	0.098196
27	oar3_OAR›	34861934	0.05724
27	oar3_OAR›	34866549	0.082222
27	oar3_OAR›	34867777	0.101064
27	oar3_OAR›	34871534	0.013189
27	oar3_OAR›	34882998	0.06558
27	oar3_OAR›	34883391	0.06558
27	oar3_OAR›	34885555	0.052764
27	oar3_OAR›	34887803	-0.03382
27	oar3_OAR›	34908635	NA
27	oar3_OAR›	34908942	0.174618
27	oar3_OAR›	34916112	NA

27	oar3_OAR›	34916442	-0.01992
27	oar3_OAR›	34927666	0.074729
27	oar3_OAR›	34928617	0.000889
27	oar3_OAR›	34932307	0.109075
27	oar3_OAR›	34937475	0.065714
27	oar3_OAR›	34943834	0.1047
27	oar3_OAR›	34945980	0.113361
27	oar3_OAR›	34946046	-0.00187
27	oar3_OAR›	34957568	0.027296
27	oar3_OAR›	34958673	0.014568
27	oar3_OAR›	34966554	0.092421
27	oar3_OAR›	34969152	0.01726
27	oar3_OAR›	34975950	0.023749
27	oar3_OAR›	34985445	-0.00901
27	oar3_OAR›	34985457	0.000239
27	oar3_OAR›	34991014	NA
27	oar3_OAR›	34992445	0.109449
27	oar3_OAR›	34994384	NA
27	oar3_OAR›	35004968	NA
27	oar3_OAR›	35006008	NA
27	oar3_OAR›	35012523	-0.03171
27	oar3_OAR›	35013184	NA
27	oar3_OAR›	35018409	0.067927
27	oar3_OAR›	35024258	0.00686
27	oar3_OAR›	35030252	NA
27	oar3_OAR›	35032404	0.001648
27	oar3_OAR›	35033480	-0.00524
27	oar3_OAR›	35035377	0.022411
27	oar3_OAR›	35043895	0.039481
27	oar3_OAR›	35057867	-0.00961
27	oar3_OAR›	35068038	-0.00643
27	oar3_OAR›	35071631	0.076155
27	oar3_OAR›	35076626	-0.02049
27	oar3_OAR›	35080429	0.006106
27	oar3_OAR›	35082266	-0.01426
27	oar3_OAR›	35094014	-0.01338
27	oar3_OAR›	35094523	0.065168
27	oar3_OAR›	35096421	0.059594
27	oar3_OAR›	35097520	0.004594
27	oar3_OAR›	35106787	0.048082
27	oar3_OAR›	35111352	-0.0042
27	oar3_OAR›	35112239	-0.01567
27	oar3_OAR›	35116530	-0.0042
27	oar3_OAR›	35119047	-0.01789
27	oar3_OAR›	35121970	0.01975
27	oar3_OAR›	35126642	0.040046
27	oar3_OAR›	35130517	0.060528
27	oar3_OAR›	35131581	-0.00158
27	oar3_OAR›	35137493	0.004857
27	oar3_OAR›	35142064	-0.0353
27	oar3_OAR›	35142414	0.004857
27	oar3_OAR›	35159379	0.004857
27	oar3_OAR›	35160148	0.06582
27	oar3_OAR›	35160952	0.06669

27	oar3_OAR〉	35163737	0.03729
27	oar3_OAR〉	35170886	0.069377
27	oar3_OAR〉	35184224	0.004857
27	oar3_OAR〉	35186291	-0.02974
27	oar3_OAR〉	35187585	0.035747
27	oar3_OAR〉	35192783	-0.02348
27	oar3_OAR〉	35196901	0.029789
27	oar3_OAR〉	35202419	0.028803
27	oar3_OAR〉	35202744	0.025488
27	oar3_OAR〉	35210465	0.05614
27	oar3_OAR〉	35210678	NA
27	oar3_OAR〉	35214177	0.076445
27	oar3_OAR〉	35215648	-0.02049
27	oar3_OAR〉	35216787	0.093947
27	oar3_OAR〉	35220792	-0.01866
27	oar3_OAR〉	35226272	-0.02049
27	oar3_OAR〉	35232730	0.030199
27	oar3_OAR〉	35238070	-0.02049
27	oar3_OAR〉	35244958	0.013749
27	oar3_OAR〉	35246600	-0.02325
27	oar3_OAR〉	35249944	0.046998
27	oar3_OAR〉	35252345	0.073957
27	oar3_OAR〉	35256131	0.099448
27	oar3_OAR〉	35261501	0.038645
27	oar3_OAR〉	35262077	0.038645
27	oar3_OAR〉	35267696	0.170734
27	oar3_OAR〉	35274264	0.059087
27	oar3_OAR〉	35279845	-0.00041
27	oar3_OAR〉	35287990	0.015597
27	oar3_OAR〉	35291645	-0.02413
27	oar3_OAR〉	35296744	-0.00712
27	oar3_OAR〉	35300840	0.154791
27	oar3_OAR〉	35316307	0.199216
27	oar3_OAR〉	35316812	0.121347
27	oar3_OAR〉	35317821	-0.00847
27	oar3_OAR〉	35321634	0.160109
27	oar3_OAR〉	35325620	-0.00847
27	oar3_OAR〉	35329046	0.154791
27	oar3_OAR〉	35330327	0.265929
27	oar3_OAR〉	35338248	########
27	oar3_OAR〉	35362438	0.017272
27	oar3_OAR〉	35364618	0.10155
27	oar3_OAR〉	35368707	0.048267
27	oar3_OAR〉	35374772	0.032268
27	oar3_OAR〉	35380349	0.032268
27	oar3_OAR〉	35382132	0.015777
27	oar3_OAR〉	35387601	-0.02049
27	oar3_OAR〉	35398410	0.010077
27	oar3_OAR〉	35398655	0.010077
27	oar3_OAR〉	35401404	0.017727
27	oar3_OAR〉	35405912	-0.01121
27	oar3_OAR〉	35409555	-0.00934
27	oar3_OAR〉	35413486	-0.00712
27	oar3_OAR〉	35413576	-0.03502

27	oar3_OAR?	35414520	-0.01059
27	oar3_OAR?	35418734	-0.02049
27	oar3_OAR?	35424696	0.0004
27	oar3_OAR?	35425249	-0.01063
27	oar3_OAR?	35426567	0.070121
27	oar3_OAR?	35426784	0.070121
27	oar3_OAR?	35436906	0.0061
27	oar3_OAR?	35439738	-0.03673
27	oar3_OAR?	35441166	0.048875
27	oar3_OAR?	35444953	-0.03326
27	oar3_OAR?	35447812	-0.03068
27	oar3_OAR?	35450341	0.064242
27	oar3_OAR?	35451812	-0.01755
27	oar3_OAR?	35457053	-0.03515
27	oar3_OAR?	35457888	-0.03515
27	oar3_OAR?	35462295	-0.02793
27	oar3_OAR?	35464361	0.064242
27	oar3_OAR?	35466057	-0.0211
27	oar3_OAR?	35470127	0.175629
27	oar3_OAR?	35476428	0.027864
27	oar3_OAR?	35479975	-0.02485
27	oar3_OAR?	35490800	-0.01943
27	oar3_OAR?	35491076	NA
27	oar3_OAR?	35497033	-0.00673
27	oar3_OAR?	35507479	0.084643
27	oar3_OAR?	35523639	NA
27	oar3_OAR?	35542759	-0.02184
27	oar3_OAR?	35542921	0.005972
27	oar3_OAR?	35543039	-0.03383
27	oar3_OAR?	35543906	-0.02807
27	oar3_OAR?	35557130	-0.00673
27	oar3_OAR?	35562567	-0.02153
27	oar3_OAR?	35565277	-0.02926
27	oar3_OAR?	35567759	-0.01581
27	oar3_OAR?	35573744	0.108785
27	oar3_OAR?	35578942	0.108785
27	oar3_OAR?	35579059	0.108785
27	oar3_OAR?	35583344	0.043805
27	oar3_OAR?	35592099	0.037433
27	oar3_OAR?	35595573	0.020399
27	oar3_OAR?	35601656	0.013682
27	oar3_OAR?	35606351	0.028743
27	oar3_OAR?	35607286	0.069468
27	oar3_OAR?	35612348	0.003185
27	oar3_OAR?	35612431	0.069468
27	oar3_OAR?	35615180	0.020933
27	oar3_OAR?	35622064	-0.01049
27	oar3_OAR?	35623207	0.033946
27	oar3_OAR?	35624286	0.056205
27	oar3_OAR?	35625218	-0.00546
27	oar3_OAR?	35629951	0.049985
27	oar3_OAR?	35635238	-0.01896
27	oar3_OAR?	35635294	0.030477
27	oar3_OAR?	35643043	-0.02926

27	oar3_OAR〉	35651859	-0.02575
27	oar3_OAR〉	35652902	-0.02575
27	oar3_OAR〉	35657083	0.027559
27	oar3_OAR〉	35680946	-0.00195
27	oar3_OAR〉	35686478	0.003593
27	oar3_OAR〉	35691587	0.003593
27	oar3_OAR〉	35700431	-0.02049
27	oar3_OAR〉	35702302	-0.02049
27	oar3_OAR〉	35712059	-0.02236
27	oar3_OAR〉	35713720	-0.00094
27	oar3_OAR〉	35715799	0.009065
27	oar3_OAR〉	35720784	0.000889
27	oar3_OAR〉	35722435	-0.01872
27	oar3_OAR〉	35726455	-0.01498
27	oar3_OAR〉	35727119	-0.01412
27	oar3_OAR〉	35732269	-0.01412
27	oar3_OAR〉	35736820	-0.02153
27	oar3_OAR〉	35739567	0.025509
27	oar3_OAR〉	35741839	-0.01225
27	oar3_OAR〉	35752208	-0.02287
27	oar3_OAR〉	35759196	-0.02287
27	oar3_OAR〉	35763388	-0.02129
27	oar3_OAR〉	35776593	-0.00658
27	oar3_OAR〉	35776662	-0.01069
27	oar3_OAR〉	35777449	-0.01558
27	oar3_OAR〉	35789567	-0.00294
27	oar3_OAR〉	35790232	-0.0115
27	oar3_OAR〉	35795682	0.012115
27	oar3_OAR〉	35801442	-0.012
27	oar3_OAR〉	35801610	0.061088
27	oar3_OAR〉	35802596	0.005018
27	oar3_OAR〉	35810622	-0.02151
27	oar3_OAR〉	35810999	-0.02104
27	oar3_OAR〉	35817637	0.022123
27	oar3_OAR〉	35823566	0.037635
27	oar3_OAR〉	35826048	0.060364
27	oar3_OAR〉	35828939	-0.0201
27	oar3_OAR〉	35830439	0.002327
27	oar3_OAR〉	35831243	-0.03471
27	oar3_OAR〉	35848785	0.043437
27	oar3_OAR〉	35857691	-0.0259
27	oar3_OAR〉	35858802	-0.02721
27	oar3_OAR〉	35858879	-0.03175
27	oar3_OAR〉	35865938	0.028738
27	oar3_OAR〉	35866944	0.003802
27	oar3_OAR〉	35869053	0.003484
27	oar3_OAR〉	35869250	-0.0041
27	oar3_OAR〉	35873499	-0.02063
27	oar3_OAR〉	35882790	-0.01595
27	oar3_OAR〉	35884471	0.005167
27	oar3_OAR〉	35893608	0.00704
27	oar3_OAR〉	35894347	0.064227
27	oar3_OAR〉	35899195	0.042659
27	oar3_OAR〉	35902457	0.051229

27	oar3_OAR›	35908704	0.017412
27	oar3_OAR›	35908892	-0.01186
27	oar3_OAR›	35909324	0.02067
27	oar3_OAR›	35916906	0.018772
27	oar3_OAR›	35920158	0.004914
27	oar3_OAR›	35921653	-0.0249
27	oar3_OAR›	35921829	-0.0249
27	oar3_OAR›	35922060	-0.0185
27	oar3_OAR›	35927867	0.079604
27	oar3_OAR›	35932291	0.03632
27	oar3_OAR›	35938715	0.031469
27	oar3_OAR›	35951371	-0.00575
27	oar3_OAR›	35953513	-0.01117
27	oar3_OAR›	35954473	0.003611
27	oar3_OAR›	35958975	-0.0284
27	oar3_OAR›	35960038	0.00222
27	oar3_OAR›	35966090	0.059087
27	oar3_OAR›	35966190	0.00091
27	oar3_OAR›	35970399	0.032517
27	oar3_OAR›	35980731	0.006308
27	oar3_OAR›	35981224	0.041494
27	oar3_OAR›	35981464	-0.00895
27	oar3_OAR›	35981801	-0.02063
27	oar3_OAR›	35989682	0.065512
27	oar3_OAR›	35989987	0.025112
27	oar3_OAR›	35993448	-0.00056
27	oar3_OAR›	36003815	0.030192
27	oar3_OAR›	36006412	-0.00488
27	oar3_OAR›	36010482	-0.0192
27	oar3_OAR›	36010826	0.046532
27	oar3_OAR›	36014713	-0.00501
27	oar3_OAR›	36021300	-0.04077
27	oar3_OAR›	36021558	-0.03354
27	oar3_OAR›	36025382	-0.01809
27	oar3_OAR›	36025718	-0.02801
27	oar3_OAR›	36027906	0.043851
27	oar3_OAR›	36042340	-0.01862
27	oar3_OAR›	36047809	-0.01338
27	oar3_OAR›	36050679	-0.0193
27	oar3_OAR›	36053521	0.009734
27	oar3_OAR›	36053785	-0.02017
27	oar3_OAR›	36059821	0.015365
27	oar3_OAR›	36061172	0.098049
27	oar3_OAR›	36064515	-0.00596
27	oar3_OAR›	36067058	-0.01423
27	oar3_OAR›	36069020	-0.00953
27	oar3_OAR›	36072899	0.001531
27	oar3_OAR›	36073090	-0.01866
27	oar3_OAR›	36080595	0.002184
27	oar3_OAR›	36080870	0.002184
27	oar3_OAR›	36086640	0.017879
27	oar3_OAR›	36089737	0.017879
27	oar3_OAR›	36093591	0.059259
27	oar3_OAR›	36096170	-0.03231

27	oar3_OAR⟩	36098762	-0.00075
27	oar3_OAR⟩	36102400	-0.00376
27	oar3_OAR⟩	36103707	-0.00075
27	oar3_OAR⟩	36109094	0.047752
27	oar3_OAR⟩	36111543	0.053341
27	oar3_OAR⟩	36112121	0.011808
27	oar3_OAR⟩	36117027	-0.02856
27	oar3_OAR⟩	36120448	0.029788
27	oar3_OAR⟩	36121559	0.029788
27	oar3_OAR⟩	36128729	0.071027
27	oar3_OAR⟩	36129885	-0.02958
27	oar3_OAR⟩	36132327	0.000889
27	oar3_OAR⟩	36144758	-0.01338
27	oar3_OAR⟩	36152481	0.044645
27	oar3_OAR⟩	36157231	0.000889
27	oar3_OAR⟩	36163410	0.007237
27	oar3_OAR⟩	36163508	-0.02375
27	oar3_OAR⟩	36176200	-0.02348
27	oar3_OAR⟩	36182954	-0.0315
27	oar3_OAR⟩	36185026	-0.02744
27	oar3_OAR⟩	36188746	0.074002
27	oar3_OAR⟩	36190860	-0.00556
27	oar3_OAR⟩	36197148	0.012529
27	oar3_OAR⟩	36197755	0.012308
27	oar3_OAR⟩	36204412	0.001987
27	oar3_OAR⟩	36205319	0.002277
27	oar3_OAR⟩	36214455	0.015455
27	oar3_OAR⟩	36216748	-0.02577
27	oar3_OAR⟩	36220916	-0.03032
27	oar3_OAR⟩	36225528	-0.025
27	oar3_OAR⟩	36230164	0.05704
27	oar3_OAR⟩	36230890	0.05704
27	oar3_OAR⟩	36238930	0.116377
27	oar3_OAR⟩	36240125	0.043366
27	oar3_OAR⟩	36242995	-0.0143
27	oar3_OAR⟩	36248203	-0.00979
27	oar3_OAR⟩	36251689	0.065627
27	oar3_OAR⟩	36259479	0.021889
27	oar3_OAR⟩	36259528	-0.02162
27	oar3_OAR⟩	36264514	-0.01415
27	oar3_OAR⟩	36265642	-0.01123
27	oar3_OAR⟩	36267493	0.044224
27	oar3_OAR⟩	36269593	0.034627
27	oar3_OAR⟩	36275990	-0.02582
27	oar3_OAR⟩	36277238	0.088887
27	oar3_OAR⟩	36280286	0.084199
27	oar3_OAR⟩	36281650	-0.01222
27	oar3_OAR⟩	36282435	-0.02209
27	oar3_OAR⟩	36291603	0.009985
27	oar3_OAR⟩	36292826	0.009985
27	oar3_OAR⟩	36302602	0.032125
27	oar3_OAR⟩	36305112	0.02643
27	oar3_OAR⟩	36306514	0.02643
27	oar3_OAR⟩	36308613	0.008723

27	oar3_OAR〉	36316333	-0.00163
27	oar3_OAR〉	36317931	-0.02707
27	oar3_OAR〉	36319105	0.024437
27	oar3_OAR〉	36319108	0.018032
27	oar3_OAR〉	36326103	0.029081
27	oar3_OAR〉	36329046	0.069468
27	oar3_OAR〉	36329628	0.057858
27	oar3_OAR〉	36329775	-0.00197
27	oar3_OAR〉	36337764	-0.02807
27	oar3_OAR〉	36340317	0.026097
27	oar3_OAR〉	36342619	-0.02577
27	oar3_OAR〉	36349324	-0.00404
27	oar3_OAR〉	36350963	0.037899
27	oar3_OAR〉	36356925	0.119394
27	oar3_OAR〉	36370410	0.02183
27	oar3_OAR〉	36371863	0.098072
27	oar3_OAR〉	36376218	0.098462
27	oar3_OAR〉	36376288	0.065275
27	oar3_OAR〉	36376931	0.098462
27	oar3_OAR〉	36381255	-0.0148
27	oar3_OAR〉	36386715	0.144576
27	oar3_OAR〉	36390571	0.013072
27	oar3_OAR〉	36390682	0.010744
27	oar3_OAR〉	36394231	0.066736
27	oar3_OAR〉	36403664	0.004943
27	oar3_OAR〉	36405655	0.023788
27	oar3_OAR〉	36409191	0.064242
27	oar3_OAR〉	36409357	-0.01352
27	oar3_OAR〉	36415504	-0.01775
27	oar3_OAR〉	36417225	-0.0071
27	oar3_OAR〉	36422812	-0.0071
27	oar3_OAR〉	36426576	0.05565
27	oar3_OAR〉	36429250	0.064242
27	oar3_OAR〉	36430369	0.050776
27	oar3_OAR〉	36432619	0.080702
27	oar3_OAR〉	36435351	0.105766
27	oar3_OAR〉	36436492	0.152613
27	oar3_OAR〉	36444516	-0.01165
27	oar3_OAR〉	36446774	-0.02259
27	oar3_OAR〉	36448899	-0.02788
27	oar3_OAR〉	36454904	-0.02577
27	oar3_OAR〉	36460066	-0.02487
27	oar3_OAR〉	36461278	-0.02695
27	oar3_OAR〉	36464008	-0.01779
27	oar3_OAR〉	36469978	-0.00427
27	oar3_OAR〉	36474269	0.038079
27	oar3_OAR〉	36476053	0.028961
27	oar3_OAR〉	36476890	0.100281
27	oar3_OAR〉	36482991	0.024109
27	oar3_OAR〉	36483221	0.021499
27	oar3_OAR〉	36484219	0.040274
27	oar3_OAR〉	36488331	0.074647
27	oar3_OAR〉	36489351	0.032901
27	oar3_OAR〉	36494561	0.072642

27	oar3_OAR›	36497789	0.158075
27	oar3_OAR›	36499387	0.134496
27	oar3_OAR›	36509852	0.007973
27	oar3_OAR›	36510046	0.019136
27	oar3_OAR›	36516918	-0.00508
27	oar3_OAR›	36521298	0.113016
27	oar3_OAR›	36522966	0.036764
27	oar3_OAR›	36524505	-0.03521
27	oar3_OAR›	36528563	0.009324
27	oar3_OAR›	36535049	0.018601
27	oar3_OAR›	36535105	0.017279
27	oar3_OAR›	36538466	0.006556
27	oar3_OAR›	36541671	0.001047
27	oar3_OAR›	36542756	-0.00462
27	oar3_OAR›	36546832	0.058184
27	oar3_OAR›	36547070	0.058184
27	oar3_OAR›	36548261	0.001047
27	oar3_OAR›	36551757	0.078896
27	oar3_OAR›	36558673	-0.02834
27	oar3_OAR›	36562782	0.005869
27	oar3_OAR›	36570779	-0.02619
27	oar3_OAR›	36572426	-0.02283
27	oar3_OAR›	36573687	0.005915
27	oar3_OAR›	36577682	0.00481
27	oar3_OAR›	36583839	0.122375
27	oar3_OAR›	36583847	0.062295
27	oar3_OAR›	36596995	0.035684
27	oar3_OAR›	36597922	-0.02393
27	oar3_OAR›	36600212	0.026763
27	oar3_OAR›	36609123	-0.01995
27	oar3_OAR›	36613531	-0.00584
27	oar3_OAR›	36619149	0.018466
27	oar3_OAR›	36625332	0.017742
27	oar3_OAR›	36630744	-0.0168
27	oar3_OAR›	36636103	-0.00788
27	oar3_OAR›	36639625	0.006752
27	oar3_OAR›	36639650	0.085835
27	oar3_OAR›	36644425	0.077823
27	oar3_OAR›	36647641	0.025477
27	oar3_OAR›	36647969	0.046389
27	oar3_OAR›	36654653	-0.00976
27	oar3_OAR›	36657888	-0.01567
27	oar3_OAR›	36658302	0.204214
27	oar3_OAR›	36661365	0.08144
27	oar3_OAR›	36670760	0.052049
27	oar3_OAR›	36673060	0.10724
27	oar3_OAR›	36674329	0.092063
27	oar3_OAR›	36684451	-0.00776
27	oar3_OAR›	36685178	0.054621
27	oar3_OAR›	36693168	-0.02895
27	oar3_OAR›	36699539	-0.01739
27	oar3_OAR›	36699828	0.04067
27	oar3_OAR›	36701710	-0.01958
27	oar3_OAR›	36710615	-0.00886

27	oar3_OAR›	36714228	-0.02694
27	oar3_OAR›	36720541	0.047526
27	oar3_OAR›	36721486	0.105547
27	oar3_OAR›	36726522	-0.00716
27	oar3_OAR›	36730752	-0.02897
27	oar3_OAR›	36731497	-0.02818
27	oar3_OAR›	36732033	-0.02818
27	oar3_OAR›	36733773	0.015808
27	oar3_OAR›	36738612	0.001116
27	oar3_OAR›	36740239	0.047981
27	oar3_OAR›	36748660	0.010077
27	oar3_OAR›	36749948	-0.03022
27	oar3_OAR›	36752130	0.007354
27	oar3_OAR›	36754513	0.007354
27	oar3_OAR›	36761219	0.032582
27	oar3_OAR›	36761485	0.021331
27	oar3_OAR›	36763443	0.036107
27	oar3_OAR›	36769493	-0.00736
27	oar3_OAR›	36770311	0.003485
27	oar3_OAR›	36771288	-0.02662
27	oar3_OAR›	36775838	0.008806
27	oar3_OAR›	36778826	-0.02546
27	oar3_OAR›	36781374	-0.02662
27	oar3_OAR›	36786826	-0.00094
27	oar3_OAR›	36788118	-0.00593
27	oar3_OAR›	36789444	0.025413
27	oar3_OAR›	36802007	0.022278
27	oar3_OAR›	36802118	-0.00524
27	oar3_OAR›	36810353	0.124297
27	oar3_OAR›	36812170	-0.01739
27	oar3_OAR›	36817378	-0.01567
27	oar3_OAR›	36820130	-0.00928
27	oar3_OAR›	36822949	-0.02645
27	oar3_OAR›	36829571	-0.00512
27	oar3_OAR›	36829983	-0.00954
27	oar3_OAR›	36831926	-0.00954
27	oar3_OAR›	36834901	-0.01476
27	oar3_OAR›	36835102	-0.00011
27	oar3_OAR›	36843842	-0.02077
27	oar3_OAR›	36843898	0.01982
27	oar3_OAR›	36853623	-0.02498
27	oar3_OAR›	36866452	0.058228
27	oar3_OAR›	36866486	-0.02545
27	oar3_OAR›	36872419	0.161534
27	oar3_OAR›	36875381	0.037971
27	oar3_OAR›	36877391	0.025158
27	oar3_OAR›	36878573	0.145398
27	oar3_OAR›	36885111	0.082222
27	oar3_OAR›	36887376	0.003214
27	oar3_OAR›	36891021	0.082222
27	oar3_OAR›	36893074	0.167527
27	oar3_OAR›	36893122	0.032445
27	oar3_OAR›	36897259	-0.01736
27	oar3_OAR›	36897530	0.1111

27	oar3_OAR›	36905658	0.043729
27	oar3_OAR›	36906469	-0.01053
27	oar3_OAR›	36907603	-0.03785
27	oar3_OAR›	36912010	0.032582
27	oar3_OAR›	36917630	0.028042
27	oar3_OAR›	36920458	0.037003
27	oar3_OAR›	36929827	-0.01575
27	oar3_OAR›	36933669	-0.03085
27	oar3_OAR›	36934121	-0.02188
27	oar3_OAR›	36943917	0.038884
27	oar3_OAR›	36947899	-0.00385
27	oar3_OAR›	36956884	0.061149
27	oar3_OAR›	36960404	-0.00423
27	oar3_OAR›	36962075	-0.00932
27	oar3_OAR›	36964151	-0.01641
27	oar3_OAR›	36965992	NA
27	oar3_OAR›	36976429	0.030322
27	oar3_OAR›	36976442	0.002815
27	oar3_OAR›	36978235	0.033836
27	oar3_OAR›	36978775	0.016773
27	oar3_OAR›	36989455	-0.02981
27	oar3_OAR›	36991315	-0.02963
27	oar3_OAR›	36993266	-0.01367
27	oar3_OAR›	36995382	-0.011
27	oar3_OAR›	37002249	-0.0094
27	oar3_OAR›	37004248	-0.02227
27	oar3_OAR›	37004394	-0.00921
27	oar3_OAR›	37015929	-0.02527
27	oar3_OAR›	37017732	-0.00901
27	oar3_OAR›	37024075	-0.02961
27	oar3_OAR›	37024874	-0.00121
27	oar3_OAR›	37030075	-0.01665
27	oar3_OAR›	37030650	-0.01194
27	oar3_OAR›	37031602	0.005184
27	oar3_OAR›	37036332	-0.02899
27	oar3_OAR›	37044807	0.009496
27	oar3_OAR›	37048769	-0.01145
27	oar3_OAR›	37049575	-0.03164
27	oar3_OAR›	37049900	0.00811
27	oar3_OAR›	37051820	0.040031
27	oar3_OAR›	37057573	-0.03164
27	oar3_OAR›	37063611	-0.03167
27	oar3_OAR›	37063836	-0.03796
27	oar3_OAR›	37064236	-0.00802
27	oar3_OAR›	37068740	-0.0366
27	oar3_OAR›	37074835	0.016136
27	oar3_OAR›	37074962	0.016136
27	oar3_OAR›	37076586	0.041446
27	oar3_OAR›	37088483	0.036036
27	oar3_OAR›	37089692	-0.00304
27	oar3_OAR›	37090510	-0.00304
27	oar3_OAR›	37096570	-0.00216
27	oar3_OAR›	37102023	0.025222
27	oar3_OAR›	37102783	-0.03073

27	oar3_OAR›	37105416	-0.02049
27	oar3_OAR›	37107306	-0.03058
27	oar3_OAR›	37112530	-0.01423
27	oar3_OAR›	37114444	-0.0009
27	oar3_OAR›	37117733	-0.02831
27	oar3_OAR›	37122744	-0.02831
27	oar3_OAR›	37126369	-0.00319
27	oar3_OAR›	37126554	-0.00319
27	oar3_OAR›	37131900	-0.01445
27	oar3_OAR›	37132045	-0.02982
27	oar3_OAR›	37133919	-0.02831
27	oar3_OAR›	37134326	-0.02725
27	oar3_OAR›	37138199	0.031894
27	oar3_OAR›	37144568	0.031894
27	oar3_OAR›	37145236	0.031894
27	oar3_OAR›	37146369	-0.02982
27	oar3_OAR›	37149593	-0.032
27	oar3_OAR›	37150142	-0.01896
27	oar3_OAR›	37155876	-0.00723
27	oar3_OAR›	37172831	0.083619
27	oar3_OAR›	37177451	0.083619
27	oar3_OAR›	37193018	0.00395
27	oar3_OAR›	37196847	0.008681
27	oar3_OAR›	37198325	0.00395
27	oar3_OAR›	37200083	0.053499
27	oar3_OAR›	37200851	0.003562
27	oar3_OAR›	37209064	0.000889
27	oar3_OAR›	37213723	0.030887
27	oar3_OAR›	37221441	-0.01872
27	oar3_OAR›	37227480	0.025955
27	oar3_OAR›	37231452	-0.01332
27	oar3_OAR›	37238369	-0.0255
27	oar3_OAR›	37238434	0.217322
27	oar3_OAR›	37259217	0.018928
27	oar3_OAR›	37262811	0.054341
27	oar3_OAR›	37266285	0.217322
27	oar3_OAR›	37277329	0.230361
27	oar3_OAR›	37281273	0.095943
27	oar3_OAR›	37281557	0.093894
27	oar3_OAR›	37292520	0.10548
27	oar3_OAR›	37294368	8.73E-05
27	oar3_OAR›	37294754	-0.00817
27	oar3_OAR›	37302591	-0.00799
27	oar3_OAR›	37302647	-0.03938
27	oar3_OAR›	37303676	0.108231
27	oar3_OAR›	37322601	0.003805
27	oar3_OAR›	37323944	0.038472
27	oar3_OAR›	37336350	0.013016
27	oar3_OAR›	37337829	0.055946
27	oar3_OAR›	37342345	0.038185
27	oar3_OAR›	37344195	0.038185
27	oar3_OAR›	37355035	0.01928
27	oar3_OAR›	37357261	-0.02109
27	oar3_OAR›	37359869	0.032899

27	oar3_OAR›	37368014	0.071121
27	oar3_OAR›	37376843	0.060417
27	oar3_OAR›	37378855	0.078956
27	oar3_OAR›	37395588	0.010734
27	oar3_OAR›	37401151	-0.0314
27	oar3_OAR›	37414136	0.030891
27	oar3_OAR›	37414733	0.060014
27	oar3_OAR›	37419480	-0.01496
27	oar3_OAR›	37423279	0.039
27	oar3_OAR›	37429855	0.032582
27	oar3_OAR›	37432302	-0.00942
27	oar3_OAR›	37442859	0.059046
27	oar3_OAR›	37443444	0.259397
27	oar3_OAR›	37445474	0.059046
27	oar3_OAR›	37453597	0.134899
27	oar3_OAR›	37456227	0.016809
27	oar3_OAR›	37456423	-0.0218
27	oar3_OAR›	37461600	0.103899
27	oar3_OAR›	37462000	0.103899
27	oar3_OAR›	37467944	0.045114
27	oar3_OAR›	37474868	-0.02053
27	oar3_OAR›	37483684	0.005553
27	oar3_OAR›	37490783	0.009283
27	oar3_OAR›	37491758	0.012761
27	oar3_OAR›	37492864	0.004777
27	oar3_OAR›	37501292	0.010529
27	oar3_OAR›	37508606	-0.0255
27	oar3_OAR›	37515639	0.012512
27	oar3_OAR›	37517714	-0.0255
27	oar3_OAR›	37525942	-0.02171
27	oar3_OAR›	37535223	0.032003
27	oar3_OAR›	37539239	-0.02562
27	oar3_OAR›	37539678	-0.01607
27	oar3_OAR›	37551077	-0.02738
27	oar3_OAR›	37551261	-0.02801
27	oar3_OAR›	37551668	-0.02801
27	oar3_OAR›	37558037	-0.03123
27	oar3_OAR›	37560384	-0.00508
27	oar3_OAR›	37561317	-0.04266
27	oar3_OAR›	37574942	0.028942
27	oar3_OAR›	37574995	-0.03047
27	oar3_OAR›	37575248	-0.01566
27	oar3_OAR›	37579503	-0.02656
27	oar3_OAR›	37582949	-0.03047
27	oar3_OAR›	37584901	-0.03457
27	oar3_OAR›	37592033	-0.01346
27	oar3_OAR›	37593235	0.01603
27	oar3_OAR›	37597170	-0.02223
27	oar3_OAR›	37600772	-0.01776
27	oar3_OAR›	37606703	-0.00395
27	oar3_OAR›	37608682	-0.02941
27	oar3_OAR›	37609984	-0.02693
27	oar3_OAR›	37617627	-0.02377
27	oar3_OAR›	37621486	-0.02197

27	oar3_OAR›	37626738	0.048082
27	oar3_OAR›	37628277	0.048082
27	oar3_OAR›	37628538	0.048082
27	oar3_OAR›	37635270	-0.00932
27	oar3_OAR›	37639644	-0.0129
27	oar3_OAR›	37640506	0.027218
27	oar3_OAR›	37647720	0.010322
27	oar3_OAR›	37649838	0.018123
27	oar3_OAR›	37652487	-0.01317
27	oar3_OAR›	37657779	-0.01303
27	oar3_OAR›	37657895	0.064593
27	oar3_OAR›	37661079	-0.02059
27	oar3_OAR›	37663761	0.028509
27	oar3_OAR›	37672118	-0.01387
27	oar3_OAR›	37673942	-0.02875
27	oar3_OAR›	37680838	0.011116
27	oar3_OAR›	37684369	0.011116
27	oar3_OAR›	37698283	-0.02975
27	oar3_OAR›	37703275	-0.02342
27	oar3_OAR›	37713506	-0.02421
27	oar3_OAR›	37714561	-0.01758
27	oar3_OAR›	37721603	-0.01991
27	oar3_OAR›	37723636	-0.02549
27	oar3_OAR›	37723736	0.003111
27	oar3_OAR›	37728806	-0.02747
27	oar3_OAR›	37732671	-0.02982
27	oar3_OAR›	37733159	0.064341
27	oar3_OAR›	37751690	0.015096
27	oar3_OAR›	37752242	-0.00037
27	oar3_OAR›	37752497	0.01108
27	oar3_OAR›	37756958	0.007317
27	oar3_OAR›	37757349	0.027847
27	oar3_OAR›	37765079	-0.02224
27	oar3_OAR›	37769942	0.037791
27	oar3_OAR›	37773711	-0.02828
27	oar3_OAR›	37779944	-0.02036
27	oar3_OAR›	37782000	-0.01795
27	oar3_OAR›	37782040	-0.02036
27	oar3_OAR›	37784983	-0.01702
27	oar3_OAR›	37813372	-0.01733
27	oar3_OAR›	37815867	0.016173
27	oar3_OAR›	37816211	-0.01894
27	oar3_OAR›	37836394	0.126507
27	oar3_OAR›	37836568	0.168648
27	oar3_OAR›	37847801	-0.03147
27	oar3_OAR›	37856709	-0.02712
27	oar3_OAR›	37859287	-0.0041
27	oar3_OAR›	37863431	0.006716
27	oar3_OAR›	37864925	-0.0303
27	oar3_OAR›	37866211	0.081269
27	oar3_OAR›	37867246	-0.03592
27	oar3_OAR›	37871728	-0.0056
27	oar3_OAR›	37873943	0.000757
27	oar3_OAR›	37876215	0.007263

27	oar3_OAR›	37879190	-0.03418
27	oar3_OAR›	37893372	0.001522
27	oar3_OAR›	37895832	0.007181
27	oar3_OAR›	37898149	0.001807
27	oar3_OAR›	37900812	0.019808
27	oar3_OAR›	37904106	0.007104
27	oar3_OAR›	37909587	0.007104
27	oar3_OAR›	37917635	-0.02532
27	oar3_OAR›	37920389	-0.00744
27	oar3_OAR›	37920971	-0.01974
27	oar3_OAR›	37924952	-0.00744
27	oar3_OAR›	37927748	-0.00094
27	oar3_OAR›	37936443	-0.03509
27	oar3_OAR›	37941623	-0.02863
27	oar3_OAR›	37942651	-0.02398
27	oar3_OAR›	37948458	0.050445
27	oar3_OAR›	37950490	-0.01822
27	oar3_OAR›	37952875	0.02926
27	oar3_OAR›	37954359	0.050445
27	oar3_OAR›	37960449	-0.02555
27	oar3_OAR›	37981178	0.009633
27	oar3_OAR›	37991896	-0.02721
27	oar3_OAR›	37992057	0.008343
27	oar3_OAR›	37992124	0.008343
27	oar3_OAR›	37999573	0.007139
27	oar3_OAR›	38006024	0.046559
27	oar3_OAR›	38006910	0.036492
27	oar3_OAR›	38008974	0.187019
27	oar3_OAR›	38013229	-0.03222
27	oar3_OAR›	38014783	0.069252
27	oar3_OAR›	38017609	0.069252
27	oar3_OAR›	38017673	0.025664
27	oar3_OAR›	38027181	0.026399
27	oar3_OAR›	38035762	0.04878
27	oar3_OAR›	38038767	-0.01518
27	oar3_OAR›	38040866	-0.00914
27	oar3_OAR›	38041015	-0.011
27	oar3_OAR›	38046310	0.074675
27	oar3_OAR›	38051090	-0.00884
27	oar3_OAR›	38051352	0.03238
27	oar3_OAR›	38067479	0.000147
27	oar3_OAR›	38072889	0.017727
27	oar3_OAR›	38077685	-0.02398
27	oar3_OAR›	38077783	-0.01441
27	oar3_OAR›	38078721	-0.02345
27	oar3_OAR›	38079229	-0.02504
27	oar3_OAR›	38090164	0.001566
27	oar3_OAR›	38093182	-0.02961
27	oar3_OAR›	38093190	-0.02961
27	oar3_OAR›	38095598	-0.03075
27	oar3_OAR›	38106857	0.004747
27	oar3_OAR›	38107083	0.01132
27	oar3_OAR›	38116997	-0.02899
27	oar3_OAR›	38118754	0.007114

27	oar3_OAR›	38128662	-0.0168
27	oar3_OAR›	38129176	-0.0247
27	oar3_OAR›	38139392	0.01775
27	oar3_OAR›	38143490	0.05452
27	oar3_OAR›	38147784	0.020638
27	oar3_OAR›	38150045	0.106959
27	oar3_OAR›	38159876	-0.02465
27	oar3_OAR›	38173168	-0.01069
27	oar3_OAR›	38173392	0.091377
27	oar3_OAR›	38176744	-0.02974
27	oar3_OAR›	38182064	-0.03248
27	oar3_OAR›	38190039	-0.03237
27	oar3_OAR›	38203586	NA
27	oar3_OAR›	38203843	0.093344
27	oar3_OAR›	38205002	-0.00966
27	oar3_OAR›	38240463	-0.01546
27	oar3_OAR›	38253091	-0.00574
27	oar3_OAR›	38254225	########
27	oar3_OAR›	38261677	-0.03395
27	oar3_OAR›	38271848	-0.01271
27	oar3_OAR›	38274987	0.006976
27	oar3_OAR›	38279112	0.084844
27	oar3_OAR›	38279197	0.084844
27	oar3_OAR›	38283357	-0.01097
27	oar3_OAR›	38287311	-0.00917
27	oar3_OAR›	38292539	0.05199
27	oar3_OAR›	38293460	-0.01908
27	oar3_OAR›	38296734	0.02818
27	oar3_OAR›	38299502	0.036895
27	oar3_OAR›	38305445	0.036895
27	oar3_OAR›	38311850	0.05199
27	oar3_OAR›	38317254	0.05199
27	oar3_OAR›	38320651	0.020467
27	oar3_OAR›	38329586	-0.02464
27	oar3_OAR›	38329627	0.020467
27	oar3_OAR›	38341287	0.034274
27	oar3_OAR›	38346436	-0.01271
27	oar3_OAR›	38351768	-0.01815
27	oar3_OAR›	38355743	0.006014
27	oar3_OAR›	38358557	0.003989
27	oar3_OAR›	38369824	0.046159
27	oar3_OAR›	38377568	-0.01192
27	oar3_OAR›	38380609	0.009572
27	oar3_OAR›	38386536	-0.00169
27	oar3_OAR›	38393971	0.121522
27	oar3_OAR›	38396689	NA
27	oar3_OAR›	38400407	-0.02049
27	oar3_OAR›	38404833	0.042172
27	oar3_OAR›	38414728	-0.02714
27	oar3_OAR›	38414779	-0.00102
27	oar3_OAR›	38415162	0.030723
27	oar3_OAR›	38418845	-0.02292
27	oar3_OAR›	38433322	0.036182
27	oar3_OAR›	38442125	-0.01735

27	oar3_OAR›	38444721	0.063108
27	oar3_OAR›	38445329	-0.01851
27	oar3_OAR›	38451801	0.028726
27	oar3_OAR›	38458014	-0.00132
27	oar3_OAR›	38458409	0.00798
27	oar3_OAR›	38460505	0.01645
27	oar3_OAR›	38466515	-0.03151
27	oar3_OAR›	38471492	0.025714
27	oar3_OAR›	38476239	-0.02851
27	oar3_OAR›	38476713	0.001892
27	oar3_OAR›	38481454	0.035488
27	oar3_OAR›	38481829	-0.02271
27	oar3_OAR›	38488476	0.045931
27	oar3_OAR›	38488569	0.046594
27	oar3_OAR›	38497675	0.045143
27	oar3_OAR›	38497693	0.116133
27	oar3_OAR›	38498787	0.034079
27	oar3_OAR›	38502114	-0.00593
27	oar3_OAR›	38515836	0.288122
27	oar3_OAR›	38516002	0.018326
27	oar3_OAR›	38528301	0.13383
27	oar3_OAR›	38528468	0.233697
27	oar3_OAR›	38530179	-0.01737
27	oar3_OAR›	38535433	-0.01737
27	oar3_OAR›	38535505	0.150788
27	oar3_OAR›	38537940	0.06455
27	oar3_OAR›	38542964	0.20641
27	oar3_OAR›	38554528	-0.03509
27	oar3_OAR›	38561384	-0.02174
27	oar3_OAR›	38564807	-0.0353
27	oar3_OAR›	38567062	0.010527
27	oar3_OAR›	38570621	-0.00166
27	oar3_OAR›	38574086	0.047
27	oar3_OAR›	38577383	0.026942
27	oar3_OAR›	38577637	0.012006
27	oar3_OAR›	38587213	0.036455
27	oar3_OAR›	38593930	-0.0055
27	oar3_OAR›	38602688	-0.0404
27	oar3_OAR›	38602715	0.035698
27	oar3_OAR›	38602915	-0.00163
27	oar3_OAR›	38613808	0.034523
27	oar3_OAR›	38614035	-0.03763
27	oar3_OAR›	38629518	-0.00021
27	oar3_OAR›	38631269	0.057529
27	oar3_OAR›	38635471	0.013007
27	oar3_OAR›	38640519	-0.02575
27	oar3_OAR›	38641574	-0.0206
27	oar3_OAR›	38647052	0.100156
27	oar3_OAR›	38648578	0.035084
27	oar3_OAR›	38651147	-0.02674
27	oar3_OAR›	38654528	-0.02674
27	oar3_OAR›	38660081	-0.00099
27	oar3_OAR›	38661677	0.011193
27	oar3_OAR›	38669319	-0.01819

27	oar3_OAR⟩	38671485	-0.02184
27	oar3_OAR⟩	38671665	0.090385
27	oar3_OAR⟩	38679783	-0.01138
27	oar3_OAR⟩	38685063	-0.02764
27	oar3_OAR⟩	38685268	0.068069
27	oar3_OAR⟩	38690185	0.040066
27	oar3_OAR⟩	38692716	-0.01069
27	oar3_OAR⟩	38696179	0.026757
27	oar3_OAR⟩	38696639	0.026757
27	oar3_OAR⟩	38708810	-0.01562
27	oar3_OAR⟩	38709893	-0.01581
27	oar3_OAR⟩	38721842	-0.01069
27	oar3_OAR⟩	38722327	-0.00148
27	oar3_OAR⟩	38722393	-0.00726
27	oar3_OAR⟩	38735166	-0.00931
27	oar3_OAR⟩	38741102	-0.00887
27	oar3_OAR⟩	38744508	0.003107
27	oar3_OAR⟩	38745577	0.075962
27	oar3_OAR⟩	38750954	0.026894
27	oar3_OAR⟩	38751346	0.012132
27	oar3_OAR⟩	38757594	0.007893
27	oar3_OAR⟩	38759766	-0.00041
27	oar3_OAR⟩	38763306	0.036205
27	oar3_OAR⟩	38766426	-0.02846
27	oar3_OAR⟩	38767810	0.038987
27	oar3_OAR⟩	38768647	-0.0054
27	oar3_OAR⟩	38775182	-0.00081
27	oar3_OAR⟩	38779220	-0.00152
27	oar3_OAR⟩	38779707	-0.036
27	oar3_OAR⟩	38784510	0.004942
27	oar3_OAR⟩	38784875	-0.01774
27	oar3_OAR⟩	38786017	-0.03375
27	oar3_OAR⟩	38790164	-0.0275
27	oar3_OAR⟩	38796754	0.019235
27	oar3_OAR⟩	38800666	0.004942
27	oar3_OAR⟩	38806078	-0.02421
27	oar3_OAR⟩	38807491	0.015278
27	oar3_OAR⟩	38809131	-0.02963
27	oar3_OAR⟩	38812916	0.058709
27	oar3_OAR⟩	38815583	-0.02963
27	oar3_OAR⟩	38816813	-0.02421
27	oar3_OAR⟩	38819177	0.023574
27	oar3_OAR⟩	38824080	-0.02209
27	oar3_OAR⟩	38833158	0.080137
27	oar3_OAR⟩	38834162	0.087857
27	oar3_OAR⟩	38842940	0.094729
27	oar3_OAR⟩	38843556	0.094729
27	oar3_OAR⟩	38851498	0.029783
27	oar3_OAR⟩	38852617	0.053964
27	oar3_OAR⟩	38857997	0.016857
27	oar3_OAR⟩	38863293	-0.02519
27	oar3_OAR⟩	38865103	0.012337
27	oar3_OAR⟩	38865173	0.024716
27	oar3_OAR⟩	38877762	-0.00874

27	oar3_OAR›	38881255	-0.02235
27	oar3_OAR›	38884595	-0.01841
27	oar3_OAR›	38891078	-0.00811
27	oar3_OAR›	38892068	0.035972
27	oar3_OAR›	38899914	0.004904
27	oar3_OAR›	38915015	0.006839
27	oar3_OAR›	38930634	0.016364
27	oar3_OAR›	38934570	-0.03521
27	oar3_OAR›	38941567	-0.04065
27	oar3_OAR›	38948071	-0.04332
27	oar3_OAR›	38949437	0.028517
27	oar3_OAR›	38961799	-0.02797
27	oar3_OAR›	38963158	-0.04082
27	oar3_OAR›	38963675	-0.04065
27	oar3_OAR›	38971280	0.000321
27	oar3_OAR›	38973280	0.010393
27	oar3_OAR›	38975097	-0.02681
27	oar3_OAR›	38977897	0.016364
27	oar3_OAR›	38984281	-0.03219
27	oar3_OAR›	38987262	0.015492
27	oar3_OAR›	38987439	0.016364
27	oar3_OAR›	39001969	0.083953
27	oar3_OAR›	39007303	0.042308
27	oar3_OAR›	39013336	0.008019
27	oar3_OAR›	39013489	0.040698
27	oar3_OAR›	39017659	0.038949
27	oar3_OAR›	39024676	0.037747
27	oar3_OAR›	39025624	-0.0177
27	oar3_OAR›	39028474	-0.00249
27	oar3_OAR›	39030964	0.037747
27	oar3_OAR›	39037125	0.034159
27	oar3_OAR›	39038060	0.109969
27	oar3_OAR›	39043903	0.109914
27	oar3_OAR›	39050324	0.011067
27	oar3_OAR›	39053577	0.031225
27	oar3_OAR›	39054509	-0.01427
27	oar3_OAR›	39060961	0.028961
27	oar3_OAR›	39069373	0.006216
27	oar3_OAR›	39077056	0.044724
27	oar3_OAR›	39081173	0.112681
27	oar3_OAR›	39083487	0.029811
27	oar3_OAR›	39088903	0.07412
27	oar3_OAR›	39090854	-0.03521
27	oar3_OAR›	39095704	0.060934
27	oar3_OAR›	39109734	-0.01386
27	oar3_OAR›	39120981	0.065285
27	oar3_OAR›	39121487	-0.02461
27	oar3_OAR›	39121731	-0.02348
27	oar3_OAR›	39129825	-0.00735
27	oar3_OAR›	39136610	0.002764
27	oar3_OAR›	39139713	0.005477
27	oar3_OAR›	39147408	-0.03811
27	oar3_OAR›	39147753	0.00834
27	oar3_OAR›	39148011	9.44E-05

27	oar3_OAR>	39152538	0.008214
27	oar3_OAR>	39165137	0.008214
27	oar3_OAR>	39167387	0.013093
27	oar3_OAR>	39169531	0.013093
27	oar3_OAR>	39174071	0.063599
27	oar3_OAR>	39175330	-0.01316
27	oar3_OAR>	39187788	-0.00687
27	oar3_OAR>	39190271	0.040246
27	oar3_OAR>	39192117	-0.02413
27	oar3_OAR>	39199183	0.014156
27	oar3_OAR>	39200712	-0.02413
27	oar3_OAR>	39202258	-0.02413
27	oar3_OAR>	39209392	-0.03439
27	oar3_OAR>	39213678	0.028231
27	oar3_OAR>	39219019	-0.03842
27	oar3_OAR>	39225187	-0.01202
27	oar3_OAR>	39227230	-0.02413
27	oar3_OAR>	39229470	0.004295
27	oar3_OAR>	39236958	-0.01243
27	oar3_OAR>	39245759	-0.02035
27	oar3_OAR>	39246507	-0.02413
27	oar3_OAR>	39251150	-0.03116
27	oar3_OAR>	39257196	-0.02674
27	oar3_OAR>	39258006	-0.02768
27	oar3_OAR>	39259056	-0.01295
27	oar3_OAR>	39267310	-0.02217
27	oar3_OAR>	39269036	-0.01567
27	oar3_OAR>	39278955	-0.02545
27	oar3_OAR>	39279497	-0.02217
27	oar3_OAR>	39291636	0.145398
27	oar3_OAR>	39296700	-0.00356
27	oar3_OAR>	39302127	-0.00895
27	oar3_OAR>	39303912	-0.02545
27	oar3_OAR>	39305768	-0.0184
27	oar3_OAR>	39315204	-0.02959
27	oar3_OAR>	39316944	-0.02545
27	oar3_OAR>	39320820	0.018645
27	oar3_OAR>	39322108	0.113647
27	oar3_OAR>	39329840	0.01578
27	oar3_OAR>	39331344	0.027215
27	oar3_OAR>	39333923	-0.01413
27	oar3_OAR>	39337571	0.030893
27	oar3_OAR>	39339629	-0.02442
27	oar3_OAR>	39344057	-0.00887
27	oar3_OAR>	39348327	-0.02842
27	oar3_OAR>	39349294	0.035778
27	oar3_OAR>	39355263	0.035778
27	oar3_OAR>	39363245	-0.02618
27	oar3_OAR>	39364305	0.130828
27	oar3_OAR>	39372394	-0.00698
27	oar3_OAR>	39373036	0.071928
27	oar3_OAR>	39375500	-0.03616
27	oar3_OAR>	39380904	0.096034
27	oar3_OAR>	39391313	0.123308

27	oar3_OAR›	39401924	0.071488
27	oar3_OAR›	39412778	-0.02068
27	oar3_OAR›	39413258	-0.01737
27	oar3_OAR›	39415631	-0.01395
27	oar3_OAR›	39423538	-0.01605
27	oar3_OAR›	39424402	0.106455
27	oar3_OAR›	39432171	-0.01737
27	oar3_OAR›	39444526	0.073971
27	oar3_OAR›	39445581	-0.01224
27	oar3_OAR›	39446544	0.096115
27	oar3_OAR›	39447175	0.037931
27	oar3_OAR›	39455063	0.113439
27	oar3_OAR›	39465958	0.052651
27	oar3_OAR›	39475269	0.031709
27	oar3_OAR›	39479058	-0.01567
27	oar3_OAR›	39484556	0.02955
27	oar3_OAR›	39484666	-0.01737
27	oar3_OAR›	39489830	0.02955
27	oar3_OAR›	39493892	0.074647
27	oar3_OAR›	39496189	-0.00677
27	oar3_OAR›	39501156	0.06561
27	oar3_OAR›	39502612	0.020415
27	oar3_OAR›	39505157	0.071564
27	oar3_OAR›	39506996	0.020498
27	oar3_OAR›	39507928	0.085304
27	oar3_OAR›	39520233	0.146006
27	oar3_OAR›	39521209	0.082199
27	oar3_OAR›	39532455	-0.01459
27	oar3_OAR›	39533265	-0.01459
27	oar3_OAR›	39536313	0.025009
27	oar3_OAR›	39539908	0.034307
27	oar3_OAR›	39541865	0.057107
27	oar3_OAR›	39545964	0.009985
27	oar3_OAR›	39551600	-0.01058
27	oar3_OAR›	39554369	-0.01426
27	oar3_OAR›	39557021	0.076597
27	oar3_OAR›	39563106	-0.01426
27	oar3_OAR›	39568221	-0.01426
27	oar3_OAR›	39579388	-0.02891
27	oar3_OAR›	39579606	0.005042
27	oar3_OAR›	39583197	-0.01737
27	oar3_OAR›	39589925	-0.03488
27	oar3_OAR›	39590197	-0.03488
27	oar3_OAR›	39591774	-0.03022
27	oar3_OAR›	39603096	-0.03094
27	oar3_OAR›	39606215	-0.02165
27	oar3_OAR›	39613132	-0.02087
27	oar3_OAR›	39613742	-0.02147
27	oar3_OAR›	39626007	0.011365
27	oar3_OAR›	39628985	0.011365
27	oar3_OAR›	39629508	-0.00334
27	oar3_OAR›	39635774	-0.025
27	oar3_OAR›	39640166	-0.03287
27	oar3_OAR›	39652285	0.018804

27	oar3_OAR›	39652437	0.006183
27	oar3_OAR›	39653386	0.009727
27	oar3_OAR›	39659881	0.023093
27	oar3_OAR›	39663511	0.004606
27	oar3_OAR›	39665703	0.036456
27	oar3_OAR›	39667869	0.009727
27	oar3_OAR›	39672552	0.011365
27	oar3_OAR›	39674666	########
27	oar3_OAR›	39680024	########
27	oar3_OAR›	39682009	-0.00081
27	oar3_OAR›	39684890	0.074072
27	oar3_OAR›	39685102	0.074072
27	oar3_OAR›	39692854	0.008555
27	oar3_OAR›	39693080	0.093921
27	oar3_OAR›	39693512	0.009727
27	oar3_OAR›	39700495	0.008896
27	oar3_OAR›	39706450	-0.02154
27	oar3_OAR›	39707399	-0.01649
27	oar3_OAR›	39708164	0.008088
27	oar3_OAR›	39712683	0.005915
27	oar3_OAR›	39719209	0.009643
27	oar3_OAR›	39719659	0.050809
27	oar3_OAR›	39719839	-0.0023
27	oar3_OAR›	39721650	-0.01661
27	oar3_OAR›	39732150	-0.01661
27	oar3_OAR›	39734852	-0.01074
27	oar3_OAR›	39736257	0.02247
27	oar3_OAR›	39736427	-0.0023
27	oar3_OAR›	39744571	0.093921
27	oar3_OAR›	39753791	0.016891
27	oar3_OAR›	39754611	0.069893
27	oar3_OAR›	39759716	0.000464
27	oar3_OAR›	39762684	0.017167
27	oar3_OAR›	39762967	0.017167
27	oar3_OAR›	39770351	0.018804
27	oar3_OAR›	39773024	0.037131
27	oar3_OAR›	39775007	-0.00748
27	oar3_OAR›	39775298	0.075923
27	oar3_OAR›	39779471	0.037131
27	oar3_OAR›	39784469	0.056326
27	oar3_OAR›	39797024	0.077226
27	oar3_OAR›	39797513	0.095426
27	oar3_OAR›	39804885	0.020778
27	oar3_OAR›	39809800	0.021882
27	oar3_OAR›	39812106	0.05804
27	oar3_OAR›	39813448	0.05804
27	oar3_OAR›	39815250	-0.01367
27	oar3_OAR›	39822944	0.018624
27	oar3_OAR›	39825139	0.048686
27	oar3_OAR›	39834219	0.067755
27	oar3_OAR›	39837283	-0.01628
27	oar3_OAR›	39839612	0.03103
27	oar3_OAR›	39840382	NA
27	oar3_OAR›	39847871	0.071317

27	oar3_OAR⟩	39850523	0.032012
27	oar3_OAR⟩	39860733	0.032012
27	oar3_OAR⟩	39865286	0.022167
27	oar3_OAR⟩	39865383	0.032012
27	oar3_OAR⟩	39872645	0.017279
27	oar3_OAR⟩	39876802	0.032012
27	oar3_OAR⟩	39878121	0.033324
27	oar3_OAR⟩	39878643	0.001585
27	oar3_OAR⟩	39879002	0.017279
27	oar3_OAR⟩	39880614	-0.0093
27	oar3_OAR⟩	39887166	0.015023
27	oar3_OAR⟩	39887251	0.049642
27	oar3_OAR⟩	39893215	0.049642
27	oar3_OAR⟩	39898226	0.021037
27	oar3_OAR⟩	39904309	0.033324
27	oar3_OAR⟩	39907612	-0.00633
27	oar3_OAR⟩	39908104	0.040537
27	oar3_OAR⟩	39910593	0.040537
27	oar3_OAR⟩	39916225	0.06759
27	oar3_OAR⟩	39921431	0.040537
27	oar3_OAR⟩	39923433	0.040537
27	oar3_OAR⟩	39924125	0.015023
27	oar3_OAR⟩	39927631	0.017279
27	oar3_OAR⟩	39929880	0.011677
27	oar3_OAR⟩	39935211	0.009529
27	oar3_OAR⟩	39941672	0.016681
27	oar3_OAR⟩	39943880	0.039481
27	oar3_OAR⟩	39943908	0.011765
27	oar3_OAR⟩	39945264	0.011765
27	oar3_OAR⟩	39950719	0.036036
27	oar3_OAR⟩	39955817	-0.0085
27	oar3_OAR⟩	39956119	0.013183
27	oar3_OAR⟩	39957690	-0.0085
27	oar3_OAR⟩	39962612	0.008296
27	oar3_OAR⟩	39972621	0.00019
27	oar3_OAR⟩	39973015	0.094837
27	oar3_OAR⟩	39973396	0.050112
27	oar3_OAR⟩	39978672	0.050112
27	oar3_OAR⟩	39985138	0.037163
27	oar3_OAR⟩	39997311	-0.01757
27	oar3_OAR⟩	39997379	-0.01747
27	oar3_OAR⟩	40001764	-0.01757
27	oar3_OAR⟩	40001823	-0.01399
27	oar3_OAR⟩	40009791	0.07723
27	oar3_OAR⟩	40012942	0.035327
27	oar3_OAR⟩	40014793	0.007039
27	oar3_OAR⟩	40034188	0.00182
27	oar3_OAR⟩	40034216	0.025222
27	oar3_OAR⟩	40050359	-0.01794
27	oar3_OAR⟩	40050385	-0.0057
27	oar3_OAR⟩	40058470	-0.02336
27	oar3_OAR⟩	40073298	-0.00428
27	oar3_OAR⟩	40073524	-0.00687
27	oar3_OAR⟩	40079342	-0.00287

27	oar3_OAR›	40084837	-0.00687
27	oar3_OAR›	40095003	0.038755
27	oar3_OAR›	40096228	0.027371
27	oar3_OAR›	40099112	0.025934
27	oar3_OAR›	40118793	0.013045
27	oar3_OAR›	40121213	0.087691
27	oar3_OAR›	40123918	0.200733
27	oar3_OAR›	40142592	0.148388
27	oar3_OAR›	40143001	0.023763
27	oar3_OAR›	40143812	0.023763
27	oar3_OAR›	40147241	0.122188
27	oar3_OAR›	40148243	0.148388
27	oar3_OAR›	40160226	-0.02057
27	oar3_OAR›	40163019	-0.00322
27	oar3_OAR›	40164197	0.087691
27	oar3_OAR›	40172400	0.087691
27	oar3_OAR›	40173688	-0.00188
27	oar3_OAR›	40193815	0.00651
27	oar3_OAR›	40193978	-0.00287
27	oar3_OAR›	40201054	-0.00791
27	oar3_OAR›	40204868	0.007494
27	oar3_OAR›	40212358	0.040078
27	oar3_OAR›	40217173	-0.02304
27	oar3_OAR›	40217742	0.030887
27	oar3_OAR›	40218359	-0.01795
27	oar3_OAR›	40236057	-0.02304
27	oar3_OAR›	40236157	0.040078
27	oar3_OAR›	40247269	0.040078
27	oar3_OAR›	40248142	-0.03186
27	oar3_OAR›	40252501	0.119533
27	oar3_OAR›	40256689	0.037262
27	oar3_OAR›	40262100	0.040078
27	oar3_OAR›	40267874	0.040078
27	oar3_OAR›	40271547	0.040141
27	oar3_OAR›	40271640	0.130699
27	oar3_OAR›	40279753	0.066754
27	oar3_OAR›	40288771	-0.00243
27	oar3_OAR›	40302259	-0.01387
27	oar3_OAR›	40309127	-0.01646
27	oar3_OAR›	40317305	0.036197
27	oar3_OAR›	40320848	-0.01578
27	oar3_OAR›	40333014	0.036197
27	oar3_OAR›	40333939	-0.00508
27	oar3_OAR›	40335488	-0.00508
27	oar3_OAR›	40344578	0.000889
27	oar3_OAR›	40344744	-0.01186
27	oar3_OAR›	40345710	0.041692
27	oar3_OAR›	40361551	0.103661
27	oar3_OAR›	40364536	0.148411
27	oar3_OAR›	40365319	0.081547
27	oar3_OAR›	40375798	-0.00606
27	oar3_OAR›	40378647	0.002868
27	oar3_OAR›	40384955	0.031933
27	oar3_OAR›	40386173	0.023659

27	oar3_OAR›	40391261	-0.01364
27	oar3_OAR›	40396677	0.070004
27	oar3_OAR›	40413913	0.023023
27	oar3_OAR›	40414403	0.005188
27	oar3_OAR›	40414831	0.028715
27	oar3_OAR›	40422050	0.028715
27	oar3_OAR›	40431455	-0.00299
27	oar3_OAR›	40435465	0.028715
27	oar3_OAR›	40435893	0.005188
27	oar3_OAR›	40448259	0.028715
27	oar3_OAR›	40461341	0.084199
27	oar3_OAR›	40463434	0.025112
27	oar3_OAR›	40476542	0.040591
27	oar3_OAR›	40476685	0.049754
27	oar3_OAR›	40477592	0.084199
27	oar3_OAR›	40480303	-0.03268
27	oar3_OAR›	40485561	-0.02161
27	oar3_OAR›	40486501	0.056041
27	oar3_OAR›	40488588	0.061625
27	oar3_OAR›	40496033	0.039295
27	oar3_OAR›	40501945	-0.02188
27	oar3_OAR›	40504507	-0.02254
27	oar3_OAR›	40505772	-0.03123
27	oar3_OAR›	40509791	-0.00735
27	oar3_OAR›	40512711	-0.01594
27	oar3_OAR›	40515625	-0.02926
27	oar3_OAR›	40516068	-0.03191
27	oar3_OAR›	40523940	-0.03191
27	oar3_OAR›	40527400	-0.03191
27	oar3_OAR›	40528169	0.003989
27	oar3_OAR›	40535413	-0.00882
27	oar3_OAR›	40536871	-0.02996
27	oar3_OAR›	40543616	-0.02957
27	oar3_OAR›	40548272	-0.02957
27	oar3_OAR›	40548896	-0.03085
27	oar3_OAR›	40549186	-0.00999
27	oar3_OAR›	40554489	-0.00869
27	oar3_OAR›	40558515	-0.00322
27	oar3_OAR›	40575775	0.000605
27	oar3_OAR›	40578728	8.73E-05
27	oar3_OAR›	40585815	8.73E-05
27	oar3_OAR›	40592273	8.73E-05
27	oar3_OAR›	40598932	8.73E-05
27	oar3_OAR›	40600849	-0.03806
27	oar3_OAR›	40601287	0.000605
27	oar3_OAR›	40605561	8.73E-05
27	oar3_OAR›	40611046	0.009734
27	oar3_OAR›	40614037	-0.04118
27	oar3_OAR›	40615387	-0.04118
27	oar3_OAR›	40616045	-0.03129
27	oar3_OAR›	40619429	-0.03129
27	oar3_OAR›	40621941	0.009734
27	oar3_OAR›	40629352	-0.01567
27	oar3_OAR›	40632510	-0.00209

27	oar3_OAR›	40632544	-0.03857
27	oar3_OAR›	40637313	8.73E-05
27	oar3_OAR›	40646008	-0.04118
27	oar3_OAR›	40646253	-0.01567
27	oar3_OAR›	40646256	0.009734
27	oar3_OAR›	40654250	0.009734
27	oar3_OAR›	40654512	-0.03129
27	oar3_OAR›	40662854	-0.01567
27	oar3_OAR›	40677025	-0.03772
27	oar3_OAR›	40677786	-0.03857
27	oar3_OAR›	40680127	-0.03418
27	oar3_OAR›	40681240	-0.03418
27	oar3_OAR›	40682032	0.009734
27	oar3_OAR›	40689758	0.017279
27	oar3_OAR›	40691108	0.0188
27	oar3_OAR›	40694038	0.0188
27	oar3_OAR›	40697595	0.001103
27	oar3_OAR›	40702830	-0.01232
27	oar3_OAR›	40708118	0.019394
27	oar3_OAR›	40708577	-0.00428
27	oar3_OAR›	40713542	0.02374
27	oar3_OAR›	40720058	-0.01423
27	oar3_OAR›	40725723	0.00504
27	oar3_OAR›	40725887	-0.02063
27	oar3_OAR›	40734890	-0.01066
27	oar3_OAR›	40742393	-0.01066
27	oar3_OAR›	40745004	-0.02237
27	oar3_OAR›	40754635	-0.02149
27	oar3_OAR›	40758438	0.032882
27	oar3_OAR›	40758457	-0.0115
27	oar3_OAR›	40765243	0.082935
27	oar3_OAR›	40770720	0.019294
27	oar3_OAR›	40770820	0.001811
27	oar3_OAR›	40775661	0.000889
27	oar3_OAR›	40782841	-0.0107
27	oar3_OAR›	40784318	0.032565
27	oar3_OAR›	40784544	0.035942
27	oar3_OAR›	40787655	0.022514
27	oar3_OAR›	40798458	-0.0132
27	oar3_OAR›	40800130	0.03488
27	oar3_OAR›	40803610	-0.02785
27	oar3_OAR›	40806033	3.85E-05
27	oar3_OAR›	40810268	-0.02463
27	oar3_OAR›	40814507	-0.02794
27	oar3_OAR›	40821724	-0.01092
27	oar3_OAR›	40822260	-0.01976
27	oar3_OAR›	40830038	0.052764
27	oar3_OAR›	40831758	-0.01473
27	oar3_OAR›	40835131	0.007746
27	oar3_OAR›	40835353	0.077726
27	oar3_OAR›	40835726	0.012147
27	oar3_OAR›	40840918	0.042893
27	oar3_OAR›	40841826	0.083803
27	oar3_OAR›	40846875	0.012147

27	oar3_OAR›	40848206	0.012147
27	oar3_OAR›	40851754	0.057024
27	oar3_OAR›	40852616	-0.01644
27	oar3_OAR›	40863121	-0.02389
27	oar3_OAR›	40867724	-0.0055
27	oar3_OAR›	40867727	-0.0055
27	oar3_OAR›	40872082	-0.01423
27	oar3_OAR›	40877532	-0.01905
27	oar3_OAR›	40878387	-0.0321
27	oar3_OAR›	40879944	-0.02243
27	oar3_OAR›	40881965	-0.01572
27	oar3_OAR›	40885010	-0.01789
27	oar3_OAR›	40891669	0.022582
27	oar3_OAR›	40898306	0.008012
27	oar3_OAR›	40913796	0.022786
27	oar3_OAR›	40913873	-0.02198
27	oar3_OAR›	40914268	-0.01567
27	oar3_OAR›	40921698	-0.02294
27	oar3_OAR›	40923234	-0.00528
27	oar3_OAR›	40927984	-0.00459
27	oar3_OAR›	40929799	0.012043
27	oar3_OAR›	40930563	0.013976
27	oar3_OAR›	40934773	0.031013
27	oar3_OAR›	40935859	0.006912
27	oar3_OAR›	40938787	0.008214
27	oar3_OAR›	40944924	0.021533
27	oar3_OAR›	40952554	-0.01558
27	oar3_OAR›	40953581	8.73E-05
27	oar3_OAR›	40955969	0.022266
27	oar3_OAR›	40956650	-0.00544
27	oar3_OAR›	40964866	-0.02337
27	oar3_OAR›	40965442	-0.01375
27	oar3_OAR›	40965583	0.013185
27	oar3_OAR›	40967831	-0.0056
27	oar3_OAR›	40975436	-0.01652
27	oar3_OAR›	40975769	-0.03236
27	oar3_OAR›	40975790	-0.01842
27	oar3_OAR›	41001079	-0.02295
27	oar3_OAR›	41003452	0.008088
27	oar3_OAR›	41017659	-0.00826
27	oar3_OAR›	41029938	0.015298
27	oar3_OAR›	41038398	0.050108
27	oar3_OAR›	41048644	-0.02058
27	oar3_OAR›	41052190	0.046361
27	oar3_OAR›	41063067	-0.02575
27	oar3_OAR›	41067354	0.018804
27	oar3_OAR›	41074830	-0.01979
27	oar3_OAR›	41075725	-0.02867
27	oar3_OAR›	41078365	-0.00976
27	oar3_OAR›	41083812	-0.04082
27	oar3_OAR›	41097434	0.066918
27	oar3_OAR›	41106665	0.043203
27	oar3_OAR›	41106828	0.029875
27	oar3_OAR›	41108210	0.040618

27	oar3_OAR>	41109412	-0.00457
27	oar3_OAR>	41118357	0.028626
27	oar3_OAR>	41122377	0.004559
27	oar3_OAR>	41122961	-0.00967
27	oar3_OAR>	41126164	0.011926
27	oar3_OAR>	41129035	0.102998
27	oar3_OAR>	41130402	0.066691
27	oar3_OAR>	41135110	-0.02018
27	oar3_OAR>	41138762	-0.02509
27	oar3_OAR>	41142337	0.055718
27	oar3_OAR>	41144598	0.055718
27	oar3_OAR>	41144985	-0.03032
27	oar3_OAR>	41149618	0.074196
27	oar3_OAR>	41149966	-0.02646
27	oar3_OAR>	41155591	-0.02133
27	oar3_OAR>	41155984	0.007009
27	oar3_OAR>	41156687	-0.02187
27	oar3_OAR>	41157447	-0.015
27	oar3_OAR>	41165247	-0.02104
27	oar3_OAR>	41165743	0.027152
27	oar3_OAR>	41166643	-0.01062
27	oar3_OAR>	41168681	-0.02695
27	oar3_OAR>	41181533	-0.00932
27	oar3_OAR>	41200245	-0.00243
27	oar3_OAR>	41205196	0.023929
27	oar3_OAR>	41207085	-0.00606
27	oar3_OAR>	41215320	-0.00788
27	oar3_OAR>	41215413	-0.01168
27	oar3_OAR>	41221229	-0.01997
27	oar3_OAR>	41226613	0.020295
27	oar3_OAR>	41236842	-0.00767
27	oar3_OAR>	41240261	0.074839
27	oar3_OAR>	41250563	0.056621
27	oar3_OAR>	41256974	0.030586
27	oar3_OAR>	41262866	-0.01822
27	oar3_OAR>	41269482	-0.01822
27	oar3_OAR>	41277599	-0.01199
27	oar3_OAR>	41282784	-0.0085
27	oar3_OAR>	41287804	-0.00185
27	oar3_OAR>	41289993	0.012006
27	oar3_OAR>	41299260	0.018408
27	oar3_OAR>	41299767	0.030434
27	oar3_OAR>	41317465	0.060412
27	oar3_OAR>	41318393	0.063
27	oar3_OAR>	41322609	0.009734
27	oar3_OAR>	41328555	0.044065
27	oar3_OAR>	41329505	-0.01091
27	oar3_OAR>	41337851	-0.0003
27	oar3_OAR>	41343560	-0.01091
27	oar3_OAR>	41346824	-0.01154
27	oar3_OAR>	41353781	0.027864
27	oar3_OAR>	41386341	-0.02567
27	oar3_OAR>	41394614	-0.02567
27	oar3_OAR>	41403043	0.004408

27	oar3_OAR>	41408522	0.013241
27	oar3_OAR>	41443596	-0.01824
27	oar3_OAR>	41451105	-0.01995
27	oar3_OAR>	41451579	-0.01302
27	oar3_OAR>	41464476	-0.01302
27	oar3_OAR>	41466347	0.005477
27	oar3_OAR>	41474116	-0.00825
27	oar3_OAR>	41485984	-0.03738
27	oar3_OAR>	41486510	-0.02327
27	oar3_OAR>	41508569	-0.03509
27	oar3_OAR>	41509223	-0.02872
27	oar3_OAR>	41517044	0.006085
27	oar3_OAR>	41520065	0.005115
27	oar3_OAR>	41525645	0.047845
27	oar3_OAR>	41526560	-0.02459
27	oar3_OAR>	41526994	-0.0246
27	oar3_OAR>	41535583	-0.00344
27	oar3_OAR>	41535647	0.059013
27	oar3_OAR>	41538229	-0.00781
27	oar3_OAR>	41553166	0.021818
27	oar3_OAR>	41553703	-0.01872
27	oar3_OAR>	41557861	0.012761
27	oar3_OAR>	41557991	-0.01756
27	oar3_OAR>	41563713	0.007936
27	oar3_OAR>	41570172	-0.01396
27	oar3_OAR>	41572918	0.009577
27	oar3_OAR>	41573801	0.019116
27	oar3_OAR>	41601188	0.01034
27	oar3_OAR>	41604556	0.025014
27	oar3_OAR>	41606780	-0.00633
27	oar3_OAR>	41612357	0.005932
27	oar3_OAR>	41612628	0.005932
27	oar3_OAR>	41631978	0.015023
27	oar3_OAR>	41640160	-0.0078
27	oar3_OAR>	41642550	0.023963
27	oar3_OAR>	41660097	-0.03842
27	oar3_OAR>	41667944	-0.0325
27	oar3_OAR>	41672720	-0.02184
27	oar3_OAR>	41674104	0.039844
27	oar3_OAR>	41695476	-0.01154
27	oar3_OAR>	41699727	0.039922
27	oar3_OAR>	41700626	-0.01531
27	oar3_OAR>	41705780	0.040661
27	oar3_OAR>	41707027	0.099751
27	oar3_OAR>	41718870	0.056046
27	oar3_OAR>	41732575	-0.0304
27	oar3_OAR>	41734823	0.028864
27	oar3_OAR>	41738946	0.02101
27	oar3_OAR>	41742646	0.024157
27	oar3_OAR>	41756267	0.079265
27	oar3_OAR>	41768427	0.020604
27	oar3_OAR>	41771144	0.010733
27	oar3_OAR>	41776402	-0.01716
27	oar3_OAR>	41776848	-0.03073

27	oar3_OAR›	41785315	0.036205
27	oar3_OAR›	41785346	-0.03518
27	oar3_OAR›	41791121	0.099057
27	oar3_OAR›	41794886	-0.00693
27	oar3_OAR›	41795272	-0.00693
27	oar3_OAR›	41800672	0.099057
27	oar3_OAR›	41805592	0.003028
27	oar3_OAR›	41807264	-0.01302
27	oar3_OAR›	41807360	0.099057
27	oar3_OAR›	41813934	0.016617
27	oar3_OAR›	41823360	-0.03518
27	oar3_OAR›	41836560	0.061014
27	oar3_OAR›	41841199	-0.02593
27	oar3_OAR›	41856631	0.033558
27	oar3_OAR›	41860656	-0.03228
27	oar3_OAR›	41865228	0.033558
27	oar3_OAR›	41867218	0.010876
27	oar3_OAR›	41870893	0.026712
27	oar3_OAR›	41872764	0.00435
27	oar3_OAR›	41875866	0.022867
27	oar3_OAR›	41878909	-0.01415
27	oar3_OAR›	41885760	0.052226
27	oar3_OAR›	41885987	0.00481
27	oar3_OAR›	41896176	0.004183
27	oar3_OAR›	41901487	0.004183
27	oar3_OAR›	41902438	-0.00576
27	oar3_OAR›	41911861	-0.00033
27	oar3_OAR›	41918557	-0.00033
27	oar3_OAR›	41918674	-0.00033
27	oar3_OAR›	41930335	0.062903
27	oar3_OAR›	41941924	0.031927
27	oar3_OAR›	41952044	0.021523
27	oar3_OAR›	41952287	0.021523
27	oar3_OAR›	41953623	0.006708
27	oar3_OAR›	41959828	0.028669
27	oar3_OAR›	41966556	0.029959
27	oar3_OAR›	41966664	-0.00735
27	oar3_OAR›	41966876	0.007869
27	oar3_OAR›	41975690	0.011642
27	oar3_OAR›	41977231	-0.01451
27	oar3_OAR›	41977298	-0.01567
27	oar3_OAR›	41987357	-0.01646
27	oar3_OAR›	41987506	0.029077
27	oar3_OAR›	41997186	0.004461
27	oar3_OAR›	41999311	0.040851
27	oar3_OAR›	42006106	0.040851
27	oar3_OAR›	42013722	0.004461
27	oar3_OAR›	42014415	0.006027
27	oar3_OAR›	42017782	-0.00345
27	oar3_OAR›	42018188	0.041725
27	oar3_OAR›	42026656	0.00725
27	oar3_OAR›	42031773	-0.00641
27	oar3_OAR›	42032605	0.009641
27	oar3_OAR›	42036714	0.00725

27	oar3_OAR›	42036998	0.00725
27	oar3_OAR›	42044865	-0.00491
27	oar3_OAR›	42047336	0.005915
27	oar3_OAR›	42047820	0.005915
27	oar3_OAR›	42049869	-0.00163
27	oar3_OAR›	42058890	-0.02193
27	oar3_OAR›	42061870	-0.02534
27	oar3_OAR›	42063922	-0.02765
27	oar3_OAR›	42067606	-0.02534
27	oar3_OAR›	42072293	-0.02765
27	oar3_OAR›	42077051	-0.00243
27	oar3_OAR›	42081989	-0.01573
27	oar3_OAR›	42082513	-0.02534
27	oar3_OAR›	42092879	0.011943
27	oar3_OAR›	42093439	-0.01539
27	oar3_OAR›	42094650	0.019593
27	oar3_OAR›	42098989	-0.03098
27	oar3_OAR›	42104518	0.002668
27	oar3_OAR›	42110086	-0.01202
27	oar3_OAR›	42110755	0.005546
27	oar3_OAR›	42111991	-0.01094
27	oar3_OAR›	42114927	-0.00012
27	oar3_OAR›	42117889	0.044644
27	oar3_OAR›	42118607	-0.03869
27	oar3_OAR›	42119255	0.110867
27	oar3_OAR›	42127525	-0.02113
27	oar3_OAR›	42133857	-0.02569
27	oar3_OAR›	42136928	0.015879
27	oar3_OAR›	42139172	-0.01573
27	oar3_OAR›	42140376	-0.00508
27	oar3_OAR›	42149979	-0.01856
27	oar3_OAR›	42151577	-0.01463
27	oar3_OAR›	42152408	-0.01858
27	oar3_OAR›	42169678	0.064097
27	oar3_OAR›	42177269	-0.01153
27	oar3_OAR›	42180284	-0.00498
27	oar3_OAR›	42195656	-0.01153
27	oar3_OAR›	42207066	-0.01332
27	oar3_OAR›	42212632	0.006638
27	oar3_OAR›	42216025	-0.00039
27	oar3_OAR›	42220773	0.008723
27	oar3_OAR›	42223263	-0.02979
27	oar3_OAR›	42225571	0.03329
27	oar3_OAR›	42231255	-0.02636
27	oar3_OAR›	42252975	0.044724
27	oar3_OAR›	42254762	0.009985
27	oar3_OAR›	42258138	0.011095
27	oar3_OAR›	42271184	0.019617
27	oar3_OAR›	42276365	0.060566
27	oar3_OAR›	42276658	0.019617
27	oar3_OAR›	42278528	0.011095
27	oar3_OAR›	42290529	0.003944
27	oar3_OAR›	42290543	0.0317
27	oar3_OAR›	42291891	0.041776

27	oar3_OAR〉	42309559	-0.02926
27	oar3_OAR〉	42310003	0.020044
27	oar3_OAR〉	42312321	0.047057
27	oar3_OAR〉	42331190	-0.01939
27	oar3_OAR〉	42331471	0.052166
27	oar3_OAR〉	42334293	0.077054
27	oar3_OAR〉	42343552	0.018804
27	oar3_OAR〉	42350645	0.018804
27	oar3_OAR〉	42354144	-0.00364
27	oar3_OAR〉	42354681	-0.00364
27	oar3_OAR〉	42361707	-0.00364
27	oar3_OAR〉	42364820	-0.0109
27	oar3_OAR〉	42366139	-0.02497
27	oar3_OAR〉	42372287	-0.03186
27	oar3_OAR〉	42386496	0.042337
27	oar3_OAR〉	42387630	-0.03733
27	oar3_OAR〉	42401833	0.018804
27	oar3_OAR〉	42405418	0.059606
27	oar3_OAR〉	42405527	-0.02703
27	oar3_OAR〉	42408335	-0.01346
27	oar3_OAR〉	42412749	0.026318
27	oar3_OAR〉	42426731	0.067171
27	oar3_OAR〉	42426834	0.018804
27	oar3_OAR〉	42433183	0.029918
27	oar3_OAR〉	42435279	-0.01622
27	oar3_OAR〉	42457998	-0.02525
27	oar3_OAR〉	42458327	-0.02525
27	oar3_OAR〉	42468319	0.099089
27	oar3_OAR〉	42471024	0.055489
27	oar3_OAR〉	42472575	-0.01289
27	oar3_OAR〉	42473907	0.008437
27	oar3_OAR〉	42484731	0.010936
27	oar3_OAR〉	42487951	-0.02188
27	oar3_OAR〉	42488422	-0.02188
27	oar3_OAR〉	42489722	-0.03198
27	oar3_OAR〉	42499998	-0.00578
27	oar3_OAR〉	42503253	-0.00968
27	oar3_OAR〉	42504274	-0.00968
27	oar3_OAR〉	42509511	0.022582
27	oar3_OAR〉	42511094	-0.02502
27	oar3_OAR〉	42515964	0.006556
27	oar3_OAR〉	42519233	0.027002
27	oar3_OAR〉	42535311	0.03836
27	oar3_OAR〉	42535515	-0.02656
27	oar3_OAR〉	42539941	0.049985
27	oar3_OAR〉	42558271	-0.01126
27	oar3_OAR〉	42558385	-0.00384
27	oar3_OAR〉	42564503	-0.01628
27	oar3_OAR〉	42568555	0.00563
27	oar3_OAR〉	42571309	0.005869
27	oar3_OAR〉	42577371	0.009795
27	oar3_OAR〉	42581912	0.098088
27	oar3_OAR〉	42589517	0.011784
27	oar3_OAR〉	42593964	-0.01583

27	oar3_OAR›	42597363	-0.0054
27	oar3_OAR›	42597489	-0.02747
27	oar3_OAR›	42601735	0.016617
27	oar3_OAR›	42606715	0.011808
27	oar3_OAR›	42615968	-0.02065
27	oar3_OAR›	42620130	-0.0219
27	oar3_OAR›	42622578	0.002019
27	oar3_OAR›	42624088	-0.03421
27	oar3_OAR›	42631633	0.030173
27	oar3_OAR›	42632445	-0.01139
27	oar3_OAR›	42643157	0.006157
27	oar3_OAR›	42643245	0.017462
27	oar3_OAR›	42643758	0.023492
27	oar3_OAR›	42643898	0.017462
27	oar3_OAR›	42649657	-0.01605
27	oar3_OAR›	42655198	0.000374
27	oar3_OAR›	42657413	0.041488
27	oar3_OAR›	42658805	0.006121
27	oar3_OAR›	42664088	0.04569
27	oar3_OAR›	42672390	0.086728
27	oar3_OAR›	42676785	0.036197
27	oar3_OAR›	42685216	0.041197
27	oar3_OAR›	42690759	-0.00379
27	oar3_OAR›	42690847	-0.01793
27	oar3_OAR›	42695653	0.210302
27	oar3_OAR›	42697263	0.044345
27	oar3_OAR›	42702529	-0.00379
27	oar3_OAR›	42710216	0.036974
27	oar3_OAR›	42712972	-0.02996
27	oar3_OAR›	42713043	-0.01558
27	oar3_OAR›	42713495	-0.01558
27	oar3_OAR›	42715383	-0.02565
27	oar3_OAR›	42740250	-0.02268
27	oar3_OAR›	42751645	-0.02703
27	oar3_OAR›	42751954	0.009674
27	oar3_OAR›	42751970	-0.00815
27	oar3_OAR›	42759221	-0.02295
27	oar3_OAR›	42759731	0.079826
27	oar3_OAR›	42761694	0.064315
27	oar3_OAR›	42778029	0.031551
27	oar3_OAR›	42781360	0.080702
27	oar3_OAR›	42794341	-0.02381
27	oar3_OAR›	42796695	0.04889
27	oar3_OAR›	42807239	-0.02049
27	oar3_OAR›	42813465	0.059394
27	oar3_OAR›	42817616	0.042821
27	oar3_OAR›	42817727	-0.02381
27	oar3_OAR›	42827163	-0.02381
27	oar3_OAR›	42830351	-0.02381
27	oar3_OAR›	42834020	-0.02049
27	oar3_OAR›	42858171	-0.01739
27	oar3_OAR›	42859038	-0.02049
27	oar3_OAR›	42874538	0.023394
27	oar3_OAR›	42876314	-0.01145

27	oar3_OAR›	42877772	-0.01963
27	oar3_OAR›	42879266	0.042542
27	oar3_OAR›	42887407	-0.01415
27	oar3_OAR›	42888103	-0.02735
27	oar3_OAR›	42896760	-0.02049
27	oar3_OAR›	42902736	0.026911
27	oar3_OAR›	42914972	-0.02168
27	oar3_OAR›	42921322	8.73E-05
27	oar3_OAR›	42927046	-0.02063
27	oar3_OAR›	42928481	0.005071
27	oar3_OAR›	42931781	-0.01667
27	oar3_OAR›	42941871	-0.02793
27	oar3_OAR›	42947037	-0.02926
27	oar3_OAR›	42977201	-0.02044
27	oar3_OAR›	42996932	-0.02981
27	oar3_OAR›	42996997	-0.02981
27	oar3_OAR›	42997558	-0.02572
27	oar3_OAR›	43003036	-0.01184
27	oar3_OAR›	43007114	0.009985
27	oar3_OAR›	43008009	-0.03064
27	oar3_OAR›	43008688	-0.02572
27	oar3_OAR›	43020389	-0.0036
27	oar3_OAR›	43020802	0.03822
27	oar3_OAR›	43027244	0.009639
27	oar3_OAR›	43035103	-0.02314
27	oar3_OAR›	43040500	-0.02314
27	oar3_OAR›	43042342	-0.03768
27	oar3_OAR›	43042521	0.00481
27	oar3_OAR›	43055181	0.051032
27	oar3_OAR›	43055370	0.037433
27	oar3_OAR›	43062930	-0.0064
27	oar3_OAR›	43070726	-0.02825
27	oar3_OAR›	43071241	-0.0064
27	oar3_OAR›	43071792	0.037433
27	oar3_OAR›	43086652	0.051032
27	oar3_OAR›	43091854	-0.01878
27	oar3_OAR›	43104138	0.041462
27	oar3_OAR›	43135787	0.064242
27	oar3_OAR›	43150756	0.017727
27	oar3_OAR›	43174099	0.017727
27	oar3_OAR›	43178331	0.017727
27	oar3_OAR›	43179495	0.017727
27	oar3_OAR›	43181385	-0.01234
27	oar3_OAR›	43190421	0.017727
27	oar3_OAR›	43198861	0.017727
27	oar3_OAR›	43210372	0.017727
27	oar3_OAR›	43216127	0.017727
27	oar3_OAR›	43222842	-0.03269
27	oar3_OAR›	43223585	0.017727
27	oar3_OAR›	43230162	0.017727
27	oar3_OAR›	43236119	0.017727
27	oar3_OAR›	43427283	-0.02694
27	oar3_OAR›	43555332	-0.00283
27	oar3_OAR›	43570926	-0.00183

27	oar3_OAR›	43582258	-0.00283
27	oar3_OAR›	43585023	0.001274
27	oar3_OAR›	43675280	0.018804
27	oar3_OAR›	43699482	-0.00836
27	oar3_OAR›	43707277	0.018804
27	oar3_OAR›	43792227	0.021059
27	oar3_OAR›	43793423	0.018804
27	oar3_OAR›	43823163	-0.01536
27	oar3_OAR›	43823624	0.021059
27	oar3_OAR›	43829446	-0.01536
27	oar3_OAR›	43852785	0.028089
27	oar3_OAR›	43892756	-0.01303
27	oar3_OAR›	43893196	0.028089
27	oar3_OAR›	43946604	-0.01583
27	oar3_OAR›	43950088	0.028089
27	oar3_OAR›	43958716	-0.01583
27	oar3_OAR›	43966567	0.028089
27	oar3_OAR›	43974632	0.013369
27	oar3_OAR›	43988238	0.003891
27	oar3_OAR›	43989233	0.003891
27	oar3_OAR›	44004584	0.013369
27	oar3_OAR›	44004817	0.028089
27	oar3_OAR›	44015631	0.028089
27	oar3_OAR›	44015833	0.013119
27	oar3_OAR›	44020752	0.078653
27	oar3_OAR›	44030994	0.013119
27	oar3_OAR›	44035003	0.028089
27	oar3_OAR›	44053227	0.078653
27	oar3_OAR›	44057308	0.028089
27	oar3_OAR›	44065372	0.028089
27	oar3_OAR›	44066343	0.028089
27	oar3_OAR›	44068381	0.093864
27	oar3_OAR›	44081544	0.082846
27	oar3_OAR›	44081974	0.028089
27	oar3_OAR›	44082868	0.022148
27	oar3_OAR›	44106402	0.028089
27	oar3_OAR›	44108990	0.082846
27	oar3_OAR›	44128986	-0.00165
27	oar3_OAR›	44135858	0.028089
27	oar3_OAR›	44137390	-0.03552
27	oar3_OAR›	44169169	0.023642
27	oar3_OAR›	44184551	0.028089
27	oar3_OAR›	44187122	0.078896
27	oar3_OAR›	44228860	-0.03617
27	oar3_OAR›	44238938	0.093291
27	oar3_OAR›	44245235	NA
27	oar3_OAR›	44247662	0.082846
27	oar3_OAR›	44283813	0.080432
27	oar3_OAR›	44324748	0.023642
27	oar3_OAR›	44389129	0.047145
27	oar3_OAR›	44391919	0.100982
27	oar3_OAR›	44400111	-0.01069
27	oar3_OAR›	44403985	0.100982
27	oar3_OAR›	44413771	0.068568

27	oar3_OAR〉	44422636	0.06417
27	oar3_OAR〉	44446083	0.028332
27	oar3_OAR〉	44446204	0.025783
27	oar3_OAR〉	44480631	0.068568
27	oar3_OAR〉	44485369	0.06417
27	oar3_OAR〉	44488619	0.06417
27	oar3_OAR〉	44497249	0.06417
27	oar3_OAR〉	44508907	0.06417
27	oar3_OAR〉	44509776	0.025783
27	oar3_OAR〉	44605834	0.141475
27	oar3_OAR〉	44606402	0.027002
27	oar3_OAR〉	44617660	0.027002
27	oar3_OAR〉	44619146	0.141475
27	oar3_OAR〉	44621327	-0.00606
27	oar3_OAR〉	44622900	0.141475
27	oar3_OAR〉	44651602	0.141475
27	oar3_OAR〉	44659077	0.141475
27	oar3_OAR〉	44661341	0.028803
27	oar3_OAR〉	44680640	0.024014
27	oar3_OAR〉	44683137	0.141475
27	oar3_OAR〉	44692162	0.141475
27	oar3_OAR〉	44709755	0.141475
27	oar3_OAR〉	44709925	0.141475
27	oar3_OAR〉	44729305	-0.00606
27	oar3_OAR〉	44751036	0.141475
27	oar3_OAR〉	44756601	0.141475
27	oar3_OAR〉	44759602	0.027002
27	oar3_OAR〉	44760716	0.024014
27	oar3_OAR〉	44784938	0.134284
27	oar3_OAR〉	44785188	0.141475
27	oar3_OAR〉	44838246	0.001737
27	oar3_OAR〉	44863310	-0.01822
27	oar3_OAR〉	44868065	0.001737
27	oar3_OAR〉	44871810	-0.01822
27	oar3_OAR〉	44914986	0.063714
27	oar3_OAR〉	44925687	0.091036
27	oar3_OAR〉	44971868	-0.01567
27	oar3_OAR〉	44989551	-0.00606
27	oar3_OAR〉	45035242	0.063714
27	oar3_OAR〉	45036560	-0.00606
27	oar3_OAR〉	45085237	0.001737
27	oar3_OAR〉	45089002	-0.00606
27	oar3_OAR〉	45137682	-0.00606
27	oar3_OAR〉	45141794	-0.00606
27	oar3_OAR〉	45157716	-0.00606
27	oar3_OAR〉	45167003	0.104328
27	oar3_OAR〉	45226993	-0.00606
27	oar3_OAR〉	45251843	-0.00996
27	oar3_OAR〉	45253719	0.001737
27	oar3_OAR〉	45257919	0.063714
27	oar3_OAR〉	45280477	-0.01567
27	oar3_OAR〉	45297905	0.104328
27	oar3_OAR〉	45300326	0.080088
27	oar3_OAR〉	45315889	0.104328

27	oar3_OAR⟩	45325557	0.104328
27	oar3_OAR⟩	45344722	0.104328
27	oar3_OAR⟩	45358178	-0.00606
27	oar3_OAR⟩	45359930	0.104328
27	oar3_OAR⟩	45367203	0.104328
27	oar3_OAR⟩	45438393	-0.00996
27	oar3_OAR⟩	45445329	0.104328
27	oar3_OAR⟩	45467094	0.104328
27	oar3_OAR⟩	45467557	0.104328
27	oar3_OAR⟩	45468061	-0.00966
27	oar3_OAR⟩	45492718	0.104328
27	oar3_OAR⟩	45499080	0.080525
27	oar3_OAR⟩	45510547	-0.00996
27	oar3_OAR⟩	45512185	0.096775
27	oar3_OAR⟩	45513185	0.096775
27	oar3_OAR⟩	45519247	-0.00606
27	oar3_OAR⟩	45524762	0.041496
27	oar3_OAR⟩	45546055	-0.00996
27	oar3_OAR⟩	45558075	0.096775
27	oar3_OAR⟩	45562519	-0.00996
27	oar3_OAR⟩	45595724	0.072979
27	oar3_OAR⟩	45596167	-0.00996
27	oar3_OAR⟩	45597569	-0.00166
27	oar3_OAR⟩	45607192	0.045953
27	oar3_OAR⟩	45619385	-0.00606
27	oar3_OAR⟩	45619934	0.009639
27	oar3_OAR⟩	45620411	0.012045
27	oar3_OAR⟩	45634568	0.009639
27	oar3_OAR⟩	45643355	0.009639
27	oar3_OAR⟩	45645272	-0.00278
27	oar3_OAR⟩	45649526	0.007893
27	oar3_OAR⟩	45651793	-0.00091
27	oar3_OAR⟩	45670965	0.009639
27	oar3_OAR⟩	45679803	0.009639
27	oar3_OAR⟩	45692771	-0.011
27	oar3_OAR⟩	45696144	-0.00091
27	oar3_OAR⟩	45738314	-0.00091
27	oar3_OAR⟩	45740557	0.022722
27	oar3_OAR⟩	45749462	-0.00091
27	oar3_OAR⟩	45751600	-0.00606
27	oar3_OAR⟩	45764317	0.009639
27	oar3_OAR⟩	45770138	0.009639
27	oar3_OAR⟩	45775171	-0.03164
27	oar3_OAR⟩	45775255	-0.00606
27	oar3_OAR⟩	45780618	-0.03164
27	oar3_OAR⟩	45786246	0.022722
27	oar3_OAR⟩	45799409	-0.00606
27	oar3_OAR⟩	45801319	-0.00091
27	oar3_OAR⟩	45813186	0.005771
27	oar3_OAR⟩	45813794	-0.011
27	oar3_OAR⟩	45821864	-0.00091
27	oar3_OAR⟩	45833145	-0.00091
27	oar3_OAR⟩	45842773	-0.01329
27	oar3_OAR⟩	45843265	-0.02845

27	oar3_OAR〉	45843292	-0.01329
27	oar3_OAR〉	45846008	-0.01329
27	oar3_OAR〉	45863762	-0.01329
27	oar3_OAR〉	45863889	-0.02845
27	oar3_OAR〉	45909953	0.009639
27	oar3_OAR〉	45931279	-0.00606
27	oar3_OAR〉	45931356	-0.02416
27	oar3_OAR〉	45945662	-0.01856
27	oar3_OAR〉	45966514	0.009639
27	oar3_OAR〉	45967632	-0.01982
27	oar3_OAR〉	45968150	-0.02179
27	oar3_OAR〉	45979687	-0.02179
27	oar3_OAR〉	45979857	0.009639
27	oar3_OAR〉	45995546	-0.03671
27	oar3_OAR〉	45996238	-0.00606
27	oar3_OAR〉	46006164	-0.02179
27	oar3_OAR〉	46008638	-0.02179
27	oar3_OAR〉	46010000	-0.0381
27	oar3_OAR〉	46012145	-0.03671
27	oar3_OAR〉	46017798	0.009639
27	oar3_OAR〉	46017799	-0.02179
27	oar3_OAR〉	46022927	-0.01982
27	oar3_OAR〉	46027950	-0.00606
27	oar3_OAR〉	46029337	-0.02242
27	oar3_OAR〉	46031664	-0.02473
27	oar3_OAR〉	46058377	-0.0381
27	oar3_OAR〉	46060168	-0.01081
27	oar3_OAR〉	46075724	-0.00606
27	oar3_OAR〉	46081726	-0.02242
27	oar3_OAR〉	46091546	-0.02242
27	oar3_OAR〉	46095542	-0.0381
27	oar3_OAR〉	46135435	-0.02278
27	oar3_OAR〉	46135531	-0.02242
27	oar3_OAR〉	46166416	-0.02278
27	oar3_OAR〉	46169736	-0.03181
27	oar3_OAR〉	46193222	-0.03671
27	oar3_OAR〉	46193861	-0.02044
27	oar3_OAR〉	46193900	-0.02278
27	oar3_OAR〉	46199926	-0.02242
27	oar3_OAR〉	46204052	-0.02044
27	oar3_OAR〉	46205923	-0.03652
27	oar3_OAR〉	46214471	-0.00606
27	oar3_OAR〉	46219862	-0.02242
27	oar3_OAR〉	46225156	-0.02044
27	oar3_OAR〉	46256223	-0.00606
27	oar3_OAR〉	46281598	-0.00606
27	oar3_OAR〉	46289493	-0.02242
27	oar3_OAR〉	46290952	-0.00606
27	oar3_OAR〉	46291072	-0.00606
27	oar3_OAR〉	46316184	0.008759
27	oar3_OAR〉	46319592	-0.00606
27	oar3_OAR〉	46320289	-0.01735
27	oar3_OAR〉	46321981	-0.00606
27	oar3_OAR〉	46325374	0.01084

27	oar3_OAR›	46330060	0.101047
27	oar3_OAR›	46338760	-0.01735
27	oar3_OAR›	46368667	-0.00606
27	oar3_OAR›	46371571	-0.00606
27	oar3_OAR›	46377438	0.012328
27	oar3_OAR›	46380912	-0.02127
27	oar3_OAR›	46391110	-0.02127
27	oar3_OAR›	46421747	-0.01735
27	oar3_OAR›	46423573	0.012328
27	oar3_OAR›	46425685	-0.00606
27	oar3_OAR›	46433677	0.101047
27	oar3_OAR›	46433824	0.078285
27	oar3_OAR›	46448352	0.000605
27	oar3_OAR›	46450882	0.101047
27	oar3_OAR›	46461665	-0.00606
27	oar3_OAR›	46462562	-0.01735
27	oar3_OAR›	46525419	-0.00606
27	oar3_OAR›	46568017	0.115787
27	oar3_OAR›	46569504	0.078285
27	oar3_OAR›	46570795	0.012328
27	oar3_OAR›	46598404	-0.00606
27	oar3_OAR›	46672930	0.066291
27	oar3_OAR›	46709602	-0.00606
27	oar3_OAR›	46738740	0.08005
27	oar3_OAR›	46740259	NA
27	oar3_OAR›	46796304	0.08005
27	oar3_OAR›	46801913	0.083378
27	oar3_OAR›	46833383	0.070899
27	oar3_OAR›	46860940	0.011643
27	oar3_OAR›	46924286	0.08005
27	oar3_OAR›	46930062	0.109761
27	oar3_OAR›	46931800	-0.00606
27	oar3_OAR›	46964362	0.109761
27	oar3_OAR›	46997514	-0.00606
27	oar3_OAR›	47002355	0.045144
27	oar3_OAR›	47009622	0.000363
27	oar3_OAR›	47009684	-0.00606
27	oar3_OAR›	47010288	0.076769
27	oar3_OAR›	47052946	0.125423
27	oar3_OAR›	47055853	0.076769
27	oar3_OAR›	47083075	-0.00606
27	oar3_OAR›	47147015	0.109761
27	oar3_OAR›	47154856	0.105547
27	oar3_OAR›	47163134	0.105547
27	oar3_OAR›	47164718	0.015534
27	oar3_OAR›	47169077	0.015534
27	oar3_OAR›	47181304	0.019032
27	oar3_OAR›	47189460	0.019032
27	oar3_OAR›	47195772	0.109761
27	oar3_OAR›	47196138	0.015534
27	oar3_OAR›	47208936	0.053223
27	oar3_OAR›	47220058	0.160419
27	oar3_OAR›	47275354	0.182927
27	oar3_OAR›	47278913	0.000363

27	oar3_OAR›	47287027	0.074454
27	oar3_OAR›	47292228	0.035613
27	oar3_OAR›	47312794	0.182927
27	oar3_OAR›	47312969	0.109761
27	oar3_OAR›	47327437	0.000363
27	oar3_OAR›	47329537	0.182927
27	oar3_OAR›	47340430	0.182927
27	oar3_OAR›	47342425	0.074454
27	oar3_OAR›	47346338	-0.00606
27	oar3_OAR›	47359440	0.000363
27	oar3_OAR›	47390752	0.109761
27	oar3_OAR›	47399369	0.109761
27	oar3_OAR›	47421965	-0.02672
27	oar3_OAR›	47435012	0.015111
27	oar3_OAR›	47437353	0.109761
27	oar3_OAR›	47445160	0.015111
27	oar3_OAR›	47447733	0.015111
27	oar3_OAR›	47450153	0.109761
27	oar3_OAR›	47456664	0.109761
27	oar3_OAR›	47458831	0.028084
27	oar3_OAR›	47463246	0.019032
27	oar3_OAR›	47535731	0.000363
27	oar3_OAR›	47539904	0.125423
27	oar3_OAR›	47541768	0.109761
27	oar3_OAR›	47549956	0.019032
27	oar3_OAR›	47556316	0.125423
27	oar3_OAR›	47592162	0.125423
27	oar3_OAR›	47601918	0.125423
27	oar3_OAR›	47616546	0.125423
27	oar3_OAR›	47617619	0.076769
27	oar3_OAR›	47619674	0.015111
27	oar3_OAR›	47625390	0.000363
27	oar3_OAR›	47632615	0.000363
27	oar3_OAR›	47632623	0.019032
27	oar3_OAR›	47637634	0.019032
27	oar3_OAR›	47664874	-0.02144
27	oar3_OAR›	47669876	-0.00942
27	oar3_OAR›	47697044	0.020697
27	oar3_OAR›	47707985	0.075873
27	oar3_OAR›	47709981	-0.00259
27	oar3_OAR›	47755770	-0.00259
27	oar3_OAR›	47784549	-0.00822
27	oar3_OAR›	47785631	-0.00577
27	oar3_OAR›	47793452	-0.00577
27	oar3_OAR›	47795983	0.000954
27	oar3_OAR›	47799736	0.000954
27	oar3_OAR›	47823098	-0.00617
27	oar3_OAR›	47827967	-0.01805
27	oar3_OAR›	47833213	-0.00617
27	oar3_OAR›	47835017	-0.01805
27	oar3_OAR›	47846964	-0.00617
27	oar3_OAR›	47847440	-0.00617
27	oar3_OAR›	47855262	0.146474
27	oar3_OAR›	47862986	0.052414

27	oar3_OAR›	47869599	0.052414
27	oar3_OAR›	47875978	0.036114
27	oar3_OAR›	47950192	0.036114
27	oar3_OAR›	47951058	-0.00163
27	oar3_OAR›	47955566	0.036114
27	oar3_OAR›	47966844	0.036114
27	oar3_OAR›	47977899	0.036114
27	oar3_OAR›	47984468	0.036114
27	oar3_OAR›	47984983	0.036114
27	oar3_OAR›	47989837	0.036114
27	oar3_OAR›	47994470	0.014165
27	oar3_OAR›	47995142	0.036114
27	oar3_OAR›	48012342	0.109422
27	oar3_OAR›	48062094	0.042554
27	oar3_OAR›	48068754	0.042554
27	oar3_OAR›	48076613	0.042047
27	oar3_OAR›	48077009	0.042047
27	oar3_OAR›	48093516	0.067821
27	oar3_OAR›	48097007	0.013555
27	oar3_OAR›	48098002	0.067821
27	oar3_OAR›	48102735	0.013555
27	oar3_OAR›	48105289	0.067821
27	oar3_OAR›	48181871	-0.00411
27	oar3_OAR›	48203328	0.059272
27	oar3_OAR›	48204887	5.86E-06
27	oar3_OAR›	48207552	-0.01488
27	oar3_OAR›	48262526	0.012513
27	oar3_OAR›	48264191	-0.01488
27	oar3_OAR›	48269738	-0.00674
27	oar3_OAR›	48274092	-0.00674
27	oar3_OAR›	48281520	-0.00685
27	oar3_OAR›	48285530	-0.00674
27	oar3_OAR›	48289481	-0.01488
27	oar3_OAR›	48289642	-0.00685
27	oar3_OAR›	48329140	0.114196
27	oar3_OAR›	48331810	0.08352
27	oar3_OAR›	48346211	0.114766
27	oar3_OAR›	48352274	0.091167
27	oar3_OAR›	48354329	0.046691
27	oar3_OAR›	48376559	0.015856
27	oar3_OAR›	48434388	0.096361
27	oar3_OAR›	48439301	-0.00535
27	oar3_OAR›	48457729	0.001262
27	oar3_OAR›	48506162	0.082792
27	oar3_OAR›	48517422	0.007039
27	oar3_OAR›	48553324	-0.02759
27	oar3_OAR›	48566158	0.082792
27	oar3_OAR›	48590641	-0.01252
27	oar3_OAR›	48640219	-0.01902
27	oar3_OAR›	48668057	-0.02662
27	oar3_OAR›	48673911	0.119202
27	oar3_OAR›	48702441	0.119202
27	oar3_OAR›	48747155	-0.02184
27	oar3_OAR›	48753718	0.128882

27	oar3_OAR›	48780088	0.140181
27	oar3_OAR›	48786287	0.128882
27	oar3_OAR›	48796973	0.140181
27	oar3_OAR›	48811007	0.104667
27	oar3_OAR›	48823492	0.053951
27	oar3_OAR›	48826470	0.160975
27	oar3_OAR›	48837831	0.135329
27	oar3_OAR›	48838283	-0.02575
27	oar3_OAR›	48845166	0.162446
27	oar3_OAR›	48869489	0.067483
27	oar3_OAR›	48876636	0.175457
27	oar3_OAR›	48897553	0.069458
27	oar3_OAR›	48899998	0.175457
27	oar3_OAR›	48929198	0.210133
27	oar3_OAR›	48938061	0.210133
27	oar3_OAR›	48948757	-0.02926
27	oar3_OAR›	48963714	-0.02184
27	oar3_OAR›	48964101	-0.00928
27	oar3_OAR›	48984603	-0.01423
27	oar3_OAR›	48990384	0.050337
27	oar3_OAR›	48990830	0.210133
27	oar3_OAR›	49002971	-0.00252
27	oar3_OAR›	49010278	-0.02063
27	oar3_OAR›	49037764	0.047633
27	oar3_OAR›	49048379	-0.01977
27	oar3_OAR›	49072686	-0.007
27	oar3_OAR›	49074187	0.090873
27	oar3_OAR›	49087258	-0.0221
27	oar3_OAR›	49118149	0.047633
27	oar3_OAR›	49146380	0.062852
27	oar3_OAR›	49154618	0.015808
27	oar3_OAR›	49191843	0.068069
27	oar3_OAR›	49214808	0.001866
27	oar3_OAR›	49233644	-0.00449
27	oar3_OAR›	49233842	0.050019
27	oar3_OAR›	49235840	-0.02593
27	oar3_OAR›	49252916	0.050828
27	oar3_OAR›	49272561	0.024821
27	oar3_OAR›	49286407	-0.0048
27	oar3_OAR›	49306643	-0.01386
27	oar3_OAR›	49327632	0.004568
27	oar3_OAR›	49339797	-0.00761
27	oar3_OAR›	49342652	-0.01386
27	oar3_OAR›	49350660	-0.01386
27	oar3_OAR›	49352477	-0.00416
27	oar3_OAR›	49361425	0.012253
27	oar3_OAR›	49380826	0.027147
27	oar3_OAR›	49382016	0.004968
27	oar3_OAR›	49382162	0.008193
27	oar3_OAR›	49387889	0.04355
27	oar3_OAR›	49391519	-0.02822
27	oar3_OAR›	49397084	0.04355
27	oar3_OAR›	49406891	0.008559
27	oar3_OAR›	49415209	-0.01674

27	oar3_OAR›	49417223	-0.00673
27	oar3_OAR›	49466505	-0.00181
27	oar3_OAR›	49481324	-0.02278
27	oar3_OAR›	49492641	0.044635
27	oar3_OAR›	49516778	0.076631
27	oar3_OAR›	49518555	-0.03031
27	oar3_OAR›	49520966	0.07267
27	oar3_OAR›	49525342	-0.03326
27	oar3_OAR›	49532184	-0.02416
27	oar3_OAR›	49532704	0.038177
27	oar3_OAR›	49541031	0.025734
27	oar3_OAR›	49542774	-0.01997
27	oar3_OAR›	49553980	0.051707
27	oar3_OAR›	49562918	-0.00463
27	oar3_OAR›	49568114	0.046787
27	oar3_OAR›	49570165	0.082283
27	oar3_OAR›	49581688	0.157374
27	oar3_OAR›	49587723	NA
27	oar3_OAR›	49592726	0.039051
27	oar3_OAR›	49599233	0.031649
27	oar3_OAR›	49602710	-0.0131
27	oar3_OAR›	49612039	0.015732
27	oar3_OAR›	49616136	-0.01069
27	oar3_OAR›	49616260	0.01079
27	oar3_OAR›	49619730	-0.01551
27	oar3_OAR›	49620961	-0.01737
27	oar3_OAR›	49630977	-0.00606
27	oar3_OAR›	49632027	0.006752
27	oar3_OAR›	49636420	0.000363
27	oar3_OAR›	49636506	-0.0093
27	oar3_OAR›	49640381	0.000889
27	oar3_OAR›	49647277	-0.02608
27	oar3_OAR›	49649818	0.010717
27	oar3_OAR›	49659028	0.013955
27	oar3_OAR›	49757674	-0.00585
27	oar3_OAR›	49780869	-0.01789
27	oar3_OAR›	49786071	-0.02926
27	oar3_OAR›	49788912	-0.02926
27	oar3_OAR›	49842086	-0.00748
27	oar3_OAR›	49845820	0.029811
27	oar3_OAR›	49856081	0.037433
27	oar3_OAR›	49871326	0.029811
27	oar3_OAR›	49973300	0.017568
27	oar3_OAR›	49984152	-0.01777
27	oar3_OAR›	49984670	0.017568
27	oar3_OAR›	50012338	-0.00152
27	oar3_OAR›	50018513	-0.00712
27	oar3_OAR›	50033647	-0.00928
27	oar3_OAR›	50036056	0.008032
27	oar3_OAR›	50049638	-0.03171
27	oar3_OAR›	50067897	0.009985
27	oar3_OAR›	50091470	0.010744
27	oar3_OAR›	50140368	0.010744
27	oar3_OAR›	50140437	0.016238

27	oar3_OAR›	50169949	0.016238
27	oar3_OAR›	50170570	-0.02159
27	oar3_OAR›	50176547	-0.01667
27	oar3_OAR›	50181848	0.058813
27	oar3_OAR›	50181973	-0.01667
27	oar3_OAR›	50194969	0.00549
27	oar3_OAR›	50198148	0.080332
27	oar3_OAR›	50210106	0.00549
27	oar3_OAR›	50229495	0.085044
27	oar3_OAR›	50245920	-0.00928
27	oar3_OAR›	50246578	-0.00928
27	oar3_OAR›	50246730	0.005125
27	oar3_OAR›	50275995	0.015856
27	oar3_OAR›	50281741	-0.02242
27	oar3_OAR›	50282199	0.013963
27	oar3_OAR›	50300341	0.006953
27	oar3_OAR›	50303058	-0.00188
27	oar3_OAR›	50303143	-0.00188
27	oar3_OAR›	50303402	-0.00188
27	oar3_OAR›	50314509	-0.02533
27	oar3_OAR›	50314554	-0.02533
27	oar3_OAR›	50315923	-0.00898
27	oar3_OAR›	50320709	-0.027
27	oar3_OAR›	50323657	-0.03382
27	oar3_OAR›	50328006	-0.02989
27	oar3_OAR›	50328087	-0.02989
27	oar3_OAR›	50329990	-0.00751
27	oar3_OAR›	50344516	-0.02989
27	oar3_OAR›	50345552	-0.00751
27	oar3_OAR›	50353251	-0.00941
27	oar3_OAR›	50355381	-0.03354
27	oar3_OAR›	50370197	-0.03456
27	oar3_OAR›	50371600	-0.03354
27	oar3_OAR›	50380730	-0.03369
27	oar3_OAR›	50387403	-0.0347
27	oar3_OAR›	50389528	-0.03147
27	oar3_OAR›	50399676	-0.02546
27	oar3_OAR›	50414676	-0.00239
27	oar3_OAR›	50420917	-0.00239
27	oar3_OAR›	50421725	-0.00239
27	oar3_OAR›	50421961	-0.00239
27	oar3_OAR›	50425029	0.025929
27	oar3_OAR›	50442181	0.080866
27	oar3_OAR›	50446540	-0.0385
27	oar3_OAR›	50447084	0.072943
27	oar3_OAR›	50453683	0.053264
27	oar3_OAR›	50453983	0.053264
27	oar3_OAR›	50463493	-0.00155
27	oar3_OAR›	50483859	-0.00389
27	oar3_OAR›	50484793	0.040678
27	oar3_OAR›	50488498	-0.0141
27	oar3_OAR›	50494362	0.040678
27	oar3_OAR›	50496813	0.028008
27	oar3_OAR›	50506431	0.021132

27	oar3_OAR›	50506796	-0.02279
27	oar3_OAR›	50506873	0.040678
27	oar3_OAR›	50507294	0.021132
27	oar3_OAR›	50523416	-0.03079
27	oar3_OAR›	50527682	0.016273
27	oar3_OAR›	50533196	-0.01295
27	oar3_OAR›	50557383	-0.03848
27	oar3_OAR›	50559477	0.027354
27	oar3_OAR›	50562345	-0.03848
27	oar3_OAR›	50581730	0.005453
27	oar3_OAR›	50582575	0.034576
27	oar3_OAR›	50591810	-0.01613
27	oar3_OAR›	50600875	-0.02902
27	oar3_OAR›	50614860	-0.0025
27	oar3_OAR›	50615984	-0.02528
27	oar3_OAR›	50631300	0.01771
27	oar3_OAR›	50639087	0.000753
27	oar3_OAR›	50674637	-0.00994
27	oar3_OAR›	50697622	-0.00665
27	oar3_OAR›	50699465	-0.01494
27	oar3_OAR›	50701483	0.009186
27	oar3_OAR›	50702005	-0.0297
27	oar3_OAR›	50713818	0.004259
27	oar3_OAR›	50717975	-0.00705
27	oar3_OAR›	50727488	-0.0085
27	oar3_OAR›	50727695	-0.00665
27	oar3_OAR›	50796654	-0.00705
27	oar3_OAR›	50814456	-0.02251
27	oar3_OAR›	50814578	0.03105
27	oar3_OAR›	50819703	-0.02251
27	oar3_OAR›	50827445	0.054905
27	oar3_OAR›	50830500	0.054905
27	oar3_OAR›	50838563	0.054905
27	oar3_OAR›	50852168	0.054905
27	oar3_OAR›	50852578	0.054905
27	oar3_OAR›	50877746	0.042629
27	oar3_OAR›	50949406	-0.01489
27	oar3_OAR›	50953659	-0.0116
27	oar3_OAR›	50953787	-0.03116
27	oar3_OAR›	50971365	0.061173
27	oar3_OAR›	50981767	-0.02077
27	oar3_OAR›	50997976	-0.02332
27	oar3_OAR›	51002866	0.026909
27	oar3_OAR›	51006210	0.053029
27	oar3_OAR›	51015427	0.044009
27	oar3_OAR›	51015715	0.073912
27	oar3_OAR›	51023508	0.065057
27	oar3_OAR›	51031555	0.065057
27	oar3_OAR›	51077358	-0.00882
27	oar3_OAR›	51146427	-0.01574
27	oar3_OAR›	51146494	-0.02661
27	oar3_OAR›	51150323	-0.03663
27	oar3_OAR›	51151648	-0.00229
27	oar3_OAR›	51167640	-0.03646

27	oar3_OAR>	51168301	-0.03991
27	oar3_OAR>	51171749	-0.01694
27	oar3_OAR>	51173604	-0.0312
27	oar3_OAR>	51179396	-0.03954
27	oar3_OAR>	51179706	-0.00229
27	oar3_OAR>	51189780	-0.01034
27	oar3_OAR>	51195419	-0.03575
27	oar3_OAR>	51198523	-0.01034
27	oar3_OAR>	51200501	-0.01538
27	oar3_OAR>	51207032	-0.02017
27	oar3_OAR>	51207067	-0.02017
27	oar3_OAR>	51210140	-0.02017
27	oar3_OAR>	51212082	-0.02017
27	oar3_OAR>	51217335	0.002815
27	oar3_OAR>	51217411	0.002815
27	oar3_OAR>	51224802	0.044724
27	oar3_OAR>	51224877	0.002815
27	oar3_OAR>	51225136	-0.01634
27	oar3_OAR>	51234386	0.161614
27	oar3_OAR>	51235074	0.131433
27	oar3_OAR>	51242340	0.131433
27	oar3_OAR>	51245116	0.131433
27	oar3_OAR>	51246055	0.131433
27	oar3_OAR>	51261334	-0.01737
27	oar3_OAR>	51261754	0.230657
27	oar3_OAR>	51274945	-0.01737
27	oar3_OAR>	51277320	0.274284
27	oar3_OAR>	51287635	0.274284
27	oar3_OAR>	51288001	0.274284
27	oar3_OAR>	51295747	0.239119
27	oar3_OAR>	51299246	0.239119
27	oar3_OAR>	51309717	0.242976
27	oar3_OAR>	51309926	0.202925
27	oar3_OAR>	51311309	0.242976
27	oar3_OAR>	51325528	0.242976
27	oar3_OAR>	51328191	0.242976
27	oar3_OAR>	51328932	0.242976
27	oar3_OAR>	51342165	0.169242
27	oar3_OAR>	51343037	0.197924
27	oar3_OAR>	51343163	0.197924
27	oar3_OAR>	51350568	0.197924
27	oar3_OAR>	51360298	0.239119
27	oar3_OAR>	51373437	0.274284
27	oar3_OAR>	51389840	0.201744
27	oar3_OAR>	51393286	0.274284
27	oar3_OAR>	51393715	0.230657
27	oar3_OAR>	51405562	-0.03298
27	oar3_OAR>	51409869	0.300012
27	oar3_OAR>	51410392	0.300012
27	oar3_OAR>	51412076	0.300012
27	oar3_OAR>	51419926	0.26421
27	oar3_OAR>	51422415	0.26421
27	oar3_OAR>	51425340	-0.03417
27	oar3_OAR>	51457541	-0.01737

27	oar3_OAR›	51458903	0.26421
27	oar3_OAR›	51484397	0.201744
27	oar3_OAR›	51484465	0.26421
27	oar3_OAR›	51484743	-0.03327
27	oar3_OAR›	51493252	0.201744
27	oar3_OAR›	51527837	0.197924
27	oar3_OAR›	51528450	0.197924
27	oar3_OAR›	51534152	0.197924
27	oar3_OAR›	51540560	0.197924
27	oar3_OAR›	51540912	0.197924
27	oar3_OAR›	51545115	0.231623
27	oar3_OAR›	51580160	-0.02804
27	oar3_OAR›	51608457	0.243922
27	oar3_OAR›	51616841	0.243922
27	oar3_OAR›	51620356	0.243922
27	oar3_OAR›	51621131	0.243922
27	oar3_OAR›	51623984	0.243922
27	oar3_OAR›	51630109	0.243922
27	oar3_OAR›	51640628	0.243922
27	oar3_OAR›	51640908	0.128981
27	oar3_OAR›	51642166	-0.02784
27	oar3_OAR›	51650974	-0.02784
27	oar3_OAR›	51652646	0.243922
27	oar3_OAR›	51657821	-0.03941
27	oar3_OAR›	51675069	0.153095
27	oar3_OAR›	51675968	0.129698
27	oar3_OAR›	51686420	0.041588
27	oar3_OAR›	51690749	0.014412
27	oar3_OAR›	51691049	0.014412
27	oar3_OAR›	51693006	0.014412
27	oar3_OAR›	51697019	-0.01093
27	oar3_OAR›	51698730	0.005993
27	oar3_OAR›	51702429	0.014412
27	oar3_OAR›	51705470	-0.00838
27	oar3_OAR›	51706359	-0.01913
27	oar3_OAR›	51711722	-0.00417
27	oar3_OAR›	51717057	0.024528
27	oar3_OAR›	51726609	-0.02651
27	oar3_OAR›	51737981	0.001368
27	oar3_OAR›	51738199	0.001368
27	oar3_OAR›	51748831	0.001368
27	oar3_OAR›	51749694	-0.00272
27	oar3_OAR›	51750528	-0.00272
27	oar3_OAR›	51759803	-0.00272
27	oar3_OAR›	51760017	-0.00272
27	oar3_OAR›	51803409	0.000813
27	oar3_OAR›	51803537	-0.0002
27	oar3_OAR›	51807424	0.021564
27	oar3_OAR›	51810022	0.003537
27	oar3_OAR›	51836051	-0.006
27	oar3_OAR›	51844248	0.000363
27	oar3_OAR›	51847311	0.000363
27	oar3_OAR›	51863252	-0.00673
27	oar3_OAR›	51877261	0.03802

27	oar3_OAR›	51889841	0.03802
27	oar3_OAR›	51899779	0.014713
27	oar3_OAR›	51900815	0.000363
27	oar3_OAR›	51923586	0.000363
27	oar3_OAR›	51923864	0.04423
27	oar3_OAR›	51927808	-0.00673
27	oar3_OAR›	51935216	0.003805
27	oar3_OAR›	51936257	0.003805
27	oar3_OAR›	51946317	0.003805
27	oar3_OAR›	51954092	0.003805
27	oar3_OAR›	51975446	-0.00204
27	oar3_OAR›	51978998	0.038571
27	oar3_OAR›	51989046	-0.00099
27	oar3_OAR›	51994689	-0.00928
27	oar3_OAR›	51995586	0.000363
27	oar3_OAR›	52001651	-0.00389
27	oar3_OAR›	52005868	0.008343
27	oar3_OAR›	52006532	-0.00389
27	oar3_OAR›	52006636	-0.00389
27	oar3_OAR›	52008643	0.009761
27	oar3_OAR›	52011595	0.000363
27	oar3_OAR›	52016463	0.017167
27	oar3_OAR›	52016694	-0.03036
27	oar3_OAR›	52021869	-0.00333
27	oar3_OAR›	52025905	-0.00333
27	oar3_OAR›	52035167	0.000147
27	oar3_OAR›	52038021	-0.02306
27	oar3_OAR›	52040697	-0.02306
27	oar3_OAR›	52042335	-0.02507
27	oar3_OAR›	52053802	-0.02879
27	oar3_OAR›	52053955	-0.02493
27	oar3_OAR›	52058788	0.000363
27	oar3_OAR›	52062611	0.040355
27	oar3_OAR›	52068830	0.000363
27	oar3_OAR›	52072201	-0.02737
27	oar3_OAR›	52072851	0.017268
27	oar3_OAR›	52076166	0.037131
27	oar3_OAR›	52084811	0.037131
27	oar3_OAR›	52085524	-0.00841
27	oar3_OAR›	52089478	-0.0319
27	oar3_OAR›	52097396	-0.00289
27	oar3_OAR›	52112872	-0.03142
27	oar3_OAR›	52123582	0.001402
27	oar3_OAR›	52125628	-0.02662
27	oar3_OAR›	52135625	0.027637
27	oar3_OAR›	52135704	0.000363
27	oar3_OAR›	52139330	-0.02038
27	oar3_OAR›	52143224	0.085694
27	oar3_OAR›	52154756	0.048366
27	oar3_OAR›	52155333	0.076273
27	oar3_OAR›	52164662	-0.02759
27	oar3_OAR›	52168283	-0.02897
27	oar3_OAR›	52175659	-0.01704
27	oar3_OAR›	52175777	-0.02136

27	oar3_OAR›	52180724	-0.02744
27	oar3_OAR›	52182361	0.039953
27	oar3_OAR›	52191890	0.014112
27	oar3_OAR›	52193309	-0.01295
27	oar3_OAR›	52214943	-0.02182
27	oar3_OAR›	52219286	-0.00849
27	oar3_OAR›	52225308	0.120012
27	oar3_OAR›	52225710	-0.00849
27	oar3_OAR›	52236567	0.040093
27	oar3_OAR›	52237616	0.040093
27	oar3_OAR›	52244748	-0.00849
27	oar3_OAR›	52250858	-0.01172
27	oar3_OAR›	52256782	-0.03442
27	oar3_OAR›	52256854	0.004711
27	oar3_OAR›	52264398	-0.02948
27	oar3_OAR›	52264618	-0.02379
27	oar3_OAR›	52265065	0.106592
27	oar3_OAR›	52268888	0.078974
27	oar3_OAR›	52270994	-0.00606
27	oar3_OAR›	52274144	0.078974
27	oar3_OAR›	52279306	0.074964
27	oar3_OAR›	52283354	-0.0142
27	oar3_OAR›	52283880	0.040361
27	oar3_OAR›	52291372	-0.01415
27	oar3_OAR›	52300481	-0.02926
27	oar3_OAR›	52326169	-0.0196
27	oar3_OAR›	52326731	-0.00735
27	oar3_OAR›	52329100	-0.01737
27	oar3_OAR›	52341872	-0.00606
27	oar3_OAR›	52353815	-0.00606
27	oar3_OAR›	52363848	0.067884
27	oar3_OAR›	52367144	0.05932
27	oar3_OAR›	52367253	0.05932
27	oar3_OAR›	52369274	-0.01737
27	oar3_OAR›	52374201	-0.01406
27	oar3_OAR›	52375770	-0.02575
27	oar3_OAR›	52380191	0.003466
27	oar3_OAR›	52381296	0.010803
27	oar3_OAR›	52383033	0.003466
27	oar3_OAR›	52391649	0.040966
27	oar3_OAR›	52393902	0.001337
27	oar3_OAR›	52394199	-0.01737
27	oar3_OAR›	52395336	0.006445
27	oar3_OAR›	52403806	-0.01902
27	oar3_OAR›	52403919	0.006445
27	oar3_OAR›	52405359	-0.01061
27	oar3_OAR›	52409976	0.006445
27	oar3_OAR›	52417327	-0.02063
27	oar3_OAR›	52417572	0.006445
27	oar3_OAR›	52418919	0.00365
27	oar3_OAR›	52420065	-0.02152
27	oar3_OAR›	52425436	0.009217
27	oar3_OAR›	52428273	0.006445
27	oar3_OAR›	52429403	0.044085

27	oar3_OAR›	52433555	-0.01186
27	oar3_OAR›	52436373	-0.00113
27	oar3_OAR›	52438959	0.006682
27	oar3_OAR›	52446370	-0.01737
27	oar3_OAR›	52447969	0.010758
27	oar3_OAR›	52448058	0.017521
27	oar3_OAR›	52459332	0.010758
27	oar3_OAR›	52462248	0.010758
27	oar3_OAR›	52468424	0.020267
27	oar3_OAR›	52478473	0.030596
27	oar3_OAR›	52480699	0.010758
27	oar3_OAR›	52482851	0.030596
27	oar3_OAR›	52495168	-0.01069
27	oar3_OAR›	52498353	-0.00363
27	oar3_OAR›	52512543	-0.01551
27	oar3_OAR›	52514036	-0.00113
27	oar3_OAR›	52514803	0.006682
27	oar3_OAR›	52523380	0.010758
27	oar3_OAR›	52531431	-0.01069
27	oar3_OAR›	52550232	-0.01069
27	oar3_OAR›	52551151	0.060237
27	oar3_OAR›	52558775	0.010758
27	oar3_OAR›	52563849	0.011067
27	oar3_OAR›	52580137	0.011067
27	oar3_OAR›	52585319	0.156235
27	oar3_OAR›	52586747	0.029591
27	oar3_OAR›	52594190	0.017727
27	oar3_OAR›	52606182	0.006708
27	oar3_OAR›	52607983	NA
27	oar3_OAR›	52618291	NA
27	oar3_OAR›	52618291	NA
27	oar3_OAR›	52620245	NA
27	oar3_OAR›	52640601	0.185546
27	oar3_OAR›	52643727	NA
27	oar3_OAR›	52656271	-0.00965
27	oar3_OAR›	52673230	0.078106
27	oar3_OAR›	52677278	-0.00409
27	oar3_OAR›	52679567	0.185546
27	oar3_OAR›	52686253	-0.01872
27	oar3_OAR›	52691970	0.010733
27	oar3_OAR›	52695888	NA
27	oar3_OAR›	52698005	-0.01872
27	oar3_OAR›	52727537	-0.00788
27	oar3_OAR›	52735669	NA
27	oar3_OAR›	52738139	-0.00606
27	oar3_OAR›	52753304	0.052246
27	oar3_OAR›	52772673	0.002868
27	oar3_OAR›	52779630	0.056621
27	oar3_OAR›	52793215	-0.00462
27	oar3_OAR›	52801389	0.10565
27	oar3_OAR›	52801454	-0.01423
27	oar3_OAR›	52811024	-0.02562
27	oar3_OAR›	52821903	0.039229
27	oar3_OAR›	52890081	-0.00134

27	oar3_OAR›	52911524	-0.00653
27	oar3_OAR›	52916490	-0.00932
27	oar3_OAR›	52925005	0.055664
27	oar3_OAR›	52931059	-0.00932
27	oar3_OAR›	52938860	0.055664
27	oar3_OAR›	52939190	0.032517
27	oar3_OAR›	52963790	-0.0092
27	oar3_OAR›	52974171	0.102102
27	oar3_OAR›	52975857	-0.01891
27	oar3_OAR›	52988981	-0.0206
27	oar3_OAR›	52994643	-0.0206
27	oar3_OAR›	52998416	0.241187
27	oar3_OAR›	52999167	0.170523
27	oar3_OAR›	53001153	-0.01356
27	oar3_OAR›	53009102	0.241187
27	oar3_OAR›	53009915	-0.00363
27	oar3_OAR›	53021120	0.142165
27	oar3_OAR›	53030293	0.136954
27	oar3_OAR›	53067802	0.457378
27	oar3_OAR›	53085894	0.14184
27	oar3_OAR›	53085937	0.189404
27	oar3_OAR›	53086728	-0.00239
27	oar3_OAR›	53091574	-0.00239
27	oar3_OAR›	53098230	0.166882
27	oar3_OAR›	53099405	-0.0043
27	oar3_OAR›	53101692	-0.00199
27	oar3_OAR›	53103840	0.03648
27	oar3_OAR›	53109070	0.03648
27	oar3_OAR›	53110603	0.016364
27	oar3_OAR›	53110845	-0.00199
27	oar3_OAR›	53114587	-0.00659
27	oar3_OAR›	53115096	0.085932
27	oar3_OAR›	53119906	0.005693
27	oar3_OAR›	53120739	-0.01731
27	oar3_OAR›	53121667	0.005693
27	oar3_OAR›	53123027	0.005693
27	oar3_OAR›	53126100	0.016364
27	oar3_OAR›	53128367	0.005693
27	oar3_OAR›	53132994	0.058535
27	oar3_OAR›	53133028	0.058535
27	oar3_OAR›	53138606	-0.02786
27	oar3_OAR›	53148623	-0.03172
27	oar3_OAR›	53159716	-0.03413
27	oar3_OAR›	53159718	-0.03058
27	oar3_OAR›	53165036	-0.02466
27	oar3_OAR›	53166302	-0.01224
27	oar3_OAR›	53182680	0.131657
27	oar3_OAR›	53183116	0.103091
27	oar3_OAR›	53195858	-0.00606
27	oar3_OAR›	53196681	-0.00673
27	oar3_OAR›	53198832	-0.01737
27	oar3_OAR›	53206449	0.06279
27	oar3_OAR›	53212356	0.06279
27	oar3_OAR›	53215215	-0.01737

27	oar3_OAR›	53217807	-0.01737
27	oar3_OAR›	53228120	0.004461
27	oar3_OAR›	53228514	0.004461
27	oar3_OAR›	53257527	0.146161
27	oar3_OAR›	53273436	0.168031
27	oar3_OAR›	53275152	0.168031
27	oar3_OAR›	53275462	0.138029
27	oar3_OAR›	53281212	-0.01612
27	oar3_OAR›	53287408	0.098557
27	oar3_OAR›	53292384	0.10636
27	oar3_OAR›	53299308	0.10636
27	oar3_OAR›	53309043	-0.01612
27	oar3_OAR›	53309763	0.260363
27	oar3_OAR›	53313829	0.067382
27	oar3_OAR›	53341428	0.245447
27	oar3_OAR›	53348964	0.165097
27	oar3_OAR›	53355419	0.245447
27	oar3_OAR›	53356977	0.245447
27	oar3_OAR›	53357931	0.245447
27	oar3_OAR›	53377180	0.229523
27	oar3_OAR›	53408802	0.316967
27	oar3_OAR›	53418019	0.237657
27	oar3_OAR›	53419962	-0.01737
27	oar3_OAR›	53426035	0.295988
27	oar3_OAR›	53429833	0.253452
27	oar3_OAR›	53433767	0.277824
27	oar3_OAR›	53434418	-0.02526
27	oar3_OAR›	53457378	0.098602
27	oar3_OAR›	53468533	0.157013
27	oar3_OAR›	53484584	0.184353
27	oar3_OAR›	53490425	-0.01737
27	oar3_OAR›	53506254	0.245447
27	oar3_OAR›	53508427	0.022582
27	oar3_OAR›	53510634	0.245447
27	oar3_OAR›	53512304	0.022582
27	oar3_OAR›	53523704	0.022582
27	oar3_OAR›	53523811	0.303286
27	oar3_OAR›	53529359	0.245447
27	oar3_OAR›	53529519	0.022582
27	oar3_OAR›	53533205	0.022582
27	oar3_OAR›	53548960	0.072293
27	oar3_OAR›	53549662	-0.01737
27	oar3_OAR›	53573418	0.229523
27	oar3_OAR›	53573654	0.238495
27	oar3_OAR›	53574908	0.253452
27	oar3_OAR›	53578625	-0.02279
27	oar3_OAR›	53587470	-0.03375
27	oar3_OAR›	53595494	0.371303
27	oar3_OAR›	53595990	0.324825
27	oar3_OAR›	53606944	0.343687
27	oar3_OAR›	53619641	-0.01939
27	oar3_OAR›	53624640	-0.02064
27	oar3_OAR›	53624785	0.253959
27	oar3_OAR›	53662490	0.182927

27	oar3_OAR〉	53663005	0.23958
27	oar3_OAR〉	53672289	0.117192
27	oar3_OAR〉	53673224	0.216114
27	oar3_OAR〉	53696169	-0.01082
27	oar3_OAR〉	53697377	-0.01082
27	oar3_OAR〉	53699821	-0.01295
27	oar3_OAR〉	53719826	0.22427
27	oar3_OAR〉	53732771	-0.02222
27	oar3_OAR〉	53751604	0.275459
27	oar3_OAR〉	53756339	0.276231
27	oar3_OAR〉	53756683	0.276231
27	oar3_OAR〉	53761845	0.337193
27	oar3_OAR〉	53767245	0.29799
27	oar3_OAR〉	53770810	0.29799
27	oar3_OAR〉	53771894	0.26957
27	oar3_OAR〉	53778639	0.337193
27	oar3_OAR〉	53791831	0.337193
27	oar3_OAR〉	53803946	0.327217
27	oar3_OAR〉	53821358	0.363954
27	oar3_OAR〉	53821803	0.015055
27	oar3_OAR〉	53830882	0.229756
27	oar3_OAR〉	53832288	0.352273
27	oar3_OAR〉	53833211	0.352273
27	oar3_OAR〉	53851600	0.10206
27	oar3_OAR〉	53853620	0.060603
27	oar3_OAR〉	53858607	0.127413
27	oar3_OAR〉	53864437	0.10206
27	oar3_OAR〉	53871318	0.299156
27	oar3_OAR〉	53884458	0.115437
27	oar3_OAR〉	53889311	0.150763
27	oar3_OAR〉	53892822	0.044908
27	oar3_OAR〉	53905571	-0.01998
27	oar3_OAR〉	53905939	0.111325
27	oar3_OAR〉	53907984	0.058546
27	oar3_OAR〉	53912391	0.015055
27	oar3_OAR〉	53931382	0.07445
27	oar3_OAR〉	53957601	-0.04136
27	oar3_OAR〉	53960884	0.158595
27	oar3_OAR〉	53977145	0.218238
27	oar3_OAR〉	53978490	0.032181
27	oar3_OAR〉	54021704	0.166572
27	oar3_OAR〉	54022729	0.166572
27	oar3_OAR〉	54024353	0.371303
27	oar3_OAR〉	54031178	-0.00123
27	oar3_OAR〉	54033594	0.166572
27	oar3_OAR〉	54037379	-0.02355
27	oar3_OAR〉	54045875	0.2902
27	oar3_OAR〉	54046684	-0.0433
27	oar3_OAR〉	54052273	0.155817
27	oar3_OAR〉	54053339	-0.02306
27	oar3_OAR〉	54058073	-0.04159
27	oar3_OAR〉	54069421	-0.01748
27	oar3_OAR〉	54071344	0.3383
27	oar3_OAR〉	54091480	0.195031

27	oar3_OAR›	54098207	0.043817
27	oar3_OAR›	54106061	0.085494
27	oar3_OAR›	54116119	0.018354
27	oar3_OAR›	54122715	-0.01194
27	oar3_OAR›	54133935	-0.01567
27	oar3_OAR›	54134826	-0.0213
27	oar3_OAR›	54140278	0.117003
27	oar3_OAR›	54142249	0.001581
27	oar3_OAR›	54155460	-0.0172
27	oar3_OAR›	54156702	-0.0041
27	oar3_OAR›	54160883	0.083393
27	oar3_OAR›	54162911	0.028212
27	oar3_OAR›	54170606	0.010485
27	oar3_OAR›	54178457	0.007863
27	oar3_OAR›	54183989	0.012768
27	oar3_OAR›	54187939	0.025478
27	oar3_OAR›	54194849	0.00851
27	oar3_OAR›	54196753	0.00851
27	oar3_OAR›	54210815	0.016379
27	oar3_OAR›	54231464	0.041365
27	oar3_OAR›	54234818	0.041365
27	oar3_OAR›	54235463	0.0204
27	oar3_OAR›	54247012	-0.02221
27	oar3_OAR›	54248427	-0.01643
27	oar3_OAR›	54248572	-0.02221
27	oar3_OAR›	54263702	0.037237
27	oar3_OAR›	54281080	0.053884
27	oar3_OAR›	54287749	0.031532
27	oar3_OAR›	54296800	-0.00652
27	oar3_OAR›	54300080	0.027403
27	oar3_OAR›	54312234	0.045466
27	oar3_OAR›	54320675	-0.02633
27	oar3_OAR›	54332798	-0.01916
27	oar3_OAR›	54336819	-0.00396
27	oar3_OAR›	54338409	-0.00396
27	oar3_OAR›	54344857	-0.01139
27	oar3_OAR›	54349438	0.037521
27	oar3_OAR›	54360370	-0.00396
27	oar3_OAR›	54381542	-0.00396
27	oar3_OAR›	54397779	0.018772
27	oar3_OAR›	54408024	0.018772
27	oar3_OAR›	54415855	-0.00396
27	oar3_OAR›	54421239	0.035169
27	oar3_OAR›	54423431	0.020903
27	oar3_OAR›	54425357	-0.02186
27	oar3_OAR›	54425522	0.010054
27	oar3_OAR›	54431628	-0.00396
27	oar3_OAR›	54461664	0.016046
27	oar3_OAR›	54467994	0.034383
27	oar3_OAR›	54470657	0.034383
27	oar3_OAR›	54481800	0.008196
27	oar3_OAR›	54485373	0.011871
27	oar3_OAR›	54493165	-0.01935
27	oar3_OAR›	54493919	0.011531

27	oar3_OAR›	54498247	-0.00283
27	oar3_OAR›	54500998	0.02926
27	oar3_OAR›	54508831	0.018287
27	oar3_OAR›	54518383	0.002651
27	oar3_OAR›	54527956	-0.03374
27	oar3_OAR›	54531687	0.0598
27	oar3_OAR›	54532687	0.037007
27	oar3_OAR›	54545952	0.038645
27	oar3_OAR›	54547291	0.113517
27	oar3_OAR›	54547676	0.028437
27	oar3_OAR›	54557295	0.084291
27	oar3_OAR›	54558735	0.024356
27	oar3_OAR›	54559640	0.038645
27	oar3_OAR›	54572564	-0.02646
27	oar3_OAR›	54583937	-0.02575
27	oar3_OAR›	54596278	-0.02646
27	oar3_OAR›	54619142	-0.01859
27	oar3_OAR›	54620940	-0.0068
27	oar3_OAR›	54621106	-0.0068
27	oar3_OAR›	54626614	-0.00703
27	oar3_OAR›	54630956	-0.0206
27	oar3_OAR›	54639111	0.021977
27	oar3_OAR›	54639289	0.025934
27	oar3_OAR›	54640170	0.008805
27	oar3_OAR›	54657876	-0.0233
27	oar3_OAR›	54658127	-0.0233
27	oar3_OAR›	54663326	0.053574
27	oar3_OAR›	54673435	0.031078
27	oar3_OAR›	54676236	0.052096
27	oar3_OAR›	54677498	-0.01445
27	oar3_OAR›	54701361	0.052096
27	oar3_OAR›	54705874	0.028326
27	oar3_OAR›	54706755	0.004591
27	oar3_OAR›	54717029	0.005158
27	oar3_OAR›	54722792	0.004591
27	oar3_OAR›	54722813	0.005158
27	oar3_OAR›	54739883	0.052096
27	oar3_OAR›	54753311	0.052096
27	oar3_OAR›	54754931	0.004591
27	oar3_OAR›	54763433	0.042629
27	oar3_OAR›	54766745	0.011365
27	oar3_OAR›	54768320	0.011365
27	oar3_OAR›	54769353	0.042629
27	oar3_OAR›	54773530	0.01084
27	oar3_OAR›	54780331	0.011365
27	oar3_OAR›	54782811	-0.01356
27	oar3_OAR›	54783050	0.032059
27	oar3_OAR›	54784620	-0.00521
27	oar3_OAR›	54793182	-0.00601
27	oar3_OAR›	54793725	-0.00521
27	oar3_OAR›	54803105	0.043755
27	oar3_OAR›	54804724	-0.00521
27	oar3_OAR›	54809221	0.043755
27	oar3_OAR›	54809372	-0.01234

27	oar3_OAR›	54814301	-0.01234
27	oar3_OAR›	54820440	-0.01234
27	oar3_OAR›	54827631	-0.01737
27	oar3_OAR›	54827863	-0.01737
27	oar3_OAR›	54832867	0.093921
27	oar3_OAR›	54833619	0.051241
27	oar3_OAR›	54837423	0.56981
27	oar3_OAR›	54848787	0.093195
27	oar3_OAR›	54851893	0.036019
27	oar3_OAR›	54859779	0.018466
27	oar3_OAR›	54861508	0.440786
27	oar3_OAR›	54862271	0.440786
27	oar3_OAR›	54921752	0.503078
27	oar3_OAR›	54922414	NA
27	oar3_OAR›	54931621	0.503078
27	oar3_OAR›	54933345	NA
27	oar3_OAR›	54940409	0.534611
27	oar3_OAR›	54946057	0.534611
27	oar3_OAR›	54959181	NA
27	oar3_OAR›	54960225	-0.00673
27	oar3_OAR›	54974555	-0.00673
27	oar3_OAR›	55023453	0.534611
27	oar3_OAR›	55023807	0.534611
27	oar3_OAR›	55025471	-0.01737
27	oar3_OAR›	55051621	-0.01737
27	oar3_OAR›	55052204	0.534611
27	oar3_OAR›	55061138	-0.01737
27	oar3_OAR›	55062243	0.534611
27	oar3_OAR›	55074666	0.534611
27	oar3_OAR›	55080693	0.521628
27	oar3_OAR›	55095663	-0.01737
27	oar3_OAR›	55114823	0.534611
27	oar3_OAR›	55205865	-0.00673
27	oar3_OAR›	55213348	-0.00673
27	oar3_OAR›	55213523	-0.01737
27	oar3_OAR›	55253268	-0.01737
27	oar3_OAR›	55273274	0.274022
27	oar3_OAR›	55273678	0.22603
27	oar3_OAR›	55294598	0.274022
27	oar3_OAR›	55327982	-0.00673
27	oar3_OAR›	55332901	-0.01737
27	oar3_OAR›	55340384	-0.00836
27	oar3_OAR›	55344160	0.018804
27	oar3_OAR›	55365595	-0.00836
27	oar3_OAR›	55374543	-0.00673
27	oar3_OAR›	55397104	-0.0206
27	oar3_OAR›	55397123	0.571746
27	oar3_OAR›	55399614	0.571746
27	oar3_OAR›	55449430	-0.01737
27	oar3_OAR›	55471518	-0.01737
27	oar3_OAR›	55485113	0.546751
27	oar3_OAR›	55485360	-0.01737
27	oar3_OAR›	55485780	0.546751
27	oar3_OAR›	55536736	0.546751

27	oar3_OAR〉	55556160	-0.01737
27	oar3_OAR〉	55558544	0.546751
27	oar3_OAR〉	55561698	0.546751
27	oar3_OAR〉	55569007	-0.01737
27	oar3_OAR〉	55608408	0.000889
27	oar3_OAR〉	55608785	0.546751
27	oar3_OAR〉	55627200	0.546751
27	oar3_OAR〉	55646191	0.531958
27	oar3_OAR〉	55658032	0.531958
27	oar3_OAR〉	55659900	0.000889
27	oar3_OAR〉	55688216	0.546751
27	oar3_OAR〉	55759309	0.546751
27	oar3_OAR〉	55762118	0.531958
27	oar3_OAR〉	55770763	0.546751
27	oar3_OAR〉	55774978	0.000889
27	oar3_OAR〉	55787813	0.000889
27	oar3_OAR〉	55790793	0.546751
27	oar3_OAR〉	55804822	0.597147
27	oar3_OAR〉	55907658	NA
27	oar3_OAR〉	55919600	NA
27	oar3_OAR〉	55990348	0.043755
27	oar3_OAR〉	56030002	0.043755
27	oar3_OAR〉	56036347	0.043755
27	oar3_OAR〉	56100849	0.043755
27	oar3_OAR〉	56103184	0.011365
27	oar3_OAR〉	56120898	0.531958
27	oar3_OAR〉	56136532	0.011365
27	oar3_OAR〉	56165017	0.043755
27	oar3_OAR〉	56254899	0.531958
27	oar3_OAR〉	56258308	0.531958
27	oar3_OAR〉	56268673	0.043755
27	oar3_OAR〉	56328527	NA
27	oar3_OAR〉	56337743	-0.00344
27	oar3_OAR〉	56360750	NA
27	oar3_OAR〉	56367011	0.043755
27	oar3_OAR〉	56401340	0.00661
27	oar3_OAR〉	56403062	NA
27	oar3_OAR〉	56433855	NA
27	oar3_OAR〉	56436701	-0.00344
27	oar3_OAR〉	56453975	0.00661
27	oar3_OAR〉	56465320	0.00661
27	oar3_OAR〉	56483523	0.00661
27	oar3_OAR〉	56493592	0.00661
27	oar3_OAR〉	56504885	-0.00344
27	oar3_OAR〉	56538081	-0.00344
27	oar3_OAR〉	56588829	NA
27	oar3_OAR〉	56592499	0.00661
27	oar3_OAR〉	56609654	NA
27	oar3_OAR〉	56619728	NA
27	oar3_OAR〉	56624555	NA
27	oar3_OAR〉	56625645	NA
27	oar3_OAR〉	56633635	0.032517
27	oar3_OAR〉	56646939	-0.01737
27	oar3_OAR〉	56655440	-0.01737

27	oar3_OAR›	56660103	0.000889
27	oar3_OAR›	56662643	-0.01737
27	oar3_OAR›	56664295	-0.01737
27	oar3_OAR›	56669636	NA
27	oar3_OAR›	56741416	NA
27	oar3_OAR›	56743811	0.000889
27	oar3_OAR›	56753471	NA
27	oar3_OAR›	56756810	0.000889
27	oar3_OAR›	56763288	NA
27	oar3_OAR›	56765431	0.032582
27	oar3_OAR›	56765571	0.032582
27	oar3_OAR›	56769447	NA
27	oar3_OAR›	56774252	0.032582
27	oar3_OAR›	56781093	0.032582
27	oar3_OAR›	56791888	0.032582
27	oar3_OAR›	56793593	NA
27	oar3_OAR›	56797884	0.032582
27	oar3_OAR›	56807424	0.032582
27	oar3_OAR›	56812381	0.032582
27	oar3_OAR›	56815370	0.032582
27	oar3_OAR›	56827620	NA
27	oar3_OAR›	56828987	0.032582
27	oar3_OAR›	56838429	0.032582
27	oar3_OAR›	56841867	0.032582
27	oar3_OAR›	56851215	0.032582
27	oar3_OAR›	56853047	NA
27	oar3_OAR›	56871410	NA
27	oar3_OAR›	56888064	NA
27	oar3_OAR›	56905862	0.032582
27	oar3_OAR›	56914710	0.032582
27	oar3_OAR›	56914955	0.032582
27	oar3_OAR›	56925344	0.032582
27	oar3_OAR›	56928283	0.032582
27	oar3_OAR›	56944640	0.032582
27	oar3_OAR›	56990134	NA
27	oar3_OAR›	57174123	NA
27	oar3_OAR›	57184172	0.032582
27	oar3_OAR›	57271439	NA
27	oar3_OAR›	57291292	NA
27	oar3_OAR›	57368023	NA
27	oar3_OAR›	57400366	NA
27	oar3_OAR›	57406609	NA
27	oar3_OAR›	57408016	NA
27	oar3_OAR›	57497988	-0.01745
27	oar3_OAR›	57541069	0.000889
27	oar3_OAR›	57545334	0.000889
27	oar3_OAR›	57564181	0.000889
27	oar3_OAR›	57652689	0.007039
27	oar3_OAR›	57669657	0.159032
27	oar3_OAR›	57675314	0.159032
27	oar3_OAR›	57734678	0.000889
27	oar3_OAR›	57744384	0.000889
27	oar3_OAR›	57783944	0.000889
27	oar3_OAR›	57789997	0.000889

27	oar3_OAR›	57800444	0.000889
27	oar3_OAR›	57808129	0.000889
27	oar3_OAR›	57824137	0.000889
27	oar3_OAR›	57851434	0.159032
27	oar3_OAR›	57852707	0.012115
27	oar3_OAR›	57855058	0.159032
27	oar3_OAR›	57867417	0.159032
27	oar3_OAR›	57878646	0.000889
27	oar3_OAR›	57882770	0.000889
27	oar3_OAR›	57884479	0.000889
27	oar3_OAR›	57889084	0.159032
27	oar3_OAR›	57897996	0.000889
27	oar3_OAR›	57898183	0.159032
27	oar3_OAR›	57918674	0.000889
27	oar3_OAR›	57979905	0.190565
27	oar3_OAR›	57981745	0.00481
27	oar3_OAR›	57989810	0.059965
27	oar3_OAR›	58000213	0.036114
27	oar3_OAR›	58012995	0.191903
27	oar3_OAR›	58017052	0.124701
27	oar3_OAR›	58031420	0.000889
27	oar3_OAR›	58055444	-0.00127
27	oar3_OAR›	58056035	-0.00127
27	oar3_OAR›	58064493	########
27	oar3_OAR›	58065655	0.017332
27	oar3_OAR›	58065991	0.124701
27	oar3_OAR›	58066485	0.124701
27	oar3_OAR›	58075845	0.015777
27	oar3_OAR›	58091888	0.129378
27	oar3_OAR›	58098808	0.000889
27	oar3_OAR›	58099490	0.242208
27	oar3_OAR›	58111518	0.167906
27	oar3_OAR›	58112702	0.216872
27	oar3_OAR›	58115575	0.258914
27	oar3_OAR›	58116772	0.000889
27	oar3_OAR›	58116945	0.042554
27	oar3_OAR›	58124293	0.000889
27	oar3_OAR›	58128478	0.007009
27	oar3_OAR›	58129615	-0.01737
27	oar3_OAR›	58136324	0.181608
27	oar3_OAR›	58137013	0.226098
27	oar3_OAR›	58149646	0.167501
27	oar3_OAR›	58151638	0.067499
27	oar3_OAR›	58160209	0.084864
27	oar3_OAR›	58164296	0.084864
27	oar3_OAR›	58171607	0.14441
27	oar3_OAR›	58175284	-0.01964
27	oar3_OAR›	58177435	0.069893
27	oar3_OAR›	58182925	-0.00195
27	oar3_OAR›	58189232	0.173867
27	oar3_OAR›	58189783	0.224882
27	oar3_OAR›	58194420	0.000889
27	oar3_OAR›	58200045	0.146442
27	oar3_OAR›	58201259	0.111335

27	oar3_OAR}	58201788	0.111335
27	oar3_OAR}	58202482	0.111335
27	oar3_OAR}	58220571	0.144395
27	oar3_OAR}	58238876	0.108435
27	oar3_OAR}	58293840	-0.01891
27	oar3_OAR}	58294669	-0.01891
27	oar3_OAR}	58307401	-0.0309
27	oar3_OAR}	58312829	0.199315
27	oar3_OAR}	58320691	-0.01378
27	oar3_OAR}	58341381	-0.00209
27	oar3_OAR}	58341725	-0.02549
27	oar3_OAR}	58342228	-0.02549
27	oar3_OAR}	58346644	-0.02549
27	oar3_OAR}	58354420	0.010197
27	oar3_OAR}	58357840	-0.02678
27	oar3_OAR}	58363505	-0.02678
27	oar3_OAR}	58368422	8.73E-05
27	oar3_OAR}	58368779	0.01388
27	oar3_OAR}	58375684	-0.01673
27	oar3_OAR}	58391311	0.052764
27	oar3_OAR}	58406507	-0.01674
27	oar3_OAR}	58409413	-0.00814
27	oar3_OAR}	58410157	0.152607
27	oar3_OAR}	58414450	0.154972
27	oar3_OAR}	58416696	0.154972
27	oar3_OAR}	58421144	-0.01629
27	oar3_OAR}	58422483	0.16021
27	oar3_OAR}	58430493	0.155012
27	oar3_OAR}	58432264	0.170211
27	oar3_OAR}	58468568	-0.02624
27	oar3_OAR}	58469432	0.201451
27	oar3_OAR}	58472681	0.183439
27	oar3_OAR}	58474064	0.006173
27	oar3_OAR}	58485468	-0.01341
27	oar3_OAR}	58498141	2.97E-05
27	oar3_OAR}	58500039	2.97E-05
27	oar3_OAR}	58507138	2.97E-05
27	oar3_OAR}	58515685	0.037641
27	oar3_OAR}	58515746	0.037641
27	oar3_OAR}	58519101	0.108902
27	oar3_OAR}	58534136	0.280322
27	oar3_OAR}	58542714	0.043437
27	oar3_OAR}	58547769	0.011321
27	oar3_OAR}	58548335	0.043437
27	oar3_OAR}	58549768	0.037641
27	oar3_OAR}	58549981	0.037641
27	oar3_OAR}	58554091	0.056437
27	oar3_OAR}	58560543	0.056437
27	oar3_OAR}	58564163	0.280322
27	oar3_OAR}	58565694	0.056437
27	oar3_OAR}	58569054	0.011321
27	oar3_OAR}	58574066	-0.00032
27	oar3_OAR}	58579929	-0.01489
27	oar3_OAR}	58586791	0.295093

27	oar3_OAR›	58588222	0.012941
27	oar3_OAR›	58591562	0.157521
27	oar3_OAR›	58596419	0.198118
27	oar3_OAR›	58604868	0.360407
27	oar3_OAR›	58604977	0.021533
27	oar3_OAR›	58611409	0.198118
27	oar3_OAR›	58617657	0.012941
27	oar3_OAR›	58621221	0.371527
27	oar3_OAR›	58625574	-0.00308
27	oar3_OAR›	58627625	0.012941
27	oar3_OAR›	58631172	0.349158
27	oar3_OAR›	58631759	-0.02393
27	oar3_OAR›	58642295	0.009985
27	oar3_OAR›	58647543	0.340934
27	oar3_OAR›	58649679	0.289612
27	oar3_OAR›	58654161	-0.03248
27	oar3_OAR›	58657590	-0.02393
27	oar3_OAR›	58666382	0.215631
27	oar3_OAR›	58668421	0.215631
27	oar3_OAR›	58668519	0.215631
27	oar3_OAR›	58675782	0.294443
27	oar3_OAR›	58678583	0.215631
27	oar3_OAR›	58681638	0.003335
27	oar3_OAR›	58683335	-0.00276
27	oar3_OAR›	58687373	0.197226
27	oar3_OAR›	58688911	0.014419
27	oar3_OAR›	58695536	-0.00935
27	oar3_OAR›	58699321	0.017279
27	oar3_OAR›	58711218	0.213537
27	oar3_OAR›	58711334	0.067833
27	oar3_OAR›	58714676	0.115049
27	oar3_OAR›	58722957	0.202693
27	oar3_OAR›	58723759	0.012231
27	oar3_OAR›	58734703	-0.03522
27	oar3_OAR›	58750129	-0.01445
27	oar3_OAR›	58751925	-0.03699
27	oar3_OAR›	58757114	-0.0143
27	oar3_OAR›	58762246	-0.02697
27	oar3_OAR›	58763576	0.002166
27	oar3_OAR›	58770859	0.002702
27	oar3_OAR›	58784063	-0.0122
27	oar3_OAR›	58786788	-0.03573
27	oar3_OAR›	58790654	-0.0058
27	oar3_OAR›	58792696	-0.02159
27	oar3_OAR›	58799642	0.103097
27	oar3_OAR›	58803204	-0.01091
27	oar3_OAR›	58805197	-0.01165
27	oar3_OAR›	58806022	-0.02679
27	oar3_OAR›	58807606	-0.0199
27	oar3_OAR›	58821194	-0.01211
27	oar3_OAR›	58823003	0.009739
27	oar3_OAR›	58823628	-0.01991
27	oar3_OAR›	58836596	-0.02291
27	oar3_OAR›	58837654	0.019414

27	oar3_OAR}	58844435	0.041601
27	oar3_OAR}	58855981	-0.00606
27	oar3_OAR}	58856143	0.050917
27	oar3_OAR}	58856219	-0.0133
27	oar3_OAR}	58868148	0.060033
27	oar3_OAR}	58881951	0.033773
27	oar3_OAR}	58889452	0.00811
27	oar3_OAR}	58916894	0.035628
27	oar3_OAR}	58918641	0.023537
27	oar3_OAR}	58934504	0.071488
27	oar3_OAR}	58935397	0.007219
27	oar3_OAR}	58948092	0.071488
27	oar3_OAR}	58948156	0.01388
27	oar3_OAR}	58949836	-0.00946
27	oar3_OAR}	58952106	0.071488
27	oar3_OAR}	58954475	-0.03375
27	oar3_OAR}	58974586	-0.03375
27	oar3_OAR}	58974646	NA
27	oar3_OAR}	58976044	0.042893
27	oar3_OAR}	58984666	-0.01809
27	oar3_OAR}	58984762	0.059294
27	oar3_OAR}	58994677	0.007726
27	oar3_OAR}	59009769	0.125244
27	oar3_OAR}	59018126	-0.02926
27	oar3_OAR}	59021145	0.023929
27	oar3_OAR}	59026179	-0.01274
27	oar3_OAR}	59026565	0.055489
27	oar3_OAR}	59034719	-0.00863
27	oar3_OAR}	59037382	-0.00863
27	oar3_OAR}	59039527	-0.00863
27	oar3_OAR}	59042616	0.200403
27	oar3_OAR}	59045948	0.126113
27	oar3_OAR}	59054378	0.126113
27	oar3_OAR}	59055339	0.126113
27	oar3_OAR}	59055572	0.061637
27	oar3_OAR}	59063743	-0.01887
27	oar3_OAR}	59064484	0.13584
27	oar3_OAR}	59068930	0.051662
27	oar3_OAR}	59069902	0.008808
27	oar3_OAR}	59075913	0.13584
27	oar3_OAR}	59084642	0.066488
27	oar3_OAR}	59085887	0.066488
27	oar3_OAR}	59103930	0.13584
27	oar3_OAR}	59128270	0.056503
27	oar3_OAR}	59131027	0.056515
27	oar3_OAR}	59132299	-0.02851
27	oar3_OAR}	59138455	0.065711
27	oar3_OAR}	59143126	0.214174
27	oar3_OAR}	59145531	0.029019
27	oar3_OAR}	59153092	0.129321
27	oar3_OAR}	59174015	-0.02359
27	oar3_OAR}	59185327	-0.01749
27	oar3_OAR}	59186124	-0.02731
27	oar3_OAR}	59188597	0.042574

27	oar3_OAR›	59197009	0.015023
27	oar3_OAR›	59197063	-0.00528
27	oar3_OAR›	59200215	0.051356
27	oar3_OAR›	59203567	-0.0146
27	oar3_OAR›	59214387	0.020654
27	oar3_OAR›	59227496	-0.01338
27	oar3_OAR›	59227809	0.20077
27	oar3_OAR›	59232806	-0.01338
27	oar3_OAR›	59234630	0.118013
27	oar3_OAR›	59245108	-0.00871
27	oar3_OAR›	59245237	-0.00871
27	oar3_OAR›	59249626	0.069912
27	oar3_OAR›	59268083	-0.00275
27	oar3_OAR›	59276642	-0.00295
27	oar3_OAR›	59277006	-0.00295
27	oar3_OAR›	59277325	0.03745
27	oar3_OAR›	59278223	0.043209
27	oar3_OAR›	59282634	0.03745
27	oar3_OAR›	59287913	0.113517
27	oar3_OAR›	59289859	0.19171
27	oar3_OAR›	59290533	0.043209
27	oar3_OAR›	59305152	0.043209
27	oar3_OAR›	59305622	-0.02767
27	oar3_OAR›	59310770	0.158239
27	oar3_OAR›	59316074	-0.00969
27	oar3_OAR›	59316182	0.03073
27	oar3_OAR›	59316415	-0.00969
27	oar3_OAR›	59323923	0.017271
27	oar3_OAR›	59324458	-0.00815
27	oar3_OAR›	59339661	-0.0054
27	oar3_OAR›	59339771	-0.02492
27	oar3_OAR›	59343373	-0.02076
27	oar3_OAR›	59347450	0.0139
27	oar3_OAR›	59349911	0.019396
27	oar3_OAR›	59356799	0.035666
27	oar3_OAR›	59367302	0.161217
27	oar3_OAR›	59376915	0.092086
27	oar3_OAR›	59382840	0.021346
27	oar3_OAR›	59388206	0.106158
27	oar3_OAR›	59393206	0.018645
27	oar3_OAR›	59397364	0.103182
27	oar3_OAR›	59399197	-0.00068
27	oar3_OAR›	59399589	0.123413
27	oar3_OAR›	59405703	0.02715
27	oar3_OAR›	59411368	0.103182
27	oar3_OAR›	59412571	-0.0236
27	oar3_OAR›	59413366	-0.03029
27	oar3_OAR›	59419456	0.011203
27	oar3_OAR›	59429058	0.021415
27	oar3_OAR›	59441674	-0.0229
27	oar3_OAR›	59444625	-0.0229
27	oar3_OAR›	59447588	0.135158
27	oar3_OAR›	59462240	0.027152
27	oar3_OAR›	59462723	-0.0191

27	oar3_OAR›	59463407	0.151066
27	oar3_OAR›	59475428	0.014434
27	oar3_OAR›	59490361	0.01105
27	oar3_OAR›	59490739	0.066207
27	oar3_OAR›	59497528	0.007188
27	oar3_OAR›	59502913	0.050393
27	oar3_OAR›	59503374	-0.03332
27	oar3_OAR›	59508843	-0.03332
27	oar3_OAR›	59515187	-0.03332
27	oar3_OAR›	59518826	-0.00083
27	oar3_OAR›	59519753	0.004928
27	oar3_OAR›	59527417	0.007188
27	oar3_OAR›	59544419	0.031025
27	oar3_OAR›	59546908	-0.01194
27	oar3_OAR›	59547555	0.096997
27	oar3_OAR›	59550322	0.203127
27	oar3_OAR›	59555217	-0.0181
27	oar3_OAR›	59555228	0.075892
27	oar3_OAR›	59561082	-0.01518
27	oar3_OAR›	59561231	-0.01913
27	oar3_OAR›	59566188	0.083703
27	oar3_OAR›	59567262	-0.01932
27	oar3_OAR›	59571115	-0.01932
27	oar3_OAR›	59586608	0.035007
27	oar3_OAR›	59587384	0.003086
27	oar3_OAR›	59589130	-0.02899
27	oar3_OAR›	59591955	-0.02899
27	oar3_OAR›	59597467	-0.02899
27	oar3_OAR›	59634212	0.039668
27	oar3_OAR›	59635061	-0.02847
27	oar3_OAR›	59636161	0.093901
27	oar3_OAR›	59643801	-0.01309
27	oar3_OAR›	59647583	0.028113
27	oar3_OAR›	59648612	-0.03047
27	oar3_OAR›	59649605	0.042868
27	oar3_OAR›	59656573	0.036779
27	oar3_OAR›	59656814	0.036779
27	oar3_OAR›	59656932	0.036779
27	oar3_OAR›	59658969	-0.03483
27	oar3_OAR›	59662706	0.011203
27	oar3_OAR›	59683671	-0.03157
27	oar3_OAR›	59689961	-0.02799
27	oar3_OAR›	59690768	0.046012
27	oar3_OAR›	59691907	0.005755
27	oar3_OAR›	59695972	0.023611
27	oar3_OAR›	59700221	0.005755
27	oar3_OAR›	59713060	0.005608
27	oar3_OAR›	59716491	-0.00934
27	oar3_OAR›	59716719	0.127467
27	oar3_OAR›	59717239	0.095731
27	oar3_OAR›	59718011	0.095731
27	oar3_OAR›	59720512	0.095731
27	oar3_OAR›	59727676	0.057694
27	oar3_OAR›	59728413	0.095731

27	oar3_OAR›	59732344	0.00686
27	oar3_OAR›	59734726	-0.00455
27	oar3_OAR›	59736642	0.032976
27	oar3_OAR›	59739840	0.182445
27	oar3_OAR›	59739919	0.182445
27	oar3_OAR›	59748969	0.095731
27	oar3_OAR›	59753456	0.057694
27	oar3_OAR›	59754927	0.127467
27	oar3_OAR›	59760961	-0.00414
27	oar3_OAR›	59781152	0.093575
27	oar3_OAR›	59782030	0.013212
27	oar3_OAR›	59789393	-0.00414
27	oar3_OAR›	59790029	0.093575
27	oar3_OAR›	59790726	0.044131
27	oar3_OAR›	59796428	0.044416
27	oar3_OAR›	59801858	0.010879
27	oar3_OAR›	59808606	0.013212
27	oar3_OAR›	59827857	0.013212
27	oar3_OAR›	59829960	0.10636
27	oar3_OAR›	59850441	0.02501
27	oar3_OAR›	59863164	-0.02636
27	oar3_OAR›	59867129	0.013212
27	oar3_OAR›	59868347	-0.00961
27	oar3_OAR›	59879484	0.03474
27	oar3_OAR›	59892198	0.049765
27	oar3_OAR›	59893411	0.034787
27	oar3_OAR›	59895603	0.015702
27	oar3_OAR›	59904910	0.01715
27	oar3_OAR›	59905634	0.01715
27	oar3_OAR›	59910701	0.020777
27	oar3_OAR›	59913710	0.021533
27	oar3_OAR›	59923557	0.062295
27	oar3_OAR›	59931749	0.272541
27	oar3_OAR›	59934420	0.087717
27	oar3_OAR›	59935085	-0.02292
27	oar3_OAR›	59943049	-0.01529
27	oar3_OAR›	59943867	-0.00349
27	oar3_OAR›	59948993	0.041446
27	oar3_OAR›	59949745	0.057581
27	oar3_OAR›	59958908	0.021901
27	oar3_OAR›	59958995	0.036019
27	oar3_OAR›	59964964	-0.00349
27	oar3_OAR›	59967989	0.005276
27	oar3_OAR›	59973603	0.016284
27	oar3_OAR›	59978981	0.016284
27	oar3_OAR›	59980466	-0.0092
27	oar3_OAR›	60001647	0.011067
27	oar3_OAR›	60031530	NA
27	oar3_OAR›	60033149	-0.03776
27	oar3_OAR›	60033732	-0.03776
27	oar3_OAR›	60043630	0.333662
27	oar3_OAR›	60047995	0.333662
27	oar3_OAR›	60059879	0.250432
27	oar3_OAR›	60060366	0.333662

27	oar3_OAR›	60060666	0.333662
27	oar3_OAR›	60068965	0.298885
27	oar3_OAR›	60072437	0.179234
27	oar3_OAR›	60073919	0.012749
27	oar3_OAR›	60083154	0.207888
27	oar3_OAR›	60083251	0.2194
27	oar3_OAR›	60094366	0.179234
27	oar3_OAR›	60094746	0.179234
27	oar3_OAR›	60095153	0.298885
27	oar3_OAR›	60101270	0.179234
27	oar3_OAR›	60125250	0.2194
27	oar3_OAR›	60147662	0.03329
27	oar3_OAR›	60161541	0.022582
27	oar3_OAR›	60164009	0.007009
27	oar3_OAR›	60182450	0.047855
27	oar3_OAR›	60184486	0.009746
27	oar3_OAR›	60203905	-0.01665
27	oar3_OAR›	60204415	-0.01665
27	oar3_OAR›	60212115	-0.02645
27	oar3_OAR›	60215101	-0.01665
27	oar3_OAR›	60220773	0.380098
27	oar3_OAR›	60225816	0.349227
27	oar3_OAR›	60227084	NA
27	oar3_OAR›	60238749	0.392437
27	oar3_OAR›	60239506	0.448572
27	oar3_OAR›	60245496	0.392437
27	oar3_OAR›	60278646	0.388235
27	oar3_OAR›	60285002	0.398996
27	oar3_OAR›	60286002	0.341993
27	oar3_OAR›	60305507	0.436167
27	oar3_OAR›	60306000	0.358366
27	oar3_OAR›	60310548	-0.02562
27	oar3_OAR›	60313745	-0.00308
27	oar3_OAR›	60341954	0.358366
27	oar3_OAR›	60348257	-0.0284
27	oar3_OAR›	60370250	0.207566
27	oar3_OAR›	60370846	0.416232
27	oar3_OAR›	60376057	0.416232
27	oar3_OAR›	60385562	0.416232
27	oar3_OAR›	60391066	0.465503
27	oar3_OAR›	60396632	0.465503
27	oar3_OAR›	60397696	0.465503
27	oar3_OAR›	60412186	0.000889
27	oar3_OAR›	60425938	0.561878
27	oar3_OAR›	60432418	0.50327
27	oar3_OAR›	60454181	0.463765
27	oar3_OAR›	60466351	0.065275
27	oar3_OAR›	60478328	0.070004
27	oar3_OAR›	60506013	0.04998
27	oar3_OAR›	60516217	-0.00091
27	oar3_OAR›	60532112	0.204583
27	oar3_OAR›	60534067	0.075737
27	oar3_OAR›	60537334	0.204583
27	oar3_OAR›	60554716	0.059087

27	oar3_OAR›	60563543	0.17039
27	oar3_OAR›	60566703	0.17039
27	oar3_OAR›	60570035	0.17039
27	oar3_OAR›	60575848	0.17039
27	oar3_OAR›	60578536	0.007039
27	oar3_OAR›	60586397	0.01591
27	oar3_OAR›	60613332	0.051958
27	oar3_OAR›	60613398	0.074084
27	oar3_OAR›	60613560	0.051958
27	oar3_OAR›	60632399	0.003214
27	oar3_OAR›	60632795	0.003214
27	oar3_OAR›	60632952	0.139658
27	oar3_OAR›	60641377	-0.02325
27	oar3_OAR›	60654817	0.13058
27	oar3_OAR›	60657482	-0.0047
27	oar3_OAR›	60666760	0.13058
27	oar3_OAR›	60667660	0.14011
27	oar3_OAR›	60672640	0.168648
27	oar3_OAR›	60672908	0.089075
27	oar3_OAR›	60677989	0.273831
27	oar3_OAR›	60682186	0.372819
27	oar3_OAR›	60688445	0.27953
27	oar3_OAR›	60694209	0.27953
27	oar3_OAR›	60709378	0.311093
27	oar3_OAR›	60709819	0.22115
27	oar3_OAR›	60712388	0.188533
27	oar3_OAR›	60726422	0.292977
27	oar3_OAR›	60739924	-0.02381
27	oar3_OAR›	60769014	########
27	oar3_OAR›	60777496	0.007871
27	oar3_OAR›	60779609	0.00215
27	oar3_OAR›	60784348	-0.02398
27	oar3_OAR›	60784769	0.022164
27	oar3_OAR›	60789343	0.046835
27	oar3_OAR›	60797737	0.055003
27	oar3_OAR›	60799505	0.048438
27	oar3_OAR›	60815044	0.002668
27	oar3_OAR›	60817175	0.0275
27	oar3_OAR›	60821915	0.019123
27	oar3_OAR›	60832257	0.038304
27	oar3_OAR›	60835388	0.009617
27	oar3_OAR›	60837014	0.038304
27	oar3_OAR›	60841450	0.038304
27	oar3_OAR›	60846855	-0.00097
27	oar3_OAR›	60847356	0.03574
27	oar3_OAR›	60847360	0.051801
27	oar3_OAR›	60851502	0.115437
27	oar3_OAR›	60854232	-0.00524
27	oar3_OAR›	60864783	0.013631
27	oar3_OAR›	60870384	0.061242
27	oar3_OAR›	60872192	0.013357
27	oar3_OAR›	60875591	0.061242
27	oar3_OAR›	60879808	0.021081
27	oar3_OAR›	60880300	0.141752

27	oar3_OAR›	60881034	0.021081
27	oar3_OAR›	60889726	-0.0323
27	oar3_OAR›	60892320	-0.0323
27	oar3_OAR›	60909836	0.075873
27	oar3_OAR›	60916435	0.03648
27	oar3_OAR›	60964149	0.087014
27	oar3_OAR›	61014606	-0.00826
27	oar3_OAR›	61032894	0.135002
27	oar3_OAR›	61041379	0.061153
27	oar3_OAR›	61045174	0.14318
27	oar3_OAR›	61048454	0.111569
27	oar3_OAR›	61048752	0.111569
27	oar3_OAR›	61055798	0.035869
27	oar3_OAR›	61063602	-0.01818
27	oar3_OAR›	61066428	0.049509
27	oar3_OAR›	61071871	-0.01242
27	oar3_OAR›	61081425	-0.00205
27	oar3_OAR›	61082210	-0.01131
27	oar3_OAR›	61084395	-0.02002
27	oar3_OAR›	61090389	-0.03293
27	oar3_OAR›	61094825	-0.0194
27	oar3_OAR›	61102856	-0.01162
27	oar3_OAR›	61106779	-0.02827
27	oar3_OAR›	61107155	-0.02345
27	oar3_OAR›	61134682	0.097904
27	oar3_OAR›	61143075	0.069806
27	oar3_OAR›	61144474	0.062909
27	oar3_OAR›	61149471	0.086984
27	oar3_OAR›	61150026	0.140896
27	oar3_OAR›	61151913	0.015085
27	oar3_OAR›	61152418	0.086984
27	oar3_OAR›	61160827	0.086984
27	oar3_OAR›	61163877	0.109006
27	oar3_OAR›	61174380	0.043085
27	oar3_OAR›	61175801	-0.01496
27	oar3_OAR›	61190175	0.014522
27	oar3_OAR›	61190424	0.014522
27	oar3_OAR›	61190647	0.014522
27	oar3_OAR›	61191935	-0.04214
27	oar3_OAR›	61195603	0.190469
27	oar3_OAR›	61203395	-0.03258
27	oar3_OAR›	61214117	0.146144
27	oar3_OAR›	61214977	0.046525
27	oar3_OAR›	61215109	-0.02489
27	oar3_OAR›	61220783	-0.02016
27	oar3_OAR›	61221310	0.202722
27	oar3_OAR›	61229689	-0.02489
27	oar3_OAR›	61237542	-0.00583
27	oar3_OAR›	61237670	0.008643
27	oar3_OAR›	61247347	-0.02916
27	oar3_OAR›	61249326	-0.01426
27	oar3_OAR›	61259489	-0.01426
27	oar3_OAR›	61260837	0.178469
27	oar3_OAR›	61263371	0.087185

27	oar3_OAR›	61266543	0.0062
27	oar3_OAR›	61272864	-0.02916
27	oar3_OAR›	61272973	-0.02916
27	oar3_OAR›	61273920	-0.02916
27	oar3_OAR›	61284644	0.011798
27	oar3_OAR›	61286359	-0.0164
27	oar3_OAR›	61292715	-0.0164
27	oar3_OAR›	61295469	-0.01321
27	oar3_OAR›	61300466	0.043451
27	oar3_OAR›	61302926	0.043566
27	oar3_OAR›	61308842	0.034069
27	oar3_OAR›	61311246	-0.01772
27	oar3_OAR›	61315021	0.050161
27	oar3_OAR›	61319034	0.057936
27	oar3_OAR›	61319716	-0.00606
27	oar3_OAR›	61331760	-0.01321
27	oar3_OAR›	61337382	0.016306
27	oar3_OAR›	61337414	0.016306
27	oar3_OAR›	61337867	0.035734
27	oar3_OAR›	61346995	0.001889
27	oar3_OAR›	61347854	0.016306
27	oar3_OAR›	61351525	-0.01717
27	oar3_OAR›	61358408	0.025934
27	oar3_OAR›	61366026	0.012486
27	oar3_OAR›	61388346	0.014112
27	oar3_OAR›	61388469	0.003446
27	oar3_OAR›	61390765	0.072054
27	oar3_OAR›	61394968	0.003446
27	oar3_OAR›	61405988	0.06533
27	oar3_OAR›	61407349	-0.00776
27	oar3_OAR›	61407486	0.00986
27	oar3_OAR›	61407709	0.003446
27	oar3_OAR›	61421209	0.002668
27	oar3_OAR›	61430277	0.000363
27	oar3_OAR›	61435886	0.035734
27	oar3_OAR›	61436509	0.044363
27	oar3_OAR›	61446335	0.01726
27	oar3_OAR›	61458041	-0.01822
27	oar3_OAR›	61459901	0.011365
27	oar3_OAR›	61475825	-0.00788
27	oar3_OAR›	61476161	0.057126
27	oar3_OAR›	61481427	-0.03745
27	oar3_OAR›	61488946	0.071863
27	oar3_OAR›	61499375	-0.01117
27	oar3_OAR›	61509474	0.193534
27	oar3_OAR›	61509521	0.129678
27	oar3_OAR›	61515849	-0.00255
27	oar3_OAR›	61525782	0.076187
27	oar3_OAR›	61525834	0.041098
27	oar3_OAR›	61525840	0.178
27	oar3_OAR›	61527299	0.078041
27	oar3_OAR›	61536440	0.048281
27	oar3_OAR›	61550761	0.091767
27	oar3_OAR›	61565993	-0.03738

27	oar3_OAR›	61569548	0.076842
27	oar3_OAR›	61576060	0.091243
27	oar3_OAR›	61578363	0.020399
27	oar3_OAR›	61578670	-0.03627
27	oar3_OAR›	61580137	-0.03531
27	oar3_OAR›	61590145	-0.03531
27	oar3_OAR›	61601613	0.024436
27	oar3_OAR›	61602368	0.040241
27	oar3_OAR›	61604986	-0.02647
27	oar3_OAR›	61614206	0.051674
27	oar3_OAR›	61618633	-0.01851
27	oar3_OAR›	61619312	-0.01851
27	oar3_OAR›	61638564	0.000889
27	oar3_OAR›	61638974	0.076774
27	oar3_OAR›	61641076	0.003046
27	oar3_OAR›	61660455	0.112076
27	oar3_OAR›	61662455	-0.02279
27	oar3_OAR›	61666317	0.124706
27	oar3_OAR›	61671974	0.138706
27	oar3_OAR›	61688684	-0.01599
27	oar3_OAR›	61689386	0.138706
27	oar3_OAR›	61694134	0.138706
27	oar3_OAR›	61700943	0.048897
27	oar3_OAR›	61701723	0.138706
27	oar3_OAR›	61706543	-0.02782
27	oar3_OAR›	61706672	0.1461
27	oar3_OAR›	61707088	-0.02179
27	oar3_OAR›	61720546	0.135049
27	oar3_OAR›	61735480	0.028743
27	oar3_OAR›	61751715	0.135049
27	oar3_OAR›	61752695	0.000889
27	oar3_OAR›	61753845	0.183064
27	oar3_OAR›	61754239	0.191016
27	oar3_OAR›	61765175	-0.02179
27	oar3_OAR›	61767219	-0.02077
27	oar3_OAR›	61776655	0.037529
27	oar3_OAR›	61777456	0.081573
27	oar3_OAR›	61783064	-0.01969
27	oar3_OAR›	61783623	-0.01969
27	oar3_OAR›	61787555	-0.00606
27	oar3_OAR›	61794160	-0.00681
27	oar3_OAR›	61795973	-0.00606
27	oar3_OAR›	61799422	0.026055
27	oar3_OAR›	61804877	0.058103
27	oar3_OAR›	61804937	-0.027
27	oar3_OAR›	61806032	0.058103
27	oar3_OAR›	61813341	0.058103
27	oar3_OAR›	61813395	-0.027
27	oar3_OAR›	61813659	0.069912
27	oar3_OAR›	61817471	0.046857
27	oar3_OAR›	61843060	0.207778
27	oar3_OAR›	61845090	0.224915
27	oar3_OAR›	61846136	0.238495
27	oar3_OAR›	61850224	0.224915

27	oar3_OAR>	61856777	0.125097
27	oar3_OAR>	61856837	0.018804
27	oar3_OAR>	61857883	0.148605
27	oar3_OAR>	61870093	0.224915
27	oar3_OAR>	61870165	0.224915
27	oar3_OAR>	61884770	0.021274
27	oar3_OAR>	61886716	0.207778
27	oar3_OAR>	61890354	0.224915
27	oar3_OAR>	61910113	-0.01069
27	oar3_OAR>	61920919	0.001634
27	oar3_OAR>	61936482	-0.01145
27	oar3_OAR>	61941421	0.187846
27	oar3_OAR>	61947055	-0.02041
27	oar3_OAR>	61948391	-0.01252
27	oar3_OAR>	61957491	0.001585
27	oar3_OAR>	61958959	-0.00188
27	oar3_OAR>	61959002	-0.00521
27	oar3_OAR>	61962443	0.149755
27	oar3_OAR>	61972898	0.113056
27	oar3_OAR>	61978059	-0.00521
27	oar3_OAR>	61982898	-0.02119
27	oar3_OAR>	61986159	-0.00521
27	oar3_OAR>	61987132	0.122867
27	oar3_OAR>	61997589	0.164638
27	oar3_OAR>	62006573	0.014466
27	oar3_OAR>	62007052	0.014466
27	oar3_OAR>	62014523	0.192665
27	oar3_OAR>	62044201	0.033554
27	oar3_OAR>	62053912	0.107252
27	oar3_OAR>	62079419	0.130458
27	oar3_OAR>	62087333	0.014466
27	oar3_OAR>	62099079	0.072109
27	oar3_OAR>	62115801	0.164638
27	oar3_OAR>	62115990	0.164638
27	oar3_OAR>	62162891	0.130458
27	oar3_OAR>	62162991	-0.01574
27	oar3_OAR>	62195930	0.057497
27	oar3_OAR>	62196194	0.014402
27	oar3_OAR>	62251011	0.014402
27	oar3_OAR>	62257668	0.014402
27	oar3_OAR>	62284381	0.000889
27	oar3_OAR>	62290508	NA
27	oar3_OAR>	62323248	0.00161
27	oar3_OAR>	62376997	0.031474
27	oar3_OAR>	62389436	0.031474
27	oar3_OAR>	62389687	0.031474
27	oar3_OAR>	62406040	0.007039
27	oar3_OAR>	62413067	-0.01457
27	oar3_OAR>	62429795	-0.02516
27	oar3_OAR>	62431681	-0.01214
27	oar3_OAR>	62490469	0.088482
27	oar3_OAR>	62491718	0.038483
27	oar3_OAR>	62493897	NA
27	oar3_OAR>	62494552	-0.00287

27	oar3_OAR›	62573175	-0.00322
27	oar3_OAR›	62573445	-0.00322
27	oar3_OAR›	62579820	-0.00322
27	oar3_OAR›	62584680	-0.00322
27	oar3_OAR›	62586083	0.038483
27	oar3_OAR›	62592720	0.035084
27	oar3_OAR›	62596229	0.052636
27	oar3_OAR›	62598456	NA
27	oar3_OAR›	62610121	NA
27	oar3_OAR›	62610787	0.120111
27	oar3_OAR›	62618426	0.170051
27	oar3_OAR›	62621379	NA
27	oar3_OAR›	62622694	NA
27	oar3_OAR›	62630133	0.105123
27	oar3_OAR›	62634323	-0.02645
27	oar3_OAR›	62639315	0.120111
27	oar3_OAR›	62639839	-0.02645
27	oar3_OAR›	62710112	0.291926
27	oar3_OAR›	62746503	0.021081
27	oar3_OAR›	62827581	0.265464
27	oar3_OAR›	62863334	0.021081
27	oar3_OAR›	62869410	0.021081
27	oar3_OAR›	62880692	0.081472
27	oar3_OAR›	62882194	0.021081
27	oar3_OAR›	62888266	0.069843
27	oar3_OAR›	62890215	-0.01891
27	oar3_OAR›	62898204	0.091414
27	oar3_OAR›	62901072	0.091414
27	oar3_OAR›	62924733	0.094664
27	oar3_OAR›	62933059	0.094664
27	oar3_OAR›	62953678	0.041833
27	oar3_OAR›	62966811	0.296531
27	oar3_OAR›	62967208	0.046341
27	oar3_OAR›	62970636	-0.0281
27	oar3_OAR›	62981967	0.000631
27	oar3_OAR›	62984458	0.032696
27	oar3_OAR›	63002338	0.101938
27	oar3_OAR›	63002879	0.000631
27	oar3_OAR›	63026112	0.018601
27	oar3_OAR›	63072134	0.118705
27	oar3_OAR›	63072210	0.120022
27	oar3_OAR›	63083304	0.120022
27	oar3_OAR›	63084718	0.064242
27	oar3_OAR›	63089205	0.077761
27	oar3_OAR›	63095177	0.077761
27	oar3_OAR›	63100364	0.018601
27	oar3_OAR›	63100510	0.05918
27	oar3_OAR›	63104912	0.023589
27	oar3_OAR›	63106340	0.116367
27	oar3_OAR›	63109989	0.013467
27	oar3_OAR›	63124525	0.013467
27	oar3_OAR›	63129134	0.013467
27	oar3_OAR›	63130665	-0.00521
27	oar3_OAR›	63136912	0.013467

27	oar3_OAR›	63139945	0.013467
27	oar3_OAR›	63143139	0.005595
27	oar3_OAR›	63147317	0.005595
27	oar3_OAR›	63178444	0.101357
27	oar3_OAR›	63178541	0.101357
27	oar3_OAR›	63180169	-0.00114
27	oar3_OAR›	63181961	0.017015
27	oar3_OAR›	63191679	0.340384
27	oar3_OAR›	63192077	0.377543
27	oar3_OAR›	63208996	0.166262
27	oar3_OAR›	63211937	0.340384
27	oar3_OAR›	63214200	0.377543
27	oar3_OAR›	63215193	-0.03648
27	oar3_OAR›	63228759	0.377543
27	oar3_OAR›	63229291	0.340384
27	oar3_OAR›	63235315	0.377543
27	oar3_OAR›	63235876	0.140978
27	oar3_OAR›	63250521	0.140978
27	oar3_OAR›	63251969	0.025629
27	oar3_OAR›	63253038	0.140978
27	oar3_OAR›	63263562	0.009972
27	oar3_OAR›	63265855	0.182878
27	oar3_OAR›	63268403	0.013414
27	oar3_OAR›	63268700	0.196221
27	oar3_OAR›	63278256	0.187058
27	oar3_OAR›	63281709	0.181571
27	oar3_OAR›	63326874	0.193809
27	oar3_OAR›	63357650	0.347806
27	oar3_OAR›	63368625	0.004461
27	oar3_OAR›	63412985	0.23446
27	oar3_OAR›	63427685	0.19135
27	oar3_OAR›	63477292	0.159096
27	oar3_OAR›	63523886	0.000363
27	oar3_OAR›	63538737	0.105123
27	oar3_OAR›	63545468	0.000363
27	oar3_OAR›	63551637	0.220795
27	oar3_OAR›	63566182	0.0891
27	oar3_OAR›	63570790	0.196909
27	oar3_OAR›	63593877	0.196909
27	oar3_OAR›	63601729	0.196909
27	oar3_OAR›	63620053	0.196909
27	oar3_OAR›	63630475	0.238472
27	oar3_OAR›	63652730	0.36934
27	oar3_OAR›	63662734	0.36934
27	oar3_OAR›	63680508	0.33103
27	oar3_OAR›	63681527	0.044943
27	oar3_OAR›	63681687	0.159875
27	oar3_OAR›	63682534	0.151993
27	oar3_OAR›	63690478	0.331352
27	oar3_OAR›	63696366	0.33103
27	oar3_OAR›	63701948	0.157425
27	oar3_OAR›	63720197	0.044943
27	oar3_OAR›	63738659	0.36934
27	oar3_OAR›	63757460	0.404429

27	oar3_OAR\	63758547	0.201193
27	oar3_OAR\	63861084	0.044943
27	oar3_OAR\	63919055	0.003497
27	oar3_OAR\	63925247	0.127069
27	oar3_OAR\	63963493	0.127613
27	oar3_OAR\	63965592	0.127613
27	oar3_OAR\	63973451	0.081704
27	oar3_OAR\	64082428	0.146906
27	oar3_OAR\	64085354	0.146906
27	oar3_OAR\	64096354	0.146906
27	oar3_OAR\	64210878	0.089143
27	oar3_OAR\	64213255	0.129463
27	oar3_OAR\	64226346	0.089143
27	oar3_OAR\	64228852	0.098397
27	oar3_OAR\	64230271	0.098397
27	oar3_OAR\	64243996	0.098397
27	oar3_OAR\	64273844	0.129463
27	oar3_OAR\	64289062	0.125191
27	oar3_OAR\	64292701	0.125191
27	oar3_OAR\	64300244	0.129463
27	oar3_OAR\	64308939	0.091813
27	oar3_OAR\	64309232	0.091813
27	oar3_OAR\	64320788	0.091813
27	oar3_OAR\	64321003	0.091813
27	oar3_OAR\	64323647	0.081704
27	oar3_OAR\	64339808	0.114595
27	oar3_OAR\	64355968	0.009639
27	oar3_OAR\	64381208	0.149792
27	oar3_OAR\	64396059	0.248174
27	oar3_OAR\	64396639	0.096034
27	oar3_OAR\	64427941	0.248174
27	oar3_OAR\	64444601	0.216605
27	oar3_OAR\	64445352	0.072812
27	oar3_OAR\	64445422	0.081148
27	oar3_OAR\	64464617	0.064332
27	oar3_OAR\	64465454	0.216605
27	oar3_OAR\	64477644	0.216605
27	oar3_OAR\	64489939	0.043647
27	oar3_OAR\	64495496	-0.00882
27	oar3_OAR\	64517757	0.043647
27	oar3_OAR\	64559813	-0.01643
27	oar3_OAR\	64597133	0.038594
27	oar3_OAR\	64626469	0.123611
27	oar3_OAR\	64632129	0.315855
27	oar3_OAR\	64639146	0.205108
27	oar3_OAR\	64647249	0.311266
27	oar3_OAR\	64657326	0.142981
27	oar3_OAR\	64658845	0.112524
27	oar3_OAR\	64659019	0.311266
27	oar3_OAR\	64662792	0.142981
27	oar3_OAR\	64694459	0.142981
27	oar3_OAR\	64697033	0.123611
27	oar3_OAR\	64716449	0.123611
27	oar3_OAR\	64718687	0.142981

27	oar3_OAR›	64764612	0.123611
27	oar3_OAR›	64786751	0.123611
27	oar3_OAR›	64787504	0.123611
27	oar3_OAR›	64799341	0.123611
27	oar3_OAR›	64801999	0.123611
27	oar3_OAR›	64805359	0.123611
27	oar3_OAR›	64812700	0.336169
27	oar3_OAR›	64814906	0.123611
27	oar3_OAR›	64843922	0.123611
27	oar3_OAR›	64885536	0.123611
27	oar3_OAR›	64938081	0.123611
27	oar3_OAR›	64948743	0.142981
27	oar3_OAR›	64954237	0.336169
27	oar3_OAR›	64957734	NA
27	oar3_OAR›	64986093	0.142981
27	oar3_OAR›	64988874	0.142981
27	oar3_OAR›	64998451	0.142981
27	oar3_OAR›	65000226	0.142981
27	oar3_OAR›	65013171	0.336169
27	oar3_OAR›	65024907	0.004461
27	oar3_OAR›	65035210	0.050577
27	oar3_OAR›	65059071	0.251447
27	oar3_OAR›	65061172	0.212276
27	oar3_OAR›	65069397	0.227851
27	oar3_OAR›	65069939	0.227851
27	oar3_OAR›	65088045	0.227851
27	oar3_OAR›	65088135	0.227851
27	oar3_OAR›	65089308	0.227851
27	oar3_OAR›	65093724	0.015728
27	oar3_OAR›	65107031	0.015728
27	oar3_OAR›	65107606	0.015728
27	oar3_OAR›	65107872	0.274207
27	oar3_OAR›	65122243	0.274207
27	oar3_OAR›	65123478	0.274207
27	oar3_OAR›	65131674	0.274207
27	oar3_OAR›	65139451	0.274207
27	oar3_OAR›	65162844	0.247734
27	oar3_OAR›	65171285	0.032059
27	oar3_OAR›	65173048	0.247734
27	oar3_OAR›	65181703	0.244274
27	oar3_OAR›	65225957	0.149792
27	oar3_OAR›	65256723	0.244274
27	oar3_OAR›	65267344	0.032059
27	oar3_OAR›	65278477	0.244274
27	oar3_OAR›	65278685	0.032059
27	oar3_OAR›	65313074	0.244274
27	oar3_OAR›	65323846	0.244274
27	oar3_OAR›	65329540	0.14335
27	oar3_OAR›	65344121	0.032059
27	oar3_OAR›	65345804	0.244274
27	oar3_OAR›	65349674	0.14335
27	oar3_OAR›	65354704	0.07412
27	oar3_OAR›	65357519	0.032059
27	oar3_OAR›	65374974	0.244274

27	oar3_OAR›	65401855	0.244274
27	oar3_OAR›	65403547	0.244274
27	oar3_OAR›	65431302	0.14335
27	oar3_OAR›	65497519	0.149792
27	oar3_OAR›	65514259	0.149792
27	oar3_OAR›	65529243	0.07412
27	oar3_OAR›	65530442	0.149792
27	oar3_OAR›	65537213	0.07412
27	oar3_OAR›	65538479	0.251748
27	oar3_OAR›	65542915	0.015728
27	oar3_OAR›	65550516	0.07412
27	oar3_OAR›	65555987	0.427836
27	oar3_OAR›	65586747	0.032059
27	oar3_OAR›	65594027	0.07412
27	oar3_OAR›	65638609	0.032059
27	oar3_OAR›	65652453	0.076111
27	oar3_OAR›	65652998	0.114595
27	oar3_OAR›	65654459	0.038645
27	oar3_OAR›	65661922	0.114595
27	oar3_OAR›	65681072	0.135532
27	oar3_OAR›	65720973	0.176598
27	oar3_OAR›	65722070	0.081485
27	oar3_OAR›	65728021	0.119366
27	oar3_OAR›	65739562	-0.02025
27	oar3_OAR›	65750542	0.106659
27	oar3_OAR›	65775904	0.057808
27	oar3_OAR›	65837171	0.106659
27	oar3_OAR›	65884699	0.106659
27	oar3_OAR›	65890222	0.106659
27	oar3_OAR›	65908735	0.106659
27	oar3_OAR›	65917844	0.106659
27	oar3_OAR›	65928588	0.106659
27	oar3_OAR›	65952663	0.106659
27	oar3_OAR›	66046573	0.106659
27	oar3_OAR›	66063058	0.106659
27	oar3_OAR›	66070406	0.106659
27	oar3_OAR›	66134004	0.106659
27	oar3_OAR›	66190687	0.157114
27	oar3_OAR›	66192295	0.106659
27	oar3_OAR›	66214481	0.186529
27	oar3_OAR›	66217194	0.106659
27	oar3_OAR›	66222874	0.106659
27	oar3_OAR›	66262563	0.106659
27	oar3_OAR›	66290415	0.106659
27	oar3_OAR›	66303864	0.157114
27	oar3_OAR›	66326161	0.153016
27	oar3_OAR›	66351301	0.157114
27	oar3_OAR›	66353025	0.157114
27	oar3_OAR›	66369142	0.157114
27	oar3_OAR›	66379234	0.111261
27	oar3_OAR›	66397394	0.111261
27	oar3_OAR›	66425389	0.111261
27	oar3_OAR›	66470305	0.111261
27	oar3_OAR›	66481918	0.111261

27	oar3_OAR›	66512432	0.111261
27	oar3_OAR›	66512507	0.111261
27	oar3_OAR›	66517289	0.146484
27	oar3_OAR›	66522933	0.111261
27	oar3_OAR›	66555984	0.172566
27	oar3_OAR›	66568522	0.204635
27	oar3_OAR›	66572092	0.204635
27	oar3_OAR›	66611929	0.204635
27	oar3_OAR›	66627187	0.204635
27	oar3_OAR›	66639128	0.204635
27	oar3_OAR›	66658161	0.204635
27	oar3_OAR›	66673608	0.204635
27	oar3_OAR›	66679290	0.204635
27	oar3_OAR›	66698254	0.172566
27	oar3_OAR›	66709241	0.172566
27	oar3_OAR›	66731464	0.204635
27	oar3_OAR›	66731528	0.204635
27	oar3_OAR›	66753202	0.204635
27	oar3_OAR›	66777216	0.204635
27	oar3_OAR›	66777828	0.204635
27	oar3_OAR›	66781573	0.152466
27	oar3_OAR›	66788601	0.01982
27	oar3_OAR›	66792034	0.204635
27	oar3_OAR›	66797024	0.01982
27	oar3_OAR›	66801060	0.152466
27	oar3_OAR›	66804539	0.01982
27	oar3_OAR›	66812557	0.204635
27	oar3_OAR›	66837861	0.18245
27	oar3_OAR›	66851301	0.18245
27	oar3_OAR›	66853548	0.16209
27	oar3_OAR›	66858550	0.16209
27	oar3_OAR›	66867898	0.16209
27	oar3_OAR›	66892676	0.16209
27	oar3_OAR›	66922389	0.16209
27	oar3_OAR›	66925345	0.16209
27	oar3_OAR›	66969160	0.14742
27	oar3_OAR›	67004561	0.18245
27	oar3_OAR›	67016034	0.18245
27	oar3_OAR›	67017692	0.18245
27	oar3_OAR›	67029891	0.14742
27	oar3_OAR›	67073484	0.14742
27	oar3_OAR›	67085752	0.18245
27	oar3_OAR›	67088770	0.18245
27	oar3_OAR›	67089819	0.18245
27	oar3_OAR›	67114171	0.107777
27	oar3_OAR›	67154378	0.132458
27	oar3_OAR›	67169531	0.132458
27	oar3_OAR›	67223237	0.147078
27	oar3_OAR›	67238161	0.107777
27	oar3_OAR›	67264104	0.018804
27	oar3_OAR›	67300705	0.018804
27	oar3_OAR›	67319570	-0.00446
27	oar3_OAR›	67387844	0.132458
27	oar3_OAR›	67491781	0.044645

27	oar3_OAR›	67518891	0.018804
27	oar3_OAR›	67559282	0.018804
27	oar3_OAR›	67564457	0.044645
27	oar3_OAR›	67579140	0.044645
27	oar3_OAR›	67610418	0.053273
27	oar3_OAR›	67620633	0.018804
27	oar3_OAR›	67632939	0.121981
27	oar3_OAR›	67635478	0.044645
27	oar3_OAR›	67641485	0.044645
27	oar3_OAR›	67686817	0.072914
27	oar3_OAR›	67709291	0.106461
27	oar3_OAR›	67718793	0.018804
27	oar3_OAR›	67794610	0.018804
27	oar3_OAR›	67888286	0.143831
27	oar3_OAR›	67928840	0.043755
27	oar3_OAR›	67929733	0.043755
27	oar3_OAR›	67963788	0.043755
27	oar3_OAR›	67963925	0.043755
27	oar3_OAR›	67973626	0.043755
27	oar3_OAR›	67986514	0.043755
27	oar3_OAR›	68018424	0.143831
27	oar3_OAR›	68018601	0.043755
27	oar3_OAR›	68031006	0.148695
27	oar3_OAR›	68045607	0.043755
27	oar3_OAR›	68047909	0.043755
27	oar3_OAR›	68154710	0.032517
27	oar3_OAR›	68162762	0.032517
27	oar3_OAR›	68177262	0.032517
27	oar3_OAR›	68193467	0.032517
27	oar3_OAR›	68231152	0.032517
27	oar3_OAR›	68244088	0.426813
27	oar3_OAR›	68265090	0.032517
27	oar3_OAR›	68316905	0.032517
27	oar3_OAR›	68333628	0.032517
27	oar3_OAR›	68352002	0.032517
27	oar3_OAR›	68364173	0.032517
27	oar3_OAR›	68366931	0.214924
27	oar3_OAR›	68422220	0.032517
27	oar3_OAR›	68422958	0.032517
27	oar3_OAR›	68423252	0.135251
27	oar3_OAR›	68423298	0.135251
27	oar3_OAR›	68433434	0.032517
27	oar3_OAR›	68433844	0.032517
27	oar3_OAR›	68435868	0.032517
27	oar3_OAR›	68447191	0.032517
27	oar3_OAR›	68447342	0.214924
27	oar3_OAR›	68478359	0.135251
27	oar3_OAR›	68479272	0.036019
27	oar3_OAR›	68480996	0.032517
27	oar3_OAR›	68517285	0.135251
27	oar3_OAR›	68523863	0.214924
27	oar3_OAR›	68529261	0.032517
27	oar3_OAR›	68535799	0.032517
27	oar3_OAR›	68546987	0.214924

27	oar3_OAR›	68589071	0.036019
27	oar3_OAR›	68600290	0.036019
27	oar3_OAR›	68600725	0.032517
27	oar3_OAR›	68620384	0.032517
27	oar3_OAR›	68621015	0.032517
27	oar3_OAR›	68629233	0.032517
27	oar3_OAR›	68636374	0.032517
27	oar3_OAR›	68666452	0.032517
27	oar3_OAR›	68717233	0.236416
27	oar3_OAR›	68720700	0.036019
27	oar3_OAR›	68753705	0.004461
27	oar3_OAR›	68754208	0.004461
27	oar3_OAR›	68756142	0.292782
27	oar3_OAR›	68769453	0.267399
27	oar3_OAR›	68783090	0.004461
27	oar3_OAR›	68794098	0.292782
27	oar3_OAR›	68794891	0.292782
27	oar3_OAR›	68800633	0.017742
27	oar3_OAR›	68836121	0.017742
27	oar3_OAR›	68874570	0.317774
27	oar3_OAR›	68876511	0.004461
27	oar3_OAR›	68934554	0.017742
27	oar3_OAR›	68976956	0.017742
27	oar3_OAR›	69091693	0.295143
27	oar3_OAR›	69152886	0.340554
27	oar3_OAR›	69153151	0.004461
27	oar3_OAR›	69194338	0.017742
27	oar3_OAR›	69211472	0.004461
27	oar3_OAR›	69224801	0.017742
27	oar3_OAR›	69307671	0.253077
27	oar3_OAR›	69315118	0.011365
27	oar3_OAR›	69462380	0.018466
27	oar3_OAR›	69483949	0.265629
27	oar3_OAR›	69530634	0.011365
27	oar3_OAR›	69533217	0.018466
27	oar3_OAR›	69545406	0.018466
27	oar3_OAR›	69549749	0.018466
27	oar3_OAR›	69557687	0.018466
27	oar3_OAR›	69558836	0.018466
27	oar3_OAR›	69563113	0.018466
27	oar3_OAR›	69568982	0.018466
27	oar3_OAR›	69570142	0.126714
27	oar3_OAR›	69578397	0.018466
27	oar3_OAR›	69581888	0.018466
27	oar3_OAR›	69624447	0.126714
27	oar3_OAR›	69626653	0.018466
27	oar3_OAR›	69657230	0.018466
27	oar3_OAR›	69658569	0.018466
27	oar3_OAR›	69659590	0.018466
27	oar3_OAR›	69697389	0.018466
27	oar3_OAR›	69770337	0.011365
27	oar3_OAR›	69810046	0.018466
27	oar3_OAR›	69853001	0.011365
27	oar3_OAR›	69990733	0.191118

27	oar3_OAR›	69998888	0.178107
27	oar3_OAR›	70014320	0.178107
27	oar3_OAR›	70042673	0.191118
27	oar3_OAR›	70112709	0.191118
27	oar3_OAR›	70131274	0.126714
27	oar3_OAR›	70218624	0.129595
27	oar3_OAR›	70235658	0.129595
27	oar3_OAR›	70330447	0.141867
27	oar3_OAR›	70357084	0.141867
27	oar3_OAR›	70369186	0.141867
27	oar3_OAR›	70551584	0.141867
27	oar3_OAR›	70614867	-0.01737
27	oar3_OAR›	70644726	-0.01737
27	oar3_OAR›	70775282	0.063386
27	oar3_OAR›	70805340	0.093904
27	oar3_OAR›	70823848	0.093904
27	oar3_OAR›	70827843	-0.01737
27	oar3_OAR›	70944061	-0.01737
27	oar3_OAR›	71060162	-0.01737
27	oar3_OAR›	71084857	-0.01737
27	oar3_OAR›	71162545	-0.02419
27	oar3_OAR›	71188808	0.029607
27	oar3_OAR›	71359416	0.224245
27	oar3_OAR›	71364206	-0.01737
27	oar3_OAR›	71389901	0.252457
27	oar3_OAR›	71445260	0.252457
27	oar3_OAR›	71450851	0.224245
27	oar3_OAR›	71488501	0.252457
27	oar3_OAR›	71489320	-0.01737
27	oar3_OAR›	71492668	0.224245
27	oar3_OAR›	71655937	0.252457
27	oar3_OAR›	71656418	0.220061
27	oar3_OAR›	71671293	0.224245
27	oar3_OAR›	71754381	0.245288
27	oar3_OAR›	71762649	0.220884
27	oar3_OAR›	71771904	0.074614
27	oar3_OAR›	71920917	0.198141
27	oar3_OAR›	71959923	0.097816
27	oar3_OAR›	72002213	0.05576
27	oar3_OAR›	72161558	0.108876
27	oar3_OAR›	72165327	0.108876
27	oar3_OAR›	72228164	0.05576
27	oar3_OAR›	72370000	0.05576
27	oar3_OAR›	72380149	0.108876
27	oar3_OAR›	72393145	0.05576
27	oar3_OAR›	72394444	0.108876
27	oar3_OAR›	72528494	0.108876
27	oar3_OAR›	72582172	0.108876
27	oar3_OAR›	72592531	0.05576
27	oar3_OAR›	72650349	0.108876
27	oar3_OAR›	72683650	0.05576
27	oar3_OAR›	72702216	0.1216
27	oar3_OAR›	72717390	-0.01737
27	oar3_OAR›	72744881	0.05576

27	oar3_OAR›	72776840	0.108876
27	oar3_OAR›	72814959	0.108876
27	oar3_OAR›	72815940	0.05576
27	oar3_OAR›	72842091	0.075222
27	oar3_OAR›	72940652	0.108876
27	oar3_OAR›	72940764	0.107161
27	oar3_OAR›	72955695	0.108876
27	oar3_OAR›	73077640	0.106113
27	oar3_OAR›	73086058	0.063221
27	oar3_OAR›	73101804	0.063221
27	oar3_OAR›	73120432	0.052764
27	oar3_OAR›	73151778	0.063221
27	oar3_OAR›	73167971	0.098673
27	oar3_OAR›	73168991	0.05493
27	oar3_OAR›	73174589	0.098673
27	oar3_OAR›	73197917	0.063221
27	oar3_OAR›	73198255	0.063221
27	oar3_OAR›	73238991	0.093904
27	oar3_OAR›	73265522	0.153738
27	oar3_OAR›	73284991	0.196013
27	oar3_OAR›	73288140	-0.00606
27	oar3_OAR›	73308527	0.196013
27	oar3_OAR›	73347728	0.044085
27	oar3_OAR›	73361547	0.042323
27	oar3_OAR›	73407208	0.23193
27	oar3_OAR›	73410488	0.049315
27	oar3_OAR›	73420647	0.044085
27	oar3_OAR›	73427559	0.23193
27	oar3_OAR›	73454886	0.23193
27	oar3_OAR›	73456237	0.23193
27	oar3_OAR›	73481965	0.23193
27	oar3_OAR›	73498528	0.23193
27	oar3_OAR›	73504342	0.049315
27	oar3_OAR›	73542274	0.276694
27	oar3_OAR›	73553263	0.276694
27	oar3_OAR›	73565333	0.276694
27	oar3_OAR›	73574478	0.039598
27	oar3_OAR›	73576908	0.276694
27	oar3_OAR›	73592901	0.276694
27	oar3_OAR›	73608291	0.276694
27	oar3_OAR›	73629126	0.276694
27	oar3_OAR›	73641813	0.017522
27	oar3_OAR›	73642789	0.189446
27	oar3_OAR›	73656853	0.017522
27	oar3_OAR›	73657298	0.189446
27	oar3_OAR›	73666316	0.276694
27	oar3_OAR›	73667274	0.017522
27	oar3_OAR›	73679821	0.276694
27	oar3_OAR›	73686214	0.276694
27	oar3_OAR›	73686774	0.276694
27	oar3_OAR›	73691806	0.276694
27	oar3_OAR›	73701353	0.276694
27	oar3_OAR›	73709272	0.276694
27	oar3_OAR›	73718120	0.276694

27	oar3_OAR›	73731000	0.017522
27	oar3_OAR›	73736776	0.276694
27	oar3_OAR›	73743005	0.276694
27	oar3_OAR›	73753121	0.017522
27	oar3_OAR›	73753867	0.276694
27	oar3_OAR›	73754904	0.017522
27	oar3_OAR›	73775409	0.276694
27	oar3_OAR›	73778941	0.017522
27	oar3_OAR›	73794258	0.017522
27	oar3_OAR›	73794587	0.276694
27	oar3_OAR›	73805113	0.007726
27	oar3_OAR›	73807736	0.276694
27	oar3_OAR›	73813772	0.017522
27	oar3_OAR›	73815310	0.276694
27	oar3_OAR›	73836007	0.276694
27	oar3_OAR›	73844000	0.276694
27	oar3_OAR›	73845660	0.017522
27	oar3_OAR›	73869482	0.250354
27	oar3_OAR›	73887140	0.250354
27	oar3_OAR›	73902594	0.250354
27	oar3_OAR›	73906977	0.250354
27	oar3_OAR›	73913865	0.250354
27	oar3_OAR›	73914853	0.250354
27	oar3_OAR›	73918643	0.250354
27	oar3_OAR›	73920757	0.250354
27	oar3_OAR›	73930602	0.250354
27	oar3_OAR›	73938930	0.250354
27	oar3_OAR›	73947324	0.017522
27	oar3_OAR›	73989703	0.250354
27	oar3_OAR›	73994257	0.017522
27	oar3_OAR›	73994888	0.017522
27	oar3_OAR›	74002581	0.017522
27	oar3_OAR›	74007577	0.250354
27	oar3_OAR›	74018169	0.017522
27	oar3_OAR›	74027203	0.017522
27	oar3_OAR›	74029840	0.017522
27	oar3_OAR›	74047861	0.017522
27	oar3_OAR›	74064152	0.017522
27	oar3_OAR›	74064814	0.017522
27	oar3_OAR›	74075964	0.017522
27	oar3_OAR›	74078065	0.017522
27	oar3_OAR›	74080431	0.017522
27	oar3_OAR›	74087580	0.017522
27	oar3_OAR›	74110598	0.23846
27	oar3_OAR›	74127267	0.017522
27	oar3_OAR›	74138049	0.23846
27	oar3_OAR›	74140523	0.23846
27	oar3_OAR›	74140883	0.23846
27	oar3_OAR›	74159336	0.017522
27	oar3_OAR›	74159840	0.23846
27	oar3_OAR›	74169993	0.1511
27	oar3_OAR›	74175839	0.23846
27	oar3_OAR›	74181490	0.23846
27	oar3_OAR›	74184751	0.23846

27	oar3_OAR›	74199324	0.23846
27	oar3_OAR›	74213493	0.228729
27	oar3_OAR›	74248264	0.130616
27	oar3_OAR›	74254809	0.228729
27	oar3_OAR›	74256406	0.228729
27	oar3_OAR›	74262700	0.228729
27	oar3_OAR›	74269213	0.130616
27	oar3_OAR›	74271008	0.228729
27	oar3_OAR›	74272442	0.228729
27	oar3_OAR›	74282568	0.228729
27	oar3_OAR›	74286064	0.228729
27	oar3_OAR›	74286200	0.228729
27	oar3_OAR›	74292065	0.228729
27	oar3_OAR›	74293892	0.228729
27	oar3_OAR›	74312753	0.161499
27	oar3_OAR›	74335483	0.148069
27	oar3_OAR›	74345267	0.272691
27	oar3_OAR›	74365043	0.161499
27	oar3_OAR›	74397184	0.161499
27	oar3_OAR›	74401449	0.272691
27	oar3_OAR›	74401751	-0.02926
27	oar3_OAR›	74407433	0.230533
27	oar3_OAR›	74409440	0.084431
27	oar3_OAR›	74423311	0.084431
27	oar3_OAR›	74443097	0.084431
27	oar3_OAR›	74449618	0.230533
27	oar3_OAR›	74452130	0.084431
27	oar3_OAR›	74460736	0.12737
27	oar3_OAR›	74462632	0.084431
27	oar3_OAR›	74469592	0.084431
27	oar3_OAR›	74470589	0.230533
27	oar3_OAR›	74511584	0.208289
27	oar3_OAR›	74524283	0.208289
27	oar3_OAR›	74535804	0.000889
27	oar3_OAR›	74536567	0.078981
27	oar3_OAR›	74576875	0.071649
27	oar3_OAR›	74577009	0.208289
27	oar3_OAR›	74578665	0.078981
27	oar3_OAR›	74590448	0.091283
27	oar3_OAR›	74603684	0.1887
27	oar3_OAR›	74606588	0.1887
27	oar3_OAR›	74655928	0.1887
27	oar3_OAR›	74660336	0.1887
27	oar3_OAR›	74661304	0.1887
27	oar3_OAR›	74668484	0.1887
27	oar3_OAR›	74678182	0.1887
27	oar3_OAR›	74690678	0.1887
27	oar3_OAR›	74691989	0.1887
27	oar3_OAR›	74707848	-0.02593
27	oar3_OAR›	74717067	-0.02593
27	oar3_OAR›	74734577	0.204949
27	oar3_OAR›	74763338	0.000889
27	oar3_OAR›	74784674	0.1887
27	oar3_OAR›	74794874	0.1887

27	oar3_OAR>	74799643	0.1887
27	oar3_OAR>	74857719	0.091283
27	oar3_OAR>	74872692	0.091283
27	oar3_OAR>	74877174	-0.02926
27	oar3_OAR>	74878651	-0.01737
27	oar3_OAR>	74885862	0.167339
27	oar3_OAR>	74895942	0.091283
27	oar3_OAR>	74909127	-0.01737
27	oar3_OAR>	74920467	0.097488
27	oar3_OAR>	74922040	0.000363
27	oar3_OAR>	74943260	0.097488
27	oar3_OAR>	74944617	0.097488
27	oar3_OAR>	74963161	0.097488
27	oar3_OAR>	74966063	0.000363
27	oar3_OAR>	75015812	0.140262
27	oar3_OAR>	75021759	0.000889
27	oar3_OAR>	75027393	0.000889
27	oar3_OAR>	75033878	0.087266
27	oar3_OAR>	75035375	0.000889
27	oar3_OAR>	75039009	0.056203
27	oar3_OAR>	75040456	0.195402
27	oar3_OAR>	75085349	-0.02855
27	oar3_OAR>	75085461	-0.03397
27	oar3_OAR>	75086980	-0.03397
27	oar3_OAR>	75106849	-0.03164
27	oar3_OAR>	75108539	-0.02855
27	oar3_OAR>	75119026	-0.03164
27	oar3_OAR>	75144532	-0.03504
27	oar3_OAR>	75144606	-0.01705
27	oar3_OAR>	75148786	-0.01997
27	oar3_OAR>	75150858	-0.01705
27	oar3_OAR>	75172218	-0.01705
27	oar3_OAR>	75174938	-0.02512
27	oar3_OAR>	75186984	-0.01199
27	oar3_OAR>	75193564	-0.01962
27	oar3_OAR>	75200159	-0.02374
27	oar3_OAR>	75204990	-0.02374
27	oar3_OAR>	75220536	0.002868
27	oar3_OAR>	75220943	-0.02997
27	oar3_OAR>	75253855	7.86E-05
27	oar3_OAR>	75254330	-0.02997
27	oar3_OAR>	75296315	0.0764
27	oar3_OAR>	75298507	0.0764
27	oar3_OAR>	75300772	0.126639
27	oar3_OAR>	75301837	-0.0107
27	oar3_OAR>	75304118	-0.00285
27	oar3_OAR>	75311031	-0.02026
27	oar3_OAR>	75311103	-0.02174
27	oar3_OAR>	75316686	0.04239
27	oar3_OAR>	75327494	0.011151
27	oar3_OAR>	75342828	-0.01304
27	oar3_OAR>	75349003	-0.03249
27	oar3_OAR>	75349497	0.04239
27	oar3_OAR>	75352659	0.079876

27	oar3_OAR›	75353534	-0.00364
27	oar3_OAR›	75360550	-0.01737
27	oar3_OAR›	75365538	-0.03249
27	oar3_OAR›	75368325	0.073101
27	oar3_OAR›	75382484	-0.00364
27	oar3_OAR›	75385441	0.079876
27	oar3_OAR›	75397038	0.033987
27	oar3_OAR›	75398086	0.033987
27	oar3_OAR›	75411781	0.04358
27	oar3_OAR›	75422502	0.04358
27	oar3_OAR›	75423631	0.04358
27	oar3_OAR›	75434923	0.03064
27	oar3_OAR›	75438476	0.016664
27	oar3_OAR›	75441698	0.021018
27	oar3_OAR›	75460189	-0.00597
27	oar3_OAR›	75460369	-0.01368
27	oar3_OAR›	75462024	-0.01872
27	oar3_OAR›	75473692	-0.02793
27	oar3_OAR›	75487650	0.059225
27	oar3_OAR›	75493738	0.032582
27	oar3_OAR›	75493942	0.107211
27	oar3_OAR›	75512126	0.025392
27	oar3_OAR›	75514332	0.031105
27	oar3_OAR›	75521670	0.054992
27	oar3_OAR›	75530333	0.000374
27	oar3_OAR›	75545444	0.078664
27	oar3_OAR›	75546876	0.143008
27	oar3_OAR›	75557730	0.113371
27	oar3_OAR›	75566255	0.113371
27	oar3_OAR›	75571798	0.132635
27	oar3_OAR›	75574494	0.019974
27	oar3_OAR›	75589146	0.092691
27	oar3_OAR›	75604867	0.120834
27	oar3_OAR›	75607923	NA
27	oar3_OAR›	75610788	0.120834
27	oar3_OAR›	75663662	NA
27	oar3_OAR›	75663800	0.05462
27	oar3_OAR›	75666783	0.048295
27	oar3_OAR›	75668977	-0.02993
27	oar3_OAR›	75736537	0.048295
27	oar3_OAR›	75739950	0.05462
27	oar3_OAR›	75753919	NA
27	oar3_OAR›	75759217	NA
27	oar3_OAR›	75763844	-0.02411
27	oar3_OAR›	75767354	0.048295
27	oar3_OAR›	75779535	-0.00114
27	oar3_OAR›	75780286	0.017546
27	oar3_OAR›	75795730	0.000889
27	oar3_OAR›	75796786	0.000889
27	oar3_OAR›	75797524	0.032258
27	oar3_OAR›	75799450	0.032258
27	oar3_OAR›	75805469	0.000889
27	oar3_OAR›	75815634	0.037917
27	oar3_OAR›	75844719	0.039637

27	oar3_OAR›	75854069	0.012289
27	oar3_OAR›	75875828	0.012349
27	oar3_OAR›	75878122	0.025488
27	oar3_OAR›	75883974	0.008007
27	oar3_OAR›	75894358	0.076913
27	oar3_OAR›	75898636	0.024431
27	oar3_OAR›	75899507	0.021533
27	oar3_OAR›	75900941	-0.03117
27	oar3_OAR›	75901683	0.024431
27	oar3_OAR›	75913375	-0.00352
27	oar3_OAR›	75941869	-0.03437
27	oar3_OAR›	75959647	-0.00352
27	oar3_OAR›	75965643	0.0104
27	oar3_OAR›	75974764	0.008782
27	oar3_OAR›	75978037	0.008782
27	oar3_OAR›	75979090	0.008782
27	oar3_OAR›	75993593	0.138416
27	oar3_OAR›	76016790	0.138416
27	oar3_OAR›	76017638	-0.02356
27	oar3_OAR›	76022596	0.138416
27	oar3_OAR›	76029935	0.138416
27	oar3_OAR›	76049165	-0.02629
27	oar3_OAR›	76054851	-0.03509
27	oar3_OAR›	76056496	0.000821
27	oar3_OAR›	76067646	0.038645
27	oar3_OAR›	76094556	0.048281
27	oar3_OAR›	76108255	-0.03437
27	oar3_OAR›	76113858	-0.03437
27	oar3_OAR›	76132850	-0.04114
27	oar3_OAR›	76141989	-0.01657
27	oar3_OAR›	76147908	-0.01657
27	oar3_OAR›	76154489	-0.01295
27	oar3_OAR›	76160431	-0.02362
27	oar3_OAR›	76166904	-0.01584
27	oar3_OAR›	76173268	-0.01295
27	oar3_OAR›	76175763	0.116993
27	oar3_OAR›	76176165	0.125464
27	oar3_OAR›	76186363	-0.01295
27	oar3_OAR›	76238951	-0.03097
27	oar3_OAR›	76244777	0.008723
27	oar3_OAR›	76302191	-0.03097
27	oar3_OAR›	76332856	-0.03097
27	oar3_OAR›	76344627	-0.03097
27	oar3_OAR›	76345936	-0.03097
27	oar3_OAR›	76357897	-0.03097
27	oar3_OAR›	76361847	-0.03097
27	oar3_OAR›	76368031	-0.01567
27	oar3_OAR›	76368140	-0.02849
27	oar3_OAR›	76374925	-0.03479
27	oar3_OAR›	76376338	-0.01567
27	oar3_OAR›	76379165	-0.02562
27	oar3_OAR›	76383955	0.020529
27	oar3_OAR›	76385127	0.020529
27	oar3_OAR›	76394562	0.020529

27	oar3_OAR?	76416444	0.020529
27	oar3_OAR?	76448116	0.001279
27	oar3_OAR?	76452709	-0.02453
27	oar3_OAR?	76454929	0.001279
27	oar3_OAR?	76473701	0.001279
27	oar3_OAR?	76495893	-0.02562
27	oar3_OAR?	76565028	0.000889
27	oar3_OAR?	76592115	0.145743
27	oar3_OAR?	76593325	0.000889
27	oar3_OAR?	76594417	0.117073
27	oar3_OAR?	76600837	0.145743
27	oar3_OAR?	76616343	0.110922
27	oar3_OAR?	76617181	0.000889
27	oar3_OAR?	76617429	0.102307
27	oar3_OAR?	76619178	0.110922
27	oar3_OAR?	76643564	0.102307
27	oar3_OAR?	76644528	0.194595
27	oar3_OAR?	76650461	0.117073
27	oar3_OAR?	76650551	0.194595
27	oar3_OAR?	76712136	0.182402
27	oar3_OAR?	76749938	-0.02774
27	oar3_OAR?	76762758	0.105795
27	oar3_OAR?	76765152	0.109905
27	oar3_OAR?	76771831	0.203721
27	oar3_OAR?	76779869	0.176306
27	oar3_OAR?	76780471	0.203721
27	oar3_OAR?	76785909	0.186988
27	oar3_OAR?	76792785	0.167227
27	oar3_OAR?	76796489	-0.00836
27	oar3_OAR?	76800239	-0.00446
27	oar3_OAR?	76805833	0.127276
27	oar3_OAR?	76814451	0.090427
27	oar3_OAR?	76816283	-0.00673
27	oar3_OAR?	76820498	0.000363
27	oar3_OAR?	76828818	0.193888
27	oar3_OAR?	76830734	0.193888
27	oar3_OAR?	76839306	0.138282
27	oar3_OAR?	76845430	0.053273
27	oar3_OAR?	76847301	0.101168
27	oar3_OAR?	76851125	0.223555
27	oar3_OAR?	76856383	0.223555
27	oar3_OAR?	76860977	0.022123
27	oar3_OAR?	76865114	0.089766
27	oar3_OAR?	76873061	0.32109
27	oar3_OAR?	76917122	0.090094
27	oar3_OAR?	76923489	0.199415
27	oar3_OAR?	76953170	0.199415
27	oar3_OAR?	76962466	0.174546
27	oar3_OAR?	76984507	0.199415
27	oar3_OAR?	77017466	0.021279
27	oar3_OAR?	77022060	0.101522
27	oar3_OAR?	77024111	0.329444
27	oar3_OAR?	77030029	0.018804
27	oar3_OAR?	77046828	-0.03869

27	oar3_OAR›	77047401	0.032582
27	oar3_OAR›	77076715	0.04814
27	oar3_OAR›	77080809	0.20077
27	oar3_OAR›	77085113	0.091062
27	oar3_OAR›	77096530	0.081953
27	oar3_OAR›	77099263	0.04814
27	oar3_OAR›	77107233	-0.01001
27	oar3_OAR›	77111649	0.032582
27	oar3_OAR›	77113989	0.04814
27	oar3_OAR›	77137946	0.04522
27	oar3_OAR›	77144219	0.099966
27	oar3_OAR›	77145424	0.08853
27	oar3_OAR›	77176461	0.088785
27	oar3_OAR›	77181189	0.066444
27	oar3_OAR›	77187427	0.066444
27	oar3_OAR›	77194059	0.066444
27	oar3_OAR›	77197590	0.108575
27	oar3_OAR›	77206075	0.108575
27	oar3_OAR›	77216236	0.085958
27	oar3_OAR›	77216277	-0.01423
27	oar3_OAR›	77253089	NA
27	oar3_OAR›	77311757	0.137464
27	oar3_OAR›	77326832	0.000889
27	oar3_OAR›	77331912	0.041609
27	oar3_OAR›	77339488	-0.02593
27	oar3_OAR›	77341049	0.079446
27	oar3_OAR›	77344747	-0.01731
27	oar3_OAR›	77347836	0.124095
27	oar3_OAR›	77350768	-0.01731
27	oar3_OAR›	77353781	0.124095
27	oar3_OAR›	77356355	-0.02593
27	oar3_OAR›	77362610	0.15957
27	oar3_OAR›	77374839	-0.02593
27	oar3_OAR›	77376307	-0.02593
27	oar3_OAR›	77382547	0.189627
27	oar3_OAR›	77405054	NA
27	oar3_OAR›	77420675	0.018804
27	oar3_OAR›	77428428	0.249695
27	oar3_OAR›	77430977	-0.01069
27	oar3_OAR›	77442285	0.018804
27	oar3_OAR›	77447214	0.249695
27	oar3_OAR›	77459676	0.249695
27	oar3_OAR›	77465015	0.000889
27	oar3_OAR›	77467897	0.000889
27	oar3_OAR›	77478819	0.000889
27	oar3_OAR›	77490749	0.237346
27	oar3_OAR›	77509056	0.08981
27	oar3_OAR›	77517751	0.01388
27	oar3_OAR›	77524247	0.01388
27	oar3_OAR›	77524582	0.01388
27	oar3_OAR›	77530558	0.01388
27	oar3_OAR›	77533232	0.01388
27	oar3_OAR›	77539474	0.021081
27	oar3_OAR›	77541194	0.01388

27	oar3_OAR›	77542251	0.021081
27	oar3_OAR›	77570924	-0.00901
27	oar3_OAR›	77580034	-0.01871
27	oar3_OAR›	77585880	-0.01871
27	oar3_OAR›	77589551	-0.01871
27	oar3_OAR›	77591005	-0.01871
27	oar3_OAR›	77597342	-0.01871
27	oar3_OAR›	77606244	-0.02485
27	oar3_OAR›	77652893	0.043661
27	oar3_OAR›	77655645	-0.00673
27	oar3_OAR›	77663518	-0.00673
27	oar3_OAR›	77680039	0.119621
27	oar3_OAR›	77695471	-0.01737
27	oar3_OAR›	77732923	-0.00606
27	oar3_OAR›	77747463	0.114255
27	oar3_OAR›	77763135	0.069823
27	oar3_OAR›	77767602	0.002306
27	oar3_OAR›	77769650	0.072883
27	oar3_OAR›	77788613	0.052399
27	oar3_OAR›	77804840	-0.01737
27	oar3_OAR›	77807703	0.356704
27	oar3_OAR›	77808654	-0.00673
27	oar3_OAR›	77819560	-0.0353
27	oar3_OAR›	77827887	0.247544
27	oar3_OAR›	77841100	-0.013
27	oar3_OAR›	77846976	-0.02784
27	oar3_OAR›	77849469	0.33627
27	oar3_OAR›	77855853	0.260989
27	oar3_OAR›	77856713	0.305632
27	oar3_OAR›	77872830	-0.03456
27	oar3_OAR›	77878253	-0.03456
27	oar3_OAR›	77878365	0.280426
27	oar3_OAR›	77883858	0.247544
27	oar3_OAR›	77884057	0.039481
27	oar3_OAR›	77903105	0.317716
27	oar3_OAR›	77907370	0.314674
27	oar3_OAR›	77925715	0.317716
27	oar3_OAR›	77925960	0.255936
27	oar3_OAR›	77942325	0.039481
27	oar3_OAR›	77942553	-0.01567
27	oar3_OAR›	77944456	0.314674
27	oar3_OAR›	77946289	0.012043
27	oar3_OAR›	77949526	0.180494
27	oar3_OAR›	77954865	0.059823
27	oar3_OAR›	77956891	0.13594
27	oar3_OAR›	77959048	0.12662
27	oar3_OAR›	77960553	0.011937
27	oar3_OAR›	77966450	0.070305
27	oar3_OAR›	77987216	0.053463
27	oar3_OAR›	77988043	0.005751
27	oar3_OAR›	77992129	-0.01069
27	oar3_OAR›	77993324	-0.01224
27	oar3_OAR›	78011950	0.030434
27	oar3_OAR›	78016948	0.050917

27	oar3_OAR›	78018772	0.001415
27	oar3_OAR›	78021403	-0.01224
27	oar3_OAR›	78023906	0.022793
27	oar3_OAR›	78030404	0.02221
27	oar3_OAR›	78030456	0.022793
27	oar3_OAR›	78031801	0.022793
27	oar3_OAR›	78098783	0.044121
27	oar3_OAR›	78171702	-0.03249
27	oar3_OAR›	78171753	-0.03249
27	oar3_OAR›	78191150	-0.00999
27	oar3_OAR›	78211472	-0.01932
27	oar3_OAR›	78211995	0.055085
27	oar3_OAR›	78248202	-0.01932
27	oar3_OAR›	78251550	-0.01932
27	oar3_OAR›	78252087	-0.02562
27	oar3_OAR›	78267534	-0.00322
27	oar3_OAR›	78274121	-0.02555
27	oar3_OAR›	78277262	-0.02376
27	oar3_OAR›	78278545	-0.02336
27	oar3_OAR›	78287339	0.09868
27	oar3_OAR›	78288421	0.024187
27	oar3_OAR›	78288509	0.09868
27	oar3_OAR›	78290004	0.024187
27	oar3_OAR›	78290661	0.024187
27	oar3_OAR›	78298997	0.024187
27	oar3_OAR›	78299054	0.09868
27	oar3_OAR›	78302173	0.024187
27	oar3_OAR›	78305765	0.09868
27	oar3_OAR›	78312150	-0.013
27	oar3_OAR›	78312987	0.015992
27	oar3_OAR›	78313100	0.015992
27	oar3_OAR›	78322090	0.0104
27	oar3_OAR›	78324587	-0.03054
27	oar3_OAR›	78327270	-0.01402
27	oar3_OAR›	78336105	-0.013
27	oar3_OAR›	78343138	-0.00109
27	oar3_OAR›	78344523	-0.02179
27	oar3_OAR›	78346935	-0.00109
27	oar3_OAR›	78347473	0.000161
27	oar3_OAR›	78355217	-0.00109
27	oar3_OAR›	78356206	-0.0115
27	oar3_OAR›	78369438	-0.02179
27	oar3_OAR›	78375735	-0.00109
27	oar3_OAR›	78382992	-0.00872
27	oar3_OAR›	78393864	-0.02812
27	oar3_OAR›	78394673	-0.02812
27	oar3_OAR›	78404834	-0.02562
27	oar3_OAR›	78433005	-0.02179
27	oar3_OAR›	78433362	-0.00057
27	oar3_OAR›	78439754	-0.03151
27	oar3_OAR›	78453007	-0.03151
27	oar3_OAR›	78466323	-0.01142
27	oar3_OAR›	78467360	0.015096
27	oar3_OAR›	78467600	0.015096

27	oar3_OAR›	78468862	-0.00755
27	oar3_OAR›	78477463	0.026817
27	oar3_OAR›	78495563	-0.01142
27	oar3_OAR›	78509441	-0.01214
27	oar3_OAR›	78514123	0.004356
27	oar3_OAR›	78514570	0.026817
27	oar3_OAR›	78523920	-0.00997
27	oar3_OAR›	78526215	0.026817
27	oar3_OAR›	78530974	0.004356
27	oar3_OAR›	78534741	-0.01135
27	oar3_OAR›	78535495	0.026817
27	oar3_OAR›	78540312	-0.03151
27	oar3_OAR›	78550426	-0.01962
27	oar3_OAR›	78561838	-0.01879
27	oar3_OAR›	78562123	-0.01879
27	oar3_OAR›	78571608	-0.00998
27	oar3_OAR›	78572113	-0.00998
27	oar3_OAR›	78573308	-0.03151
27	oar3_OAR›	78594284	-0.00998
27	oar3_OAR›	78596678	-0.03151
27	oar3_OAR›	78598108	0.020415
27	oar3_OAR›	78604618	-0.00998
27	oar3_OAR›	78608804	-0.03202
27	oar3_OAR›	78643616	-0.02318
27	oar3_OAR›	78644419	-0.02278
27	oar3_OAR›	78644470	0.01985
27	oar3_OAR›	78644510	-0.02278
27	oar3_OAR›	78650383	0.01985
27	oar3_OAR›	78671781	-0.02179
27	oar3_OAR›	78676349	-0.02049
27	oar3_OAR›	78678315	0.137311
27	oar3_OAR›	78678579	0.137311
27	oar3_OAR›	78680448	0.137311
27	oar3_OAR›	78681462	0.020782
27	oar3_OAR›	78691502	-0.02158
27	oar3_OAR›	78692558	0.07748
27	oar3_OAR›	78694308	-0.02411
27	oar3_OAR›	78714356	0.115972
27	oar3_OAR›	78716211	0.004689
27	oar3_OAR›	78723222	0.000889
27	oar3_OAR›	78733336	-0.00855
27	oar3_OAR›	78739877	0.02564
27	oar3_OAR›	78742467	-0.0152
27	oar3_OAR›	78743693	0.080918
27	oar3_OAR›	78745582	0.004183
27	oar3_OAR›	78756763	0.059315
27	oar3_OAR›	78768183	-0.02716
27	oar3_OAR›	78769212	0.066871
27	oar3_OAR›	78771957	-0.00777
27	oar3_OAR›	78774681	0.05584
27	oar3_OAR›	78787706	0.12328
27	oar3_OAR›	78790273	0.12328
27	oar3_OAR›	78791283	0.12328
27	oar3_OAR›	78816672	0.053463

27	oar3_OAR}	78817468	0.053463
27	oar3_OAR}	78818062	0.021705
27	oar3_OAR}	78818285	0.053463
27	oar3_OAR}	78823262	0.030586
27	oar3_OAR}	78826288	0.12328
27	oar3_OAR}	78828167	0.05823
27	oar3_OAR}	78828435	0.12328
27	oar3_OAR}	78829524	0.05823
27	oar3_OAR}	78837142	0.030586
27	oar3_OAR}	78848657	0.030586
27	oar3_OAR}	78851323	0.018042
27	oar3_OAR}	78851576	0.018042
27	oar3_OAR}	78856539	0.028681
27	oar3_OAR}	78870742	0.028681
27	oar3_OAR}	78870872	0.030586
27	oar3_OAR}	78881526	0.028681
27	oar3_OAR}	78884693	0.030586
27	oar3_OAR}	78891780	0.030586
27	oar3_OAR}	78912608	-0.02841
27	oar3_OAR}	78913826	0.020427
27	oar3_OAR}	78918385	-0.04195
27	oar3_OAR}	78925162	0.078766
27	oar3_OAR}	78925226	0.000821
27	oar3_OAR}	78939005	0.000821
27	oar3_OAR}	78941448	-0.04195
27	oar3_OAR}	78945772	0.089507
27	oar3_OAR}	78946782	0.043301
27	oar3_OAR}	78972547	-0.03117
27	oar3_OAR}	78980853	0.037475
27	oar3_OAR}	78987019	0.032223
27	oar3_OAR}	78989222	-0.02325
27	oar3_OAR}	78996139	0.034786
27	oar3_OAR}	79005356	0.037407
27	oar3_OAR}	79014602	0.039013
27	oar3_OAR}	79015694	-0.03191
27	oar3_OAR}	79017158	-0.03191
27	oar3_OAR}	79043277	-0.02154
27	oar3_OAR}	79045311	-0.02154
27	oar3_OAR}	79045791	-0.02482
27	oar3_OAR}	79061857	-0.02869
27	oar3_OAR}	79075581	-0.02154
27	oar3_OAR}	79080129	-0.01986
27	oar3_OAR}	79105248	-0.0156
27	oar3_OAR}	79111453	-0.02706
27	oar3_OAR}	79119054	-0.03543
27	oar3_OAR}	79120988	-0.03694
27	oar3_OAR}	79124313	-0.03383
27	oar3_OAR}	79134891	-0.02576
27	oar3_OAR}	79137159	-0.03694
27	oar3_OAR}	79137387	-0.02237
27	oar3_OAR}	79143944	-0.02576
27	oar3_OAR}	79168598	-0.03477
27	oar3_OAR}	79170290	-0.03079
27	oar3_OAR}	79170821	-0.02502

27	oar3_OAR⟩	79176624	-0.03946
27	oar3_OAR⟩	79178127	-0.00867
27	oar3_OAR⟩	79181246	-0.02502
27	oar3_OAR⟩	79182585	-0.03946
27	oar3_OAR⟩	79187827	-0.03116
27	oar3_OAR⟩	79189023	0.004924
27	oar3_OAR⟩	79198733	0.000889
27	oar3_OAR⟩	79200509	-0.00184
27	oar3_OAR⟩	79200819	-0.00184
27	oar3_OAR⟩	79205858	-0.00353
27	oar3_OAR⟩	79206282	0.00916
27	oar3_OAR⟩	79210042	0.021396
27	oar3_OAR⟩	79218584	0.042308
27	oar3_OAR⟩	79219017	0.081485
27	oar3_OAR⟩	79226624	0.016521
27	oar3_OAR⟩	79231269	0.023589
27	oar3_OAR⟩	79231456	0.023589
27	oar3_OAR⟩	79233017	0.044645
27	oar3_OAR⟩	79239980	-0.03148
27	oar3_OAR⟩	79247568	-0.01356
27	oar3_OAR⟩	79247720	-0.02209
27	oar3_OAR⟩	79254142	-0.02209
27	oar3_OAR⟩	79257983	-0.01356
27	oar3_OAR⟩	79261774	0.008796
27	oar3_OAR⟩	79261967	-0.01423
27	oar3_OAR⟩	79277404	0.011643
27	oar3_OAR⟩	79282962	0.020179
27	oar3_OAR⟩	79287073	0.018119
27	oar3_OAR⟩	79304091	0.039953
27	oar3_OAR⟩	79317408	0.019569
27	oar3_OAR⟩	79318040	-0.00165
27	oar3_OAR⟩	79327935	0.057354
27	oar3_OAR⟩	79328626	0.057354
27	oar3_OAR⟩	79332896	-0.01956
27	oar3_OAR⟩	79339936	0.081472
27	oar3_OAR⟩	79341129	0.047721
27	oar3_OAR⟩	79352858	0.069502
27	oar3_OAR⟩	79353018	0.047721
27	oar3_OAR⟩	79357204	0.051313
27	oar3_OAR⟩	79363886	0.040147
27	oar3_OAR⟩	79364580	0.040147
27	oar3_OAR⟩	79372094	-0.0049
27	oar3_OAR⟩	79380918	-0.00626
27	oar3_OAR⟩	79382409	0.006417
27	oar3_OAR⟩	79386209	0.038304
27	oar3_OAR⟩	79394340	-0.03527
27	oar3_OAR⟩	79397018	0.061274
27	oar3_OAR⟩	79407705	0.026732
27	oar3_OAR⟩	79409154	0.026732
27	oar3_OAR⟩	79423242	0.026732
27	oar3_OAR⟩	79425515	0.026732
27	oar3_OAR⟩	79442683	0.007188
27	oar3_OAR⟩	79443462	0.007188
27	oar3_OAR⟩	79447883	-0.02517

27	oar3_OAR>	79459946	0.026732
27	oar3_OAR>	79461020	0.026732
27	oar3_OAR>	79462505	-0.02316
27	oar3_OAR>	79488198	-0.0139
27	oar3_OAR>	79499815	-0.01503
27	oar3_OAR>	79514589	0.089029
27	oar3_OAR>	79519506	0.015142
27	oar3_OAR>	79526521	0.027041
27	oar3_OAR>	79527393	0.005933
27	oar3_OAR>	79534474	-0.0203
27	oar3_OAR>	79546372	-0.00776
27	oar3_OAR>	79546575	0.027041
27	oar3_OAR>	79546791	-0.00776
27	oar3_OAR>	79547164	-0.01215
27	oar3_OAR>	79560035	0.027041
27	oar3_OAR>	79566456	0.02664
27	oar3_OAR>	79567561	0.02664
27	oar3_OAR>	79567683	0.027041
27	oar3_OAR>	79570645	0.000729
27	oar3_OAR>	79574686	0.078333
27	oar3_OAR>	79580299	0.013551
27	oar3_OAR>	79580538	0.052546
27	oar3_OAR>	79582387	-0.00188
27	oar3_OAR>	79582561	-0.00188
27	oar3_OAR>	79590713	0.016046
27	oar3_OAR>	79591082	0.007039
27	oar3_OAR>	79594012	-0.03144
27	oar3_OAR>	79596972	0.044724
27	oar3_OAR>	79610439	-0.02724
27	oar3_OAR>	79610492	0.011849
27	oar3_OAR>	79614594	-0.02724
27	oar3_OAR>	79616008	-0.01406
27	oar3_OAR>	79619557	-0.00792
27	oar3_OAR>	79620480	-0.01496
27	oar3_OAR>	79621570	-0.01762
27	oar3_OAR>	79624767	-0.01762
27	oar3_OAR>	79634505	0.016306
27	oar3_OAR>	79635904	-0.02501
27	oar3_OAR>	79636040	-0.00204
27	oar3_OAR>	79643367	-0.02492
27	oar3_OAR>	79645038	-0.01847
27	oar3_OAR>	79646504	-0.02078
27	oar3_OAR>	79648640	-0.02797
27	oar3_OAR>	79652247	0.023321
27	oar3_OAR>	79655385	0.004085
27	oar3_OAR>	79659898	0.038084
27	oar3_OAR>	79660496	-0.03513
27	oar3_OAR>	79665857	-0.02174
27	oar3_OAR>	79666165	-0.00327
27	oar3_OAR>	79668488	0.02553
27	oar3_OAR>	79668546	0.026851
27	oar3_OAR>	79673732	0.01236
27	oar3_OAR>	79681828	0.02553
27	oar3_OAR>	79681928	0.008813

27	oar3_OAR〉	79690450	0.01236
27	oar3_OAR〉	79691729	0.017648
27	oar3_OAR〉	79692962	-0.01152
27	oar3_OAR〉	79695646	-0.02054
27	oar3_OAR〉	79699438	-0.00768
27	oar3_OAR〉	79700747	-0.00937
27	oar3_OAR〉	79709442	0.007124
27	oar3_OAR〉	79713472	0.005498
27	oar3_OAR〉	79719060	-0.03326
27	oar3_OAR〉	79719150	0.012422
27	oar3_OAR〉	79723507	0.031465
27	oar3_OAR〉	79726426	-0.01998
27	oar3_OAR〉	79726673	0.075797
27	oar3_OAR〉	79745770	0.040066
27	oar3_OAR〉	79752893	0.066477
27	oar3_OAR〉	79753098	0.069986
27	oar3_OAR〉	79753266	-0.02374
27	oar3_OAR〉	79758303	-0.00956
27	oar3_OAR〉	79762074	-0.00956
27	oar3_OAR〉	79762412	-0.01793
27	oar3_OAR〉	79765105	0.022348
27	oar3_OAR〉	79769999	0.011328
27	oar3_OAR〉	79770477	-0.01452
27	oar3_OAR〉	79778575	-0.0234
27	oar3_OAR〉	79779477	5.87E-06
27	oar3_OAR〉	79784425	0.03329
27	oar3_OAR〉	79785380	-0.01545
27	oar3_OAR〉	79787657	-0.0003
27	oar3_OAR〉	79801800	-0.01459
27	oar3_OAR〉	79807021	0.051316
27	oar3_OAR〉	79807237	0.0204
27	oar3_OAR〉	79816954	0.001955
27	oar3_OAR〉	79829766	0.001955
27	oar3_OAR〉	79831703	0.099203
27	oar3_OAR〉	79833389	-0.01633
27	oar3_OAR〉	79835741	-0.03504
27	oar3_OAR〉	79841555	0.221886
27	oar3_OAR〉	79842693	0.200776
27	oar3_OAR〉	79843276	0.156079
27	oar3_OAR〉	79843531	0.205078
27	oar3_OAR〉	79847612	0.301985
27	oar3_OAR〉	79857096	0.250867
27	oar3_OAR〉	79858302	0.010074
27	oar3_OAR〉	79860253	0.082423
27	oar3_OAR〉	79864961	0.250867
27	oar3_OAR〉	79866885	0.250867
27	oar3_OAR〉	79874981	0.248543
27	oar3_OAR〉	79886150	0.279983
27	oar3_OAR〉	79892727	0.015579
27	oar3_OAR〉	79894093	0.279983
27	oar3_OAR〉	79895089	-0.00578
27	oar3_OAR〉	79896259	-0.00578
27	oar3_OAR〉	79905417	-0.02237
27	oar3_OAR〉	79905644	0.095379

27	oar3_OAR›	79908738	-0.0047
27	oar3_OAR›	79914271	0.007009
27	oar3_OAR›	79915496	0.208114
27	oar3_OAR›	79917916	0.007039
27	oar3_OAR›	79925106	0.096723
27	oar3_OAR›	79933247	0.082423
27	oar3_OAR›	79935080	-0.01767
27	oar3_OAR›	79975855	-0.0068
27	oar3_OAR›	79977941	0.080451
27	oar3_OAR›	79986057	0.080451
27	oar3_OAR›	79986768	-0.01894
27	oar3_OAR›	79994499	0.097549
27	oar3_OAR›	79997885	0.021715
27	oar3_OAR›	79998683	-0.03248
27	oar3_OAR›	80002310	0.107702
27	oar3_OAR›	80017680	0.22349
27	oar3_OAR›	80047822	0.100702
27	oar3_OAR›	80058911	-0.02449
27	oar3_OAR›	80066006	-0.01766
27	oar3_OAR›	80072099	0.154301
27	oar3_OAR›	80078908	0.059665
27	oar3_OAR›	80082245	0.088779
27	oar3_OAR›	80086130	0.074703
27	oar3_OAR›	80090226	0.069429
27	oar3_OAR›	80093910	0.017206
27	oar3_OAR›	80094207	0.017206
27	oar3_OAR›	80096532	0.03474
27	oar3_OAR›	80100775	0.021533
27	oar3_OAR›	80101904	-0.02797
27	oar3_OAR›	80108809	-0.03606
27	oar3_OAR›	80112691	0.021533
27	oar3_OAR›	80124297	-0.02162
27	oar3_OAR›	80129817	-0.01193
27	oar3_OAR›	80142280	-0.01193
27	oar3_OAR›	80152123	-0.02151
27	oar3_OAR›	80154114	-0.01327
27	oar3_OAR›	80158253	-0.02371
27	oar3_OAR›	80160464	-0.02899
27	oar3_OAR›	80163155	-0.01062
27	oar3_OAR›	80166885	-0.01062
27	oar3_OAR›	80169748	-0.02899
27	oar3_OAR›	80175599	-0.02834
27	oar3_OAR›	80177370	-0.02834
27	oar3_OAR›	80179193	-0.03248
27	oar3_OAR›	80187083	-0.01789
27	oar3_OAR›	80187540	-0.01789
27	oar3_OAR›	80187817	-0.01789
27	oar3_OAR›	80196254	-0.0245
27	oar3_OAR›	80197498	-0.0245
27	oar3_OAR›	80206815	-0.01847
27	oar3_OAR›	80206975	-0.01756
27	oar3_OAR›	80213385	0.046527
27	oar3_OAR›	80213533	-0.03414
27	oar3_OAR›	80213904	-0.01687

27	oar3_OAR›	80218535	0.046527
27	oar3_OAR›	80225192	0.024821
27	oar3_OAR›	80230184	-0.02647
27	oar3_OAR›	80232529	-0.00213
27	oar3_OAR›	80238437	-0.0163
27	oar3_OAR›	80242059	-0.00711
27	oar3_OAR›	80246716	-0.01059
27	oar3_OAR›	80247727	-0.01367
27	oar3_OAR›	80250644	-0.01059
27	oar3_OAR›	80250833	-0.01059
27	oar3_OAR›	80259575	-0.02562
27	oar3_OAR›	80260884	-0.0163
27	oar3_OAR›	80266126	-0.00121
27	oar3_OAR›	80269855	-0.01367
27	oar3_OAR›	80275867	-0.02209
27	oar3_OAR›	80275950	-0.04065
27	oar3_OAR›	80280227	-0.02793
27	oar3_OAR›	80281062	-0.02782
27	oar3_OAR›	80283155	-0.0298
27	oar3_OAR›	80288575	-0.01224
27	oar3_OAR›	80289110	0.014031
27	oar3_OAR›	80290311	0.015013
27	oar3_OAR›	80294000	-0.02445
27	oar3_OAR›	80296289	0.035317
27	oar3_OAR›	80299031	-0.00015
27	oar3_OAR›	80304582	0.041197
27	oar3_OAR›	80305209	0.010176
27	oar3_OAR›	80309419	0.043995
27	oar3_OAR›	80318722	-0.02628
27	oar3_OAR›	80323835	0.049327
27	oar3_OAR›	80328916	0.016716
27	oar3_OAR›	80336962	-0.01298
27	oar3_OAR›	80339310	-0.01298
27	oar3_OAR›	80340498	0.058663
27	oar3_OAR›	80341107	0.016819
27	oar3_OAR›	80345449	0.028467
27	oar3_OAR›	80347279	0.043997
27	oar3_OAR›	80350025	0.121695
27	oar3_OAR›	80356089	0.006442
27	oar3_OAR›	80359010	0.019861
27	oar3_OAR›	80359666	0.005053
27	oar3_OAR›	80373573	0.082222
27	oar3_OAR›	80376965	0.016379
27	oar3_OAR›	80378162	0.016379
27	oar3_OAR›	80379949	0.044724
27	oar3_OAR›	80380358	0.019861
27	oar3_OAR›	80391825	-0.00606
27	oar3_OAR›	80392223	0.034065
27	oar3_OAR›	80392277	0.046263
27	oar3_OAR›	80397011	-0.02292
27	oar3_OAR›	80404145	0.04522
27	oar3_OAR›	80404354	-0.02292
27	oar3_OAR›	80406708	-0.00606
27	oar3_OAR›	80410672	0.033645

27	oar3_OAR›	80415216	-0.02102
27	oar3_OAR›	80420424	-0.00043
27	oar3_OAR›	80424338	-0.02102
27	oar3_OAR›	80426717	-0.01404
27	oar3_OAR›	80427513	-0.02102
27	oar3_OAR›	80434189	-0.00841
27	oar3_OAR›	80437731	-0.0218
27	oar3_OAR›	80455444	0.041434
27	oar3_OAR›	80456285	0.007039
27	oar3_OAR›	80458689	0.007039
27	oar3_OAR›	80463843	0.051052
27	oar3_OAR›	80467922	0.10636
27	oar3_OAR›	80468828	0.235239
27	oar3_OAR›	80471721	0.186577
27	oar3_OAR›	80474018	0.171087
27	oar3_OAR›	80481890	0.186577
27	oar3_OAR›	80484491	0.051052
27	oar3_OAR›	80484992	0.001452
27	oar3_OAR›	80497766	0.010633
27	oar3_OAR›	80508870	NA
27	oar3_OAR›	80508988	0.074803
27	oar3_OAR›	80514026	0.105396
27	oar3_OAR›	80518016	0.016306
27	oar3_OAR›	80528213	0.016306
27	oar3_OAR›	80531318	0.074803
27	oar3_OAR›	80535078	NA
27	oar3_OAR›	80541473	0.080171
27	oar3_OAR›	80542486	0.074803
27	oar3_OAR›	80542493	0.039198
27	oar3_OAR›	80543647	0.117003
27	oar3_OAR›	80547735	0.074803
27	oar3_OAR›	80554335	-0.01778
27	oar3_OAR›	80563012	0.01863
27	oar3_OAR›	80568600	0.115174
27	oar3_OAR›	80571007	0.000889
27	oar3_OAR›	80573949	0.003305
27	oar3_OAR›	80580725	NA
27	oar3_OAR›	80583679	0.115174
27	oar3_OAR›	80593558	0.056553
27	oar3_OAR›	80593838	0.007181
27	oar3_OAR›	80599783	-0.0065
27	oar3_OAR›	80601464	0.056553
27	oar3_OAR›	80604045	0.070422
27	oar3_OAR›	80607444	0.152233
27	oar3_OAR›	80610523	-0.00673
27	oar3_OAR›	80614455	-0.03142
27	oar3_OAR›	80615810	-0.00673
27	oar3_OAR›	80617612	-0.00673
27	oar3_OAR›	80623612	0.048284
27	oar3_OAR›	80630720	0.010014
27	oar3_OAR›	80630983	0.043649
27	oar3_OAR›	80634527	0.003447
27	oar3_OAR›	80634973	0.058681
27	oar3_OAR›	80660737	-0.0018

27	oar3_OAR›	80669082	0.023872
27	oar3_OAR›	80669174	0.016042
27	oar3_OAR›	80679240	-0.0042
27	oar3_OAR›	80679772	0.023872
27	oar3_OAR›	80682924	0.021891
27	oar3_OAR›	80694949	-0.01334
27	oar3_OAR›	80695868	0.021891
27	oar3_OAR›	80701373	-0.00287
27	oar3_OAR›	80706620	-0.02191
27	oar3_OAR›	80707636	0.109122
27	oar3_OAR›	80709828	########
27	oar3_OAR›	80712785	-0.00019
27	oar3_OAR›	80720845	-0.00037
27	oar3_OAR›	80726133	-0.0018
27	oar3_OAR›	80734544	0.064547
27	oar3_OAR›	80737192	0.025869
27	oar3_OAR›	80742341	-0.03035
27	oar3_OAR›	80753619	-0.01452
27	oar3_OAR›	80754229	-0.01452
27	oar3_OAR›	80754588	0.030937
27	oar3_OAR›	80776096	0.097086
27	oar3_OAR›	80819139	0.001984
27	oar3_OAR›	80830597	0.001984
27	oar3_OAR›	80842114	-0.02588
27	oar3_OAR›	80845421	0.05259
27	oar3_OAR›	80861232	0.019679
27	oar3_OAR›	80864937	0.023806
27	oar3_OAR›	80869946	0.018082
27	oar3_OAR›	80870257	0.011893
27	oar3_OAR›	80870822	-0.01338
27	oar3_OAR›	80876520	0.003446
27	oar3_OAR›	80878360	-0.01857
27	oar3_OAR›	80880899	-0.02038
27	oar3_OAR›	80891720	-0.00641
27	oar3_OAR›	80894034	-0.03268
27	oar3_OAR›	80900220	0.008296
27	oar3_OAR›	80902375	0.003253
27	oar3_OAR›	80902515	0.028226
27	oar3_OAR›	80907877	0.008296
27	oar3_OAR›	80911155	-0.01283
27	oar3_OAR›	80912836	0.044724
27	oar3_OAR›	80917184	-0.01547
27	oar3_OAR›	80919462	-0.02709
27	oar3_OAR›	80932397	-0.01404
27	oar3_OAR›	80940076	0.018955
27	oar3_OAR›	80940818	0.007059
27	oar3_OAR›	80942480	-0.01404
27	oar3_OAR›	80947371	0.000889
27	oar3_OAR›	80950921	0.018955
27	oar3_OAR›	80965975	0.011704
27	oar3_OAR›	80969273	0.028277
27	oar3_OAR›	80969609	-0.03414
27	oar3_OAR›	80974238	-0.03295
27	oar3_OAR›	80976638	-0.03295

27	oar3_OAR›	80983034	0.080493
27	oar3_OAR›	80991465	0.02864
27	oar3_OAR›	80998317	0.043609
27	oar3_OAR›	81024333	0.02374
27	oar3_OAR›	81024372	0.044633
27	oar3_OAR›	81033763	0.044633
27	oar3_OAR›	81036002	-0.01906
27	oar3_OAR›	81051991	0.077381
27	oar3_OAR›	81058692	0.016062
27	oar3_OAR›	81059200	0.016062
27	oar3_OAR›	81063512	-0.01315
27	oar3_OAR›	81064291	-0.02425
27	oar3_OAR›	81071028	-0.01655
27	oar3_OAR›	81076629	0.022977
27	oar3_OAR›	81086589	0.001262
27	oar3_OAR›	81139266	0.007045
27	oar3_OAR›	81186554	-0.01747
27	oar3_OAR›	81203613	0.040802
27	oar3_OAR›	81222701	0.029717
27	oar3_OAR›	81223754	-0.00082
27	oar3_OAR›	81258928	0.030671
27	oar3_OAR›	81260378	0.123495
27	oar3_OAR›	81265407	0.082222
27	oar3_OAR›	81270394	0.082222
27	oar3_OAR›	81292504	0.095373
27	oar3_OAR›	81294013	0.061098
27	oar3_OAR›	81299279	0.039844
27	oar3_OAR›	81300384	0.039844
27	oar3_OAR›	81305590	0.007181
27	oar3_OAR›	81308343	0.049128
27	oar3_OAR›	81314190	0.033768
27	oar3_OAR›	81314265	-0.00898
27	oar3_OAR›	81317303	0.033768
27	oar3_OAR›	81319305	0.033768
27	oar3_OAR›	81322110	0.046215
27	oar3_OAR›	81322259	0.033768
27	oar3_OAR›	81329093	0.093962
27	oar3_OAR›	81332394	0.024879
27	oar3_OAR›	81347502	0.051674
27	oar3_OAR›	81347540	0.126528
27	oar3_OAR›	81347613	0.126528
27	oar3_OAR›	81348898	0.126528
27	oar3_OAR›	81352937	-0.01082
27	oar3_OAR›	81358779	0.026923
27	oar3_OAR›	81369500	0.017354
27	oar3_OAR›	81380057	0.028864
27	oar3_OAR›	81380104	0.109621
27	oar3_OAR›	81382795	-0.01377
27	oar3_OAR›	81398290	-0.00574
27	oar3_OAR›	81403972	-0.01891
27	oar3_OAR›	81405106	0.045085
27	oar3_OAR›	81409065	0.03648
27	oar3_OAR›	81409744	0.057173
27	oar3_OAR›	81421671	0.002472

27	oar3_OAR›	81426574	-0.01803
27	oar3_OAR›	81431945	-0.01904
27	oar3_OAR›	81435570	-0.00404
27	oar3_OAR›	81443923	-0.01634
27	oar3_OAR›	81448008	0.011887
27	oar3_OAR›	81448083	0.011887
27	oar3_OAR›	81454937	-0.02835
27	oar3_OAR›	81460843	-0.00653
27	oar3_OAR›	81464401	0.028467
27	oar3_OAR›	81471135	-0.02529
27	oar3_OAR›	81472629	-0.00189
27	oar3_OAR›	81473498	0.028467
27	oar3_OAR›	81483061	0.028467
27	oar3_OAR›	81484694	-0.00189
27	oar3_OAR›	81484761	-0.00189
27	oar3_OAR›	81494283	-0.00262
27	oar3_OAR›	81495532	-0.01779
27	oar3_OAR›	81498845	0.013973
27	oar3_OAR›	81499654	0.013973
27	oar3_OAR›	81502237	-0.00262
27	oar3_OAR›	81510745	-0.01581
27	oar3_OAR›	81515963	0.038353
27	oar3_OAR›	81535056	-0.01737
27	oar3_OAR›	81536730	0.053574
27	oar3_OAR›	81537935	0.007511
27	oar3_OAR›	81546143	-0.0285
27	oar3_OAR›	81547100	0.003012
27	oar3_OAR›	81547978	0.035332
27	oar3_OAR›	81562529	-0.01822
27	oar3_OAR›	81563800	0.006089
27	oar3_OAR›	81568725	-0.03314
27	oar3_OAR›	81571053	-0.01437
27	oar3_OAR›	81597802	-0.00936
27	oar3_OAR›	81597907	NA
27	oar3_OAR›	81598312	-0.00936
27	oar3_OAR›	81606660	-0.01939
27	oar3_OAR›	81607928	-0.01737
27	oar3_OAR›	81611192	-0.0137
27	oar3_OAR›	81614781	-0.03075
27	oar3_OAR›	81615366	-0.03441
27	oar3_OAR›	81616142	-0.0154
27	oar3_OAR›	81621690	-0.03679
27	oar3_OAR›	81630442	-0.027
27	oar3_OAR›	81635341	-0.02105
27	oar3_OAR›	81635382	-0.023
27	oar3_OAR›	81637166	-0.027
27	oar3_OAR›	81640026	0.004072
27	oar3_OAR›	81645638	-0.02362
27	oar3_OAR›	81646493	0.003446
27	oar3_OAR›	81652110	-0.02926
27	oar3_OAR›	81656306	0.002815
27	oar3_OAR›	81657351	0.006708
27	oar3_OAR›	81663622	0.055974
27	oar3_OAR›	81669898	0.020529

27	oar3_OAR›	81674955	-0.03542
27	oar3_OAR›	81681410	-0.03324
27	oar3_OAR›	81685749	0.036767
27	oar3_OAR›	81686959	0.036767
27	oar3_OAR›	81693079	0.033357
27	oar3_OAR›	81698333	-0.03091
27	oar3_OAR›	81698914	0.031886
27	oar3_OAR›	81705043	0.012941
27	oar3_OAR›	81710512	0.082095
27	oar3_OAR›	81712420	0.00877
27	oar3_OAR›	81718578	0.060591
27	oar3_OAR›	81723413	0.000889
27	oar3_OAR›	81726897	0.026653
27	oar3_OAR›	81733174	0.082095
27	oar3_OAR›	81738521	0.001778
27	oar3_OAR›	81738832	0.060591
27	oar3_OAR›	81743653	0.000889
27	oar3_OAR›	81744205	0.043722
27	oar3_OAR›	81750262	-0.00925
27	oar3_OAR›	81753763	0.012198
27	oar3_OAR›	81756032	0.054933
27	oar3_OAR›	81756742	NA
27	oar3_OAR›	81757215	NA
27	oar3_OAR›	81760993	0.055942
27	oar3_OAR›	81764385	0.040047
27	oar3_OAR›	81766332	0.033114
27	oar3_OAR›	81766759	0.048016
27	oar3_OAR›	81766902	0.051801
27	oar3_OAR›	81772026	0.033114
27	oar3_OAR›	81776526	-0.02044
27	oar3_OAR›	81786612	-0.03702
27	oar3_OAR›	81789848	0.034592
27	oar3_OAR›	81790067	-0.01484
27	oar3_OAR›	81791952	0.010874
27	oar3_OAR›	81797320	-0.00075
27	oar3_OAR›	81798615	-0.00128
27	oar3_OAR›	81798662	-0.03144
27	oar3_OAR›	81801936	-0.02211
27	oar3_OAR›	81803761	0.028805
27	oar3_OAR›	81809038	0.006216
27	oar3_OAR›	81810902	0.006216
27	oar3_OAR›	81814643	-0.02199
27	oar3_OAR›	81821059	-0.0264
27	oar3_OAR›	81836592	-0.03175
27	oar3_OAR›	81837819	-0.03175
27	oar3_OAR›	81838584	-0.02695
27	oar3_OAR›	81855983	-0.01262
27	oar3_OAR›	81862014	-0.02695
27	oar3_OAR›	81862680	-0.02695
27	oar3_OAR›	81865560	-0.01986
27	oar3_OAR›	81868570	0.038594
27	oar3_OAR›	81872054	0.047633
27	oar3_OAR›	81879485	-0.01986
27	oar3_OAR›	81880462	0.001866

27	oar3_OAR›	81880816	-0.02049
27	oar3_OAR›	81886325	-0.02049
27	oar3_OAR›	81893562	-0.01775
27	oar3_OAR›	81896015	-0.02557
27	oar3_OAR›	81896538	0.010341
27	oar3_OAR›	81896757	0.010341
27	oar3_OAR›	81901751	-0.0236
27	oar3_OAR›	81908343	0.116098
27	oar3_OAR›	81910312	0.050318
27	oar3_OAR›	81910545	0.050318
27	oar3_OAR›	81917151	0.138797
27	oar3_OAR›	81918647	0.041401
27	oar3_OAR›	81921499	-0.01013
27	oar3_OAR›	81923138	-0.00451
27	oar3_OAR›	81925391	0.053463
27	oar3_OAR›	81931373	0.041221
27	oar3_OAR›	81936113	0.001685
27	oar3_OAR›	81936586	-0.00455
27	oar3_OAR›	81937408	-0.03135
27	oar3_OAR›	81944731	-0.01791
27	oar3_OAR›	81945012	0.023613
27	oar3_OAR›	81946622	-0.02522
27	oar3_OAR›	81949703	-0.00122
27	oar3_OAR›	81950789	-0.02371
27	oar3_OAR›	81957346	0.086371
27	oar3_OAR›	81961735	0.041221
27	oar3_OAR›	81965134	-0.01655
27	oar3_OAR›	81966011	0.028326
27	oar3_OAR›	81967585	0.02374
27	oar3_OAR›	81972534	0.028326
27	oar3_OAR›	81981103	0.044472
27	oar3_OAR›	81981310	0.083755
27	oar3_OAR›	81981460	0.083755
27	oar3_OAR›	81986544	-0.01091
27	oar3_OAR›	81992796	0.036764
27	oar3_OAR›	81994027	0.011067
27	oar3_OAR›	81995741	0.007497
27	oar3_OAR›	81997947	-0.00094
27	oar3_OAR›	82000824	0.011067
27	oar3_OAR›	82007228	0.176495
27	oar3_OAR›	82010041	0.041221
27	oar3_OAR›	82010783	-0.00945
27	oar3_OAR›	82016325	-0.00362
27	oar3_OAR›	82017427	8.73E-05
27	oar3_OAR›	82021803	-0.02721
27	oar3_OAR›	82022831	-0.02721
27	oar3_OAR›	82022963	0.007039
27	oar3_OAR›	82028585	-0.01127
27	oar3_OAR›	82033571	0.069468
27	oar3_OAR›	82034717	0.037433
27	oar3_OAR›	82037192	0.109621
27	oar3_OAR›	82041314	0.004284
27	oar3_OAR›	82041726	0.018287
27	oar3_OAR›	82049033	0.061943

27	oar3_OAR›	82050193	0.061943
27	oar3_OAR›	82055510	0.027982
27	oar3_OAR›	82055955	0.061943
27	oar3_OAR›	82057510	-0.00717
27	oar3_OAR›	82064609	-0.02764
27	oar3_OAR›	82066434	0.09673
27	oar3_OAR›	82066479	-0.02764
27	oar3_OAR›	82066847	-0.02764
27	oar3_OAR›	82074652	0.027982
27	oar3_OAR›	82085847	-0.01406
27	oar3_OAR›	82085928	-0.00606
27	oar3_OAR›	82090956	-0.02014
27	oar3_OAR›	82096652	-0.03114
27	oar3_OAR›	82099511	0.017962
27	oar3_OAR›	82101388	-0.02819
27	oar3_OAR›	82104034	0.051662
27	oar3_OAR›	82114083	-0.02064
27	oar3_OAR›	82116428	-0.0324
27	oar3_OAR›	82117370	-0.0324
27	oar3_OAR›	82118856	0.004003
27	oar3_OAR›	82123772	-0.0255
27	oar3_OAR›	82127672	-0.03059
27	oar3_OAR›	82135293	-0.01739
27	oar3_OAR›	82136068	-0.03059
27	oar3_OAR›	82137121	NA
27	oar3_OAR›	82137452	NA
27	oar3_OAR›	82143724	NA
27	oar3_OAR›	82148150	-0.01767
27	oar3_OAR›	82148876	0.00219
27	oar3_OAR›	82155343	-0.02864
27	oar3_OAR›	82159825	-0.00585
27	oar3_OAR›	82163333	-0.00427
27	oar3_OAR›	82166052	0.179501
27	oar3_OAR›	82170004	0.137874
27	oar3_OAR›	82176331	0.150786
27	oar3_OAR›	82176829	-0.00462
27	oar3_OAR›	82180411	NA
27	oar3_OAR›	82180480	0.179501
27	oar3_OAR›	82181821	-0.00462
27	oar3_OAR›	82191198	-0.00462
27	oar3_OAR›	82194706	0.137023
27	oar3_OAR›	82197499	0.123999
27	oar3_OAR›	82202755	-0.00422
27	oar3_OAR›	82205083	0.141517
27	oar3_OAR›	82206344	0.070714
27	oar3_OAR›	82210810	-0.00422
27	oar3_OAR›	82212135	0.046647
27	oar3_OAR›	82215768	0.017807
27	oar3_OAR›	82215823	0.038628
27	oar3_OAR›	82222860	-0.02891
27	oar3_OAR›	82226717	0.024184
27	oar3_OAR›	82231228	-0.00422
27	oar3_OAR›	82233313	-0.0233
27	oar3_OAR›	82239675	-0.01227

27	oar3_OAR›	82242320	0.092322
27	oar3_OAR›	82242462	NA
27	oar3_OAR›	82256210	NA
27	oar3_OAR›	82258574	0.023487
27	oar3_OAR›	82258809	0.020608
27	oar3_OAR›	82264281	0.053273
27	oar3_OAR›	82266163	0.043755
27	oar3_OAR›	82266685	0.018326
27	oar3_OAR›	82270597	-0.03266
27	oar3_OAR›	82271303	-0.03266
27	oar3_OAR›	82271423	0.018326
27	oar3_OAR›	82275762	0.02829
27	oar3_OAR›	82278040	NA
27	oar3_OAR›	82291267	0.032223
27	oar3_OAR›	82296668	-0.03287
27	oar3_OAR›	82297832	0.020654
27	oar3_OAR›	82304740	-0.02926
27	oar3_OAR›	82310242	-0.02744
27	oar3_OAR›	82322822	-0.02468
27	oar3_OAR›	82325758	0.061323
27	oar3_OAR›	82326590	0.061323
27	oar3_OAR›	82336385	-0.03324
27	oar3_OAR›	82336903	0.028681
27	oar3_OAR›	82350318	0.028681
27	oar3_OAR›	82368235	-0.02294
27	oar3_OAR›	82389498	0.031617
27	oar3_OAR›	82392119	0.031617
27	oar3_OAR›	82394388	0.036779
27	oar3_OAR›	82405451	0.055262
27	oar3_OAR›	82407114	0.053114
27	oar3_OAR›	82410885	-0.01617
27	oar3_OAR›	82411486	0.020874
27	oar3_OAR›	82423988	0.004689
27	oar3_OAR›	82437340	-0.01736
27	oar3_OAR›	82446488	-0.00535
27	oar3_OAR›	82452728	-0.02527
27	oar3_OAR›	82459192	0.005641
27	oar3_OAR›	82464037	0.039481
27	oar3_OAR›	82467333	0.016615
27	oar3_OAR›	82469309	-0.02061
27	oar3_OAR›	82474496	-0.02555
27	oar3_OAR›	82478786	0.00077
27	oar3_OAR›	82479597	-0.03139
27	oar3_OAR›	82486880	0.015989
27	oar3_OAR›	82499839	-0.03057
27	oar3_OAR›	82500012	-0.02555
27	oar3_OAR›	82502739	-0.03072
27	oar3_OAR›	82506265	-0.03072
27	oar3_OAR›	82512530	-0.02555
27	oar3_OAR›	82512977	0.012017
27	oar3_OAR›	82512989	-0.02188
27	oar3_OAR›	82518059	-0.02748
27	oar3_OAR›	82518457	-0.02748
27	oar3_OAR›	82526687	0.044724

27	oar3_OAR〉	82531532	-0.00379
27	oar3_OAR〉	82535213	-0.03569
27	oar3_OAR〉	82535583	-0.03569
27	oar3_OAR〉	82542236	-0.03569
27	oar3_OAR〉	82558911	-0.02908
27	oar3_OAR〉	82574436	-0.02444
27	oar3_OAR〉	82589783	-0.01694
27	oar3_OAR〉	82598053	-0.02222
27	oar3_OAR〉	82617363	-0.00435
27	oar3_OAR〉	82618847	-0.02562
27	oar3_OAR〉	82621437	0.053987
27	oar3_OAR〉	82626490	0.083987
27	oar3_OAR〉	82640736	-0.01795
27	oar3_OAR〉	82642873	-0.00963
27	oar3_OAR〉	82656701	-0.00671
27	oar3_OAR〉	82662209	-0.00984
27	oar3_OAR〉	82675546	0.009975
27	oar3_OAR〉	82684598	0.020466
27	oar3_OAR〉	82685098	-0.03215
27	oar3_OAR〉	82690178	0.168935
27	oar3_OAR〉	82691785	-0.03215
27	oar3_OAR〉	82697417	0.001934
27	oar3_OAR〉	82703091	0.001934
27	oar3_OAR〉	82706906	0.055406
27	oar3_OAR〉	82712108	0.033602
27	oar3_OAR〉	82714122	0.097573
27	oar3_OAR〉	82723371	-0.0206
27	oar3_OAR〉	82728288	0.040528
27	oar3_OAR〉	82742433	0.076927
27	oar3_OAR〉	82745756	0.069915
27	oar3_OAR〉	82765470	0.081595
27	oar3_OAR〉	82766353	0.019997
27	oar3_OAR〉	82771863	0.04322
27	oar3_OAR〉	82773197	0.019997
27	oar3_OAR〉	82773712	0.04322
27	oar3_OAR〉	82783762	0.070572
27	oar3_OAR〉	82792656	-0.00601
27	oar3_OAR〉	82810340	0.075534
27	oar3_OAR〉	82812199	0.116401
27	oar3_OAR〉	82816024	0.065918
27	oar3_OAR〉	82824463	0.010985
27	oar3_OAR〉	82826216	0.116401
27	oar3_OAR〉	82836434	0.064242
27	oar3_OAR〉	82840105	0.068244
27	oar3_OAR〉	82841502	0.064242
27	oar3_OAR〉	82848537	0.028803
27	oar3_OAR〉	82848922	0.010985
27	oar3_OAR〉	82849426	0.010985
27	oar3_OAR〉	82857029	0.003335
27	oar3_OAR〉	82859753	0.109427
27	oar3_OAR〉	82862315	0.022635
27	oar3_OAR〉	82863339	0.053398
27	oar3_OAR〉	82863600	0.036634
27	oar3_OAR〉	82877800	0.044493

27	oar3_OAR›	82879584	0.00563
27	oar3_OAR›	82890085	0.032582
27	oar3_OAR›	82895452	0.032582
27	oar3_OAR›	82906608	0.016689
27	oar3_OAR›	82909771	0.035054
27	oar3_OAR›	82909875	0.066685
27	oar3_OAR›	82917031	0.066685
27	oar3_OAR›	82924209	0.009277
27	oar3_OAR›	82929289	0.102108
27	oar3_OAR›	82930252	-0.02699
27	oar3_OAR›	82935100	-0.03461
27	oar3_OAR›	82942548	0.021533
27	oar3_OAR›	82943769	0.025702
27	oar3_OAR›	82949175	0.012992
27	oar3_OAR›	82958668	0.102108
27	oar3_OAR›	82968098	0.173822
27	oar3_OAR›	82968172	0.1432
27	oar3_OAR›	82969664	-0.00597
27	oar3_OAR›	82975496	-0.01791
27	oar3_OAR›	82977585	-0.01874
27	oar3_OAR›	82979478	0.028035
27	oar3_OAR›	82979628	0.028035
27	oar3_OAR›	82985809	0.117829
27	oar3_OAR›	82991564	0.078045
27	oar3_OAR›	82994508	0.078045
27	oar3_OAR›	83012857	0.078045
27	oar3_OAR›	83013695	0.087331
27	oar3_OAR›	83020776	-0.03461
27	oar3_OAR›	83021153	0.078045
27	oar3_OAR›	83046580	-0.01058
27	oar3_OAR›	83047068	-0.03914
27	oar3_OAR›	83053504	-0.01655
27	oar3_OAR›	83061080	-0.00109
27	oar3_OAR›	83061221	-0.02582
27	oar3_OAR›	83061938	0.003079
27	oar3_OAR›	83075468	-0.01848
27	oar3_OAR›	83076895	0.003079
27	oar3_OAR›	83089443	-0.03284
27	oar3_OAR›	83092522	-0.02536
27	oar3_OAR›	83098957	-0.01789
27	oar3_OAR›	83099339	-0.00109
27	oar3_OAR›	83100133	-0.02941
27	oar3_OAR›	83104043	-0.01605
27	oar3_OAR›	83104702	-0.01069
27	oar3_OAR›	83109668	-0.01069
27	oar3_OAR›	83112600	-0.03284
27	oar3_OAR›	83113972	-0.02941
27	oar3_OAR›	83114299	0.031152
27	oar3_OAR›	83121615	-0.02941
27	oar3_OAR›	83125147	0.031152
27	oar3_OAR›	83125409	0.031152
27	oar3_OAR›	83132126	0.000889
27	oar3_OAR›	83138626	0.035405
27	oar3_OAR›	83139064	-0.02788

27	oar3_OAR›	83139960	-0.02818
27	oar3_OAR›	83149882	0.032319
27	oar3_OAR›	83150603	0.032319
27	oar3_OAR›	83177367	-0.00991
27	oar3_OAR›	83180939	-0.00991
27	oar3_OAR›	83183195	-0.00991
27	oar3_OAR›	83199847	-0.00713
27	oar3_OAR›	83200275	-0.00713
27	oar3_OAR›	83204960	-0.00713
27	oar3_OAR›	83210570	-0.01699
27	oar3_OAR›	83210949	-0.00713
27	oar3_OAR›	83211304	-0.02301
27	oar3_OAR›	83211588	-0.00713
27	oar3_OAR›	83216162	-0.02449
27	oar3_OAR›	83222546	-0.02301
27	oar3_OAR›	83236711	0.014822
27	oar3_OAR›	83237724	-0.00892
27	oar3_OAR›	83244055	0.014822
27	oar3_OAR›	83274898	-0.00043
27	oar3_OAR›	83275551	-0.00043
27	oar3_OAR›	83285649	0.00115
27	oar3_OAR›	83290414	-0.0214
27	oar3_OAR›	83292133	-0.01402
27	oar3_OAR›	83296171	-0.00043
27	oar3_OAR›	83297960	-0.00043
27	oar3_OAR›	83307164	0.00115
27	oar3_OAR›	83316518	-0.00307
27	oar3_OAR›	83326945	0.007039
27	oar3_OAR›	83333902	0.010241
27	oar3_OAR›	83336031	0.038636
27	oar3_OAR›	83340243	0.007039
27	oar3_OAR›	83341654	-0.00895
27	oar3_OAR›	83347283	-0.01279
27	oar3_OAR›	83348579	0.024422
27	oar3_OAR›	83352557	-0.02645
27	oar3_OAR›	83360729	-0.01496
27	oar3_OAR›	83368449	-0.00298
27	oar3_OAR›	83380851	-0.00439
27	oar3_OAR›	83400197	-0.01817
27	oar3_OAR›	83400727	0.007039
27	oar3_OAR›	83425876	0.007039
27	oar3_OAR›	83425962	-0.02287
27	oar3_OAR›	83431713	-0.02958
27	oar3_OAR›	83438156	-0.02958
27	oar3_OAR›	83439177	0.010227
27	oar3_OAR›	83461036	-0.02782
27	oar3_OAR›	83461064	0.073657
27	oar3_OAR›	83461376	-0.02782
27	oar3_OAR›	83461709	0.111174
27	oar3_OAR›	83476776	0.111174
27	oar3_OAR›	83476820	0.101806
27	oar3_OAR›	83477452	0.111174
27	oar3_OAR›	83484637	-0.02688
27	oar3_OAR›	83490440	0.111174

27	oar3_OAR›	83490953	-0.01891
27	oar3_OAR›	83491063	-0.02688
27	oar3_OAR›	83509947	-0.02703
27	oar3_OAR›	83514249	0.095498
27	oar3_OAR›	83521421	0.095498
27	oar3_OAR›	83526700	-0.00574
27	oar3_OAR›	83528330	0.143502
27	oar3_OAR›	83543305	-0.00574
27	oar3_OAR›	83556249	0.077893
27	oar3_OAR›	83561727	-0.02531
27	oar3_OAR›	83565576	0.083036
27	oar3_OAR›	83566689	0.00481
27	oar3_OAR›	83576342	0.010077
27	oar3_OAR›	83581415	0.046131
27	oar3_OAR›	83581818	0.083036
27	oar3_OAR›	83589994	0.024937
27	oar3_OAR›	83593343	0.07759
27	oar3_OAR›	83599945	0.07759
27	oar3_OAR›	83600368	-0.00456
27	oar3_OAR›	83601417	0.048614
27	oar3_OAR›	83601524	-0.01456
27	oar3_OAR›	83608504	0.024014
27	oar3_OAR›	83611240	-0.00456
27	oar3_OAR›	83614063	-0.00456
27	oar3_OAR›	83624078	-0.01279
27	oar3_OAR›	83624978	-0.01279
27	oar3_OAR›	83634996	0.003053
27	oar3_OAR›	83635026	0.003053
27	oar3_OAR›	83636836	0.004316
27	oar3_OAR›	83639980	0.024014
27	oar3_OAR›	83642742	-0.01738
27	oar3_OAR›	83649100	-0.01456
27	oar3_OAR›	83654076	-0.01735
27	oar3_OAR›	83654226	-0.00456
27	oar3_OAR›	83661183	0.003053
27	oar3_OAR›	83661631	0.00019
27	oar3_OAR›	83662299	0.011798
27	oar3_OAR›	83674001	0.003053
27	oar3_OAR›	83674064	0.003053
27	oar3_OAR›	83682363	-0.01528
27	oar3_OAR›	83686965	0.039609
27	oar3_OAR›	83687767	0.039609
27	oar3_OAR›	83690561	0.007863
27	oar3_OAR›	83694472	-0.02437
27	oar3_OAR›	83707087	-0.01528
27	oar3_OAR›	83707160	-0.01528
27	oar3_OAR›	83715005	0.024342
27	oar3_OAR›	83716669	-0.01528
27	oar3_OAR›	83720619	-0.01528
27	oar3_OAR›	83721577	-0.02437
27	oar3_OAR›	83725844	0.000889
27	oar3_OAR›	83729008	-0.01571
27	oar3_OAR›	83731305	-0.04008
27	oar3_OAR›	83735944	-0.02347

27	oar3_OAR)	83737489	0.096449
27	oar3_OAR)	83749467	0.087233
27	oar3_OAR)	83754043	0.015777
27	oar3_OAR)	83757752	0.078144
27	oar3_OAR)	83761198	-0.0284
27	oar3_OAR)	83767401	-0.0284
27	oar3_OAR)	83767522	0.030397
27	oar3_OAR)	83769302	-0.03218
27	oar3_OAR)	83772995	0.070392
27	oar3_OAR)	83773819	-0.01727
27	oar3_OAR)	83778347	0.01501
27	oar3_OAR)	83820720	0.023625
27	oar3_OAR)	83820869	0.007178
27	oar3_OAR)	83839160	0.039198
27	oar3_OAR)	83840728	0.000283
27	oar3_OAR)	83841010	-0.02336
27	oar3_OAR)	83874654	-0.02336
27	oar3_OAR)	83876495	-0.0156
27	oar3_OAR)	83876569	-0.02755
27	oar3_OAR)	83878977	-0.02755
27	oar3_OAR)	83884390	-0.0156
27	oar3_OAR)	83895336	-0.0156
27	oar3_OAR)	83896374	-0.02151
27	oar3_OAR)	83903635	-0.02336
27	oar3_OAR)	83904382	-0.0156
27	oar3_OAR)	83911276	0.007039
27	oar3_OAR)	83913095	0.000708
27	oar3_OAR)	83913151	-0.0156
27	oar3_OAR)	83922246	-0.01153
27	oar3_OAR)	83922672	0.007039
27	oar3_OAR)	83930093	-0.01153
27	oar3_OAR)	83934755	-0.02336
27	oar3_OAR)	83976637	-0.00384
27	oar3_OAR)	83979073	0.027784
27	oar3_OAR)	83987905	-0.02336
27	oar3_OAR)	83991461	-0.02805
27	oar3_OAR)	83994447	-0.02805
27	oar3_OAR)	83997691	0.007178
27	oar3_OAR)	83998632	-0.01644
27	oar3_OAR)	84001537	-0.02805
27	oar3_OAR)	84011866	-0.01775
27	oar3_OAR)	84012484	-0.02981
27	oar3_OAR)	84012556	-0.01328
27	oar3_OAR)	84026519	-0.03571
27	oar3_OAR)	84037732	-0.01636
27	oar3_OAR)	84043187	0.081485
27	oar3_OAR)	84055080	-0.0151
27	oar3_OAR)	84055600	0.019617
27	oar3_OAR)	84071936	-0.01415
27	oar3_OAR)	84074851	-0.02981
27	oar3_OAR)	84083110	-0.02981
27	oar3_OAR)	84087553	-0.0016
27	oar3_OAR)	84089913	-0.02981
27	oar3_OAR)	84090563	-0.0016

27	oar3_OAR›	84093798	-0.0016
27	oar3_OAR›	84100222	-0.0016
27	oar3_OAR›	84101866	-0.0016
27	oar3_OAR›	84102689	-0.01775
27	oar3_OAR›	84104176	-0.0016
27	oar3_OAR›	84109883	-0.0016
27	oar3_OAR›	84123282	-0.01775
27	oar3_OAR›	84123667	-0.02981
27	oar3_OAR›	84129753	-0.02304
27	oar3_OAR›	84137674	0.000889
27	oar3_OAR›	84140263	-0.01859
27	oar3_OAR›	84148712	-0.01859
27	oar3_OAR›	84160117	-0.0113
27	oar3_OAR›	84164296	-0.0042
27	oar3_OAR›	84170133	-0.00195
27	oar3_OAR›	84170792	0.001737
27	oar3_OAR›	84171598	-0.02664
27	oar3_OAR›	84175053	-0.0218
27	oar3_OAR›	84177067	-0.0218
27	oar3_OAR›	84181491	-0.02424
27	oar3_OAR›	84182755	0.019393
27	oar3_OAR›	84193734	-0.02424
27	oar3_OAR›	84197538	0.019393
27	oar3_OAR›	84210974	-0.02424
27	oar3_OAR›	84221612	0.081485
27	oar3_OAR›	84225832	-0.02857
27	oar3_OAR›	84226276	-0.02049
27	oar3_OAR›	84233207	-0.03163
27	oar3_OAR›	84233644	-0.0283
27	oar3_OAR›	84236189	-0.02049
27	oar3_OAR›	84244000	-0.00508
27	oar3_OAR›	84245623	-0.02049
27	oar3_OAR›	84247480	-0.00429
27	oar3_OAR›	84249833	-0.0232
27	oar3_OAR›	84253983	-0.0008
27	oar3_OAR›	84268519	-0.02228
27	oar3_OAR›	84269729	-0.02049
27	oar3_OAR›	84273264	-0.02228
27	oar3_OAR›	84273998	-0.02228
27	oar3_OAR›	84282553	-0.04175
27	oar3_OAR›	84290268	0.011067
27	oar3_OAR›	84291569	0.088898
27	oar3_OAR›	84293070	0.088898
27	oar3_OAR›	84294910	0.090287
27	oar3_OAR›	84299718	0.059419
27	oar3_OAR›	84303338	0.026955
27	oar3_OAR›	84307497	-0.00898
27	oar3_OAR›	84308820	0.051069
27	oar3_OAR›	84317506	-0.00898
27	oar3_OAR›	84318764	0.022582
27	oar3_OAR›	84332486	-0.00898
27	oar3_OAR›	84333785	0.038996
27	oar3_OAR›	84345062	0.038996
27	oar3_OAR›	84348940	0.075764

27	oar3_OAR›	84366753	-0.00898
27	oar3_OAR›	84368788	-0.00898
27	oar3_OAR›	84369363	-0.00898
27	oar3_OAR›	84378164	0.075764
27	oar3_OAR›	84398288	0.075764
27	oar3_OAR›	84403221	0.02024
27	oar3_OAR›	84404482	0.004594
27	oar3_OAR›	84413551	0.100812
27	oar3_OAR›	84419930	0.004594
27	oar3_OAR›	84421683	0.000135
27	oar3_OAR›	84423538	-0.00809
27	oar3_OAR›	84431699	0.091007
27	oar3_OAR›	84433513	0.091007
27	oar3_OAR›	84442808	0.091007
27	oar3_OAR›	84443102	0.003055
27	oar3_OAR›	84448354	0.004594
27	oar3_OAR›	84464633	0.091007
27	oar3_OAR›	84469230	0.004594
27	oar3_OAR›	84474900	-0.01766
27	oar3_OAR›	84480388	-0.01766
27	oar3_OAR›	84498196	-0.0043
27	oar3_OAR›	84506896	-0.00068
27	oar3_OAR›	84515473	-0.00068
27	oar3_OAR›	84527655	7.84E-05
27	oar3_OAR›	84530137	-0.0043
27	oar3_OAR›	84530163	-0.0043
27	oar3_OAR›	84534425	0.047198
27	oar3_OAR›	84540787	-0.00068
27	oar3_OAR›	84542120	0.000289
27	oar3_OAR›	84542931	0.000289
27	oar3_OAR›	84549584	0.027738
27	oar3_OAR›	84551626	NA
27	oar3_OAR›	84553110	0.04444
27	oar3_OAR›	84560296	0.03753
27	oar3_OAR›	84563177	0.040691
27	oar3_OAR›	84563378	0.047624
27	oar3_OAR›	84563529	0.040691
27	oar3_OAR›	84571448	0.03644
27	oar3_OAR›	84580436	NA
27	oar3_OAR›	84580706	0.047624
27	oar3_OAR›	84589108	0.043549
27	oar3_OAR›	84595290	0.038954
27	oar3_OAR›	84609588	0.047624
27	oar3_OAR›	84611424	0.070927
27	oar3_OAR›	84611566	0.070927
27	oar3_OAR›	84615371	0.047624
27	oar3_OAR›	84619391	0.089452
27	oar3_OAR›	84623857	0.089452
27	oar3_OAR›	84636273	0.070927
27	oar3_OAR›	84638197	0.070927
27	oar3_OAR›	84645099	0.132214
27	oar3_OAR›	84660965	0.04739
27	oar3_OAR›	84664820	0.014893
27	oar3_OAR›	84670449	-0.00011

27	oar3_OAR〉	84675482	0.132214
27	oar3_OAR〉	84676928	0.044009
27	oar3_OAR〉	84679450	0.008071
27	oar3_OAR〉	84683707	0.132214
27	oar3_OAR〉	84692543	0.032181
27	oar3_OAR〉	84693862	0.004999
27	oar3_OAR〉	84699959	0.04483
27	oar3_OAR〉	84707192	0.059047
27	oar3_OAR〉	84707384	0.04483
27	oar3_OAR〉	84712646	0.158239
27	oar3_OAR〉	84713883	0.04483
27	oar3_OAR〉	84715544	0.04483
27	oar3_OAR〉	84718032	0.050664
27	oar3_OAR〉	84726869	0.038382
27	oar3_OAR〉	84727518	0.038382
27	oar3_OAR〉	84727641	0.050664
27	oar3_OAR〉	84735595	0.021004
27	oar3_OAR〉	84737775	0.059403
27	oar3_OAR〉	84744354	0.048953
27	oar3_OAR〉	84748088	0.048953
27	oar3_OAR〉	84749231	0.059403
27	oar3_OAR〉	84759739	0.048953
27	oar3_OAR〉	84766057	0.059403
27	oar3_OAR〉	84773858	0.059403
27	oar3_OAR〉	84774417	0.048953
27	oar3_OAR〉	84775683	0.088043
27	oar3_OAR〉	84790937	0.048953
27	oar3_OAR〉	84792494	0.048953
27	oar3_OAR〉	84797233	0.006364
27	oar3_OAR〉	84798160	0.009542
27	oar3_OAR〉	84799274	0.009542
27	oar3_OAR〉	84811656	0.2902
27	oar3_OAR〉	84811982	-0.00697
27	oar3_OAR〉	84812232	0.124961
27	oar3_OAR〉	84829120	0.2902
27	oar3_OAR〉	84829321	0.0061
27	oar3_OAR〉	84834316	0.031348
27	oar3_OAR〉	84836233	-0.00322
27	oar3_OAR〉	84843680	0.169971
27	oar3_OAR〉	84845447	0.034744
27	oar3_OAR〉	84848381	-0.02018
27	oar3_OAR〉	84858212	0.030434
27	oar3_OAR〉	84868769	-0.02018
27	oar3_OAR〉	84869616	0.169971
27	oar3_OAR〉	84869880	0.025277
27	oar3_OAR〉	84875504	0.338393
27	oar3_OAR〉	84881112	0.338393
27	oar3_OAR〉	84891468	0.014345
27	oar3_OAR〉	84893117	0.276114
27	oar3_OAR〉	84898273	0.338393
27	oar3_OAR〉	84911834	0.01078
27	oar3_OAR〉	84912045	0.276114
27	oar3_OAR〉	84922651	0.276114
27	oar3_OAR〉	84928604	-0.00259

27	oar3_OAR⟩	84938554	0.276114
27	oar3_OAR⟩	84945633	0.276114
27	oar3_OAR⟩	84945875	0.276114
27	oar3_OAR⟩	84949922	0.276114
27	oar3_OAR⟩	84953596	-0.00259
27	oar3_OAR⟩	85019842	0.22949
27	oar3_OAR⟩	85062218	-0.00259
27	oar3_OAR⟩	85088201	0.22949
27	oar3_OAR⟩	85112536	0.167401
27	oar3_OAR⟩	85113849	0.167401
27	oar3_OAR⟩	85139234	0.092229
27	oar3_OAR⟩	85165647	0.092229
27	oar3_OAR⟩	85183121	0.146948
27	oar3_OAR⟩	85183318	0.026576
27	oar3_OAR⟩	85203445	0.128809
27	oar3_OAR⟩	85207279	0.146948
27	oar3_OAR⟩	85212971	0.063134
27	oar3_OAR⟩	85213834	0.063134
27	oar3_OAR⟩	85217352	0.166589
27	oar3_OAR⟩	85227823	0.167401
27	oar3_OAR⟩	85231480	0.063134
27	oar3_OAR⟩	85235233	########
27	oar3_OAR⟩	85236936	0.165644
27	oar3_OAR⟩	85241031	0.195857
27	oar3_OAR⟩	85255428	-0.02049
27	oar3_OAR⟩	85256502	0.145539
27	oar3_OAR⟩	85259316	0.208993
27	oar3_OAR⟩	85259382	0.195857
27	oar3_OAR⟩	85266681	0.208993
27	oar3_OAR⟩	85280290	0.195857
27	oar3_OAR⟩	85296666	0.208993
27	oar3_OAR⟩	85298166	0.22949
27	oar3_OAR⟩	85298952	0.195857
27	oar3_OAR⟩	85299635	-0.00435
27	oar3_OAR⟩	85314376	-0.02049
27	oar3_OAR⟩	85322661	-0.02049
27	oar3_OAR⟩	85322729	-0.02049
27	oar3_OAR⟩	85323112	-0.02049
27	oar3_OAR⟩	85335376	0.170477
27	oar3_OAR⟩	85336989	0.063134
27	oar3_OAR⟩	85337049	0.165644
27	oar3_OAR⟩	85348538	0.168123
27	oar3_OAR⟩	85349415	0.250832
27	oar3_OAR⟩	85358789	-0.02922
27	oar3_OAR⟩	85368290	-0.02922
27	oar3_OAR⟩	85380879	0.030434
27	oar3_OAR⟩	85381904	0.105415
27	oar3_OAR⟩	85383059	0.125815
27	oar3_OAR⟩	85390000	-0.0236
27	oar3_OAR⟩	85392628	0.201636
27	oar3_OAR⟩	85393391	0.078196
27	oar3_OAR⟩	85395855	-0.01961
27	oar3_OAR⟩	85400746	0.063134
27	oar3_OAR⟩	85404244	0.201636

27	oar3_OAR›	85404447	0.037433
27	oar3_OAR›	85413705	0.073768
27	oar3_OAR›	85416733	0.201636
27	oar3_OAR›	85420005	-0.01573
27	oar3_OAR›	85426126	0.178161
27	oar3_OAR›	85449803	-0.01315
27	oar3_OAR›	85468820	0.193336
27	oar3_OAR›	85470604	0.04015
27	oar3_OAR›	85471556	0.178161
27	oar3_OAR›	85474280	0.04015
27	oar3_OAR›	85481618	0.178161
27	oar3_OAR›	85483581	0.194981
27	oar3_OAR›	85484763	0.04015
27	oar3_OAR›	85498381	0.035332
27	oar3_OAR›	85498984	-0.00797
27	oar3_OAR›	85501162	-0.02603
27	oar3_OAR›	85512498	0.171219
27	oar3_OAR›	85519577	0.171219
27	oar3_OAR›	85522331	0.057268
27	oar3_OAR›	85538125	-0.01818
27	oar3_OAR›	85539177	0.138854
27	oar3_OAR›	85562132	0.053616
27	oar3_OAR›	85571745	0.076447
27	oar3_OAR›	85573677	########
27	oar3_OAR›	85576847	0.037756
27	oar3_OAR›	85590448	0.019722
27	oar3_OAR›	85602001	-0.01068
27	oar3_OAR›	85621346	0.058103
27	oar3_OAR›	85621590	-0.03439
27	oar3_OAR›	85630325	-0.00308
27	oar3_OAR›	85634392	-0.03355
27	oar3_OAR›	85639067	-0.00308
27	oar3_OAR›	85652357	-0.01282
27	oar3_OAR›	85662735	0.005579
27	oar3_OAR›	85664402	-0.01282
27	oar3_OAR›	85679030	0.052755
27	oar3_OAR›	85679984	0.032661
27	oar3_OAR›	85683640	0.043484
27	oar3_OAR›	85684107	-0.02921
27	oar3_OAR›	85686585	0.043484
27	oar3_OAR›	85691746	0.052755
27	oar3_OAR›	85697441	0.049274
27	oar3_OAR›	85709693	-0.01859
27	oar3_OAR›	85715544	-0.02695
27	oar3_OAR›	85721304	-0.02921
27	oar3_OAR›	85728168	-0.01415
27	oar3_OAR›	85742115	-0.02716
27	oar3_OAR›	85750207	-0.01415
27	oar3_OAR›	85750362	-0.01415
27	oar3_OAR›	85792004	0.032703
27	oar3_OAR›	85793799	0.075827
27	oar3_OAR›	85801051	-0.03363
27	oar3_OAR›	85813897	0.025372
27	oar3_OAR›	85829775	-0.01815

27	oar3_OAR›	85838425	-0.01815
27	oar3_OAR›	85856304	-0.01815
27	oar3_OAR›	85865722	-0.02295
27	oar3_OAR›	85870162	0.000239
27	oar3_OAR›	85879309	0.000239
27	oar3_OAR›	85884086	-0.02295
27	oar3_OAR›	85884591	0.000239
27	oar3_OAR›	85892451	0.000239
27	oar3_OAR›	85897485	-0.02295
27	oar3_OAR›	85904595	-0.02295
27	oar3_OAR›	85905631	-0.02295
27	oar3_OAR›	85906079	0.006306
27	oar3_OAR›	85916560	0.0062
27	oar3_OAR›	85922065	0.0062
27	oar3_OAR›	85923333	0.004928
27	oar3_OAR›	85944706	0.043011
27	oar3_OAR›	85945954	0.004148
27	oar3_OAR›	85954402	0.043011
27	oar3_OAR›	85961694	0.043011
27	oar3_OAR›	85978623	-0.01445
27	oar3_OAR›	85981546	-0.01546
27	oar3_OAR›	85999171	0.014684
27	oar3_OAR›	85999311	0.126758
27	oar3_OAR›	86005493	0.043011
27	oar3_OAR›	86007576	0.000427
27	oar3_OAR›	86014720	0.014684
27	oar3_OAR›	86016955	0.126758
27	oar3_OAR›	86023346	0.18275
27	oar3_OAR›	86037491	-0.02268
27	oar3_OAR›	86046222	0.103364
27	oar3_OAR›	86049544	-0.01546
27	oar3_OAR›	86056488	0.083495
27	oar3_OAR›	86058026	0.056293
27	oar3_OAR›	86059967	-0.00279
27	oar3_OAR›	86062126	0.001544
27	oar3_OAR›	86069183	-0.03075
27	oar3_OAR›	86069233	0.03994
27	oar3_OAR›	86075529	0.003874
27	oar3_OAR›	86076133	0.016594
27	oar3_OAR›	86079030	0.016594
27	oar3_OAR›	86080021	0.016594
27	oar3_OAR›	86084112	0.109539
27	oar3_OAR›	86087347	-0.02785
27	oar3_OAR›	86089465	0.032887
27	oar3_OAR›	86091675	-0.02785
27	oar3_OAR›	86093548	0.084572
27	oar3_OAR›	86099469	0.084572
27	oar3_OAR›	86115185	0.068095
27	oar3_OAR›	86120017	-0.02642
27	oar3_OAR›	86120033	0.038505
27	oar3_OAR›	86129432	0.057443
27	oar3_OAR›	86130156	-0.02642
27	oar3_OAR›	86134975	0.063459
27	oar3_OAR›	86137758	0.057704

27	oar3_OAR〉	86144203	0.092047
27	oar3_OAR〉	86154711	0.008408
27	oar3_OAR〉	86156154	0.017537
27	oar3_OAR〉	86161257	0.069111
27	oar3_OAR〉	86162495	0.069111
27	oar3_OAR〉	86168338	0.069111
27	oar3_OAR〉	86171582	0.069111
27	oar3_OAR〉	86184298	0.117388
27	oar3_OAR〉	86188904	0.017537
27	oar3_OAR〉	86192854	0.050915
27	oar3_OAR〉	86193181	0.050915
27	oar3_OAR〉	86199036	-0.00445
27	oar3_OAR〉	86200342	0.052414
27	oar3_OAR〉	86207353	########
27	oar3_OAR〉	86209953	0.039811
27	oar3_OAR〉	86210871	0.095171
27	oar3_OAR〉	86211670	0.045102
27	oar3_OAR〉	86217888	0.070548
27	oar3_OAR〉	86230929	-0.0027
27	oar3_OAR〉	86231025	0.000385
27	oar3_OAR〉	86232460	0.089
27	oar3_OAR〉	86237972	0.048383
27	oar3_OAR〉	86239347	0.032615
27	oar3_OAR〉	86239563	-0.02827
27	oar3_OAR〉	86246011	0.095481
27	oar3_OAR〉	86250614	-0.02032
27	oar3_OAR〉	86252045	0.032615
27	oar3_OAR〉	86262348	0.032615
27	oar3_OAR〉	86262461	0.107719
27	oar3_OAR〉	86262702	-0.03382
27	oar3_OAR〉	86263822	-0.003
27	oar3_OAR〉	86270208	0.00598
27	oar3_OAR〉	86271161	0.009694
27	oar3_OAR〉	86272516	0.019669
27	oar3_OAR〉	86279607	-0.00259
27	oar3_OAR〉	86287560	0.006881
27	oar3_OAR〉	86287825	0.09229
27	oar3_OAR〉	86288369	-0.00567
27	oar3_OAR〉	86293611	0.09229
27	oar3_OAR〉	86301613	0.09229
27	oar3_OAR〉	86306584	0.017879
27	oar3_OAR〉	86306663	0.09229
27	oar3_OAR〉	86315837	0.010847
27	oar3_OAR〉	86316395	0.017879
27	oar3_OAR〉	86321447	0.010847
27	oar3_OAR〉	86330870	0.015757
27	oar3_OAR〉	86334188	0.024495
27	oar3_OAR〉	86340121	0.028578
27	oar3_OAR〉	86342574	0.096509
27	oar3_OAR〉	86354892	0.096509
27	oar3_OAR〉	86380297	0.006716
27	oar3_OAR〉	86380774	-0.0285
27	oar3_OAR〉	86386787	0.028578
27	oar3_OAR〉	86389148	0.044345

27	oar3_OAR›	86392879	-0.02228
27	oar3_OAR›	86393105	-0.02228
27	oar3_OAR›	86405133	-0.00289
27	oar3_OAR›	86414352	0.012429
27	oar3_OAR›	86428194	-0.02228
27	oar3_OAR›	86447244	0.028616
27	oar3_OAR›	86448962	0.037433
27	oar3_OAR›	86450688	0.001015
27	oar3_OAR›	86453784	0.028616
27	oar3_OAR›	86464522	-0.01333
27	oar3_OAR›	86469600	-0.01333
27	oar3_OAR›	86474729	0.037433
27	oar3_OAR›	86483662	0.028578
27	oar3_OAR›	86486672	0.037433
27	oar3_OAR›	86496369	0.074726
27	oar3_OAR›	86499222	0.028939
27	oar3_OAR›	86499647	0.098474
27	oar3_OAR›	86516104	-0.01573
27	oar3_OAR›	86517799	0.037433
27	oar3_OAR›	86526434	0.008997
27	oar3_OAR›	86540939	0.018202
27	oar3_OAR›	86569639	0.018169
27	oar3_OAR›	86569915	0.018169
27	oar3_OAR›	86572304	0.008997
27	oar3_OAR›	86585066	0.008997
27	oar3_OAR›	86585280	0.006953
27	oar3_OAR›	86585661	0.008997
27	oar3_OAR›	86587806	0.00011
27	oar3_OAR›	86598418	0.043222
27	oar3_OAR›	86598900	0.026563
27	oar3_OAR›	86601862	0.00011
27	oar3_OAR›	86604506	0.026563
27	oar3_OAR›	86615224	0.008997
27	oar3_OAR›	86621975	0.008997
27	oar3_OAR›	86625182	0.014883
27	oar3_OAR›	86625537	0.011834
27	oar3_OAR›	86630290	0.040756
27	oar3_OAR›	86638398	0.095783
27	oar3_OAR›	86640083	0.047556
27	oar3_OAR›	86648943	0.084357
27	oar3_OAR›	86649901	-0.01378
27	oar3_OAR›	86654702	0.044724
27	oar3_OAR›	86663071	0.0839
27	oar3_OAR›	86663718	0.090567
27	oar3_OAR›	86678846	0.029875
27	oar3_OAR›	86679264	0.029875
27	oar3_OAR›	86680734	0.114237
27	oar3_OAR›	86682346	-0.02036
27	oar3_OAR›	86689029	0.019184
27	oar3_OAR›	86690099	0.091198
27	oar3_OAR›	86690866	0.014267
27	oar3_OAR›	86691976	0.014267
27	oar3_OAR›	86706294	0.014267
27	oar3_OAR›	86706856	0.014267

27	oar3_OAR›	86707305	0.014267
27	oar3_OAR›	86709195	0.002627
27	oar3_OAR›	86717707	0.135158
27	oar3_OAR›	86718526	0.135158
27	oar3_OAR›	86718610	0.049
27	oar3_OAR›	86719795	-0.02677
27	oar3_OAR›	86723625	-0.02159
27	oar3_OAR›	86731191	0.135158
27	oar3_OAR›	86731298	0.178669
27	oar3_OAR›	86739561	0.105703
27	oar3_OAR›	86744015	0.092099
27	oar3_OAR›	86744496	0.018804
27	oar3_OAR›	86754382	0.105703
27	oar3_OAR›	86754926	0.130855
27	oar3_OAR›	86767056	0.116133
27	oar3_OAR›	86779583	0.009277
27	oar3_OAR›	86781586	0.01741
27	oar3_OAR›	86782421	0.015591
27	oar3_OAR›	86789223	0.150866
27	oar3_OAR›	86792181	0.168278
27	oar3_OAR›	86792933	0.035644
27	oar3_OAR›	86794531	0.116133
27	oar3_OAR›	86803648	0.036944
27	oar3_OAR›	86805165	0.036944
27	oar3_OAR›	86814554	0.151378
27	oar3_OAR›	86826494	0.092603
27	oar3_OAR›	86834314	0.116133
27	oar3_OAR›	86835489	0.072747
27	oar3_OAR›	86840302	0.116133
27	oar3_OAR›	86841622	0.072747
27	oar3_OAR›	86845268	0.043941
27	oar3_OAR›	86847141	0.130972
27	oar3_OAR›	86865145	-0.03418
27	oar3_OAR›	86870959	0.048332
27	oar3_OAR›	86871587	-0.00898
27	oar3_OAR›	86879649	-0.01709
27	oar3_OAR›	86880631	0.025449
27	oar3_OAR›	86881494	0.069765
27	oar3_OAR›	86884770	0.002693
27	oar3_OAR›	86886390	0.023934
27	oar3_OAR›	86901115	-0.04177
27	oar3_OAR›	86901930	0.009825
27	oar3_OAR›	86902698	0.059902
27	oar3_OAR›	86915067	0.059902
27	oar3_OAR›	86922997	-0.025
27	oar3_OAR›	86933372	-0.00213
27	oar3_OAR›	86933922	-0.00213
27	oar3_OAR›	86939459	-0.02838
27	oar3_OAR›	86941813	0.024024
27	oar3_OAR›	86946731	0.024024
27	oar3_OAR›	86949789	-0.0288
27	oar3_OAR›	86951581	-0.00798
27	oar3_OAR›	86951850	0.024024
27	oar3_OAR›	86951998	-0.03475

27	oar3_OAR›	86961104	-0.03293
27	oar3_OAR›	86965599	-0.0288
27	oar3_OAR›	86967152	0.029527
27	oar3_OAR›	86973423	0.050218
27	oar3_OAR›	86977768	0.050218
27	oar3_OAR›	86989873	-0.01527
27	oar3_OAR›	86992104	-0.03012
27	oar3_OAR›	86992952	-0.03012
27	oar3_OAR›	86994469	-0.00987
27	oar3_OAR›	86998634	0.018366
27	oar3_OAR›	87006056	-0.02772
27	oar3_OAR›	87006781	-0.02051
27	oar3_OAR›	87011033	-0.02772
27	oar3_OAR›	87020811	-0.00912
27	oar3_OAR›	87022242	0.003301
27	oar3_OAR›	87025586	-0.00584
27	oar3_OAR›	87033041	0.002897
27	oar3_OAR›	87035969	0.002897
27	oar3_OAR›	87037169	-0.00584
27	oar3_OAR›	87040711	-0.02606
27	oar3_OAR›	87044455	-0.00298
27	oar3_OAR›	87046315	0.002897
27	oar3_OAR›	87047716	-0.03188
27	oar3_OAR›	87051235	-0.00304
27	oar3_OAR›	87054345	-0.03188
27	oar3_OAR›	87056781	-0.02746
27	oar3_OAR›	87056901	-0.01046
27	oar3_OAR›	87062867	0.093272
27	oar3_OAR›	87064964	0.019413
27	oar3_OAR›	87070473	0.018388
27	oar3_OAR›	87071009	0.020874
27	oar3_OAR›	87071384	-0.02454
27	oar3_OAR›	87075981	0.02539
27	oar3_OAR›	87080972	-0.03429
27	oar3_OAR›	87081253	0.026977
27	oar3_OAR›	87083253	0.011328
27	oar3_OAR›	87083443	0.011328
27	oar3_OAR›	87088718	0.011328
27	oar3_OAR›	87097829	-0.03435
27	oar3_OAR›	87098176	-0.02377
27	oar3_OAR›	87099387	0.00481
27	oar3_OAR›	87106973	0.038505
27	oar3_OAR›	87107180	-0.02565
27	oar3_OAR›	87109362	0.051958
27	oar3_OAR›	87113475	-0.00863
27	oar3_OAR›	87115024	0.048106
27	oar3_OAR›	87118478	0.002308
27	oar3_OAR›	87118672	-0.0216
27	oar3_OAR›	87120290	0.002308
27	oar3_OAR›	87124785	0.032086
27	oar3_OAR›	87128793	-0.01402
27	oar3_OAR›	87134460	########
27	oar3_OAR›	87134635	-0.00013
27	oar3_OAR›	87139618	-0.02603

27	oar3_OAR›	87146315	########
27	oar3_OAR›	87150763	-0.00935
27	oar3_OAR›	87152195	-0.00935
27	oar3_OAR›	87161396	-0.02119
27	oar3_OAR›	87162272	0.004281
27	oar3_OAR›	87163007	0.025477
27	oar3_OAR›	87167179	-0.02766
27	oar3_OAR›	87168352	0.004281
27	oar3_OAR›	87170949	-0.02347
27	oar3_OAR›	87177211	-0.00751
27	oar3_OAR›	87181127	-0.00298
27	oar3_OAR›	87183549	-0.00751
27	oar3_OAR›	87185264	-0.00606
27	oar3_OAR›	87188798	0.039358
27	oar3_OAR›	87189173	0.049076
27	oar3_OAR›	87189806	0.046507
27	oar3_OAR›	87195144	0.016689
27	oar3_OAR›	87197434	-0.00065
27	oar3_OAR›	87201824	-0.01777
27	oar3_OAR›	87201978	0.013887
27	oar3_OAR›	87209485	-0.02896
27	oar3_OAR›	87211795	0.024356
27	oar3_OAR›	87215202	-0.00707
27	oar3_OAR›	87215295	0.040514
27	oar3_OAR›	87216144	0.03458
27	oar3_OAR›	87222991	0.01642
27	oar3_OAR›	87229647	-0.01643
27	oar3_OAR›	87230643	-0.03219
27	oar3_OAR›	87230746	-0.03219
27	oar3_OAR›	87240840	0.007438
27	oar3_OAR›	87243070	0.009004
27	oar3_OAR›	87243125	-0.01356
27	oar3_OAR›	87247241	0.066598
27	oar3_OAR›	87254694	0.009496
27	oar3_OAR›	87261080	-0.03679
27	oar3_OAR›	87262277	0.010617
27	oar3_OAR›	87263488	-0.01321
27	oar3_OAR›	87272047	0.02221
27	oar3_OAR›	87286387	-0.02982
27	oar3_OAR›	87288718	-0.00407
27	oar3_OAR›	87288787	-0.02982
27	oar3_OAR›	87291237	-0.01544
27	oar3_OAR›	87298615	-0.0185
27	oar3_OAR›	87299593	-0.00936
27	oar3_OAR›	87301587	########
27	oar3_OAR›	87308168	0.033773
27	oar3_OAR›	87317830	-0.01317
27	oar3_OAR›	87318110	0.033773
27	oar3_OAR›	87318596	-0.01735
27	oar3_OAR›	87323596	0.02374
27	oar3_OAR›	87330779	0.025417
27	oar3_OAR›	87331501	-0.02406
27	oar3_OAR›	87333611	0.003291
27	oar3_OAR›	87334097	-0.01679

27	oar3_OAR〉	87345298	-0.01406
27	oar3_OAR〉	87350095	0.033466
27	oar3_OAR〉	87354164	-0.00234
27	oar3_OAR〉	87355159	0.026834
27	oar3_OAR〉	87355515	0.140288
27	oar3_OAR〉	87355863	-0.01142
27	oar3_OAR〉	87370998	-0.02699
27	oar3_OAR〉	87376640	-0.0109
27	oar3_OAR〉	87377424	0.046857
27	oar3_OAR〉	87380925	0.023596
27	oar3_OAR〉	87382409	1.82E-05
27	oar3_OAR〉	87412542	0.086472
27	oar3_OAR〉	87415590	0.084818
27	oar3_OAR〉	87420068	0.016176
27	oar3_OAR〉	87420185	0.027147
27	oar3_OAR〉	87436623	0.014419
27	oar3_OAR〉	87437543	0.058681
27	oar3_OAR〉	87450142	0.078197
27	oar3_OAR〉	87452019	0.084285
27	oar3_OAR〉	87453180	0.042587
27	oar3_OAR〉	87453255	0.193721
27	oar3_OAR〉	87457061	0.018141
27	oar3_OAR〉	87462437	0.145682
27	oar3_OAR〉	87467351	-0.01194
27	oar3_OAR〉	87468462	-0.01194
27	oar3_OAR〉	87471120	0.065543
27	oar3_OAR〉	87478964	-0.03461
27	oar3_OAR〉	87479110	0.059298
27	oar3_OAR〉	87479281	0.007039
27	oar3_OAR〉	87486727	-0.03461
27	oar3_OAR〉	87487328	0.007039
27	oar3_OAR〉	87489899	0.007304
27	oar3_OAR〉	87490348	-0.03351
27	oar3_OAR〉	87496392	0.020675
27	oar3_OAR〉	87501650	0.11618
27	oar3_OAR〉	87505593	-0.0021
27	oar3_OAR〉	87507722	0.028844
27	oar3_OAR〉	87517482	0.027928
27	oar3_OAR〉	87517606	0.029692
27	oar3_OAR〉	87521892	0.027928
27	oar3_OAR〉	87526904	0.019105
27	oar3_OAR〉	87529190	-0.02905
27	oar3_OAR〉	87529716	0.019509
27	oar3_OAR〉	87533290	0.027928
27	oar3_OAR〉	87534236	0.062292
27	oar3_OAR〉	87537105	0.038792
27	oar3_OAR〉	87539178	0.038792
27	oar3_OAR〉	87540552	0.00844
27	oar3_OAR〉	87548276	0.063642
27	oar3_OAR〉	87548692	0.030301
27	oar3_OAR〉	87551430	0.06201
27	oar3_OAR〉	87552845	0.006708
27	oar3_OAR〉	87557927	0.060417
27	oar3_OAR〉	87568137	-0.00678

27	oar3_OAR›	87570393	-0.00678
27	oar3_OAR›	87590800	-0.00678
27	oar3_OAR›	87591118	-0.00678
27	oar3_OAR›	87605979	0.126987
27	oar3_OAR›	87610921	0.030477
27	oar3_OAR›	87612532	0.027864
27	oar3_OAR›	87635932	0.03591
27	oar3_OAR›	87636585	-0.01465
27	oar3_OAR›	87639100	0.011616
27	oar3_OAR›	87642283	0.03591
27	oar3_OAR›	87651050	0.013854
27	oar3_OAR›	87651196	0.025116
27	oar3_OAR›	87655166	-0.01423
27	oar3_OAR›	87655312	-0.00013
27	oar3_OAR›	87667855	-0.00422
27	oar3_OAR›	87669127	0.006902
27	oar3_OAR›	87669726	0.119174
27	oar3_OAR›	87677078	0.057775
27	oar3_OAR›	87683850	0.040679
27	oar3_OAR›	87688978	0.040679
27	oar3_OAR›	87693960	0.040679
27	oar3_OAR›	87702653	0.040679
27	oar3_OAR›	87707617	0.040679
27	oar3_OAR›	87717045	0.040679
27	oar3_OAR›	87722441	0.040679
27	oar3_OAR›	87725192	-0.0018
27	oar3_OAR›	87728271	0.040679
27	oar3_OAR›	87738356	0.040679
27	oar3_OAR›	87742782	0.040679
27	oar3_OAR›	87755337	0.033653
27	oar3_OAR›	87759294	0.050577
27	oar3_OAR›	87763021	0.050577
27	oar3_OAR›	87766773	0.033653
27	oar3_OAR›	87781983	0.050577
27	oar3_OAR›	87786584	-0.02639
27	oar3_OAR›	87793451	0.016478
27	oar3_OAR›	87802371	0.095866
27	oar3_OAR›	87802509	-0.0002
27	oar3_OAR›	87808373	-0.0002
27	oar3_OAR›	87814765	-0.0002
27	oar3_OAR›	87821354	0.009572
27	oar3_OAR›	87825234	0.016478
27	oar3_OAR›	87825712	0.030434
27	oar3_OAR›	87829623	0.045876
27	oar3_OAR›	87829797	0.095866
27	oar3_OAR›	87830763	0.016478
27	oar3_OAR›	87837076	-0.02419
27	oar3_OAR›	87842168	-0.03075
27	oar3_OAR›	87863260	0.05893
27	oar3_OAR›	87863823	0.001145
27	oar3_OAR›	87864611	0.013119
27	oar3_OAR›	87884851	-0.01309
27	oar3_OAR›	87885129	-0.00754
27	oar3_OAR›	87888590	-0.00163

27	oar3_OAR›	87906622	0.056734
27	oar3_OAR›	87926824	0.007901
27	oar3_OAR›	87927057	0.056734
27	oar3_OAR›	87939240	-0.01914
27	oar3_OAR›	87939547	-0.01914
27	oar3_OAR›	87961114	0.081698
27	oar3_OAR›	87963121	0.076553
27	oar3_OAR›	87976517	0.08616
27	oar3_OAR›	87980700	0.081698
27	oar3_OAR›	87981913	-0.03075
27	oar3_OAR›	87990197	-0.03075
27	oar3_OAR›	87990249	-0.03075
27	oar3_OAR›	88000965	0.02468
27	oar3_OAR›	88015461	0.02468
27	oar3_OAR›	88016739	-0.03075
27	oar3_OAR›	88017993	0.081698
27	oar3_OAR›	88025886	0.081698
27	oar3_OAR›	88026435	0.081698
27	oar3_OAR›	88028613	0.02468
27	oar3_OAR›	88030313	0.081698
27	oar3_OAR›	88034488	0.018446
27	oar3_OAR›	88038565	0.016196
27	oar3_OAR›	88042364	0.016196
27	oar3_OAR›	88045898	0.018446
27	oar3_OAR›	88048711	-0.03332
27	oar3_OAR›	88048843	0.006949
27	oar3_OAR›	88054509	0.018446
27	oar3_OAR›	88059009	-0.03497
27	oar3_OAR›	88061191	-0.01635
27	oar3_OAR›	88061663	-0.03497
27	oar3_OAR›	88062837	0.013791
27	oar3_OAR›	88075374	-0.01451
27	oar3_OAR›	88076132	-0.0105
27	oar3_OAR›	88081432	-0.02267
27	oar3_OAR›	88084895	0.013791
27	oar3_OAR›	88094422	-0.01872
27	oar3_OAR›	88103736	-0.02267
27	oar3_OAR›	88104414	0.013791
27	oar3_OAR›	88105455	-0.0105
27	oar3_OAR›	88108834	0.001984
27	oar3_OAR›	88109886	-0.01673
27	oar3_OAR›	88119578	0.013791
27	oar3_OAR›	88119894	-0.02104
27	oar3_OAR›	88119980	-0.02104
27	oar3_OAR›	88147034	-0.02104
27	oar3_OAR›	88160327	-0.01757
27	oar3_OAR›	88185095	-0.03242
27	oar3_OAR›	88190205	-0.01757
27	oar3_OAR›	88190360	-0.01757
27	oar3_OAR›	88195970	-0.01757
27	oar3_OAR›	88204155	-0.00571
27	oar3_OAR›	88204911	0.009734
27	oar3_OAR›	88207451	-0.02068
27	oar3_OAR›	88207900	-0.01199

27	oar3_OAR>	88214167	-0.02922
27	oar3_OAR>	88220156	0.036342
27	oar3_OAR>	88220287	0.012034
27	oar3_OAR>	88225878	0.003457
27	oar3_OAR>	88228963	0.069823
27	oar3_OAR>	88229230	-0.01815
27	oar3_OAR>	88236756	0.003457
27	oar3_OAR>	88237778	0.044645
27	oar3_OAR>	88240328	0.084998
27	oar3_OAR>	88251441	0.036342
27	oar3_OAR>	88252938	0.036342
27	oar3_OAR>	88255530	-0.00181
27	oar3_OAR>	88262817	-0.01815
27	oar3_OAR>	88266266	-0.01815
27	oar3_OAR>	88267407	-0.0156
27	oar3_OAR>	88274016	0.003457
27	oar3_OAR>	88275425	0.003457
27	oar3_OAR>	88286058	0.01839
27	oar3_OAR>	88286248	-0.01574
27	oar3_OAR>	88296859	0.020529
27	oar3_OAR>	88312059	0.006701
27	oar3_OAR>	88318116	-0.0156
27	oar3_OAR>	88323682	-0.004
27	oar3_OAR>	88328699	-0.004
27	oar3_OAR>	88332293	0.034468
27	oar3_OAR>	88332841	0.020529
27	oar3_OAR>	88333650	0.020529
27	oar3_OAR>	88333970	-0.004
27	oar3_OAR>	88342253	-0.004
27	oar3_OAR>	88360194	-0.01574
27	oar3_OAR>	88361010	-0.00606
27	oar3_OAR>	88361580	0.045044
27	oar3_OAR>	88361835	0.045044
27	oar3_OAR>	88369933	0.045044
27	oar3_OAR>	88371612	0.045044
27	oar3_OAR>	88375846	-0.02049
27	oar3_OAR>	88378479	0.045044
27	oar3_OAR>	88382892	-0.01574
27	oar3_OAR>	88383096	-0.01574
27	oar3_OAR>	88391956	-0.01574
27	oar3_OAR>	88395756	-0.01574
27	oar3_OAR>	88397987	-0.02612
27	oar3_OAR>	88400129	-0.01574
27	oar3_OAR>	88411874	-0.02252
27	oar3_OAR>	88413006	0.019192
27	oar3_OAR>	88422623	-0.02252
27	oar3_OAR>	88425398	-0.02612
27	oar3_OAR>	88432057	0.019192
27	oar3_OAR>	88432695	-0.02252
27	oar3_OAR>	88434591	0.062244
27	oar3_OAR>	88443178	-0.02252
27	oar3_OAR>	88454058	-0.02612
27	oar3_OAR>	88454740	-0.0131
27	oar3_OAR>	88454860	-0.01248

27	oar3_OAR›	88469342	0.069843
27	oar3_OAR›	88469398	-0.00748
27	oar3_OAR›	88474864	-0.00748
27	oar3_OAR›	88481142	0.016847
27	oar3_OAR›	88483366	0.010721
27	oar3_OAR›	88483924	0.010721
27	oar3_OAR›	88485526	0.010721
27	oar3_OAR›	88497863	0.02918
27	oar3_OAR›	88501466	0.046305
27	oar3_OAR›	88501911	0.016847
27	oar3_OAR›	88505733	-0.02374
27	oar3_OAR›	88542325	-0.00333
27	oar3_OAR›	88544278	-0.00333
27	oar3_OAR›	88550354	0.009004
27	oar3_OAR›	88550586	-0.01303
27	oar3_OAR›	88559657	-0.00735
27	oar3_OAR›	88574242	-0.01303
27	oar3_OAR›	88578111	0.033254
27	oar3_OAR›	88590842	-0.01303
27	oar3_OAR›	88592997	0.009004
27	oar3_OAR›	88593217	0.001279
27	oar3_OAR›	88599579	-0.01303
27	oar3_OAR›	88600063	0.033254
27	oar3_OAR›	88610686	0.009004
27	oar3_OAR›	88611152	0.033254
27	oar3_OAR›	88611215	0.009004
27	oar3_OAR›	88631072	0.017095
27	oar3_OAR›	88634825	-0.01303
27	oar3_OAR›	88637115	0.009004
27	oar3_OAR›	88646174	0.009004
27	oar3_OAR›	88664291	0.001279
27	oar3_OAR›	88664384	-0.01704
27	oar3_OAR›	88668258	0.001279
27	oar3_OAR›	88689398	0.001279
27	oar3_OAR›	88690526	0.030827
27	oar3_OAR›	88708972	-0.01872
27	oar3_OAR›	88710055	-0.01872
27	oar3_OAR›	88737367	-0.01872
27	oar3_OAR›	88737861	-0.01872
27	oar3_OAR›	88738175	0.028055
27	oar3_OAR›	88740669	0.003028
27	oar3_OAR›	88742846	-0.01872
27	oar3_OAR›	88756807	-0.01872
27	oar3_OAR›	88768296	0.001279
27	oar3_OAR›	88774024	-0.01872
27	oar3_OAR›	88778893	0.028055
27	oar3_OAR›	88779641	0.001279
27	oar3_OAR›	88779943	-0.01872
27	oar3_OAR›	88791682	-0.01872
27	oar3_OAR›	88793055	-0.01872
27	oar3_OAR›	88806006	-0.01872
27	oar3_OAR›	88817001	-0.01872
27	oar3_OAR›	88818427	-0.01872
27	oar3_OAR›	88823614	-0.01872

27	oar3_OAR›	88826806	0.00937
27	oar3_OAR›	88829274	0.012984
27	oar3_OAR›	88835198	0.012984
27	oar3_OAR›	88835884	0.023793
27	oar3_OAR›	88844308	0.012984
27	oar3_OAR›	88844524	-0.01872
27	oar3_OAR›	88845296	0.028055
27	oar3_OAR›	88854686	0.012984
27	oar3_OAR›	88855926	-0.01872
27	oar3_OAR›	88864214	0.012984
27	oar3_OAR›	88870091	-0.01872
27	oar3_OAR›	88883074	0.028055
27	oar3_OAR›	88883793	0.00937
27	oar3_OAR›	88892926	-0.01872
27	oar3_OAR›	88902689	0.00937
27	oar3_OAR›	88903446	-0.01872
27	oar3_OAR›	88903575	0.001279
27	oar3_OAR›	88919888	0.028055
27	oar3_OAR›	88922999	0.00937
27	oar3_OAR›	88929898	-0.00597
27	oar3_OAR›	88930489	0.020688
27	oar3_OAR›	88931165	0.020688
27	oar3_OAR›	88937206	-0.013
27	oar3_OAR›	88940166	-0.00043
27	oar3_OAR›	88942325	0.000464
27	oar3_OAR›	88945387	-0.00847
27	oar3_OAR›	88948185	-0.013
27	oar3_OAR›	88954934	-0.01327
27	oar3_OAR›	88958153	-0.013
27	oar3_OAR›	88958410	-0.01698
27	oar3_OAR›	88958472	-0.013
27	oar3_OAR›	88963365	-0.013
27	oar3_OAR›	88970271	-0.013
27	oar3_OAR›	88970652	0.000464
27	oar3_OAR›	88976687	0.006377
27	oar3_OAR›	89014973	-0.00847
27	oar3_OAR›	89016143	-0.013
27	oar3_OAR›	89027624	0.000464
27	oar3_OAR›	89033754	-0.02736
27	oar3_OAR›	89043908	-0.02835
27	oar3_OAR›	89045910	-0.00838
27	oar3_OAR›	89046500	-0.02736
27	oar3_OAR›	89057335	-0.013
27	oar3_OAR›	89065216	-0.02688
27	oar3_OAR›	89066286	-0.013
27	oar3_OAR›	89070538	-0.0313
27	oar3_OAR›	89071062	-0.02902
27	oar3_OAR›	89085113	-0.01423
27	oar3_OAR›	89085519	0.050818
27	oar3_OAR›	89085957	-0.01423
27	oar3_OAR›	89096667	-0.03606
27	oar3_OAR›	89101267	0.049787
27	oar3_OAR›	89109276	-0.01809
27	oar3_OAR›	89110861	-0.00577

27	oar3_OAR›	89114102	-0.00577
27	oar3_OAR›	89114875	-0.01406
27	oar3_OAR›	89120933	0.053999
27	oar3_OAR›	89121653	0.053999
27	oar3_OAR›	89127207	-0.01141
27	oar3_OAR›	89136783	0.031063
27	oar3_OAR›	89137366	0.031063
27	oar3_OAR›	89138739	0.031063
27	oar3_OAR›	89143959	-0.02699
27	oar3_OAR›	89143980	-0.00158
27	oar3_OAR›	89148925	-0.02699
27	oar3_OAR›	89150712	0.002173
27	oar3_OAR›	89162978	0.02269
27	oar3_OAR›	89164245	0.014501
27	oar3_OAR›	89165492	-0.01141
27	oar3_OAR›	89180211	-0.02003
27	oar3_OAR›	89181902	-0.01234
27	oar3_OAR›	89184017	-0.00738
27	oar3_OAR›	89184874	0.032787
27	oar3_OAR›	89186896	0.032787
27	oar3_OAR›	89190627	-0.01775
27	oar3_OAR›	89193831	-0.02931
27	oar3_OAR›	89201263	-0.01033
27	oar3_OAR›	89202886	0.022643
27	oar3_OAR›	89203921	-0.01142
27	oar3_OAR›	89210890	0.012047
27	oar3_OAR›	89212077	0.01417
27	oar3_OAR›	89219639	0.060929
27	oar3_OAR›	89225430	0.026851
27	oar3_OAR›	89230997	0.062973
27	oar3_OAR›	89231274	-0.00748
27	oar3_OAR›	89240692	-0.02462
27	oar3_OAR›	89262145	-0.00462
27	oar3_OAR›	89273075	-0.02061
27	oar3_OAR›	89280489	0.084811
27	oar3_OAR›	89303696	-0.02061
27	oar3_OAR›	89304840	-0.02061
27	oar3_OAR›	89327057	0.014442
27	oar3_OAR›	89327427	0.019158
27	oar3_OAR›	89331623	-0.02256
27	oar3_OAR›	89332360	-0.01069
27	oar3_OAR›	89342255	0.083051
27	oar3_OAR›	89342991	0.081221
27	oar3_OAR›	89348888	0.083051
27	oar3_OAR›	89351658	0.012047
27	oar3_OAR›	89359770	0.115734
27	oar3_OAR›	89370090	0.024931
27	oar3_OAR›	89384776	0.001575
27	oar3_OAR›	89384867	0.001575
27	oar3_OAR›	89390606	0.001575
27	oar3_OAR›	89397908	-0.01598
27	oar3_OAR›	89410459	-0.01069
27	oar3_OAR›	89417002	-0.01069
27	oar3_OAR›	89417272	-0.01872

27	oar3_OAR›	89419150	0.067835
27	oar3_OAR›	89429915	-0.02793
27	oar3_OAR›	89430577	0.06516
27	oar3_OAR›	89435999	-0.01598
27	oar3_OAR›	89451366	-0.01069
27	oar3_OAR›	89467394	-0.01069
27	oar3_OAR›	89492099	0.006637
27	oar3_OAR›	89499459	0.008123
27	oar3_OAR›	89520145	0.021695
27	oar3_OAR›	89520941	0.021695
27	oar3_OAR›	89526411	-0.00255
27	oar3_OAR›	89533359	0.003904
27	oar3_OAR›	89542720	-0.03601
27	oar3_OAR›	89543858	-0.01746
27	oar3_OAR›	89543981	0.039013
27	oar3_OAR›	89547660	0.039013
27	oar3_OAR›	89549447	0.039013
27	oar3_OAR›	89555513	0.070714
27	oar3_OAR›	89561856	-0.00751
27	oar3_OAR›	89562995	0.166102
27	oar3_OAR›	89563731	0.166102
27	oar3_OAR›	89567136	0.011067
27	oar3_OAR›	89572207	0.12966
27	oar3_OAR›	89579035	0.000605
27	oar3_OAR›	89579480	0.141386
27	oar3_OAR›	89579685	0.12966
27	oar3_OAR›	89587500	-0.02184
27	oar3_OAR›	89591708	0.162809
27	oar3_OAR›	89594122	0.12966
27	oar3_OAR›	89595341	-0.01264
27	oar3_OAR›	89598259	-0.00181
27	oar3_OAR›	89607186	-0.01731
27	oar3_OAR›	89608415	-0.01731
27	oar3_OAR›	89612277	0.037113
27	oar3_OAR›	89619666	-0.01743
27	oar3_OAR›	89620052	-0.02517
27	oar3_OAR›	89620353	-0.02517
27	oar3_OAR›	89623882	-0.02583
27	oar3_OAR›	89639497	-0.03171
27	oar3_OAR›	89639534	-0.02688
27	oar3_OAR›	89639889	-0.02688
27	oar3_OAR›	89642663	-0.02688
27	oar3_OAR›	89650152	0.028864
27	oar3_OAR›	89651698	0.005158
27	oar3_OAR›	89657509	0.010792
27	oar3_OAR›	89664239	0.029915
27	oar3_OAR›	89667277	-0.01628
27	oar3_OAR›	89674026	0.009373
27	oar3_OAR›	89679733	0.031225
27	oar3_OAR›	89682811	0.009373
27	oar3_OAR›	89691885	-0.01348
27	oar3_OAR›	89692260	0.00703
27	oar3_OAR›	89693498	-0.01348
27	oar3_OAR›	89694747	-0.01348

27	oar3_OAR›	89702311	0.160975
27	oar3_OAR›	89703638	-0.01928
27	oar3_OAR›	89704761	-0.03332
27	oar3_OAR›	89708082	0.160975
27	oar3_OAR›	89717233	0.18645
27	oar3_OAR›	89718431	0.18645
27	oar3_OAR›	89724217	0.18645
27	oar3_OAR›	89748091	-0.00693
27	oar3_OAR›	89748556	0.028803
27	oar3_OAR›	89762423	-0.01731
27	oar3_OAR›	89783280	0.008578
27	oar3_OAR›	89796370	0.000889
27	oar3_OAR›	89796454	-0.04332
27	oar3_OAR›	89796546	0.009825
27	oar3_OAR›	89797606	0.016763
27	oar3_OAR›	89812139	-0.01758
27	oar3_OAR›	89815383	0.000821
27	oar3_OAR›	89820441	0.023957
27	oar3_OAR›	89821344	-0.00914
27	oar3_OAR›	89822528	-0.03688
27	oar3_OAR›	89828866	-0.02831
27	oar3_OAR›	89829355	0.0049
27	oar3_OAR›	89845804	-0.02381
27	oar3_OAR›	89850589	-0.02292
27	oar3_OAR›	89856612	0.000134
27	oar3_OAR›	89861459	0.003587
27	oar3_OAR›	89875244	0.020697
27	oar3_OAR›	89879161	0.000134
27	oar3_OAR›	89896360	NA
27	oar3_OAR›	89897879	0.040893
27	oar3_OAR›	89899653	-0.02926
27	oar3_OAR›	89908594	0.041658
27	oar3_OAR›	89911924	-0.01849
27	oar3_OAR›	89917362	-0.00148
27	oar3_OAR›	89924906	-0.01933
27	oar3_OAR›	89927495	-0.01933
27	oar3_OAR›	89941481	0.011567
27	oar3_OAR›	89968418	0.038166
27	oar3_OAR›	89971793	0.009985
27	oar3_OAR›	89976404	0.079476
27	oar3_OAR›	89978407	0.029104
27	oar3_OAR›	89979441	0.009985
27	oar3_OAR›	89979874	-0.00148
27	oar3_OAR›	89984508	0.009985
27	oar3_OAR›	89992809	0.029104
27	oar3_OAR›	89994666	0.033827
27	oar3_OAR›	89994974	0.029104
27	oar3_OAR›	89999974	0.009985
27	oar3_OAR›	90003018	0.000879
27	oar3_OAR›	90003561	0.033827
27	oar3_OAR›	90004873	0.029104
27	oar3_OAR›	90010083	0.022432
27	oar3_OAR›	90015141	0.015729
27	oar3_OAR›	90016331	0.054375

27	oar3_OAR›	90018772	0.033827
27	oar3_OAR›	90022625	-0.00471
27	oar3_OAR›	90040548	0.089096
27	oar3_OAR›	90045871	0.073798
27	oar3_OAR›	90056532	0.059429
27	oar3_OAR›	90062532	########
27	oar3_OAR›	90068840	########
27	oar3_OAR›	90074211	-0.02921
27	oar3_OAR›	90093181	0.008296
27	oar3_OAR›	90099955	-0.02086
27	oar3_OAR›	90109939	-0.00195
27	oar3_OAR›	90157114	0.009129
27	oar3_OAR›	90180030	-0.01396
27	oar3_OAR›	90180587	0.016178
27	oar3_OAR›	90183670	-0.0084
27	oar3_OAR›	90240027	-0.00912
27	oar3_OAR›	90246088	-0.02361
27	oar3_OAR›	90262824	0.002954
27	oar3_OAR›	90273324	0.031091
27	oar3_OAR›	90288181	0.007424
27	oar3_OAR›	90345118	0.089992
27	oar3_OAR›	90354488	0.089992
27	oar3_OAR›	90354892	0.089992
27	oar3_OAR›	90371165	0.081453
27	oar3_OAR›	90372939	0.081453
27	oar3_OAR›	90380139	0.113727
27	oar3_OAR›	90380726	0.081198
27	oar3_OAR›	90387812	-0.0014
27	oar3_OAR›	90394252	-0.03932
27	oar3_OAR›	90397194	-0.03792
27	oar3_OAR›	90397246	-0.03792
27	oar3_OAR›	90409489	-0.04033
27	oar3_OAR›	90410304	-0.04033
27	oar3_OAR›	90411849	-0.03792
27	oar3_OAR›	90418596	0.035647
27	oar3_OAR›	90427595	0.058407
27	oar3_OAR›	90429987	0.079127
27	oar3_OAR›	90435130	0.037762
27	oar3_OAR›	90454303	-0.03296
27	oar3_OAR›	90463179	-0.03231
27	oar3_OAR›	90480387	-0.03296
27	oar3_OAR›	90486033	-0.00501
27	oar3_OAR›	90496419	-0.03296
27	oar3_OAR›	90499898	0.031225
27	oar3_OAR›	90506351	-0.01622
27	oar3_OAR›	90506512	-0.02897
27	oar3_OAR›	90515415	0.092598
27	oar3_OAR›	90517354	0.092598
27	oar3_OAR›	90524126	0.092598
27	oar3_OAR›	90535939	0.067206
27	oar3_OAR›	90536067	0.020334
27	oar3_OAR›	90542545	0.091676
27	oar3_OAR›	90561287	-0.03021
27	oar3_OAR›	90564546	-0.00269

27	oar3_OAR›	90569533	0.087771
27	oar3_OAR›	90580654	0.133686
27	oar3_OAR›	90586177	0.009324
27	oar3_OAR›	90591852	-0.03582
27	oar3_OAR›	90591907	########
27	oar3_OAR›	90603182	0.063485
27	oar3_OAR›	90604436	0.048314
27	oar3_OAR›	90605070	-0.00567
27	oar3_OAR›	90618494	-0.00751
27	oar3_OAR›	90627325	-0.00241
27	oar3_OAR›	90630690	-0.00817
27	oar3_OAR›	90630799	-0.0081
27	oar3_OAR›	90640793	-0.04422
27	oar3_OAR›	90647779	0.00834
27	oar3_OAR›	90650912	-0.01899
27	oar3_OAR›	90656026	0.021987
27	oar3_OAR›	90661660	0.14441
27	oar3_OAR›	90666758	-0.03755
27	oar3_OAR›	90672608	0.050828
27	oar3_OAR›	90673138	-0.00329
27	oar3_OAR›	90681588	-0.03755
27	oar3_OAR›	90686125	-0.01033
27	oar3_OAR›	90686235	0.050828
27	oar3_OAR›	90697920	-0.02159
27	oar3_OAR›	90703192	-0.03508
27	oar3_OAR›	90708234	0.027086
27	oar3_OAR›	90708566	0.14441
27	oar3_OAR›	90708692	0.019791
27	oar3_OAR›	90717466	0.004183
27	oar3_OAR›	90721447	0.008005
27	oar3_OAR›	90721625	0.008005
27	oar3_OAR›	90727238	0.009734
27	oar3_OAR›	90730216	0.117182
27	oar3_OAR›	90731988	0.008005
27	oar3_OAR›	90738431	0.200733
27	oar3_OAR›	90742476	0.026101
27	oar3_OAR›	90743481	0.04919
27	oar3_OAR›	90746839	0.113939
27	oar3_OAR›	90765348	0.149631
27	oar3_OAR›	90765456	-0.00886
27	oar3_OAR›	90778930	0.061524
27	oar3_OAR›	90779317	0.068254
27	oar3_OAR›	90782534	0.05746
27	oar3_OAR›	90783186	0.063434
27	oar3_OAR›	90784476	-0.00287
27	oar3_OAR›	90790831	-0.00287
27	oar3_OAR›	90791152	0.019349
27	oar3_OAR›	90791213	-0.00287
27	oar3_OAR›	90799004	0.063434
27	oar3_OAR›	90815374	-0.00517
27	oar3_OAR›	90815785	0.063734
27	oar3_OAR›	90819878	-0.00517
27	oar3_OAR›	90825482	-0.00517
27	oar3_OAR›	90838092	-0.00517

27	oar3_OAR〉	90838168	0.058371
27	oar3_OAR〉	90857614	0.059202
27	oar3_OAR〉	90868122	0.039636
27	oar3_OAR〉	90880946	-0.01413
27	oar3_OAR〉	90881364	0.049761
27	oar3_OAR〉	90884426	0.019349
27	oar3_OAR〉	90895365	0.113783
27	oar3_OAR〉	90895637	0.090773
27	oar3_OAR〉	90897680	0.113783
27	oar3_OAR〉	90898751	-0.01604
27	oar3_OAR〉	90906569	0.113783
27	oar3_OAR〉	90906685	0.07189
27	oar3_OAR〉	90908121	0.113783
27	oar3_OAR〉	90912561	0.135747
27	oar3_OAR〉	90917964	0.089814
27	oar3_OAR〉	90929562	0.046772
27	oar3_OAR〉	90930543	0.04191
27	oar3_OAR〉	90930756	0.025112
27	oar3_OAR〉	90931404	0.025112
27	oar3_OAR〉	90954501	0.09104
27	oar3_OAR〉	90954884	0.055027
27	oar3_OAR〉	90955441	0.09104
27	oar3_OAR〉	90959990	0.076549
27	oar3_OAR〉	90973293	0.034253
27	oar3_OAR〉	90974423	0.088589
27	oar3_OAR〉	90985147	0.077684
27	oar3_OAR〉	90985213	0.045813
27	oar3_OAR〉	90985666	0.057975
27	oar3_OAR〉	90987059	0.057975
27	oar3_OAR〉	90995576	0.030019
27	oar3_OAR〉	90998856	0.044391
27	oar3_OAR〉	91002319	0.060323
27	oar3_OAR〉	91004401	-0.0059
27	oar3_OAR〉	91006093	0.036586
27	oar3_OAR〉	91010483	0.058224
27	oar3_OAR〉	91018212	0.054073
27	oar3_OAR〉	91019584	0.089856
27	oar3_OAR〉	91021774	-0.0013
27	oar3_OAR〉	91022026	0.028562
27	oar3_OAR〉	91023380	0.054073
27	oar3_OAR〉	91031424	0.089856
27	oar3_OAR〉	91037202	0.028562
27	oar3_OAR〉	91038036	0.028562
27	oar3_OAR〉	91051363	0.028562
27	oar3_OAR〉	91054146	-0.01437
27	oar3_OAR〉	91060142	0.049739
27	oar3_OAR〉	91065328	-0.01456
27	oar3_OAR〉	91068693	0.028562
27	oar3_OAR〉	91084622	0.0365
27	oar3_OAR〉	91088123	0.0365
27	oar3_OAR〉	91091368	0.076251
27	oar3_OAR〉	91093678	0.076251
27	oar3_OAR〉	91099618	0.080574
27	oar3_OAR〉	91100200	0.024496

27	oar3_OAR>	91100829	0.024496
27	oar3_OAR>	91109053	0.035877
27	oar3_OAR>	91109995	0.020184
27	oar3_OAR>	91114688	-0.01268
27	oar3_OAR>	91123067	0.052866
27	oar3_OAR>	91131107	-0.02481
27	oar3_OAR>	91133457	0.010769
27	oar3_OAR>	91136234	-0.02046
27	oar3_OAR>	91136900	-0.01789
27	oar3_OAR>	91139313	-0.01204
27	oar3_OAR>	91146244	0.044627
27	oar3_OAR>	91150824	0.044627
27	oar3_OAR>	91155954	0.064425
27	oar3_OAR>	91156710	0.007446
27	oar3_OAR>	91163273	-0.01789
27	oar3_OAR>	91163582	0.044627
27	oar3_OAR>	91167612	0.044627
27	oar3_OAR>	91174053	-0.02875
27	oar3_OAR>	91175245	-0.01789
27	oar3_OAR>	91184657	-0.01142
27	oar3_OAR>	91187587	-0.00511
27	oar3_OAR>	91188553	8.73E-05
27	oar3_OAR>	91189739	-0.00408
27	oar3_OAR>	91196344	-0.00408
27	oar3_OAR>	91196781	0.016179
27	oar3_OAR>	91198399	-0.00511
27	oar3_OAR>	91205887	-0.00548
27	oar3_OAR>	91207507	0.061242
27	oar3_OAR>	91208941	-0.00511
27	oar3_OAR>	91211241	0.073537
27	oar3_OAR>	91230506	0.016179
27	oar3_OAR>	91242770	0.03733
27	oar3_OAR>	91242821	0.03733
27	oar3_OAR>	91244009	0.03733
27	oar3_OAR>	91260235	-0.01906
27	oar3_OAR>	91262257	-0.00511
27	oar3_OAR>	91269552	-0.0243
27	oar3_OAR>	91269659	0.035537
27	oar3_OAR>	91271026	0.051142
27	oar3_OAR>	91272784	-0.01906
27	oar3_OAR>	91275395	0.035537
27	oar3_OAR>	91279910	-0.0243
27	oar3_OAR>	91282989	-0.00511
27	oar3_OAR>	91286895	-0.00548
27	oar3_OAR>	91292969	-0.02815
27	oar3_OAR>	91296404	-0.02815
27	oar3_OAR>	91302466	-0.02815
27	oar3_OAR>	91302968	0.005326
27	oar3_OAR>	91309871	-0.03921
27	oar3_OAR>	91320184	0.001934
27	oar3_OAR>	91320946	-0.03921
27	oar3_OAR>	91327446	0.136001
27	oar3_OAR>	91328024	0.159797
27	oar3_OAR>	91335283	0.116199

27	oar3_OAR›	91339318	-0.02416
27	oar3_OAR›	91344286	0.116199
27	oar3_OAR›	91357659	0.02221
27	oar3_OAR›	91363536	-0.01834
27	oar3_OAR›	91363618	0.013268
27	oar3_OAR›	91367041	-0.00304
27	oar3_OAR›	91375249	0.033554
27	oar3_OAR›	91379933	0.08299
27	oar3_OAR›	91381728	0.044736
27	oar3_OAR›	91388822	0.029959
27	oar3_OAR›	91390061	0.08296
27	oar3_OAR›	91392598	0.174541
27	oar3_OAR›	91394168	0.124493
27	oar3_OAR›	91399120	0.011041
27	oar3_OAR›	91400183	0.143611
27	oar3_OAR›	91401910	0.067725
27	oar3_OAR›	91410343	0.055044
27	oar3_OAR›	91410869	0.007333
27	oar3_OAR›	91416080	0.12966
27	oar3_OAR›	91418664	0.028743
27	oar3_OAR›	91420682	0.028743
27	oar3_OAR›	91421515	0.044736
27	oar3_OAR›	91427344	0.12966
27	oar3_OAR›	91430746	0.122761
27	oar3_OAR›	91432377	0.143611
27	oar3_OAR›	91433877	0.120924
27	oar3_OAR›	91441112	0.138396
27	oar3_OAR›	91443060	0.073272
27	oar3_OAR›	91443937	0.073272
27	oar3_OAR›	91448715	0.153383
27	oar3_OAR›	91452925	0.138396
27	oar3_OAR›	91453716	0.153383
27	oar3_OAR›	91454770	-0.02512
27	oar3_OAR›	91458826	0.138396
27	oar3_OAR›	91461712	0.200987
27	oar3_OAR›	91464593	0.208901
27	oar3_OAR›	91466197	0.05071
27	oar3_OAR›	91471546	0.189981
27	oar3_OAR›	91477953	0.050472
27	oar3_OAR›	91482833	0.058238
27	oar3_OAR›	91492235	-0.0002
27	oar3_OAR›	91495551	0.00811
27	oar3_OAR›	91500285	-0.00923
27	oar3_OAR›	91503161	-0.0002
27	oar3_OAR›	91505248	-0.01815
27	oar3_OAR›	91505842	0.014649
27	oar3_OAR›	91509323	-0.00615
27	oar3_OAR›	91515817	-0.02327
27	oar3_OAR›	91515831	-0.00271
27	oar3_OAR›	91522933	-0.00857
27	oar3_OAR›	91523864	-0.01365
27	oar3_OAR›	91524622	0.012043
27	oar3_OAR›	91529814	-0.01657
27	oar3_OAR›	91533614	-0.00255

27	oar3_OAR›	91535046	-0.0214
27	oar3_OAR›	91541900	-0.02158
27	oar3_OAR›	91552419	-0.02355
27	oar3_OAR›	91552492	-0.04195
27	oar3_OAR›	91553079	-0.01634
27	oar3_OAR›	91563124	-0.02355
27	oar3_OAR›	91564324	0.011193
27	oar3_OAR›	91570473	-0.03477
27	oar3_OAR›	91578193	0.005602
27	oar3_OAR›	91581732	0.224519
27	oar3_OAR›	91582212	0.012172
27	oar3_OAR›	91590862	-0.03688
27	oar3_OAR›	91599532	-0.03688
27	oar3_OAR›	91607807	-0.00815
27	oar3_OAR›	91617598	0.000135
27	oar3_OAR›	91626208	-0.02793
27	oar3_OAR›	91629343	0.123685
27	oar3_OAR›	91631482	-0.02793
27	oar3_OAR›	91640125	0.03378
27	oar3_OAR›	91645494	0.03378
27	oar3_OAR›	91650458	-0.02897
27	oar3_OAR›	91659259	-0.01225
27	oar3_OAR›	91660158	-0.03073
27	oar3_OAR›	91663590	-0.03124
27	oar3_OAR›	91664798	0.085255
27	oar3_OAR›	91669120	-0.01069
27	oar3_OAR›	91674097	0.051142
27	oar3_OAR›	91680790	-0.00355
27	oar3_OAR›	91683896	-0.00355
27	oar3_OAR›	91689035	-0.02618
27	oar3_OAR›	91689451	-0.02618
27	oar3_OAR›	91691723	-0.02618
27	oar3_OAR›	91692287	-0.02618
27	oar3_OAR›	91699215	-0.0281
27	oar3_OAR›	91699563	-0.03093
27	oar3_OAR›	91706193	0.14531
27	oar3_OAR›	91713867	-0.00996
27	oar3_OAR›	91715249	-0.02191
27	oar3_OAR›	91715833	0.128691
27	oar3_OAR›	91717292	0.116199
27	oar3_OAR›	91724896	-0.02191
27	oar3_OAR›	91724947	-0.02859
27	oar3_OAR›	91730821	0.06174
27	oar3_OAR›	91731089	-0.00767
27	oar3_OAR›	91736158	-0.01628
27	oar3_OAR›	91736245	0.004986
27	oar3_OAR›	91739057	0.010838
27	oar3_OAR›	91748012	0.115662
27	oar3_OAR›	91749810	0.059823
27	oar3_OAR›	91750407	0.115662
27	oar3_OAR›	91760356	-0.02415
27	oar3_OAR›	91760930	0.046694
27	oar3_OAR›	91763010	0.183417
27	oar3_OAR›	91771199	0.00826

27	oar3_OAR›	91776083	0.149033
27	oar3_OAR›	91797250	0.016368
27	oar3_OAR›	91797324	0.016368
27	oar3_OAR›	91803028	0.063524
27	oar3_OAR›	91805559	-0.01106
27	oar3_OAR›	91811640	0.047845
27	oar3_OAR›	91811725	-0.01515
27	oar3_OAR›	91824492	-0.01515
27	oar3_OAR›	91824806	-0.03036
27	oar3_OAR›	91825903	0.018645
27	oar3_OAR›	91827050	-0.02123
27	oar3_OAR›	91831809	0.007973
27	oar3_OAR›	91837387	0.043597
27	oar3_OAR›	91843786	0.00872
27	oar3_OAR›	91843835	0.006667
27	oar3_OAR›	91847563	-0.01551
27	oar3_OAR›	91849357	0.066751
27	oar3_OAR›	91858938	0.024378
27	oar3_OAR›	91864190	-0.00817
27	oar3_OAR›	91868734	-0.038
27	oar3_OAR›	91869033	-0.0308
27	oar3_OAR›	91877125	0.046963
27	oar3_OAR›	91881923	-0.0132
27	oar3_OAR›	91885528	-0.02327
27	oar3_OAR›	91885722	0.018027
27	oar3_OAR›	91905717	-0.01139
27	oar3_OAR›	91906529	-0.0168
27	oar3_OAR›	91912693	-0.02296
27	oar3_OAR›	91917446	0.043014
27	oar3_OAR›	91917579	-0.0168
27	oar3_OAR›	91927596	0.029325
27	oar3_OAR›	91938234	0.03039
27	oar3_OAR›	91939954	0.045804
27	oar3_OAR›	91941445	0.04976
27	oar3_OAR›	91950289	-0.01856
27	oar3_OAR›	91950729	-0.03509
27	oar3_OAR›	91951976	-0.03509
27	oar3_OAR›	91955562	-0.0092
27	oar3_OAR›	91983918	0.009985
27	oar3_OAR›	92016250	0.054415
27	oar3_OAR›	92023545	0.043609
27	oar3_OAR›	92026453	0.021882
27	oar3_OAR›	92027361	0.054415
27	oar3_OAR›	92033245	0.043609
27	oar3_OAR›	92035960	0.009633
27	oar3_OAR›	92038806	0.009633
27	oar3_OAR›	92039144	0.04878
27	oar3_OAR›	92045489	0.04878
27	oar3_OAR›	92050510	0.02101
27	oar3_OAR›	92050959	0.02101
27	oar3_OAR›	92060972	0.02101
27	oar3_OAR›	92061499	-0.00158
27	oar3_OAR›	92064944	0.02101
27	oar3_OAR›	92075092	0.032608

27	oar3_OAR)	92077656	-0.02528
27	oar3_OAR)	92088561	-0.02917
27	oar3_OAR)	92089702	-0.03392
27	oar3_OAR)	92094344	-0.02258
27	oar3_OAR)	92095421	-0.02258
27	oar3_OAR)	92099560	0.040387
27	oar3_OAR)	92110461	0.032491
27	oar3_OAR)	92111797	0.02062
27	oar3_OAR)	92118089	-0.0051
27	oar3_OAR)	92118330	0.025372
27	oar3_OAR)	92120581	-0.02652
27	oar3_OAR)	92124161	-0.03825
27	oar3_OAR)	92128342	0.025372
27	oar3_OAR)	92128953	-0.03825
27	oar3_OAR)	92129195	-0.03552
27	oar3_OAR)	92129729	0.023823
27	oar3_OAR)	92134871	-0.03764
27	oar3_OAR)	92148761	0.158095
27	oar3_OAR)	92149493	0.158095
27	oar3_OAR)	92153548	0.147855
27	oar3_OAR)	92165215	0.120915
27	oar3_OAR)	92170828	0.021314
27	oar3_OAR)	92178747	-0.03439
27	oar3_OAR)	92181252	-0.01824
27	oar3_OAR)	92191003	-0.01824
27	oar3_OAR)	92202971	-0.01069
27	oar3_OAR)	92206270	-0.02242
27	oar3_OAR)	92207874	-0.00528
27	oar3_OAR)	92208256	0.000605
27	oar3_OAR)	92216266	0.113191
27	oar3_OAR)	92226063	0.140791
27	oar3_OAR)	92226553	0.140791
27	oar3_OAR)	92226948	-0.02242
27	oar3_OAR)	92227049	-0.02242
27	oar3_OAR)	92232502	0.079265
27	oar3_OAR)	92239618	0.124735
27	oar3_OAR)	92240710	-0.0177
27	oar3_OAR)	92251004	-0.03675
27	oar3_OAR)	92253237	-0.01745
27	oar3_OAR)	92260894	0.092841
27	oar3_OAR)	92266071	-0.00673
27	oar3_OAR)	92269821	-0.02974
27	oar3_OAR)	92278738	0.115867
27	oar3_OAR)	92279300	0.034512
27	oar3_OAR)	92282593	-0.02921
27	oar3_OAR)	92283539	0.037009
27	oar3_OAR)	92286106	-0.01519
27	oar3_OAR)	92291562	0.079911
27	oar3_OAR)	92292055	0.108424
27	oar3_OAR)	92294316	-0.02251
27	oar3_OAR)	92297251	0.00834
27	oar3_OAR)	92311525	0.041634
27	oar3_OAR)	92316127	0.030586
27	oar3_OAR)	92320663	NA

27	oar3_OAR>	92323979	-0.02961
27	oar3_OAR>	92324694	-0.04144
27	oar3_OAR>	92329431	-0.02961
27	oar3_OAR>	92329720	-0.02961
27	oar3_OAR>	92336096	-0.01995
27	oar3_OAR>	92337143	0.046169
27	oar3_OAR>	92337965	-0.01995
27	oar3_OAR>	92338056	-0.02918
27	oar3_OAR>	92353461	-0.02419
27	oar3_OAR>	92354720	-0.03047
27	oar3_OAR>	92355159	-0.0109
27	oar3_OAR>	92356920	-0.02444
27	oar3_OAR>	92370181	-0.01553
27	oar3_OAR>	92374800	-0.02786
27	oar3_OAR>	92378232	-0.03439
27	oar3_OAR>	92383969	-0.0347
27	oar3_OAR>	92384036	0.069904
27	oar3_OAR>	92386783	0.007835
27	oar3_OAR>	92396165	0.016891
27	oar3_OAR>	92398573	-0.00157
27	oar3_OAR>	92399453	-0.00462
27	oar3_OAR>	92406494	-0.00462
27	oar3_OAR>	92407736	0.002815
27	oar3_OAR>	92407829	-0.03098
27	oar3_OAR>	92409298	-0.00898
27	oar3_OAR>	92413354	0.002815
27	oar3_OAR>	92417901	0.03637
27	oar3_OAR>	92418480	-0.00462
27	oar3_OAR>	92424013	-0.03664
27	oar3_OAR>	92431318	-0.03511
27	oar3_OAR>	92431403	0.008934
27	oar3_OAR>	92439685	0.008934
27	oar3_OAR>	92445583	-0.00462
27	oar3_OAR>	92450605	-0.00462
27	oar3_OAR>	92461385	-0.04243
27	oar3_OAR>	92464640	-0.03509
27	oar3_OAR>	92476551	-0.01303
27	oar3_OAR>	92481694	0.015985
27	oar3_OAR>	92489541	0.022722
27	oar3_OAR>	92492006	0.021756
27	oar3_OAR>	92497423	-0.01642
27	oar3_OAR>	92502439	-0.03631
27	oar3_OAR>	92503079	0.027638
27	oar3_OAR>	92510455	-0.02611
27	oar3_OAR>	92511384	NA
27	oar3_OAR>	92526413	-0.01069
27	oar3_OAR>	92526987	-0.03234
27	oar3_OAR>	92542732	0.095171
27	oar3_OAR>	92556080	0.039032
27	oar3_OAR>	92561446	0.104313
27	oar3_OAR>	92567481	NA
27	oar3_OAR>	92568313	0.104313
27	oar3_OAR>	92570683	0.128235
27	oar3_OAR>	92576138	0.106798

27	oar3_OAR›	92577313	0.128235
27	oar3_OAR›	92597509	0.016847
27	oar3_OAR›	92600708	-0.0206
27	oar3_OAR›	92607405	-0.01954
27	oar3_OAR›	92609798	-0.03231
27	oar3_OAR›	92617750	-0.00604
27	oar3_OAR›	92618335	-0.02204
27	oar3_OAR›	92625918	-0.02008
27	oar3_OAR›	92630413	-0.03295
27	oar3_OAR›	92631118	0.020992
27	oar3_OAR›	92636758	-0.03295
27	oar3_OAR›	92642801	-0.00955
27	oar3_OAR›	92643366	-0.01942
27	oar3_OAR›	92648367	-0.03439
27	oar3_OAR›	92657206	-0.03208
27	oar3_OAR›	92660041	0.0051
27	oar3_OAR›	92661404	-0.02856
27	oar3_OAR›	92720776	-0.03151
27	oar3_OAR›	92723686	-0.03151
27	oar3_OAR›	92728125	-0.03151
27	oar3_OAR›	92731894	-0.03665
27	oar3_OAR›	92735638	-0.03384
27	oar3_OAR›	92738982	-0.03497
27	oar3_OAR›	92741211	-0.02709
27	oar3_OAR›	92749173	-0.03688
27	oar3_OAR›	92749239	-0.02113
27	oar3_OAR›	92749586	-0.03688
27	oar3_OAR›	92756104	-0.03724
27	oar3_OAR›	92759600	-0.02158
27	oar3_OAR›	92759709	-0.02709
27	oar3_OAR›	92766102	-0.03688
27	oar3_OAR›	92767326	-0.02158
27	oar3_OAR›	92767789	-0.02158
27	oar3_OAR›	92779100	-0.02709
27	oar3_OAR›	92779127	-0.02709
27	oar3_OAR›	92790289	-0.00048
27	oar3_OAR›	92792221	-0.02731
27	oar3_OAR›	92807258	-0.0184
27	oar3_OAR›	92808528	0.014979
27	oar3_OAR›	92814257	-0.00462
27	oar3_OAR›	92818897	0.109621
27	oar3_OAR›	92819498	0.109621
27	oar3_OAR›	92823334	0.109621
27	oar3_OAR›	92824279	0.109621
27	oar3_OAR›	92829559	NA
27	oar3_OAR›	92831577	0.069468
27	oar3_OAR›	92831854	0.109621
27	oar3_OAR›	92835455	0.109621
27	oar3_OAR›	92838411	-0.01644
27	oar3_OAR›	92842055	0.017279
27	oar3_OAR›	92843755	0.021197
27	oar3_OAR›	92860673	-0.01642
27	oar3_OAR›	92865754	-0.03498
27	oar3_OAR›	92879374	-0.01218

27	oar3_OAR›	92903490	-0.01069
27	oar3_OAR›	92917137	-0.01423
27	oar3_OAR›	92922320	-0.01737
27	oar3_OAR›	92930270	-0.0178
27	oar3_OAR›	92933702	-0.02566
27	oar3_OAR›	92938199	-0.03189
27	oar3_OAR›	92942932	-0.03189
27	oar3_OAR›	92944433	-0.03189
27	oar3_OAR›	92946786	0.037433
27	oar3_OAR›	92983171	0.031477
27	oar3_OAR›	92992248	0.119533
27	oar3_OAR›	92998465	0.025748
27	oar3_OAR›	92998606	0.025748
27	oar3_OAR›	93005193	0.016478
27	oar3_OAR›	93017494	0.015215
27	oar3_OAR›	93022986	0.015215
27	oar3_OAR›	93026503	0.016478
27	oar3_OAR›	93028488	0.010733
27	oar3_OAR›	93032683	0.030356
27	oar3_OAR›	93033738	0.002439
27	oar3_OAR›	93045739	0.007487
27	oar3_OAR›	93045881	0.007487
27	oar3_OAR›	93072326	-0.00193
27	oar3_OAR›	93073687	0.026654
27	oar3_OAR›	93075865	0.026654
27	oar3_OAR›	93081275	-0.0236
27	oar3_OAR›	93087390	0.026654
27	oar3_OAR›	93087817	-0.02724
27	oar3_OAR›	93091930	0.029545
27	oar3_OAR›	93097832	-0.00796
27	oar3_OAR›	93097961	-0.02812
27	oar3_OAR›	93099814	-0.02168
27	oar3_OAR›	93103252	-0.03171
27	oar3_OAR›	93106494	0.000889
27	oar3_OAR›	93133362	-0.02429
27	oar3_OAR›	93143851	-0.00735
27	oar3_OAR›	93146153	-0.01682
27	oar3_OAR›	93146738	-0.00961
27	oar3_OAR›	93147855	-0.01069
27	oar3_OAR›	93153217	-0.01069
27	oar3_OAR›	93159259	-0.02087
27	oar3_OAR›	93160909	-0.01069
27	oar3_OAR›	93161224	-0.00936
27	oar3_OAR›	93169189	-0.01517
27	oar3_OAR›	93171774	-0.03287
27	oar3_OAR›	93174576	NA
27	oar3_OAR›	93180328	0.024024
27	oar3_OAR›	93182098	-0.02387
27	oar3_OAR›	93186739	-0.01377
27	oar3_OAR›	93194897	-0.02387
27	oar3_OAR›	93197963	-0.02765
27	oar3_OAR›	93199152	-0.03028
27	oar3_OAR›	93199243	-0.03028
27	oar3_OAR›	93212966	-0.0144

27	oar3_OAR>	93213083	-0.0144
27	oar3_OAR>	93214276	0.007039
27	oar3_OAR>	93217878	0.007039
27	oar3_OAR>	93227421	0.007039
27	oar3_OAR>	93229696	0.044724
27	oar3_OAR>	93252735	-0.0255
27	oar3_OAR>	93257986	-0.02759
27	oar3_OAR>	93283230	NA
27	oar3_OAR>	93286898	NA
27	oar3_OAR>	93288590	NA
27	oar3_OAR>	93289653	NA
27	oar3_OAR>	93300185	0.10849
27	oar3_OAR>	93302794	0.029744
27	oar3_OAR>	93304422	0.00215
27	oar3_OAR>	93311558	0.079826
27	oar3_OAR>	93315062	0.00215
27	oar3_OAR>	93317815	0.039481
27	oar3_OAR>	93320522	0.039481
27	oar3_OAR>	93323312	0.025477
27	oar3_OAR>	93326237	0.039481
27	oar3_OAR>	93331134	0.015698
27	oar3_OAR>	93340157	0.00489
27	oar3_OAR>	93341287	-0.0211
27	oar3_OAR>	93344769	-0.02517
27	oar3_OAR>	93350299	0.044724
27	oar3_OAR>	93353935	-0.02517
27	oar3_OAR>	93375885	-0.02916
27	oar3_OAR>	93377710	-0.03681
27	oar3_OAR>	93379941	0.008723
27	oar3_OAR>	93380744	0.008723
27	oar3_OAR>	93387018	0.000889
27	oar3_OAR>	93387274	-0.03852
27	oar3_OAR>	93388621	-0.03569
27	oar3_OAR>	93389807	-0.03805
27	oar3_OAR>	93393297	0.008723
27	oar3_OAR>	93399730	0.025345
27	oar3_OAR>	93400009	-0.01826
27	oar3_OAR>	93403180	0.012464
27	oar3_OAR>	93414987	-0.03979
27	oar3_OAR>	93420581	-0.03979
27	oar3_OAR>	93424681	-0.0213
27	oar3_OAR>	93429775	-0.03979
27	oar3_OAR>	93430595	-0.02913
27	oar3_OAR>	93440819	-0.03979
27	oar3_OAR>	93441212	-0.02913
27	oar3_OAR>	93441450	-0.02735
27	oar3_OAR>	93454924	-0.02972
27	oar3_OAR>	93460501	-0.03404
27	oar3_OAR>	93463014	-0.04265
27	oar3_OAR>	93465564	-0.03806
27	oar3_OAR>	93472447	-0.02652
27	oar3_OAR>	93490617	-0.01368
27	oar3_OAR>	93491071	-0.03459
27	oar3_OAR>	93492015	-0.04185

27	oar3_OAR›	93511607	0.012112
27	oar3_OAR›	93517049	0.008808
27	oar3_OAR›	93530388	-0.00979
27	oar3_OAR›	93535758	0.053235
27	oar3_OAR›	93536434	0.053235
27	oar3_OAR›	93551215	0.085457
27	oar3_OAR›	93554000	0.076282
27	oar3_OAR›	93562570	0.048875
27	oar3_OAR›	93564487	0.109252
27	oar3_OAR›	93571897	0.019617
27	oar3_OAR›	93572501	0.024638
27	oar3_OAR›	93580721	0.019617
27	oar3_OAR›	93581359	0.048875
27	oar3_OAR›	93585260	0.024638
27	oar3_OAR›	93589022	-0.0025
27	oar3_OAR›	93593258	0.006414
27	oar3_OAR›	93596578	0.007024
27	oar3_OAR›	93597784	0.015055
27	oar3_OAR›	93598185	-0.01069
27	oar3_OAR›	93603503	0.029828
27	oar3_OAR›	93617380	0.004078
27	oar3_OAR›	93618795	-0.03113
27	oar3_OAR›	93620832	-0.0268
27	oar3_OAR›	93626597	-0.0268
27	oar3_OAR›	93629078	-0.02237
27	oar3_OAR›	93630874	0.050638
27	oar3_OAR›	93640542	-0.02237
27	oar3_OAR›	93643999	-0.0268
27	oar3_OAR›	93652851	0.00839
27	oar3_OAR›	93660205	0.037044
27	oar3_OAR›	93669700	0.0365
27	oar3_OAR›	93670431	-0.0289
27	oar3_OAR›	93673670	0.050638
27	oar3_OAR›	93675013	0.050638
27	oar3_OAR›	93681254	-0.03332
27	oar3_OAR›	93689414	-0.02756
27	oar3_OAR›	93689976	0.000963
27	oar3_OAR›	93695019	0.023325
27	oar3_OAR›	93695194	-0.03737
27	oar3_OAR›	93695664	-0.01628
27	oar3_OAR›	93699174	0.023064
27	oar3_OAR›	93701641	-0.03392
27	oar3_OAR›	93719077	-0.00404
27	oar3_OAR›	93719271	0.071509
27	oar3_OAR›	93724126	-0.00404
27	oar3_OAR›	93729422	0.042308
27	oar3_OAR›	93732347	0.071509
27	oar3_OAR›	93733209	0.042308
27	oar3_OAR›	93741500	0.040691
27	oar3_OAR›	93747677	0.084756
27	oar3_OAR›	93747902	0.084756
27	oar3_OAR›	93761549	0.025736
27	oar3_OAR›	93765408	-0.02681
27	oar3_OAR›	93765770	0.011755

27	oar3_OAR›	93770588	-0.01225
27	oar3_OAR›	93776854	0.028383
27	oar3_OAR›	93780605	0.097681
27	oar3_OAR›	93794116	0.091523
27	oar3_OAR›	93794369	-0.00836
27	oar3_OAR›	93794802	0.091523
27	oar3_OAR›	93796281	0.028383
27	oar3_OAR›	93805816	0.014143
27	oar3_OAR›	93806350	NA
27	oar3_OAR›	93807091	0.021213
27	oar3_OAR›	93810997	0.004918
27	oar3_OAR›	93830455	0.014143
27	oar3_OAR›	93840567	0.03378
27	oar3_OAR›	93850806	0.03378
27	oar3_OAR›	93871953	-0.03699
27	oar3_OAR›	93872294	0.026333
27	oar3_OAR›	93873756	0.092501
27	oar3_OAR›	93882242	0.007039
27	oar3_OAR›	93884053	-0.02652
27	oar3_OAR›	93887713	0.190436
27	oar3_OAR›	93893099	-0.00031
27	oar3_OAR›	93893508	-0.00766
27	oar3_OAR›	93900645	-0.01194
27	oar3_OAR›	93905368	0.050459
27	oar3_OAR›	93906912	-0.00961
27	oar3_OAR›	93907386	-0.01496
27	oar3_OAR›	93911738	-0.0147
27	oar3_OAR›	93920773	0.031432
27	oar3_OAR›	93924585	-0.02358
27	oar3_OAR›	93927927	-0.01737
27	oar3_OAR›	93934883	-0.01161
27	oar3_OAR›	93935950	0.021662
27	oar3_OAR›	93938700	-0.03679
27	oar3_OAR›	93952313	0.009985
27	oar3_OAR›	93952919	0.049827
27	oar3_OAR›	93954648	-0.0002
27	oar3_OAR›	93969153	-0.01236
27	oar3_OAR›	93988652	-0.00705
27	oar3_OAR›	93992629	0.034975
27	oar3_OAR›	94012749	-0.01437
27	oar3_OAR›	94013614	-0.01437
27	oar3_OAR›	94021434	0.076164
27	oar3_OAR›	94023916	-0.02759
27	oar3_OAR›	94035878	0.150317
27	oar3_OAR›	94042989	0.174129
27	oar3_OAR›	94043489	0.00481
27	oar3_OAR›	94051047	0.143962
27	oar3_OAR›	94051308	-0.00462
27	oar3_OAR›	94062454	0.174129
27	oar3_OAR›	94065179	0.186985
27	oar3_OAR›	94077259	-0.01069
27	oar3_OAR›	94088648	0.212161
27	oar3_OAR›	94092948	0.044724
27	oar3_OAR›	94106982	0.118612

27	oar3_OAR⟩	94123410	0.193953
27	oar3_OAR⟩	94123535	0.193953
27	oar3_OAR⟩	94132665	0.019861
27	oar3_OAR⟩	94140439	0.019861
27	oar3_OAR⟩	94144059	-0.02292
27	oar3_OAR⟩	94158968	0.019861
27	oar3_OAR⟩	94181610	0.042803
27	oar3_OAR⟩	94192112	0.049204
27	oar3_OAR⟩	94192294	0.00177
27	oar3_OAR⟩	94194624	0.022722
27	oar3_OAR⟩	94206877	0.041488
27	oar3_OAR⟩	94220100	0.011115
27	oar3_OAR⟩	94228197	-0.00597
27	oar3_OAR⟩	94242017	0.102573
27	oar3_OAR⟩	94250546	-0.02766
27	oar3_OAR⟩	94252764	0.04198
27	oar3_OAR⟩	94254248	-0.00508
27	oar3_OAR⟩	94259589	-0.00508
27	oar3_OAR⟩	94261324	0.019388
27	oar3_OAR⟩	94267440	-0.00508
27	oar3_OAR⟩	94267719	-0.01762
27	oar3_OAR⟩	94271812	-0.01208
27	oar3_OAR⟩	94272892	0.030048
27	oar3_OAR⟩	94273112	0.047083
27	oar3_OAR⟩	94280337	-0.00991
27	oar3_OAR⟩	94289912	0.036682
27	oar3_OAR⟩	94297453	-0.01574
27	oar3_OAR⟩	94303028	0.022514
27	oar3_OAR⟩	94303801	0.041992
27	oar3_OAR⟩	94310967	0.003128
27	oar3_OAR⟩	94319590	0.102589
27	oar3_OAR⟩	94323969	0.056582
27	oar3_OAR⟩	94325698	0.102589
27	oar3_OAR⟩	94333110	0.006682
27	oar3_OAR⟩	94333735	0.018913
27	oar3_OAR⟩	94335147	-0.00665
27	oar3_OAR⟩	94340248	-0.02014
27	oar3_OAR⟩	94344797	0.007736
27	oar3_OAR⟩	94345596	-0.01378
27	oar3_OAR⟩	94353212	0.023831
27	oar3_OAR⟩	94378705	0.028747
27	oar3_OAR⟩	94403528	-0.01567
27	oar3_OAR⟩	94418760	0.044241
27	oar3_OAR⟩	94423805	0.044241
27	oar3_OAR⟩	94434657	-0.00791
27	oar3_OAR⟩	94435247	-0.01262
27	oar3_OAR⟩	94435336	0.01915
27	oar3_OAR⟩	94436824	-0.01585
27	oar3_OAR⟩	94447209	-0.00791
27	oar3_OAR⟩	94447616	-0.00791
27	oar3_OAR⟩	94447943	0.01915
27	oar3_OAR⟩	94466458	0.002815
27	oar3_OAR⟩	94466880	0.002815
27	oar3_OAR⟩	94467636	0.002815

27	oar3_OAR›	94475716	-0.01069
27	oar3_OAR›	94480775	-0.00791
27	oar3_OAR›	94495264	-0.02049
27	oar3_OAR›	94499806	-0.01088
27	oar3_OAR›	94500170	0.036117
27	oar3_OAR›	94523845	0.028743
27	oar3_OAR›	94528796	0.068143
27	oar3_OAR›	94534302	0.028743
27	oar3_OAR›	94539707	0.103985
27	oar3_OAR›	94545368	0.11769
27	oar3_OAR›	94555995	-0.02645
27	oar3_OAR›	94557798	0.034323
27	oar3_OAR›	94558548	0.148588
27	oar3_OAR›	94563903	0.076979
27	oar3_OAR›	94569281	0.099613
27	oar3_OAR›	94569467	0.042659
27	oar3_OAR›	94580958	0.073461
27	oar3_OAR›	94581911	-0.01069
27	oar3_OAR›	94583092	0.001575
27	oar3_OAR›	94589280	0.049331
27	oar3_OAR›	94590021	0.073461
27	oar3_OAR›	94609058	0.000135
27	oar3_OAR›	94609428	0.003874
27	oar3_OAR›	94614193	0.021481
27	oar3_OAR›	94630989	-0.00986
27	oar3_OAR›	94635982	-0.013
27	oar3_OAR›	94637480	0.040142
27	oar3_OAR›	94638345	-0.02639
27	oar3_OAR›	94642160	0.087305
27	oar3_OAR›	94649466	0.070884
27	oar3_OAR›	94655693	0.066899
27	oar3_OAR›	94660179	-0.02031
27	oar3_OAR›	94660970	0.085121
27	oar3_OAR›	94662324	0.018032
27	oar3_OAR›	94674401	0.085121
27	oar3_OAR›	94674867	0.118432
27	oar3_OAR›	94678535	0.122325
27	oar3_OAR›	94682916	0.0026
27	oar3_OAR›	94688108	-0.02031
27	oar3_OAR›	94700436	0.020865
27	oar3_OAR›	94700506	0.020865
27	oar3_OAR›	94700517	0.085121
27	oar3_OAR›	94710689	0.053231
27	oar3_OAR›	94711499	-0.01885
27	oar3_OAR›	94731987	0.000135
27	oar3_OAR›	94740710	-0.01303
27	oar3_OAR›	94745618	-0.00562
27	oar3_OAR›	94746655	0.028404
27	oar3_OAR›	94749710	0.016301
27	oar3_OAR›	94758201	-0.00335
27	oar3_OAR›	94758301	-0.0321
27	oar3_OAR›	94761390	0.019393
27	oar3_OAR›	94762646	0.019393
27	oar3_OAR›	94768949	0.014419

27	oar3_OAR〉	94773939	0.019393
27	oar3_OAR〉	94774011	-0.03185
27	oar3_OAR〉	94775585	-0.01846
27	oar3_OAR〉	94783200	-0.01491
27	oar3_OAR〉	94785679	0.062709
27	oar3_OAR〉	94786208	0.062709
27	oar3_OAR〉	94786627	0.021081
27	oar3_OAR〉	94796085	0.098072
27	oar3_OAR〉	94796303	0.060339
27	oar3_OAR〉	94803961	0.03117
27	oar3_OAR〉	94814138	-0.01612
27	oar3_OAR〉	94814219	-0.01612
27	oar3_OAR〉	94814416	-0.01612
27	oar3_OAR〉	94822312	-0.02787
27	oar3_OAR〉	94833525	-0.04007
27	oar3_OAR〉	94840782	-0.02785
27	oar3_OAR〉	94841982	-0.02724
27	oar3_OAR〉	94844243	0.022123
27	oar3_OAR〉	94851075	-0.01841
27	oar3_OAR〉	94852198	-0.01841
27	oar3_OAR〉	94863677	-0.0185
27	oar3_OAR〉	94890127	-0.02114
27	oar3_OAR〉	94902254	0.053231
27	oar3_OAR〉	94905709	-0.01428
27	oar3_OAR〉	94908814	0.08472
27	oar3_OAR〉	94925690	-0.01698
27	oar3_OAR〉	94935619	0.017085
27	oar3_OAR〉	94935853	0.082589
27	oar3_OAR〉	94949481	0.013352
27	oar3_OAR〉	94950270	0.171324
27	oar3_OAR〉	94954255	0.059901
27	oar3_OAR〉	94954441	0.059901
27	oar3_OAR〉	94961256	0.017085
27	oar3_OAR〉	94964633	0.132616
27	oar3_OAR〉	94965602	0.051379
27	oar3_OAR〉	94967457	0.017085
27	oar3_OAR〉	94970453	0.078974
27	oar3_OAR〉	94974744	0.017085
27	oar3_OAR〉	94975392	-0.00773
27	oar3_OAR〉	94976354	0.327217
27	oar3_OAR〉	94981432	0.263342
27	oar3_OAR〉	94983917	0.065541
27	oar3_OAR〉	94986810	0.022164
27	oar3_OAR〉	94992824	0.152378
27	oar3_OAR〉	94993025	0.152139
27	oar3_OAR〉	94993231	0.18159
27	oar3_OAR〉	94993410	0.18159
27	oar3_OAR〉	95005171	0.026698
27	oar3_OAR〉	95006294	0.122305
27	oar3_OAR〉	95007058	0.034553
27	oar3_OAR〉	95019864	-0.01498
27	oar3_OAR〉	95021956	0.218973
27	oar3_OAR〉	95026298	0.210236
27	oar3_OAR〉	95030394	0.160134

27	oar3_OAR›	95037497	-0.00287
27	oar3_OAR›	95043612	NA
27	oar3_OAR›	95050840	-0.0385
27	oar3_OAR›	95053570	NA
27	oar3_OAR›	95058409	-0.04215
27	oar3_OAR›	95059820	-0.03909
27	oar3_OAR›	95062939	-0.0355
27	oar3_OAR›	95068572	-0.0355
27	oar3_OAR›	95068745	-0.0355
27	oar3_OAR›	95072250	-0.03909
27	oar3_OAR›	95079104	-0.0385
27	oar3_OAR›	95087139	-0.0355
27	oar3_OAR›	95088608	-0.0385
27	oar3_OAR›	95089388	-0.0385
27	oar3_OAR›	95099532	0.110054
27	oar3_OAR›	95102347	0.22501
27	oar3_OAR›	95106904	0.130925
27	oar3_OAR›	95118672	0.035747
27	oar3_OAR›	95123548	-0.00406
27	oar3_OAR›	95127991	-0.03236
27	oar3_OAR›	95134800	0.024356
27	oar3_OAR›	95138034	0.117943
27	oar3_OAR›	95153784	0.131579
27	oar3_OAR›	95154809	-0.0114
27	oar3_OAR›	95155889	-0.03195
27	oar3_OAR›	95160180	0.187108
27	oar3_OAR›	95166567	0.137766
27	oar3_OAR›	95166981	0.137766
27	oar3_OAR›	95178363	-0.01978
27	oar3_OAR›	95183167	0.075073
27	oar3_OAR›	95193339	0.049003
27	oar3_OAR›	95194981	0.064374
27	oar3_OAR›	95200662	-0.00909
27	oar3_OAR›	95206813	0.141506
27	oar3_OAR›	95222635	0.103147
27	oar3_OAR›	95238792	0.071538
27	oar3_OAR›	95247666	-0.01069
27	oar3_OAR›	95252700	-0.01069
27	oar3_OAR›	95264028	0.071538
27	oar3_OAR›	95265126	0.071538
27	oar3_OAR›	95275346	0.111545
27	oar3_OAR›	95275592	0.211018
27	oar3_OAR›	95279302	0.172042
27	oar3_OAR›	95279478	0.028803
27	oar3_OAR›	95288725	0.031413
27	oar3_OAR›	95288960	0.138038
27	oar3_OAR›	95289669	0.010173
27	oar3_OAR›	95295623	0.009992
27	oar3_OAR›	95309916	0.015728
27	oar3_OAR›	95319448	0.011887
27	oar3_OAR›	95324169	0.011887
27	oar3_OAR›	95329841	0.090911
27	oar3_OAR›	95332214	0.072149
27	oar3_OAR›	95336950	-0.00945

27	oar3_OAR›	95351560	-0.00144
27	oar3_OAR›	95353205	0.022977
27	oar3_OAR›	95353603	0.022977
27	oar3_OAR›	95369780	0.022043
27	oar3_OAR›	95370752	-0.02804
27	oar3_OAR›	95375984	0.056734
27	oar3_OAR›	95387914	0.016866
27	oar3_OAR›	95391918	0.021577
27	oar3_OAR›	95397112	-0.02565
27	oar3_OAR›	95400388	0.033689
27	oar3_OAR›	95405361	-0.01751
27	oar3_OAR›	95405851	8.73E-05
27	oar3_OAR›	95421127	0.083437
27	oar3_OAR›	95436303	0.143829
27	oar3_OAR›	95442430	0.050192
27	oar3_OAR›	95456568	0.007039
27	oar3_OAR›	95465140	0.053353
27	oar3_OAR›	95465699	0.053353
27	oar3_OAR›	95470751	0.007039
27	oar3_OAR›	95472594	0.070968
27	oar3_OAR›	95474573	0.053353
27	oar3_OAR›	95481522	-0.00065
27	oar3_OAR›	95482942	0.009324
27	oar3_OAR›	95492295	-0.01549
27	oar3_OAR›	95492944	-0.01549
27	oar3_OAR›	95495209	-0.00258
27	oar3_OAR›	95495323	-0.01549
27	oar3_OAR›	95512182	0.01145
27	oar3_OAR›	95531216	0.01145
27	oar3_OAR›	95538357	0.034924
27	oar3_OAR›	95552013	0.003095
27	oar3_OAR›	95558030	0.01145
27	oar3_OAR›	95560763	-0.00362
27	oar3_OAR›	95562485	0.000363
27	oar3_OAR›	95563004	0.056063
27	oar3_OAR›	95563477	0.056063
27	oar3_OAR›	95574395	0.032582
27	oar3_OAR›	95574623	0.028803
27	oar3_OAR›	95574859	-0.0373
27	oar3_OAR›	95585589	-0.02085
27	oar3_OAR›	95587182	-0.01069
27	oar3_OAR›	95596175	-0.02842
27	oar3_OAR›	95597839	0.053795
27	oar3_OAR›	95602652	0.053235
27	oar3_OAR›	95603135	0.053795
27	oar3_OAR›	95606099	0.117383
27	oar3_OAR›	95609744	-0.00065
27	oar3_OAR›	95615961	-0.01315
27	oar3_OAR›	95616055	-0.02307
27	oar3_OAR›	95618551	-0.01315
27	oar3_OAR›	95618774	-0.00953
27	oar3_OAR›	95624051	-0.01496
27	oar3_OAR›	95626295	-0.00407
27	oar3_OAR›	95632854	-0.00953

27	oar3_OAR›	95633782	-0.01315
27	oar3_OAR›	95639241	0.005941
27	oar3_OAR›	95652191	0.012749
27	oar3_OAR›	95657954	-0.03509
27	oar3_OAR›	95663643	-0.03158
27	oar3_OAR›	95670658	-0.0148
27	oar3_OAR›	95677095	-0.03158
27	oar3_OAR›	95685311	-0.01655
27	oar3_OAR›	95694509	-0.0059
27	oar3_OAR›	95695568	-0.0059
27	oar3_OAR›	95701028	-0.01254
27	oar3_OAR›	95705772	-0.02188
27	oar3_OAR›	95725098	-0.00195
27	oar3_OAR›	95728314	0.01985
27	oar3_OAR›	95732379	0.043437
27	oar3_OAR›	95738918	-0.00751
27	oar3_OAR›	95741192	-0.00195
27	oar3_OAR›	95745211	-0.00581
27	oar3_OAR›	95750103	0.064242
27	oar3_OAR›	95761460	-0.02638
27	oar3_OAR›	95766561	0.077286
27	oar3_OAR›	95766661	0.051987
27	oar3_OAR›	95772402	0.077286
27	oar3_OAR›	95779740	0.061827
27	oar3_OAR›	95785143	0.14449
27	oar3_OAR›	95785499	0.14449
27	oar3_OAR›	95790912	0.105086
27	oar3_OAR›	95791899	-0.01208
27	oar3_OAR›	95792390	0.104279
27	oar3_OAR›	95798032	-0.00745
27	oar3_OAR›	95800396	-0.00745
27	oar3_OAR›	95802153	-0.00745
27	oar3_OAR›	95805922	0.017203
27	oar3_OAR›	95809885	0.017203
27	oar3_OAR›	95815272	-0.01737
27	oar3_OAR›	95822600	-0.02277
27	oar3_OAR›	95823548	0.054281
27	oar3_OAR›	95829406	0.034548
27	oar3_OAR›	95832435	-0.01957
27	oar3_OAR›	95837497	0.07935
27	oar3_OAR›	95844700	-0.02345
27	oar3_OAR›	95847855	-0.02807
27	oar3_OAR›	95851521	-0.00758
27	oar3_OAR›	95860818	0.044557
27	oar3_OAR›	95861702	-0.01789
27	oar3_OAR›	95864206	0.10283
27	oar3_OAR›	95867752	-0.01778
27	oar3_OAR›	95872146	0.012945
27	oar3_OAR›	95872705	-0.0321
27	oar3_OAR›	95880877	0.041495
27	oar3_OAR›	95890481	0.038627
27	oar3_OAR›	95898696	0.000708
27	oar3_OAR›	95899942	0.02221
27	oar3_OAR›	95906814	-0.00598

27	oar3_OAR'	95907261	0.036036
27	oar3_OAR'	95916969	0.003619
27	oar3_OAR'	95917486	-0.00508
27	oar3_OAR'	95918958	-0.00508
27	oar3_OAR'	95928171	-0.01588
27	oar3_OAR'	95929149	-0.02818
27	oar3_OAR'	95931802	-0.004
27	oar3_OAR'	95938528	0.02636
27	oar3_OAR'	95941076	0.069715
27	oar3_OAR'	95944171	0.086246
27	oar3_OAR'	95944529	0.04649
27	oar3_OAR'	95954270	0.027122
27	oar3_OAR'	95955932	-0.01024
27	oar3_OAR'	95965245	0.142378
27	oar3_OAR'	95968216	0.079488
27	oar3_OAR'	95969836	-0.01024
27	oar3_OAR'	95971065	0.02673
27	oar3_OAR'	95974429	0.109539
27	oar3_OAR'	95982816	0.029253
27	oar3_OAR'	95985140	0.086198
27	oar3_OAR'	95990380	0.01079
27	oar3_OAR'	95997503	-0.02555
27	oar3_OAR'	95998232	0.009612
27	oar3_OAR'	95998609	-0.03126
27	oar3_OAR'	96003156	-0.02555
27	oar3_OAR'	96009971	-0.02429
27	oar3_OAR'	96011866	-0.03126
27	oar3_OAR'	96015636	-0.0285
27	oar3_OAR'	96022773	-0.00969
27	oar3_OAR'	96036775	-0.03354
27	oar3_OAR'	96038569	-0.03488
27	oar3_OAR'	96043923	-0.03488
27	oar3_OAR'	96045303	-0.0292
27	oar3_OAR'	96046394	-0.01256
27	oar3_OAR'	96052083	0.01967
27	oar3_OAR'	96055410	0.01967
27	oar3_OAR'	96061773	0.044516
27	oar3_OAR'	96063512	-0.01423
27	oar3_OAR'	96068556	-0.00735
27	oar3_OAR'	96073635	0.012006
27	oar3_OAR'	96075394	0.045334
27	oar3_OAR'	96080607	-0.00181
27	oar3_OAR'	96082718	-0.01224
27	oar3_OAR'	96084761	0.001866
27	oar3_OAR'	96085995	0.020467
27	oar3_OAR'	96088303	0.026923
27	oar3_OAR'	96090563	0.011116
27	oar3_OAR'	96091215	-0.04094
27	oar3_OAR'	96098324	-0.00952
27	oar3_OAR'	96098692	0.011116
27	oar3_OAR'	96100804	0.028089
27	oar3_OAR'	96119267	0.035084
27	oar3_OAR'	96120890	-0.02806
27	oar3_OAR'	96123321	-0.02806

27	oar3_OAR>	96123531	-0.0184
27	oar3_OAR>	96131509	-0.0184
27	oar3_OAR>	96135290	-0.01299
27	oar3_OAR>	96141782	-0.02174
27	oar3_OAR>	96144587	-0.00335
27	oar3_OAR>	96144661	NA
27	oar3_OAR>	96146495	-0.00335
27	oar3_OAR>	96150410	-0.00335
27	oar3_OAR>	96152241	-0.02222
27	oar3_OAR>	96164222	-0.00356
27	oar3_OAR>	96164313	NA
27	oar3_OAR>	96165404	0.015332
27	oar3_OAR>	96172276	0.030102
27	oar3_OAR>	96174030	NA
27	oar3_OAR>	96185400	-0.01214
27	oar3_OAR>	96190261	NA
27	oar3_OAR>	96191201	-0.02645
27	oar3_OAR>	96200542	0.020848
27	oar3_OAR>	96202661	0.025283
27	oar3_OAR>	96206194	-0.01725
27	oar3_OAR>	96208892	0.005453
27	oar3_OAR>	96219677	-0.02926
27	oar3_OAR>	96220476	0.006819
27	oar3_OAR>	96221447	-0.01567
27	oar3_OAR>	96227091	-0.02593
27	oar3_OAR>	96234310	-0.02593
27	oar3_OAR>	96235747	0.048875
27	oar3_OAR>	96238438	-0.02593
27	oar3_OAR>	96239616	0.032461
27	oar3_OAR>	96246450	-0.01102
27	oar3_OAR>	96248556	-0.01745
27	oar3_OAR>	96259460	0.019791
27	oar3_OAR>	96265829	-0.01673
27	oar3_OAR>	96266231	0.075382
27	oar3_OAR>	96266425	-0.01673
27	oar3_OAR>	96276119	0.133305
27	oar3_OAR>	96276730	0.027713
27	oar3_OAR>	96288274	0.0935
27	oar3_OAR>	96292338	0.135374
27	oar3_OAR>	96295001	-0.01932
27	oar3_OAR>	96295045	-0.01995
27	oar3_OAR>	96297447	0.011984
27	oar3_OAR>	96303749	-0.0152
27	oar3_OAR>	96305966	-0.00462
27	oar3_OAR>	96306564	-0.00462
27	oar3_OAR>	96315549	-0.00823
27	oar3_OAR>	96318707	-0.00861
27	oar3_OAR>	96327361	0.034383
27	oar3_OAR>	96332943	-0.0337
27	oar3_OAR>	96337683	0.031127
27	oar3_OAR>	96340341	-0.01214
27	oar3_OAR>	96347521	-0.02742
27	oar3_OAR>	96350015	0.083816
27	oar3_OAR>	96357797	0.011067

27	oar3_OAR)	96359645	0.007022
27	oar3_OAR)	96360342	0.109621
27	oar3_OAR)	96365062	NA
27	oar3_OAR)	96370544	0.016855
27	oar3_OAR)	96370607	0.004822
27	oar3_OAR)	96371194	0.016855
27	oar3_OAR)	96381136	-0.02742
27	oar3_OAR)	96381213	0.032578
27	oar3_OAR)	96388042	0.032578
27	oar3_OAR)	96394070	-0.04167
27	oar3_OAR)	96395257	0.032578
27	oar3_OAR)	96398647	0.014686
27	oar3_OAR)	96399313	-0.02742
27	oar3_OAR)	96407069	-0.00997
27	oar3_OAR)	96409427	0.072717
27	oar3_OAR)	96409932	0.00161
27	oar3_OAR)	96411438	0.224682
27	oar3_OAR)	96413684	0.114367
27	oar3_OAR)	96420230	0.024639
27	oar3_OAR)	96421774	0.120818
27	oar3_OAR)	96429491	0.014681
27	oar3_OAR)	96433563	-0.02732
27	oar3_OAR)	96436716	-0.03152
27	oar3_OAR)	96441019	0.067123
27	oar3_OAR)	96442468	0.072212
27	oar3_OAR)	96447249	0.018559
27	oar3_OAR)	96450278	0.006667
27	oar3_OAR)	96450562	0.006667
27	oar3_OAR)	96456101	0.027964
27	oar3_OAR)	96459258	0.006667
27	oar3_OAR)	96460065	0.018559
27	oar3_OAR)	96461390	0.006667
27	oar3_OAR)	96469374	0.007022
27	oar3_OAR)	96478365	0.027964
27	oar3_OAR)	96481141	0.042308
27	oar3_OAR)	96482769	0.006667
27	oar3_OAR)	96484772	0.027964
27	oar3_OAR)	96487457	0.006667
27	oar3_OAR)	96493842	0.041197
27	oar3_OAR)	96498987	0.041333
27	oar3_OAR)	96502070	0.036197
27	oar3_OAR)	96511486	0.013077
27	oar3_OAR)	96516284	0.013077
27	oar3_OAR)	96519746	-0.02639
27	oar3_OAR)	96522735	-0.03371
27	oar3_OAR)	96524001	-0.00386
27	oar3_OAR)	96533392	0.001584
27	oar3_OAR)	96539616	0.001584
27	oar3_OAR)	96542585	0.001584
27	oar3_OAR)	96556583	0.001584
27	oar3_OAR)	96562659	0.001584
27	oar3_OAR)	96562757	0.001584
27	oar3_OAR)	96565941	-0.01138
27	oar3_OAR)	96573071	0.003055

27	oar3_OAR›	96575810	0.005857
27	oar3_OAR›	96580417	-0.01745
27	oar3_OAR›	96605033	-0.0108
27	oar3_OAR›	96606116	0.014159
27	oar3_OAR›	96607779	-0.01895
27	oar3_OAR›	96612834	-0.02766
27	oar3_OAR›	96613221	0.029527
27	oar3_OAR›	96618398	0.079293
27	oar3_OAR›	96620597	0.036722
27	oar3_OAR›	96621518	0.049228
27	oar3_OAR›	96621587	0.056256
27	oar3_OAR›	96628674	0.076432
27	oar3_OAR›	96631979	0.083511
27	oar3_OAR›	96634229	0.035435
27	oar3_OAR›	96638706	0.032203
27	oar3_OAR›	96643162	0.052562
27	oar3_OAR›	96646828	0.070714
27	oar3_OAR›	96647657	0.106461
27	oar3_OAR›	96649887	0.093889
27	oar3_OAR›	96649979	0.052769
27	oar3_OAR›	96658512	0.052769
27	oar3_OAR›	96662498	0.036873
27	oar3_OAR›	96664446	0.036873
27	oar3_OAR›	96670833	0.157258
27	oar3_OAR›	96672266	-0.02064
27	oar3_OAR›	96674209	0.055663
27	oar3_OAR›	96687604	0.074848
27	oar3_OAR›	96692736	-0.0236
27	oar3_OAR›	96696473	0.047083
27	oar3_OAR›	96697835	-0.0236
27	oar3_OAR›	96702295	0.055663
27	oar3_OAR›	96709057	-0.02465
27	oar3_OAR›	96710524	0.062369
27	oar3_OAR›	96715951	-0.02208
27	oar3_OAR›	96717243	0.004277
27	oar3_OAR›	96720439	0.011193
27	oar3_OAR›	96722182	-0.02415
27	oar3_OAR›	96724287	-0.02929
27	oar3_OAR›	96725530	0.090908
27	oar3_OAR›	96729087	0.034924
27	oar3_OAR›	96731435	0.034924
27	oar3_OAR›	96735541	0.078363
27	oar3_OAR›	96791079	-0.01528
27	oar3_OAR›	96791755	0.075547
27	oar3_OAR›	96797731	-0.01528
27	oar3_OAR›	96803825	-0.02151
27	oar3_OAR›	96806514	0.038715
27	oar3_OAR›	96809673	-0.02362
27	oar3_OAR›	96817913	-0.03562
27	oar3_OAR›	96834002	0.042803
27	oar3_OAR›	96834690	0.011261
27	oar3_OAR›	96843080	0.048314
27	oar3_OAR›	96843247	0.048314
27	oar3_OAR›	96845270	-0.01875

27	oar3_OAR›	96848373	0.09982
27	oar3_OAR›	96851196	0.011479
27	oar3_OAR›	96859043	8.73E-05
27	oar3_OAR›	96859350	-0.01875
27	oar3_OAR›	96874149	-0.01875
27	oar3_OAR›	96877822	8.73E-05
27	oar3_OAR›	96905412	0.038741
27	oar3_OAR›	96906751	0.007039
27	oar3_OAR›	96908632	0.015321
27	oar3_OAR›	96915995	0.00481
27	oar3_OAR›	96919155	0.00481
27	oar3_OAR›	96926305	-0.0213
27	oar3_OAR›	96932491	-0.03404
27	oar3_OAR›	96933846	-0.01702
27	oar3_OAR›	96935862	-0.0151
27	oar3_OAR›	96942617	-0.0151
27	oar3_OAR›	96942719	-0.03387
27	oar3_OAR›	96944016	0.024917
27	oar3_OAR›	96951340	-0.03198
27	oar3_OAR›	96955837	0.024917
27	oar3_OAR›	96959130	0.025546
27	oar3_OAR›	96959376	0.071166
27	oar3_OAR›	96964412	0.071166
27	oar3_OAR›	96967463	-0.03145
27	oar3_OAR›	96975525	0.086799
27	oar3_OAR›	96977440	-0.02063
27	oar3_OAR›	96977613	-0.02063
27	oar3_OAR›	96978673	0.010418
27	oar3_OAR›	96986774	0.011966
27	oar3_OAR›	96989409	0.102018
27	oar3_OAR›	96997111	-0.02185
27	oar3_OAR›	96997990	-0.02185
27	oar3_OAR›	97005800	-0.00735
27	oar3_OAR›	97012165	0.03648
27	oar3_OAR›	97014825	-0.02198
27	oar3_OAR›	97017212	-0.00791
27	oar3_OAR›	97026066	-0.03375
27	oar3_OAR›	97028680	-0.01304
27	oar3_OAR›	97029970	0.038407
27	oar3_OAR›	97031306	0.038407
27	oar3_OAR›	97033345	0.007241
27	oar3_OAR›	97041502	0.038815
27	oar3_OAR›	97042767	0.012458
27	oar3_OAR›	97048030	-0.01061
27	oar3_OAR›	97052158	0.05434
27	oar3_OAR›	97053066	0.05434
27	oar3_OAR›	97053221	-0.00606
27	oar3_OAR›	97057798	0.03046
27	oar3_OAR›	97063762	0.042071
27	oar3_OAR›	97063832	0.040006
27	oar3_OAR›	97065543	-0.02375
27	oar3_OAR›	97068936	-0.00181
27	oar3_OAR›	97075510	0.024716
27	oar3_OAR›	97079979	0.024716

27	oar3_OAR›	97087848	-0.02753
27	oar3_OAR›	97089851	-0.01649
27	oar3_OAR›	97091666	-0.03098
27	oar3_OAR›	97096966	-0.03098
27	oar3_OAR›	97097209	0.009982
27	oar3_OAR›	97100906	0.004003
27	oar3_OAR›	97102432	-0.03098
27	oar3_OAR›	97109751	-0.02576
27	oar3_OAR›	97116146	-0.03709
27	oar3_OAR›	97120471	-0.01063
27	oar3_OAR›	97120581	0.042471
27	oar3_OAR›	97127493	-0.00844
27	oar3_OAR›	97132384	0.09383
27	oar3_OAR›	97142586	0.091175
27	oar3_OAR›	97144787	0.041125
27	oar3_OAR›	97146309	0.091175
27	oar3_OAR›	97163890	0.014725
27	oar3_OAR›	97169737	-0.01063
27	oar3_OAR›	97174431	-0.00663
27	oar3_OAR›	97178089	0.004591
27	oar3_OAR›	97191357	0.004591
27	oar3_OAR›	97191499	-0.0211
27	oar3_OAR›	97197545	-0.01886
27	oar3_OAR›	97205348	0.035873
27	oar3_OAR›	97205448	0.030243
27	oar3_OAR›	97210367	0.044774
27	oar3_OAR›	97213141	-0.02783
27	oar3_OAR›	97221818	-0.01257
27	oar3_OAR›	97226782	-0.03439
27	oar3_OAR›	97229089	-0.00012
27	oar3_OAR›	97234676	-0.00321
27	oar3_OAR›	97237387	-0.00754
27	oar3_OAR›	97248463	-0.02221
27	oar3_OAR›	97249271	0.021215
27	oar3_OAR›	97249617	0.021215
27	oar3_OAR›	97257379	-0.03673
27	oar3_OAR›	97268348	0.12704
27	oar3_OAR›	97268912	0.014064
27	oar3_OAR›	97269762	-0.00082
27	oar3_OAR›	97273887	0.013952
27	oar3_OAR›	97283533	-0.03178
27	oar3_OAR›	97283923	0.003253
27	oar3_OAR›	97288823	0.041531
27	oar3_OAR›	97289777	0.039481
27	oar3_OAR›	97294540	0.042505
27	oar3_OAR›	97297320	0.035969
27	oar3_OAR›	97301021	0.017429
27	oar3_OAR›	97306583	-0.00424
27	oar3_OAR›	97310335	0.000605
27	oar3_OAR›	97311375	0.055372
27	oar3_OAR›	97317426	0.069468
27	oar3_OAR›	97327748	-0.02284
27	oar3_OAR›	97328305	-0.02304
27	oar3_OAR›	97334575	-0.02049

27	oar3_OAR›	97334658	-0.02049
27	oar3_OAR›	97344662	NA
27	oar3_OAR›	97346937	-0.00975
27	oar3_OAR›	97350142	NA
27	oar3_OAR›	97355097	0.011067
27	oar3_OAR›	97367078	-0.02599
27	oar3_OAR›	97378741	0.056082
27	oar3_OAR›	97380407	0.007039
27	oar3_OAR›	97388699	0.064284
27	oar3_OAR›	97390420	0.079969
27	oar3_OAR›	97396747	0.013849
27	oar3_OAR›	97397086	0.078045
27	oar3_OAR›	97402989	-0.00606
27	oar3_OAR›	97424510	-0.00615
27	oar3_OAR›	97427469	0.061699
27	oar3_OAR›	97436515	-0.01423
27	oar3_OAR›	97438442	-0.02565
27	oar3_OAR›	97439266	0.005092
27	oar3_OAR›	97439499	-0.0125
27	oar3_OAR›	97446173	0.000889
27	oar3_OAR›	97449887	-0.01644
27	oar3_OAR›	97460652	0.02243
27	oar3_OAR›	97460823	0.012855
27	oar3_OAR›	97460948	0.012855
27	oar3_OAR›	97475325	0.002158
27	oar3_OAR›	97475390	-0.02608
27	oar3_OAR›	97490202	0.005909
27	oar3_OAR›	97497605	-0.02926
27	oar3_OAR›	97502760	0.057126
27	oar3_OAR›	97504736	0.039481
27	oar3_OAR›	97505563	0.032192
27	oar3_OAR›	97527710	0.023411
27	oar3_OAR›	97530074	0.011257
27	oar3_OAR›	97532227	0.156052
27	oar3_OAR›	97534263	0.001263
27	oar3_OAR›	97539385	0.188224
27	oar3_OAR›	97541287	0.134483
27	oar3_OAR›	97561493	0.048585
27	oar3_OAR›	97564640	-0.0267
27	oar3_OAR›	97577914	-0.02917
27	oar3_OAR›	97583630	0.000889
27	oar3_OAR›	97588221	-0.00795
27	oar3_OAR›	97589771	-0.02534
27	oar3_OAR›	97592457	-0.02534
27	oar3_OAR›	97595381	0.02539
27	oar3_OAR›	97600358	-0.00658
27	oar3_OAR›	97600750	0.066366
27	oar3_OAR›	97610656	0.036401
27	oar3_OAR›	97611639	0.061491
27	oar3_OAR›	97617624	0.009041
27	oar3_OAR›	97617843	0.073321
27	oar3_OAR›	97620140	0.104525
27	oar3_OAR›	97624040	0.031225
27	oar3_OAR›	97632845	-0.0385

27	oar3_OAR›	97635800	0.105577
27	oar3_OAR›	97637313	0.105577
27	oar3_OAR›	97645073	-0.00313
27	oar3_OAR›	97646019	-0.00606
27	oar3_OAR›	97666355	0.051623
27	oar3_OAR›	97668967	0.051623
27	oar3_OAR›	97672061	0.005848
27	oar3_OAR›	97672814	0.0183
27	oar3_OAR›	97677267	0.031584
27	oar3_OAR›	97679721	0.051623
27	oar3_OAR›	97684739	-0.0141
27	oar3_OAR›	97686796	0.038001
27	oar3_OAR›	97687138	0.018728
27	oar3_OAR›	97690623	0.09378
27	oar3_OAR›	97697101	0.136159
27	oar3_OAR›	97697916	0.120974
27	oar3_OAR›	97702516	0.096731
27	oar3_OAR›	97708549	0.167322
27	oar3_OAR›	97708746	-0.0284
27	oar3_OAR›	97708963	0.046329
27	oar3_OAR›	97717656	-0.02709
27	oar3_OAR›	97723949	0.175777
27	oar3_OAR›	97725522	-0.00711
27	oar3_OAR›	97727544	-0.02774
27	oar3_OAR›	97730264	0.114907
27	oar3_OAR›	97740247	-0.01406
27	oar3_OAR›	97744832	0.022761
27	oar3_OAR›	97746801	0.022761
27	oar3_OAR›	97748738	0.108323
27	oar3_OAR›	97759049	-0.01567
27	oar3_OAR›	97759160	-0.02774
27	oar3_OAR›	97760393	-0.02774
27	oar3_OAR›	97760790	0.049381
27	oar3_OAR›	97767880	0.102821
27	oar3_OAR›	97774357	0.014354
27	oar3_OAR›	97774405	0.007039
27	oar3_OAR›	97786524	0.028357
27	oar3_OAR›	97792765	-0.02441
27	oar3_OAR›	97792982	-0.02441
27	oar3_OAR›	97796732	-0.01449
27	oar3_OAR›	97800518	0.000605
27	oar3_OAR›	97811076	0.010502
27	oar3_OAR›	97813268	-0.01186
27	oar3_OAR›	97814481	0.077463
27	oar3_OAR›	97819810	0.066918
27	oar3_OAR›	97820210	0.013065
27	oar3_OAR›	97825242	0.013065
27	oar3_OAR›	97831244	-0.01423
27	oar3_OAR›	97840437	-0.00751
27	oar3_OAR›	97842963	0.067788
27	oar3_OAR›	97845478	-0.00751
27	oar3_OAR›	97853167	0.081522
27	oar3_OAR›	97853295	0.055721
27	oar3_OAR›	97853867	0.081522

27	oar3_OAR›	97895052	0.024266
27	oar3_OAR›	97908104	0.055521
27	oar3_OAR›	97910321	0.081148
27	oar3_OAR›	97915468	0.052362
27	oar3_OAR›	97920033	0.077713
27	oar3_OAR›	97920795	0.091882
27	oar3_OAR›	97925225	0.02636
27	oar3_OAR›	97926181	0.028803
27	oar3_OAR›	97927289	0.018271
27	oar3_OAR›	97940907	0.018271
27	oar3_OAR›	97941057	-0.0059
27	oar3_OAR›	97941811	0.018271
27	oar3_OAR›	97945084	0.020878
27	oar3_OAR›	97946065	0.018271
27	oar3_OAR›	97951262	0.028803
27	oar3_OAR›	97955232	0.077713
27	oar3_OAR›	97960412	0.018271
27	oar3_OAR›	97967499	0.040756
27	oar3_OAR›	97980306	-0.02916
27	oar3_OAR›	97985926	0.040756
27	oar3_OAR›	97990144	-0.02818
27	oar3_OAR›	97990736	0.001049
27	oar3_OAR›	97992227	-0.02818
27	oar3_OAR›	97998085	-0.01097
27	oar3_OAR›	97999913	-0.00195
27	oar3_OAR›	98019449	0.028803
27	oar3_OAR›	98020045	0.009912
27	oar3_OAR›	98021949	0.025108
27	oar3_OAR›	98022012	0.03696
27	oar3_OAR›	98027217	-0.00057
27	oar3_OAR›	98034895	-0.01423
27	oar3_OAR›	98038430	-0.00637
27	oar3_OAR›	98042851	-0.00246
27	oar3_OAR›	98046720	-0.00137
27	oar3_OAR›	98048093	-0.01731
27	oar3_OAR›	98049194	-0.01605
27	oar3_OAR›	98049266	-0.01605
27	oar3_OAR›	98064294	-0.00574
27	oar3_OAR›	98101091	-0.00998
27	oar3_OAR›	98106333	-0.00998
27	oar3_OAR›	98115366	0.003335
27	oar3_OAR›	98115379	0.003335
27	oar3_OAR›	98115537	0.000363
27	oar3_OAR›	98118297	0.04444
27	oar3_OAR›	98123951	-0.01745
27	oar3_OAR›	98124452	0.05072
27	oar3_OAR›	98134942	-0.01745
27	oar3_OAR›	98148274	0.028803
27	oar3_OAR›	98148589	0.028803
27	oar3_OAR›	98185953	0.030172
27	oar3_OAR›	98195655	0.005018
27	oar3_OAR›	98205195	-0.02527
27	oar3_OAR›	98214367	-0.03739
27	oar3_OAR›	98214748	-0.03553

27	oar3_OAR›	98217968	-0.03565
27	oar3_OAR›	98227028	-0.02375
27	oar3_OAR›	98227161	0.013268
27	oar3_OAR›	98237668	0.073138
27	oar3_OAR›	98238491	0.021358
27	oar3_OAR›	98252245	-0.01787
27	oar3_OAR›	98256948	-0.01003
27	oar3_OAR›	98264057	0.000944
27	oar3_OAR›	98269775	-0.00126
27	oar3_OAR›	98277518	-0.00957
27	oar3_OAR›	98286745	0.104328
27	oar3_OAR›	98311118	NA
27	oar3_OAR›	98317040	0.007039
27	oar3_OAR›	98319809	0.002651
27	oar3_OAR›	98322263	-0.02566
27	oar3_OAR›	98322549	0.026278
27	oar3_OAR›	98326935	-0.01246
27	oar3_OAR›	98329846	-0.00083
27	oar3_OAR›	98333030	0.02818
27	oar3_OAR›	98334915	0.02818
27	oar3_OAR›	98338990	0.012017
27	oar3_OAR›	98351073	0.031085
27	oar3_OAR›	98355636	0.031087
27	oar3_OAR›	98356474	0.086793
27	oar3_OAR›	98359037	0.045963
27	oar3_OAR›	98364290	0.013236
27	oar3_OAR›	98365611	0.023496
27	oar3_OAR›	98370893	-0.03461
27	oar3_OAR›	98371397	0.000135
27	oar3_OAR›	98376282	-0.01281
27	oar3_OAR›	98378587	0.027046
27	oar3_OAR›	98378682	0.033182
27	oar3_OAR›	98381650	-0.01295
27	oar3_OAR›	98388850	-0.01202
27	oar3_OAR›	98397399	-0.02078
27	oar3_OAR›	98403652	0.039481
27	oar3_OAR›	98411413	0.025112
27	oar3_OAR›	98412122	0.025112
27	oar3_OAR›	98425508	0.023859
27	oar3_OAR›	98432586	0.091072
27	oar3_OAR›	98435774	0.042351
27	oar3_OAR›	98448399	0.007292
27	oar3_OAR›	98453648	0.018388
27	oar3_OAR›	98463882	0.07359
27	oar3_OAR›	98481413	-0.01069
27	oar3_OAR›	98485049	0.116979
27	oar3_OAR›	98490765	0.018804
27	oar3_OAR›	98504414	0.045834
27	oar3_OAR›	98505680	0.100273
27	oar3_OAR›	98515825	0.160077
27	oar3_OAR›	98524355	0.116845
27	oar3_OAR›	98524526	0.116845
27	oar3_OAR›	98532121	0.094229
27	oar3_OAR›	98533587	0.098599

27	oar3_OAR〉	98542219	-0.02926
27	oar3_OAR〉	98573163	-0.01906
27	oar3_OAR〉	98574106	-0.02347
27	oar3_OAR〉	98577608	-0.02713
27	oar3_OAR〉	98583528	-0.02505
27	oar3_OAR〉	98594420	0.011067
27	oar3_OAR〉	98599058	-0.01745
27	oar3_OAR〉	98601009	-0.02958
27	oar3_OAR〉	98601381	-0.02958
27	oar3_OAR〉	98607368	0.097086
27	oar3_OAR〉	98610130	-0.01737
27	oar3_OAR〉	98619881	0.017423
27	oar3_OAR〉	98621235	0.095434
27	oar3_OAR〉	98622820	0.017423
27	oar3_OAR〉	98636386	0.077381
27	oar3_OAR〉	98636555	0.077381
27	oar3_OAR〉	98637151	0.077381
27	oar3_OAR〉	98652565	0.193671
27	oar3_OAR〉	98682130	0.009815
27	oar3_OAR〉	98718302	-0.00188
27	oar3_OAR〉	98719832	0.017951
27	oar3_OAR〉	98747814	0.004281
27	oar3_OAR〉	98752538	-0.01356
27	oar3_OAR〉	98753620	-0.00915
27	oar3_OAR〉	98754454	-0.01567
27	oar3_OAR〉	98755650	-0.00777
27	oar3_OAR〉	98756467	0.074037
27	oar3_OAR〉	98816541	-0.02416
27	oar3_OAR〉	98818261	0.027463
27	oar3_OAR〉	98824917	-0.03059
27	oar3_OAR〉	98840832	-0.00053
27	oar3_OAR〉	98841541	-0.00053
27	oar3_OAR〉	98858298	0.116931
27	oar3_OAR〉	98875164	0.136868
27	oar3_OAR〉	98920290	0.112334
27	oar3_OAR〉	98921685	-0.02294
27	oar3_OAR〉	98922685	0.18366
27	oar3_OAR〉	98959704	0.064796
27	oar3_OAR〉	98971369	-0.00574
27	oar3_OAR〉	98972097	-0.00576
27	oar3_OAR〉	98974748	0.003806
27	oar3_OAR〉	98983586	-0.00881
27	oar3_OAR〉	98985496	0.003806
27	oar3_OAR〉	98988883	0.008867
27	oar3_OAR〉	98995543	-0.01092
27	oar3_OAR〉	99002885	-0.03181
27	oar3_OAR〉	99006999	-0.03181
27	oar3_OAR〉	99015381	0.00295
27	oar3_OAR〉	99019084	0.009608
27	oar3_OAR〉	99021350	-0.03869
27	oar3_OAR〉	99024742	-0.03198
27	oar3_OAR〉	99035644	0.02864
27	oar3_OAR〉	99043769	0.014344
27	oar3_OAR〉	99043858	0.014344

27	oar3_OAR›	99065386	0.015859
27	oar3_OAR›	99065534	-0.0164
27	oar3_OAR›	99072060	-0.01373
27	oar3_OAR›	99076229	0.065275
27	oar3_OAR›	99090278	0.030208
27	oar3_OAR›	99096383	0.029331
27	oar3_OAR›	99097809	0.044144
27	oar3_OAR›	99099766	-0.00351
27	oar3_OAR›	99102004	-0.02576
27	oar3_OAR›	99119626	0.018011
27	oar3_OAR›	99123939	0.147143
27	oar3_OAR›	99129694	0.030316
27	oar3_OAR›	99135841	0.082222
27	oar3_OAR›	99141316	0.007039
27	oar3_OAR›	99141887	-0.01972
27	oar3_OAR›	99146372	0.018084
27	oar3_OAR›	99153274	0.035962
27	oar3_OAR›	99161983	0.007039
27	oar3_OAR›	99162186	0.075682
27	oar3_OAR›	99163664	-0.02419
27	oar3_OAR›	99164178	0.076769
27	oar3_OAR›	99171805	0.08119
27	oar3_OAR›	99174315	0.014442
27	oar3_OAR›	99175158	0.035549
27	oar3_OAR›	99177680	0.026583
27	oar3_OAR›	99183999	0.008723
27	oar3_OAR›	99194325	0.072235
27	oar3_OAR›	99194541	0.126591
27	oar3_OAR›	99210837	-0.03123
27	oar3_OAR›	99210937	-0.00178
27	oar3_OAR›	99215093	-0.00178
27	oar3_OAR›	99226672	0.069047
27	oar3_OAR›	99235575	-0.03148
27	oar3_OAR›	99243307	-0.01838
27	oar3_OAR›	99246288	0.027218
27	oar3_OAR›	99247904	-0.04128
27	oar3_OAR›	99255800	-0.03688
27	oar3_OAR›	99258829	0.008295
27	oar3_OAR›	99258924	-0.00232
27	oar3_OAR›	99260850	0.065556
27	oar3_OAR›	99268359	0.013072
27	oar3_OAR›	99271102	0.018408
27	oar3_OAR›	99274220	0.020382
27	oar3_OAR›	99277596	0.007398
27	oar3_OAR›	99284572	0.007398
27	oar3_OAR›	99286981	0.00481
27	oar3_OAR›	99297549	-0.03531
27	oar3_OAR›	99305293	0.023148
27	oar3_OAR›	99313193	0.139297
27	oar3_OAR›	99317725	0.049739
27	oar3_OAR›	99322612	0.144645
27	oar3_OAR›	99333715	-0.02444
27	oar3_OAR›	99334351	-0.00799
27	oar3_OAR›	99359276	-0.00799

27	oar3_OAR}	99364540	-0.03225
27	oar3_OAR}	99384321	-0.03582
27	oar3_OAR}	99385842	0.013555
27	oar3_OAR}	99395034	0.025417
27	oar3_OAR}	99403400	-0.01761
27	oar3_OAR}	99403704	-0.03035
27	oar3_OAR}	99409363	-0.03035
27	oar3_OAR}	99414844	-0.03035
27	oar3_OAR}	99418004	-0.03035
27	oar3_OAR}	99424787	0.058388
27	oar3_OAR}	99426868	-0.02714
27	oar3_OAR}	99434987	-0.02714
27	oar3_OAR}	99435247	0.096086
27	oar3_OAR}	99435480	-0.02714
27	oar3_OAR}	99454114	-0.02899
27	oar3_OAR}	99454188	-0.00019
27	oar3_OAR}	99456519	-0.03203
27	oar3_OAR}	99460328	0.043437
27	oar3_OAR}	99466408	0.095259
27	oar3_OAR}	99470801	-0.01775
27	oar3_OAR}	99473115	-0.00257
27	oar3_OAR}	99475761	-0.00257
27	oar3_OAR}	99491304	0.110329
27	oar3_OAR}	99499627	0.110698
27	oar3_OAR}	99500854	0.036114
27	oar3_OAR}	99505504	0.035007
27	oar3_OAR}	99509578	0.035007
27	oar3_OAR}	99510365	-0.0177
27	oar3_OAR}	99517947	0.035007
27	oar3_OAR}	99521064	0.048281
27	oar3_OAR}	99521121	0.056976
27	oar3_OAR}	99530832	0.006254
27	oar3_OAR}	99533165	0.040809
27	oar3_OAR}	99533698	0.177066
27	oar3_OAR}	99536741	0.06542
27	oar3_OAR}	99541383	-0.00276
27	oar3_OAR}	99544823	-0.00276
27	oar3_OAR}	99551058	0.114936
27	oar3_OAR}	99560790	-0.00276
27	oar3_OAR}	99563359	0.002424
27	oar3_OAR}	99567941	0.054029
27	oar3_OAR}	99574678	0.0156
27	oar3_OAR}	99576317	-0.03185
27	oar3_OAR}	99577832	-0.01418
27	oar3_OAR}	99598706	0.00037
27	oar3_OAR}	99598733	0.00037
27	oar3_OAR}	99599195	0.00037
27	oar3_OAR}	99610974	0.027501
27	oar3_OAR}	99611626	0.059918
27	oar3_OAR}	99623100	0.07719
27	oar3_OAR}	99623485	-0.02202
27	oar3_OAR}	99624018	0.023872
27	oar3_OAR}	99631963	-0.02659
27	oar3_OAR}	99634174	0.035848

27	oar3_OAR>	99642235	0.008378
27	oar3_OAR>	99647136	0.008378
27	oar3_OAR>	99649186	0.101709
27	oar3_OAR>	99650115	-0.00367
27	oar3_OAR>	99653200	0.075987
27	oar3_OAR>	99660724	0.025179
27	oar3_OAR>	99661005	0.032156
27	oar3_OAR>	99663058	-0.00859
27	oar3_OAR>	99663663	0.029766
27	oar3_OAR>	99690436	-0.03186
27	oar3_OAR>	99694675	0.043765
27	oar3_OAR>	99695832	-0.01593
27	oar3_OAR>	99701409	0.024204
27	oar3_OAR>	99711118	0.153971
27	oar3_OAR>	99714584	0.049469
27	oar3_OAR>	99722834	0.097431
27	oar3_OAR>	99723242	0.097431
27	oar3_OAR>	99724405	-0.02533
27	oar3_OAR>	99730739	-0.0206
27	oar3_OAR>	99730748	-0.0206
27	oar3_OAR>	99738568	-0.00606
27	oar3_OAR>	99738729	-0.00606
27	oar3_OAR>	99740172	-0.0206
27	oar3_OAR>	99744238	-0.0206
27	oar3_OAR>	99764837	0.118917
27	oar3_OAR>	99765053	-0.0433
27	oar3_OAR>	99773494	-0.01928
27	oar3_OAR>	99778294	-0.02888
27	oar3_OAR>	99797566	-0.00606
27	oar3_OAR>	99799448	0.128869
27	oar3_OAR>	99800273	-0.01849
27	oar3_OAR>	99808904	0.005952
27	oar3_OAR>	99811728	0.042554
27	oar3_OAR>	99822408	0.026955
27	oar3_OAR>	99823592	-0.00524
27	oar3_OAR>	99832807	-0.02151
27	oar3_OAR>	99844235	0.011374
27	oar3_OAR>	99845857	0.006377
27	oar3_OAR>	99849202	-0.00998
27	oar3_OAR>	99858190	-0.02415
27	oar3_OAR>	99860221	0.058332
27	oar3_OAR>	99861062	-0.00924
27	oar3_OAR>	99866219	0.024815
27	oar3_OAR>	99866794	-0.00568
27	oar3_OAR>	99882332	-0.03171
27	oar3_OAR>	99888548	-0.02051
27	oar3_OAR>	99888763	0.008988
27	oar3_OAR>	99891409	-0.03171
27	oar3_OAR>	99893232	0.095866
27	oar3_OAR>	99899778	-0.00771
27	oar3_OAR>	99901220	-0.00868
27	oar3_OAR>	99908313	-0.00771
27	oar3_OAR>	99910027	-0.00868
27	oar3_OAR>	99910876	-0.00868

27	oar3_OAR>	99912079	-0.00868
27	oar3_OAR>	99917189	0.008988
27	oar3_OAR>	99921035	-0.00568
27	oar3_OAR>	99921152	-0.02383
27	oar3_OAR>	99923555	0.023138
27	oar3_OAR>	99923898	-0.03298
27	oar3_OAR>	99930421	0.001969
27	oar3_OAR>	99930661	-0.02892
27	oar3_OAR>	99931897	-0.02892
27	oar3_OAR>	99940350	0.006769
27	oar3_OAR>	99941121	-0.03275
27	oar3_OAR>	99941597	-0.0366
27	oar3_OAR>	99950681	-0.04013
27	oar3_OAR>	99951126	0.030952
27	oar3_OAR>	99952772	-0.03135
27	oar3_OAR>	99955800	-0.00561
27	oar3_OAR>	99958684	0.005261
27	oar3_OAR>	99961572	-0.02304
27	oar3_OAR>	99964108	-0.02095
27	oar3_OAR>	99969187	-0.00561
27	oar3_OAR>	99970717	0.110642
27	oar3_OAR>	99972255	0.033935
27	oar3_OAR>	99979276	-0.00508
27	oar3_OAR>	99980043	0.002108
27	oar3_OAR>	99980087	-0.02351
27	oar3_OAR>	99983920	-0.03582
27	oar3_OAR>	99988072	-0.03114
27	oar3_OAR>	99995139	0.003859
27	oar3_OAR>	99997471	-0.00082
27	oar3_OAR>	1E+08	-0.02179
27	oar3_OAR>	1E+08	0.049914
27	oar3_OAR>	1E+08	-0.00082
27	oar3_OAR>	1E+08	-0.02804
27	oar3_OAR>	1E+08	-0.02181
27	oar3_OAR>	1E+08	0.049914
27	oar3_OAR>	1E+08	0.015047
27	oar3_OAR>	1E+08	0.047157
27	oar3_OAR>	1E+08	0.047157
27	oar3_OAR>	1E+08	-0.02209
27	oar3_OAR>	1E+08	0.014046
27	oar3_OAR>	1E+08	-0.03698
27	oar3_OAR>	1E+08	0.014046
27	oar3_OAR>	1E+08	-0.01809
27	oar3_OAR>	1E+08	0.026578
27	oar3_OAR>	1E+08	0.026578
27	oar3_OAR>	1E+08	NA
27	oar3_OAR>	1E+08	-0.01809
27	oar3_OAR>	1E+08	0.014046
27	oar3_OAR>	1E+08	NA
27	oar3_OAR>	1E+08	0.018326
27	oar3_OAR>	1E+08	-0.02209
27	oar3_OAR>	1E+08	-0.01295
27	oar3_OAR>	1E+08	-0.01809
27	oar3_OAR>	1E+08	-0.00524

27	oar3_OAR〉	1E+08	-0.02209
27	oar3_OAR〉	1E+08	-0.02377
27	oar3_OAR〉	1E+08	-0.02209
27	oar3_OAR〉	1E+08	-0.02377
27	oar3_OAR〉	1E+08	-0.02377
27	oar3_OAR〉	1E+08	-0.01445
27	oar3_OAR〉	1E+08	0.032125
27	oar3_OAR〉	1E+08	0.094749
27	oar3_OAR〉	1E+08	0.007207
27	oar3_OAR〉	1E+08	0.069173
27	oar3_OAR〉	1E+08	-0.02137
27	oar3_OAR〉	1E+08	-0.02137
27	oar3_OAR〉	1E+08	-0.02137
27	oar3_OAR〉	1E+08	0.113754
27	oar3_OAR〉	1E+08	-0.02032
27	oar3_OAR〉	1E+08	-0.01745
27	oar3_OAR〉	1E+08	-0.00464
27	oar3_OAR〉	1E+08	-0.03168
27	oar3_OAR〉	1E+08	0.042244
27	oar3_OAR〉	1E+08	0.117575
27	oar3_OAR〉	1E+08	-0.00195
27	oar3_OAR〉	1E+08	-0.008
27	oar3_OAR〉	1E+08	0.031119
27	oar3_OAR〉	1E+08	-0.008
27	oar3_OAR〉	1E+08	-0.00995
27	oar3_OAR〉	1E+08	0.031805
27	oar3_OAR〉	1E+08	-0.02565
27	oar3_OAR〉	1E+08	0.018438
27	oar3_OAR〉	1E+08	0.039371
27	oar3_OAR〉	1E+08	0.039371
27	oar3_OAR〉	1E+08	0.116426
27	oar3_OAR〉	1E+08	-0.00602
27	oar3_OAR〉	1E+08	0.039371
27	oar3_OAR〉	1E+08	0.003004
27	oar3_OAR〉	1E+08	0.095871
27	oar3_OAR〉	1E+08	0.005932
27	oar3_OAR〉	1E+08	-0.007
27	oar3_OAR〉	1E+08	-0.01644
27	oar3_OAR〉	1E+08	-0.00697
27	oar3_OAR〉	1E+08	0.01079
27	oar3_OAR〉	1E+08	-0.01657
27	oar3_OAR〉	1E+08	0.027454
27	oar3_OAR〉	1E+08	-0.02593
27	oar3_OAR〉	1E+08	0.052515
27	oar3_OAR〉	1E+08	-0.03298
27	oar3_OAR〉	1E+08	0.019394
27	oar3_OAR〉	1E+08	-0.01423
27	oar3_OAR〉	1E+08	0.009277
27	oar3_OAR〉	1E+08	-0.01007
27	oar3_OAR〉	1E+08	-0.02382
27	oar3_OAR〉	1E+08	-0.00508
27	oar3_OAR〉	1E+08	-0.03147
27	oar3_OAR〉	1E+08	0.073464
27	oar3_OAR〉	1E+08	-0.01939

27	oar3_OAR›	1E+08	0.003055
27	oar3_OAR›	1E+08	0.003055
27	oar3_OAR›	1E+08	-0.01639
27	oar3_OAR›	1E+08	-0.02856
27	oar3_OAR›	1E+08	-0.00941
27	oar3_OAR›	1E+08	-0.03124
27	oar3_OAR›	1E+08	0.062987
27	oar3_OAR›	1E+08	0.062987
27	oar3_OAR›	1E+08	-0.00405
27	oar3_OAR›	1E+08	0.024575
27	oar3_OAR›	1E+08	-0.00405
27	oar3_OAR›	1E+08	0.003253
27	oar3_OAR›	1E+08	-0.00405
27	oar3_OAR›	1E+08	-0.00405
27	oar3_OAR›	1E+08	0.049131
27	oar3_OAR›	1E+08	-0.02895
27	oar3_OAR›	1.01E+08	0.064935
27	oar3_OAR›	1.01E+08	0.018141
27	oar3_OAR›	1.01E+08	0.145441
27	oar3_OAR›	1.01E+08	0.215511
27	oar3_OAR›	1.01E+08	0.046929
27	oar3_OAR›	1.01E+08	0.122589
27	oar3_OAR›	1.01E+08	0.098478
27	oar3_OAR›	1.01E+08	-0.00028
27	oar3_OAR›	1.01E+08	0.045066
27	oar3_OAR›	1.01E+08	0.015769
27	oar3_OAR›	1.01E+08	0.073511
27	oar3_OAR›	1.01E+08	0.005381
27	oar3_OAR›	1.01E+08	-0.02642
27	oar3_OAR›	1.01E+08	-0.03029
27	oar3_OAR›	1.01E+08	-0.01643
27	oar3_OAR›	1.01E+08	-0.01816
27	oar3_OAR›	1.01E+08	-0.03029
27	oar3_OAR›	1.01E+08	-0.01643
27	oar3_OAR›	1.01E+08	-0.02077
27	oar3_OAR›	1.01E+08	-0.03488
27	oar3_OAR›	1.01E+08	NA
27	oar3_OAR›	1.01E+08	0.000889
27	oar3_OAR›	1.01E+08	0.015215
27	oar3_OAR›	1.01E+08	0.000889
27	oar3_OAR›	1.01E+08	0.019075
27	oar3_OAR›	1.01E+08	0.019075
27	oar3_OAR›	1.01E+08	-0.02476
27	oar3_OAR›	1.01E+08	0.000889
27	oar3_OAR›	1.01E+08	-0.02619
27	oar3_OAR›	1.01E+08	0.01026
27	oar3_OAR›	1.01E+08	0.019819
27	oar3_OAR›	1.01E+08	0.03438
27	oar3_OAR›	1.01E+08	0.03438
27	oar3_OAR›	1.01E+08	0.067307
27	oar3_OAR›	1.01E+08	0.000889
27	oar3_OAR›	1.01E+08	-0.0234
27	oar3_OAR›	1.01E+08	0.017008
27	oar3_OAR›	1.01E+08	0.010752

27	oar3_OAR›	1.01E+08	0.010752
27	oar3_OAR›	1.01E+08	-0.00048
27	oar3_OAR›	1.01E+08	0.097352
27	oar3_OAR›	1.01E+08	-0.00622
27	oar3_OAR›	1.01E+08	-0.02371
27	oar3_OAR›	1.01E+08	-0.02371
27	oar3_OAR›	1.01E+08	-0.0025
27	oar3_OAR›	1.01E+08	-0.02371
27	oar3_OAR›	1.01E+08	0.031105
27	oar3_OAR›	1.01E+08	-0.01367
27	oar3_OAR›	1.01E+08	0.064783
27	oar3_OAR›	1.01E+08	0.001778
27	oar3_OAR›	1.01E+08	-0.02921
27	oar3_OAR›	1.01E+08	0.084825
27	oar3_OAR›	1.01E+08	0.128465
27	oar3_OAR›	1.01E+08	0.073837
27	oar3_OAR›	1.01E+08	0.128465
27	oar3_OAR›	1.01E+08	0.128465
27	oar3_OAR›	1.01E+08	-0.00365
27	oar3_OAR›	1.01E+08	-0.00365
27	oar3_OAR›	1.01E+08	0.008256
27	oar3_OAR›	1.01E+08	-0.00408
27	oar3_OAR›	1.01E+08	-0.00327
27	oar3_OAR›	1.01E+08	0.030402
27	oar3_OAR›	1.01E+08	0.004596
27	oar3_OAR›	1.01E+08	0.004596
27	oar3_OAR›	1.01E+08	0.017513
27	oar3_OAR›	1.01E+08	-0.03941
27	oar3_OAR›	1.01E+08	-0.03181
27	oar3_OAR›	1.01E+08	-0.03351
27	oar3_OAR›	1.01E+08	-0.02269
27	oar3_OAR›	1.01E+08	-0.03758
27	oar3_OAR›	1.01E+08	0.004363
27	oar3_OAR›	1.01E+08	-0.00214
27	oar3_OAR›	1.01E+08	0.014275
27	oar3_OAR›	1.01E+08	0.004363
27	oar3_OAR›	1.01E+08	-0.04059
27	oar3_OAR›	1.01E+08	0.026516
27	oar3_OAR›	1.01E+08	0.022003
27	oar3_OAR›	1.01E+08	0.010722
27	oar3_OAR›	1.01E+08	-0.0384
27	oar3_OAR›	1.01E+08	0.010722
27	oar3_OAR›	1.01E+08	0.010722
27	oar3_OAR›	1.01E+08	-0.02127
27	oar3_OAR›	1.01E+08	0.007039
27	oar3_OAR›	1.01E+08	0.007039
27	oar3_OAR›	1.01E+08	0.024265
27	oar3_OAR›	1.01E+08	0.000214
27	oar3_OAR›	1.01E+08	0.050828
27	oar3_OAR›	1.01E+08	-0.03593
27	oar3_OAR›	1.01E+08	0.002157
27	oar3_OAR›	1.01E+08	0.036045
27	oar3_OAR›	1.01E+08	-0.0302
27	oar3_OAR›	1.01E+08	-0.0276

27	oar3_OAR›	1.01E+08	0.036045
27	oar3_OAR›	1.01E+08	0.036045
27	oar3_OAR›	1.01E+08	0.036045
27	oar3_OAR›	1.01E+08	-0.013
27	oar3_OAR›	1.01E+08	0.006964
27	oar3_OAR›	1.01E+08	0.018571
27	oar3_OAR›	1.01E+08	-0.03096
27	oar3_OAR›	1.01E+08	-0.03096
27	oar3_OAR›	1.01E+08	-0.03096
27	oar3_OAR›	1.01E+08	0.000101
27	oar3_OAR›	1.01E+08	-0.01704
27	oar3_OAR›	1.01E+08	-0.02082
27	oar3_OAR›	1.01E+08	-0.00403
27	oar3_OAR›	1.01E+08	-0.03914
27	oar3_OAR›	1.01E+08	-0.031
27	oar3_OAR›	1.01E+08	-0.03914
27	oar3_OAR›	1.01E+08	-0.03126
27	oar3_OAR›	1.01E+08	-0.01698
27	oar3_OAR›	1.01E+08	-0.02827
27	oar3_OAR›	1.01E+08	-0.03096
27	oar3_OAR›	1.01E+08	0.000877
27	oar3_OAR›	1.01E+08	-0.03389
27	oar3_OAR›	1.01E+08	-0.02989
27	oar3_OAR›	1.01E+08	0.080189
27	oar3_OAR›	1.01E+08	0.010327
27	oar3_OAR›	1.01E+08	0.038884
27	oar3_OAR›	1.01E+08	0.055059
27	oar3_OAR›	1.01E+08	0.075585
27	oar3_OAR›	1.01E+08	0.007193
27	oar3_OAR›	1.01E+08	0.021626
27	oar3_OAR›	1.01E+08	0.040893
27	oar3_OAR›	1.01E+08	0.070714
27	oar3_OAR›	1.01E+08	0.064516
27	oar3_OAR›	1.01E+08	-0.01639
27	oar3_OAR›	1.01E+08	-0.03209
27	oar3_OAR›	1.01E+08	-0.03079
27	oar3_OAR›	1.01E+08	-0.03209
27	oar3_OAR›	1.01E+08	-0.03209
27	oar3_OAR›	1.01E+08	-0.02903
27	oar3_OAR›	1.01E+08	-0.02903
27	oar3_OAR›	1.01E+08	-0.0297
27	oar3_OAR›	1.01E+08	-0.02951
27	oar3_OAR›	1.01E+08	-0.02903
27	oar3_OAR›	1.01E+08	-0.03471
27	oar3_OAR›	1.01E+08	-0.02714
27	oar3_OAR›	1.01E+08	0.021215
27	oar3_OAR›	1.01E+08	0.0126
27	oar3_OAR›	1.01E+08	-0.00991
27	oar3_OAR›	1.01E+08	0.02243
27	oar3_OAR›	1.01E+08	0.044724
27	oar3_OAR›	1.01E+08	0.02243
27	oar3_OAR›	1.01E+08	-0.0293
27	oar3_OAR›	1.01E+08	NA
27	oar3_OAR›	1.01E+08	NA

27	oar3_OAR〉	1.01E+08	NA
27	oar3_OAR〉	1.01E+08	-0.0243
27	oar3_OAR〉	1.01E+08	-0.03277
27	oar3_OAR〉	1.01E+08	-0.03308
27	oar3_OAR〉	1.01E+08	-0.03308
27	oar3_OAR〉	1.01E+08	0.02763
27	oar3_OAR〉	1.01E+08	-0.0057
27	oar3_OAR〉	1.01E+08	-0.00799
27	oar3_OAR〉	1.01E+08	0.028831
27	oar3_OAR〉	1.01E+08	0.007039
27	oar3_OAR〉	1.01E+08	0.040674
27	oar3_OAR〉	1.01E+08	-0.03341
27	oar3_OAR〉	1.01E+08	-0.03447
27	oar3_OAR〉	1.01E+08	0.007039
27	oar3_OAR〉	1.01E+08	-0.01272
27	oar3_OAR〉	1.01E+08	0.007039
27	oar3_OAR〉	1.01E+08	0.011854
27	oar3_OAR〉	1.01E+08	0.007039
27	oar3_OAR〉	1.01E+08	0.009507
27	oar3_OAR〉	1.01E+08	-0.01598
27	oar3_OAR〉	1.01E+08	-0.02158
27	oar3_OAR〉	1.01E+08	-0.02548
27	oar3_OAR〉	1.01E+08	0.002868
27	oar3_OAR〉	1.01E+08	0.002868
27	oar3_OAR〉	1.01E+08	-0.01687
27	oar3_OAR〉	1.01E+08	0.145567
27	oar3_OAR〉	1.01E+08	-0.01687
27	oar3_OAR〉	1.01E+08	-0.02676
27	oar3_OAR〉	1.01E+08	-0.01687
27	oar3_OAR〉	1.01E+08	-0.01378
27	oar3_OAR〉	1.01E+08	-0.01191
27	oar3_OAR〉	1.01E+08	-0.03591
27	oar3_OAR〉	1.01E+08	0.023064
27	oar3_OAR〉	1.01E+08	0.086617
27	oar3_OAR〉	1.01E+08	-0.01191
27	oar3_OAR〉	1.01E+08	-0.02566
27	oar3_OAR〉	1.01E+08	0.074711
27	oar3_OAR〉	1.01E+08	0.038594
27	oar3_OAR〉	1.01E+08	0.074711
27	oar3_OAR〉	1.01E+08	0.082222
27	oar3_OAR〉	1.02E+08	0.07468
27	oar3_OAR〉	1.02E+08	-0.0014
27	oar3_OAR〉	1.02E+08	0.087841
27	oar3_OAR〉	1.02E+08	-0.02083
27	oar3_OAR〉	1.02E+08	0.058574
27	oar3_OAR〉	1.02E+08	0.087494
27	oar3_OAR〉	1.02E+08	0.140902
27	oar3_OAR〉	1.02E+08	0.066389
27	oar3_OAR〉	1.02E+08	0.045963
27	oar3_OAR〉	1.02E+08	0.140902
27	oar3_OAR〉	1.02E+08	0.140902
27	oar3_OAR〉	1.02E+08	-0.00609
27	oar3_OAR〉	1.02E+08	0.059252
27	oar3_OAR〉	1.02E+08	0.082222

27	oar3_OAR〉	1.02E+08	-0.0281
27	oar3_OAR〉	1.02E+08	0.061868
27	oar3_OAR〉	1.02E+08	-0.0281
27	oar3_OAR〉	1.02E+08	0.049227
27	oar3_OAR〉	1.02E+08	0.154191
27	oar3_OAR〉	1.02E+08	0.140902
27	oar3_OAR〉	1.02E+08	0.081464
27	oar3_OAR〉	1.02E+08	0.140902
27	oar3_OAR〉	1.02E+08	0.140902
27	oar3_OAR〉	1.02E+08	0.207695
27	oar3_OAR〉	1.02E+08	0.012043
27	oar3_OAR〉	1.02E+08	0.039085
27	oar3_OAR〉	1.02E+08	0.081453
27	oar3_OAR〉	1.02E+08	-0.01423
27	oar3_OAR〉	1.02E+08	-0.0303
27	oar3_OAR〉	1.02E+08	-0.03437
27	oar3_OAR〉	1.02E+08	-0.02377
27	oar3_OAR〉	1.02E+08	-0.00357
27	oar3_OAR〉	1.02E+08	0.008723
27	oar3_OAR〉	1.02E+08	-0.01415
27	oar3_OAR〉	1.02E+08	0.04569
27	oar3_OAR〉	1.02E+08	0.182187
27	oar3_OAR〉	1.02E+08	0.110848
27	oar3_OAR〉	1.02E+08	0.044724
27	oar3_OAR〉	1.02E+08	0.203524
27	oar3_OAR〉	1.02E+08	0.248504
27	oar3_OAR〉	1.02E+08	0.248504
27	oar3_OAR〉	1.02E+08	0.248504
27	oar3_OAR〉	1.02E+08	-0.02721
27	oar3_OAR〉	1.02E+08	-0.02721
27	oar3_OAR〉	1.02E+08	0.077842
27	oar3_OAR〉	1.02E+08	0.01079
27	oar3_OAR〉	1.02E+08	-0.01157
27	oar3_OAR〉	1.02E+08	-0.02721
27	oar3_OAR〉	1.02E+08	-0.02458
27	oar3_OAR〉	1.02E+08	-0.03454
27	oar3_OAR〉	1.02E+08	-0.02721
27	oar3_OAR〉	1.02E+08	0.022148
27	oar3_OAR〉	1.02E+08	0.055998
27	oar3_OAR〉	1.02E+08	0.055998
27	oar3_OAR〉	1.02E+08	-0.02721
27	oar3_OAR〉	1.02E+08	-0.02721
27	oar3_OAR〉	1.02E+08	-0.00284
27	oar3_OAR〉	1.02E+08	0.107252
27	oar3_OAR〉	1.02E+08	0.233045
27	oar3_OAR〉	1.02E+08	0.059524
27	oar3_OAR〉	1.02E+08	0.05901
27	oar3_OAR〉	1.02E+08	0.232351
27	oar3_OAR〉	1.02E+08	0.233045
27	oar3_OAR〉	1.02E+08	-0.02721
27	oar3_OAR〉	1.02E+08	0.084783
27	oar3_OAR〉	1.02E+08	0.233045
27	oar3_OAR〉	1.02E+08	-0.015
27	oar3_OAR〉	1.02E+08	0.030434

27	oar3_OAR〉	1.02E+08	0.055774
27	oar3_OAR〉	1.02E+08	-0.00898
27	oar3_OAR〉	1.02E+08	-0.02474
27	oar3_OAR〉	1.02E+08	0.030434
27	oar3_OAR〉	1.02E+08	0.030434
27	oar3_OAR〉	1.02E+08	0.030434
27	oar3_OAR〉	1.02E+08	-0.0164
27	oar3_OAR〉	1.02E+08	0.06269
27	oar3_OAR〉	1.02E+08	0.06269
27	oar3_OAR〉	1.02E+08	-0.01692
27	oar3_OAR〉	1.02E+08	0.0841
27	oar3_OAR〉	1.02E+08	0.049504
27	oar3_OAR〉	1.02E+08	0.049504
27	oar3_OAR〉	1.02E+08	0.017015
27	oar3_OAR〉	1.02E+08	-0.02921
27	oar3_OAR〉	1.02E+08	-0.00207
27	oar3_OAR〉	1.02E+08	0.109539
27	oar3_OAR〉	1.02E+08	-0.02003
27	oar3_OAR〉	1.02E+08	-0.02003
27	oar3_OAR〉	1.02E+08	0.051623
27	oar3_OAR〉	1.02E+08	-0.02647
27	oar3_OAR〉	1.02E+08	-0.02471
27	oar3_OAR〉	1.02E+08	0.026679
27	oar3_OAR〉	1.02E+08	0.018106
27	oar3_OAR〉	1.02E+08	-0.02944
27	oar3_OAR〉	1.02E+08	-0.03414
27	oar3_OAR〉	1.02E+08	0.050888
27	oar3_OAR〉	1.02E+08	-0.0247
27	oar3_OAR〉	1.02E+08	0.10944
27	oar3_OAR〉	1.02E+08	-0.03302
27	oar3_OAR〉	1.02E+08	-0.00174
27	oar3_OAR〉	1.02E+08	0.085455
27	oar3_OAR〉	1.02E+08	0.017521
27	oar3_OAR〉	1.02E+08	0.00549
27	oar3_OAR〉	1.02E+08	-0.03024
27	oar3_OAR〉	1.02E+08	0.032459
27	oar3_OAR〉	1.02E+08	-0.00365
27	oar3_OAR〉	1.02E+08	0.037984
27	oar3_OAR〉	1.02E+08	0.021138
27	oar3_OAR〉	1.02E+08	0.021138
27	oar3_OAR〉	1.02E+08	0.021138
27	oar3_OAR〉	1.02E+08	0.014333
27	oar3_OAR〉	1.02E+08	0.034468
27	oar3_OAR〉	1.02E+08	0.021138
27	oar3_OAR〉	1.02E+08	0.021138
27	oar3_OAR〉	1.02E+08	0.02239
27	oar3_OAR〉	1.02E+08	0.013045
27	oar3_OAR〉	1.02E+08	0.013045
27	oar3_OAR〉	1.02E+08	0.034468
27	oar3_OAR〉	1.02E+08	0.098557
27	oar3_OAR〉	1.02E+08	-0.01727
27	oar3_OAR〉	1.02E+08	0.011067
27	oar3_OAR〉	1.02E+08	0.002819
27	oar3_OAR〉	1.02E+08	-0.00278

27	oar3_OAR⟩	1.02E+08	0.017203
27	oar3_OAR⟩	1.02E+08	0.068563
27	oar3_OAR⟩	1.02E+08	0.007973
27	oar3_OAR⟩	1.02E+08	0.003268
27	oar3_OAR⟩	1.02E+08	0.044724
27	oar3_OAR⟩	1.02E+08	0.037973
27	oar3_OAR⟩	1.02E+08	0.035885
27	oar3_OAR⟩	1.02E+08	0.003305
27	oar3_OAR⟩	1.02E+08	-0.03202
27	oar3_OAR⟩	1.02E+08	0.044724
27	oar3_OAR⟩	1.02E+08	0.028042
27	oar3_OAR⟩	1.02E+08	0.044724
27	oar3_OAR⟩	1.02E+08	-0.01295
27	oar3_OAR⟩	1.02E+08	-0.03405
27	oar3_OAR⟩	1.02E+08	-0.03032
27	oar3_OAR⟩	1.02E+08	-0.03405
27	oar3_OAR⟩	1.02E+08	-0.02325
27	oar3_OAR⟩	1.02E+08	-0.01214
27	oar3_OAR⟩	1.02E+08	-0.02961
27	oar3_OAR⟩	1.02E+08	-0.05164
27	oar3_OAR⟩	1.02E+08	-0.02171
27	oar3_OAR⟩	1.02E+08	-0.0156
27	oar3_OAR⟩	1.02E+08	-0.02564
27	oar3_OAR⟩	1.02E+08	-0.01142
27	oar3_OAR⟩	1.02E+08	-0.01366
27	oar3_OAR⟩	1.02E+08	-0.02897
27	oar3_OAR⟩	1.02E+08	0.000817
27	oar3_OAR⟩	1.02E+08	-0.009
27	oar3_OAR⟩	1.02E+08	-0.0385
27	oar3_OAR⟩	1.02E+08	-0.01173
27	oar3_OAR⟩	1.02E+08	-0.0156
27	oar3_OAR⟩	1.02E+08	-0.01164
27	oar3_OAR⟩	1.02E+08	-0.02897
27	oar3_OAR⟩	1.02E+08	-0.00563
27	oar3_OAR⟩	1.02E+08	-0.00563
27	oar3_OAR⟩	1.02E+08	-0.01423
27	oar3_OAR⟩	1.02E+08	-0.02003
27	oar3_OAR⟩	1.02E+08	-0.02003
27	oar3_OAR⟩	1.02E+08	########
27	oar3_OAR⟩	1.02E+08	########
27	oar3_OAR⟩	1.02E+08	-0.01423
27	oar3_OAR⟩	1.02E+08	-0.02429
27	oar3_OAR⟩	1.02E+08	0.002533
27	oar3_OAR⟩	1.02E+08	0.031796
27	oar3_OAR⟩	1.02E+08	-0.0334
27	oar3_OAR⟩	1.02E+08	-0.02151
27	oar3_OAR⟩	1.02E+08	-0.02766
27	oar3_OAR⟩	1.02E+08	0.004356
27	oar3_OAR⟩	1.02E+08	0.002635
27	oar3_OAR⟩	1.02E+08	-0.02913
27	oar3_OAR⟩	1.02E+08	0.065103
27	oar3_OAR⟩	1.02E+08	0.029507
27	oar3_OAR⟩	1.02E+08	-0.01255
27	oar3_OAR⟩	1.02E+08	0.04707

27	oar3_OAR〉	1.02E+08	-0.01121
27	oar3_OAR〉	1.02E+08	-0.01173
27	oar3_OAR〉	1.02E+08	0.049314
27	oar3_OAR〉	1.02E+08	0.031886
27	oar3_OAR〉	1.02E+08	-0.02216
27	oar3_OAR〉	1.02E+08	-0.02216
27	oar3_OAR〉	1.02E+08	-0.02793
27	oar3_OAR〉	1.02E+08	-0.00609
27	oar3_OAR〉	1.02E+08	0.103749
27	oar3_OAR〉	1.02E+08	-0.02502
27	oar3_OAR〉	1.02E+08	0.016885
27	oar3_OAR〉	1.02E+08	0.060136
27	oar3_OAR〉	1.02E+08	0.035346
27	oar3_OAR〉	1.02E+08	-0.00289
27	oar3_OAR〉	1.02E+08	0.122671
27	oar3_OAR〉	1.02E+08	0.052615
27	oar3_OAR〉	1.02E+08	0.018063
27	oar3_OAR〉	1.02E+08	-0.0199
27	oar3_OAR〉	1.02E+08	0.015641
27	oar3_OAR〉	1.02E+08	0.005255
27	oar3_OAR〉	1.02E+08	0.039481
27	oar3_OAR〉	1.02E+08	-0.0006
27	oar3_OAR〉	1.02E+08	0.004523
27	oar3_OAR〉	1.02E+08	0.006981
27	oar3_OAR〉	1.02E+08	0.03369
27	oar3_OAR〉	1.02E+08	0.03369
27	oar3_OAR〉	1.02E+08	0.015829
27	oar3_OAR〉	1.02E+08	0.047728
27	oar3_OAR〉	1.02E+08	-0.00428
27	oar3_OAR〉	1.02E+08	-0.01889
27	oar3_OAR〉	1.02E+08	-0.02255
27	oar3_OAR〉	1.02E+08	-0.02031
27	oar3_OAR〉	1.02E+08	-0.00428
27	oar3_OAR〉	1.02E+08	0.006752
27	oar3_OAR〉	1.02E+08	-0.03332
27	oar3_OAR〉	1.02E+08	0.090331
27	oar3_OAR〉	1.02E+08	0.028822
27	oar3_OAR〉	1.02E+08	0.001805
27	oar3_OAR〉	1.02E+08	0.029401
27	oar3_OAR〉	1.02E+08	-0.0136
27	oar3_OAR〉	1.02E+08	0.024495
27	oar3_OAR〉	1.02E+08	0.007039
27	oar3_OAR〉	1.02E+08	0.008954
27	oar3_OAR〉	1.02E+08	0.044471
27	oar3_OAR〉	1.03E+08	0.02864
27	oar3_OAR〉	1.03E+08	0.17405
27	oar3_OAR〉	1.03E+08	-0.032
27	oar3_OAR〉	1.03E+08	-0.02769
27	oar3_OAR〉	1.03E+08	-0.01753
27	oar3_OAR〉	1.03E+08	0.007039
27	oar3_OAR〉	1.03E+08	-0.0216
27	oar3_OAR〉	1.03E+08	-0.01558
27	oar3_OAR〉	1.03E+08	0.010036
27	oar3_OAR〉	1.03E+08	-0.03092

27	oar3_OAR〉	1.03E+08	-0.04114
27	oar3_OAR〉	1.03E+08	0.002825
27	oar3_OAR〉	1.03E+08	-0.00756
27	oar3_OAR〉	1.03E+08	-0.0292
27	oar3_OAR〉	1.03E+08	-0.0292
27	oar3_OAR〉	1.03E+08	-0.00354
27	oar3_OAR〉	1.03E+08	-0.01199
27	oar3_OAR〉	1.03E+08	0.069963
27	oar3_OAR〉	1.03E+08	-0.0107
27	oar3_OAR〉	1.03E+08	-0.0042
27	oar3_OAR〉	1.03E+08	0.022498
27	oar3_OAR〉	1.03E+08	0.017028
27	oar3_OAR〉	1.03E+08	-0.01847
27	oar3_OAR〉	1.03E+08	-0.01547
27	oar3_OAR〉	1.03E+08	-0.02216
27	oar3_OAR〉	1.03E+08	0.002715
27	oar3_OAR〉	1.03E+08	-0.03022
27	oar3_OAR〉	1.03E+08	-0.00765
27	oar3_OAR〉	1.03E+08	-0.03372
27	oar3_OAR〉	1.03E+08	0.016681
27	oar3_OAR〉	1.03E+08	-0.01766
27	oar3_OAR〉	1.03E+08	-0.01926
27	oar3_OAR〉	1.03E+08	0.006633
27	oar3_OAR〉	1.03E+08	-0.01208
27	oar3_OAR〉	1.03E+08	-0.01926
27	oar3_OAR〉	1.03E+08	0.001086
27	oar3_OAR〉	1.03E+08	0.000451
27	oar3_OAR〉	1.03E+08	-0.01308
27	oar3_OAR〉	1.03E+08	0.00215
27	oar3_OAR〉	1.03E+08	-0.02327
27	oar3_OAR〉	1.03E+08	-0.00174
27	oar3_OAR〉	1.03E+08	-0.00585
27	oar3_OAR〉	1.03E+08	0.053912
27	oar3_OAR〉	1.03E+08	0.010004
27	oar3_OAR〉	1.03E+08	-0.00776
27	oar3_OAR〉	1.03E+08	0.031688
27	oar3_OAR〉	1.03E+08	-0.00776
27	oar3_OAR〉	1.03E+08	0.065423
27	oar3_OAR〉	1.03E+08	0.018379
27	oar3_OAR〉	1.03E+08	0.052523
27	oar3_OAR〉	1.03E+08	-0.00654
27	oar3_OAR〉	1.03E+08	0.012921
27	oar3_OAR〉	1.03E+08	-0.00759
27	oar3_OAR〉	1.03E+08	-0.02292
27	oar3_OAR〉	1.03E+08	0.025205
27	oar3_OAR〉	1.03E+08	-0.0262
27	oar3_OAR〉	1.03E+08	-0.00788
27	oar3_OAR〉	1.03E+08	-0.00322
27	oar3_OAR〉	1.03E+08	-0.01248
27	oar3_OAR〉	1.03E+08	0.000889
27	oar3_OAR〉	1.03E+08	0.000889
27	oar3_OAR〉	1.03E+08	-0.02877
27	oar3_OAR〉	1.03E+08	-0.03183
27	oar3_OAR〉	1.03E+08	-0.02688

27	oar3_OAR〉	1.03E+08	-0.02716
27	oar3_OAR〉	1.03E+08	0.000889
27	oar3_OAR〉	1.03E+08	0.008997
27	oar3_OAR〉	1.03E+08	0.026977
27	oar3_OAR〉	1.03E+08	0.018678
27	oar3_OAR〉	1.03E+08	-0.02474
27	oar3_OAR〉	1.03E+08	0.10359
27	oar3_OAR〉	1.03E+08	0.10359
27	oar3_OAR〉	1.03E+08	0.004917
27	oar3_OAR〉	1.03E+08	-0.02964
27	oar3_OAR〉	1.03E+08	-0.02805
27	oar3_OAR〉	1.03E+08	0.020267
27	oar3_OAR〉	1.03E+08	0.026212
27	oar3_OAR〉	1.03E+08	-0.03024
27	oar3_OAR〉	1.03E+08	0.040355
27	oar3_OAR〉	1.03E+08	-0.02652
27	oar3_OAR〉	1.03E+08	-0.02645
27	oar3_OAR〉	1.03E+08	0.073704
27	oar3_OAR〉	1.03E+08	-0.01107
27	oar3_OAR〉	1.03E+08	0.008343
27	oar3_OAR〉	1.03E+08	-0.03157
27	oar3_OAR〉	1.03E+08	0.002702
27	oar3_OAR〉	1.03E+08	0.031222
27	oar3_OAR〉	1.03E+08	-0.02348
27	oar3_OAR〉	1.03E+08	0.027628
27	oar3_OAR〉	1.03E+08	-0.00539
27	oar3_OAR〉	1.03E+08	0.02269
27	oar3_OAR〉	1.03E+08	0.000135
27	oar3_OAR〉	1.03E+08	0.001207
27	oar3_OAR〉	1.03E+08	0.075352
27	oar3_OAR〉	1.03E+08	-0.02017
27	oar3_OAR〉	1.03E+08	0.035851
27	oar3_OAR〉	1.03E+08	0.026045
27	oar3_OAR〉	1.03E+08	0.153403
27	oar3_OAR〉	1.03E+08	0.195575
27	oar3_OAR〉	1.03E+08	-0.02974
27	oar3_OAR〉	1.03E+08	-0.02974
27	oar3_OAR〉	1.03E+08	0.141906
27	oar3_OAR〉	1.03E+08	-0.00209
27	oar3_OAR〉	1.03E+08	0.040756
27	oar3_OAR〉	1.03E+08	0.023703
27	oar3_OAR〉	1.03E+08	0.158426
27	oar3_OAR〉	1.03E+08	-0.02159
27	oar3_OAR〉	1.03E+08	0.043995
27	oar3_OAR〉	1.03E+08	0.037433
27	oar3_OAR〉	1.03E+08	0.102002
27	oar3_OAR〉	1.03E+08	0.102002
27	oar3_OAR〉	1.03E+08	0.05418
27	oar3_OAR〉	1.03E+08	-0.02719
27	oar3_OAR〉	1.03E+08	-0.02755
27	oar3_OAR〉	1.03E+08	0.102002
27	oar3_OAR〉	1.03E+08	0.074336
27	oar3_OAR〉	1.03E+08	0.062642
27	oar3_OAR〉	1.03E+08	0.012542

27	oar3_OAR›	1.03E+08	-0.02958
27	oar3_OAR›	1.03E+08	-0.03835
27	oar3_OAR›	1.03E+08	-0.03734
27	oar3_OAR›	1.03E+08	0.000363
27	oar3_OAR›	1.03E+08	-0.03171
27	oar3_OAR›	1.03E+08	0.032582
27	oar3_OAR›	1.03E+08	0.018804
27	oar3_OAR›	1.03E+08	0.011365
27	oar3_OAR›	1.03E+08	0.021197
27	oar3_OAR›	1.03E+08	0.000363
27	oar3_OAR›	1.03E+08	0.000363
27	oar3_OAR›	1.03E+08	-0.03389
27	oar3_OAR›	1.03E+08	0.018804
27	oar3_OAR›	1.03E+08	0.018804
27	oar3_OAR›	1.03E+08	-0.01588
27	oar3_OAR›	1.03E+08	-0.02891
27	oar3_OAR›	1.03E+08	-0.02648
27	oar3_OAR›	1.03E+08	-0.01158
27	oar3_OAR›	1.03E+08	-0.03417
27	oar3_OAR›	1.03E+08	-0.02831
27	oar3_OAR›	1.03E+08	-0.02737
27	oar3_OAR›	1.03E+08	-0.03359
27	oar3_OAR›	1.03E+08	-0.02926
27	oar3_OAR›	1.03E+08	-0.02279
27	oar3_OAR›	1.03E+08	0.013085
27	oar3_OAR›	1.03E+08	0.043186
27	oar3_OAR›	1.03E+08	-0.01126
27	oar3_OAR›	1.03E+08	0.01237
27	oar3_OAR›	1.03E+08	-0.00991
27	oar3_OAR›	1.03E+08	-0.00991
27	oar3_OAR›	1.03E+08	-0.01879
27	oar3_OAR›	1.03E+08	-0.02348
27	oar3_OAR›	1.03E+08	-0.02562
27	oar3_OAR›	1.03E+08	0.00661
27	oar3_OAR›	1.03E+08	0.010803
27	oar3_OAR›	1.03E+08	0.010803
27	oar3_OAR›	1.03E+08	0.00661
27	oar3_OAR›	1.03E+08	-0.02362
27	oar3_OAR›	1.03E+08	-0.01282
27	oar3_OAR›	1.03E+08	-0.02176
27	oar3_OAR›	1.03E+08	-0.00559
27	oar3_OAR›	1.03E+08	-0.02176
27	oar3_OAR›	1.03E+08	0.013573
27	oar3_OAR›	1.03E+08	0.013573
27	oar3_OAR›	1.03E+08	0.017138
27	oar3_OAR›	1.03E+08	0.064141
27	oar3_OAR›	1.03E+08	0.040859
27	oar3_OAR›	1.03E+08	0.01736
27	oar3_OAR›	1.03E+08	0.012045
27	oar3_OAR›	1.03E+08	-0.01547
27	oar3_OAR›	1.03E+08	-0.02564
27	oar3_OAR›	1.03E+08	-0.02167
27	oar3_OAR›	1.03E+08	-0.02769
27	oar3_OAR›	1.03E+08	-0.03337

27	oar3_OAR〉	1.03E+08	-0.03303
27	oar3_OAR〉	1.03E+08	-0.02847
27	oar3_OAR〉	1.03E+08	-0.01808
27	oar3_OAR〉	1.03E+08	-0.01585
27	oar3_OAR〉	1.03E+08	-0.02926
27	oar3_OAR〉	1.03E+08	0.042679
27	oar3_OAR〉	1.03E+08	0.022906
27	oar3_OAR〉	1.03E+08	0.003056
27	oar3_OAR〉	1.03E+08	-0.02677
27	oar3_OAR〉	1.03E+08	-0.02393
27	oar3_OAR〉	1.03E+08	-0.01069
27	oar3_OAR〉	1.03E+08	-0.03816
27	oar3_OAR〉	1.03E+08	-0.03488
27	oar3_OAR〉	1.03E+08	-0.00901
27	oar3_OAR〉	1.03E+08	-0.03543
27	oar3_OAR〉	1.03E+08	-0.03819
27	oar3_OAR〉	1.03E+08	-0.03688
27	oar3_OAR〉	1.03E+08	0.00172
27	oar3_OAR〉	1.03E+08	-0.03543
27	oar3_OAR〉	1.03E+08	0.004917
27	oar3_OAR〉	1.03E+08	-0.02533
27	oar3_OAR〉	1.03E+08	0.00172
27	oar3_OAR〉	1.03E+08	-0.03688
27	oar3_OAR〉	1.03E+08	-0.02533
27	oar3_OAR〉	1.03E+08	0.004917
27	oar3_OAR〉	1.03E+08	-0.02388
27	oar3_OAR〉	1.03E+08	-0.04057
27	oar3_OAR〉	1.03E+08	-0.03662
27	oar3_OAR〉	1.03E+08	-0.03662
27	oar3_OAR〉	1.03E+08	-0.02268
27	oar3_OAR〉	1.03E+08	-0.00322
27	oar3_OAR〉	1.03E+08	-0.01334
27	oar3_OAR〉	1.03E+08	-0.0036
27	oar3_OAR〉	1.03E+08	-0.01334
27	oar3_OAR〉	1.03E+08	0.018082
27	oar3_OAR〉	1.03E+08	0.016857
27	oar3_OAR〉	1.03E+08	-0.01163
27	oar3_OAR〉	1.03E+08	-0.00934
27	oar3_OAR〉	1.03E+08	0.051356
27	oar3_OAR〉	1.03E+08	0.001262
27	oar3_OAR〉	1.03E+08	0.099207
27	oar3_OAR〉	1.03E+08	0.03487
27	oar3_OAR〉	1.03E+08	0.04067
27	oar3_OAR〉	1.03E+08	0.038173
27	oar3_OAR〉	1.03E+08	0.038173
27	oar3_OAR〉	1.03E+08	0.038173
27	oar3_OAR〉	1.03E+08	-0.00382
27	oar3_OAR〉	1.03E+08	-0.0075
27	oar3_OAR〉	1.03E+08	-0.00809
27	oar3_OAR〉	1.03E+08	-0.01132
27	oar3_OAR〉	1.03E+08	0.000605
27	oar3_OAR〉	1.03E+08	-0.00356
27	oar3_OAR〉	1.03E+08	0.156659
27	oar3_OAR〉	1.03E+08	-0.0105

27	oar3_OAR〉	1.03E+08	-0.00465
27	oar3_OAR〉	1.03E+08	-0.0105
27	oar3_OAR〉	1.03E+08	0.062009
27	oar3_OAR〉	1.03E+08	-0.00348
27	oar3_OAR〉	1.03E+08	0.086912
27	oar3_OAR〉	1.03E+08	-0.01282
27	oar3_OAR〉	1.03E+08	-0.02374
27	oar3_OAR〉	1.03E+08	-0.02065
27	oar3_OAR〉	1.03E+08	-0.0315
27	oar3_OAR〉	1.03E+08	-0.0315
27	oar3_OAR〉	1.03E+08	-0.02327
27	oar3_OAR〉	1.03E+08	-0.00455
27	oar3_OAR〉	1.03E+08	-0.01416
27	oar3_OAR〉	1.03E+08	-0.02374
27	oar3_OAR〉	1.04E+08	0.069843
27	oar3_OAR〉	1.04E+08	-0.02562
27	oar3_OAR〉	1.04E+08	-0.02046
27	oar3_OAR〉	1.04E+08	0.012661
27	oar3_OAR〉	1.04E+08	0.003989
27	oar3_OAR〉	1.04E+08	-0.01569
27	oar3_OAR〉	1.04E+08	0.007893
27	oar3_OAR〉	1.04E+08	0.008007
27	oar3_OAR〉	1.04E+08	0.015808
27	oar3_OAR〉	1.04E+08	0.061606
27	oar3_OAR〉	1.04E+08	0.101047
27	oar3_OAR〉	1.04E+08	0.074254
27	oar3_OAR〉	1.04E+08	0.019938
27	oar3_OAR〉	1.04E+08	0.005926
27	oar3_OAR〉	1.04E+08	0.005926
27	oar3_OAR〉	1.04E+08	0.000605
27	oar3_OAR〉	1.04E+08	-0.02517
27	oar3_OAR〉	1.04E+08	0.028803
27	oar3_OAR〉	1.04E+08	0.002815
27	oar3_OAR〉	1.04E+08	0.064242
27	oar3_OAR〉	1.04E+08	-0.0185
27	oar3_OAR〉	1.04E+08	-0.01338
27	oar3_OAR〉	1.04E+08	0.031983
27	oar3_OAR〉	1.04E+08	0.086627
27	oar3_OAR〉	1.04E+08	-0.01338
27	oar3_OAR〉	1.04E+08	-0.00354
27	oar3_OAR〉	1.04E+08	-0.01856
27	oar3_OAR〉	1.04E+08	-0.02744
27	oar3_OAR〉	1.04E+08	0.016951
27	oar3_OAR〉	1.04E+08	-0.00875
27	oar3_OAR〉	1.04E+08	-0.01214
27	oar3_OAR〉	1.04E+08	0.000547
27	oar3_OAR〉	1.04E+08	0.003979
27	oar3_OAR〉	1.04E+08	0.040551
27	oar3_OAR〉	1.04E+08	0.003979
27	oar3_OAR〉	1.04E+08	-0.02873
27	oar3_OAR〉	1.04E+08	-0.013
27	oar3_OAR〉	1.04E+08	0.068428
27	oar3_OAR〉	1.04E+08	0.062127
27	oar3_OAR〉	1.04E+08	0.008196

27	oar3_OAR›	1.04E+08	0.075541
27	oar3_OAR›	1.04E+08	-0.01624
27	oar3_OAR›	1.04E+08	-0.01611
27	oar3_OAR›	1.04E+08	-0.01611
27	oar3_OAR›	1.04E+08	-0.00168
27	oar3_OAR›	1.04E+08	-0.00031
27	oar3_OAR›	1.04E+08	0.014309
27	oar3_OAR›	1.04E+08	-0.02133
27	oar3_OAR›	1.04E+08	0.051303
27	oar3_OAR›	1.04E+08	0.023064
27	oar3_OAR›	1.04E+08	0.023167
27	oar3_OAR›	1.04E+08	0.044724
27	oar3_OAR›	1.04E+08	0.007039
27	oar3_OAR›	1.04E+08	0.023167
27	oar3_OAR›	1.04E+08	0.007039
27	oar3_OAR›	1.04E+08	-0.00031
27	oar3_OAR›	1.04E+08	0.008894
27	oar3_OAR›	1.04E+08	-0.00502
27	oar3_OAR›	1.04E+08	0.026651
27	oar3_OAR›	1.04E+08	0.009322
27	oar3_OAR›	1.04E+08	-0.00299
27	oar3_OAR›	1.04E+08	0.022103
27	oar3_OAR›	1.04E+08	-0.02017
27	oar3_OAR›	1.04E+08	0.013973
27	oar3_OAR›	1.04E+08	-0.00199
27	oar3_OAR›	1.04E+08	-0.02782
27	oar3_OAR›	1.04E+08	-0.01655
27	oar3_OAR›	1.04E+08	0.087803
27	oar3_OAR›	1.04E+08	0.063319
27	oar3_OAR›	1.04E+08	-0.03062
27	oar3_OAR›	1.04E+08	0.011067
27	oar3_OAR›	1.04E+08	-0.02017
27	oar3_OAR›	1.04E+08	-0.00024
27	oar3_OAR›	1.04E+08	0.116655
27	oar3_OAR›	1.04E+08	0.185488
27	oar3_OAR›	1.04E+08	0.109786
27	oar3_OAR›	1.04E+08	-0.01412
27	oar3_OAR›	1.04E+08	-0.02787
27	oar3_OAR›	1.04E+08	0.043222
27	oar3_OAR›	1.04E+08	-0.01939
27	oar3_OAR›	1.04E+08	-0.02652
27	oar3_OAR›	1.04E+08	-0.0199
27	oar3_OAR›	1.04E+08	-0.02448
27	oar3_OAR›	1.04E+08	0.032582
27	oar3_OAR›	1.04E+08	-0.01644
27	oar3_OAR›	1.04E+08	-0.00243
27	oar3_OAR›	1.04E+08	0.026977
27	oar3_OAR›	1.04E+08	-0.03153
27	oar3_OAR›	1.04E+08	0.00481
27	oar3_OAR›	1.04E+08	-0.03153
27	oar3_OAR›	1.04E+08	0.026977
27	oar3_OAR›	1.04E+08	0.081248
27	oar3_OAR›	1.04E+08	0.00481
27	oar3_OAR›	1.04E+08	-0.02206

27	oar3_OAR>	1.04E+08	-0.01995
27	oar3_OAR>	1.04E+08	-0.02388
27	oar3_OAR>	1.04E+08	0.064345
27	oar3_OAR>	1.04E+08	-0.02697
27	oar3_OAR>	1.04E+08	-0.01538
27	oar3_OAR>	1.04E+08	-0.03705
27	oar3_OAR>	1.04E+08	-0.02017
27	oar3_OAR>	1.04E+08	-0.03064
27	oar3_OAR>	1.04E+08	-0.01673
27	oar3_OAR>	1.04E+08	-0.01673
27	oar3_OAR>	1.04E+08	-0.02063
27	oar3_OAR>	1.04E+08	-0.02943
27	oar3_OAR>	1.04E+08	-0.03809
27	oar3_OAR>	1.04E+08	-0.0334
27	oar3_OAR>	1.04E+08	-0.0343
27	oar3_OAR>	1.04E+08	-0.03761
27	oar3_OAR>	1.04E+08	-0.03761
27	oar3_OAR>	1.04E+08	-0.02593
27	oar3_OAR>	1.04E+08	-0.03543
27	oar3_OAR>	1.04E+08	0.026045
27	oar3_OAR>	1.04E+08	-0.02444
27	oar3_OAR>	1.04E+08	-0.02683
27	oar3_OAR>	1.04E+08	-0.0071
27	oar3_OAR>	1.04E+08	-0.00322
27	oar3_OAR>	1.04E+08	-0.02683
27	oar3_OAR>	1.04E+08	-0.00809
27	oar3_OAR>	1.04E+08	-0.00685
27	oar3_OAR>	1.04E+08	-0.03153
27	oar3_OAR>	1.04E+08	-0.00685
27	oar3_OAR>	1.04E+08	0.013793
27	oar3_OAR>	1.04E+08	-0.03135
27	oar3_OAR>	1.04E+08	-0.01789
27	oar3_OAR>	1.04E+08	-0.013
27	oar3_OAR>	1.04E+08	-0.02188
27	oar3_OAR>	1.04E+08	-0.00771
27	oar3_OAR>	1.04E+08	-0.03622
27	oar3_OAR>	1.04E+08	-0.04006
27	oar3_OAR>	1.04E+08	-0.03359
27	oar3_OAR>	1.04E+08	-0.01747
27	oar3_OAR>	1.04E+08	-0.03068
27	oar3_OAR>	1.04E+08	-0.02619
27	oar3_OAR>	1.04E+08	-0.0156
27	oar3_OAR>	1.04E+08	0.053987
27	oar3_OAR>	1.04E+08	-0.02188
27	oar3_OAR>	1.04E+08	-0.00411
27	oar3_OAR>	1.04E+08	-0.03239
27	oar3_OAR>	1.04E+08	0.022971
27	oar3_OAR>	1.04E+08	-0.00597
27	oar3_OAR>	1.04E+08	0.044724
27	oar3_OAR>	1.04E+08	0.010985
27	oar3_OAR>	1.04E+08	-0.0196
27	oar3_OAR>	1.04E+08	0.002327
27	oar3_OAR>	1.04E+08	0.000135
27	oar3_OAR>	1.04E+08	0.022281

27	oar3_OAR›	1.04E+08	0.01603
27	oar3_OAR›	1.04E+08	-0.0234
27	oar3_OAR›	1.04E+08	-0.01389
27	oar3_OAR›	1.04E+08	-0.03143
27	oar3_OAR›	1.04E+08	-0.01567
27	oar3_OAR›	1.04E+08	-0.00261
27	oar3_OAR›	1.04E+08	-0.01547
27	oar3_OAR›	1.05E+08	-0.02534
27	oar3_OAR›	1.05E+08	-0.03143
27	oar3_OAR›	1.05E+08	-0.03123
27	oar3_OAR›	1.05E+08	0.007039
27	oar3_OAR›	1.05E+08	-0.01908
27	oar3_OAR›	1.05E+08	0.080619
27	oar3_OAR›	1.05E+08	-0.00716
27	oar3_OAR›	1.05E+08	0.007039
27	oar3_OAR›	1.05E+08	0.009674
27	oar3_OAR›	1.05E+08	-0.01818
27	oar3_OAR›	1.05E+08	-0.02345
27	oar3_OAR›	1.05E+08	0.043995
27	oar3_OAR›	1.05E+08	0.050444
27	oar3_OAR›	1.05E+08	0.082222
27	oar3_OAR›	1.05E+08	0.080619
27	oar3_OAR›	1.05E+08	0.010341
27	oar3_OAR›	1.05E+08	0.036878
27	oar3_OAR›	1.05E+08	0.130345
27	oar3_OAR›	1.05E+08	0.011328
27	oar3_OAR›	1.05E+08	-0.01954
27	oar3_OAR›	1.05E+08	0.005608
27	oar3_OAR›	1.05E+08	0.023164
27	oar3_OAR›	1.05E+08	0.01578
27	oar3_OAR›	1.05E+08	-0.03232
27	oar3_OAR›	1.05E+08	0.071047
27	oar3_OAR›	1.05E+08	0.071047
27	oar3_OAR›	1.05E+08	0.057694
27	oar3_OAR›	1.05E+08	0.037217
27	oar3_OAR›	1.05E+08	0.036868
27	oar3_OAR›	1.05E+08	-0.02301
27	oar3_OAR›	1.05E+08	0.009767
27	oar3_OAR›	1.05E+08	0.081173
27	oar3_OAR›	1.05E+08	0.067058
27	oar3_OAR›	1.05E+08	0.074775
27	oar3_OAR›	1.05E+08	0.074775
27	oar3_OAR›	1.05E+08	-0.00309
27	oar3_OAR›	1.05E+08	0.011203
27	oar3_OAR›	1.05E+08	-0.02788
27	oar3_OAR›	1.05E+08	-0.0248
27	oar3_OAR›	1.05E+08	-0.00874
27	oar3_OAR›	1.05E+08	-0.00353
27	oar3_OAR›	1.05E+08	-0.03508
27	oar3_OAR›	1.05E+08	-0.01408
27	oar3_OAR›	1.05E+08	-0.02024
27	oar3_OAR›	1.05E+08	-0.01408
27	oar3_OAR›	1.05E+08	-0.02414
27	oar3_OAR›	1.05E+08	-0.01732

27	oar3_OAR›	1.05E+08	-0.00462
27	oar3_OAR›	1.05E+08	-0.01386
27	oar3_OAR›	1.05E+08	0.043603
27	oar3_OAR›	1.05E+08	-0.03493
27	oar3_OAR›	1.05E+08	0.017879
27	oar3_OAR›	1.05E+08	-0.02441
27	oar3_OAR›	1.05E+08	0.017106
27	oar3_OAR›	1.05E+08	-0.01673
27	oar3_OAR›	1.05E+08	-0.02996
27	oar3_OAR›	1.05E+08	0.017106
27	oar3_OAR›	1.05E+08	-0.02727
27	oar3_OAR›	1.05E+08	0.044623
27	oar3_OAR›	1.05E+08	-0.00789
27	oar3_OAR›	1.05E+08	-0.00513
27	oar3_OAR›	1.05E+08	-0.02217
27	oar3_OAR›	1.05E+08	-0.00513
27	oar3_OAR›	1.05E+08	0.000135
27	oar3_OAR›	1.05E+08	-0.04082
27	oar3_OAR›	1.05E+08	-0.03231
27	oar3_OAR›	1.05E+08	-0.02576
27	oar3_OAR›	1.05E+08	-0.03534
27	oar3_OAR›	1.05E+08	-0.04114
27	oar3_OAR›	1.05E+08	-0.02813
27	oar3_OAR›	1.05E+08	-0.0333
27	oar3_OAR›	1.05E+08	-0.02534
27	oar3_OAR›	1.05E+08	-0.02534
27	oar3_OAR›	1.05E+08	-0.00613
27	oar3_OAR›	1.05E+08	-0.03609
27	oar3_OAR›	1.05E+08	-0.02534
27	oar3_OAR›	1.05E+08	-0.02546
27	oar3_OAR›	1.05E+08	-0.0398
27	oar3_OAR›	1.05E+08	0.001128
27	oar3_OAR›	1.05E+08	-0.0152
27	oar3_OAR›	1.05E+08	0.001128
27	oar3_OAR›	1.05E+08	0.001128
27	oar3_OAR›	1.05E+08	0.001128
27	oar3_OAR›	1.05E+08	-0.03946
27	oar3_OAR›	1.05E+08	-0.0152
27	oar3_OAR›	1.05E+08	-0.02126
27	oar3_OAR›	1.05E+08	-0.0109
27	oar3_OAR›	1.05E+08	-0.0152
27	oar3_OAR›	1.05E+08	-0.01757
27	oar3_OAR›	1.05E+08	-0.02732
27	oar3_OAR›	1.05E+08	-0.0156
27	oar3_OAR›	1.05E+08	-0.00842
27	oar3_OAR›	1.05E+08	-0.00842
27	oar3_OAR›	1.05E+08	-0.03868
27	oar3_OAR›	1.05E+08	-0.02587
27	oar3_OAR›	1.05E+08	-0.02587
27	oar3_OAR›	1.05E+08	0.016285
27	oar3_OAR›	1.05E+08	-0.03209
27	oar3_OAR›	1.05E+08	-0.00844
27	oar3_OAR›	1.05E+08	-0.0163
27	oar3_OAR›	1.05E+08	0.003053

27	oar3_OAR〉	1.05E+08	-0.01384
27	oar3_OAR〉	1.05E+08	0.001737
27	oar3_OAR〉	1.05E+08	-0.01423
27	oar3_OAR〉	1.05E+08	0.031741
27	oar3_OAR〉	1.05E+08	0.004924
27	oar3_OAR〉	1.05E+08	0.004924
27	oar3_OAR〉	1.05E+08	-0.01069
27	oar3_OAR〉	1.05E+08	0.004924
27	oar3_OAR〉	1.05E+08	0.001737
27	oar3_OAR〉	1.05E+08	-0.01567
27	oar3_OAR〉	1.05E+08	-0.02926
27	oar3_OAR〉	1.05E+08	-0.02546
27	oar3_OAR〉	1.05E+08	-0.03531
27	oar3_OAR〉	1.05E+08	-0.01069
27	oar3_OAR〉	1.05E+08	-0.02269
27	oar3_OAR〉	1.05E+08	-0.02546
27	oar3_OAR〉	1.05E+08	-0.02824
27	oar3_OAR〉	1.05E+08	0.019057
27	oar3_OAR〉	1.05E+08	-0.00126
27	oar3_OAR〉	1.05E+08	-0.0467
27	oar3_OAR〉	1.05E+08	-0.03414
27	oar3_OAR〉	1.05E+08	-0.04134
27	oar3_OAR〉	1.05E+08	0.05549
27	oar3_OAR〉	1.05E+08	-0.00606
27	oar3_OAR〉	1.05E+08	0.015868
27	oar3_OAR〉	1.05E+08	-0.02627
27	oar3_OAR〉	1.05E+08	-0.02534
27	oar3_OAR〉	1.05E+08	0.052467
27	oar3_OAR〉	1.05E+08	0.011019
27	oar3_OAR〉	1.05E+08	-0.01767
27	oar3_OAR〉	1.05E+08	-0.01978
27	oar3_OAR〉	1.05E+08	0.052467
27	oar3_OAR〉	1.05E+08	0.056934
27	oar3_OAR〉	1.05E+08	-0.01625
27	oar3_OAR〉	1.05E+08	0.053574
27	oar3_OAR〉	1.05E+08	-0.01961
27	oar3_OAR〉	1.05E+08	0.030452
27	oar3_OAR〉	1.05E+08	0.030452
27	oar3_OAR〉	1.05E+08	0.012184
27	oar3_OAR〉	1.05E+08	0.044726
27	oar3_OAR〉	1.05E+08	0.053574
27	oar3_OAR〉	1.05E+08	0.008291
27	oar3_OAR〉	1.05E+08	0.012184
27	oar3_OAR〉	1.05E+08	0.028277
27	oar3_OAR〉	1.05E+08	-0.00534
27	oar3_OAR〉	1.05E+08	-0.00492
27	oar3_OAR〉	1.05E+08	0.028277
27	oar3_OAR〉	1.05E+08	0.042815
27	oar3_OAR〉	1.05E+08	0.028277
27	oar3_OAR〉	1.05E+08	0.042815
27	oar3_OAR〉	1.05E+08	-0.03796
27	oar3_OAR〉	1.05E+08	-0.03796
27	oar3_OAR〉	1.05E+08	0.028277
27	oar3_OAR〉	1.05E+08	0.00778

27	oar3_OAR〉	1.05E+08	0.052071
27	oar3_OAR〉	1.05E+08	-0.02565
27	oar3_OAR〉	1.05E+08	0.006164
27	oar3_OAR〉	1.05E+08	0.053574
27	oar3_OAR〉	1.05E+08	0.044003
27	oar3_OAR〉	1.05E+08	0.025222
27	oar3_OAR〉	1.05E+08	-0.02013
27	oar3_OAR〉	1.05E+08	-0.01367
27	oar3_OAR〉	1.05E+08	0.002868
27	oar3_OAR〉	1.05E+08	0.012472
27	oar3_OAR〉	1.05E+08	0.002868
27	oar3_OAR〉	1.05E+08	-0.01121
27	oar3_OAR〉	1.05E+08	0.002357
27	oar3_OAR〉	1.05E+08	0.075764
27	oar3_OAR〉	1.05E+08	-0.02616
27	oar3_OAR〉	1.05E+08	-0.00447
27	oar3_OAR〉	1.05E+08	-0.02616
27	oar3_OAR〉	1.05E+08	-0.02616
27	oar3_OAR〉	1.05E+08	0.028562
27	oar3_OAR〉	1.05E+08	-0.00776
27	oar3_OAR〉	1.05E+08	-0.02616
27	oar3_OAR〉	1.05E+08	-0.02616
27	oar3_OAR〉	1.05E+08	-0.02616
27	oar3_OAR〉	1.05E+08	0.002178
27	oar3_OAR〉	1.05E+08	0.002178
27	oar3_OAR〉	1.05E+08	0.002178
27	oar3_OAR〉	1.05E+08	0.002178
27	oar3_OAR〉	1.06E+08	0.002178
27	oar3_OAR〉	1.06E+08	0.002178
27	oar3_OAR〉	1.06E+08	-0.02709
27	oar3_OAR〉	1.06E+08	-0.00447
27	oar3_OAR〉	1.06E+08	-0.00447
27	oar3_OAR〉	1.06E+08	-0.00447
27	oar3_OAR〉	1.06E+08	-0.01643
27	oar3_OAR〉	1.06E+08	-0.03152
27	oar3_OAR〉	1.06E+08	-0.02703
27	oar3_OAR〉	1.06E+08	-0.026
27	oar3_OAR〉	1.06E+08	-0.02703
27	oar3_OAR〉	1.06E+08	-0.0236
27	oar3_OAR〉	1.06E+08	-0.02703
27	oar3_OAR〉	1.06E+08	-0.026
27	oar3_OAR〉	1.06E+08	-0.02703
27	oar3_OAR〉	1.06E+08	-0.00315
27	oar3_OAR〉	1.06E+08	-0.00061
27	oar3_OAR〉	1.06E+08	-0.02703
27	oar3_OAR〉	1.06E+08	-0.03248
27	oar3_OAR〉	1.06E+08	-0.03248
27	oar3_OAR〉	1.06E+08	-0.01286
27	oar3_OAR〉	1.06E+08	-0.01859
27	oar3_OAR〉	1.06E+08	-0.03248
27	oar3_OAR〉	1.06E+08	-0.01859
27	oar3_OAR〉	1.06E+08	-0.03248
27	oar3_OAR〉	1.06E+08	-0.02027
27	oar3_OAR〉	1.06E+08	-0.03248

27	oar3_OAR〉	1.06E+08	0.010869
27	oar3_OAR〉	1.06E+08	-0.02915
27	oar3_OAR〉	1.06E+08	-0.02915
27	oar3_OAR〉	1.06E+08	0.010869
27	oar3_OAR〉	1.06E+08	-0.034
27	oar3_OAR〉	1.06E+08	-0.03248
27	oar3_OAR〉	1.06E+08	-0.03298
27	oar3_OAR〉	1.06E+08	-0.01567
27	oar3_OAR〉	1.06E+08	-0.03101
27	oar3_OAR〉	1.06E+08	-0.00941
27	oar3_OAR〉	1.06E+08	-0.02057
27	oar3_OAR〉	1.06E+08	-0.01567
27	oar3_OAR〉	1.06E+08	-0.01079
27	oar3_OAR〉	1.06E+08	-0.02057
27	oar3_OAR〉	1.06E+08	0.016937
27	oar3_OAR〉	1.06E+08	0.016937
27	oar3_OAR〉	1.06E+08	-0.02744
27	oar3_OAR〉	1.06E+08	0.003949
27	oar3_OAR〉	1.06E+08	0.003949
27	oar3_OAR〉	1.06E+08	-0.01011
27	oar3_OAR〉	1.06E+08	-0.02184
27	oar3_OAR〉	1.06E+08	-0.0254
27	oar3_OAR〉	1.06E+08	-0.0254
27	oar3_OAR〉	1.06E+08	-0.0254
27	oar3_OAR〉	1.06E+08	-0.01588
27	oar3_OAR〉	1.06E+08	-0.01588
27	oar3_OAR〉	1.06E+08	-0.03126
27	oar3_OAR〉	1.06E+08	-0.01588
27	oar3_OAR〉	1.06E+08	0.009985
27	oar3_OAR〉	1.06E+08	-0.02759
27	oar3_OAR〉	1.06E+08	-0.03126
27	oar3_OAR〉	1.06E+08	-0.03126
27	oar3_OAR〉	1.06E+08	-0.03126
27	oar3_OAR〉	1.06E+08	-0.01897
27	oar3_OAR〉	1.06E+08	-0.03126
27	oar3_OAR〉	1.06E+08	-0.03126
27	oar3_OAR〉	1.06E+08	0.009985
27	oar3_OAR〉	1.06E+08	-0.03126
27	oar3_OAR〉	1.06E+08	-0.03126
27	oar3_OAR〉	1.06E+08	-0.03126
27	oar3_OAR〉	1.06E+08	-0.03126
27	oar3_OAR〉	1.06E+08	-0.03126
27	oar3_OAR〉	1.06E+08	-0.03126
27	oar3_OAR〉	1.06E+08	-0.00953
27	oar3_OAR〉	1.06E+08	-0.03126
27	oar3_OAR〉	1.06E+08	-0.03126
27	oar3_OAR〉	1.06E+08	-0.03126
27	oar3_OAR〉	1.06E+08	-0.03126
27	oar3_OAR〉	1.06E+08	-0.00788
27	oar3_OAR〉	1.06E+08	-0.03126
27	oar3_OAR〉	1.06E+08	-0.0248
27	oar3_OAR〉	1.06E+08	-0.0248
27	oar3_OAR〉	1.06E+08	-0.0248
27	oar3_OAR〉	1.06E+08	-0.02716

27	oar3_OAR〉	1.06E+08	-0.0248
27	oar3_OAR〉	1.06E+08	0.015138
27	oar3_OAR〉	1.06E+08	-0.01588
27	oar3_OAR〉	1.06E+08	-0.03152
27	oar3_OAR〉	1.06E+08	-0.01588
27	oar3_OAR〉	1.06E+08	-0.01588
27	oar3_OAR〉	1.06E+08	-0.01588
27	oar3_OAR〉	1.06E+08	0.018887
27	oar3_OAR〉	1.06E+08	-0.01588
27	oar3_OAR〉	1.06E+08	-0.02184
27	oar3_OAR〉	1.06E+08	-0.01588
27	oar3_OAR〉	1.06E+08	0.018887
27	oar3_OAR〉	1.06E+08	-0.0248
27	oar3_OAR〉	1.06E+08	-0.02413
27	oar3_OAR〉	1.06E+08	0.021371
27	oar3_OAR〉	1.06E+08	0.001737
27	oar3_OAR〉	1.06E+08	0.021371
27	oar3_OAR〉	1.06E+08	0.021371
27	oar3_OAR〉	1.06E+08	0.018887
27	oar3_OAR〉	1.06E+08	0.021371
27	oar3_OAR〉	1.06E+08	0.020069
27	oar3_OAR〉	1.06E+08	-0.02413
27	oar3_OAR〉	1.06E+08	0.022259
27	oar3_OAR〉	1.06E+08	-0.01503
27	oar3_OAR〉	1.06E+08	-0.00654
27	oar3_OAR〉	1.06E+08	0.012231
27	oar3_OAR〉	1.06E+08	-0.01503
27	oar3_OAR〉	1.06E+08	-0.00408
27	oar3_OAR〉	1.06E+08	-0.04065
27	oar3_OAR〉	1.06E+08	-0.01089
27	oar3_OAR〉	1.06E+08	0.010518
27	oar3_OAR〉	1.06E+08	-0.04065
27	oar3_OAR〉	1.06E+08	-0.01089
27	oar3_OAR〉	1.06E+08	-0.00654
27	oar3_OAR〉	1.06E+08	0.010518
27	oar3_OAR〉	1.06E+08	0.030065
27	oar3_OAR〉	1.06E+08	-0.01051
27	oar3_OAR〉	1.06E+08	-0.01051
27	oar3_OAR〉	1.06E+08	-0.01334
27	oar3_OAR〉	1.06E+08	-0.01086
27	oar3_OAR〉	1.06E+08	-0.04065
27	oar3_OAR〉	1.06E+08	-0.04065
27	oar3_OAR〉	1.06E+08	-0.02721
27	oar3_OAR〉	1.06E+08	-0.02721
27	oar3_OAR〉	1.06E+08	-0.00049
27	oar3_OAR〉	1.06E+08	-0.02114
27	oar3_OAR〉	1.06E+08	-0.02114
27	oar3_OAR〉	1.06E+08	-0.02721
27	oar3_OAR〉	1.06E+08	-0.03785
27	oar3_OAR〉	1.06E+08	-0.02721
27	oar3_OAR〉	1.06E+08	-0.02721
27	oar3_OAR〉	1.06E+08	-0.00049
27	oar3_OAR〉	1.06E+08	-0.02114
27	oar3_OAR〉	1.06E+08	0.003802

27	oar3_OAR>	1.06E+08	0.028768
27	oar3_OAR>	1.06E+08	-0.01439
27	oar3_OAR>	1.06E+08	-0.00677
27	oar3_OAR>	1.06E+08	-0.01629
27	oar3_OAR>	1.06E+08	-0.02645
27	oar3_OAR>	1.07E+08	-0.02645
27	oar3_OAR>	1.07E+08	-0.02716
27	oar3_OAR>	1.07E+08	-0.02197
27	oar3_OAR>	1.07E+08	-0.02716
27	oar3_OAR>	1.07E+08	-0.02645
27	oar3_OAR>	1.07E+08	-0.00677
27	oar3_OAR>	1.07E+08	-0.02716
27	oar3_OAR>	1.07E+08	-0.02716
27	oar3_OAR>	1.07E+08	-0.02197
27	oar3_OAR>	1.07E+08	-0.00677
27	oar3_OAR>	1.07E+08	-0.01779
27	oar3_OAR>	1.07E+08	0.01421
27	oar3_OAR>	1.07E+08	-0.02645
27	oar3_OAR>	1.07E+08	-0.03201
27	oar3_OAR>	1.07E+08	-0.03201
27	oar3_OAR>	1.07E+08	-0.02497
27	oar3_OAR>	1.07E+08	0.01421
27	oar3_OAR>	1.07E+08	-0.0031
27	oar3_OAR>	1.07E+08	-0.02497
27	oar3_OAR>	1.07E+08	-0.02497
27	oar3_OAR>	1.07E+08	-0.0031
27	oar3_OAR>	1.07E+08	-0.02497
27	oar3_OAR>	1.07E+08	0.028055
27	oar3_OAR>	1.07E+08	0.01421
27	oar3_OAR>	1.07E+08	0.005932
27	oar3_OAR>	1.07E+08	-0.02497
27	oar3_OAR>	1.07E+08	0.01421
27	oar3_OAR>	1.07E+08	-0.02497
27	oar3_OAR>	1.07E+08	-0.02529
27	oar3_OAR>	1.07E+08	0.041117
27	oar3_OAR>	1.07E+08	0.012289
27	oar3_OAR>	1.07E+08	0.041117
27	oar3_OAR>	1.07E+08	-0.01153
27	oar3_OAR>	1.07E+08	0.006417
27	oar3_OAR>	1.07E+08	0.022174
27	oar3_OAR>	1.07E+08	-0.01612
27	oar3_OAR>	1.07E+08	0.01421
27	oar3_OAR>	1.07E+08	-0.02197
27	oar3_OAR>	1.07E+08	-0.02197
27	oar3_OAR>	1.07E+08	-0.01612
27	oar3_OAR>	1.07E+08	0.051313
27	oar3_OAR>	1.07E+08	-0.02143
27	oar3_OAR>	1.07E+08	-0.01107
27	oar3_OAR>	1.07E+08	0.051313
27	oar3_OAR>	1.07E+08	0.031384
27	oar3_OAR>	1.07E+08	0.006417
27	oar3_OAR>	1.07E+08	-0.02946
27	oar3_OAR>	1.07E+08	0.006417
27	oar3_OAR>	1.07E+08	-0.02946

27	oar3_OAR›	1.07E+08	-0.02946
27	oar3_OAR›	1.07E+08	0.031384
27	oar3_OAR›	1.07E+08	-0.02946
27	oar3_OAR›	1.07E+08	-0.03273
27	oar3_OAR›	1.07E+08	-0.03463
27	oar3_OAR›	1.07E+08	0.006417
27	oar3_OAR›	1.07E+08	-0.03273
27	oar3_OAR›	1.07E+08	-0.03273
27	oar3_OAR›	1.07E+08	-0.03273
27	oar3_OAR›	1.07E+08	0.031384
27	oar3_OAR›	1.07E+08	0.056884
27	oar3_OAR›	1.07E+08	0.003001
27	oar3_OAR›	1.07E+08	0.039481
27	oar3_OAR›	1.07E+08	0.097113
27	oar3_OAR›	1.07E+08	0.071907
27	oar3_OAR›	1.07E+08	0.022982
27	oar3_OAR›	1.07E+08	0.022982
27	oar3_OAR›	1.07E+08	0.04878
27	oar3_OAR›	1.07E+08	0.022982
27	oar3_OAR›	1.07E+08	-0.01386
27	oar3_OAR›	1.07E+08	0.1143
27	oar3_OAR›	1.07E+08	0.126239
27	oar3_OAR›	1.07E+08	0.059093
27	oar3_OAR›	1.07E+08	0.039481
27	oar3_OAR›	1.07E+08	0.039481
27	oar3_OAR›	1.07E+08	0.071907
27	oar3_OAR›	1.07E+08	0.07368
27	oar3_OAR›	1.07E+08	0.049648
27	oar3_OAR›	1.07E+08	0.005942
27	oar3_OAR›	1.07E+08	0.039481
27	oar3_OAR›	1.07E+08	-0.01428
27	oar3_OAR›	1.07E+08	-0.01428
27	oar3_OAR›	1.07E+08	-0.00298
27	oar3_OAR›	1.07E+08	0.039481
27	oar3_OAR›	1.07E+08	0.001228
27	oar3_OAR›	1.07E+08	0.036751
27	oar3_OAR›	1.07E+08	0.128944
27	oar3_OAR›	1.07E+08	0.016726
27	oar3_OAR›	1.07E+08	0.013381
27	oar3_OAR›	1.07E+08	-0.04332
27	oar3_OAR›	1.07E+08	0.150234
27	oar3_OAR›	1.07E+08	-0.00236
27	oar3_OAR›	1.07E+08	0.086036
27	oar3_OAR›	1.07E+08	-0.01709
27	oar3_OAR›	1.07E+08	0.110573
27	oar3_OAR›	1.07E+08	0.117825
27	oar3_OAR›	1.07E+08	-0.03075
27	oar3_OAR›	1.07E+08	0.110573
27	oar3_OAR›	1.07E+08	0.117825
27	oar3_OAR›	1.07E+08	0.117825
27	oar3_OAR›	1.07E+08	0.093671
27	oar3_OAR›	1.07E+08	0.006027
27	oar3_OAR›	1.07E+08	0.056642
27	oar3_OAR›	1.07E+08	-0.00942

27	oar3_OAR〉	1.07E+08	0.012081
27	oar3_OAR〉	1.07E+08	0.056642
27	oar3_OAR〉	1.07E+08	-0.00942
27	oar3_OAR〉	1.07E+08	0.117825
27	oar3_OAR〉	1.07E+08	0.056642
27	oar3_OAR〉	1.07E+08	-0.02051
27	oar3_OAR〉	1.07E+08	0.027998
27	oar3_OAR〉	1.07E+08	-0.01959
27	oar3_OAR〉	1.07E+08	0.005381
27	oar3_OAR〉	1.07E+08	0.005381
27	oar3_OAR〉	1.07E+08	0.005381
27	oar3_OAR〉	1.07E+08	0.005381
27	oar3_OAR〉	1.07E+08	-0.02114
27	oar3_OAR〉	1.07E+08	0.072044
27	oar3_OAR〉	1.07E+08	0.010207
27	oar3_OAR〉	1.07E+08	0.068934
27	oar3_OAR〉	1.07E+08	0.036036
27	oar3_OAR〉	1.07E+08	0.060712
27	oar3_OAR〉	1.07E+08	0.036036
27	oar3_OAR〉	1.07E+08	0.036036
27	oar3_OAR〉	1.07E+08	0.061695
27	oar3_OAR〉	1.07E+08	0.060712
27	oar3_OAR〉	1.07E+08	0.010439
27	oar3_OAR〉	1.07E+08	0.01788
27	oar3_OAR〉	1.07E+08	0.02918
27	oar3_OAR〉	1.07E+08	0.02918
27	oar3_OAR〉	1.07E+08	0.014522
27	oar3_OAR〉	1.07E+08	0.055663
27	oar3_OAR〉	1.07E+08	0.055663
27	oar3_OAR〉	1.07E+08	0.014522
27	oar3_OAR〉	1.07E+08	0.055663
27	oar3_OAR〉	1.07E+08	0.014522
27	oar3_OAR〉	1.07E+08	0.055663
27	oar3_OAR〉	1.07E+08	0.000893
27	oar3_OAR〉	1.07E+08	-0.01423
27	oar3_OAR〉	1.07E+08	-0.00792
27	oar3_OAR〉	1.07E+08	-0.02988
27	oar3_OAR〉	1.07E+08	-0.02734
27	oar3_OAR〉	1.07E+08	0.007039
27	oar3_OAR〉	1.07E+08	-0.01327
27	oar3_OAR〉	1.07E+08	0.007039
27	oar3_OAR〉	1.07E+08	-0.01151
27	oar3_OAR〉	1.07E+08	-0.0293
27	oar3_OAR〉	1.07E+08	-0.0293
27	oar3_OAR〉	1.07E+08	-0.01327
27	oar3_OAR〉	1.07E+08	-0.01327
27	oar3_OAR〉	1.07E+08	-0.03555
27	oar3_OAR〉	1.07E+08	-0.02348
27	oar3_OAR〉	1.07E+08	-0.0253
27	oar3_OAR〉	1.07E+08	-0.03899
27	oar3_OAR〉	1.07E+08	-0.02348
27	oar3_OAR〉	1.07E+08	0.087334
27	oar3_OAR〉	1.07E+08	-0.00946
27	oar3_OAR〉	1.07E+08	-0.02348

27	oar3_OAR>	1.07E+08	-0.04195
27	oar3_OAR>	1.07E+08	0.069904
27	oar3_OAR>	1.07E+08	-0.01778
27	oar3_OAR>	1.07E+08	-0.0006
27	oar3_OAR>	1.07E+08	-0.00791
27	oar3_OAR>	1.07E+08	-0.01368
27	oar3_OAR>	1.07E+08	-0.0006
27	oar3_OAR>	1.07E+08	-0.01368
27	oar3_OAR>	1.07E+08	-0.01368
27	oar3_OAR>	1.07E+08	-0.00597
27	oar3_OAR>	1.07E+08	-0.01368
27	oar3_OAR>	1.07E+08	0.006912
27	oar3_OAR>	1.07E+08	-0.00181
27	oar3_OAR>	1.07E+08	0.020654
27	oar3_OAR>	1.07E+08	-0.00928
27	oar3_OAR>	1.07E+08	-0.00209
27	oar3_OAR>	1.07E+08	3.85E-05
27	oar3_OAR>	1.07E+08	-0.00128
27	oar3_OAR>	1.07E+08	0.015186
27	oar3_OAR>	1.07E+08	-0.00345
27	oar3_OAR>	1.07E+08	-0.00209
27	oar3_OAR>	1.07E+08	0.020399
27	oar3_OAR>	1.07E+08	0.015186
27	oar3_OAR>	1.07E+08	0.015186
27	oar3_OAR>	1.07E+08	-0.01887
27	oar3_OAR>	1.07E+08	0.061695
27	oar3_OAR>	1.07E+08	0.028803
27	oar3_OAR>	1.07E+08	0.039481
27	oar3_OAR>	1.07E+08	-0.02576
27	oar3_OAR>	1.07E+08	-0.00384
27	oar3_OAR>	1.07E+08	-0.00817
27	oar3_OAR>	1.07E+08	-0.00546
27	oar3_OAR>	1.07E+08	0.010219
27	oar3_OAR>	1.07E+08	0.028803
27	oar3_OAR>	1.07E+08	0.039658
27	oar3_OAR>	1.07E+08	0.034627
27	oar3_OAR>	1.07E+08	0.034627
27	oar3_OAR>	1.07E+08	0.085513
27	oar3_OAR>	1.07E+08	-0.02127
27	oar3_OAR>	1.07E+08	-0.03606
27	oar3_OAR>	1.07E+08	-0.02715
27	oar3_OAR>	1.07E+08	0.011365
27	oar3_OAR>	1.07E+08	0.029554
27	oar3_OAR>	1.07E+08	0.011365
27	oar3_OAR>	1.07E+08	0.076445
27	oar3_OAR>	1.07E+08	0.016058
27	oar3_OAR>	1.07E+08	0.076445
27	oar3_OAR>	1.07E+08	0.011365
27	oar3_OAR>	1.07E+08	0.011365
27	oar3_OAR>	1.07E+08	0.020125
27	oar3_OAR>	1.07E+08	-0.01333
27	oar3_OAR>	1.07E+08	0.076445
27	oar3_OAR>	1.07E+08	0.061098
27	oar3_OAR>	1.07E+08	0.076445

27	oar3_OAR〉	1.07E+08	0.026811
27	oar3_OAR〉	1.07E+08	-0.00901
27	oar3_OAR〉	1.07E+08	0.030729
27	oar3_OAR〉	1.07E+08	0.14977
27	oar3_OAR〉	1.07E+08	-0.02575
27	oar3_OAR〉	1.07E+08	0.052664
27	oar3_OAR〉	1.07E+08	0.136344
27	oar3_OAR〉	1.07E+08	-0.02593
27	oar3_OAR〉	1.07E+08	0.044724
27	oar3_OAR〉	1.08E+08	0.010073
27	oar3_OAR〉	1.08E+08	0.038643
27	oar3_OAR〉	1.08E+08	0.054599
27	oar3_OAR〉	1.08E+08	-0.0147
27	oar3_OAR〉	1.08E+08	0.034835
27	oar3_OAR〉	1.08E+08	-0.01653
27	oar3_OAR〉	1.08E+08	-0.0073
27	oar3_OAR〉	1.08E+08	0.022216
27	oar3_OAR〉	1.08E+08	0.055754
27	oar3_OAR〉	1.08E+08	-0.00927
27	oar3_OAR〉	1.08E+08	-0.0225
27	oar3_OAR〉	1.08E+08	-0.0225
27	oar3_OAR〉	1.08E+08	0.037224
27	oar3_OAR〉	1.08E+08	0.055899
27	oar3_OAR〉	1.08E+08	0.098571
27	oar3_OAR〉	1.08E+08	0.098571
27	oar3_OAR〉	1.08E+08	0.027002
27	oar3_OAR〉	1.08E+08	0.098571
27	oar3_OAR〉	1.08E+08	0.098571
27	oar3_OAR〉	1.08E+08	-0.03752
27	oar3_OAR〉	1.08E+08	-0.02612
27	oar3_OAR〉	1.08E+08	-0.0065
27	oar3_OAR〉	1.08E+08	-0.04941
27	oar3_OAR〉	1.08E+08	-0.03435
27	oar3_OAR〉	1.08E+08	0.001205
27	oar3_OAR〉	1.08E+08	0.052351
27	oar3_OAR〉	1.08E+08	0.013414
27	oar3_OAR〉	1.08E+08	-0.03153
27	oar3_OAR〉	1.08E+08	-0.02822
27	oar3_OAR〉	1.08E+08	-0.03153
27	oar3_OAR〉	1.08E+08	-0.00998
27	oar3_OAR〉	1.08E+08	0.002787
27	oar3_OAR〉	1.08E+08	-0.03153
27	oar3_OAR〉	1.08E+08	-0.02351
27	oar3_OAR〉	1.08E+08	-0.00052
27	oar3_OAR〉	1.08E+08	-0.01905
27	oar3_OAR〉	1.08E+08	-0.02652
27	oar3_OAR〉	1.08E+08	-0.02372
27	oar3_OAR〉	1.08E+08	-0.01406
27	oar3_OAR〉	1.08E+08	-0.02128
27	oar3_OAR〉	1.08E+08	-0.02128
27	oar3_OAR〉	1.08E+08	-0.01905
27	oar3_OAR〉	1.08E+08	-0.01787
27	oar3_OAR〉	1.08E+08	0.007039
27	oar3_OAR〉	1.08E+08	-0.02128

27	oar3_OAR〉	1.08E+08	-0.00795
27	oar3_OAR〉	1.08E+08	-0.00865
27	oar3_OAR〉	1.08E+08	-0.01905
27	oar3_OAR〉	1.08E+08	-0.02065
27	oar3_OAR〉	1.08E+08	0.056002
27	oar3_OAR〉	1.08E+08	-0.02972
27	oar3_OAR〉	1.08E+08	-0.01476
27	oar3_OAR〉	1.08E+08	-0.00181
27	oar3_OAR〉	1.08E+08	0.011522
27	oar3_OAR〉	1.08E+08	-0.01465
27	oar3_OAR〉	1.08E+08	-0.01024
27	oar3_OAR〉	1.08E+08	-0.00421
27	oar3_OAR〉	1.08E+08	-0.01343
27	oar3_OAR〉	1.08E+08	-0.0059
27	oar3_OAR〉	1.08E+08	-0.02933
27	oar3_OAR〉	1.08E+08	-0.02933
27	oar3_OAR〉	1.08E+08	-0.02511
27	oar3_OAR〉	1.08E+08	-0.03117
27	oar3_OAR〉	1.08E+08	0.023932
27	oar3_OAR〉	1.08E+08	-0.00583
27	oar3_OAR〉	1.08E+08	-0.02099
27	oar3_OAR〉	1.08E+08	0.023929
27	oar3_OAR〉	1.08E+08	-0.0276
27	oar3_OAR〉	1.08E+08	-0.01324
27	oar3_OAR〉	1.08E+08	-0.00814
27	oar3_OAR〉	1.08E+08	0.023929
27	oar3_OAR〉	1.08E+08	-0.00712
27	oar3_OAR〉	1.08E+08	-0.02289
27	oar3_OAR〉	1.08E+08	0.023929
27	oar3_OAR〉	1.08E+08	-0.00712
27	oar3_OAR〉	1.08E+08	0.023929
27	oar3_OAR〉	1.08E+08	-0.02531
27	oar3_OAR〉	1.08E+08	-0.02774
27	oar3_OAR〉	1.08E+08	-0.00712
27	oar3_OAR〉	1.08E+08	0.055673
27	oar3_OAR〉	1.08E+08	-0.02194
27	oar3_OAR〉	1.08E+08	0.023793
27	oar3_OAR〉	1.08E+08	-0.02835
27	oar3_OAR〉	1.08E+08	-0.00548
27	oar3_OAR〉	1.08E+08	-0.02835
27	oar3_OAR〉	1.08E+08	-0.02896
27	oar3_OAR〉	1.08E+08	-0.02292
27	oar3_OAR〉	1.08E+08	-0.02039
27	oar3_OAR〉	1.08E+08	-0.00548
27	oar3_OAR〉	1.08E+08	-0.02039
27	oar3_OAR〉	1.08E+08	-0.00267
27	oar3_OAR〉	1.08E+08	-0.01375
27	oar3_OAR〉	1.08E+08	-0.00548
27	oar3_OAR〉	1.08E+08	-0.01375
27	oar3_OAR〉	1.08E+08	-0.00174
27	oar3_OAR〉	1.08E+08	-0.0094
27	oar3_OAR〉	1.08E+08	-0.02753
27	oar3_OAR〉	1.08E+08	-0.02753
27	oar3_OAR〉	1.08E+08	-0.02578

27	oar3_OAR〉	1.08E+08	-0.00664
27	oar3_OAR〉	1.08E+08	-0.01164
27	oar3_OAR〉	1.08E+08	-0.02423
27	oar3_OAR〉	1.08E+08	-0.02423
27	oar3_OAR〉	1.08E+08	-0.02423
27	oar3_OAR〉	1.08E+08	-0.02423
27	oar3_OAR〉	1.08E+08	-0.02423
27	oar3_OAR〉	1.08E+08	0.256208
27	oar3_OAR〉	1.08E+08	0.059942
27	oar3_OAR〉	1.08E+08	-0.03858
27	oar3_OAR〉	1.08E+08	0.059942
27	oar3_OAR〉	1.08E+08	-0.02502
27	oar3_OAR〉	1.08E+08	-0.01262
27	oar3_OAR〉	1.08E+08	-0.00777
27	oar3_OAR〉	1.08E+08	0.01928
27	oar3_OAR〉	1.08E+08	0.01928
27	oar3_OAR〉	1.08E+08	0.01928
27	oar3_OAR〉	1.08E+08	0.01928
27	oar3_OAR〉	1.08E+08	0.034159
27	oar3_OAR〉	1.08E+08	-0.00952
27	oar3_OAR〉	1.08E+08	0.059341
27	oar3_OAR〉	1.08E+08	0.028665
27	oar3_OAR〉	1.08E+08	0.028665
27	oar3_OAR〉	1.08E+08	0.059341
27	oar3_OAR〉	1.08E+08	0.05685
27	oar3_OAR〉	1.08E+08	-0.0079
27	oar3_OAR〉	1.08E+08	0.045453
27	oar3_OAR〉	1.08E+08	0.011943
27	oar3_OAR〉	1.08E+08	0.013861
27	oar3_OAR〉	1.08E+08	0.013861
27	oar3_OAR〉	1.08E+08	0.03057
27	oar3_OAR〉	1.08E+08	-0.00221
27	oar3_OAR〉	1.08E+08	-0.02981
27	oar3_OAR〉	1.08E+08	########
27	oar3_OAR〉	1.08E+08	0.064031
27	oar3_OAR〉	1.08E+08	0.00471
27	oar3_OAR〉	1.08E+08	0.064031
27	oar3_OAR〉	1.08E+08	0.003904
27	oar3_OAR〉	1.08E+08	0.000708
27	oar3_OAR〉	1.08E+08	0.041221
27	oar3_OAR〉	1.08E+08	0.043722
27	oar3_OAR〉	1.08E+08	0.044724
27	oar3_OAR〉	1.08E+08	0.315251
27	oar3_OAR〉	1.08E+08	0.146938
27	oar3_OAR〉	1.08E+08	0.108753
27	oar3_OAR〉	1.08E+08	0.041776
27	oar3_OAR〉	1.08E+08	0.041776
27	oar3_OAR〉	1.08E+08	0.038472
27	oar3_OAR〉	1.08E+08	0.028473
27	oar3_OAR〉	1.08E+08	0.007035
27	oar3_OAR〉	1.08E+08	0.007035
27	oar3_OAR〉	1.08E+08	-0.02067
27	oar3_OAR〉	1.08E+08	-0.02067
27	oar3_OAR〉	1.08E+08	0.061695

27	oar3_OAR〉	1.08E+08	4.32E-05
27	oar3_OAR〉	1.08E+08	-0.02604
27	oar3_OAR〉	1.08E+08	0.007039
27	oar3_OAR〉	1.08E+08	-0.0158
27	oar3_OAR〉	1.08E+08	4.32E-05
27	oar3_OAR〉	1.08E+08	0.025857
27	oar3_OAR〉	1.08E+08	0.019459
27	oar3_OAR〉	1.08E+08	-0.02604
27	oar3_OAR〉	1.08E+08	0.019459
27	oar3_OAR〉	1.08E+08	0.004724
27	oar3_OAR〉	1.08E+08	0.004724
27	oar3_OAR〉	1.08E+08	0.050365
27	oar3_OAR〉	1.08E+08	0.088444
27	oar3_OAR〉	1.08E+08	-0.0036
27	oar3_OAR〉	1.08E+08	0.004724
27	oar3_OAR〉	1.08E+08	-0.02604
27	oar3_OAR〉	1.08E+08	0.019124
27	oar3_OAR〉	1.08E+08	-0.02604
27	oar3_OAR〉	1.08E+08	-0.01286
27	oar3_OAR〉	1.08E+08	0.019124
27	oar3_OAR〉	1.08E+08	0.076972
27	oar3_OAR〉	1.08E+08	0.02221
27	oar3_OAR〉	1.08E+08	-0.02974
27	oar3_OAR〉	1.08E+08	0.02221
27	oar3_OAR〉	1.08E+08	0.010036
27	oar3_OAR〉	1.08E+08	-0.01352
27	oar3_OAR〉	1.08E+08	0.02221
27	oar3_OAR〉	1.08E+08	-0.02974
27	oar3_OAR〉	1.09E+08	-0.00813
27	oar3_OAR〉	1.09E+08	0.047145
27	oar3_OAR〉	1.09E+08	-0.00813
27	oar3_OAR〉	1.09E+08	0.002213
27	oar3_OAR〉	1.09E+08	-0.00468
27	oar3_OAR〉	1.09E+08	-0.00468
27	oar3_OAR〉	1.09E+08	0.002213
27	oar3_OAR〉	1.09E+08	0.002213
27	oar3_OAR〉	1.09E+08	0.002213
27	oar3_OAR〉	1.09E+08	-0.00468
27	oar3_OAR〉	1.09E+08	0.002213
27	oar3_OAR〉	1.09E+08	0.036157
27	oar3_OAR〉	1.09E+08	0.029946
27	oar3_OAR〉	1.09E+08	-0.01166
27	oar3_OAR〉	1.09E+08	0.137437
27	oar3_OAR〉	1.09E+08	0.05072
27	oar3_OAR〉	1.09E+08	0.016214
27	oar3_OAR〉	1.09E+08	-0.00717
27	oar3_OAR〉	1.09E+08	0.07283
27	oar3_OAR〉	1.09E+08	0.07283
27	oar3_OAR〉	1.09E+08	0.07283
27	oar3_OAR〉	1.09E+08	0.040033
27	oar3_OAR〉	1.09E+08	0.069089
27	oar3_OAR〉	1.09E+08	0.016214
27	oar3_OAR〉	1.09E+08	0.22436
27	oar3_OAR〉	1.09E+08	0.19916

27	oar3_OAR〉	1.09E+08	-0.01234
27	oar3_OAR〉	1.09E+08	-0.00099
27	oar3_OAR〉	1.09E+08	-0.00276
27	oar3_OAR〉	1.09E+08	-0.02393
27	oar3_OAR〉	1.09E+08	-0.00276
27	oar3_OAR〉	1.09E+08	-0.00276
27	oar3_OAR〉	1.09E+08	-0.00276
27	oar3_OAR〉	1.09E+08	0.139863
27	oar3_OAR〉	1.09E+08	-0.00659
27	oar3_OAR〉	1.09E+08	0.060517
27	oar3_OAR〉	1.09E+08	-0.02051
27	oar3_OAR〉	1.09E+08	0.056101
27	oar3_OAR〉	1.09E+08	0.056101
27	oar3_OAR〉	1.09E+08	-0.03502
27	oar3_OAR〉	1.09E+08	-0.03265
27	oar3_OAR〉	1.09E+08	0.009985
27	oar3_OAR〉	1.09E+08	0.032839
27	oar3_OAR〉	1.09E+08	-0.0008
27	oar3_OAR〉	1.09E+08	0.013189
27	oar3_OAR〉	1.09E+08	0.017453
27	oar3_OAR〉	1.09E+08	0.013189
27	oar3_OAR〉	1.09E+08	-0.02209
27	oar3_OAR〉	1.09E+08	-0.03414
27	oar3_OAR〉	1.09E+08	0.008997
27	oar3_OAR〉	1.09E+08	0.025414
27	oar3_OAR〉	1.09E+08	0.002723
27	oar3_OAR〉	1.09E+08	-0.0313
27	oar3_OAR〉	1.09E+08	-0.02421
27	oar3_OAR〉	1.09E+08	0.057975
27	oar3_OAR〉	1.09E+08	0.081032
27	oar3_OAR〉	1.09E+08	0.048495
27	oar3_OAR〉	1.09E+08	-0.02421
27	oar3_OAR〉	1.09E+08	0.024156
27	oar3_OAR〉	1.09E+08	0.039297
27	oar3_OAR〉	1.09E+08	0.034743
27	oar3_OAR〉	1.09E+08	0.056085
27	oar3_OAR〉	1.09E+08	-0.01165
27	oar3_OAR〉	1.09E+08	0.099651
27	oar3_OAR〉	1.09E+08	-0.01031
27	oar3_OAR〉	1.09E+08	0.023306
27	oar3_OAR〉	1.09E+08	0.073421
27	oar3_OAR〉	1.09E+08	0.041373
27	oar3_OAR〉	1.09E+08	0.073421
27	oar3_OAR〉	1.09E+08	0.011067
27	oar3_OAR〉	1.09E+08	-0.02732
27	oar3_OAR〉	1.09E+08	0.039783
27	oar3_OAR〉	1.09E+08	0.00481
27	oar3_OAR〉	1.09E+08	-0.03738
27	oar3_OAR〉	1.09E+08	-0.02529
27	oar3_OAR〉	1.09E+08	-0.01459
27	oar3_OAR〉	1.09E+08	-0.02529
27	oar3_OAR〉	1.09E+08	-0.02737
27	oar3_OAR〉	1.09E+08	-0.03385
27	oar3_OAR〉	1.09E+08	-0.01171

27	oar3_OAR〉	1.09E+08	-0.02529
27	oar3_OAR〉	1.09E+08	0.055851
27	oar3_OAR〉	1.09E+08	0.131045
27	oar3_OAR〉	1.09E+08	0.021134
27	oar3_OAR〉	1.09E+08	-0.00178
27	oar3_OAR〉	1.09E+08	0.007039
27	oar3_OAR〉	1.09E+08	0.098799
27	oar3_OAR〉	1.09E+08	-0.00989
27	oar3_OAR〉	1.09E+08	-0.03471
27	oar3_OAR〉	1.09E+08	0.04428
27	oar3_OAR〉	1.09E+08	0.003214
27	oar3_OAR〉	1.09E+08	NA
27	oar3_OAR〉	1.09E+08	-0.0313
27	oar3_OAR〉	1.09E+08	-0.03489
27	oar3_OAR〉	1.09E+08	-0.02671
27	oar3_OAR〉	1.09E+08	-0.00307
27	oar3_OAR〉	1.09E+08	0.036193
27	oar3_OAR〉	1.09E+08	-0.03164
27	oar3_OAR〉	1.09E+08	-0.03559
27	oar3_OAR〉	1.09E+08	-0.03559
27	oar3_OAR〉	1.09E+08	-0.0168
27	oar3_OAR〉	1.09E+08	-0.00606
27	oar3_OAR〉	1.09E+08	-0.03498
27	oar3_OAR〉	1.09E+08	-0.0168
27	oar3_OAR〉	1.09E+08	0.055003
27	oar3_OAR〉	1.09E+08	-0.02162
27	oar3_OAR〉	1.09E+08	0.067788
27	oar3_OAR〉	1.09E+08	-0.02162
27	oar3_OAR〉	1.09E+08	0.067788
27	oar3_OAR〉	1.09E+08	-0.0168
27	oar3_OAR〉	1.09E+08	0.016767
27	oar3_OAR〉	1.09E+08	-0.01309
27	oar3_OAR〉	1.09E+08	0.054954
27	oar3_OAR〉	1.09E+08	0.065575
27	oar3_OAR〉	1.09E+08	0.065575
27	oar3_OAR〉	1.09E+08	0.054392
27	oar3_OAR〉	1.09E+08	0.054392
27	oar3_OAR〉	1.09E+08	-0.01214
27	oar3_OAR〉	1.09E+08	0.023093
27	oar3_OAR〉	1.09E+08	0.023093
27	oar3_OAR〉	1.09E+08	NA
27	oar3_OAR〉	1.09E+08	-0.01078
27	oar3_OAR〉	1.09E+08	-0.01629
27	oar3_OAR〉	1.09E+08	0.038564
27	oar3_OAR〉	1.09E+08	-0.01718
27	oar3_OAR〉	1.09E+08	-0.02358
27	oar3_OAR〉	1.09E+08	0.015211
27	oar3_OAR〉	1.09E+08	-0.0006
27	oar3_OAR〉	1.09E+08	-0.02276
27	oar3_OAR〉	1.09E+08	0.003874
27	oar3_OAR〉	1.09E+08	-0.00181
27	oar3_OAR〉	1.09E+08	-0.0006
27	oar3_OAR〉	1.09E+08	-0.0006
27	oar3_OAR〉	1.09E+08	-0.0006

27	oar3_OAR〉	1.09E+08	-0.02492
27	oar3_OAR〉	1.09E+08	0.003874
27	oar3_OAR〉	1.09E+08	0.003874
27	oar3_OAR〉	1.09E+08	0.011067
27	oar3_OAR〉	1.09E+08	0.00834
27	oar3_OAR〉	1.09E+08	0.142465
27	oar3_OAR〉	1.09E+08	0.027454
27	oar3_OAR〉	1.09E+08	0.063497
27	oar3_OAR〉	1.09E+08	0.031643
27	oar3_OAR〉	1.09E+08	0.050192
27	oar3_OAR〉	1.09E+08	0.031643
27	oar3_OAR〉	1.09E+08	-0.02872
27	oar3_OAR〉	1.09E+08	0.002897
27	oar3_OAR〉	1.09E+08	0.014864
27	oar3_OAR〉	1.09E+08	0.08119
27	oar3_OAR〉	1.09E+08	0.093958
27	oar3_OAR〉	1.09E+08	0.061695
27	oar3_OAR〉	1.09E+08	0.01278
27	oar3_OAR〉	1.09E+08	0.061695
27	oar3_OAR〉	1.09E+08	-0.02958
27	oar3_OAR〉	1.09E+08	-0.02216
27	oar3_OAR〉	1.09E+08	-0.02216
27	oar3_OAR〉	1.09E+08	0.002754
27	oar3_OAR〉	1.09E+08	-0.01594
27	oar3_OAR〉	1.09E+08	-0.01594
27	oar3_OAR〉	1.09E+08	-0.01286
27	oar3_OAR〉	1.09E+08	-0.01286
27	oar3_OAR〉	1.09E+08	0.055085
27	oar3_OAR〉	1.09E+08	7.84E-05
27	oar3_OAR〉	1.09E+08	-0.03193
27	oar3_OAR〉	1.09E+08	-0.0108
27	oar3_OAR〉	1.09E+08	-0.02072
27	oar3_OAR〉	1.09E+08	-0.02072
27	oar3_OAR〉	1.09E+08	-0.03679
27	oar3_OAR〉	1.09E+08	-0.01625
27	oar3_OAR〉	1.09E+08	-0.01625
27	oar3_OAR〉	1.09E+08	-0.01394
27	oar3_OAR〉	1.09E+08	-0.01394
27	oar3_OAR〉	1.09E+08	0.003394
27	oar3_OAR〉	1.09E+08	-0.02236
27	oar3_OAR〉	1.09E+08	-0.02768
27	oar3_OAR〉	1.09E+08	-0.01588
27	oar3_OAR〉	1.09E+08	-0.03073
27	oar3_OAR〉	1.1E+08	-0.01757
27	oar3_OAR〉	1.1E+08	0.000101
27	oar3_OAR〉	1.1E+08	-0.01248
27	oar3_OAR〉	1.1E+08	0.045494
27	oar3_OAR〉	1.1E+08	0.043504
27	oar3_OAR〉	1.1E+08	0.043504
27	oar3_OAR〉	1.1E+08	0.057831
27	oar3_OAR〉	1.1E+08	0.078549
27	oar3_OAR〉	1.1E+08	-0.00188
27	oar3_OAR〉	1.1E+08	0.078549
27	oar3_OAR〉	1.1E+08	-0.00188

27	oar3_OAR〉	1.1E+08	0.097086
27	oar3_OAR〉	1.1E+08	0.097086
27	oar3_OAR〉	1.1E+08	0.140361
27	oar3_OAR〉	1.1E+08	0.111878
27	oar3_OAR〉	1.1E+08	0.124302
27	oar3_OAR〉	1.1E+08	0.032591
27	oar3_OAR〉	1.1E+08	0.091709
27	oar3_OAR〉	1.1E+08	0.120726
27	oar3_OAR〉	1.1E+08	0.036955
27	oar3_OAR〉	1.1E+08	-0.03073
27	oar3_OAR〉	1.1E+08	0.004473
27	oar3_OAR〉	1.1E+08	-0.02031
27	oar3_OAR〉	1.1E+08	0.003448
27	oar3_OAR〉	1.1E+08	-0.02895
27	oar3_OAR〉	1.1E+08	-0.02031
27	oar3_OAR〉	1.1E+08	-0.02847
27	oar3_OAR〉	1.1E+08	-0.01639
27	oar3_OAR〉	1.1E+08	-0.00665
27	oar3_OAR〉	1.1E+08	-0.01067
27	oar3_OAR〉	1.1E+08	-0.03275
27	oar3_OAR〉	1.1E+08	0.010117
27	oar3_OAR〉	1.1E+08	0.012006
27	oar3_OAR〉	1.1E+08	-0.01657
27	oar3_OAR〉	1.1E+08	0.03648
27	oar3_OAR〉	1.1E+08	0.023228
27	oar3_OAR〉	1.1E+08	0.023228
27	oar3_OAR〉	1.1E+08	0.023228
27	oar3_OAR〉	1.1E+08	0.023228
27	oar3_OAR〉	1.1E+08	0.006015
27	oar3_OAR〉	1.1E+08	0.023228
27	oar3_OAR〉	1.1E+08	0.113754
27	oar3_OAR〉	1.1E+08	0.049985
27	oar3_OAR〉	1.1E+08	0.017899
27	oar3_OAR〉	1.1E+08	0.044943
27	oar3_OAR〉	1.1E+08	0.079352
27	oar3_OAR〉	1.1E+08	0.006828
27	oar3_OAR〉	1.1E+08	0.069912
27	oar3_OAR〉	1.1E+08	0.168008
27	oar3_OAR〉	1.1E+08	0.018618
27	oar3_OAR〉	1.1E+08	-0.00626
27	oar3_OAR〉	1.1E+08	0.026503
27	oar3_OAR〉	1.1E+08	0.054458
27	oar3_OAR〉	1.1E+08	0.168008
27	oar3_OAR〉	1.1E+08	0.056578
27	oar3_OAR〉	1.1E+08	0.000147
27	oar3_OAR〉	1.1E+08	-0.01314
27	oar3_OAR〉	1.1E+08	-0.01314
27	oar3_OAR〉	1.1E+08	-0.01314
27	oar3_OAR〉	1.1E+08	-0.01314
27	oar3_OAR〉	1.1E+08	0.053463
27	oar3_OAR〉	1.1E+08	0.014616
27	oar3_OAR〉	1.1E+08	0.013849
27	oar3_OAR〉	1.1E+08	-0.01007
27	oar3_OAR〉	1.1E+08	-0.00527

27	oar3_OAR〉	1.1E+08	-0.00527
27	oar3_OAR〉	1.1E+08	-0.01182
27	oar3_OAR〉	1.1E+08	0.021901
27	oar3_OAR〉	1.1E+08	0.031551
27	oar3_OAR〉	1.1E+08	-0.03171
27	oar3_OAR〉	1.1E+08	-0.04067
27	oar3_OAR〉	1.1E+08	-0.01567
27	oar3_OAR〉	1.1E+08	-0.03731
27	oar3_OAR〉	1.1E+08	-0.01567
27	oar3_OAR〉	1.1E+08	-0.03731
27	oar3_OAR〉	1.1E+08	-0.01567
27	oar3_OAR〉	1.1E+08	-0.00287
27	oar3_OAR〉	1.1E+08	-0.01567
27	oar3_OAR〉	1.1E+08	-0.01567
27	oar3_OAR〉	1.1E+08	-0.01567
27	oar3_OAR〉	1.1E+08	-0.01567
27	oar3_OAR〉	1.1E+08	-0.01567
27	oar3_OAR〉	1.1E+08	-0.0185
27	oar3_OAR〉	1.1E+08	-0.01612
27	oar3_OAR〉	1.1E+08	0.082222
27	oar3_OAR〉	1.1E+08	0.152405
27	oar3_OAR〉	1.1E+08	0.034274
27	oar3_OAR〉	1.1E+08	0.00365
27	oar3_OAR〉	1.1E+08	0.00746
27	oar3_OAR〉	1.1E+08	-0.00901
27	oar3_OAR〉	1.1E+08	-0.01223
27	oar3_OAR〉	1.1E+08	-0.0206
27	oar3_OAR〉	1.1E+08	0.10636
27	oar3_OAR〉	1.1E+08	0.032582
27	oar3_OAR〉	1.1E+08	0.052302
27	oar3_OAR〉	1.1E+08	-0.01194
27	oar3_OAR〉	1.1E+08	0.085861
27	oar3_OAR〉	1.1E+08	-0.0182
27	oar3_OAR〉	1.1E+08	0.038185
27	oar3_OAR〉	1.1E+08	0.022258
27	oar3_OAR〉	1.1E+08	-0.03734
27	oar3_OAR〉	1.1E+08	0.081176
27	oar3_OAR〉	1.1E+08	-0.01605
27	oar3_OAR〉	1.1E+08	0.022731
27	oar3_OAR〉	1.1E+08	0.029433
27	oar3_OAR〉	1.1E+08	0.025713
27	oar3_OAR〉	1.1E+08	-0.00199
27	oar3_OAR〉	1.1E+08	-0.00199
27	oar3_OAR〉	1.1E+08	0.025713
27	oar3_OAR〉	1.1E+08	0.011653
27	oar3_OAR〉	1.1E+08	0.03452
27	oar3_OAR〉	1.1E+08	0.005104
27	oar3_OAR〉	1.1E+08	0.009252
27	oar3_OAR〉	1.1E+08	0.000728
27	oar3_OAR〉	1.1E+08	0.01226
27	oar3_OAR〉	1.1E+08	0.000728
27	oar3_OAR〉	1.1E+08	0.077259
27	oar3_OAR〉	1.1E+08	0.021766
27	oar3_OAR〉	1.1E+08	0.052065

27	oar3_OAR〉	1.1E+08	0.052065
27	oar3_OAR〉	1.1E+08	0.021766
27	oar3_OAR〉	1.1E+08	0.052065
27	oar3_OAR〉	1.1E+08	0.031078
27	oar3_OAR〉	1.1E+08	0.031078
27	oar3_OAR〉	1.1E+08	0.024603
27	oar3_OAR〉	1.1E+08	0.021766
27	oar3_OAR〉	1.1E+08	-0.02693
27	oar3_OAR〉	1.1E+08	0.021766
27	oar3_OAR〉	1.1E+08	0.031078
27	oar3_OAR〉	1.1E+08	0.021766
27	oar3_OAR〉	1.1E+08	0.021766
27	oar3_OAR〉	1.1E+08	0.018157
27	oar3_OAR〉	1.1E+08	-0.0085
27	oar3_OAR〉	1.11E+08	0.120336
27	oar3_OAR〉	1.11E+08	0.059093
27	oar3_OAR〉	1.11E+08	-0.01869
27	oar3_OAR〉	1.11E+08	-0.01869
27	oar3_OAR〉	1.11E+08	-0.03167
27	oar3_OAR〉	1.11E+08	0.055946
27	oar3_OAR〉	1.11E+08	0.01463
27	oar3_OAR〉	1.11E+08	0.111915
27	oar3_OAR〉	1.11E+08	0.032747
27	oar3_OAR〉	1.11E+08	0.072751
27	oar3_OAR〉	1.11E+08	0.072751
27	oar3_OAR〉	1.11E+08	0.082905
27	oar3_OAR〉	1.11E+08	0.027049
27	oar3_OAR〉	1.11E+08	0.136899
27	oar3_OAR〉	1.11E+08	-0.00148
27	oar3_OAR〉	1.11E+08	0.012316
27	oar3_OAR〉	1.11E+08	0.037529
27	oar3_OAR〉	1.11E+08	0.034973
27	oar3_OAR〉	1.11E+08	-0.01702
27	oar3_OAR〉	1.11E+08	-0.01702
27	oar3_OAR〉	1.11E+08	-0.01702
27	oar3_OAR〉	1.11E+08	-0.01702
27	oar3_OAR〉	1.11E+08	-0.01986
27	oar3_OAR〉	1.11E+08	-0.01605
27	oar3_OAR〉	1.11E+08	0.056888
27	oar3_OAR〉	1.11E+08	-0.01434
27	oar3_OAR〉	1.11E+08	0.031371
27	oar3_OAR〉	1.11E+08	0.041036
27	oar3_OAR〉	1.11E+08	-0.02083
27	oar3_OAR〉	1.11E+08	-0.03081
27	oar3_OAR〉	1.11E+08	-0.02786
27	oar3_OAR〉	1.11E+08	0.008511
27	oar3_OAR〉	1.11E+08	0.00481
27	oar3_OAR〉	1.11E+08	-0.0284
27	oar3_OAR〉	1.11E+08	-0.04116
27	oar3_OAR〉	1.11E+08	0.046572
27	oar3_OAR〉	1.11E+08	0.018027
27	oar3_OAR〉	1.11E+08	0.004523
27	oar3_OAR〉	1.11E+08	0.07748
27	oar3_OAR〉	1.11E+08	-0.01809

27	oar3_OAR>	1.11E+08	0.07748
27	oar3_OAR>	1.11E+08	0.029081
27	oar3_OAR>	1.11E+08	-0.01404
27	oar3_OAR>	1.11E+08	-0.00383
27	oar3_OAR>	1.11E+08	-0.03679
27	oar3_OAR>	1.11E+08	-0.01859
27	oar3_OAR>	1.11E+08	-0.03721
27	oar3_OAR>	1.11E+08	-0.03768
27	oar3_OAR>	1.11E+08	-0.03052
27	oar3_OAR>	1.11E+08	-0.03768
27	oar3_OAR>	1.11E+08	-0.03052
27	oar3_OAR>	1.11E+08	-0.001
27	oar3_OAR>	1.11E+08	0.032491
27	oar3_OAR>	1.11E+08	-0.03337
27	oar3_OAR>	1.11E+08	0.032491
27	oar3_OAR>	1.11E+08	0.032491
27	oar3_OAR>	1.11E+08	0.032491
27	oar3_OAR>	1.11E+08	0.032491
27	oar3_OAR>	1.11E+08	0.032491
27	oar3_OAR>	1.11E+08	-0.02693
27	oar3_OAR>	1.11E+08	0.012352
27	oar3_OAR>	1.11E+08	-0.02926
27	oar3_OAR>	1.11E+08	0.012352
27	oar3_OAR>	1.11E+08	-0.03088
27	oar3_OAR>	1.11E+08	-0.02734
27	oar3_OAR>	1.11E+08	-0.02677
27	oar3_OAR>	1.11E+08	-0.0049
27	oar3_OAR>	1.11E+08	-0.03375
27	oar3_OAR>	1.11E+08	-0.0049
27	oar3_OAR>	1.11E+08	-0.02262
27	oar3_OAR>	1.11E+08	-0.0049
27	oar3_OAR>	1.11E+08	-0.03637
27	oar3_OAR>	1.11E+08	-0.04067
27	oar3_OAR>	1.11E+08	0.028212
27	oar3_OAR>	1.11E+08	0.013849
27	oar3_OAR>	1.11E+08	0.049729
27	oar3_OAR>	1.11E+08	-0.03792
27	oar3_OAR>	1.11E+08	0.018399
27	oar3_OAR>	1.11E+08	0.049764
27	oar3_OAR>	1.11E+08	0.062765
27	oar3_OAR>	1.11E+08	0.062765
27	oar3_OAR>	1.11E+08	0.044724
27	oar3_OAR>	1.11E+08	-0.02321
27	oar3_OAR>	1.11E+08	0.076597
27	oar3_OAR>	1.11E+08	0.076597
27	oar3_OAR>	1.11E+08	-0.01344
27	oar3_OAR>	1.11E+08	0.030208
27	oar3_OAR>	1.11E+08	0.020735
27	oar3_OAR>	1.11E+08	0.086403
27	oar3_OAR>	1.11E+08	-0.02054
27	oar3_OAR>	1.11E+08	-0.00017
27	oar3_OAR>	1.11E+08	0.075958
27	oar3_OAR>	1.11E+08	-0.02054
27	oar3_OAR>	1.11E+08	-0.02054

27	oar3_OAR〉	1.11E+08	-0.02054
27	oar3_OAR〉	1.11E+08	-0.02054
27	oar3_OAR〉	1.11E+08	NA
27	oar3_OAR〉	1.11E+08	0.020466
27	oar3_OAR〉	1.11E+08	0.040205
27	oar3_OAR〉	1.11E+08	0.103893
27	oar3_OAR〉	1.11E+08	0.007982
27	oar3_OAR〉	1.11E+08	0.00073
27	oar3_OAR〉	1.11E+08	-0.0311
27	oar3_OAR〉	1.11E+08	0.000889
27	oar3_OAR〉	1.11E+08	-0.0187
27	oar3_OAR〉	1.11E+08	-0.01842
27	oar3_OAR〉	1.11E+08	-0.02822
27	oar3_OAR〉	1.11E+08	-0.0141
27	oar3_OAR〉	1.11E+08	-0.01776
27	oar3_OAR〉	1.11E+08	0.023751
27	oar3_OAR〉	1.11E+08	-0.01904
27	oar3_OAR〉	1.11E+08	0.02438
27	oar3_OAR〉	1.11E+08	0.063518
27	oar3_OAR〉	1.11E+08	0.02438
27	oar3_OAR〉	1.11E+08	0.102783
27	oar3_OAR〉	1.11E+08	0.048359
27	oar3_OAR〉	1.11E+08	0.022582
27	oar3_OAR〉	1.11E+08	0.022582
27	oar3_OAR〉	1.11E+08	-0.02425
27	oar3_OAR〉	1.11E+08	0.004406
27	oar3_OAR〉	1.11E+08	0.102723
27	oar3_OAR〉	1.11E+08	-0.00012
27	oar3_OAR〉	1.11E+08	-0.02419
27	oar3_OAR〉	1.11E+08	-0.00777
27	oar3_OAR〉	1.11E+08	0.015319
27	oar3_OAR〉	1.11E+08	-0.0049
27	oar3_OAR〉	1.11E+08	0.007039
27	oar3_OAR〉	1.11E+08	-0.01657
27	oar3_OAR〉	1.11E+08	0.031337
27	oar3_OAR〉	1.11E+08	0.075489
27	oar3_OAR〉	1.11E+08	0.044724
27	oar3_OAR〉	1.11E+08	0.165016
27	oar3_OAR〉	1.11E+08	-0.03752
27	oar3_OAR〉	1.11E+08	0.075175
27	oar3_OAR〉	1.11E+08	0.098171
27	oar3_OAR〉	1.11E+08	0.098171
27	oar3_OAR〉	1.11E+08	-0.02759
27	oar3_OAR〉	1.11E+08	0.06915
27	oar3_OAR〉	1.11E+08	0.075923
27	oar3_OAR〉	1.11E+08	0.012341
27	oar3_OAR〉	1.11E+08	0.000135
27	oar3_OAR〉	1.11E+08	-0.01675
27	oar3_OAR〉	1.11E+08	-0.02916
27	oar3_OAR〉	1.11E+08	0.095426
27	oar3_OAR〉	1.11E+08	0.014628
27	oar3_OAR〉	1.11E+08	0.08175
27	oar3_OAR〉	1.11E+08	0.125094
27	oar3_OAR〉	1.11E+08	0.08175

27	oar3_OAR〉	1.11E+08	0.010806
27	oar3_OAR〉	1.11E+08	-0.00141
27	oar3_OAR〉	1.11E+08	-0.02562
27	oar3_OAR〉	1.11E+08	-0.00685
27	oar3_OAR〉	1.11E+08	0.026177
27	oar3_OAR〉	1.11E+08	0.045484
27	oar3_OAR〉	1.11E+08	0.093817
27	oar3_OAR〉	1.11E+08	0.075649
27	oar3_OAR〉	1.11E+08	0.067589
27	oar3_OAR〉	1.11E+08	0.057808
27	oar3_OAR〉	1.11E+08	0.022184
27	oar3_OAR〉	1.11E+08	0.100078
27	oar3_OAR〉	1.11E+08	0.042251
27	oar3_OAR〉	1.11E+08	0.052764
27	oar3_OAR〉	1.11E+08	0.052764
27	oar3_OAR〉	1.11E+08	-0.01068
27	oar3_OAR〉	1.11E+08	0.032181
27	oar3_OAR〉	1.11E+08	0.01388
27	oar3_OAR〉	1.11E+08	0.016689
27	oar3_OAR〉	1.11E+08	-0.02926
27	oar3_OAR〉	1.11E+08	0.009918
27	oar3_OAR〉	1.11E+08	0.012749
27	oar3_OAR〉	1.11E+08	-0.00083
27	oar3_OAR〉	1.11E+08	-0.00735
27	oar3_OAR〉	1.11E+08	-0.00126
27	oar3_OAR〉	1.11E+08	-0.01819
27	oar3_OAR〉	1.11E+08	-0.02926
27	oar3_OAR〉	1.11E+08	0.009918
27	oar3_OAR〉	1.11E+08	0.022148
27	oar3_OAR〉	1.12E+08	0.011653
27	oar3_OAR〉	1.12E+08	-0.02926
27	oar3_OAR〉	1.12E+08	-0.01126
27	oar3_OAR〉	1.12E+08	-0.01414
27	oar3_OAR〉	1.12E+08	-0.02744
27	oar3_OAR〉	1.12E+08	0.002815
27	oar3_OAR〉	1.12E+08	-0.02414
27	oar3_OAR〉	1.12E+08	-0.02102
27	oar3_OAR〉	1.12E+08	0.064242
27	oar3_OAR〉	1.12E+08	0.020033
27	oar3_OAR〉	1.12E+08	-0.02897
27	oar3_OAR〉	1.12E+08	-0.02393
27	oar3_OAR〉	1.12E+08	-0.02447
27	oar3_OAR〉	1.12E+08	-0.0263
27	oar3_OAR〉	1.12E+08	0.016716
27	oar3_OAR〉	1.12E+08	0.016716
27	oar3_OAR〉	1.12E+08	-0.00063
27	oar3_OAR〉	1.12E+08	0.033898
27	oar3_OAR〉	1.12E+08	0.000889
27	oar3_OAR〉	1.12E+08	-0.03023
27	oar3_OAR〉	1.12E+08	-0.03023
27	oar3_OAR〉	1.12E+08	NA
27	oar3_OAR〉	1.12E+08	0.030434
27	oar3_OAR〉	1.12E+08	-0.00615
27	oar3_OAR〉	1.12E+08	-0.00615

27	oar3_OAR›	1.12E+08	0.030434
27	oar3_OAR›	1.12E+08	-0.01012
27	oar3_OAR›	1.12E+08	-0.01012
27	oar3_OAR›	1.12E+08	0.046686
27	oar3_OAR›	1.12E+08	0.02374
27	oar3_OAR›	1.12E+08	0.109594
27	oar3_OAR›	1.12E+08	0.079536
27	oar3_OAR›	1.12E+08	0.063705
27	oar3_OAR›	1.12E+08	0.017714
27	oar3_OAR›	1.12E+08	0.063705
27	oar3_OAR›	1.12E+08	0.043236
27	oar3_OAR›	1.12E+08	-0.01494
27	oar3_OAR›	1.12E+08	-0.00485
27	oar3_OAR›	1.12E+08	0.036403
27	oar3_OAR›	1.12E+08	0.048354
27	oar3_OAR›	1.12E+08	-0.02452
27	oar3_OAR›	1.12E+08	-0.02774
27	oar3_OAR›	1.12E+08	-0.02774
27	oar3_OAR›	1.12E+08	0.007477
27	oar3_OAR›	1.12E+08	0.012138
27	oar3_OAR›	1.12E+08	-0.03869
27	oar3_OAR›	1.12E+08	-0.01651
27	oar3_OAR›	1.12E+08	0.009812
27	oar3_OAR›	1.12E+08	0.004893
27	oar3_OAR›	1.12E+08	0.01645
27	oar3_OAR›	1.12E+08	0.043853
27	oar3_OAR›	1.12E+08	0.015597
27	oar3_OAR›	1.12E+08	0.015597
27	oar3_OAR›	1.12E+08	0.008408
27	oar3_OAR›	1.12E+08	0.125192
27	oar3_OAR›	1.12E+08	-0.003
27	oar3_OAR›	1.12E+08	0.086533
27	oar3_OAR›	1.12E+08	0.005147
27	oar3_OAR›	1.12E+08	-0.00882
27	oar3_OAR›	1.12E+08	-0.00841
27	oar3_OAR›	1.12E+08	-0.01694
27	oar3_OAR›	1.12E+08	NA
27	oar3_OAR›	1.12E+08	0.033653
27	oar3_OAR›	1.12E+08	2.97E-05
27	oar3_OAR›	1.12E+08	-0.03041
27	oar3_OAR›	1.12E+08	0.040353
27	oar3_OAR›	1.12E+08	0.038231
27	oar3_OAR›	1.12E+08	0.018804
27	oar3_OAR›	1.12E+08	-0.01538
27	oar3_OAR›	1.12E+08	-0.00882
27	oar3_OAR›	1.12E+08	0.007039
27	oar3_OAR›	1.12E+08	0.06046
27	oar3_OAR›	1.12E+08	-0.00298
27	oar3_OAR›	1.12E+08	0.018804
27	oar3_OAR›	1.12E+08	-0.02575
27	oar3_OAR›	1.12E+08	0.007039
27	oar3_OAR›	1.12E+08	0.007039
27	oar3_OAR›	1.12E+08	0.004308
27	oar3_OAR›	1.12E+08	0.004308

27	oar3_OAR〉	1.12E+08	-0.01491
27	oar3_OAR〉	1.12E+08	0.008296
27	oar3_OAR〉	1.12E+08	0.054677
27	oar3_OAR〉	1.12E+08	0.022216
27	oar3_OAR〉	1.12E+08	0.016196
27	oar3_OAR〉	1.12E+08	0.004308
27	oar3_OAR〉	1.12E+08	0.01839
27	oar3_OAR〉	1.12E+08	-0.01737
27	oar3_OAR〉	1.12E+08	0.002275
27	oar3_OAR〉	1.12E+08	-0.0076
27	oar3_OAR〉	1.12E+08	0.006741
27	oar3_OAR〉	1.12E+08	-0.02551
27	oar3_OAR〉	1.12E+08	0.012392
27	oar3_OAR〉	1.12E+08	0.081627
27	oar3_OAR〉	1.12E+08	0.015624
27	oar3_OAR〉	1.12E+08	0.015624
27	oar3_OAR〉	1.12E+08	0.054286
27	oar3_OAR〉	1.12E+08	0.028715
27	oar3_OAR〉	1.12E+08	0.003337
27	oar3_OAR〉	1.12E+08	0.072265
27	oar3_OAR〉	1.12E+08	0.003337
27	oar3_OAR〉	1.12E+08	0.072265
27	oar3_OAR〉	1.12E+08	-0.02831
27	oar3_OAR〉	1.12E+08	6.17E-06
27	oar3_OAR〉	1.12E+08	-0.02831
27	oar3_OAR〉	1.12E+08	-0.02831
27	oar3_OAR〉	1.12E+08	0.072265
27	oar3_OAR〉	1.12E+08	-0.02831
27	oar3_OAR〉	1.12E+08	0.030065
27	oar3_OAR〉	1.12E+08	0.008214
27	oar3_OAR〉	1.12E+08	0.061279
27	oar3_OAR〉	1.12E+08	-0.02764
27	oar3_OAR〉	1.12E+08	-0.00209
27	oar3_OAR〉	1.12E+08	0.023172
27	oar3_OAR〉	1.12E+08	0.086636
27	oar3_OAR〉	1.12E+08	0.023172
27	oar3_OAR〉	1.12E+08	-0.01622
27	oar3_OAR〉	1.12E+08	0.074918
27	oar3_OAR〉	1.12E+08	0.074918
27	oar3_OAR〉	1.12E+08	-0.02006
27	oar3_OAR〉	1.12E+08	-0.02006
27	oar3_OAR〉	1.12E+08	-0.0146
27	oar3_OAR〉	1.12E+08	-0.02897
27	oar3_OAR〉	1.12E+08	-0.02006
27	oar3_OAR〉	1.12E+08	-0.03857
27	oar3_OAR〉	1.12E+08	-0.02006
27	oar3_OAR〉	1.12E+08	-0.03349
27	oar3_OAR〉	1.12E+08	-0.03769
27	oar3_OAR〉	1.12E+08	-0.02793
27	oar3_OAR〉	1.12E+08	-0.02744
27	oar3_OAR〉	1.12E+08	-0.03086
27	oar3_OAR〉	1.12E+08	-0.02744
27	oar3_OAR〉	1.12E+08	-0.01538
27	oar3_OAR〉	1.12E+08	0.07136

27	oar3_OAR›	1.12E+08	-0.02744
27	oar3_OAR›	1.12E+08	0.023625
27	oar3_OAR›	1.12E+08	0.023625
27	oar3_OAR›	1.12E+08	0.023625
27	oar3_OAR›	1.12E+08	0.023625
27	oar3_OAR›	1.12E+08	0.023625
27	oar3_OAR›	1.12E+08	-0.02863
27	oar3_OAR›	1.12E+08	0.01448
27	oar3_OAR›	1.12E+08	-0.02941
27	oar3_OAR›	1.12E+08	-0.02744
27	oar3_OAR›	1.12E+08	0.01448
27	oar3_OAR›	1.12E+08	-0.02941
27	oar3_OAR›	1.12E+08	0.023625
27	oar3_OAR›	1.12E+08	-0.0089
27	oar3_OAR›	1.12E+08	-0.0089
27	oar3_OAR›	1.12E+08	0.023625
27	oar3_OAR›	1.12E+08	-0.0089
27	oar3_OAR›	1.12E+08	-0.0089
27	oar3_OAR›	1.12E+08	-0.03463
27	oar3_OAR›	1.12E+08	0.058292
27	oar3_OAR›	1.12E+08	-0.03463
27	oar3_OAR›	1.12E+08	0.062289
27	oar3_OAR›	1.12E+08	0.06287
27	oar3_OAR›	1.12E+08	0.062289
27	oar3_OAR›	1.12E+08	0.054341
27	oar3_OAR›	1.12E+08	0.096155
27	oar3_OAR›	1.12E+08	0.054341
27	oar3_OAR›	1.12E+08	0.054341
27	oar3_OAR›	1.12E+08	0.123502
27	oar3_OAR›	1.12E+08	-0.02973
27	oar3_OAR›	1.12E+08	0.041519
27	oar3_OAR›	1.12E+08	0.041519
27	oar3_OAR›	1.12E+08	-0.02973
27	oar3_OAR›	1.12E+08	-0.03287
27	oar3_OAR›	1.12E+08	-0.02973
27	oar3_OAR›	1.12E+08	-0.03287
27	oar3_OAR›	1.12E+08	0.05556
27	oar3_OAR›	1.12E+08	0.041519
27	oar3_OAR›	1.12E+08	0.05556
27	oar3_OAR›	1.12E+08	0.119169
27	oar3_OAR›	1.12E+08	-0.01596
27	oar3_OAR›	1.12E+08	0.069166
27	oar3_OAR›	1.12E+08	-0.02875
27	oar3_OAR›	1.12E+08	0.091958
27	oar3_OAR›	1.12E+08	0.005553
27	oar3_OAR›	1.12E+08	-0.02087
27	oar3_OAR›	1.12E+08	0.102167
27	oar3_OAR›	1.12E+08	0.146975
27	oar3_OAR›	1.12E+08	-0.02087
27	oar3_OAR›	1.12E+08	-0.02087
27	oar3_OAR›	1.12E+08	0.102167
27	oar3_OAR›	1.12E+08	0.102167
27	oar3_OAR›	1.12E+08	0.102167
27	oar3_OAR›	1.12E+08	0.080073

27	oar3_OAR〉	1.12E+08	0.061278
27	oar3_OAR〉	1.12E+08	0.061278
27	oar3_OAR〉	1.12E+08	0.061278
27	oar3_OAR〉	1.12E+08	-0.01836
27	oar3_OAR〉	1.12E+08	0.086799
27	oar3_OAR〉	1.12E+08	-0.01012
27	oar3_OAR〉	1.12E+08	0.080073
27	oar3_OAR〉	1.12E+08	0.080073
27	oar3_OAR〉	1.12E+08	0.086799
27	oar3_OAR〉	1.13E+08	0.080073
27	oar3_OAR〉	1.13E+08	0.080073
27	oar3_OAR〉	1.13E+08	-0.01012
27	oar3_OAR〉	1.13E+08	0.006953
27	oar3_OAR〉	1.13E+08	0.006953
27	oar3_OAR〉	1.13E+08	0.001274
27	oar3_OAR〉	1.13E+08	-0.01012
27	oar3_OAR〉	1.13E+08	-0.02442
27	oar3_OAR〉	1.13E+08	0.064284
27	oar3_OAR〉	1.13E+08	0.064284
27	oar3_OAR〉	1.13E+08	0.09096
27	oar3_OAR〉	1.13E+08	-0.04195
27	oar3_OAR〉	1.13E+08	0.064284
27	oar3_OAR〉	1.13E+08	0.064284
27	oar3_OAR〉	1.13E+08	0.064284
27	oar3_OAR〉	1.13E+08	0.064284
27	oar3_OAR〉	1.13E+08	0.064284
27	oar3_OAR〉	1.13E+08	0.034035
27	oar3_OAR〉	1.13E+08	0.063401
27	oar3_OAR〉	1.13E+08	0.055993
27	oar3_OAR〉	1.13E+08	0.022323
27	oar3_OAR〉	1.13E+08	-0.00462
27	oar3_OAR〉	1.13E+08	0.04569
27	oar3_OAR〉	1.13E+08	-0.01859
27	oar3_OAR〉	1.13E+08	-0.03234
27	oar3_OAR〉	1.13E+08	-0.00901
27	oar3_OAR〉	1.13E+08	-0.00413
27	oar3_OAR〉	1.13E+08	0.032597
27	oar3_OAR〉	1.13E+08	0.051517
27	oar3_OAR〉	1.13E+08	0.034967
27	oar3_OAR〉	1.13E+08	0.034967
27	oar3_OAR〉	1.13E+08	0.013189
27	oar3_OAR〉	1.13E+08	-0.02867
27	oar3_OAR〉	1.13E+08	-0.03806
27	oar3_OAR〉	1.13E+08	-0.03508
27	oar3_OAR〉	1.13E+08	-0.02127
27	oar3_OAR〉	1.13E+08	-0.03819
27	oar3_OAR〉	1.13E+08	-0.03314
27	oar3_OAR〉	1.13E+08	-0.02603
27	oar3_OAR〉	1.13E+08	-0.01757
27	oar3_OAR〉	1.13E+08	0.049617
27	oar3_OAR〉	1.13E+08	0.021134
27	oar3_OAR〉	1.13E+08	0.050888
27	oar3_OAR〉	1.13E+08	0.021027
27	oar3_OAR〉	1.13E+08	0.063971

27	oar3_OAR›	1.13E+08	-0.02995
27	oar3_OAR›	1.13E+08	0.041344
27	oar3_OAR›	1.13E+08	0.041344
27	oar3_OAR›	1.13E+08	0.063971
27	oar3_OAR›	1.13E+08	0.058103
27	oar3_OAR›	1.13E+08	0.043853
27	oar3_OAR›	1.13E+08	-0.03099
27	oar3_OAR›	1.13E+08	-0.03099
27	oar3_OAR›	1.13E+08	-0.03284
27	oar3_OAR›	1.13E+08	-0.03681
27	oar3_OAR›	1.13E+08	0.061601
27	oar3_OAR›	1.13E+08	0.029875
27	oar3_OAR›	1.13E+08	-0.01925
27	oar3_OAR›	1.13E+08	0.057489
27	oar3_OAR›	1.13E+08	0.110456
27	oar3_OAR›	1.13E+08	0.046782
27	oar3_OAR›	1.13E+08	0.095089
27	oar3_OAR›	1.13E+08	0.044595
27	oar3_OAR›	1.13E+08	0.011103
27	oar3_OAR›	1.13E+08	-0.03498
27	oar3_OAR›	1.13E+08	-0.02981
27	oar3_OAR›	1.13E+08	-0.04077
27	oar3_OAR›	1.13E+08	0.005477
27	oar3_OAR›	1.13E+08	0.005477
27	oar3_OAR›	1.13E+08	0.006708
27	oar3_OAR›	1.13E+08	-0.04221
27	oar3_OAR›	1.13E+08	-0.01527
27	oar3_OAR›	1.13E+08	-0.02964
27	oar3_OAR›	1.13E+08	-0.0214
27	oar3_OAR›	1.13E+08	-0.0214
27	oar3_OAR›	1.13E+08	-0.03745
27	oar3_OAR›	1.13E+08	-0.0314
27	oar3_OAR›	1.13E+08	-0.0314
27	oar3_OAR›	1.13E+08	-0.04084
27	oar3_OAR›	1.13E+08	-0.04084
27	oar3_OAR›	1.13E+08	-0.04039
27	oar3_OAR›	1.13E+08	-0.0314
27	oar3_OAR›	1.13E+08	-0.01018
27	oar3_OAR›	1.13E+08	-0.02053
27	oar3_OAR›	1.13E+08	-0.03041
27	oar3_OAR›	1.13E+08	-0.02053
27	oar3_OAR›	1.13E+08	-0.03041
27	oar3_OAR›	1.13E+08	0.009985
27	oar3_OAR›	1.13E+08	0.009985
27	oar3_OAR›	1.13E+08	-0.00751
27	oar3_OAR›	1.13E+08	-0.03776
27	oar3_OAR›	1.13E+08	-0.03267
27	oar3_OAR›	1.13E+08	-0.04135
27	oar3_OAR›	1.13E+08	-0.02748
27	oar3_OAR›	1.13E+08	-0.03298
27	oar3_OAR›	1.13E+08	0.005137
27	oar3_OAR›	1.13E+08	-0.02974
27	oar3_OAR›	1.13E+08	-0.02974
27	oar3_OAR›	1.13E+08	0.023064

27	oar3_OAR〉	1.13E+08	-0.01739
27	oar3_OAR〉	1.13E+08	0.002868
27	oar3_OAR〉	1.13E+08	NA
27	oar3_OAR〉	1.13E+08	0.002868
27	oar3_OAR〉	1.13E+08	-0.03058
27	oar3_OAR〉	1.13E+08	-0.03456
27	oar3_OAR〉	1.13E+08	-0.03456
27	oar3_OAR〉	1.13E+08	-0.00598
27	oar3_OAR〉	1.13E+08	0.008296
27	oar3_OAR〉	1.13E+08	-0.0353
27	oar3_OAR〉	1.13E+08	0.057401
27	oar3_OAR〉	1.13E+08	0.057401
27	oar3_OAR〉	1.13E+08	0.057401
27	oar3_OAR〉	1.13E+08	0.038594
27	oar3_OAR〉	1.13E+08	0.057401
27	oar3_OAR〉	1.13E+08	0.03967
27	oar3_OAR〉	1.13E+08	0.009572
27	oar3_OAR〉	1.13E+08	0.030424
27	oar3_OAR〉	1.13E+08	-0.01649
27	oar3_OAR〉	1.13E+08	-0.03098
27	oar3_OAR〉	1.13E+08	0.020467
27	oar3_OAR〉	1.13E+08	-0.01689
27	oar3_OAR〉	1.13E+08	-0.03231
27	oar3_OAR〉	1.13E+08	-0.00868
27	oar3_OAR〉	1.13E+08	-0.01309
27	oar3_OAR〉	1.13E+08	-0.02974
27	oar3_OAR〉	1.13E+08	-0.01567
27	oar3_OAR〉	1.13E+08	-0.02184
27	oar3_OAR〉	1.13E+08	0.007497
27	oar3_OAR〉	1.13E+08	0.02875
27	oar3_OAR〉	1.13E+08	0.007497
27	oar3_OAR〉	1.13E+08	0.007497
27	oar3_OAR〉	1.13E+08	0.007497
27	oar3_OAR〉	1.13E+08	0.007497
27	oar3_OAR〉	1.13E+08	0.011116
27	oar3_OAR〉	1.13E+08	0.011116
27	oar3_OAR〉	1.13E+08	0.016003
27	oar3_OAR〉	1.13E+08	-0.01736
27	oar3_OAR〉	1.13E+08	0.050638
27	oar3_OAR〉	1.13E+08	0.00937
27	oar3_OAR〉	1.13E+08	0.128853
27	oar3_OAR〉	1.13E+08	0.061319
27	oar3_OAR〉	1.13E+08	0.061319
27	oar3_OAR〉	1.13E+08	0.095373
27	oar3_OAR〉	1.13E+08	6.16E-06
27	oar3_OAR〉	1.13E+08	-0.00527
27	oar3_OAR〉	1.13E+08	0.005909
27	oar3_OAR〉	1.14E+08	0.048196
27	oar3_OAR〉	1.14E+08	0.035383
27	oar3_OAR〉	1.14E+08	0.028
27	oar3_OAR〉	1.14E+08	0.034052
27	oar3_OAR〉	1.14E+08	0.135818
27	oar3_OAR〉	1.14E+08	0.028688
27	oar3_OAR〉	1.14E+08	0.00481

27	oar3_OAR〉	1.14E+08	-0.02919
27	oar3_OAR〉	1.14E+08	-0.0268
27	oar3_OAR〉	1.14E+08	-0.02919
27	oar3_OAR〉	1.14E+08	-0.02242
27	oar3_OAR〉	1.14E+08	NA
27	oar3_OAR〉	1.14E+08	-0.03248
27	oar3_OAR〉	1.14E+08	0.085572
27	oar3_OAR〉	1.14E+08	0.107121
27	oar3_OAR〉	1.14E+08	0.029821
27	oar3_OAR〉	1.14E+08	0.070374
27	oar3_OAR〉	1.14E+08	-0.00712
27	oar3_OAR〉	1.14E+08	-0.02044
27	oar3_OAR〉	1.14E+08	0.003093
27	oar3_OAR〉	1.14E+08	0.003093
27	oar3_OAR〉	1.14E+08	-0.00712
27	oar3_OAR〉	1.14E+08	0.075382
27	oar3_OAR〉	1.14E+08	0.032217
27	oar3_OAR〉	1.14E+08	0.013963
27	oar3_OAR〉	1.14E+08	-0.01002
27	oar3_OAR〉	1.14E+08	-0.01485
27	oar3_OAR〉	1.14E+08	-0.01485
27	oar3_OAR〉	1.14E+08	-0.0254
27	oar3_OAR〉	1.14E+08	-0.0148
27	oar3_OAR〉	1.14E+08	-0.02237
27	oar3_OAR〉	1.14E+08	-0.01739
27	oar3_OAR〉	1.14E+08	-0.01739
27	oar3_OAR〉	1.14E+08	-0.00328
27	oar3_OAR〉	1.14E+08	-0.02425
27	oar3_OAR〉	1.14E+08	0.034967
27	oar3_OAR〉	1.14E+08	0.054262
27	oar3_OAR〉	1.14E+08	-0.00895
27	oar3_OAR〉	1.14E+08	0.026078
27	oar3_OAR〉	1.14E+08	-0.02893
27	oar3_OAR〉	1.14E+08	0.040531
27	oar3_OAR〉	1.14E+08	0.051834
27	oar3_OAR〉	1.14E+08	-0.01423
27	oar3_OAR〉	1.14E+08	-0.02017
27	oar3_OAR〉	1.14E+08	-0.0256
27	oar3_OAR〉	1.14E+08	0.019294
27	oar3_OAR〉	1.14E+08	0.019294
27	oar3_OAR〉	1.14E+08	-0.00097
27	oar3_OAR〉	1.14E+08	-0.01859
27	oar3_OAR〉	1.14E+08	-0.00097
27	oar3_OAR〉	1.14E+08	-0.00097
27	oar3_OAR〉	1.14E+08	-0.01527
27	oar3_OAR〉	1.14E+08	-0.01527
27	oar3_OAR〉	1.14E+08	-0.01527
27	oar3_OAR〉	1.14E+08	-0.02118
27	oar3_OAR〉	1.14E+08	0.057005
27	oar3_OAR〉	1.14E+08	-0.00287
27	oar3_OAR〉	1.14E+08	0.021324
27	oar3_OAR〉	1.14E+08	-0.02645
27	oar3_OAR〉	1.14E+08	0.003874
27	oar3_OAR〉	1.14E+08	0.056349

27	oar3_OAR〉	1.14E+08	0.052414
27	oar3_OAR〉	1.14E+08	-0.01525
27	oar3_OAR〉	1.14E+08	0.010518
27	oar3_OAR〉	1.14E+08	-0.01043
27	oar3_OAR〉	1.14E+08	0.050477
27	oar3_OAR〉	1.14E+08	0.018328
27	oar3_OAR〉	1.14E+08	0.017052
27	oar3_OAR〉	1.14E+08	0.008932
27	oar3_OAR〉	1.14E+08	-0.0196
27	oar3_OAR〉	1.14E+08	0.033773
27	oar3_OAR〉	1.14E+08	-0.02739
27	oar3_OAR〉	1.14E+08	-0.03389
27	oar3_OAR〉	1.14E+08	0.036764
27	oar3_OAR〉	1.14E+08	0.013656
27	oar3_OAR〉	1.14E+08	0.019236
27	oar3_OAR〉	1.14E+08	0.018687
27	oar3_OAR〉	1.14E+08	0.039013
27	oar3_OAR〉	1.14E+08	-0.01252
27	oar3_OAR〉	1.14E+08	-0.02606
27	oar3_OAR〉	1.14E+08	0.000459
27	oar3_OAR〉	1.14E+08	-0.02807
27	oar3_OAR〉	1.14E+08	-0.02806
27	oar3_OAR〉	1.14E+08	0.009812
27	oar3_OAR〉	1.14E+08	0.094733
27	oar3_OAR〉	1.14E+08	0.094733
27	oar3_OAR〉	1.14E+08	-0.03842
27	oar3_OAR〉	1.14E+08	-0.02989
27	oar3_OAR〉	1.14E+08	-0.0321
27	oar3_OAR〉	1.14E+08	-0.01451
27	oar3_OAR〉	1.14E+08	-0.00606
27	oar3_OAR〉	1.14E+08	-0.0259
27	oar3_OAR〉	1.14E+08	0.00481
27	oar3_OAR〉	1.14E+08	-0.01027
27	oar3_OAR〉	1.14E+08	-0.03226
27	oar3_OAR〉	1.14E+08	-0.00956
27	oar3_OAR〉	1.14E+08	-0.03709
27	oar3_OAR〉	1.14E+08	-0.02338
27	oar3_OAR〉	1.14E+08	0.029336
27	oar3_OAR〉	1.14E+08	0.046345
27	oar3_OAR〉	1.14E+08	-0.01485
27	oar3_OAR〉	1.14E+08	-0.03601
27	oar3_OAR〉	1.14E+08	-0.03601
27	oar3_OAR〉	1.14E+08	-0.00356
27	oar3_OAR〉	1.14E+08	0.02024
27	oar3_OAR〉	1.14E+08	-0.01219
27	oar3_OAR〉	1.14E+08	0.050308
27	oar3_OAR〉	1.14E+08	-0.00616
27	oar3_OAR〉	1.14E+08	-0.03849
27	oar3_OAR〉	1.14E+08	-0.02782
27	oar3_OAR〉	1.14E+08	-0.02782
27	oar3_OAR〉	1.14E+08	-0.01866
27	oar3_OAR〉	1.14E+08	-0.02782
27	oar3_OAR〉	1.14E+08	-0.02782
27	oar3_OAR〉	1.14E+08	-0.00347

27	oar3_OAR〉	1.14E+08	0.000821
27	oar3_OAR〉	1.14E+08	-0.00401
27	oar3_OAR〉	1.14E+08	-0.00901
27	oar3_OAR〉	1.14E+08	-0.01859
27	oar3_OAR〉	1.14E+08	-0.01154
27	oar3_OAR〉	1.14E+08	-0.00754
27	oar3_OAR〉	1.14E+08	0.002967
27	oar3_OAR〉	1.14E+08	0.002967
27	oar3_OAR〉	1.14E+08	-0.02672
27	oar3_OAR〉	1.14E+08	-0.02828
27	oar3_OAR〉	1.14E+08	-0.02828
27	oar3_OAR〉	1.14E+08	-0.02828
27	oar3_OAR〉	1.14E+08	-0.03075
27	oar3_OAR〉	1.14E+08	-0.03075
27	oar3_OAR〉	1.14E+08	-0.03075
27	oar3_OAR〉	1.14E+08	-0.01485
27	oar3_OAR〉	1.14E+08	-0.02348
27	oar3_OAR〉	1.14E+08	-0.02348
27	oar3_OAR〉	1.14E+08	-0.02348
27	oar3_OAR〉	1.14E+08	-0.00783
27	oar3_OAR〉	1.14E+08	-0.02782
27	oar3_OAR〉	1.14E+08	0.082222
27	oar3_OAR〉	1.14E+08	-0.00094
27	oar3_OAR〉	1.14E+08	-0.02782
27	oar3_OAR〉	1.14E+08	0.056122
27	oar3_OAR〉	1.14E+08	-0.03118
27	oar3_OAR〉	1.14E+08	0.097408
27	oar3_OAR〉	1.14E+08	0.101522
27	oar3_OAR〉	1.14E+08	0.044724
27	oar3_OAR〉	1.14E+08	0.023093
27	oar3_OAR〉	1.14E+08	-0.02339
27	oar3_OAR〉	1.14E+08	-0.01529
27	oar3_OAR〉	1.15E+08	-0.02339
27	oar3_OAR〉	1.15E+08	5.87E-06
27	oar3_OAR〉	1.15E+08	-0.03857
27	oar3_OAR〉	1.15E+08	0.030202
27	oar3_OAR〉	1.15E+08	-0.02577
27	oar3_OAR〉	1.15E+08	-0.00421
27	oar3_OAR〉	1.15E+08	0.012558
27	oar3_OAR〉	1.15E+08	-0.02577
27	oar3_OAR〉	1.15E+08	0.145463
27	oar3_OAR〉	1.15E+08	-0.02577
27	oar3_OAR〉	1.15E+08	-0.01954
27	oar3_OAR〉	1.15E+08	-0.01963
27	oar3_OAR〉	1.15E+08	-0.01473
27	oar3_OAR〉	1.15E+08	-0.03062
27	oar3_OAR〉	1.15E+08	-0.01209
27	oar3_OAR〉	1.15E+08	-0.01327
27	oar3_OAR〉	1.15E+08	-0.01209
27	oar3_OAR〉	1.15E+08	-0.01209
27	oar3_OAR〉	1.15E+08	-0.02152
27	oar3_OAR〉	1.15E+08	-0.01473
27	oar3_OAR〉	1.15E+08	-0.02152
27	oar3_OAR〉	1.15E+08	0.025449

27	oar3_OAR)	1.15E+08	-0.01866
27	oar3_OAR)	1.15E+08	-0.03606
27	oar3_OAR)	1.15E+08	0.012269
27	oar3_OAR)	1.15E+08	-0.00375
27	oar3_OAR)	1.15E+08	-0.01755
27	oar3_OAR)	1.15E+08	-0.02465
27	oar3_OAR)	1.15E+08	0.062599
27	oar3_OAR)	1.15E+08	0.080789
27	oar3_OAR)	1.15E+08	0.090916
27	oar3_OAR)	1.15E+08	0.019099
27	oar3_OAR)	1.15E+08	0.063569
27	oar3_OAR)	1.15E+08	0.037354
27	oar3_OAR)	1.15E+08	-0.02661
27	oar3_OAR)	1.15E+08	0.000359
27	oar3_OAR)	1.15E+08	-0.02091
27	oar3_OAR)	1.15E+08	0.023496
27	oar3_OAR)	1.15E+08	0.003654
27	oar3_OAR)	1.15E+08	0.003654
27	oar3_OAR)	1.15E+08	-0.00012
27	oar3_OAR)	1.15E+08	-0.03518
27	oar3_OAR)	1.15E+08	-0.02899
27	oar3_OAR)	1.15E+08	-0.03554
27	oar3_OAR)	1.15E+08	0.019123
27	oar3_OAR)	1.15E+08	0.02286
27	oar3_OAR)	1.15E+08	-0.03193
27	oar3_OAR)	1.15E+08	-0.00501
27	oar3_OAR)	1.15E+08	-0.02707
27	oar3_OAR)	1.15E+08	0.02286
27	oar3_OAR)	1.15E+08	0.037321
27	oar3_OAR)	1.15E+08	0.078078
27	oar3_OAR)	1.15E+08	0.008007
27	oar3_OAR)	1.15E+08	0.163707
27	oar3_OAR)	1.15E+08	0.008007
27	oar3_OAR)	1.15E+08	0.04522
27	oar3_OAR)	1.15E+08	0.001262
27	oar3_OAR)	1.15E+08	0.131175
27	oar3_OAR)	1.15E+08	0.131175
27	oar3_OAR)	1.15E+08	0.037321
27	oar3_OAR)	1.15E+08	0.037321
27	oar3_OAR)	1.15E+08	0.131175
27	oar3_OAR)	1.15E+08	0.159032
27	oar3_OAR)	1.15E+08	-0.02688
27	oar3_OAR)	1.15E+08	-0.00096
27	oar3_OAR)	1.15E+08	-0.02168
27	oar3_OAR)	1.15E+08	-0.02951
27	oar3_OAR)	1.15E+08	-0.02051
27	oar3_OAR)	1.15E+08	-0.02724
27	oar3_OAR)	1.15E+08	0.016028
27	oar3_OAR)	1.15E+08	0.027509
27	oar3_OAR)	1.15E+08	0.000954
27	oar3_OAR)	1.15E+08	0.013571
27	oar3_OAR)	1.15E+08	0.008971
27	oar3_OAR)	1.15E+08	0.013571
27	oar3_OAR)	1.15E+08	0.013571

27	oar3_OAR〉	1.15E+08	0.092019
27	oar3_OAR〉	1.15E+08	-0.01264
27	oar3_OAR〉	1.15E+08	-0.02551
27	oar3_OAR〉	1.15E+08	-0.02551
27	oar3_OAR〉	1.15E+08	-0.01063
27	oar3_OAR〉	1.15E+08	-0.0141
27	oar3_OAR〉	1.15E+08	-0.02783
27	oar3_OAR〉	1.15E+08	0.032012
27	oar3_OAR〉	1.15E+08	-0.02981
27	oar3_OAR〉	1.15E+08	0.108436
27	oar3_OAR〉	1.15E+08	0.031894
27	oar3_OAR〉	1.15E+08	0.007523
27	oar3_OAR〉	1.15E+08	0.007523
27	oar3_OAR〉	1.15E+08	-0.02973
27	oar3_OAR〉	1.15E+08	-0.01545
27	oar3_OAR〉	1.15E+08	-0.02229
27	oar3_OAR〉	1.15E+08	0.057067
27	oar3_OAR〉	1.15E+08	0.121825
27	oar3_OAR〉	1.15E+08	-0.02229
27	oar3_OAR〉	1.15E+08	0.09111
27	oar3_OAR〉	1.15E+08	0.082663
27	oar3_OAR〉	1.15E+08	0.031946
27	oar3_OAR〉	1.15E+08	0.000363
27	oar3_OAR〉	1.15E+08	-0.02575
27	oar3_OAR〉	1.15E+08	-0.02276
27	oar3_OAR〉	1.15E+08	-0.00031
27	oar3_OAR〉	1.15E+08	0.009248
27	oar3_OAR〉	1.15E+08	0.015483
27	oar3_OAR〉	1.15E+08	0.015483
27	oar3_OAR〉	1.15E+08	-0.00239
27	oar3_OAR〉	1.15E+08	-0.00239
27	oar3_OAR〉	1.15E+08	-0.036
27	oar3_OAR〉	1.15E+08	0.007127
27	oar3_OAR〉	1.15E+08	0.007127
27	oar3_OAR〉	1.15E+08	-0.03842
27	oar3_OAR〉	1.15E+08	-0.03842
27	oar3_OAR〉	1.15E+08	-0.03281
27	oar3_OAR〉	1.15E+08	-0.03281
27	oar3_OAR〉	1.15E+08	0.006027
27	oar3_OAR〉	1.15E+08	-0.03196
27	oar3_OAR〉	1.15E+08	0.02438
27	oar3_OAR〉	1.15E+08	-0.03281
27	oar3_OAR〉	1.15E+08	0.050262
27	oar3_OAR〉	1.15E+08	0.033354
27	oar3_OAR〉	1.15E+08	0.033354
27	oar3_OAR〉	1.15E+08	0.033354
27	oar3_OAR〉	1.15E+08	-0.02848
27	oar3_OAR〉	1.15E+08	-0.02086
27	oar3_OAR〉	1.15E+08	-0.00952
27	oar3_OAR〉	1.15E+08	0.005218
27	oar3_OAR〉	1.15E+08	0.017521
27	oar3_OAR〉	1.15E+08	2.97E-05
27	oar3_OAR〉	1.15E+08	0.076404
27	oar3_OAR〉	1.15E+08	0.076404

27	oar3_OAR〉	1.15E+08	0.011164
27	oar3_OAR〉	1.15E+08	0.036857
27	oar3_OAR〉	1.15E+08	-0.02413
27	oar3_OAR〉	1.15E+08	-0.00096
27	oar3_OAR〉	1.15E+08	-0.02782
27	oar3_OAR〉	1.15E+08	-0.01955
27	oar3_OAR〉	1.15E+08	-0.02744
27	oar3_OAR〉	1.15E+08	-0.02593
27	oar3_OAR〉	1.15E+08	-0.02593
27	oar3_OAR〉	1.15E+08	-0.01406
27	oar3_OAR〉	1.15E+08	-0.02051
27	oar3_OAR〉	1.15E+08	0.017271
27	oar3_OAR〉	1.15E+08	-0.01126
27	oar3_OAR〉	1.15E+08	0.063627
27	oar3_OAR〉	1.15E+08	-0.02726
27	oar3_OAR〉	1.15E+08	0.062739
27	oar3_OAR〉	1.15E+08	0.130345
27	oar3_OAR〉	1.15E+08	0.130345
27	oar3_OAR〉	1.15E+08	0.045521
27	oar3_OAR〉	1.15E+08	0.045521
27	oar3_OAR〉	1.15E+08	8.73E-05
27	oar3_OAR〉	1.15E+08	-0.02471
27	oar3_OAR〉	1.15E+08	0.049985
27	oar3_OAR〉	1.15E+08	-0.02471
27	oar3_OAR〉	1.15E+08	0.049985
27	oar3_OAR〉	1.15E+08	-0.02471
27	oar3_OAR〉	1.15E+08	0.049985
27	oar3_OAR〉	1.15E+08	-0.015
27	oar3_OAR〉	1.15E+08	8.73E-05
27	oar3_OAR〉	1.15E+08	8.73E-05
27	oar3_OAR〉	1.15E+08	0.049985
27	oar3_OAR〉	1.15E+08	0.051465
27	oar3_OAR〉	1.15E+08	0.019817
27	oar3_OAR〉	1.15E+08	-0.01737
27	oar3_OAR〉	1.15E+08	0.029753
27	oar3_OAR〉	1.15E+08	-0.0299
27	oar3_OAR〉	1.15E+08	0.100474
27	oar3_OAR〉	1.15E+08	0.025714
27	oar3_OAR〉	1.15E+08	-0.01737
27	oar3_OAR〉	1.15E+08	0.007973
27	oar3_OAR〉	1.15E+08	0.007973
27	oar3_OAR〉	1.15E+08	-0.0299
27	oar3_OAR〉	1.15E+08	-0.0055
27	oar3_OAR〉	1.15E+08	0.138926
27	oar3_OAR〉	1.15E+08	0.138926
27	oar3_OAR〉	1.15E+08	0.138926
27	oar3_OAR〉	1.15E+08	0.138926
27	oar3_OAR〉	1.15E+08	0.138926
27	oar3_OAR〉	1.15E+08	0.058238
27	oar3_OAR〉	1.15E+08	-0.02388
27	oar3_OAR〉	1.15E+08	-0.00768
27	oar3_OAR〉	1.15E+08	0.016028
27	oar3_OAR〉	1.16E+08	-0.00768
27	oar3_OAR〉	1.16E+08	0.043995

27	oar3_OAR›	1.16E+08	0.075383
27	oar3_OAR›	1.16E+08	0.025783
27	oar3_OAR›	1.16E+08	0.135079
27	oar3_OAR›	1.16E+08	0.007181
27	oar3_OAR›	1.16E+08	-0.00187
27	oar3_OAR›	1.16E+08	-0.0298
27	oar3_OAR›	1.16E+08	0.007181
27	oar3_OAR›	1.16E+08	0.008511
27	oar3_OAR›	1.16E+08	-0.01931
27	oar3_OAR›	1.16E+08	0.001984
27	oar3_OAR›	1.16E+08	0.008511
27	oar3_OAR›	1.16E+08	0.073408
27	oar3_OAR›	1.16E+08	0.035992
27	oar3_OAR›	1.16E+08	0.109074
27	oar3_OAR›	1.16E+08	0.035992
27	oar3_OAR›	1.16E+08	0.063401
27	oar3_OAR›	1.16E+08	0.005608
27	oar3_OAR›	1.16E+08	0.053548
27	oar3_OAR›	1.16E+08	0.005608
27	oar3_OAR›	1.16E+08	0.045933
27	oar3_OAR›	1.16E+08	0.005608
27	oar3_OAR›	1.16E+08	0.018157
27	oar3_OAR›	1.16E+08	0.018157
27	oar3_OAR›	1.16E+08	0.005608
27	oar3_OAR›	1.16E+08	0.005608
27	oar3_OAR›	1.16E+08	0.005608
27	oar3_OAR›	1.16E+08	0.005608
27	oar3_OAR›	1.16E+08	0.038816
27	oar3_OAR›	1.16E+08	0.072516
27	oar3_OAR›	1.16E+08	0.038816
27	oar3_OAR›	1.16E+08	0.046169
27	oar3_OAR›	1.16E+08	0.046169
27	oar3_OAR›	1.16E+08	0.009192
27	oar3_OAR›	1.16E+08	0.060911
27	oar3_OAR›	1.16E+08	0.005608
27	oar3_OAR›	1.16E+08	0.009192
27	oar3_OAR›	1.16E+08	0.005608
27	oar3_OAR›	1.16E+08	0.005608
27	oar3_OAR›	1.16E+08	-0.01665
27	oar3_OAR›	1.16E+08	0.016176
27	oar3_OAR›	1.16E+08	0.025783
27	oar3_OAR›	1.16E+08	-0.00915
27	oar3_OAR›	1.16E+08	0.071622
27	oar3_OAR›	1.16E+08	0.034256
27	oar3_OAR›	1.16E+08	0.00834
27	oar3_OAR›	1.16E+08	0.022924
27	oar3_OAR›	1.16E+08	0.022924
27	oar3_OAR›	1.16E+08	0.003944
27	oar3_OAR›	1.16E+08	0.010148
27	oar3_OAR›	1.16E+08	-0.00389
27	oar3_OAR›	1.16E+08	-0.00928
27	oar3_OAR›	1.16E+08	0.0348
27	oar3_OAR›	1.16E+08	-0.00928
27	oar3_OAR›	1.16E+08	0.0348

27	oar3_OAR〉	1.16E+08	0.017373
27	oar3_OAR〉	1.16E+08	0.007039
27	oar3_OAR〉	1.16E+08	0.021523
27	oar3_OAR〉	1.16E+08	0.013555
27	oar3_OAR〉	1.16E+08	0.021523
27	oar3_OAR〉	1.16E+08	-0.03042
27	oar3_OAR〉	1.16E+08	-0.00169
27	oar3_OAR〉	1.16E+08	-0.02546
27	oar3_OAR〉	1.16E+08	-0.02695
27	oar3_OAR〉	1.16E+08	-0.01549
27	oar3_OAR〉	1.16E+08	-0.01549
27	oar3_OAR〉	1.16E+08	-0.01273
27	oar3_OAR〉	1.16E+08	-0.01834
27	oar3_OAR〉	1.16E+08	-0.01549
27	oar3_OAR〉	1.16E+08	-0.01549
27	oar3_OAR〉	1.16E+08	-0.02695
27	oar3_OAR〉	1.16E+08	-0.01549
27	oar3_OAR〉	1.16E+08	0.026542
27	oar3_OAR〉	1.16E+08	0.07558
27	oar3_OAR〉	1.16E+08	-0.02235
27	oar3_OAR〉	1.16E+08	-0.01075
27	oar3_OAR〉	1.16E+08	-0.00791
27	oar3_OAR〉	1.16E+08	-0.00791
27	oar3_OAR〉	1.16E+08	-0.02184
27	oar3_OAR〉	1.16E+08	-0.01588
27	oar3_OAR〉	1.16E+08	-0.01588
27	oar3_OAR〉	1.16E+08	-0.02846
27	oar3_OAR〉	1.16E+08	0.002868
27	oar3_OAR〉	1.16E+08	0.01171
27	oar3_OAR〉	1.16E+08	-0.0287
27	oar3_OAR〉	1.16E+08	-0.01252
27	oar3_OAR〉	1.16E+08	0.017788
27	oar3_OAR〉	1.16E+08	0.023661
27	oar3_OAR〉	1.16E+08	-0.01175
27	oar3_OAR〉	1.16E+08	0.007385
27	oar3_OAR〉	1.16E+08	-0.01069
27	oar3_OAR〉	1.16E+08	-0.00209
27	oar3_OAR〉	1.16E+08	-0.00058
27	oar3_OAR〉	1.16E+08	-0.00058
27	oar3_OAR〉	1.16E+08	-0.00058
27	oar3_OAR〉	1.16E+08	########
27	oar3_OAR〉	1.16E+08	-0.03142
27	oar3_OAR〉	1.16E+08	-0.01567
27	oar3_OAR〉	1.16E+08	-0.00508
27	oar3_OAR〉	1.16E+08	-0.03688
27	oar3_OAR〉	1.16E+08	0.071155
27	oar3_OAR〉	1.16E+08	0.071155
27	oar3_OAR〉	1.16E+08	0.143566
27	oar3_OAR〉	1.16E+08	0.119303
27	oar3_OAR〉	1.16E+08	0.004597
27	oar3_OAR〉	1.16E+08	0.060033
27	oar3_OAR〉	1.16E+08	0.021381
27	oar3_OAR〉	1.16E+08	0.030891
27	oar3_OAR〉	1.16E+08	0.012542

27	oar3_OAR›	1.16E+08	0.016271
27	oar3_OAR›	1.16E+08	0.012542
27	oar3_OAR›	1.16E+08	-0.00767
27	oar3_OAR›	1.16E+08	NA
27	oar3_OAR›	1.16E+08	0.143566
27	oar3_OAR›	1.16E+08	0.012542
27	oar3_OAR›	1.16E+08	-0.02325
27	oar3_OAR›	1.16E+08	0.02492
27	oar3_OAR›	1.16E+08	0.02492
27	oar3_OAR›	1.16E+08	0.02492
27	oar3_OAR›	1.16E+08	0.02492
27	oar3_OAR›	1.16E+08	0.02492
27	oar3_OAR›	1.16E+08	0.02492
27	oar3_OAR›	1.16E+08	0.02492
27	oar3_OAR›	1.16E+08	0.02492
27	oar3_OAR›	1.16E+08	0.02492
27	oar3_OAR›	1.16E+08	0.02492
27	oar3_OAR›	1.16E+08	0.02492
27	oar3_OAR›	1.16E+08	0.02492
27	oar3_OAR›	1.16E+08	0.02492
27	oar3_OAR›	1.16E+08	0.077203
27	oar3_OAR›	1.16E+08	0.02492
27	oar3_OAR›	1.16E+08	-0.01117
27	oar3_OAR›	1.16E+08	-0.01117
27	oar3_OAR›	1.16E+08	0.02492
27	oar3_OAR›	1.16E+08	-0.02292
27	oar3_OAR›	1.16E+08	0.005388
27	oar3_OAR›	1.16E+08	0.005388
27	oar3_OAR›	1.16E+08	0.112804
27	oar3_OAR›	1.16E+08	0.115361
27	oar3_OAR›	1.16E+08	-0.02555
27	oar3_OAR›	1.16E+08	0.066589
27	oar3_OAR›	1.16E+08	0.020939
27	oar3_OAR›	1.16E+08	0.007039
27	oar3_OAR›	1.16E+08	0.053195
27	oar3_OAR›	1.16E+08	0.020939
27	oar3_OAR›	1.16E+08	0.06346
27	oar3_OAR›	1.16E+08	0.063259
27	oar3_OAR›	1.16E+08	0.085884
27	oar3_OAR›	1.16E+08	0.003628
27	oar3_OAR›	1.16E+08	-0.03504
27	oar3_OAR›	1.16E+08	-0.03504
27	oar3_OAR›	1.16E+08	-0.03504
27	oar3_OAR›	1.16E+08	0.120022
27	oar3_OAR›	1.16E+08	-0.01173
27	oar3_OAR›	1.16E+08	-0.03504
27	oar3_OAR›	1.16E+08	-0.01121
27	oar3_OAR›	1.16E+08	0.054444
27	oar3_OAR›	1.16E+08	-0.02612
27	oar3_OAR›	1.16E+08	-0.01639
27	oar3_OAR›	1.16E+08	0.00395
27	oar3_OAR›	1.16E+08	-0.0146
27	oar3_OAR›	1.16E+08	-0.0247
27	oar3_OAR›	1.16E+08	-0.02353

27	oar3_OAR›	1.16E+08	-0.02825
27	oar3_OAR›	1.16E+08	-0.03025
27	oar3_OAR›	1.16E+08	-0.01386
27	oar3_OAR›	1.16E+08	-0.03025
27	oar3_OAR›	1.16E+08	0.144343
27	oar3_OAR›	1.16E+08	0.144343
27	oar3_OAR›	1.16E+08	-0.0334
27	oar3_OAR›	1.16E+08	0.130318
27	oar3_OAR›	1.16E+08	-0.01069
27	oar3_OAR›	1.16E+08	-0.02712
27	oar3_OAR›	1.16E+08	-0.00685
27	oar3_OAR›	1.16E+08	0.00756
27	oar3_OAR›	1.16E+08	0.004093
27	oar3_OAR›	1.16E+08	-0.003
27	oar3_OAR›	1.16E+08	0.023951
27	oar3_OAR›	1.16E+08	-0.00298
27	oar3_OAR›	1.16E+08	-0.003
27	oar3_OAR›	1.16E+08	0.015702
27	oar3_OAR›	1.16E+08	-0.0081
27	oar3_OAR›	1.16E+08	-0.025
27	oar3_OAR›	1.16E+08	-0.02636
27	oar3_OAR›	1.16E+08	0.006912
27	oar3_OAR›	1.16E+08	0.006912
27	oar3_OAR›	1.16E+08	-0.01594
27	oar3_OAR›	1.16E+08	-0.01594
27	oar3_OAR›	1.16E+08	0.064966
27	oar3_OAR›	1.16E+08	0.023951
27	oar3_OAR›	1.16E+08	0.006912
27	oar3_OAR›	1.16E+08	0.036059
27	oar3_OAR›	1.16E+08	0.004689
27	oar3_OAR›	1.16E+08	0.052705
27	oar3_OAR›	1.16E+08	-0.03143
27	oar3_OAR›	1.16E+08	0.011067
27	oar3_OAR›	1.16E+08	0.003611
27	oar3_OAR›	1.16E+08	-0.01738
27	oar3_OAR›	1.16E+08	0.085991
27	oar3_OAR›	1.16E+08	0.023093
27	oar3_OAR›	1.16E+08	-0.02114
27	oar3_OAR›	1.16E+08	-0.02114
27	oar3_OAR›	1.16E+08	0.056919
27	oar3_OAR›	1.16E+08	0.057656
27	oar3_OAR›	1.16E+08	-0.03134
27	oar3_OAR›	1.16E+08	-0.03134
27	oar3_OAR›	1.16E+08	0.05823
27	oar3_OAR›	1.16E+08	-0.01386
27	oar3_OAR›	1.17E+08	0.046169
27	oar3_OAR›	1.17E+08	NA
27	oar3_OAR›	1.17E+08	0.046169
27	oar3_OAR›	1.17E+08	-0.03237
27	oar3_OAR›	1.17E+08	-0.03377
27	oar3_OAR›	1.17E+08	-0.01389
27	oar3_OAR›	1.17E+08	0.012997
27	oar3_OAR›	1.17E+08	0.014149
27	oar3_OAR›	1.17E+08	-0.0041

27	oar3_OAR〉	1.17E+08	0.050941
27	oar3_OAR〉	1.17E+08	0.050941
27	oar3_OAR〉	1.17E+08	0.012143
27	oar3_OAR〉	1.17E+08	-0.03152
27	oar3_OAR〉	1.17E+08	-0.03152
27	oar3_OAR〉	1.17E+08	-0.03483
27	oar3_OAR〉	1.17E+08	-0.00406
27	oar3_OAR〉	1.17E+08	-0.00556
27	oar3_OAR〉	1.17E+08	-0.00431
27	oar3_OAR〉	1.17E+08	-0.00431
27	oar3_OAR〉	1.17E+08	0.025392
27	oar3_OAR〉	1.17E+08	-0.03208
27	oar3_OAR〉	1.17E+08	0.009617
27	oar3_OAR〉	1.17E+08	-0.03152
27	oar3_OAR〉	1.17E+08	0.046998
27	oar3_OAR〉	1.17E+08	0.000321
27	oar3_OAR〉	1.17E+08	0.0677
27	oar3_OAR〉	1.17E+08	-0.01777
27	oar3_OAR〉	1.17E+08	0.090865
27	oar3_OAR〉	1.17E+08	0.013119
27	oar3_OAR〉	1.17E+08	0.011186
27	oar3_OAR〉	1.17E+08	0.013119
27	oar3_OAR〉	1.17E+08	0.127277
27	oar3_OAR〉	1.17E+08	-0.02272
27	oar3_OAR〉	1.17E+08	0.037852
27	oar3_OAR〉	1.17E+08	0.025602
27	oar3_OAR〉	1.17E+08	0.00872
27	oar3_OAR〉	1.17E+08	0.030386
27	oar3_OAR〉	1.17E+08	-0.0324
27	oar3_OAR〉	1.17E+08	-0.0324
27	oar3_OAR〉	1.17E+08	0.066523
27	oar3_OAR〉	1.17E+08	0.066523
27	oar3_OAR〉	1.17E+08	0.095578
27	oar3_OAR〉	1.17E+08	0.095578
27	oar3_OAR〉	1.17E+08	-0.02703
27	oar3_OAR〉	1.17E+08	0.072479
27	oar3_OAR〉	1.17E+08	-0.0052
27	oar3_OAR〉	1.17E+08	0.037354
27	oar3_OAR〉	1.17E+08	0.019886
27	oar3_OAR〉	1.17E+08	-0.0137
27	oar3_OAR〉	1.17E+08	-0.0262
27	oar3_OAR〉	1.17E+08	-0.02294
27	oar3_OAR〉	1.17E+08	0.164518
27	oar3_OAR〉	1.17E+08	-0.00957
27	oar3_OAR〉	1.17E+08	-0.00955
27	oar3_OAR〉	1.17E+08	-0.02575
27	oar3_OAR〉	1.17E+08	0.029304
27	oar3_OAR〉	1.17E+08	-0.00817
27	oar3_OAR〉	1.17E+08	-0.02575
27	oar3_OAR〉	1.17E+08	0.023115
27	oar3_OAR〉	1.17E+08	-0.02492
27	oar3_OAR〉	1.17E+08	-0.01891
27	oar3_OAR〉	1.17E+08	0.003501
27	oar3_OAR〉	1.17E+08	-0.0308

27	oar3_OAR〉	1.17E+08	-0.01891
27	oar3_OAR〉	1.17E+08	-0.01069
27	oar3_OAR〉	1.17E+08	-0.0308
27	oar3_OAR〉	1.17E+08	0.022259
27	oar3_OAR〉	1.17E+08	0.022259
27	oar3_OAR〉	1.17E+08	0.022259
27	oar3_OAR〉	1.17E+08	0.022259
27	oar3_OAR〉	1.17E+08	-0.0308
27	oar3_OAR〉	1.17E+08	-0.0308
27	oar3_OAR〉	1.17E+08	-0.01069
27	oar3_OAR〉	1.17E+08	0.022259
27	oar3_OAR〉	1.17E+08	0.022259
27	oar3_OAR〉	1.17E+08	0.024704
27	oar3_OAR〉	1.17E+08	0.071927
27	oar3_OAR〉	1.17E+08	0.071927
27	oar3_OAR〉	1.17E+08	0.017015
27	oar3_OAR〉	1.17E+08	0.116426
27	oar3_OAR〉	1.17E+08	0.098049
27	oar3_OAR〉	1.17E+08	-0.01124
27	oar3_OAR〉	1.17E+08	-0.00352
27	oar3_OAR〉	1.17E+08	0.070622
27	oar3_OAR〉	1.17E+08	-0.00844
27	oar3_OAR〉	1.17E+08	-0.01456
27	oar3_OAR〉	1.17E+08	0.134816
27	oar3_OAR〉	1.17E+08	0.134816
27	oar3_OAR〉	1.17E+08	0.008296
27	oar3_OAR〉	1.17E+08	-0.01384
27	oar3_OAR〉	1.17E+08	0.063386
27	oar3_OAR〉	1.17E+08	-0.02993
27	oar3_OAR〉	1.17E+08	-0.02993
27	oar3_OAR〉	1.17E+08	-0.01384
27	oar3_OAR〉	1.17E+08	0.008296
27	oar3_OAR〉	1.17E+08	-0.02993
27	oar3_OAR〉	1.17E+08	0.001316
27	oar3_OAR〉	1.17E+08	0.001316
27	oar3_OAR〉	1.17E+08	-0.02047
27	oar3_OAR〉	1.17E+08	-0.03149
27	oar3_OAR〉	1.17E+08	-0.03143
27	oar3_OAR〉	1.17E+08	-0.03149
27	oar3_OAR〉	1.17E+08	0.039326
27	oar3_OAR〉	1.17E+08	-0.01872
27	oar3_OAR〉	1.17E+08	-0.03143
27	oar3_OAR〉	1.17E+08	0.008723
27	oar3_OAR〉	1.17E+08	-0.03143
27	oar3_OAR〉	1.17E+08	-0.03143
27	oar3_OAR〉	1.17E+08	-0.03149
27	oar3_OAR〉	1.17E+08	-0.03143
27	oar3_OAR〉	1.17E+08	-0.03143
27	oar3_OAR〉	1.17E+08	-0.01117
27	oar3_OAR〉	1.17E+08	0.020467
27	oar3_OAR〉	1.17E+08	0.073492
27	oar3_OAR〉	1.17E+08	-0.01567
27	oar3_OAR〉	1.17E+08	-0.02614
27	oar3_OAR〉	1.17E+08	0.008723

27	oar3_OAR〉	1.17E+08	-0.00449
27	oar3_OAR〉	1.17E+08	0.003053
27	oar3_OAR〉	1.17E+08	0.008723
27	oar3_OAR〉	1.17E+08	0.007212
27	oar3_OAR〉	1.17E+08	-0.02087
27	oar3_OAR〉	1.17E+08	-0.04061
27	oar3_OAR〉	1.17E+08	0.099948
27	oar3_OAR〉	1.17E+08	0.008723
27	oar3_OAR〉	1.17E+08	-0.00898
27	oar3_OAR〉	1.17E+08	0.003806
27	oar3_OAR〉	1.17E+08	0.003806
27	oar3_OAR〉	1.17E+08	0.003806
27	oar3_OAR〉	1.17E+08	-0.0213
27	oar3_OAR〉	1.17E+08	-0.0213
27	oar3_OAR〉	1.17E+08	-0.00324
27	oar3_OAR〉	1.17E+08	-0.03149
27	oar3_OAR〉	1.17E+08	0.064699
27	oar3_OAR〉	1.17E+08	0.107923
27	oar3_OAR〉	1.17E+08	-0.02565
27	oar3_OAR〉	1.17E+08	0.039013
27	oar3_OAR〉	1.17E+08	0.076597
27	oar3_OAR〉	1.17E+08	0.006329
27	oar3_OAR〉	1.17E+08	0.039481
27	oar3_OAR〉	1.17E+08	0.034081
27	oar3_OAR〉	1.17E+08	-0.01282
27	oar3_OAR〉	1.17E+08	-0.02108
27	oar3_OAR〉	1.17E+08	-0.02108
27	oar3_OAR〉	1.17E+08	0.034866
27	oar3_OAR〉	1.17E+08	0.006205
27	oar3_OAR〉	1.17E+08	0.094569
27	oar3_OAR〉	1.17E+08	-0.01738
27	oar3_OAR〉	1.17E+08	6.15E-05
27	oar3_OAR〉	1.17E+08	-0.00838
27	oar3_OAR〉	1.17E+08	-0.02575
27	oar3_OAR〉	1.17E+08	-0.02526
27	oar3_OAR〉	1.17E+08	-0.0245
27	oar3_OAR〉	1.17E+08	0.007039
27	oar3_OAR〉	1.17E+08	-0.00376
27	oar3_OAR〉	1.17E+08	0.011067
27	oar3_OAR〉	1.17E+08	0.039481
27	oar3_OAR〉	1.17E+08	0.037433
27	oar3_OAR〉	1.17E+08	0.034703
27	oar3_OAR〉	1.17E+08	0.075173
27	oar3_OAR〉	1.17E+08	0.046772
27	oar3_OAR〉	1.17E+08	-0.01423
27	oar3_OAR〉	1.17E+08	-0.01423
27	oar3_OAR〉	1.17E+08	-0.01423
27	oar3_OAR〉	1.17E+08	-0.01069
27	oar3_OAR〉	1.17E+08	0.008723
27	oar3_OAR〉	1.17E+08	0.155578
27	oar3_OAR〉	1.17E+08	0.162207
27	oar3_OAR〉	1.17E+08	-0.00965
27	oar3_OAR〉	1.17E+08	0.041449
27	oar3_OAR〉	1.17E+08	-0.01756

27	oar3_OAR⟩	1.17E+08	0.009734
27	oar3_OAR⟩	1.17E+08	0.022432
27	oar3_OAR⟩	1.17E+08	0.048875
27	oar3_OAR⟩	1.17E+08	0.031225
27	oar3_OAR⟩	1.17E+08	-0.02788
27	oar3_OAR⟩	1.17E+08	0.008681
27	oar3_OAR⟩	1.17E+08	-0.02688
27	oar3_OAR⟩	1.17E+08	-0.02688
27	oar3_OAR⟩	1.17E+08	0.021081
27	oar3_OAR⟩	1.17E+08	0.023492
27	oar3_OAR⟩	1.17E+08	0.023492
27	oar3_OAR⟩	1.17E+08	0.036303
27	oar3_OAR⟩	1.17E+08	0.011893
27	oar3_OAR⟩	1.17E+08	0.023167
27	oar3_OAR⟩	1.17E+08	0.005869
27	oar3_OAR⟩	1.17E+08	-0.00169
27	oar3_OAR⟩	1.17E+08	-0.03151
27	oar3_OAR⟩	1.17E+08	-0.00092
27	oar3_OAR⟩	1.17E+08	-0.03041
27	oar3_OAR⟩	1.17E+08	-0.03041
27	oar3_OAR⟩	1.17E+08	-0.01995
27	oar3_OAR⟩	1.17E+08	0.036229
27	oar3_OAR⟩	1.17E+08	0.024626
27	oar3_OAR⟩	1.17E+08	0.009825
27	oar3_OAR⟩	1.17E+08	0.01417
27	oar3_OAR⟩	1.17E+08	0.009825
27	oar3_OAR⟩	1.17E+08	0.01417
27	oar3_OAR⟩	1.17E+08	-0.00606
27	oar3_OAR⟩	1.17E+08	0.003215
27	oar3_OAR⟩	1.17E+08	-0.03471
27	oar3_OAR⟩	1.17E+08	0.033773
27	oar3_OAR⟩	1.18E+08	-0.03355
27	oar3_OAR⟩	1.18E+08	0.016329
27	oar3_OAR⟩	1.18E+08	-0.00269
27	oar3_OAR⟩	1.18E+08	-0.02933
27	oar3_OAR⟩	1.18E+08	0.013686
27	oar3_OAR⟩	1.18E+08	0.124458
27	oar3_OAR⟩	1.18E+08	0.035652
27	oar3_OAR⟩	1.18E+08	-0.02238
27	oar3_OAR⟩	1.18E+08	0.110902
27	oar3_OAR⟩	1.18E+08	-0.01178
27	oar3_OAR⟩	1.18E+08	-0.01178
27	oar3_OAR⟩	1.18E+08	-0.00406
27	oar3_OAR⟩	1.18E+08	NA
27	oar3_OAR⟩	1.18E+08	-0.02167
27	oar3_OAR⟩	1.18E+08	-0.0202
27	oar3_OAR⟩	1.18E+08	0.001737
27	oar3_OAR⟩	1.18E+08	0.001737
27	oar3_OAR⟩	1.18E+08	0.025097
27	oar3_OAR⟩	1.18E+08	-0.03706
27	oar3_OAR⟩	1.18E+08	-0.03819
27	oar3_OAR⟩	1.18E+08	NA
27	oar3_OAR⟩	1.18E+08	-0.0282
27	oar3_OAR⟩	1.18E+08	-0.02372

27	oar3_OAR〉	1.18E+08	-0.03143
27	oar3_OAR〉	1.18E+08	-0.02732
27	oar3_OAR〉	1.18E+08	-0.02209
27	oar3_OAR〉	1.18E+08	0.008291
27	oar3_OAR〉	1.18E+08	0.008291
27	oar3_OAR〉	1.18E+08	-0.03527
27	oar3_OAR〉	1.18E+08	-0.03326
27	oar3_OAR〉	1.18E+08	0.021533
27	oar3_OAR〉	1.18E+08	0.047311
27	oar3_OAR〉	1.18E+08	0.053563
27	oar3_OAR〉	1.18E+08	0.007039
27	oar3_OAR〉	1.18E+08	0.021533
27	oar3_OAR〉	1.18E+08	-0.02509
27	oar3_OAR〉	1.18E+08	-0.01895
27	oar3_OAR〉	1.18E+08	-0.03499
27	oar3_OAR〉	1.18E+08	-0.02374
27	oar3_OAR〉	1.18E+08	-0.02794
27	oar3_OAR〉	1.18E+08	0.016767
27	oar3_OAR〉	1.18E+08	0.016767
27	oar3_OAR〉	1.18E+08	0.016767
27	oar3_OAR〉	1.18E+08	-0.01587
27	oar3_OAR〉	1.18E+08	0.010621
27	oar3_OAR〉	1.18E+08	-0.03149
27	oar3_OAR〉	1.18E+08	-0.02961
27	oar3_OAR〉	1.18E+08	-0.02961
27	oar3_OAR〉	1.18E+08	-0.03043
27	oar3_OAR〉	1.18E+08	0.061278
27	oar3_OAR〉	1.18E+08	0.072367
27	oar3_OAR〉	1.18E+08	-0.01567
27	oar3_OAR〉	1.18E+08	-0.00328
27	oar3_OAR〉	1.18E+08	0.08904
27	oar3_OAR〉	1.18E+08	0.001441
27	oar3_OAR〉	1.18E+08	-0.00328
27	oar3_OAR〉	1.18E+08	0.086929
27	oar3_OAR〉	1.18E+08	0.001441
27	oar3_OAR〉	1.18E+08	0.045876
27	oar3_OAR〉	1.18E+08	0.028957
27	oar3_OAR〉	1.18E+08	0.057831
27	oar3_OAR〉	1.18E+08	0.057831
27	oar3_OAR〉	1.18E+08	-0.01234
27	oar3_OAR〉	1.18E+08	0.064242
27	oar3_OAR〉	1.18E+08	0.0139
27	oar3_OAR〉	1.18E+08	-0.02562
27	oar3_OAR〉	1.18E+08	0.054334
27	oar3_OAR〉	1.18E+08	-0.02492
27	oar3_OAR〉	1.18E+08	-0.00278
27	oar3_OAR〉	1.18E+08	-0.00723
27	oar3_OAR〉	1.18E+08	-0.01396
27	oar3_OAR〉	1.18E+08	0.06608
27	oar3_OAR〉	1.18E+08	0.06608
27	oar3_OAR〉	1.18E+08	0.042599
27	oar3_OAR〉	1.18E+08	0.005039
27	oar3_OAR〉	1.18E+08	0.033799
27	oar3_OAR〉	1.18E+08	0.033799

27	oar3_OAR›	1.18E+08	0.072923
27	oar3_OAR›	1.18E+08	-0.00953
27	oar3_OAR›	1.18E+08	-0.00603
27	oar3_OAR›	1.18E+08	0.005039
27	oar3_OAR›	1.18E+08	0.030548
27	oar3_OAR›	1.18E+08	0.047897
27	oar3_OAR›	1.18E+08	0.047897
27	oar3_OAR›	1.18E+08	0.029824
27	oar3_OAR›	1.18E+08	0.003987
27	oar3_OAR›	1.18E+08	-0.00322
27	oar3_OAR›	1.18E+08	-0.01069
27	oar3_OAR›	1.18E+08	-0.0254
27	oar3_OAR›	1.18E+08	0.038448
27	oar3_OAR›	1.18E+08	-0.02637
27	oar3_OAR›	1.18E+08	-0.02119
27	oar3_OAR›	1.18E+08	0.017537
27	oar3_OAR›	1.18E+08	-0.01762
27	oar3_OAR›	1.18E+08	0.022737
27	oar3_OAR›	1.18E+08	0.030545
27	oar3_OAR›	1.18E+08	0.022737
27	oar3_OAR›	1.18E+08	0.022737
27	oar3_OAR›	1.18E+08	0.030545
27	oar3_OAR›	1.18E+08	-0.03232
27	oar3_OAR›	1.18E+08	0.099038
27	oar3_OAR›	1.18E+08	0.01448
27	oar3_OAR›	1.18E+08	-0.0354
27	oar3_OAR›	1.18E+08	0.048657
27	oar3_OAR›	1.18E+08	0.116574
27	oar3_OAR›	1.18E+08	0.116574
27	oar3_OAR›	1.18E+08	0.017106
27	oar3_OAR›	1.18E+08	-0.0128
27	oar3_OAR›	1.18E+08	0.087444
27	oar3_OAR›	1.18E+08	0.058233
27	oar3_OAR›	1.18E+08	0.058233
27	oar3_OAR›	1.18E+08	0.005125
27	oar3_OAR›	1.18E+08	0.010937
27	oar3_OAR›	1.18E+08	-0.01567
27	oar3_OAR›	1.18E+08	-0.0122
27	oar3_OAR›	1.18E+08	-0.02425
27	oar3_OAR›	1.18E+08	-0.01033
27	oar3_OAR›	1.18E+08	-0.0059
27	oar3_OAR›	1.18E+08	-0.0059
27	oar3_OAR›	1.18E+08	-0.01456
27	oar3_OAR›	1.18E+08	-0.02271
27	oar3_OAR›	1.18E+08	0.018271
27	oar3_OAR›	1.18E+08	-0.01948
27	oar3_OAR›	1.18E+08	-0.00082
27	oar3_OAR›	1.18E+08	-0.01845
27	oar3_OAR›	1.18E+08	0.012542
27	oar3_OAR›	1.18E+08	-0.00452
27	oar3_OAR›	1.18E+08	0.068262
27	oar3_OAR›	1.18E+08	-0.01303
27	oar3_OAR›	1.18E+08	-0.03541
27	oar3_OAR›	1.18E+08	-0.03541

27	oar3_OAR〉	1.18E+08	0.029019
27	oar3_OAR〉	1.18E+08	-0.02854
27	oar3_OAR〉	1.18E+08	-0.00177
27	oar3_OAR〉	1.18E+08	0.009739
27	oar3_OAR〉	1.18E+08	0.013007
27	oar3_OAR〉	1.18E+08	-0.01745
27	oar3_OAR〉	1.18E+08	-0.00774
27	oar3_OAR〉	1.18E+08	0.018027
27	oar3_OAR〉	1.18E+08	0.0882
27	oar3_OAR〉	1.18E+08	0.094485
27	oar3_OAR〉	1.18E+08	-0.01341
27	oar3_OAR〉	1.18E+08	-0.04032
27	oar3_OAR〉	1.18E+08	-0.02606
27	oar3_OAR〉	1.18E+08	0.018459
27	oar3_OAR〉	1.18E+08	0.057489
27	oar3_OAR〉	1.18E+08	0.057489
27	oar3_OAR〉	1.18E+08	0.057489
27	oar3_OAR〉	1.18E+08	0.011193
27	oar3_OAR〉	1.18E+08	-0.02652
27	oar3_OAR〉	1.18E+08	-0.01369
27	oar3_OAR〉	1.18E+08	0.109761
27	oar3_OAR〉	1.18E+08	0.124707
27	oar3_OAR〉	1.18E+08	-0.02923
27	oar3_OAR〉	1.18E+08	0.000342
27	oar3_OAR〉	1.18E+08	0.012853
27	oar3_OAR〉	1.18E+08	-0.02584
27	oar3_OAR〉	1.18E+08	0.056326
27	oar3_OAR〉	1.18E+08	-0.01746
27	oar3_OAR〉	1.18E+08	-0.00187
27	oar3_OAR〉	1.18E+08	-0.00187
27	oar3_OAR〉	1.18E+08	-0.02362
27	oar3_OAR〉	1.18E+08	0.044724
27	oar3_OAR〉	1.18E+08	-0.01859
27	oar3_OAR〉	1.18E+08	-0.01859
27	oar3_OAR〉	1.18E+08	-0.02087
27	oar3_OAR〉	1.18E+08	0.044724
27	oar3_OAR〉	1.18E+08	-0.03324
27	oar3_OAR〉	1.18E+08	0.044724
27	oar3_OAR〉	1.18E+08	-0.01519
27	oar3_OAR〉	1.18E+08	-0.02793
27	oar3_OAR〉	1.18E+08	-0.01974
27	oar3_OAR〉	1.18E+08	0.031307
27	oar3_OAR〉	1.18E+08	-0.02793
27	oar3_OAR〉	1.18E+08	0.007039
27	oar3_OAR〉	1.18E+08	0.031307
27	oar3_OAR〉	1.18E+08	-0.02793
27	oar3_OAR〉	1.18E+08	-0.02546
27	oar3_OAR〉	1.18E+08	-0.01529
27	oar3_OAR〉	1.18E+08	-0.00508
27	oar3_OAR〉	1.18E+08	0.014822
27	oar3_OAR〉	1.18E+08	0.007039
27	oar3_OAR〉	1.18E+08	-0.02418
27	oar3_OAR〉	1.18E+08	-0.03504
27	oar3_OAR〉	1.18E+08	-0.02866

27	oar3_OAR›	1.18E+08	-0.02866
27	oar3_OAR›	1.18E+08	-0.02645
27	oar3_OAR›	1.18E+08	-0.02857
27	oar3_OAR›	1.18E+08	-0.0218
27	oar3_OAR›	1.18E+08	-0.02477
27	oar3_OAR›	1.18E+08	-0.01039
27	oar3_OAR›	1.18E+08	-0.01194
27	oar3_OAR›	1.18E+08	-0.03681
27	oar3_OAR›	1.18E+08	-0.00632
27	oar3_OAR›	1.18E+08	-0.00042
27	oar3_OAR›	1.18E+08	-0.02618
27	oar3_OAR›	1.19E+08	-0.02009
27	oar3_OAR›	1.19E+08	0.028866
27	oar3_OAR›	1.19E+08	-0.01386
27	oar3_OAR›	1.19E+08	0.033947
27	oar3_OAR›	1.19E+08	0.049209
27	oar3_OAR›	1.19E+08	0.049209
27	oar3_OAR›	1.19E+08	-0.01295
27	oar3_OAR›	1.19E+08	-0.00157
27	oar3_OAR›	1.19E+08	0.000889
27	oar3_OAR›	1.19E+08	-0.02158
27	oar3_OAR›	1.19E+08	0.000889
27	oar3_OAR›	1.19E+08	0.016028
27	oar3_OAR›	1.19E+08	-0.01423
27	oar3_OAR›	1.19E+08	-0.01031
27	oar3_OAR›	1.19E+08	-0.01069
27	oar3_OAR›	1.19E+08	0.022239
27	oar3_OAR›	1.19E+08	0.094693
27	oar3_OAR›	1.19E+08	-0.01069
27	oar3_OAR›	1.19E+08	0.10441
27	oar3_OAR›	1.19E+08	0.038288
27	oar3_OAR›	1.19E+08	0.045915
27	oar3_OAR›	1.19E+08	-0.02926
27	oar3_OAR›	1.19E+08	-0.02455
27	oar3_OAR›	1.19E+08	-0.01419
27	oar3_OAR›	1.19E+08	NA
27	oar3_OAR›	1.19E+08	0.028942
27	oar3_OAR›	1.19E+08	-0.02191
27	oar3_OAR›	1.19E+08	0.019178
27	oar3_OAR›	1.19E+08	0.029788
27	oar3_OAR›	1.19E+08	0.044724
27	oar3_OAR›	1.19E+08	0.011871
27	oar3_OAR›	1.19E+08	-0.00568
27	oar3_OAR›	1.19E+08	0.084908
27	oar3_OAR›	1.19E+08	0.101713
27	oar3_OAR›	1.19E+08	0.015985
27	oar3_OAR›	1.19E+08	-0.01316
27	oar3_OAR›	1.19E+08	0.059208
27	oar3_OAR›	1.19E+08	0.006748
27	oar3_OAR›	1.19E+08	-0.00754
27	oar3_OAR›	1.19E+08	-0.03745
27	oar3_OAR›	1.19E+08	0.003212
27	oar3_OAR›	1.19E+08	-0.00165
27	oar3_OAR›	1.19E+08	-0.02398

27	oar3_OAR⟩	1.19E+08	-0.01423
27	oar3_OAR⟩	1.19E+08	-0.02797
27	oar3_OAR⟩	1.19E+08	-0.03351
27	oar3_OAR⟩	1.19E+08	-0.0244
27	oar3_OAR⟩	1.19E+08	-0.01186
27	oar3_OAR⟩	1.19E+08	-0.02555
27	oar3_OAR⟩	1.19E+08	0.028803
27	oar3_OAR⟩	1.19E+08	0.07139
27	oar3_OAR⟩	1.19E+08	0.104169
27	oar3_OAR⟩	1.19E+08	0.014017
27	oar3_OAR⟩	1.19E+08	-0.0244
27	oar3_OAR⟩	1.19E+08	-0.01186
27	oar3_OAR⟩	1.19E+08	0.014017
27	oar3_OAR⟩	1.19E+08	-0.01218
27	oar3_OAR⟩	1.19E+08	0.096314
27	oar3_OAR⟩	1.19E+08	0.027927
27	oar3_OAR⟩	1.19E+08	0.007009
27	oar3_OAR⟩	1.19E+08	0.013189
27	oar3_OAR⟩	1.19E+08	-0.00308
27	oar3_OAR⟩	1.19E+08	0.007009
27	oar3_OAR⟩	1.19E+08	-0.01406
27	oar3_OAR⟩	1.19E+08	0.127971
27	oar3_OAR⟩	1.19E+08	0.100633
27	oar3_OAR⟩	1.19E+08	-0.00605
27	oar3_OAR⟩	1.19E+08	-0.01303
27	oar3_OAR⟩	1.19E+08	-0.00454
27	oar3_OAR⟩	1.19E+08	-0.02996
27	oar3_OAR⟩	1.19E+08	-0.02283
27	oar3_OAR⟩	1.19E+08	0.015728
27	oar3_OAR⟩	1.19E+08	0.064242
27	oar3_OAR⟩	1.19E+08	-0.02167
27	oar3_OAR⟩	1.19E+08	0.04569
27	oar3_OAR⟩	1.19E+08	0.041206
27	oar3_OAR⟩	1.19E+08	-0.00213
27	oar3_OAR⟩	1.19E+08	-0.01276
27	oar3_OAR⟩	1.19E+08	-0.01456
27	oar3_OAR⟩	1.19E+08	-0.02586
27	oar3_OAR⟩	1.19E+08	-0.01416
27	oar3_OAR⟩	1.19E+08	-0.01416
27	oar3_OAR⟩	1.19E+08	-0.0259
27	oar3_OAR⟩	1.19E+08	0.007039
27	oar3_OAR⟩	1.19E+08	-0.02487
27	oar3_OAR⟩	1.19E+08	0.045251
27	oar3_OAR⟩	1.19E+08	-0.02565
27	oar3_OAR⟩	1.19E+08	-0.01302
27	oar3_OAR⟩	1.19E+08	0.00097
27	oar3_OAR⟩	1.19E+08	0.056704
27	oar3_OAR⟩	1.19E+08	-0.02188
27	oar3_OAR⟩	1.19E+08	0.00097
27	oar3_OAR⟩	1.19E+08	0.067842
27	oar3_OAR⟩	1.19E+08	0.033486
27	oar3_OAR⟩	1.19E+08	-0.03152
27	oar3_OAR⟩	1.19E+08	-0.01496
27	oar3_OAR⟩	1.19E+08	0.06269

27	oar3_OAR〉	1.19E+08	-0.03152
27	oar3_OAR〉	1.19E+08	0.10621
27	oar3_OAR〉	1.19E+08	-0.01536
27	oar3_OAR〉	1.19E+08	-0.02863
27	oar3_OAR〉	1.19E+08	-0.00895
27	oar3_OAR〉	1.19E+08	-0.0268
27	oar3_OAR〉	1.19E+08	-0.03941
27	oar3_OAR〉	1.19E+08	-0.02694
27	oar3_OAR〉	1.19E+08	0.086746
27	oar3_OAR〉	1.19E+08	-0.02699
27	oar3_OAR〉	1.19E+08	-0.00068
27	oar3_OAR〉	1.19E+08	-0.02981
27	oar3_OAR〉	1.19E+08	-0.03062
27	oar3_OAR〉	1.19E+08	-0.01196
27	oar3_OAR〉	1.19E+08	0.002656
27	oar3_OAR〉	1.19E+08	NA
27	oar3_OAR〉	1.19E+08	-0.01575
27	oar3_OAR〉	1.19E+08	-0.0237
27	oar3_OAR〉	1.19E+08	0.003316
27	oar3_OAR〉	1.19E+08	-0.01423
27	oar3_OAR〉	1.19E+08	0.033341
27	oar3_OAR〉	1.19E+08	0.011164
27	oar3_OAR〉	1.19E+08	0.049699
27	oar3_OAR〉	1.19E+08	0.013868
27	oar3_OAR〉	1.19E+08	-0.03464
27	oar3_OAR〉	1.19E+08	-0.02151
27	oar3_OAR〉	1.19E+08	0.013773
27	oar3_OAR〉	1.19E+08	0.029304
27	oar3_OAR〉	1.19E+08	0.080185
27	oar3_OAR〉	1.19E+08	0.055946
27	oar3_OAR〉	1.19E+08	0.075382
27	oar3_OAR〉	1.19E+08	0.001512
27	oar3_OAR〉	1.19E+08	0.029289
27	oar3_OAR〉	1.19E+08	0.001512
27	oar3_OAR〉	1.19E+08	-0.01327
27	oar3_OAR〉	1.19E+08	0.016364
27	oar3_OAR〉	1.19E+08	0.002954
27	oar3_OAR〉	1.19E+08	-0.01653
27	oar3_OAR〉	1.19E+08	-0.01653
27	oar3_OAR〉	1.19E+08	0.071928
27	oar3_OAR〉	1.19E+08	-0.00491
27	oar3_OAR〉	1.19E+08	0.002954
27	oar3_OAR〉	1.19E+08	0.070703
27	oar3_OAR〉	1.19E+08	0.01418
27	oar3_OAR〉	1.19E+08	0.003593
27	oar3_OAR〉	1.19E+08	-0.04015
27	oar3_OAR〉	1.19E+08	0.073272
27	oar3_OAR〉	1.19E+08	-0.02377
27	oar3_OAR〉	1.19E+08	-0.02151
27	oar3_OAR〉	1.19E+08	-0.02377
27	oar3_OAR〉	1.19E+08	-0.0237
27	oar3_OAR〉	1.19E+08	0.053026
27	oar3_OAR〉	1.19E+08	-0.02619
27	oar3_OAR〉	1.19E+08	0.017726

27	oar3_OAR〉	1.19E+08	0.055946
27	oar3_OAR〉	1.19E+08	NA
27	oar3_OAR〉	1.19E+08	0.010572
27	oar3_OAR〉	1.19E+08	-0.01158
27	oar3_OAR〉	1.19E+08	0.03591
27	oar3_OAR〉	1.19E+08	0.015729
27	oar3_OAR〉	1.19E+08	0.043612
27	oar3_OAR〉	1.19E+08	0.066236
27	oar3_OAR〉	1.19E+08	0.003056
27	oar3_OAR〉	1.19E+08	0.003056
27	oar3_OAR〉	1.19E+08	0.011563
27	oar3_OAR〉	1.19E+08	-0.03582
27	oar3_OAR〉	1.19E+08	0.016973
27	oar3_OAR〉	1.19E+08	-0.01567
27	oar3_OAR〉	1.19E+08	0.003056
27	oar3_OAR〉	1.19E+08	-0.01735
27	oar3_OAR〉	1.19E+08	-0.02743
27	oar3_OAR〉	1.19E+08	0.022239
27	oar3_OAR〉	1.19E+08	0.022756
27	oar3_OAR〉	1.19E+08	0.036687
27	oar3_OAR〉	1.19E+08	-0.02629
27	oar3_OAR〉	1.19E+08	0.04764
27	oar3_OAR〉	1.19E+08	0.191335
27	oar3_OAR〉	1.19E+08	-0.00301
27	oar3_OAR〉	1.19E+08	0.025109
27	oar3_OAR〉	1.19E+08	-0.0293
27	oar3_OAR〉	1.19E+08	0.023411
27	oar3_OAR〉	1.19E+08	-0.01826
27	oar3_OAR〉	1.19E+08	0.092996
27	oar3_OAR〉	1.19E+08	0.061827
27	oar3_OAR〉	1.19E+08	-0.01826
27	oar3_OAR〉	1.19E+08	0.008214
27	oar3_OAR〉	1.19E+08	-0.01766
27	oar3_OAR〉	1.19E+08	0.035242
27	oar3_OAR〉	1.19E+08	0.011067
27	oar3_OAR〉	1.19E+08	-0.02184
27	oar3_OAR〉	1.19E+08	0.15776
27	oar3_OAR〉	1.19E+08	0.004483
27	oar3_OAR〉	1.19E+08	-0.01751
27	oar3_OAR〉	1.19E+08	-0.018
27	oar3_OAR〉	1.19E+08	0.007311
27	oar3_OAR〉	1.19E+08	-0.02832
27	oar3_OAR〉	1.19E+08	-0.02191
27	oar3_OAR〉	1.19E+08	0.097299
27	oar3_OAR〉	1.19E+08	-0.03184
27	oar3_OAR〉	1.19E+08	0.024156
27	oar3_OAR〉	1.19E+08	0.010393
27	oar3_OAR〉	1.2E+08	0.006191
27	oar3_OAR〉	1.2E+08	0.000164
27	oar3_OAR〉	1.2E+08	0.04895
27	oar3_OAR〉	1.2E+08	-0.027
27	oar3_OAR〉	1.2E+08	0.008867
27	oar3_OAR〉	1.2E+08	0.04895
27	oar3_OAR〉	1.2E+08	0.008867

27	oar3_OAR〉	1.2E+08	0.018013
27	oar3_OAR〉	1.2E+08	-0.00209
27	oar3_OAR〉	1.2E+08	-0.01439
27	oar3_OAR〉	1.2E+08	0.000889
27	oar3_OAR〉	1.2E+08	0.050459
27	oar3_OAR〉	1.2E+08	-0.02306
27	oar3_OAR〉	1.2E+08	0.004473
27	oar3_OAR〉	1.2E+08	0.053393
27	oar3_OAR〉	1.2E+08	-0.02054
27	oar3_OAR〉	1.2E+08	-0.00398
27	oar3_OAR〉	1.2E+08	-0.00941
27	oar3_OAR〉	1.2E+08	-0.02565
27	oar3_OAR〉	1.2E+08	0.003185
27	oar3_OAR〉	1.2E+08	0.031347
27	oar3_OAR〉	1.2E+08	0.093544
27	oar3_OAR〉	1.2E+08	0.031918
27	oar3_OAR〉	1.2E+08	-0.0249
27	oar3_OAR〉	1.2E+08	-0.0249
27	oar3_OAR〉	1.2E+08	-0.0249
27	oar3_OAR〉	1.2E+08	-0.03209
27	oar3_OAR〉	1.2E+08	-0.0249
27	oar3_OAR〉	1.2E+08	0.047303
27	oar3_OAR〉	1.2E+08	-0.03597
27	oar3_OAR〉	1.2E+08	0.040888
27	oar3_OAR〉	1.2E+08	-0.04284
27	oar3_OAR〉	1.2E+08	0.08616
27	oar3_OAR〉	1.2E+08	-0.01178
27	oar3_OAR〉	1.2E+08	0.029419
27	oar3_OAR〉	1.2E+08	0.037269
27	oar3_OAR〉	1.2E+08	-0.02797
27	oar3_OAR〉	1.2E+08	-0.00914
27	oar3_OAR〉	1.2E+08	-0.03175
27	oar3_OAR〉	1.2E+08	-0.01437
27	oar3_OAR〉	1.2E+08	-0.01963
27	oar3_OAR〉	1.2E+08	0.150248
27	oar3_OAR〉	1.2E+08	0.169235
27	oar3_OAR〉	1.2E+08	0.169235
27	oar3_OAR〉	1.2E+08	0.071027
27	oar3_OAR〉	1.2E+08	NA
27	oar3_OAR〉	1.2E+08	0.041359
27	oar3_OAR〉	1.2E+08	-0.00335
27	oar3_OAR〉	1.2E+08	-0.02699
27	oar3_OAR〉	1.2E+08	-0.00325
27	oar3_OAR〉	1.2E+08	-0.00288
27	oar3_OAR〉	1.2E+08	-0.00883
27	oar3_OAR〉	1.2E+08	-0.02119
27	oar3_OAR〉	1.2E+08	-0.01338
27	oar3_OAR〉	1.2E+08	-0.02348
27	oar3_OAR〉	1.2E+08	NA
27	oar3_OAR〉	1.2E+08	NA
27	oar3_OAR〉	1.2E+08	-0.00673
27	oar3_OAR〉	1.2E+08	0.073823
27	oar3_OAR〉	1.2E+08	0.004474
27	oar3_OAR〉	1.2E+08	-0.03153

27	oar3_OAR〉	1.2E+08	0.029811
27	oar3_OAR〉	1.2E+08	0.004696
27	oar3_OAR〉	1.2E+08	0.017152
27	oar3_OAR〉	1.2E+08	-0.00682
27	oar3_OAR〉	1.2E+08	-0.01418
27	oar3_OAR〉	1.2E+08	0.008511
27	oar3_OAR〉	1.2E+08	-0.00239
27	oar3_OAR〉	1.2E+08	-0.0172
27	oar3_OAR〉	1.2E+08	-0.01076
27	oar3_OAR〉	1.2E+08	0.047277
27	oar3_OAR〉	1.2E+08	0.079374
27	oar3_OAR〉	1.2E+08	0.060548
27	oar3_OAR〉	1.2E+08	-0.03139
27	oar3_OAR〉	1.2E+08	0.06824
27	oar3_OAR〉	1.2E+08	0.06824
27	oar3_OAR〉	1.2E+08	0.062129
27	oar3_OAR〉	1.2E+08	-0.02179
27	oar3_OAR〉	1.2E+08	0.006254
27	oar3_OAR〉	1.2E+08	0.026204
27	oar3_OAR〉	1.2E+08	0.024879
27	oar3_OAR〉	1.2E+08	0.025821
27	oar3_OAR〉	1.2E+08	0.045513
27	oar3_OAR〉	1.2E+08	0.05685
27	oar3_OAR〉	1.2E+08	-0.00375
27	oar3_OAR〉	1.2E+08	0.041107
27	oar3_OAR〉	1.2E+08	0.043604
27	oar3_OAR〉	1.2E+08	0.043604
27	oar3_OAR〉	1.2E+08	0.011946
27	oar3_OAR〉	1.2E+08	-0.00065
27	oar3_OAR〉	1.2E+08	-0.00266
27	oar3_OAR〉	1.2E+08	0.012464
27	oar3_OAR〉	1.2E+08	0.033987
27	oar3_OAR〉	1.2E+08	0.047311
27	oar3_OAR〉	1.2E+08	0.112585
27	oar3_OAR〉	1.2E+08	0.041221
27	oar3_OAR〉	1.2E+08	0.041221
27	oar3_OAR〉	1.2E+08	-0.01772
27	oar3_OAR〉	1.2E+08	0.026561
27	oar3_OAR〉	1.2E+08	0.070997
27	oar3_OAR〉	1.2E+08	-0.02136
27	oar3_OAR〉	1.2E+08	-0.02849
27	oar3_OAR〉	1.2E+08	0.09057
27	oar3_OAR〉	1.2E+08	0.017962
27	oar3_OAR〉	1.2E+08	-0.03498
27	oar3_OAR〉	1.2E+08	0.098993
27	oar3_OAR〉	1.2E+08	-0.01997
27	oar3_OAR〉	1.2E+08	-0.02899
27	oar3_OAR〉	1.2E+08	0.077147
27	oar3_OAR〉	1.2E+08	0.008177
27	oar3_OAR〉	1.2E+08	-0.00996
27	oar3_OAR〉	1.2E+08	0.008177
27	oar3_OAR〉	1.2E+08	0.008177
27	oar3_OAR〉	1.2E+08	0.077147
27	oar3_OAR〉	1.2E+08	0.028059

27	oar3_OAR〉	1.2E+08	-0.00728
27	oar3_OAR〉	1.2E+08	-0.02485
27	oar3_OAR〉	1.2E+08	0.039013
27	oar3_OAR〉	1.2E+08	-0.0236
27	oar3_OAR〉	1.2E+08	8.73E-05
27	oar3_OAR〉	1.2E+08	-0.02593
27	oar3_OAR〉	1.2E+08	-0.02031
27	oar3_OAR〉	1.2E+08	-0.02158
27	oar3_OAR〉	1.2E+08	0.018946
27	oar3_OAR〉	1.2E+08	-0.0236
27	oar3_OAR〉	1.2E+08	0.050639
27	oar3_OAR〉	1.2E+08	0.022647
27	oar3_OAR〉	1.2E+08	0.000239
27	oar3_OAR〉	1.2E+08	-0.00434
27	oar3_OAR〉	1.2E+08	-0.03035
27	oar3_OAR〉	1.2E+08	-0.01216
27	oar3_OAR〉	1.2E+08	0.02346
27	oar3_OAR〉	1.2E+08	0.022731
27	oar3_OAR〉	1.2E+08	-0.02701
27	oar3_OAR〉	1.2E+08	-0.03681
27	oar3_OAR〉	1.2E+08	-0.01262
27	oar3_OAR〉	1.2E+08	-0.03147
27	oar3_OAR〉	1.2E+08	-0.01262
27	oar3_OAR〉	1.2E+08	-0.01262
27	oar3_OAR〉	1.2E+08	-0.00032
27	oar3_OAR〉	1.2E+08	0.047877
27	oar3_OAR〉	1.2E+08	0.080405
27	oar3_OAR〉	1.2E+08	-0.01179
27	oar3_OAR〉	1.2E+08	-0.01191
27	oar3_OAR〉	1.2E+08	-0.00744
27	oar3_OAR〉	1.2E+08	-0.00022
27	oar3_OAR〉	1.2E+08	-0.01403
27	oar3_OAR〉	1.2E+08	0.044724
27	oar3_OAR〉	1.2E+08	0.044724
27	oar3_OAR〉	1.2E+08	-0.03062
27	oar3_OAR〉	1.2E+08	-0.03062
27	oar3_OAR〉	1.2E+08	-0.03101
27	oar3_OAR〉	1.2E+08	-0.01803
27	oar3_OAR〉	1.2E+08	-0.03101
27	oar3_OAR〉	1.2E+08	-0.02603
27	oar3_OAR〉	1.2E+08	0.019191
27	oar3_OAR〉	1.2E+08	0.217156
27	oar3_OAR〉	1.2E+08	-0.02603
27	oar3_OAR〉	1.2E+08	-0.02603
27	oar3_OAR〉	1.2E+08	0.070734
27	oar3_OAR〉	1.2E+08	-0.02603
27	oar3_OAR〉	1.2E+08	0.00976
27	oar3_OAR〉	1.2E+08	-0.02534
27	oar3_OAR〉	1.2E+08	0.060146
27	oar3_OAR〉	1.2E+08	0.053048
27	oar3_OAR〉	1.2E+08	0.008272
27	oar3_OAR〉	1.2E+08	0.031469
27	oar3_OAR〉	1.2E+08	0.055998
27	oar3_OAR〉	1.2E+08	0.145113

27	oar3_OAR〉	1.2E+08	-0.00932
27	oar3_OAR〉	1.2E+08	0.083051
27	oar3_OAR〉	1.2E+08	0.107591
27	oar3_OAR〉	1.2E+08	-0.02471
27	oar3_OAR〉	1.2E+08	0.083293
27	oar3_OAR〉	1.2E+08	0.035855
27	oar3_OAR〉	1.2E+08	0.014923
27	oar3_OAR〉	1.2E+08	-0.01718
27	oar3_OAR〉	1.2E+08	-0.02869
27	oar3_OAR〉	1.2E+08	-0.02606
27	oar3_OAR〉	1.2E+08	-0.02208
27	oar3_OAR〉	1.2E+08	-0.02606
27	oar3_OAR〉	1.2E+08	-0.02217
27	oar3_OAR〉	1.2E+08	-0.02576
27	oar3_OAR〉	1.2E+08	-0.02294
27	oar3_OAR〉	1.2E+08	0.110358
27	oar3_OAR〉	1.2E+08	-0.00965
27	oar3_OAR〉	1.2E+08	-0.02769
27	oar3_OAR〉	1.2E+08	-0.00462
27	oar3_OAR〉	1.2E+08	-0.02531
27	oar3_OAR〉	1.2E+08	-0.03202
27	oar3_OAR〉	1.2E+08	0.114816
27	oar3_OAR〉	1.2E+08	NA
27	oar3_OAR〉	1.2E+08	0.000889
27	oar3_OAR〉	1.2E+08	0.020939
27	oar3_OAR〉	1.2E+08	0.008867
27	oar3_OAR〉	1.2E+08	0.037433
27	oar3_OAR〉	1.2E+08	-0.02443
27	oar3_OAR〉	1.2E+08	-0.02443
27	oar3_OAR〉	1.2E+08	0.113517
27	oar3_OAR〉	1.2E+08	0.047767
27	oar3_OAR〉	1.2E+08	NA
27	oar3_OAR〉	1.2E+08	0.036
27	oar3_OAR〉	1.2E+08	0.027062
27	oar3_OAR〉	1.2E+08	-0.03268
27	oar3_OAR〉	1.2E+08	0.030815
27	oar3_OAR〉	1.2E+08	-0.01613
27	oar3_OAR〉	1.21E+08	0.030815
27	oar3_OAR〉	1.21E+08	0.019038
27	oar3_OAR〉	1.21E+08	0.079374
27	oar3_OAR〉	1.21E+08	0.028602
27	oar3_OAR〉	1.21E+08	-0.01673
27	oar3_OAR〉	1.21E+08	0.042432
27	oar3_OAR〉	1.21E+08	-0.00544
27	oar3_OAR〉	1.21E+08	-0.02492
27	oar3_OAR〉	1.21E+08	0.020827
27	oar3_OAR〉	1.21E+08	0.009639
27	oar3_OAR〉	1.21E+08	-0.00952
27	oar3_OAR〉	1.21E+08	0.01686
27	oar3_OAR〉	1.21E+08	0.01686
27	oar3_OAR〉	1.21E+08	-0.00756
27	oar3_OAR〉	1.21E+08	0.026817
27	oar3_OAR〉	1.21E+08	0.029806
27	oar3_OAR〉	1.21E+08	0.063845

27	oar3_OAR〉	1.21E+08	-0.02132
27	oar3_OAR〉	1.21E+08	-0.00109
27	oar3_OAR〉	1.21E+08	0.012034
27	oar3_OAR〉	1.21E+08	0.031225
27	oar3_OAR〉	1.21E+08	0.022027
27	oar3_OAR〉	1.21E+08	-0.03553
27	oar3_OAR〉	1.21E+08	-0.00317
27	oar3_OAR〉	1.21E+08	-0.02672
27	oar3_OAR〉	1.21E+08	-0.03073
27	oar3_OAR〉	1.21E+08	0.062943
27	oar3_OAR〉	1.21E+08	0.050366
27	oar3_OAR〉	1.21E+08	-0.01013
27	oar3_OAR〉	1.21E+08	-0.03152
27	oar3_OAR〉	1.21E+08	-0.0048
27	oar3_OAR〉	1.21E+08	-0.02926
27	oar3_OAR〉	1.21E+08	0.036531
27	oar3_OAR〉	1.21E+08	0.107488
27	oar3_OAR〉	1.21E+08	-0.00767
27	oar3_OAR〉	1.21E+08	-0.02053
27	oar3_OAR〉	1.21E+08	0.043755
27	oar3_OAR〉	1.21E+08	0.153362
27	oar3_OAR〉	1.21E+08	0.067198
27	oar3_OAR〉	1.21E+08	8.73E-05
27	oar3_OAR〉	1.21E+08	-0.02053
27	oar3_OAR〉	1.21E+08	0.153362
27	oar3_OAR〉	1.21E+08	0.120106
27	oar3_OAR〉	1.21E+08	0.063485
27	oar3_OAR〉	1.21E+08	-0.02517
27	oar3_OAR〉	1.21E+08	-0.01551
27	oar3_OAR〉	1.21E+08	0.010176
27	oar3_OAR〉	1.21E+08	0.179758
27	oar3_OAR〉	1.21E+08	-0.01624
27	oar3_OAR〉	1.21E+08	0.011067
27	oar3_OAR〉	1.21E+08	0.019257
27	oar3_OAR〉	1.21E+08	-0.04332
27	oar3_OAR〉	1.21E+08	0.023886
27	oar3_OAR〉	1.21E+08	0.037925
27	oar3_OAR〉	1.21E+08	-0.01069
27	oar3_OAR〉	1.21E+08	0.02053
27	oar3_OAR〉	1.21E+08	0.1162
27	oar3_OAR〉	1.21E+08	0.023774
27	oar3_OAR〉	1.21E+08	0.022242
27	oar3_OAR〉	1.21E+08	-0.02926
27	oar3_OAR〉	1.21E+08	0.177884
27	oar3_OAR〉	1.21E+08	-0.00966
27	oar3_OAR〉	1.21E+08	0.021695
27	oar3_OAR〉	1.21E+08	NA
27	oar3_OAR〉	1.21E+08	-0.01423
27	oar3_OAR〉	1.21E+08	-0.01423
27	oar3_OAR〉	1.21E+08	-0.01423
27	oar3_OAR〉	1.21E+08	-0.02681
27	oar3_OAR〉	1.21E+08	-0.03437
27	oar3_OAR〉	1.21E+08	-0.00575
27	oar3_OAR〉	1.21E+08	-0.00575

27	oar3_OAR〉	1.21E+08	0.014499
27	oar3_OAR〉	1.21E+08	-0.01661
27	oar3_OAR〉	1.21E+08	-0.01935
27	oar3_OAR〉	1.21E+08	0.071551
27	oar3_OAR〉	1.21E+08	0.044724
27	oar3_OAR〉	1.21E+08	-0.01822
27	oar3_OAR〉	1.21E+08	-0.01455
27	oar3_OAR〉	1.21E+08	0.007398
27	oar3_OAR〉	1.21E+08	0.032582
27	oar3_OAR〉	1.21E+08	0.007398
27	oar3_OAR〉	1.21E+08	0.032054
27	oar3_OAR〉	1.21E+08	0.032582
27	oar3_OAR〉	1.21E+08	0.002724
27	oar3_OAR〉	1.21E+08	-0.02818
27	oar3_OAR〉	1.21E+08	0.096318
27	oar3_OAR〉	1.21E+08	-0.02818
27	oar3_OAR〉	1.21E+08	0.078685
27	oar3_OAR〉	1.21E+08	0.078685
27	oar3_OAR〉	1.21E+08	-0.00188
27	oar3_OAR〉	1.21E+08	0.063259
27	oar3_OAR〉	1.21E+08	0.239879
27	oar3_OAR〉	1.21E+08	0.16344
27	oar3_OAR〉	1.21E+08	0.000889
27	oar3_OAR〉	1.21E+08	0.049715
27	oar3_OAR〉	1.21E+08	0.190574
27	oar3_OAR〉	1.21E+08	-0.01956
27	oar3_OAR〉	1.21E+08	0.049715
27	oar3_OAR〉	1.21E+08	0.191899
27	oar3_OAR〉	1.21E+08	0.025531
27	oar3_OAR〉	1.21E+08	0.000889
27	oar3_OAR〉	1.21E+08	0.148467
27	oar3_OAR〉	1.21E+08	-0.02926
27	oar3_OAR〉	1.21E+08	-0.03148
27	oar3_OAR〉	1.21E+08	0.197485
27	oar3_OAR〉	1.21E+08	0.100979
27	oar3_OAR〉	1.21E+08	-0.03226
27	oar3_OAR〉	1.21E+08	0.166761
27	oar3_OAR〉	1.21E+08	-0.01646
27	oar3_OAR〉	1.21E+08	-0.01194
27	oar3_OAR〉	1.21E+08	-0.00666
27	oar3_OAR〉	1.21E+08	0.032582
27	oar3_OAR〉	1.21E+08	0.000889
27	oar3_OAR〉	1.21E+08	0.026177
27	oar3_OAR〉	1.21E+08	0.071254
27	oar3_OAR〉	1.21E+08	0.00481
27	oar3_OAR〉	1.21E+08	0.031165
27	oar3_OAR〉	1.21E+08	0.004281
27	oar3_OAR〉	1.21E+08	0.020136
27	oar3_OAR〉	1.21E+08	0.020321
27	oar3_OAR〉	1.21E+08	-0.01389
27	oar3_OAR〉	1.21E+08	-0.0338
27	oar3_OAR〉	1.21E+08	0.028813
27	oar3_OAR〉	1.21E+08	0.028813
27	oar3_OAR〉	1.21E+08	-0.01396

27	oar3_OAR>	1.21E+08	-0.03431
27	oar3_OAR>	1.21E+08	0.00481
27	oar3_OAR>	1.21E+08	-0.01776
27	oar3_OAR>	1.21E+08	0.071605
27	oar3_OAR>	1.21E+08	0.176535
27	oar3_OAR>	1.21E+08	0.008805
27	oar3_OAR>	1.21E+08	0.040241
27	oar3_OAR>	1.21E+08	-0.03461
27	oar3_OAR>	1.21E+08	0.018013
27	oar3_OAR>	1.21E+08	-0.00751
27	oar3_OAR>	1.21E+08	0.058961
27	oar3_OAR>	1.21E+08	-0.01762
27	oar3_OAR>	1.21E+08	0.08659
27	oar3_OAR>	1.21E+08	-0.00751
27	oar3_OAR>	1.21E+08	0.0523
27	oar3_OAR>	1.21E+08	0.000161
27	oar3_OAR>	1.21E+08	-0.00751
27	oar3_OAR>	1.21E+08	0.066795
27	oar3_OAR>	1.21E+08	0.03342
27	oar3_OAR>	1.21E+08	0.009985
27	oar3_OAR>	1.21E+08	0.054317
27	oar3_OAR>	1.21E+08	0.012941
27	oar3_OAR>	1.21E+08	0.064529
27	oar3_OAR>	1.21E+08	0.064529
27	oar3_OAR>	1.21E+08	-0.00954
27	oar3_OAR>	1.21E+08	-0.02902
27	oar3_OAR>	1.21E+08	-0.02002
27	oar3_OAR>	1.21E+08	-0.01934
27	oar3_OAR>	1.21E+08	-0.01934
27	oar3_OAR>	1.21E+08	-0.03267
27	oar3_OAR>	1.21E+08	-0.01843
27	oar3_OAR>	1.21E+08	0.004928
27	oar3_OAR>	1.21E+08	-0.03148
27	oar3_OAR>	1.21E+08	-0.03059
27	oar3_OAR>	1.21E+08	0.007823
27	oar3_OAR>	1.21E+08	-0.02863
27	oar3_OAR>	1.21E+08	-0.0222
27	oar3_OAR>	1.21E+08	-0.02863
27	oar3_OAR>	1.21E+08	0.032012
27	oar3_OAR>	1.21E+08	0.051493
27	oar3_OAR>	1.21E+08	-0.02645
27	oar3_OAR>	1.21E+08	-0.01536
27	oar3_OAR>	1.21E+08	0.117106
27	oar3_OAR>	1.21E+08	-0.00606
27	oar3_OAR>	1.21E+08	-0.02292
27	oar3_OAR>	1.21E+08	0.040808
27	oar3_OAR>	1.21E+08	-0.01476
27	oar3_OAR>	1.21E+08	0.118017
27	oar3_OAR>	1.21E+08	0.097552
27	oar3_OAR>	1.21E+08	0.062775
27	oar3_OAR>	1.21E+08	0.102821
27	oar3_OAR>	1.21E+08	0.058103
27	oar3_OAR>	1.21E+08	-0.00882
27	oar3_OAR>	1.21E+08	-0.01607

27	oar3_OAR›	1.22E+08	-0.00405
27	oar3_OAR›	1.22E+08	-0.01886
27	oar3_OAR›	1.22E+08	-0.02827
27	oar3_OAR›	1.22E+08	0.01677
27	oar3_OAR›	1.22E+08	0.077185
27	oar3_OAR›	1.22E+08	-0.02765
27	oar3_OAR›	1.22E+08	-0.02822
27	oar3_OAR›	1.22E+08	-0.01978
27	oar3_OAR›	1.22E+08	-0.02633
27	oar3_OAR›	1.22E+08	-0.00898
27	oar3_OAR›	1.22E+08	-0.03554
27	oar3_OAR›	1.22E+08	-0.03617
27	oar3_OAR›	1.22E+08	-0.02636
27	oar3_OAR›	1.22E+08	-0.02127
27	oar3_OAR›	1.22E+08	-0.02236
27	oar3_OAR›	1.22E+08	0.018893
27	oar3_OAR›	1.22E+08	0.039845
27	oar3_OAR›	1.22E+08	-0.0249
27	oar3_OAR›	1.22E+08	-0.02463
27	oar3_OAR›	1.22E+08	0.055581
27	oar3_OAR›	1.22E+08	0.024907
27	oar3_OAR›	1.22E+08	-0.00457
27	oar3_OAR›	1.22E+08	-0.02616
27	oar3_OAR›	1.22E+08	0.007039
27	oar3_OAR›	1.22E+08	0.099057
27	oar3_OAR›	1.22E+08	0.006708
27	oar3_OAR›	1.22E+08	0.146872
27	oar3_OAR›	1.22E+08	-0.01251
27	oar3_OAR›	1.22E+08	0.053465
27	oar3_OAR›	1.22E+08	0.053057
27	oar3_OAR›	1.22E+08	-0.01771
27	oar3_OAR›	1.22E+08	0.011978
27	oar3_OAR›	1.22E+08	0.018044
27	oar3_OAR›	1.22E+08	-0.00989
27	oar3_OAR›	1.22E+08	0.000374
27	oar3_OAR›	1.22E+08	-0.02737
27	oar3_OAR›	1.22E+08	-0.02719
27	oar3_OAR›	1.22E+08	0.042344
27	oar3_OAR›	1.22E+08	-0.01519
27	oar3_OAR›	1.22E+08	0.039098
27	oar3_OAR›	1.22E+08	-0.00993
27	oar3_OAR›	1.22E+08	-0.00993
27	oar3_OAR›	1.22E+08	-0.0287
27	oar3_OAR›	1.22E+08	-0.03192
27	oar3_OAR›	1.22E+08	-0.00188
27	oar3_OAR›	1.22E+08	0.095721
27	oar3_OAR›	1.22E+08	-0.02996
27	oar3_OAR›	1.22E+08	-0.00716
27	oar3_OAR›	1.22E+08	0.01778
27	oar3_OAR›	1.22E+08	0.164267
27	oar3_OAR›	1.22E+08	0.003107
27	oar3_OAR›	1.22E+08	-0.01354
27	oar3_OAR›	1.22E+08	-0.00157
27	oar3_OAR›	1.22E+08	0.042857

27	oar3_OAR〉	1.22E+08	0.034687
27	oar3_OAR〉	1.22E+08	-0.02262
27	oar3_OAR〉	1.22E+08	0.041681
27	oar3_OAR〉	1.22E+08	0.000753
27	oar3_OAR〉	1.22E+08	0.009316
27	oar3_OAR〉	1.22E+08	0.071674
27	oar3_OAR〉	1.22E+08	-0.03583
27	oar3_OAR〉	1.22E+08	-0.01291
27	oar3_OAR〉	1.22E+08	-0.01527
27	oar3_OAR〉	1.22E+08	0.099061
27	oar3_OAR〉	1.22E+08	-0.006
27	oar3_OAR〉	1.22E+08	-0.0205
27	oar3_OAR〉	1.22E+08	0.111038
27	oar3_OAR〉	1.22E+08	0.021577
27	oar3_OAR〉	1.22E+08	-0.02292
27	oar3_OAR〉	1.22E+08	-0.02988
27	oar3_OAR〉	1.22E+08	0.016406
27	oar3_OAR〉	1.22E+08	-0.02786
27	oar3_OAR〉	1.22E+08	0.971077
27	oar3_OAR〉	1.22E+08	0.038901
27	oar3_OAR〉	1.22E+08	0.030905
27	oar3_OAR〉	1.22E+08	0.003055
27	oar3_OAR〉	1.22E+08	0.044737
27	oar3_OAR〉	1.22E+08	0.044737
27	oar3_OAR〉	1.22E+08	NA
27	oar3_OAR〉	1.22E+08	-0.02242
27	oar3_OAR〉	1.22E+08	0.081984
27	oar3_OAR〉	1.22E+08	0.026384
27	oar3_OAR〉	1.22E+08	0.064242
27	oar3_OAR〉	1.22E+08	0.000889
27	oar3_OAR〉	1.22E+08	-0.00287
27	oar3_OAR〉	1.22E+08	0.017154
27	oar3_OAR〉	1.22E+08	0.006682
27	oar3_OAR〉	1.22E+08	0.058709
27	oar3_OAR〉	1.22E+08	0.043729
27	oar3_OAR〉	1.22E+08	-0.03193
27	oar3_OAR〉	1.22E+08	-0.00463
27	oar3_OAR〉	1.22E+08	0.141497
27	oar3_OAR〉	1.22E+08	0.028743
27	oar3_OAR〉	1.22E+08	0.081549
27	oar3_OAR〉	1.22E+08	0.061457
27	oar3_OAR〉	1.22E+08	0.030548
27	oar3_OAR〉	1.22E+08	-0.02785
27	oar3_OAR〉	1.22E+08	-0.02338
27	oar3_OAR〉	1.22E+08	-0.01673
27	oar3_OAR〉	1.22E+08	0.08545
27	oar3_OAR〉	1.22E+08	0.054341
27	oar3_OAR〉	1.22E+08	-0.03147
27	oar3_OAR〉	1.22E+08	-0.01841
27	oar3_OAR〉	1.22E+08	0.048474
27	oar3_OAR〉	1.22E+08	0.048474
27	oar3_OAR〉	1.22E+08	0.033898
27	oar3_OAR〉	1.22E+08	0.037417
27	oar3_OAR〉	1.22E+08	-0.00563

27	oar3_OAR〉	1.22E+08	0.050456
27	oar3_OAR〉	1.22E+08	0.037417
27	oar3_OAR〉	1.22E+08	0.066389
27	oar3_OAR〉	1.22E+08	-0.03273
27	oar3_OAR〉	1.22E+08	-0.02218
27	oar3_OAR〉	1.22E+08	0.045453
27	oar3_OAR〉	1.22E+08	-0.00208
27	oar3_OAR〉	1.22E+08	-0.00605
27	oar3_OAR〉	1.22E+08	-0.01107
27	oar3_OAR〉	1.22E+08	-0.01737
27	oar3_OAR〉	1.22E+08	-0.00386
27	oar3_OAR〉	1.22E+08	0.047001
27	oar3_OAR〉	1.22E+08	0.031994
27	oar3_OAR〉	1.22E+08	-0.04195
27	oar3_OAR〉	1.22E+08	0.008082
27	oar3_OAR〉	1.22E+08	-0.04236
27	oar3_OAR〉	1.22E+08	-0.00935
27	oar3_OAR〉	1.22E+08	0.031514
27	oar3_OAR〉	1.22E+08	0.031514
27	oar3_OAR〉	1.22E+08	-0.01717
27	oar3_OAR〉	1.22E+08	-0.00935
27	oar3_OAR〉	1.22E+08	-0.02168
27	oar3_OAR〉	1.22E+08	0.000889
27	oar3_OAR〉	1.22E+08	-0.01623
27	oar3_OAR〉	1.22E+08	-0.02438
27	oar3_OAR〉	1.22E+08	-0.02209
27	oar3_OAR〉	1.22E+08	0.093421
27	oar3_OAR〉	1.22E+08	0.045876
27	oar3_OAR〉	1.22E+08	0.228457
27	oar3_OAR〉	1.22E+08	-0.01024
27	oar3_OAR〉	1.22E+08	-0.00423
27	oar3_OAR〉	1.22E+08	-0.00083
27	oar3_OAR〉	1.22E+08	0.051729
27	oar3_OAR〉	1.22E+08	-0.00395
27	oar3_OAR〉	1.22E+08	-0.02773
27	oar3_OAR〉	1.22E+08	0.007056
27	oar3_OAR〉	1.22E+08	-0.03183
27	oar3_OAR〉	1.22E+08	-0.03096
27	oar3_OAR〉	1.22E+08	-0.01072
27	oar3_OAR〉	1.22E+08	0.040066
27	oar3_OAR〉	1.22E+08	0.030049
27	oar3_OAR〉	1.22E+08	-0.02416
27	oar3_OAR〉	1.22E+08	-0.02834
27	oar3_OAR〉	1.22E+08	-0.02996
27	oar3_OAR〉	1.22E+08	-0.02662
27	oar3_OAR〉	1.22E+08	-0.00423
27	oar3_OAR〉	1.22E+08	0.126647
27	oar3_OAR〉	1.22E+08	0.001737
27	oar3_OAR〉	1.22E+08	0.038773
27	oar3_OAR〉	1.22E+08	-0.00942
27	oar3_OAR〉	1.22E+08	-0.00113
27	oar3_OAR〉	1.22E+08	0.009322
27	oar3_OAR〉	1.22E+08	0.038838
27	oar3_OAR〉	1.22E+08	0.002764

27	oar3_OAR〉	1.22E+08	-0.00928
27	oar3_OAR〉	1.22E+08	-0.02452
27	oar3_OAR〉	1.22E+08	0.002954
27	oar3_OAR〉	1.22E+08	0.014122
27	oar3_OAR〉	1.22E+08	0.006417
27	oar3_OAR〉	1.22E+08	0.027521
27	oar3_OAR〉	1.22E+08	0.006308
27	oar3_OAR〉	1.22E+08	0.014282
27	oar3_OAR〉	1.22E+08	-0.04075
27	oar3_OAR〉	1.22E+08	-0.04075
27	oar3_OAR〉	1.22E+08	-0.0156
27	oar3_OAR〉	1.22E+08	0.054267
27	oar3_OAR〉	1.22E+08	-0.02841
27	oar3_OAR〉	1.22E+08	0.00258
27	oar3_OAR〉	1.22E+08	0.053904
27	oar3_OAR〉	1.22E+08	-0.03161
27	oar3_OAR〉	1.22E+08	-0.00181
27	oar3_OAR〉	1.22E+08	-0.0309
27	oar3_OAR〉	1.22E+08	0.036197
27	oar3_OAR〉	1.22E+08	-0.01069
27	oar3_OAR〉	1.22E+08	0.003262
27	oar3_OAR〉	1.22E+08	-0.01279
27	oar3_OAR〉	1.22E+08	0.079446
27	oar3_OAR〉	1.22E+08	0.111926
27	oar3_OAR〉	1.22E+08	0.00481
27	oar3_OAR〉	1.22E+08	0.107959
27	oar3_OAR〉	1.22E+08	0.002651
27	oar3_OAR〉	1.22E+08	-0.02697
27	oar3_OAR〉	1.22E+08	0.044159
27	oar3_OAR〉	1.22E+08	0.124368
27	oar3_OAR〉	1.22E+08	0.124368
27	oar3_OAR〉	1.22E+08	-0.0385
27	oar3_OAR〉	1.22E+08	0.071007
27	oar3_OAR〉	1.22E+08	0.114899
27	oar3_OAR〉	1.22E+08	-0.02512
27	oar3_OAR〉	1.22E+08	-0.01762
27	oar3_OAR〉	1.22E+08	-0.02529
27	oar3_OAR〉	1.22E+08	-0.02529
27	oar3_OAR〉	1.22E+08	-0.01288
27	oar3_OAR〉	1.22E+08	-0.02475
27	oar3_OAR〉	1.22E+08	-0.01433
27	oar3_OAR〉	1.22E+08	0.028919
27	oar3_OAR〉	1.22E+08	0.099096
27	oar3_OAR〉	1.22E+08	-0.01211
27	oar3_OAR〉	1.22E+08	0.084035
27	oar3_OAR〉	1.22E+08	0.010181
27	oar3_OAR〉	1.22E+08	0.052599
27	oar3_OAR〉	1.22E+08	-0.03695
27	oar3_OAR〉	1.22E+08	-0.01773
27	oar3_OAR〉	1.22E+08	-0.03695
27	oar3_OAR〉	1.22E+08	-0.00438
27	oar3_OAR〉	1.22E+08	-0.00822
27	oar3_OAR〉	1.22E+08	0.002868
27	oar3_OAR〉	1.22E+08	-0.02405

27	oar3_OAR›	1.22E+08	-0.01538
27	oar3_OAR›	1.22E+08	-0.00756
27	oar3_OAR›	1.22E+08	-0.01538
27	oar3_OAR›	1.22E+08	0.048256
27	oar3_OAR›	1.22E+08	-0.03335
27	oar3_OAR›	1.22E+08	-0.03335
27	oar3_OAR›	1.22E+08	-0.03335
27	oar3_OAR›	1.22E+08	0.01645
27	oar3_OAR›	1.23E+08	0.002868
27	oar3_OAR›	1.23E+08	-0.01735
27	oar3_OAR›	1.23E+08	0.064315
27	oar3_OAR›	1.23E+08	-0.03175
27	oar3_OAR›	1.23E+08	0.07533
27	oar3_OAR›	1.23E+08	0.106659
27	oar3_OAR›	1.23E+08	0.196287
27	oar3_OAR›	1.23E+08	0.153419
27	oar3_OAR›	1.23E+08	-0.0012
27	oar3_OAR›	1.23E+08	-0.0012
27	oar3_OAR›	1.23E+08	0.007039
27	oar3_OAR›	1.23E+08	0.002527
27	oar3_OAR›	1.23E+08	0.002527
27	oar3_OAR›	1.23E+08	-0.01291
27	oar3_OAR›	1.23E+08	-0.0234
27	oar3_OAR›	1.23E+08	-0.0234
27	oar3_OAR›	1.23E+08	-0.01291
27	oar3_OAR›	1.23E+08	-0.02945
27	oar3_OAR›	1.23E+08	-0.00864
27	oar3_OAR›	1.23E+08	-0.01095
27	oar3_OAR›	1.23E+08	-0.0178
27	oar3_OAR›	1.23E+08	-0.01473
27	oar3_OAR›	1.23E+08	-0.01931
27	oar3_OAR›	1.23E+08	0.009572
27	oar3_OAR›	1.23E+08	0.013077
27	oar3_OAR›	1.23E+08	0.057602
27	oar3_OAR›	1.23E+08	0.082377
27	oar3_OAR›	1.23E+08	-0.01974
27	oar3_OAR›	1.23E+08	-0.02597
27	oar3_OAR›	1.23E+08	-0.02757
27	oar3_OAR›	1.23E+08	-0.00953
27	oar3_OAR›	1.23E+08	0.000321
27	oar3_OAR›	1.23E+08	-0.01161
27	oar3_OAR›	1.23E+08	-0.00177
27	oar3_OAR›	1.23E+08	-0.00177
27	oar3_OAR›	1.23E+08	-0.02273
27	oar3_OAR›	1.23E+08	-0.0264
27	oar3_OAR›	1.23E+08	-0.01019
27	oar3_OAR›	1.23E+08	-0.02186
27	oar3_OAR›	1.23E+08	-0.01772
27	oar3_OAR›	1.23E+08	0.078504
27	oar3_OAR›	1.23E+08	-0.02186
27	oar3_OAR›	1.23E+08	-0.01167
27	oar3_OAR›	1.23E+08	-0.03416
27	oar3_OAR›	1.23E+08	-0.02222
27	oar3_OAR›	1.23E+08	-0.01096

27	oar3_OAR〉	1.23E+08	0.048359
27	oar3_OAR〉	1.23E+08	-0.00354
27	oar3_OAR〉	1.23E+08	-0.01089
27	oar3_OAR〉	1.23E+08	-0.02784
27	oar3_OAR〉	1.23E+08	-0.01031
27	oar3_OAR〉	1.23E+08	-0.03116
27	oar3_OAR〉	1.23E+08	-0.00577
27	oar3_OAR〉	1.23E+08	-0.03662
27	oar3_OAR〉	1.23E+08	-0.02489
27	oar3_OAR〉	1.23E+08	-0.02565
27	oar3_OAR〉	1.23E+08	-0.03237
27	oar3_OAR〉	1.23E+08	-0.00577
27	oar3_OAR〉	1.23E+08	-0.00319
27	oar3_OAR〉	1.23E+08	-0.02489
27	oar3_OAR〉	1.23E+08	-0.02348
27	oar3_OAR〉	1.23E+08	0.00481
27	oar3_OAR〉	1.23E+08	-0.02967
27	oar3_OAR〉	1.23E+08	-0.02473
27	oar3_OAR〉	1.23E+08	0.064036
27	oar3_OAR〉	1.23E+08	NA
27	oar3_OAR〉	1.23E+08	-0.02967
27	oar3_OAR〉	1.23E+08	-0.02049
27	oar3_OAR〉	1.23E+08	0.03489
27	oar3_OAR〉	1.23E+08	0.050346
27	oar3_OAR〉	1.23E+08	-0.02049
27	oar3_OAR〉	1.23E+08	-0.01834
27	oar3_OAR〉	1.23E+08	-0.01834
27	oar3_OAR〉	1.23E+08	0.042508
27	oar3_OAR〉	1.23E+08	6.15E-05
27	oar3_OAR〉	1.23E+08	-0.02748
27	oar3_OAR〉	1.23E+08	0.006015
27	oar3_OAR〉	1.23E+08	-0.02744
27	oar3_OAR〉	1.23E+08	-0.00322
27	oar3_OAR〉	1.23E+08	-0.00685
27	oar3_OAR〉	1.23E+08	0.03428
27	oar3_OAR〉	1.23E+08	-0.01494
27	oar3_OAR〉	1.23E+08	0.081223
27	oar3_OAR〉	1.23E+08	0.005255
27	oar3_OAR〉	1.23E+08	-0.00488
27	oar3_OAR〉	1.23E+08	-0.02716
27	oar3_OAR〉	1.23E+08	-0.02838
27	oar3_OAR〉	1.23E+08	0.001336
27	oar3_OAR〉	1.23E+08	-0.00776
27	oar3_OAR〉	1.23E+08	0.063134
27	oar3_OAR〉	1.23E+08	0.005255
27	oar3_OAR〉	1.23E+08	0.054029
27	oar3_OAR〉	1.23E+08	0.024934
27	oar3_OAR〉	1.23E+08	NA
27	oar3_OAR〉	1.23E+08	0.102259
27	oar3_OAR〉	1.23E+08	-0.01958
27	oar3_OAR〉	1.23E+08	NA
27	oar3_OAR〉	1.23E+08	0.05606
27	oar3_OAR〉	1.23E+08	-0.00539
27	oar3_OAR〉	1.23E+08	NA

27	oar3_OAR>	1.23E+08	-0.00539
27	oar3_OAR>	1.23E+08	0.036843
27	oar3_OAR>	1.23E+08	-0.00154
27	oar3_OAR>	1.23E+08	-0.01934
27	oar3_OAR>	1.23E+08	0.071536
27	oar3_OAR>	1.23E+08	0.096132
27	oar3_OAR>	1.23E+08	-0.03461
27	oar3_OAR>	1.23E+08	-0.02475
27	oar3_OAR>	1.23E+08	0.064242
27	oar3_OAR>	1.23E+08	-0.00791
27	oar3_OAR>	1.23E+08	-0.01538
27	oar3_OAR>	1.23E+08	-0.0262
27	oar3_OAR>	1.23E+08	0.014259
27	oar3_OAR>	1.23E+08	-0.01794
27	oar3_OAR>	1.23E+08	-0.03806
27	oar3_OAR>	1.23E+08	-0.00597
27	oar3_OAR>	1.23E+08	0.080115
27	oar3_OAR>	1.23E+08	0.035747
27	oar3_OAR>	1.23E+08	0.158144
27	oar3_OAR>	1.23E+08	0.010014
27	oar3_OAR>	1.23E+08	0.035747
27	oar3_OAR>	1.23E+08	-0.00895
27	oar3_OAR>	1.23E+08	-0.02571
27	oar3_OAR>	1.23E+08	0.02318
27	oar3_OAR>	1.23E+08	-0.02736
27	oar3_OAR>	1.23E+08	-0.00351
27	oar3_OAR>	1.23E+08	0.023487
27	oar3_OAR>	1.23E+08	-0.00148
27	oar3_OAR>	1.23E+08	-0.00255
27	oar3_OAR>	1.23E+08	-0.00238
27	oar3_OAR>	1.23E+08	0.007973
27	oar3_OAR>	1.23E+08	0.044724
27	oar3_OAR>	1.23E+08	0.048248
27	oar3_OAR>	1.23E+08	-0.03471
27	oar3_OAR>	1.23E+08	-0.02981
27	oar3_OAR>	1.23E+08	-0.02191
27	oar3_OAR>	1.23E+08	0.00998
27	oar3_OAR>	1.23E+08	0.072392
27	oar3_OAR>	1.23E+08	-0.01655
27	oar3_OAR>	1.23E+08	-0.00976
27	oar3_OAR>	1.23E+08	-0.02899
27	oar3_OAR>	1.23E+08	-0.03493
27	oar3_OAR>	1.23E+08	-0.03493
27	oar3_OAR>	1.23E+08	-0.01439
27	oar3_OAR>	1.23E+08	-0.00516
27	oar3_OAR>	1.23E+08	0.00872
27	oar3_OAR>	1.23E+08	-0.02419
27	oar3_OAR>	1.23E+08	-0.02082
27	oar3_OAR>	1.23E+08	-0.02419
27	oar3_OAR>	1.23E+08	-0.02218
27	oar3_OAR>	1.23E+08	-0.02171
27	oar3_OAR>	1.23E+08	0.089974
27	oar3_OAR>	1.23E+08	-0.01525
27	oar3_OAR>	1.23E+08	0.021769

27	oar3_OAR〉	1.23E+08	0.026326
27	oar3_OAR〉	1.23E+08	0.036869
27	oar3_OAR〉	1.23E+08	0.0317
27	oar3_OAR〉	1.23E+08	0.028639
27	oar3_OAR〉	1.23E+08	0.060294
27	oar3_OAR〉	1.23E+08	0.039098
27	oar3_OAR〉	1.23E+08	0.039098
27	oar3_OAR〉	1.23E+08	0.039098
27	oar3_OAR〉	1.23E+08	0.025317
27	oar3_OAR〉	1.23E+08	0.039098
27	oar3_OAR〉	1.23E+08	-0.02782
27	oar3_OAR〉	1.23E+08	-0.00642
27	oar3_OAR〉	1.23E+08	0.030729
27	oar3_OAR〉	1.23E+08	0.063524
27	oar3_OAR〉	1.23E+08	0.013235
27	oar3_OAR〉	1.23E+08	0.066868
27	oar3_OAR〉	1.23E+08	0.037675
27	oar3_OAR〉	1.23E+08	-0.02572
27	oar3_OAR〉	1.23E+08	-0.01718
27	oar3_OAR〉	1.23E+08	-0.00622
27	oar3_OAR〉	1.23E+08	-0.02414
27	oar3_OAR〉	1.23E+08	-0.00668
27	oar3_OAR〉	1.23E+08	0.072924
27	oar3_OAR〉	1.23E+08	0.067092
27	oar3_OAR〉	1.23E+08	0.067092
27	oar3_OAR〉	1.23E+08	-0.0353
27	oar3_OAR〉	1.23E+08	0.139462
27	oar3_OAR〉	1.23E+08	-0.0353
27	oar3_OAR〉	1.23E+08	0.005125
27	oar3_OAR〉	1.23E+08	0.270136
27	oar3_OAR〉	1.23E+08	0.100378
27	oar3_OAR〉	1.23E+08	0.107914
27	oar3_OAR〉	1.23E+08	0.08114
27	oar3_OAR〉	1.23E+08	-0.0353
27	oar3_OAR〉	1.23E+08	0.005125
27	oar3_OAR〉	1.23E+08	0.188224
27	oar3_OAR〉	1.23E+08	0.152897
27	oar3_OAR〉	1.23E+08	0.082905
27	oar3_OAR〉	1.23E+08	0.081698
27	oar3_OAR〉	1.23E+08	-0.03392
27	oar3_OAR〉	1.23E+08	0.013887
27	oar3_OAR〉	1.23E+08	-0.02714
27	oar3_OAR〉	1.23E+08	-0.02604
27	oar3_OAR〉	1.23E+08	-0.00195
27	oar3_OAR〉	1.23E+08	-0.03434
27	oar3_OAR〉	1.23E+08	-0.02996
27	oar3_OAR〉	1.23E+08	-0.01859
27	oar3_OAR〉	1.23E+08	-0.02627
27	oar3_OAR〉	1.23E+08	0.005613
27	oar3_OAR〉	1.23E+08	-0.02116
27	oar3_OAR〉	1.23E+08	-0.0154
27	oar3_OAR〉	1.24E+08	0.005092
27	oar3_OAR〉	1.24E+08	0.016674
27	oar3_OAR〉	1.24E+08	0.02053

27	oar3_OAR›	1.24E+08	-0.00092
27	oar3_OAR›	1.24E+08	0.012943
27	oar3_OAR›	1.24E+08	0.05236
27	oar3_OAR›	1.24E+08	-0.03195
27	oar3_OAR›	1.24E+08	0.026712
27	oar3_OAR›	1.24E+08	-0.01199
27	oar3_OAR›	1.24E+08	0.02864
27	oar3_OAR›	1.24E+08	0.030701
27	oar3_OAR›	1.24E+08	0.088942
27	oar3_OAR›	1.24E+08	0.088942
27	oar3_OAR›	1.24E+08	-0.02025
27	oar3_OAR›	1.24E+08	0.00395
27	oar3_OAR›	1.24E+08	0.060033
27	oar3_OAR›	1.24E+08	-0.02651
27	oar3_OAR›	1.24E+08	-0.03662
27	oar3_OAR›	1.24E+08	-0.03662
27	oar3_OAR›	1.24E+08	-0.03662
27	oar3_OAR›	1.24E+08	-0.03662
27	oar3_OAR›	1.24E+08	0.153726
27	oar3_OAR›	1.24E+08	0.055484
27	oar3_OAR›	1.24E+08	-0.01731
27	oar3_OAR›	1.24E+08	-0.01665
27	oar3_OAR›	1.24E+08	0.064704
27	oar3_OAR›	1.24E+08	0.057012
27	oar3_OAR›	1.24E+08	0.145123
27	oar3_OAR›	1.24E+08	0.021865
27	oar3_OAR›	1.24E+08	0.005107
27	oar3_OAR›	1.24E+08	-0.01731
27	oar3_OAR›	1.24E+08	-0.03147
27	oar3_OAR›	1.24E+08	0.007039
27	oar3_OAR›	1.24E+08	-0.03178
27	oar3_OAR›	1.24E+08	-0.01608
27	oar3_OAR›	1.24E+08	0.00222
27	oar3_OAR›	1.24E+08	0.00721
27	oar3_OAR›	1.24E+08	0.007354
27	oar3_OAR›	1.24E+08	0.02205
27	oar3_OAR›	1.24E+08	0.028803
27	oar3_OAR›	1.24E+08	0.014872
27	oar3_OAR›	1.24E+08	0.008214
27	oar3_OAR›	1.24E+08	0.035778
27	oar3_OAR›	1.24E+08	0.031225
27	oar3_OAR›	1.24E+08	-0.00534
27	oar3_OAR›	1.24E+08	0.10621
27	oar3_OAR›	1.24E+08	0.02539
27	oar3_OAR›	1.24E+08	0.04903
27	oar3_OAR›	1.24E+08	0.04903
27	oar3_OAR›	1.24E+08	0.069173
27	oar3_OAR›	1.24E+08	0.031225
27	oar3_OAR›	1.24E+08	-0.02879
27	oar3_OAR›	1.24E+08	-0.02879
27	oar3_OAR›	1.24E+08	-0.02879
27	oar3_OAR›	1.24E+08	0.03668
27	oar3_OAR›	1.24E+08	0.03668
27	oar3_OAR›	1.24E+08	-0.0234

27	oar3_OAR›	1.24E+08	-0.00645
27	oar3_OAR›	1.24E+08	0.003762
27	oar3_OAR›	1.24E+08	-0.00459
27	oar3_OAR›	1.24E+08	0.037446
27	oar3_OAR›	1.24E+08	-0.01332
27	oar3_OAR›	1.24E+08	0.130967
27	oar3_OAR›	1.24E+08	-0.00238
27	oar3_OAR›	1.24E+08	-0.00459
27	oar3_OAR›	1.24E+08	-0.01789
27	oar3_OAR›	1.24E+08	0.068057
27	oar3_OAR›	1.24E+08	0.083909
27	oar3_OAR›	1.24E+08	-0.01685
27	oar3_OAR›	1.24E+08	-0.00205
27	oar3_OAR›	1.24E+08	0.068057
27	oar3_OAR›	1.24E+08	-0.03463
27	oar3_OAR›	1.24E+08	-0.02132
27	oar3_OAR›	1.24E+08	-0.00676
27	oar3_OAR›	1.24E+08	0.103845
27	oar3_OAR›	1.24E+08	0.004105
27	oar3_OAR›	1.24E+08	0.13501
27	oar3_OAR›	1.24E+08	-0.03723
27	oar3_OAR›	1.24E+08	0.044737
27	oar3_OAR›	1.24E+08	0.090502
27	oar3_OAR›	1.24E+08	0.017028
27	oar3_OAR›	1.24E+08	0.077783
27	oar3_OAR›	1.24E+08	-0.02977
27	oar3_OAR›	1.24E+08	-0.0334
27	oar3_OAR›	1.24E+08	0.041446
27	oar3_OAR›	1.24E+08	0.104444
27	oar3_OAR›	1.24E+08	0.120677
27	oar3_OAR›	1.24E+08	-0.0334
27	oar3_OAR›	1.24E+08	0.020149
27	oar3_OAR›	1.24E+08	-0.03237
27	oar3_OAR›	1.24E+08	0.029697
27	oar3_OAR›	1.24E+08	0.078631
27	oar3_OAR›	1.24E+08	-0.0194
27	oar3_OAR›	1.24E+08	0.036824
27	oar3_OAR›	1.24E+08	0.005553
27	oar3_OAR›	1.24E+08	0.000374
27	oar3_OAR›	1.24E+08	0.029668
27	oar3_OAR›	1.24E+08	-0.02341
27	oar3_OAR›	1.24E+08	########
27	oar3_OAR›	1.24E+08	0.099362
27	oar3_OAR›	1.24E+08	0.062055
27	oar3_OAR›	1.24E+08	0.093606
27	oar3_OAR›	1.24E+08	0.099362
27	oar3_OAR›	1.24E+08	0.028743
27	oar3_OAR›	1.24E+08	0.076758
27	oar3_OAR›	1.24E+08	0.076758
27	oar3_OAR›	1.24E+08	0.039426
27	oar3_OAR›	1.24E+08	-0.03113
27	oar3_OAR›	1.24E+08	-0.00942
27	oar3_OAR›	1.24E+08	0.028743
27	oar3_OAR›	1.24E+08	0.086371

27	oar3_OAR>	1.24E+08	-0.02551
27	oar3_OAR>	1.24E+08	-0.01251
27	oar3_OAR>	1.24E+08	0.042659
27	oar3_OAR>	1.24E+08	0.020745
27	oar3_OAR>	1.24E+08	0.02438
27	oar3_OAR>	1.24E+08	-0.02766
27	oar3_OAR>	1.24E+08	0.06675
27	oar3_OAR>	1.24E+08	0.034132
27	oar3_OAR>	1.24E+08	0.008127
27	oar3_OAR>	1.24E+08	0.042623
27	oar3_OAR>	1.24E+08	-0.01461
27	oar3_OAR>	1.24E+08	0.005601
27	oar3_OAR>	1.24E+08	-0.00115
27	oar3_OAR>	1.24E+08	0.017654
27	oar3_OAR>	1.24E+08	0.013077
27	oar3_OAR>	1.24E+08	0.021413
27	oar3_OAR>	1.24E+08	0.003447
27	oar3_OAR>	1.24E+08	0.029081
27	oar3_OAR>	1.24E+08	-0.00942
27	oar3_OAR>	1.24E+08	-0.00942
27	oar3_OAR>	1.24E+08	0.004277
27	oar3_OAR>	1.24E+08	-0.03463
27	oar3_OAR>	1.24E+08	-0.00942
27	oar3_OAR>	1.24E+08	-0.0194
27	oar3_OAR>	1.24E+08	-0.02521
27	oar3_OAR>	1.24E+08	-0.02351
27	oar3_OAR>	1.24E+08	-0.0081
27	oar3_OAR>	1.24E+08	0.002898
27	oar3_OAR>	1.24E+08	-0.04077
27	oar3_OAR>	1.24E+08	-0.01897
27	oar3_OAR>	1.24E+08	-0.01846
27	oar3_OAR>	1.24E+08	-0.01558
27	oar3_OAR>	1.24E+08	-0.0151
27	oar3_OAR>	1.24E+08	-0.0305
27	oar3_OAR>	1.24E+08	-0.0305
27	oar3_OAR>	1.24E+08	0.008437
27	oar3_OAR>	1.24E+08	0.014684
27	oar3_OAR>	1.24E+08	-0.02897
27	oar3_OAR>	1.24E+08	0.002357
27	oar3_OAR>	1.24E+08	0.002357
27	oar3_OAR>	1.24E+08	0.014684
27	oar3_OAR>	1.24E+08	-0.02921
27	oar3_OAR>	1.24E+08	-0.03161
27	oar3_OAR>	1.24E+08	0.005388
27	oar3_OAR>	1.24E+08	0.007039
27	oar3_OAR>	1.24E+08	0.041285
27	oar3_OAR>	1.24E+08	0.027637
27	oar3_OAR>	1.24E+08	-0.01943
27	oar3_OAR>	1.24E+08	-0.01733
27	oar3_OAR>	1.24E+08	0.05493
27	oar3_OAR>	1.24E+08	-0.01139
27	oar3_OAR>	1.24E+08	-0.01611
27	oar3_OAR>	1.24E+08	-0.02857
27	oar3_OAR>	1.24E+08	-0.02725

27	oar3_OAR〉	1.24E+08	0.082222
27	oar3_OAR〉	1.24E+08	-0.01628
27	oar3_OAR〉	1.24E+08	0.008296
27	oar3_OAR〉	1.24E+08	-0.01962
27	oar3_OAR〉	1.24E+08	-0.00591
27	oar3_OAR〉	1.24E+08	-0.02428
27	oar3_OAR〉	1.24E+08	-0.02721
27	oar3_OAR〉	1.24E+08	-0.02063
27	oar3_OAR〉	1.24E+08	-0.00462
27	oar3_OAR〉	1.24E+08	0.015366
27	oar3_OAR〉	1.24E+08	0.006818
27	oar3_OAR〉	1.24E+08	-0.00462
27	oar3_OAR〉	1.24E+08	-0.02593
27	oar3_OAR〉	1.24E+08	0.042336
27	oar3_OAR〉	1.24E+08	0.034468
27	oar3_OAR〉	1.24E+08	0.12265
27	oar3_OAR〉	1.24E+08	0.036769
27	oar3_OAR〉	1.24E+08	0.036769
27	oar3_OAR〉	1.24E+08	-0.01974
27	oar3_OAR〉	1.24E+08	-0.01974
27	oar3_OAR〉	1.24E+08	-0.01974
27	oar3_OAR〉	1.24E+08	-0.01379
27	oar3_OAR〉	1.24E+08	-0.01974
27	oar3_OAR〉	1.24E+08	0.068173
27	oar3_OAR〉	1.24E+08	0.05549
27	oar3_OAR〉	1.24E+08	0.156659
27	oar3_OAR〉	1.24E+08	0.050239
27	oar3_OAR〉	1.24E+08	-0.02941
27	oar3_OAR〉	1.24E+08	-0.00751
27	oar3_OAR〉	1.24E+08	-0.01028
27	oar3_OAR〉	1.24E+08	-0.02778
27	oar3_OAR〉	1.24E+08	-0.02778
27	oar3_OAR〉	1.24E+08	-0.00114
27	oar3_OAR〉	1.24E+08	0.020207
27	oar3_OAR〉	1.24E+08	-0.00114
27	oar3_OAR〉	1.24E+08	0.001585
27	oar3_OAR〉	1.24E+08	-0.01547
27	oar3_OAR〉	1.24E+08	-0.02774
27	oar3_OAR〉	1.24E+08	0.001049
27	oar3_OAR〉	1.24E+08	-0.00815
27	oar3_OAR〉	1.24E+08	-0.01612
27	oar3_OAR〉	1.24E+08	-0.01809
27	oar3_OAR〉	1.24E+08	-0.00576
27	oar3_OAR〉	1.24E+08	-0.00364
27	oar3_OAR〉	1.24E+08	0.019617
27	oar3_OAR〉	1.24E+08	-0.01368
27	oar3_OAR〉	1.24E+08	-0.01709
27	oar3_OAR〉	1.24E+08	-0.00386
27	oar3_OAR〉	1.24E+08	-0.02359
27	oar3_OAR〉	1.24E+08	-0.02359
27	oar3_OAR〉	1.24E+08	-0.02566
27	oar3_OAR〉	1.24E+08	-0.02359
27	oar3_OAR〉	1.24E+08	0.003703
27	oar3_OAR〉	1.24E+08	0.035625

27	oar3_OAR›	1.24E+08	0.003703
27	oar3_OAR›	1.24E+08	0.003703
27	oar3_OAR›	1.24E+08	-0.01531
27	oar3_OAR›	1.24E+08	-0.00892
27	oar3_OAR›	1.24E+08	-0.01938
27	oar3_OAR›	1.24E+08	-0.03265
27	oar3_OAR›	1.24E+08	-0.03265
27	oar3_OAR›	1.24E+08	-0.01368
27	oar3_OAR›	1.24E+08	-0.02187
27	oar3_OAR›	1.24E+08	-0.02271
27	oar3_OAR›	1.24E+08	-0.00376
27	oar3_OAR›	1.24E+08	0.004765
27	oar3_OAR›	1.24E+08	0.008997
27	oar3_OAR›	1.24E+08	0.042537
27	oar3_OAR›	1.24E+08	-0.00445
27	oar3_OAR›	1.24E+08	0.021087
27	oar3_OAR›	1.24E+08	0.010685
27	oar3_OAR›	1.24E+08	0.042961
27	oar3_OAR›	1.24E+08	0.134781
27	oar3_OAR›	1.24E+08	0.122686
27	oar3_OAR›	1.24E+08	-0.0236
27	oar3_OAR›	1.24E+08	0.134781
27	oar3_OAR›	1.24E+08	-0.0027
27	oar3_OAR›	1.24E+08	-0.02072
27	oar3_OAR›	1.24E+08	-0.02072
27	oar3_OAR›	1.24E+08	0.035942
27	oar3_OAR›	1.24E+08	0.096318
27	oar3_OAR›	1.24E+08	0.078253
27	oar3_OAR›	1.24E+08	0.07333
27	oar3_OAR›	1.24E+08	-0.01314
27	oar3_OAR›	1.25E+08	-0.03284
27	oar3_OAR›	1.25E+08	-0.03284
27	oar3_OAR›	1.25E+08	0.000342
27	oar3_OAR›	1.25E+08	-0.03284
27	oar3_OAR›	1.25E+08	-0.03284
27	oar3_OAR›	1.25E+08	0.011761
27	oar3_OAR›	1.25E+08	0.063642
27	oar3_OAR›	1.25E+08	-0.01283
27	oar3_OAR›	1.25E+08	0.028544
27	oar3_OAR›	1.25E+08	-0.02732
27	oar3_OAR›	1.25E+08	0.001628
27	oar3_OAR›	1.25E+08	0.028544
27	oar3_OAR›	1.25E+08	-0.0119
27	oar3_OAR›	1.25E+08	-0.01025
27	oar3_OAR›	1.25E+08	-0.02064
27	oar3_OAR›	1.25E+08	-0.01791
27	oar3_OAR›	1.25E+08	-0.01084
27	oar3_OAR›	1.25E+08	-0.02356
27	oar3_OAR›	1.25E+08	-0.02662
27	oar3_OAR›	1.25E+08	-0.02887
27	oar3_OAR›	1.25E+08	-0.0282
27	oar3_OAR›	1.25E+08	-0.02171
27	oar3_OAR›	1.25E+08	-0.02562
27	oar3_OAR›	1.25E+08	-0.02171

27	oar3_OAR⟩	1.25E+08	-0.0369
27	oar3_OAR⟩	1.25E+08	0.004183
27	oar3_OAR⟩	1.25E+08	0.017565
27	oar3_OAR⟩	1.25E+08	-0.01618
27	oar3_OAR⟩	1.25E+08	0.124831
27	oar3_OAR⟩	1.25E+08	########
27	oar3_OAR⟩	1.25E+08	0.065582
27	oar3_OAR⟩	1.25E+08	-0.01661
27	oar3_OAR⟩	1.25E+08	-0.00058
27	oar3_OAR⟩	1.25E+08	0.01501
27	oar3_OAR⟩	1.25E+08	0.008437
27	oar3_OAR⟩	1.25E+08	0.025014
27	oar3_OAR⟩	1.25E+08	0.037729
27	oar3_OAR⟩	1.25E+08	0.008437
27	oar3_OAR⟩	1.25E+08	0.11152
27	oar3_OAR⟩	1.25E+08	0.008437
27	oar3_OAR⟩	1.25E+08	-0.02889
27	oar3_OAR⟩	1.25E+08	0.025562
27	oar3_OAR⟩	1.25E+08	-0.01108
27	oar3_OAR⟩	1.25E+08	0.068794
27	oar3_OAR⟩	1.25E+08	-0.03298
27	oar3_OAR⟩	1.25E+08	0.073461
27	oar3_OAR⟩	1.25E+08	-0.02159
27	oar3_OAR⟩	1.25E+08	0.022252
27	oar3_OAR⟩	1.25E+08	0.073461
27	oar3_OAR⟩	1.25E+08	0.022252
27	oar3_OAR⟩	1.25E+08	-0.0104
27	oar3_OAR⟩	1.25E+08	0.060364
27	oar3_OAR⟩	1.25E+08	0.044736
27	oar3_OAR⟩	1.25E+08	0.004049
27	oar3_OAR⟩	1.25E+08	0.041488
27	oar3_OAR⟩	1.25E+08	0.024431
27	oar3_OAR⟩	1.25E+08	0.002183
27	oar3_OAR⟩	1.25E+08	-0.01424
27	oar3_OAR⟩	1.25E+08	0.03648
27	oar3_OAR⟩	1.25E+08	-0.02022
27	oar3_OAR⟩	1.25E+08	-0.02022
27	oar3_OAR⟩	1.25E+08	0.043532
27	oar3_OAR⟩	1.25E+08	0.016847
27	oar3_OAR⟩	1.25E+08	0.001137
27	oar3_OAR⟩	1.25E+08	-0.00152
27	oar3_OAR⟩	1.25E+08	-0.02961
27	oar3_OAR⟩	1.25E+08	-0.02961
27	oar3_OAR⟩	1.25E+08	-0.02409
27	oar3_OAR⟩	1.25E+08	-0.02488
27	oar3_OAR⟩	1.25E+08	-0.00255
27	oar3_OAR⟩	1.25E+08	0.034824
27	oar3_OAR⟩	1.25E+08	0.087089
27	oar3_OAR⟩	1.25E+08	0.002764
27	oar3_OAR⟩	1.25E+08	0.002764
27	oar3_OAR⟩	1.25E+08	0.034824
27	oar3_OAR⟩	1.25E+08	0.007181
27	oar3_OAR⟩	1.25E+08	0.11776
27	oar3_OAR⟩	1.25E+08	0.11776

27	oar3_OAR〉	1.25E+08	0.139202
27	oar3_OAR〉	1.25E+08	0.106306
27	oar3_OAR〉	1.25E+08	0.154208
27	oar3_OAR〉	1.25E+08	0.123347
27	oar3_OAR〉	1.25E+08	0.031432
27	oar3_OAR〉	1.25E+08	0.079055
27	oar3_OAR〉	1.25E+08	-0.00654
27	oar3_OAR〉	1.25E+08	0.013378
27	oar3_OAR〉	1.25E+08	0.023492
27	oar3_OAR〉	1.25E+08	0.015483
27	oar3_OAR〉	1.25E+08	-0.01069
27	oar3_OAR〉	1.25E+08	0.167751
27	oar3_OAR〉	1.25E+08	0.167751
27	oar3_OAR〉	1.25E+08	-0.00744
27	oar3_OAR〉	1.25E+08	0.094608
27	oar3_OAR〉	1.25E+08	0.022083
27	oar3_OAR〉	1.25E+08	0.017899
27	oar3_OAR〉	1.25E+08	0.006485
27	oar3_OAR〉	1.25E+08	0.006485
27	oar3_OAR〉	1.25E+08	0.006485
27	oar3_OAR〉	1.25E+08	0.086728
27	oar3_OAR〉	1.25E+08	-0.01316
27	oar3_OAR〉	1.25E+08	0.007416
27	oar3_OAR〉	1.25E+08	-0.00406
27	oar3_OAR〉	1.25E+08	0.081425
27	oar3_OAR〉	1.25E+08	0.025473
27	oar3_OAR〉	1.25E+08	0.009674
27	oar3_OAR〉	1.25E+08	0.028481
27	oar3_OAR〉	1.25E+08	0.009612
27	oar3_OAR〉	1.25E+08	-0.01872
27	oar3_OAR〉	1.25E+08	-0.01705
27	oar3_OAR〉	1.25E+08	-0.01639
27	oar3_OAR〉	1.25E+08	-0.00586
27	oar3_OAR〉	1.25E+08	-0.00586
27	oar3_OAR〉	1.25E+08	0.163385
27	oar3_OAR〉	1.25E+08	0.122864
27	oar3_OAR〉	1.25E+08	0.122864
27	oar3_OAR〉	1.25E+08	0.024935
27	oar3_OAR〉	1.25E+08	0.081447
27	oar3_OAR〉	1.25E+08	-0.01698
27	oar3_OAR〉	1.25E+08	0.106774
27	oar3_OAR〉	1.25E+08	0.106774
27	oar3_OAR〉	1.25E+08	0.045387
27	oar3_OAR〉	1.25E+08	0.128326
27	oar3_OAR〉	1.25E+08	0.045387
27	oar3_OAR〉	1.25E+08	-0.023
27	oar3_OAR〉	1.25E+08	0.105501
27	oar3_OAR〉	1.25E+08	0.110352
27	oar3_OAR〉	1.25E+08	0.055055
27	oar3_OAR〉	1.25E+08	0.019931
27	oar3_OAR〉	1.25E+08	0.032582
27	oar3_OAR〉	1.25E+08	0.130796
27	oar3_OAR〉	1.25E+08	0.03744
27	oar3_OAR〉	1.25E+08	0.014104

27	oar3_OAR〉	1.25E+08	0.03744
27	oar3_OAR〉	1.25E+08	0.022827
27	oar3_OAR〉	1.25E+08	-0.02888
27	oar3_OAR〉	1.25E+08	-0.02304
27	oar3_OAR〉	1.25E+08	0.020903
27	oar3_OAR〉	1.25E+08	-0.02185
27	oar3_OAR〉	1.25E+08	-0.02179
27	oar3_OAR〉	1.25E+08	-0.02371
27	oar3_OAR〉	1.25E+08	-0.02015
27	oar3_OAR〉	1.25E+08	-0.01665
27	oar3_OAR〉	1.25E+08	0.047307
27	oar3_OAR〉	1.25E+08	0.013955
27	oar3_OAR〉	1.25E+08	-0.03339
27	oar3_OAR〉	1.25E+08	-0.02327
27	oar3_OAR〉	1.25E+08	-0.03339
27	oar3_OAR〉	1.25E+08	-0.02993
27	oar3_OAR〉	1.25E+08	-0.01179
27	oar3_OAR〉	1.25E+08	-0.02049
27	oar3_OAR〉	1.25E+08	-0.03068
27	oar3_OAR〉	1.25E+08	0.033956
27	oar3_OAR〉	1.25E+08	0.025112
27	oar3_OAR〉	1.25E+08	0.005471
27	oar3_OAR〉	1.25E+08	0.003593
27	oar3_OAR〉	1.25E+08	-0.01494
27	oar3_OAR〉	1.25E+08	-0.01499
27	oar3_OAR〉	1.25E+08	-0.02049
27	oar3_OAR〉	1.25E+08	-0.03068
27	oar3_OAR〉	1.25E+08	0.02819
27	oar3_OAR〉	1.25E+08	-0.02325
27	oar3_OAR〉	1.25E+08	0.018909
27	oar3_OAR〉	1.25E+08	-0.00847
27	oar3_OAR〉	1.25E+08	-0.03537
27	oar3_OAR〉	1.25E+08	-0.01915
27	oar3_OAR〉	1.25E+08	0.012486
27	oar3_OAR〉	1.25E+08	-0.02683
27	oar3_OAR〉	1.25E+08	0.004844
27	oar3_OAR〉	1.25E+08	-0.01395
27	oar3_OAR〉	1.25E+08	-0.00815
27	oar3_OAR〉	1.25E+08	-0.00788
27	oar3_OAR〉	1.25E+08	-0.03136
27	oar3_OAR〉	1.25E+08	-0.03136
27	oar3_OAR〉	1.25E+08	-0.02031
27	oar3_OAR〉	1.25E+08	-0.00931
27	oar3_OAR〉	1.25E+08	-0.03435
27	oar3_OAR〉	1.25E+08	-0.02486
27	oar3_OAR〉	1.25E+08	-0.03293
27	oar3_OAR〉	1.25E+08	NA
27	oar3_OAR〉	1.25E+08	-0.01892
27	oar3_OAR〉	1.25E+08	-0.02076
27	oar3_OAR〉	1.25E+08	-0.00065
27	oar3_OAR〉	1.25E+08	-0.0289
27	oar3_OAR〉	1.25E+08	-0.01282
27	oar3_OAR〉	1.25E+08	-0.03324
27	oar3_OAR〉	1.25E+08	-0.03324

27	oar3_OAR>	1.25E+08	-0.00328
27	oar3_OAR>	1.25E+08	-0.01369
27	oar3_OAR>	1.25E+08	-0.01369
27	oar3_OAR>	1.25E+08	-0.015
27	oar3_OAR>	1.25E+08	-0.0184
27	oar3_OAR>	1.25E+08	-0.02108
27	oar3_OAR>	1.25E+08	-0.01587
27	oar3_OAR>	1.25E+08	-0.01655
27	oar3_OAR>	1.25E+08	-0.02482
27	oar3_OAR>	1.25E+08	-0.02487
27	oar3_OAR>	1.25E+08	-0.0141
27	oar3_OAR>	1.25E+08	-0.00328
27	oar3_OAR>	1.25E+08	-0.01787
27	oar3_OAR>	1.25E+08	-0.02321
27	oar3_OAR>	1.25E+08	0.023131
27	oar3_OAR>	1.25E+08	-0.00974
27	oar3_OAR>	1.25E+08	-0.02162
27	oar3_OAR>	1.25E+08	0.052177
27	oar3_OAR>	1.25E+08	-0.00642
27	oar3_OAR>	1.25E+08	-0.00454
27	oar3_OAR>	1.25E+08	0.053333
27	oar3_OAR>	1.25E+08	0.059529
27	oar3_OAR>	1.25E+08	-0.02223
27	oar3_OAR>	1.25E+08	-0.00328
27	oar3_OAR>	1.25E+08	0.057289
27	oar3_OAR>	1.26E+08	0.055899
27	oar3_OAR>	1.26E+08	-0.00599
27	oar3_OAR>	1.26E+08	0.023859
27	oar3_OAR>	1.26E+08	-0.03266
27	oar3_OAR>	1.26E+08	-0.03356
27	oar3_OAR>	1.26E+08	0.031023
27	oar3_OAR>	1.26E+08	0.0407
27	oar3_OAR>	1.26E+08	0.063569
27	oar3_OAR>	1.26E+08	-0.03439
27	oar3_OAR>	1.26E+08	0.0137
27	oar3_OAR>	1.26E+08	-0.03061
27	oar3_OAR>	1.26E+08	-0.01655
27	oar3_OAR>	1.26E+08	-0.01731
27	oar3_OAR>	1.26E+08	0.010327
27	oar3_OAR>	1.26E+08	0.010327
27	oar3_OAR>	1.26E+08	0.012138
27	oar3_OAR>	1.26E+08	-0.03637
27	oar3_OAR>	1.26E+08	-0.02698
27	oar3_OAR>	1.26E+08	-0.02856
27	oar3_OAR>	1.26E+08	-0.03582
27	oar3_OAR>	1.26E+08	-0.02856
27	oar3_OAR>	1.26E+08	-0.01069
27	oar3_OAR>	1.26E+08	-0.02886
27	oar3_OAR>	1.26E+08	-0.01602
27	oar3_OAR>	1.26E+08	-0.01414
27	oar3_OAR>	1.26E+08	0.004308
27	oar3_OAR>	1.26E+08	8.02E-05
27	oar3_OAR>	1.26E+08	0.020753
27	oar3_OAR>	1.26E+08	-0.01069

27	oar3_OAR〉	1.26E+08	-0.02427
27	oar3_OAR〉	1.26E+08	0.01417
27	oar3_OAR〉	1.26E+08	0.020688
27	oar3_OAR〉	1.26E+08	-0.02647
27	oar3_OAR〉	1.26E+08	-0.0247
27	oar3_OAR〉	1.26E+08	-0.02744
27	oar3_OAR〉	1.26E+08	0.011893
27	oar3_OAR〉	1.26E+08	0.021138
27	oar3_OAR〉	1.26E+08	-0.01445
27	oar3_OAR〉	1.26E+08	-0.03337
27	oar3_OAR〉	1.26E+08	6.16E-06
27	oar3_OAR〉	1.26E+08	-0.02828
27	oar3_OAR〉	1.26E+08	-0.00508
27	oar3_OAR〉	1.26E+08	-0.013
27	oar3_OAR〉	1.26E+08	-0.00711
27	oar3_OAR〉	1.26E+08	0.042706
27	oar3_OAR〉	1.26E+08	0.031532
27	oar3_OAR〉	1.26E+08	-0.00836
27	oar3_OAR〉	1.26E+08	0.030048
27	oar3_OAR〉	1.26E+08	0.003346
27	oar3_OAR〉	1.26E+08	-0.02217
27	oar3_OAR〉	1.26E+08	0.144395
27	oar3_OAR〉	1.26E+08	0.095503
27	oar3_OAR〉	1.26E+08	-0.02032
27	oar3_OAR〉	1.26E+08	-0.02038
27	oar3_OAR〉	1.26E+08	0.008437
27	oar3_OAR〉	1.26E+08	0.053192
27	oar3_OAR〉	1.26E+08	-0.00187
27	oar3_OAR〉	1.26E+08	-0.02049
27	oar3_OAR〉	1.26E+08	-0.02163
27	oar3_OAR〉	1.26E+08	-0.03107
27	oar3_OAR〉	1.26E+08	0.034929
27	oar3_OAR〉	1.26E+08	-0.00335
27	oar3_OAR〉	1.26E+08	0.07464
27	oar3_OAR〉	1.26E+08	0.004924
27	oar3_OAR〉	1.26E+08	0.120834
27	oar3_OAR〉	1.26E+08	-0.01059
27	oar3_OAR〉	1.26E+08	0.032582
27	oar3_OAR〉	1.26E+08	0.022541
27	oar3_OAR〉	1.26E+08	0.029185
27	oar3_OAR〉	1.26E+08	-0.00174
27	oar3_OAR〉	1.26E+08	-0.01203
27	oar3_OAR〉	1.26E+08	0.044586
27	oar3_OAR〉	1.26E+08	0.044586
27	oar3_OAR〉	1.26E+08	-0.00446
27	oar3_OAR〉	1.26E+08	0.003214
27	oar3_OAR〉	1.26E+08	0.200693
27	oar3_OAR〉	1.26E+08	0.14736
27	oar3_OAR〉	1.26E+08	0.185776
27	oar3_OAR〉	1.26E+08	0.185776
27	oar3_OAR〉	1.26E+08	-0.01287
27	oar3_OAR〉	1.26E+08	0.004406
27	oar3_OAR〉	1.26E+08	NA
27	oar3_OAR〉	1.26E+08	0.061879

27	oar3_OAR〉	1.26E+08	-0.01372
27	oar3_OAR〉	1.26E+08	-0.01368
27	oar3_OAR〉	1.26E+08	0.087052
27	oar3_OAR〉	1.26E+08	0.030671
27	oar3_OAR〉	1.26E+08	0.019236
27	oar3_OAR〉	1.26E+08	-0.02184
27	oar3_OAR〉	1.26E+08	0.042803
27	oar3_OAR〉	1.26E+08	0.021081
27	oar3_OAR〉	1.26E+08	-0.0315
27	oar3_OAR〉	1.26E+08	-0.03888
27	oar3_OAR〉	1.26E+08	0.013013
27	oar3_OAR〉	1.26E+08	0.008906
27	oar3_OAR〉	1.26E+08	0.053273
27	oar3_OAR〉	1.26E+08	0.022466
27	oar3_OAR〉	1.26E+08	0.079931
27	oar3_OAR〉	1.26E+08	-0.02942
27	oar3_OAR〉	1.26E+08	0.04569
27	oar3_OAR〉	1.26E+08	-0.031
27	oar3_OAR〉	1.26E+08	-0.0144
27	oar3_OAR〉	1.26E+08	0.021081
27	oar3_OAR〉	1.26E+08	-0.0132
27	oar3_OAR〉	1.26E+08	0.069823
27	oar3_OAR〉	1.26E+08	0.02035
27	oar3_OAR〉	1.26E+08	-0.02897
27	oar3_OAR〉	1.26E+08	0.069823
27	oar3_OAR〉	1.26E+08	0.020257
27	oar3_OAR〉	1.26E+08	0.109225
27	oar3_OAR〉	1.26E+08	-0.03523
27	oar3_OAR〉	1.26E+08	0.109225
27	oar3_OAR〉	1.26E+08	0.010073
27	oar3_OAR〉	1.26E+08	0.020257
27	oar3_OAR〉	1.26E+08	-0.03823
27	oar3_OAR〉	1.26E+08	0.116931
27	oar3_OAR〉	1.26E+08	-0.00735
27	oar3_OAR〉	1.26E+08	-0.02807
27	oar3_OAR〉	1.26E+08	-0.01618
27	oar3_OAR〉	1.26E+08	0.027365
27	oar3_OAR〉	1.26E+08	0.00215
27	oar3_OAR〉	1.26E+08	0.00215
27	oar3_OAR〉	1.26E+08	0.004689
27	oar3_OAR〉	1.26E+08	-0.02603
27	oar3_OAR〉	1.26E+08	-0.0267
27	oar3_OAR〉	1.26E+08	0.007863
27	oar3_OAR〉	1.26E+08	0.002684
27	oar3_OAR〉	1.26E+08	-0.02719
27	oar3_OAR〉	1.26E+08	0.023093
27	oar3_OAR〉	1.26E+08	0.023093
27	oar3_OAR〉	1.26E+08	-0.00528
27	oar3_OAR〉	1.26E+08	0.015365
27	oar3_OAR〉	1.26E+08	-0.02429
27	oar3_OAR〉	1.26E+08	-0.00209
27	oar3_OAR〉	1.26E+08	-0.02452
27	oar3_OAR〉	1.26E+08	0.068147
27	oar3_OAR〉	1.26E+08	0.022632

27	oar3_OAR〉	1.26E+08	-0.03242
27	oar3_OAR〉	1.26E+08	0.018227
27	oar3_OAR〉	1.26E+08	-0.02328
27	oar3_OAR〉	1.26E+08	-0.00965
27	oar3_OAR〉	1.26E+08	0.010877
27	oar3_OAR〉	1.26E+08	-0.02481
27	oar3_OAR〉	1.26E+08	-0.0057
27	oar3_OAR〉	1.26E+08	-0.01584
27	oar3_OAR〉	1.26E+08	-0.02481
27	oar3_OAR〉	1.26E+08	-0.034
27	oar3_OAR〉	1.26E+08	0.004281
27	oar3_OAR〉	1.26E+08	-0.00731
27	oar3_OAR〉	1.26E+08	0.049514
27	oar3_OAR〉	1.26E+08	0.015859
27	oar3_OAR〉	1.26E+08	0.014137
27	oar3_OAR〉	1.26E+08	-0.02454
27	oar3_OAR〉	1.26E+08	-0.02996
27	oar3_OAR〉	1.26E+08	0.025821
27	oar3_OAR〉	1.26E+08	-0.00493
27	oar3_OAR〉	1.26E+08	0.005356
27	oar3_OAR〉	1.26E+08	0.00088
27	oar3_OAR〉	1.26E+08	0.006084
27	oar3_OAR〉	1.26E+08	0.094335
27	oar3_OAR〉	1.26E+08	0.061979
27	oar3_OAR〉	1.26E+08	0.011101
27	oar3_OAR〉	1.26E+08	0.00481
27	oar3_OAR〉	1.26E+08	0.041107
27	oar3_OAR〉	1.26E+08	0.00481
27	oar3_OAR〉	1.26E+08	0.021764
27	oar3_OAR〉	1.26E+08	0.021764
27	oar3_OAR〉	1.26E+08	0.017753
27	oar3_OAR〉	1.26E+08	0.008677
27	oar3_OAR〉	1.26E+08	0.057063
27	oar3_OAR〉	1.26E+08	0.061065
27	oar3_OAR〉	1.26E+08	-0.00816
27	oar3_OAR〉	1.26E+08	-0.01078
27	oar3_OAR〉	1.26E+08	-0.00816
27	oar3_OAR〉	1.26E+08	-0.0294
27	oar3_OAR〉	1.26E+08	-0.02647
27	oar3_OAR〉	1.26E+08	-0.02642
27	oar3_OAR〉	1.26E+08	0.009487
27	oar3_OAR〉	1.26E+08	-0.00195
27	oar3_OAR〉	1.26E+08	-0.02287
27	oar3_OAR〉	1.26E+08	0.007282
27	oar3_OAR〉	1.26E+08	-0.02104
27	oar3_OAR〉	1.26E+08	-0.03276
27	oar3_OAR〉	1.26E+08	-0.00751
27	oar3_OAR〉	1.26E+08	-0.02647
27	oar3_OAR〉	1.26E+08	0.032094
27	oar3_OAR〉	1.26E+08	0.022721
27	oar3_OAR〉	1.26E+08	0.0048
27	oar3_OAR〉	1.26E+08	0.038295
27	oar3_OAR〉	1.26E+08	0.00181
27	oar3_OAR〉	1.26E+08	-0.03144

27	oar3_OAR〉	1.26E+08	-0.03673
27	oar3_OAR〉	1.26E+08	-0.03681
27	oar3_OAR〉	1.26E+08	-0.01753
27	oar3_OAR〉	1.26E+08	-0.03515
27	oar3_OAR〉	1.26E+08	0.00481
27	oar3_OAR〉	1.26E+08	-0.03681
27	oar3_OAR〉	1.26E+08	0.003447
27	oar3_OAR〉	1.26E+08	-0.03869
27	oar3_OAR〉	1.26E+08	-0.01766
27	oar3_OAR〉	1.26E+08	-0.02135
27	oar3_OAR〉	1.26E+08	-0.02926
27	oar3_OAR〉	1.26E+08	-0.02121
27	oar3_OAR〉	1.26E+08	-0.02926
27	oar3_OAR〉	1.26E+08	0.043017
27	oar3_OAR〉	1.26E+08	-0.03174
27	oar3_OAR〉	1.27E+08	-0.00934
27	oar3_OAR〉	1.27E+08	0.020399
27	oar3_OAR〉	1.27E+08	-0.00934
27	oar3_OAR〉	1.27E+08	0.049894
27	oar3_OAR〉	1.27E+08	-0.0282
27	oar3_OAR〉	1.27E+08	-0.01674
27	oar3_OAR〉	1.27E+08	-0.0282
27	oar3_OAR〉	1.27E+08	0.023625
27	oar3_OAR〉	1.27E+08	0.02205
27	oar3_OAR〉	1.27E+08	-0.03135
27	oar3_OAR〉	1.27E+08	-0.01698
27	oar3_OAR〉	1.27E+08	0.053987
27	oar3_OAR〉	1.27E+08	0.100633
27	oar3_OAR〉	1.27E+08	-0.01069
27	oar3_OAR〉	1.27E+08	0.036251
27	oar3_OAR〉	1.27E+08	0.011067
27	oar3_OAR〉	1.27E+08	0.012361
27	oar3_OAR〉	1.27E+08	0.041221
27	oar3_OAR〉	1.27E+08	NA
27	oar3_OAR〉	1.27E+08	0.00043
27	oar3_OAR〉	1.27E+08	-0.00626
27	oar3_OAR〉	1.27E+08	0.054963
27	oar3_OAR〉	1.27E+08	-0.00024
27	oar3_OAR〉	1.27E+08	0.031403
27	oar3_OAR〉	1.27E+08	-0.02208
27	oar3_OAR〉	1.27E+08	-0.0168
27	oar3_OAR〉	1.27E+08	0.016059
27	oar3_OAR〉	1.27E+08	0.042346
27	oar3_OAR〉	1.27E+08	-0.01139
27	oar3_OAR〉	1.27E+08	0.046161
27	oar3_OAR〉	1.27E+08	-0.01827
27	oar3_OAR〉	1.27E+08	-0.02523
27	oar3_OAR〉	1.27E+08	-0.03377
27	oar3_OAR〉	1.27E+08	0.022722
27	oar3_OAR〉	1.27E+08	0.01603
27	oar3_OAR〉	1.27E+08	-0.02715
27	oar3_OAR〉	1.27E+08	-0.00996
27	oar3_OAR〉	1.27E+08	0.056459
27	oar3_OAR〉	1.27E+08	0.034005

27	oar3_OAR›	1.27E+08	-0.00768
27	oar3_OAR›	1.27E+08	-0.01914
27	oar3_OAR›	1.27E+08	-0.04127
27	oar3_OAR›	1.27E+08	-0.00195
27	oar3_OAR›	1.27E+08	-0.01702
27	oar3_OAR›	1.27E+08	0.014713
27	oar3_OAR›	1.27E+08	0.009626
27	oar3_OAR›	1.27E+08	-0.00283
27	oar3_OAR›	1.27E+08	0.009324
27	oar3_OAR›	1.27E+08	-0.01891
27	oar3_OAR›	1.27E+08	0.004924
27	oar3_OAR›	1.27E+08	0.024876
27	oar3_OAR›	1.27E+08	0.04162
27	oar3_OAR›	1.27E+08	-0.02784
27	oar3_OAR›	1.27E+08	-0.01255
27	oar3_OAR›	1.27E+08	0.007587
27	oar3_OAR›	1.27E+08	0.02374
27	oar3_OAR›	1.27E+08	0.081111
27	oar3_OAR›	1.27E+08	0.225358
27	oar3_OAR›	1.27E+08	0.225358
27	oar3_OAR›	1.27E+08	0.225358
27	oar3_OAR›	1.27E+08	0.14405
27	oar3_OAR›	1.27E+08	0.079966
27	oar3_OAR›	1.27E+08	-0.01735
27	oar3_OAR›	1.27E+08	-0.02168
27	oar3_OAR›	1.27E+08	-0.01456
27	oar3_OAR›	1.27E+08	-0.00142
27	oar3_OAR›	1.27E+08	0.061975
27	oar3_OAR›	1.27E+08	0.037258
27	oar3_OAR›	1.27E+08	0.008805
27	oar3_OAR›	1.27E+08	0.000321
27	oar3_OAR›	1.27E+08	-0.0047
27	oar3_OAR›	1.27E+08	-0.00829
27	oar3_OAR›	1.27E+08	-0.03145
27	oar3_OAR›	1.27E+08	-0.00083
27	oar3_OAR›	1.27E+08	-0.0178
27	oar3_OAR›	1.27E+08	0.011067
27	oar3_OAR›	1.27E+08	0.036102
27	oar3_OAR›	1.27E+08	0.01778
27	oar3_OAR›	1.27E+08	0.01778
27	oar3_OAR›	1.27E+08	0.01778
27	oar3_OAR›	1.27E+08	0.018082
27	oar3_OAR›	1.27E+08	0.037721
27	oar3_OAR›	1.27E+08	0.028704
27	oar3_OAR›	1.27E+08	0.028743
27	oar3_OAR›	1.27E+08	0.020753
27	oar3_OAR›	1.27E+08	0.007292
27	oar3_OAR›	1.27E+08	-0.00995
27	oar3_OAR›	1.27E+08	-0.03709
27	oar3_OAR›	1.27E+08	-0.00685
27	oar3_OAR›	1.27E+08	-0.0228
27	oar3_OAR›	1.27E+08	-0.02272
27	oar3_OAR›	1.27E+08	0.060142
27	oar3_OAR›	1.27E+08	0.072479

27	oar3_OAR〉	1.27E+08	0.085766
27	oar3_OAR〉	1.27E+08	-0.03041
27	oar3_OAR〉	1.27E+08	-0.02822
27	oar3_OAR〉	1.27E+08	-0.02179
27	oar3_OAR〉	1.27E+08	0.015298
27	oar3_OAR〉	1.27E+08	-0.02388
27	oar3_OAR〉	1.27E+08	0.115215
27	oar3_OAR〉	1.27E+08	0.05157
27	oar3_OAR〉	1.27E+08	0.005261
27	oar3_OAR〉	1.27E+08	0.003805
27	oar3_OAR〉	1.27E+08	-0.02944
27	oar3_OAR〉	1.27E+08	0.020791
27	oar3_OAR〉	1.27E+08	-0.02944
27	oar3_OAR〉	1.27E+08	-0.02114
27	oar3_OAR〉	1.27E+08	-0.00328
27	oar3_OAR〉	1.27E+08	0.013963
27	oar3_OAR〉	1.27E+08	-0.02668
27	oar3_OAR〉	1.27E+08	-0.00328
27	oar3_OAR〉	1.27E+08	-0.01902
27	oar3_OAR〉	1.27E+08	0.013963
27	oar3_OAR〉	1.27E+08	0.010148
27	oar3_OAR〉	1.27E+08	-0.0108
27	oar3_OAR〉	1.27E+08	0.010148
27	oar3_OAR〉	1.27E+08	-0.00328
27	oar3_OAR〉	1.27E+08	-0.00328
27	oar3_OAR〉	1.27E+08	-0.01963
27	oar3_OAR〉	1.27E+08	-0.00328
27	oar3_OAR〉	1.27E+08	-0.00328
27	oar3_OAR〉	1.27E+08	0.000729
27	oar3_OAR〉	1.27E+08	0.00037
27	oar3_OAR〉	1.27E+08	-0.03186
27	oar3_OAR〉	1.27E+08	-0.01034
27	oar3_OAR〉	1.27E+08	0.065627
27	oar3_OAR〉	1.27E+08	-0.01815
27	oar3_OAR〉	1.27E+08	-0.03673
27	oar3_OAR〉	1.27E+08	-0.03673
27	oar3_OAR〉	1.27E+08	-0.03673
27	oar3_OAR〉	1.27E+08	0.063599
27	oar3_OAR〉	1.27E+08	-0.03673
27	oar3_OAR〉	1.27E+08	-0.03673
27	oar3_OAR〉	1.27E+08	-0.01024
27	oar3_OAR〉	1.27E+08	0.008012
27	oar3_OAR〉	1.27E+08	0.038847
27	oar3_OAR〉	1.27E+08	-0.03679
27	oar3_OAR〉	1.27E+08	0.007414
27	oar3_OAR〉	1.27E+08	-0.03069
27	oar3_OAR〉	1.27E+08	-0.01437
27	oar3_OAR〉	1.27E+08	-0.02727
27	oar3_OAR〉	1.27E+08	-0.02142
27	oar3_OAR〉	1.27E+08	-0.02064
27	oar3_OAR〉	1.27E+08	0.019889
27	oar3_OAR〉	1.27E+08	-0.01092
27	oar3_OAR〉	1.27E+08	0.031909
27	oar3_OAR〉	1.27E+08	-0.03369

27	oar3_OAR〉	1.27E+08	0.000574
27	oar3_OAR〉	1.27E+08	0.032223
27	oar3_OAR〉	1.27E+08	-0.02442
27	oar3_OAR〉	1.27E+08	0.027445
27	oar3_OAR〉	1.27E+08	0.027445
27	oar3_OAR〉	1.27E+08	-0.00584
27	oar3_OAR〉	1.27E+08	-0.0014
27	oar3_OAR〉	1.27E+08	0.002357
27	oar3_OAR〉	1.27E+08	0.102107
27	oar3_OAR〉	1.27E+08	0.102107
27	oar3_OAR〉	1.27E+08	0.094947
27	oar3_OAR〉	1.27E+08	0.028151
27	oar3_OAR〉	1.27E+08	0.00976
27	oar3_OAR〉	1.27E+08	-0.01317
27	oar3_OAR〉	1.27E+08	-0.01317
27	oar3_OAR〉	1.27E+08	-0.0131
27	oar3_OAR〉	1.27E+08	-0.01887
27	oar3_OAR〉	1.27E+08	-0.01887
27	oar3_OAR〉	1.27E+08	0.027255
27	oar3_OAR〉	1.27E+08	-0.02022
27	oar3_OAR〉	1.27E+08	-0.01887
27	oar3_OAR〉	1.27E+08	-0.01887
27	oar3_OAR〉	1.27E+08	0.028715
27	oar3_OAR〉	1.27E+08	0.016986
27	oar3_OAR〉	1.27E+08	0.018082
27	oar3_OAR〉	1.27E+08	0.018082
27	oar3_OAR〉	1.27E+08	-0.01047
27	oar3_OAR〉	1.27E+08	-0.01072
27	oar3_OAR〉	1.27E+08	0.113591
27	oar3_OAR〉	1.27E+08	-0.02693
27	oar3_OAR〉	1.27E+08	0.010873
27	oar3_OAR〉	1.27E+08	-0.01072
27	oar3_OAR〉	1.27E+08	0.202216
27	oar3_OAR〉	1.27E+08	0.009985
27	oar3_OAR〉	1.27E+08	0.062652
27	oar3_OAR〉	1.27E+08	0.03967
27	oar3_OAR〉	1.27E+08	0.077185
27	oar3_OAR〉	1.27E+08	-0.01698
27	oar3_OAR〉	1.27E+08	7.84E-05
27	oar3_OAR〉	1.27E+08	0.03213
27	oar3_OAR〉	1.27E+08	-0.00349
27	oar3_OAR〉	1.27E+08	0.074553
27	oar3_OAR〉	1.27E+08	-0.00422
27	oar3_OAR〉	1.27E+08	-0.03188
27	oar3_OAR〉	1.27E+08	-0.00685
27	oar3_OAR〉	1.27E+08	-0.03208
27	oar3_OAR〉	1.27E+08	-0.0092
27	oar3_OAR〉	1.27E+08	-0.00685
27	oar3_OAR〉	1.27E+08	-0.0287
27	oar3_OAR〉	1.27E+08	-0.02767
27	oar3_OAR〉	1.27E+08	-0.02767
27	oar3_OAR〉	1.27E+08	-0.03369
27	oar3_OAR〉	1.27E+08	-0.03356
27	oar3_OAR〉	1.27E+08	-0.03356

27	oar3_OAR〉	1.27E+08	-0.02186
27	oar3_OAR〉	1.27E+08	-0.00839
27	oar3_OAR〉	1.27E+08	-0.03979
27	oar3_OAR〉	1.27E+08	-0.00114
27	oar3_OAR〉	1.27E+08	-0.03979
27	oar3_OAR〉	1.27E+08	-0.03751
27	oar3_OAR〉	1.27E+08	0.004403
27	oar3_OAR〉	1.27E+08	-0.01702
27	oar3_OAR〉	1.27E+08	-0.01595
27	oar3_OAR〉	1.27E+08	-0.03382
27	oar3_OAR〉	1.27E+08	-0.02008
27	oar3_OAR〉	1.27E+08	-0.02008
27	oar3_OAR〉	1.27E+08	-0.00356
27	oar3_OAR〉	1.27E+08	-0.01891
27	oar3_OAR〉	1.27E+08	-0.03382
27	oar3_OAR〉	1.27E+08	-0.02831
27	oar3_OAR〉	1.27E+08	-0.0146
27	oar3_OAR〉	1.27E+08	-0.00606
27	oar3_OAR〉	1.27E+08	-0.00524
27	oar3_OAR〉	1.27E+08	-0.01804
27	oar3_OAR〉	1.27E+08	-0.00864
27	oar3_OAR〉	1.28E+08	-0.02926
27	oar3_OAR〉	1.28E+08	-0.02817
27	oar3_OAR〉	1.28E+08	0.024845
27	oar3_OAR〉	1.28E+08	0.024845
27	oar3_OAR〉	1.28E+08	0.047692
27	oar3_OAR〉	1.28E+08	0.038645
27	oar3_OAR〉	1.28E+08	0.00543
27	oar3_OAR〉	1.28E+08	-0.02205
27	oar3_OAR〉	1.28E+08	0.005022
27	oar3_OAR〉	1.28E+08	0.013718
27	oar3_OAR〉	1.28E+08	-0.03284
27	oar3_OAR〉	1.28E+08	-0.01803
27	oar3_OAR〉	1.28E+08	-0.03284
27	oar3_OAR〉	1.28E+08	-0.01891
27	oar3_OAR〉	1.28E+08	-0.02507
27	oar3_OAR〉	1.28E+08	-0.01445
27	oar3_OAR〉	1.28E+08	0.00937
27	oar3_OAR〉	1.28E+08	0.001429
27	oar3_OAR〉	1.28E+08	0.060992
27	oar3_OAR〉	1.28E+08	-0.02701
27	oar3_OAR〉	1.28E+08	-0.01883
27	oar3_OAR〉	1.28E+08	-0.0172
27	oar3_OAR〉	1.28E+08	-0.02452
27	oar3_OAR〉	1.28E+08	-0.0172
27	oar3_OAR〉	1.28E+08	-0.02922
27	oar3_OAR〉	1.28E+08	-0.01818
27	oar3_OAR〉	1.28E+08	-0.00923
27	oar3_OAR〉	1.28E+08	-0.02843
27	oar3_OAR〉	1.28E+08	-0.03673
27	oar3_OAR〉	1.28E+08	-0.01131
27	oar3_OAR〉	1.28E+08	-0.02922
27	oar3_OAR〉	1.28E+08	-0.01779
27	oar3_OAR〉	1.28E+08	0.078057

27	oar3_OAR>	1.28E+08	0.150243
27	oar3_OAR>	1.28E+08	-0.03332
27	oar3_OAR>	1.28E+08	-0.01303
27	oar3_OAR>	1.28E+08	-0.03169
27	oar3_OAR>	1.28E+08	0.090466
27	oar3_OAR>	1.28E+08	-0.02362
27	oar3_OAR>	1.28E+08	0.004591
27	oar3_OAR>	1.28E+08	-0.02551
27	oar3_OAR>	1.28E+08	-0.02551
27	oar3_OAR>	1.28E+08	-0.01581
27	oar3_OAR>	1.28E+08	-0.02551
27	oar3_OAR>	1.28E+08	-0.01581
27	oar3_OAR>	1.28E+08	-0.01758
27	oar3_OAR>	1.28E+08	0.010439
27	oar3_OAR>	1.28E+08	-0.03617
27	oar3_OAR>	1.28E+08	0.049642
27	oar3_OAR>	1.28E+08	-0.02004
27	oar3_OAR>	1.28E+08	-0.02208
27	oar3_OAR>	1.28E+08	-0.03186
27	oar3_OAR>	1.28E+08	-0.0299
27	oar3_OAR>	1.28E+08	-0.02591
27	oar3_OAR>	1.28E+08	-0.03222
27	oar3_OAR>	1.28E+08	-0.03662
27	oar3_OAR>	1.28E+08	-0.03573
27	oar3_OAR>	1.28E+08	0.034634
27	oar3_OAR>	1.28E+08	-0.03508
27	oar3_OAR>	1.28E+08	-0.02996
27	oar3_OAR>	1.28E+08	-0.03508
27	oar3_OAR>	1.28E+08	-0.02444
27	oar3_OAR>	1.28E+08	-0.01872
27	oar3_OAR>	1.28E+08	0.00258
27	oar3_OAR>	1.28E+08	-0.02793
27	oar3_OAR>	1.28E+08	0.00258
27	oar3_OAR>	1.28E+08	0.01645
27	oar3_OAR>	1.28E+08	-0.0248
27	oar3_OAR>	1.28E+08	-0.0248
27	oar3_OAR>	1.28E+08	0.007434
27	oar3_OAR>	1.28E+08	-0.01594
27	oar3_OAR>	1.28E+08	-0.01069
27	oar3_OAR>	1.28E+08	-0.01594
27	oar3_OAR>	1.28E+08	-0.01069
27	oar3_OAR>	1.28E+08	0.023793
27	oar3_OAR>	1.28E+08	-0.02123
27	oar3_OAR>	1.28E+08	-0.03515
27	oar3_OAR>	1.28E+08	0.034231
27	oar3_OAR>	1.28E+08	-0.02727
27	oar3_OAR>	1.28E+08	-0.00815
27	oar3_OAR>	1.28E+08	-0.01782
27	oar3_OAR>	1.28E+08	-0.03079
27	oar3_OAR>	1.28E+08	-0.01019
27	oar3_OAR>	1.28E+08	-0.02237
27	oar3_OAR>	1.28E+08	-0.02237
27	oar3_OAR>	1.28E+08	-0.03184
27	oar3_OAR>	1.28E+08	-0.02667

27	oar3_OAR〉	1.28E+08	0.00181
27	oar3_OAR〉	1.28E+08	-0.01939
27	oar3_OAR〉	1.28E+08	-0.01939
27	oar3_OAR〉	1.28E+08	-0.03134
27	oar3_OAR〉	1.28E+08	-0.01274
27	oar3_OAR〉	1.28E+08	0.054952
27	oar3_OAR〉	1.28E+08	0.067845
27	oar3_OAR〉	1.28E+08	0.050926
27	oar3_OAR〉	1.28E+08	-0.02486
27	oar3_OAR〉	1.28E+08	-0.02036
27	oar3_OAR〉	1.28E+08	0.012162
27	oar3_OAR〉	1.28E+08	-0.0106
27	oar3_OAR〉	1.28E+08	-0.02607
27	oar3_OAR〉	1.28E+08	-0.0106
27	oar3_OAR〉	1.28E+08	-0.0106
27	oar3_OAR〉	1.28E+08	-0.0106
27	oar3_OAR〉	1.28E+08	-0.02341
27	oar3_OAR〉	1.28E+08	-0.0106
27	oar3_OAR〉	1.28E+08	-0.0106
27	oar3_OAR〉	1.28E+08	-0.0078
27	oar3_OAR〉	1.28E+08	-0.01989
27	oar3_OAR〉	1.28E+08	-0.02891
27	oar3_OAR〉	1.28E+08	-0.01787
27	oar3_OAR〉	1.28E+08	-0.00568
27	oar3_OAR〉	1.28E+08	-0.01639
27	oar3_OAR〉	1.28E+08	-0.03043
27	oar3_OAR〉	1.28E+08	-0.02674
27	oar3_OAR〉	1.28E+08	-0.01771
27	oar3_OAR〉	1.28E+08	-0.02276
27	oar3_OAR〉	1.28E+08	-0.0216
27	oar3_OAR〉	1.28E+08	-0.00953
27	oar3_OAR〉	1.28E+08	-0.00953
27	oar3_OAR〉	1.28E+08	-0.00953
27	oar3_OAR〉	1.28E+08	-0.01292
27	oar3_OAR〉	1.28E+08	-0.00793
27	oar3_OAR〉	1.28E+08	-0.00322
27	oar3_OAR〉	1.28E+08	-0.01655
27	oar3_OAR〉	1.28E+08	-0.03673
27	oar3_OAR〉	1.28E+08	-0.00463
27	oar3_OAR〉	1.28E+08	-0.02895
27	oar3_OAR〉	1.28E+08	0.051941
27	oar3_OAR〉	1.28E+08	-0.00844
27	oar3_OAR〉	1.28E+08	0.066778
27	oar3_OAR〉	1.28E+08	0.091867
27	oar3_OAR〉	1.28E+08	-0.00901
27	oar3_OAR〉	1.28E+08	0.022003
27	oar3_OAR〉	1.28E+08	-0.0224
27	oar3_OAR〉	1.28E+08	0.049764
27	oar3_OAR〉	1.28E+08	0.013657
27	oar3_OAR〉	1.28E+08	0.013657
27	oar3_OAR〉	1.28E+08	0.002171
27	oar3_OAR〉	1.28E+08	-0.01333
27	oar3_OAR〉	1.29E+08	-0.00776
27	oar3_OAR〉	1.29E+08	0.002539

27	oar3_OAR⟩	1.29E+08	-0.02105
27	oar3_OAR⟩	1.29E+08	-0.02362
27	oar3_OAR⟩	1.29E+08	0.0082
27	oar3_OAR⟩	1.29E+08	0.018227
27	oar3_OAR⟩	1.29E+08	0.008291
27	oar3_OAR⟩	1.29E+08	0.016959
27	oar3_OAR⟩	1.29E+08	0.057529
27	oar3_OAR⟩	1.29E+08	0.009671
27	oar3_OAR⟩	1.29E+08	-0.00263
27	oar3_OAR⟩	1.29E+08	-0.01902
27	oar3_OAR⟩	1.29E+08	0.044847
27	oar3_OAR⟩	1.29E+08	0.040198
27	oar3_OAR⟩	1.29E+08	0.010216
27	oar3_OAR⟩	1.29E+08	-0.00193
27	oar3_OAR⟩	1.29E+08	-0.03255
27	oar3_OAR⟩	1.29E+08	-0.02425
27	oar3_OAR⟩	1.29E+08	0.03648
27	oar3_OAR⟩	1.29E+08	0.044847
27	oar3_OAR⟩	1.29E+08	-0.03212
27	oar3_OAR⟩	1.29E+08	-0.02238
27	oar3_OAR⟩	1.29E+08	-0.03853
27	oar3_OAR⟩	1.29E+08	-0.02842
27	oar3_OAR⟩	1.29E+08	-0.01466
27	oar3_OAR⟩	1.29E+08	-0.03457
27	oar3_OAR⟩	1.29E+08	-0.0171
27	oar3_OAR⟩	1.29E+08	0.000889
27	oar3_OAR⟩	1.29E+08	-0.04221
27	oar3_OAR⟩	1.29E+08	0.069636
27	oar3_OAR⟩	1.29E+08	0.027354
27	oar3_OAR⟩	1.29E+08	NA
27	oar3_OAR⟩	1.29E+08	0.05212
27	oar3_OAR⟩	1.29E+08	-0.03938
27	oar3_OAR⟩	1.29E+08	-0.03091
27	oar3_OAR⟩	1.29E+08	0.071879
27	oar3_OAR⟩	1.29E+08	0.064966
27	oar3_OAR⟩	1.29E+08	0.244587
27	oar3_OAR⟩	1.29E+08	0.268564
27	oar3_OAR⟩	1.29E+08	0.000134
27	oar3_OAR⟩	1.29E+08	0.129725
27	oar3_OAR⟩	1.29E+08	-0.01389
27	oar3_OAR⟩	1.29E+08	-0.03151
27	oar3_OAR⟩	1.29E+08	0.111254
27	oar3_OAR⟩	1.29E+08	-0.02077
27	oar3_OAR⟩	1.29E+08	0.088731
27	oar3_OAR⟩	1.29E+08	0.06609
27	oar3_OAR⟩	1.29E+08	-0.02348
27	oar3_OAR⟩	1.29E+08	0.145895
27	oar3_OAR⟩	1.29E+08	-0.01395
27	oar3_OAR⟩	1.29E+08	0.088961
27	oar3_OAR⟩	1.29E+08	-0.0065
27	oar3_OAR⟩	1.29E+08	0.152967
27	oar3_OAR⟩	1.29E+08	-0.0139
27	oar3_OAR⟩	1.29E+08	0.064704
27	oar3_OAR⟩	1.29E+08	0.069853

27	oar3_OAR›	1.29E+08	0.046998
27	oar3_OAR›	1.29E+08	0.069853
27	oar3_OAR›	1.29E+08	-0.0139
27	oar3_OAR›	1.29E+08	0.069853
27	oar3_OAR›	1.29E+08	0.064704
27	oar3_OAR›	1.29E+08	0.046998
27	oar3_OAR›	1.29E+08	-0.01445
27	oar3_OAR›	1.29E+08	0.018202
27	oar3_OAR›	1.29E+08	0.031441
27	oar3_OAR›	1.29E+08	0.007107
27	oar3_OAR›	1.29E+08	0.035505
27	oar3_OAR›	1.29E+08	0.031119
27	oar3_OAR›	1.29E+08	0.035505
27	oar3_OAR›	1.29E+08	NA
27	oar3_OAR›	1.29E+08	0.098972
27	oar3_OAR›	1.29E+08	0.098972
27	oar3_OAR›	1.29E+08	0.07308
27	oar3_OAR›	1.29E+08	0.098972
27	oar3_OAR›	1.29E+08	-0.01595
27	oar3_OAR›	1.29E+08	-0.00452
27	oar3_OAR›	1.29E+08	0.098972
27	oar3_OAR›	1.29E+08	0.019075
27	oar3_OAR›	1.29E+08	0.032053
27	oar3_OAR›	1.29E+08	0.012231
27	oar3_OAR›	1.29E+08	0.012231
27	oar3_OAR›	1.29E+08	0.044814
27	oar3_OAR›	1.29E+08	0.012231
27	oar3_OAR›	1.29E+08	0.147737
27	oar3_OAR›	1.29E+08	0.012231
27	oar3_OAR›	1.29E+08	0.147737
27	oar3_OAR›	1.29E+08	0.044814
27	oar3_OAR›	1.29E+08	0.109449
27	oar3_OAR›	1.29E+08	0.002762
27	oar3_OAR›	1.29E+08	0.006682
27	oar3_OAR›	1.29E+08	0.044898
27	oar3_OAR›	1.29E+08	0.147737
27	oar3_OAR›	1.29E+08	0.006682
27	oar3_OAR›	1.29E+08	0.007497
27	oar3_OAR›	1.29E+08	0.007497
27	oar3_OAR›	1.29E+08	0.147737
27	oar3_OAR›	1.29E+08	0.11687
27	oar3_OAR›	1.29E+08	-0.01534
27	oar3_OAR›	1.29E+08	-0.02156
27	oar3_OAR›	1.29E+08	-0.02156
27	oar3_OAR›	1.29E+08	-0.02845
27	oar3_OAR›	1.29E+08	0.059482
27	oar3_OAR›	1.29E+08	0.059482
27	oar3_OAR›	1.29E+08	-0.01069
27	oar3_OAR›	1.29E+08	-0.00169
27	oar3_OAR›	1.29E+08	0.01985
27	oar3_OAR›	1.29E+08	-0.02454
27	oar3_OAR›	1.29E+08	-0.03466
27	oar3_OAR›	1.29E+08	-0.01423
27	oar3_OAR›	1.29E+08	-0.0205

27	oar3_OAR〉	1.29E+08	-0.01423
27	oar3_OAR〉	1.29E+08	-0.03242
27	oar3_OAR〉	1.29E+08	0.089761
27	oar3_OAR〉	1.29E+08	0.10648
27	oar3_OAR〉	1.29E+08	0.022507
27	oar3_OAR〉	1.29E+08	0.022507
27	oar3_OAR〉	1.29E+08	0.089761
27	oar3_OAR〉	1.29E+08	-0.02509
27	oar3_OAR〉	1.29E+08	-0.00407
27	oar3_OAR〉	1.29E+08	0.029767
27	oar3_OAR〉	1.29E+08	-0.01069
27	oar3_OAR〉	1.29E+08	-0.04289
27	oar3_OAR〉	1.29E+08	-0.02822
27	oar3_OAR〉	1.29E+08	0.019868
27	oar3_OAR〉	1.29E+08	-0.01069
27	oar3_OAR〉	1.29E+08	-0.00864
27	oar3_OAR〉	1.29E+08	-0.01069
27	oar3_OAR〉	1.29E+08	-0.00994
27	oar3_OAR〉	1.29E+08	-0.01069
27	oar3_OAR〉	1.29E+08	-0.00849
27	oar3_OAR〉	1.29E+08	-0.02957
27	oar3_OAR〉	1.29E+08	-0.02926
27	oar3_OAR〉	1.29E+08	-0.01567
27	oar3_OAR〉	1.29E+08	-0.00446
27	oar3_OAR〉	1.29E+08	-0.00187
27	oar3_OAR〉	1.29E+08	-0.01762
27	oar3_OAR〉	1.29E+08	-0.01519
27	oar3_OAR〉	1.29E+08	0.013068
27	oar3_OAR〉	1.29E+08	-0.03156
27	oar3_OAR〉	1.29E+08	0.022016
27	oar3_OAR〉	1.29E+08	-0.00734
27	oar3_OAR〉	1.29E+08	0.022016
27	oar3_OAR〉	1.29E+08	-0.00837
27	oar3_OAR〉	1.29E+08	-0.00734
27	oar3_OAR〉	1.29E+08	0.044471
27	oar3_OAR〉	1.29E+08	-0.02714
27	oar3_OAR〉	1.29E+08	-0.02053
27	oar3_OAR〉	1.29E+08	0.044724
27	oar3_OAR〉	1.29E+08	-0.02053
27	oar3_OAR〉	1.29E+08	-0.02205
27	oar3_OAR〉	1.29E+08	0.090701
27	oar3_OAR〉	1.29E+08	0.048476
27	oar3_OAR〉	1.29E+08	-0.00837
27	oar3_OAR〉	1.29E+08	0.042763
27	oar3_OAR〉	1.29E+08	-0.02151
27	oar3_OAR〉	1.29E+08	0.038505
27	oar3_OAR〉	1.29E+08	-0.03287
27	oar3_OAR〉	1.29E+08	-0.02716
27	oar3_OAR〉	1.29E+08	-0.02884
27	oar3_OAR〉	1.29E+08	0.00109
27	oar3_OAR〉	1.29E+08	-0.02275
27	oar3_OAR〉	1.29E+08	-0.00926
27	oar3_OAR〉	1.29E+08	-0.03141
27	oar3_OAR〉	1.29E+08	-0.00819

27	oar3_OAR〉	1.29E+08	-0.02744
27	oar3_OAR〉	1.29E+08	-0.02744
27	oar3_OAR〉	1.29E+08	-0.01655
27	oar3_OAR〉	1.29E+08	-0.01655
27	oar3_OAR〉	1.29E+08	-0.01994
27	oar3_OAR〉	1.29E+08	0.004671
27	oar3_OAR〉	1.29E+08	0.004671
27	oar3_OAR〉	1.29E+08	-0.01699
27	oar3_OAR〉	1.29E+08	-0.03709
27	oar3_OAR〉	1.29E+08	-0.01069
27	oar3_OAR〉	1.29E+08	-0.01251
27	oar3_OAR〉	1.29E+08	-0.02694
27	oar3_OAR〉	1.29E+08	-0.03458
27	oar3_OAR〉	1.29E+08	-0.03641
27	oar3_OAR〉	1.29E+08	-0.03641
27	oar3_OAR〉	1.29E+08	0.011808
27	oar3_OAR〉	1.3E+08	-0.03893
27	oar3_OAR〉	1.3E+08	0.063627
27	oar3_OAR〉	1.3E+08	0.007683
27	oar3_OAR〉	1.3E+08	0.012476
27	oar3_OAR〉	1.3E+08	0.000363
27	oar3_OAR〉	1.3E+08	-0.0293
27	oar3_OAR〉	1.3E+08	-0.00382
27	oar3_OAR〉	1.3E+08	0.027934
27	oar3_OAR〉	1.3E+08	-0.02182
27	oar3_OAR〉	1.3E+08	-0.03375
27	oar3_OAR〉	1.3E+08	0.032597
27	oar3_OAR〉	1.3E+08	0.01457
27	oar3_OAR〉	1.3E+08	0.032597
27	oar3_OAR〉	1.3E+08	-0.02013
27	oar3_OAR〉	1.3E+08	0.00365
27	oar3_OAR〉	1.3E+08	0.117742
27	oar3_OAR〉	1.3E+08	0.032101
27	oar3_OAR〉	1.3E+08	0.01457
27	oar3_OAR〉	1.3E+08	-0.0119
27	oar3_OAR〉	1.3E+08	0.023798
27	oar3_OAR〉	1.3E+08	0.089766
27	oar3_OAR〉	1.3E+08	0.036036
27	oar3_OAR〉	1.3E+08	0.033636
27	oar3_OAR〉	1.3E+08	0.02805
27	oar3_OAR〉	1.3E+08	-0.02533
27	oar3_OAR〉	1.3E+08	0.075034
27	oar3_OAR〉	1.3E+08	-0.01209
27	oar3_OAR〉	1.3E+08	-0.01209
27	oar3_OAR〉	1.3E+08	0.021955
27	oar3_OAR〉	1.3E+08	-0.00052
27	oar3_OAR〉	1.3E+08	0.006106
27	oar3_OAR〉	1.3E+08	0.008378
27	oar3_OAR〉	1.3E+08	-0.02053
27	oar3_OAR〉	1.3E+08	0.006106
27	oar3_OAR〉	1.3E+08	0.008378
27	oar3_OAR〉	1.3E+08	-0.00036
27	oar3_OAR〉	1.3E+08	0.059098
27	oar3_OAR〉	1.3E+08	0.000134

27	oar3_OAR›	1.3E+08	-0.00224
27	oar3_OAR›	1.3E+08	-0.01964
27	oar3_OAR›	1.3E+08	-0.02156
27	oar3_OAR›	1.3E+08	-0.02156
27	oar3_OAR›	1.3E+08	0.002898
27	oar3_OAR›	1.3E+08	0.007082
27	oar3_OAR›	1.3E+08	-0.00768
27	oar3_OAR›	1.3E+08	0.059918
27	oar3_OAR›	1.3E+08	-0.01035
27	oar3_OAR›	1.3E+08	-0.01757
27	oar3_OAR›	1.3E+08	-0.01132
27	oar3_OAR›	1.3E+08	0.056789
27	oar3_OAR›	1.3E+08	-0.00866
27	oar3_OAR›	1.3E+08	-0.02834
27	oar3_OAR›	1.3E+08	0.034553
27	oar3_OAR›	1.3E+08	-0.00187
27	oar3_OAR›	1.3E+08	-0.01069
27	oar3_OAR›	1.3E+08	-0.03011
27	oar3_OAR›	1.3E+08	-0.0214
27	oar3_OAR›	1.3E+08	-0.02189
27	oar3_OAR›	1.3E+08	-0.00772
27	oar3_OAR›	1.3E+08	-0.01024
27	oar3_OAR›	1.3E+08	-0.02831
27	oar3_OAR›	1.3E+08	-0.02103
27	oar3_OAR›	1.3E+08	-0.03471
27	oar3_OAR›	1.3E+08	0.011893
27	oar3_OAR›	1.3E+08	-0.01567
27	oar3_OAR›	1.3E+08	-0.00012
27	oar3_OAR›	1.3E+08	0.075987
27	oar3_OAR›	1.3E+08	-0.00796
27	oar3_OAR›	1.3E+08	0.071634
27	oar3_OAR›	1.3E+08	0.071317
27	oar3_OAR›	1.3E+08	0.057831
27	oar3_OAR›	1.3E+08	-0.01716
27	oar3_OAR›	1.3E+08	0.008214
27	oar3_OAR›	1.3E+08	0.008214
27	oar3_OAR›	1.3E+08	0.062852
27	oar3_OAR›	1.3E+08	0.079329
27	oar3_OAR›	1.3E+08	0.030891
27	oar3_OAR›	1.3E+08	-0.00521
27	oar3_OAR›	1.3E+08	0.073534
27	oar3_OAR›	1.3E+08	0.040864
27	oar3_OAR›	1.3E+08	0.050337
27	oar3_OAR›	1.3E+08	0.077489
27	oar3_OAR›	1.3E+08	0.073534
27	oar3_OAR›	1.3E+08	0.079148
27	oar3_OAR›	1.3E+08	0.079148
27	oar3_OAR›	1.3E+08	0.105664
27	oar3_OAR›	1.3E+08	-0.02292
27	oar3_OAR›	1.3E+08	0.102107
27	oar3_OAR›	1.3E+08	-0.01787
27	oar3_OAR›	1.3E+08	0.072176
27	oar3_OAR›	1.3E+08	0.072176
27	oar3_OAR›	1.3E+08	-0.02292

27	oar3_OAR〉	1.3E+08	0.060201
27	oar3_OAR〉	1.3E+08	0.077345
27	oar3_OAR〉	1.3E+08	-0.02533
27	oar3_OAR〉	1.3E+08	0.075422
27	oar3_OAR〉	1.3E+08	-0.01851
27	oar3_OAR〉	1.3E+08	-0.02292
27	oar3_OAR〉	1.3E+08	-0.02292
27	oar3_OAR〉	1.3E+08	0.017167
27	oar3_OAR〉	1.3E+08	-0.03178
27	oar3_OAR〉	1.3E+08	-0.02398
27	oar3_OAR〉	1.3E+08	-0.01551
27	oar3_OAR〉	1.3E+08	-0.01737
27	oar3_OAR〉	1.3E+08	-0.01731
27	oar3_OAR〉	1.3E+08	0.033923
27	oar3_OAR〉	1.3E+08	0.077801
27	oar3_OAR〉	1.3E+08	-0.0168
27	oar3_OAR〉	1.3E+08	0.112717
27	oar3_OAR〉	1.3E+08	0.025847
27	oar3_OAR〉	1.3E+08	0.104169
27	oar3_OAR〉	1.3E+08	0.071077
27	oar3_OAR〉	1.3E+08	0.071077
27	oar3_OAR〉	1.3E+08	-0.02693
27	oar3_OAR〉	1.3E+08	0.13584
27	oar3_OAR〉	1.3E+08	0.063021
27	oar3_OAR〉	1.3E+08	-0.02209
27	oar3_OAR〉	1.3E+08	0.057459
27	oar3_OAR〉	1.3E+08	0.083987
27	oar3_OAR〉	1.3E+08	-0.02266
27	oar3_OAR〉	1.3E+08	-0.00524
27	oar3_OAR〉	1.3E+08	-0.01299
27	oar3_OAR〉	1.3E+08	0.044919
27	oar3_OAR〉	1.3E+08	0.053321
27	oar3_OAR〉	1.3E+08	0.00481
27	oar3_OAR〉	1.3E+08	-0.01484
27	oar3_OAR〉	1.3E+08	0.061974
27	oar3_OAR〉	1.3E+08	0.031927
27	oar3_OAR〉	1.3E+08	0.006164
27	oar3_OAR〉	1.3E+08	0.006164
27	oar3_OAR〉	1.3E+08	-0.01915
27	oar3_OAR〉	1.3E+08	-0.01915
27	oar3_OAR〉	1.3E+08	0.049315
27	oar3_OAR〉	1.3E+08	0.01496
27	oar3_OAR〉	1.3E+08	0.086036
27	oar3_OAR〉	1.3E+08	0.052462
27	oar3_OAR〉	1.3E+08	-0.03099
27	oar3_OAR〉	1.3E+08	0.027445
27	oar3_OAR〉	1.3E+08	0.073898
27	oar3_OAR〉	1.3E+08	-0.02847
27	oar3_OAR〉	1.3E+08	-0.0054
27	oar3_OAR〉	1.3E+08	0.078761
27	oar3_OAR〉	1.3E+08	0.029308
27	oar3_OAR〉	1.3E+08	0.02172
27	oar3_OAR〉	1.3E+08	0.010712
27	oar3_OAR〉	1.3E+08	0.048267

27	oar3_OAR〉	1.3E+08	-0.00422
27	oar3_OAR〉	1.3E+08	0.003891
27	oar3_OAR〉	1.3E+08	0.006548
27	oar3_OAR〉	1.3E+08	0.006548
27	oar3_OAR〉	1.3E+08	0.010745
27	oar3_OAR〉	1.3E+08	0.006548
27	oar3_OAR〉	1.3E+08	-0.0041
27	oar3_OAR〉	1.3E+08	0.011116
27	oar3_OAR〉	1.3E+08	0.002754
27	oar3_OAR〉	1.3E+08	-0.01298
27	oar3_OAR〉	1.3E+08	0.009937
27	oar3_OAR〉	1.3E+08	-0.01486
27	oar3_OAR〉	1.3E+08	-0.02961
27	oar3_OAR〉	1.3E+08	0.008988
27	oar3_OAR〉	1.3E+08	-0.01731
27	oar3_OAR〉	1.3E+08	-0.01717
27	oar3_OAR〉	1.3E+08	-0.01717
27	oar3_OAR〉	1.3E+08	-0.01655
27	oar3_OAR〉	1.3E+08	-0.0037
27	oar3_OAR〉	1.3E+08	-0.00729
27	oar3_OAR〉	1.3E+08	-0.01091
27	oar3_OAR〉	1.3E+08	-0.03047
27	oar3_OAR〉	1.3E+08	-0.01731
27	oar3_OAR〉	1.3E+08	-0.03234
27	oar3_OAR〉	1.3E+08	-0.00361
27	oar3_OAR〉	1.3E+08	-0.00043
27	oar3_OAR〉	1.3E+08	-0.02732
27	oar3_OAR〉	1.3E+08	-0.01764
27	oar3_OAR〉	1.3E+08	-0.00117
27	oar3_OAR〉	1.3E+08	-0.01338
27	oar3_OAR〉	1.3E+08	-0.02058
27	oar3_OAR〉	1.3E+08	-0.02411
27	oar3_OAR〉	1.3E+08	-0.00882
27	oar3_OAR〉	1.3E+08	-0.0108
27	oar3_OAR〉	1.3E+08	0.005432
27	oar3_OAR〉	1.3E+08	0.049642
27	oar3_OAR〉	1.3E+08	-0.00399
27	oar3_OAR〉	1.3E+08	0.003214
27	oar3_OAR〉	1.3E+08	0.030802
27	oar3_OAR〉	1.3E+08	0.004547
27	oar3_OAR〉	1.3E+08	-0.00906
27	oar3_OAR〉	1.3E+08	0.030755
27	oar3_OAR〉	1.3E+08	0.06935
27	oar3_OAR〉	1.3E+08	0.088226
27	oar3_OAR〉	1.3E+08	0.052515
27	oar3_OAR〉	1.3E+08	0.026509
27	oar3_OAR〉	1.3E+08	0.059942
27	oar3_OAR〉	1.3E+08	0.103251
27	oar3_OAR〉	1.3E+08	0.033261
27	oar3_OAR〉	1.3E+08	0.033261
27	oar3_OAR〉	1.3E+08	0.067006
27	oar3_OAR〉	1.3E+08	0.037782
27	oar3_OAR〉	1.3E+08	0.088457
27	oar3_OAR〉	1.3E+08	0.088457

27	oar3_OAR〉	1.3E+08	0.037782
27	oar3_OAR〉	1.3E+08	0.129717
27	oar3_OAR〉	1.3E+08	-0.02611
27	oar3_OAR〉	1.3E+08	0.019309
27	oar3_OAR〉	1.3E+08	-0.01462
27	oar3_OAR〉	1.3E+08	0.031942
27	oar3_OAR〉	1.3E+08	0.008635
27	oar3_OAR〉	1.3E+08	-0.00439
27	oar3_OAR〉	1.3E+08	0.081026
27	oar3_OAR〉	1.3E+08	0.024211
27	oar3_OAR〉	1.3E+08	0.10913
27	oar3_OAR〉	1.3E+08	0.024211
27	oar3_OAR〉	1.3E+08	0.044724
27	oar3_OAR〉	1.3E+08	-0.00439
27	oar3_OAR〉	1.3E+08	-0.00183
27	oar3_OAR〉	1.3E+08	-0.00844
27	oar3_OAR〉	1.3E+08	-0.00439
27	oar3_OAR〉	1.3E+08	0.025238
27	oar3_OAR〉	1.3E+08	0.025238
27	oar3_OAR〉	1.3E+08	-0.01805
27	oar3_OAR〉	1.3E+08	0.021372
27	oar3_OAR〉	1.3E+08	-0.00127
27	oar3_OAR〉	1.3E+08	-0.01031
27	oar3_OAR〉	1.3E+08	-0.00195
27	oar3_OAR〉	1.3E+08	-0.02462
27	oar3_OAR〉	1.3E+08	-0.00195
27	oar3_OAR〉	1.3E+08	-0.01753
27	oar3_OAR〉	1.3E+08	-0.02831
27	oar3_OAR〉	1.3E+08	-0.01031
27	oar3_OAR〉	1.3E+08	-0.01139
27	oar3_OAR〉	1.3E+08	-0.01869
27	oar3_OAR〉	1.3E+08	0.009888
27	oar3_OAR〉	1.3E+08	0.009888
27	oar3_OAR〉	1.3E+08	0.030925
27	oar3_OAR〉	1.3E+08	-0.00022
27	oar3_OAR〉	1.3E+08	-0.03451
27	oar3_OAR〉	1.31E+08	-0.03675
27	oar3_OAR〉	1.31E+08	0.000363
27	oar3_OAR〉	1.31E+08	0.040966
27	oar3_OAR〉	1.31E+08	-0.02184
27	oar3_OAR〉	1.31E+08	0.038185
27	oar3_OAR〉	1.31E+08	-0.00982
27	oar3_OAR〉	1.31E+08	0.000363
27	oar3_OAR〉	1.31E+08	-0.02184
27	oar3_OAR〉	1.31E+08	0.000363
27	oar3_OAR〉	1.31E+08	-0.02184
27	oar3_OAR〉	1.31E+08	-0.00611
27	oar3_OAR〉	1.31E+08	-0.00673
27	oar3_OAR〉	1.31E+08	-0.00583
27	oar3_OAR〉	1.31E+08	-0.00575
27	oar3_OAR〉	1.31E+08	0.05008
27	oar3_OAR〉	1.31E+08	0.043609
27	oar3_OAR〉	1.31E+08	0.053387
27	oar3_OAR〉	1.31E+08	-0.00575

27	oar3_OAR⟩	1.31E+08	0.052265
27	oar3_OAR⟩	1.31E+08	0.057073
27	oar3_OAR⟩	1.31E+08	-0.01414
27	oar3_OAR⟩	1.31E+08	-0.01735
27	oar3_OAR⟩	1.31E+08	-0.01414
27	oar3_OAR⟩	1.31E+08	-0.00651
27	oar3_OAR⟩	1.31E+08	-0.01911
27	oar3_OAR⟩	1.31E+08	0.046662
27	oar3_OAR⟩	1.31E+08	0.024987
27	oar3_OAR⟩	1.31E+08	-0.03513
27	oar3_OAR⟩	1.31E+08	-0.00771
27	oar3_OAR⟩	1.31E+08	0.00088
27	oar3_OAR⟩	1.31E+08	-0.01199
27	oar3_OAR⟩	1.31E+08	-0.00462
27	oar3_OAR⟩	1.31E+08	-0.01573
27	oar3_OAR⟩	1.31E+08	0.00115
27	oar3_OAR⟩	1.31E+08	-0.02973
27	oar3_OAR⟩	1.31E+08	-0.01916
27	oar3_OAR⟩	1.31E+08	0.04591
27	oar3_OAR⟩	1.31E+08	0.014098
27	oar3_OAR⟩	1.31E+08	0.051229
27	oar3_OAR⟩	1.31E+08	0.082222
27	oar3_OAR⟩	1.31E+08	-0.00901
27	oar3_OAR⟩	1.31E+08	-0.02078
27	oar3_OAR⟩	1.31E+08	-0.02642
27	oar3_OAR⟩	1.31E+08	-0.02642
27	oar3_OAR⟩	1.31E+08	0.028277
27	oar3_OAR⟩	1.31E+08	0.028277
27	oar3_OAR⟩	1.31E+08	0.038755
27	oar3_OAR⟩	1.31E+08	0.023951
27	oar3_OAR⟩	1.31E+08	-0.00874
27	oar3_OAR⟩	1.31E+08	0.023951
27	oar3_OAR⟩	1.31E+08	0.044847
27	oar3_OAR⟩	1.31E+08	-0.03171
27	oar3_OAR⟩	1.31E+08	-0.0245
27	oar3_OAR⟩	1.31E+08	0.001554
27	oar3_OAR⟩	1.31E+08	0.076445
27	oar3_OAR⟩	1.31E+08	0.033157
27	oar3_OAR⟩	1.31E+08	0.004532
27	oar3_OAR⟩	1.31E+08	0.017146
27	oar3_OAR⟩	1.31E+08	-0.00174
27	oar3_OAR⟩	1.31E+08	-0.01916
27	oar3_OAR⟩	1.31E+08	-0.025
27	oar3_OAR⟩	1.31E+08	-0.00174
27	oar3_OAR⟩	1.31E+08	-0.02647
27	oar3_OAR⟩	1.31E+08	-0.0006
27	oar3_OAR⟩	1.31E+08	-0.01596
27	oar3_OAR⟩	1.31E+08	0.036972
27	oar3_OAR⟩	1.31E+08	-0.0006
27	oar3_OAR⟩	1.31E+08	-0.02101
27	oar3_OAR⟩	1.31E+08	-0.02444
27	oar3_OAR⟩	1.31E+08	-0.00788
27	oar3_OAR⟩	1.31E+08	-0.00137
27	oar3_OAR⟩	1.31E+08	0.07052

27	oar3_OAR〉	1.31E+08	-0.02523
27	oar3_OAR〉	1.31E+08	-0.03632
27	oar3_OAR〉	1.31E+08	-0.03014
27	oar3_OAR〉	1.31E+08	-0.02328
27	oar3_OAR〉	1.31E+08	-0.03858
27	oar3_OAR〉	1.31E+08	4.31E-05
27	oar3_OAR〉	1.31E+08	-0.00169
27	oar3_OAR〉	1.31E+08	-0.01133
27	oar3_OAR〉	1.31E+08	-0.02049
27	oar3_OAR〉	1.31E+08	-0.00968
27	oar3_OAR〉	1.31E+08	-0.02645
27	oar3_OAR〉	1.31E+08	-0.01107
27	oar3_OAR〉	1.31E+08	-0.00169
27	oar3_OAR〉	1.31E+08	-0.03573
27	oar3_OAR〉	1.31E+08	0.00365
27	oar3_OAR〉	1.31E+08	0.000703
27	oar3_OAR〉	1.31E+08	-0.03738
27	oar3_OAR〉	1.31E+08	0.000703
27	oar3_OAR〉	1.31E+08	0.00365
27	oar3_OAR〉	1.31E+08	0.003106
27	oar3_OAR〉	1.31E+08	-0.02217
27	oar3_OAR〉	1.31E+08	-0.01837
27	oar3_OAR〉	1.31E+08	-0.01837
27	oar3_OAR〉	1.31E+08	0.009825
27	oar3_OAR〉	1.31E+08	0.103097
27	oar3_OAR〉	1.31E+08	0.020267
27	oar3_OAR〉	1.31E+08	0.048391
27	oar3_OAR〉	1.31E+08	-0.00864
27	oar3_OAR〉	1.31E+08	0.069787
27	oar3_OAR〉	1.31E+08	-0.00941
27	oar3_OAR〉	1.31E+08	0.036114
27	oar3_OAR〉	1.31E+08	0.075097
27	oar3_OAR〉	1.31E+08	-0.02596
27	oar3_OAR〉	1.31E+08	0.060911
27	oar3_OAR〉	1.31E+08	0.034159
27	oar3_OAR〉	1.31E+08	0.062418
27	oar3_OAR〉	1.31E+08	-0.00653
27	oar3_OAR〉	1.31E+08	0.062418
27	oar3_OAR〉	1.31E+08	0.031966
27	oar3_OAR〉	1.31E+08	0.062418
27	oar3_OAR〉	1.31E+08	0.057432
27	oar3_OAR〉	1.31E+08	-0.02013
27	oar3_OAR〉	1.31E+08	0.044562
27	oar3_OAR〉	1.31E+08	0.02501
27	oar3_OAR〉	1.31E+08	0.006682
27	oar3_OAR〉	1.31E+08	0.024925
27	oar3_OAR〉	1.31E+08	0.010029
27	oar3_OAR〉	1.31E+08	0.120316
27	oar3_OAR〉	1.31E+08	0.155383
27	oar3_OAR〉	1.31E+08	0.069502
27	oar3_OAR〉	1.31E+08	-0.01905
27	oar3_OAR〉	1.31E+08	0.103207
27	oar3_OAR〉	1.31E+08	0.103207
27	oar3_OAR〉	1.31E+08	0.043722

27	oar3_OAR〉	1.31E+08	0.052442
27	oar3_OAR〉	1.31E+08	0.039481
27	oar3_OAR〉	1.31E+08	0.09322
27	oar3_OAR〉	1.31E+08	-0.00341
27	oar3_OAR〉	1.31E+08	0.029026
27	oar3_OAR〉	1.31E+08	-0.02517
27	oar3_OAR〉	1.31E+08	0.007039
27	oar3_OAR〉	1.31E+08	0.013414
27	oar3_OAR〉	1.31E+08	0.079924
27	oar3_OAR〉	1.31E+08	0.014275
27	oar3_OAR〉	1.31E+08	0.074647
27	oar3_OAR〉	1.31E+08	0.074647
27	oar3_OAR〉	1.31E+08	-0.00068
27	oar3_OAR〉	1.31E+08	-0.02576
27	oar3_OAR〉	1.31E+08	-0.02786
27	oar3_OAR〉	1.31E+08	-0.01915
27	oar3_OAR〉	1.31E+08	-0.02819
27	oar3_OAR〉	1.31E+08	0.064529
27	oar3_OAR〉	1.31E+08	0.038847
27	oar3_OAR〉	1.31E+08	-0.02228
27	oar3_OAR〉	1.31E+08	-0.01915
27	oar3_OAR〉	1.31E+08	0.002308
27	oar3_OAR〉	1.31E+08	0.043437
27	oar3_OAR〉	1.31E+08	0.023403
27	oar3_OAR〉	1.31E+08	0.010985
27	oar3_OAR〉	1.31E+08	-0.02338
27	oar3_OAR〉	1.31E+08	0.007045
27	oar3_OAR〉	1.31E+08	0.007045
27	oar3_OAR〉	1.31E+08	-0.02661
27	oar3_OAR〉	1.31E+08	0.011328
27	oar3_OAR〉	1.31E+08	7.25E-05
27	oar3_OAR〉	1.31E+08	0.104324
27	oar3_OAR〉	1.31E+08	0.074314
27	oar3_OAR〉	1.31E+08	-0.03056
27	oar3_OAR〉	1.31E+08	0.087569
27	oar3_OAR〉	1.31E+08	-0.02453
27	oar3_OAR〉	1.31E+08	0.094221
27	oar3_OAR〉	1.31E+08	-0.00788
27	oar3_OAR〉	1.31E+08	0.065201
27	oar3_OAR〉	1.31E+08	0.020533
27	oar3_OAR〉	1.31E+08	0.235607
27	oar3_OAR〉	1.31E+08	0.08915
27	oar3_OAR〉	1.31E+08	-0.00589
27	oar3_OAR〉	1.31E+08	0.200268
27	oar3_OAR〉	1.31E+08	-0.01153
27	oar3_OAR〉	1.31E+08	-0.0108
27	oar3_OAR〉	1.31E+08	0.028049
27	oar3_OAR〉	1.31E+08	0.003654
27	oar3_OAR〉	1.31E+08	0.106997
27	oar3_OAR〉	1.31E+08	-0.00603
27	oar3_OAR〉	1.31E+08	0.043995
27	oar3_OAR〉	1.31E+08	-0.01902
27	oar3_OAR〉	1.31E+08	-0.01902
27	oar3_OAR〉	1.31E+08	0.036193

27	oar3_OAR›	1.31E+08	-0.01423
27	oar3_OAR›	1.31E+08	0.045946
27	oar3_OAR›	1.31E+08	-0.01902
27	oar3_OAR›	1.31E+08	-0.011
27	oar3_OAR›	1.31E+08	0.036193
27	oar3_OAR›	1.31E+08	0.008723
27	oar3_OAR›	1.31E+08	-0.02565
27	oar3_OAR›	1.31E+08	-0.01423
27	oar3_OAR›	1.31E+08	-0.01338
27	oar3_OAR›	1.31E+08	-0.02369
27	oar3_OAR›	1.31E+08	-0.02369
27	oar3_OAR›	1.31E+08	-0.01525
27	oar3_OAR›	1.31E+08	0.047136
27	oar3_OAR›	1.31E+08	0.003659
27	oar3_OAR›	1.31E+08	-0.036
27	oar3_OAR›	1.31E+08	-0.0243
27	oar3_OAR›	1.31E+08	0.042047
27	oar3_OAR›	1.31E+08	-0.02182
27	oar3_OAR›	1.31E+08	0.00222
27	oar3_OAR›	1.31E+08	0.093614
27	oar3_OAR›	1.31E+08	0.039958
27	oar3_OAR›	1.31E+08	0.040066
27	oar3_OAR›	1.31E+08	0.099163
27	oar3_OAR›	1.31E+08	0.053445
27	oar3_OAR›	1.31E+08	-0.01031
27	oar3_OAR›	1.31E+08	0.052033
27	oar3_OAR›	1.31E+08	0.044645
27	oar3_OAR›	1.31E+08	0.032402
27	oar3_OAR›	1.31E+08	-0.01031
27	oar3_OAR›	1.32E+08	-0.02449
27	oar3_OAR›	1.32E+08	-0.0014
27	oar3_OAR›	1.32E+08	-0.00935
27	oar3_OAR›	1.32E+08	0.046234
27	oar3_OAR›	1.32E+08	0.064784
27	oar3_OAR›	1.32E+08	0.058691
27	oar3_OAR›	1.32E+08	0.026771
27	oar3_OAR›	1.32E+08	0.046917
27	oar3_OAR›	1.32E+08	0.046917
27	oar3_OAR›	1.32E+08	-0.02184
27	oar3_OAR›	1.32E+08	0.058691
27	oar3_OAR›	1.32E+08	0.041833
27	oar3_OAR›	1.32E+08	0.043661
27	oar3_OAR›	1.32E+08	0.015421
27	oar3_OAR›	1.32E+08	-0.02899
27	oar3_OAR›	1.32E+08	-0.03134
27	oar3_OAR›	1.32E+08	0.018271
27	oar3_OAR›	1.32E+08	-0.03633
27	oar3_OAR›	1.32E+08	0.013429
27	oar3_OAR›	1.32E+08	0.002291
27	oar3_OAR›	1.32E+08	0.06397
27	oar3_OAR›	1.32E+08	-0.02732
27	oar3_OAR›	1.32E+08	-0.0321
27	oar3_OAR›	1.32E+08	-0.0321
27	oar3_OAR›	1.32E+08	-0.00677

27	oar3_OAR〉	1.32E+08	0.005851
27	oar3_OAR〉	1.32E+08	0.004272
27	oar3_OAR〉	1.32E+08	-0.02077
27	oar3_OAR〉	1.32E+08	-0.02961
27	oar3_OAR〉	1.32E+08	0.001405
27	oar3_OAR〉	1.32E+08	0.065346
27	oar3_OAR〉	1.32E+08	-0.01612
27	oar3_OAR〉	1.32E+08	-0.00583
27	oar3_OAR〉	1.32E+08	-0.00199
27	oar3_OAR〉	1.32E+08	-0.02925
27	oar3_OAR〉	1.32E+08	-0.02817
27	oar3_OAR〉	1.32E+08	-0.02719
27	oar3_OAR〉	1.32E+08	-0.03157
27	oar3_OAR〉	1.32E+08	-0.03298
27	oar3_OAR〉	1.32E+08	-0.02648
27	oar3_OAR〉	1.32E+08	-0.02396
27	oar3_OAR〉	1.32E+08	-0.03157
27	oar3_OAR〉	1.32E+08	-0.02115
27	oar3_OAR〉	1.32E+08	0.025477
27	oar3_OAR〉	1.32E+08	-0.02191
27	oar3_OAR〉	1.32E+08	0.086051
27	oar3_OAR〉	1.32E+08	-0.02064
27	oar3_OAR〉	1.32E+08	-0.01192
27	oar3_OAR〉	1.32E+08	-0.00738
27	oar3_OAR〉	1.32E+08	-0.01815
27	oar3_OAR〉	1.32E+08	-0.01121
27	oar3_OAR〉	1.32E+08	-0.0263
27	oar3_OAR〉	1.32E+08	0.013793
27	oar3_OAR〉	1.32E+08	-0.0263
27	oar3_OAR〉	1.32E+08	-0.00606
27	oar3_OAR〉	1.32E+08	-0.02984
27	oar3_OAR〉	1.32E+08	0.032181
27	oar3_OAR〉	1.32E+08	0.012436
27	oar3_OAR〉	1.32E+08	0.065544
27	oar3_OAR〉	1.32E+08	0.012436
27	oar3_OAR〉	1.32E+08	0.065544
27	oar3_OAR〉	1.32E+08	-0.02043
27	oar3_OAR〉	1.32E+08	-0.00152
27	oar3_OAR〉	1.32E+08	0.008398
27	oar3_OAR〉	1.32E+08	0.01418
27	oar3_OAR〉	1.32E+08	-0.02645
27	oar3_OAR〉	1.32E+08	-0.01379
27	oar3_OAR〉	1.32E+08	-0.01174
27	oar3_OAR〉	1.32E+08	-0.00152
27	oar3_OAR〉	1.32E+08	-0.00043
27	oar3_OAR〉	1.32E+08	-0.00083
27	oar3_OAR〉	1.32E+08	-0.0106
27	oar3_OAR〉	1.32E+08	########
27	oar3_OAR〉	1.32E+08	-0.00152
27	oar3_OAR〉	1.32E+08	-0.00083
27	oar3_OAR〉	1.32E+08	-0.00043
27	oar3_OAR〉	1.32E+08	-0.01174
27	oar3_OAR〉	1.32E+08	-0.01033
27	oar3_OAR〉	1.32E+08	0.017159

27	oar3_OAR›	1.32E+08	-0.01033
27	oar3_OAR›	1.32E+08	-0.0284
27	oar3_OAR›	1.32E+08	0.017159
27	oar3_OAR›	1.32E+08	0.025555
27	oar3_OAR›	1.32E+08	-0.00982
27	oar3_OAR›	1.32E+08	-0.01126
27	oar3_OAR›	1.32E+08	0.020885
27	oar3_OAR›	1.32E+08	0.025555
27	oar3_OAR›	1.32E+08	-0.02627
27	oar3_OAR›	1.32E+08	0.002694
27	oar3_OAR›	1.32E+08	-0.01126
27	oar3_OAR›	1.32E+08	0.009692
27	oar3_OAR›	1.32E+08	-0.02216
27	oar3_OAR›	1.32E+08	0.009692
27	oar3_OAR›	1.32E+08	-0.02216
27	oar3_OAR›	1.32E+08	-0.02375
27	oar3_OAR›	1.32E+08	-0.03012
27	oar3_OAR›	1.32E+08	-0.02216
27	oar3_OAR›	1.32E+08	0.030609
27	oar3_OAR›	1.32E+08	0.009692
27	oar3_OAR›	1.32E+08	-0.02375
27	oar3_OAR›	1.32E+08	0.031119
27	oar3_OAR›	1.32E+08	-0.02375
27	oar3_OAR›	1.32E+08	-0.02113
27	oar3_OAR›	1.32E+08	-0.02113
27	oar3_OAR›	1.32E+08	-0.02209
27	oar3_OAR›	1.32E+08	-0.03745
27	oar3_OAR›	1.32E+08	-0.03565
27	oar3_OAR›	1.32E+08	0.000877
27	oar3_OAR›	1.32E+08	-0.02199
27	oar3_OAR›	1.32E+08	0.031119
27	oar3_OAR›	1.32E+08	-0.03569
27	oar3_OAR›	1.32E+08	-0.02209
27	oar3_OAR›	1.32E+08	-0.03195
27	oar3_OAR›	1.32E+08	-0.03787
27	oar3_OAR›	1.32E+08	0.065575
27	oar3_OAR›	1.32E+08	0.029694
27	oar3_OAR›	1.32E+08	0.011058
27	oar3_OAR›	1.32E+08	0.031803
27	oar3_OAR›	1.32E+08	0.029694
27	oar3_OAR›	1.32E+08	-0.01956
27	oar3_OAR›	1.32E+08	0.065575
27	oar3_OAR›	1.32E+08	0.000214
27	oar3_OAR›	1.32E+08	-0.01956
27	oar3_OAR›	1.32E+08	-0.01956
27	oar3_OAR›	1.32E+08	0.029694
27	oar3_OAR›	1.32E+08	-0.02699
27	oar3_OAR›	1.32E+08	-0.01956
27	oar3_OAR›	1.32E+08	0.032281
27	oar3_OAR›	1.32E+08	0.032281
27	oar3_OAR›	1.32E+08	0.004072
27	oar3_OAR›	1.32E+08	0.065575
27	oar3_OAR›	1.32E+08	-0.01956
27	oar3_OAR›	1.32E+08	-0.00455

27	oar3_OAR〉	1.32E+08	0.065575
27	oar3_OAR〉	1.32E+08	-0.03432
27	oar3_OAR〉	1.32E+08	-0.01788
27	oar3_OAR〉	1.32E+08	-0.00979
27	oar3_OAR〉	1.32E+08	-0.01979
27	oar3_OAR〉	1.32E+08	-0.00979
27	oar3_OAR〉	1.32E+08	-0.02347
27	oar3_OAR〉	1.32E+08	0.065575
27	oar3_OAR〉	1.32E+08	0.065575
27	oar3_OAR〉	1.32E+08	-0.02347
27	oar3_OAR〉	1.32E+08	-0.02347
27	oar3_OAR〉	1.32E+08	-0.02347
27	oar3_OAR〉	1.32E+08	0.00986
27	oar3_OAR〉	1.32E+08	-0.02306
27	oar3_OAR〉	1.32E+08	0.048209
27	oar3_OAR〉	1.32E+08	-0.02347
27	oar3_OAR〉	1.33E+08	0.065575
27	oar3_OAR〉	1.33E+08	0.065575
27	oar3_OAR〉	1.33E+08	0.007994
27	oar3_OAR〉	1.33E+08	-0.02419
27	oar3_OAR〉	1.33E+08	-0.02419
27	oar3_OAR〉	1.33E+08	-0.02419
27	oar3_OAR〉	1.33E+08	0.021381
27	oar3_OAR〉	1.33E+08	-0.00882
27	oar3_OAR〉	1.33E+08	0.065575
27	oar3_OAR〉	1.33E+08	0.021381
27	oar3_OAR〉	1.33E+08	-0.02419
27	oar3_OAR〉	1.33E+08	-0.01969
27	oar3_OAR〉	1.33E+08	0.023934
27	oar3_OAR〉	1.33E+08	0.039326
27	oar3_OAR〉	1.33E+08	0.065575
27	oar3_OAR〉	1.33E+08	-0.03054
27	oar3_OAR〉	1.33E+08	-0.02688
27	oar3_OAR〉	1.33E+08	0.021381
27	oar3_OAR〉	1.33E+08	-0.02419
27	oar3_OAR〉	1.33E+08	0.088043
27	oar3_OAR〉	1.33E+08	1.82E-05
27	oar3_OAR〉	1.33E+08	-0.02265
27	oar3_OAR〉	1.33E+08	-0.02265
27	oar3_OAR〉	1.33E+08	-0.01822
27	oar3_OAR〉	1.33E+08	0.04616
27	oar3_OAR〉	1.33E+08	-0.0085
27	oar3_OAR〉	1.33E+08	0.032582
27	oar3_OAR〉	1.33E+08	0.046525
27	oar3_OAR〉	1.33E+08	-0.0085
27	oar3_OAR〉	1.33E+08	0.032582
27	oar3_OAR〉	1.33E+08	-0.00673
27	oar3_OAR〉	1.33E+08	0.001274
27	oar3_OAR〉	1.33E+08	-0.00673
27	oar3_OAR〉	1.33E+08	0.131175
27	oar3_OAR〉	1.33E+08	0.033114
27	oar3_OAR〉	1.33E+08	-0.00673
27	oar3_OAR〉	1.33E+08	-0.02158
27	oar3_OAR〉	1.33E+08	0.001274

27	oar3_OAR›	1.33E+08	-0.01512
27	oar3_OAR›	1.33E+08	0.08349
27	oar3_OAR›	1.33E+08	-0.00403
27	oar3_OAR›	1.33E+08	-0.01616
27	oar3_OAR›	1.33E+08	0.041494
27	oar3_OAR›	1.33E+08	0.041494
27	oar3_OAR›	1.33E+08	-0.0286
27	oar3_OAR›	1.33E+08	0.236177
27	oar3_OAR›	1.33E+08	0.239788
27	oar3_OAR›	1.33E+08	0.043051
27	oar3_OAR›	1.33E+08	0.240789
27	oar3_OAR›	1.33E+08	-0.02545
27	oar3_OAR›	1.33E+08	0.090562
27	oar3_OAR›	1.33E+08	0.240789
27	oar3_OAR›	1.33E+08	-0.02545
27	oar3_OAR›	1.33E+08	0.229726
27	oar3_OAR›	1.33E+08	0.027749
27	oar3_OAR›	1.33E+08	0.236177
27	oar3_OAR›	1.33E+08	0.364052
27	oar3_OAR›	1.33E+08	0.236177
27	oar3_OAR›	1.33E+08	0.23691
27	oar3_OAR›	1.33E+08	0.23691
27	oar3_OAR›	1.33E+08	0.090911
27	oar3_OAR›	1.33E+08	0.23691
27	oar3_OAR›	1.33E+08	0.168523
27	oar3_OAR›	1.33E+08	0.090911
27	oar3_OAR›	1.33E+08	0.053561
27	oar3_OAR›	1.33E+08	-0.02609
27	oar3_OAR›	1.33E+08	0.080498
27	oar3_OAR›	1.33E+08	0.10375
27	oar3_OAR›	1.33E+08	0.112681
27	oar3_OAR›	1.33E+08	0.111821
27	oar3_OAR›	1.33E+08	0.112681
27	oar3_OAR›	1.33E+08	-0.02051
27	oar3_OAR›	1.33E+08	0.055768
27	oar3_OAR›	1.33E+08	0.055768
27	oar3_OAR›	1.33E+08	0.095048
27	oar3_OAR›	1.33E+08	0.055768
27	oar3_OAR›	1.33E+08	0.112681
27	oar3_OAR›	1.33E+08	0.203158
27	oar3_OAR›	1.33E+08	0.024934
27	oar3_OAR›	1.33E+08	0.173206
27	oar3_OAR›	1.33E+08	0.032461
27	oar3_OAR›	1.33E+08	0.032125
27	oar3_OAR›	1.33E+08	7.84E-05
27	oar3_OAR›	1.33E+08	-0.0042
27	oar3_OAR›	1.33E+08	7.84E-05
27	oar3_OAR›	1.33E+08	0.00406
27	oar3_OAR›	1.33E+08	-0.01735
27	oar3_OAR›	1.33E+08	0.063899
27	oar3_OAR›	1.33E+08	0.000577
27	oar3_OAR›	1.33E+08	-0.02593
27	oar3_OAR›	1.33E+08	0.000605
27	oar3_OAR›	1.33E+08	0.00263

27	oar3_OAR〉	1.33E+08	-0.01625
27	oar3_OAR〉	1.33E+08	-0.03086
27	oar3_OAR〉	1.33E+08	0.01843
27	oar3_OAR〉	1.33E+08	0.026199
27	oar3_OAR〉	1.33E+08	-0.02339
27	oar3_OAR〉	1.33E+08	-0.02471
27	oar3_OAR〉	1.33E+08	0.02829
27	oar3_OAR〉	1.33E+08	0.044736
27	oar3_OAR〉	1.33E+08	-0.01735
27	oar3_OAR〉	1.33E+08	0.057067
27	oar3_OAR〉	1.33E+08	0.02829
27	oar3_OAR〉	1.33E+08	0.043609
27	oar3_OAR〉	1.33E+08	0.062797
27	oar3_OAR〉	1.33E+08	-0.00365
27	oar3_OAR〉	1.33E+08	0.011563
27	oar3_OAR〉	1.33E+08	-0.00946
27	oar3_OAR〉	1.33E+08	-0.02964
27	oar3_OAR〉	1.33E+08	-0.00365
27	oar3_OAR〉	1.33E+08	0.044645
27	oar3_OAR〉	1.33E+08	0.043609
27	oar3_OAR〉	1.33E+08	-0.03583
27	oar3_OAR〉	1.33E+08	-0.00365
27	oar3_OAR〉	1.33E+08	-0.00365
27	oar3_OAR〉	1.33E+08	0.031225
27	oar3_OAR〉	1.33E+08	0.082464
27	oar3_OAR〉	1.33E+08	0.025054
27	oar3_OAR〉	1.33E+08	-0.00545
27	oar3_OAR〉	1.33E+08	0.036205
27	oar3_OAR〉	1.33E+08	-0.03234
27	oar3_OAR〉	1.33E+08	-0.03614
27	oar3_OAR〉	1.33E+08	-0.03477
27	oar3_OAR〉	1.33E+08	0.004711
27	oar3_OAR〉	1.33E+08	-0.02603
27	oar3_OAR〉	1.33E+08	0.039033
27	oar3_OAR〉	1.33E+08	0.039033
27	oar3_OAR〉	1.33E+08	0.010315
27	oar3_OAR〉	1.33E+08	-0.02603
27	oar3_OAR〉	1.33E+08	0.039033
27	oar3_OAR〉	1.33E+08	-0.02603
27	oar3_OAR〉	1.33E+08	0.039033
27	oar3_OAR〉	1.33E+08	0.024316
27	oar3_OAR〉	1.33E+08	0.014158
27	oar3_OAR〉	1.33E+08	0.102502
27	oar3_OAR〉	1.33E+08	-0.0156
27	oar3_OAR〉	1.33E+08	-0.01126
27	oar3_OAR〉	1.33E+08	0.083051
27	oar3_OAR〉	1.33E+08	0.025748
27	oar3_OAR〉	1.33E+08	-0.0156
27	oar3_OAR〉	1.33E+08	0.025748
27	oar3_OAR〉	1.33E+08	0.002373
27	oar3_OAR〉	1.33E+08	-0.0156
27	oar3_OAR〉	1.33E+08	0.017154
27	oar3_OAR〉	1.33E+08	0.017154
27	oar3_OAR〉	1.33E+08	0.025748

27	oar3_OAR>	1.33E+08	0.111926
27	oar3_OAR>	1.33E+08	0.081798
27	oar3_OAR>	1.33E+08	0.080171
27	oar3_OAR>	1.34E+08	0.092684
27	oar3_OAR>	1.34E+08	0.080171
27	oar3_OAR>	1.34E+08	-0.02565
27	oar3_OAR>	1.34E+08	-0.03143
27	oar3_OAR>	1.34E+08	-0.03143
27	oar3_OAR>	1.34E+08	-0.00853
27	oar3_OAR>	1.34E+08	-0.00853
27	oar3_OAR>	1.34E+08	-0.00853
27	oar3_OAR>	1.34E+08	0.008272
27	oar3_OAR>	1.34E+08	0.008272
27	oar3_OAR>	1.34E+08	0.008272
27	oar3_OAR>	1.34E+08	0.008272
27	oar3_OAR>	1.34E+08	0.020467
27	oar3_OAR>	1.34E+08	0.008272
27	oar3_OAR>	1.34E+08	0.008272
27	oar3_OAR>	1.34E+08	0.066215
27	oar3_OAR>	1.34E+08	0.008272
27	oar3_OAR>	1.34E+08	0.020467
27	oar3_OAR>	1.34E+08	0.008272
27	oar3_OAR>	1.34E+08	0.066215
27	oar3_OAR>	1.34E+08	0.008272
27	oar3_OAR>	1.34E+08	0.020467
27	oar3_OAR>	1.34E+08	0.032987
27	oar3_OAR>	1.34E+08	0.009572
27	oar3_OAR>	1.34E+08	0.016273
27	oar3_OAR>	1.34E+08	0.054341
27	oar3_OAR>	1.34E+08	-0.0147
27	oar3_OAR>	1.34E+08	-0.00358
27	oar3_OAR>	1.34E+08	0.064364
27	oar3_OAR>	1.34E+08	0.006682
27	oar3_OAR>	1.34E+08	0.013268
27	oar3_OAR>	1.34E+08	-0.01628
27	oar3_OAR>	1.34E+08	0.008296
27	oar3_OAR>	1.34E+08	0.044673
27	oar3_OAR>	1.34E+08	0.048011
27	oar3_OAR>	1.34E+08	0.048011
27	oar3_OAR>	1.34E+08	-0.00787
27	oar3_OAR>	1.34E+08	0.013268
27	oar3_OAR>	1.34E+08	0.080561
27	oar3_OAR>	1.34E+08	0.001349
27	oar3_OAR>	1.34E+08	-0.02926
27	oar3_OAR>	1.34E+08	0.042517
27	oar3_OAR>	1.34E+08	-0.01522
27	oar3_OAR>	1.34E+08	0.008296
27	oar3_OAR>	1.34E+08	0.008723
27	oar3_OAR>	1.34E+08	-0.01191
27	oar3_OAR>	1.34E+08	0.035625
27	oar3_OAR>	1.34E+08	0.035625
27	oar3_OAR>	1.34E+08	0.008296
27	oar3_OAR>	1.34E+08	0.091077
27	oar3_OAR>	1.34E+08	0.091077

27	oar3_OAR〉	1.34E+08	-0.00234
27	oar3_OAR〉	1.34E+08	0.096997
27	oar3_OAR〉	1.34E+08	0.013414
27	oar3_OAR〉	1.34E+08	-0.0052
27	oar3_OAR〉	1.34E+08	0.026075
27	oar3_OAR〉	1.34E+08	-0.01922
27	oar3_OAR〉	1.34E+08	0.04878
27	oar3_OAR〉	1.34E+08	0.003802
27	oar3_OAR〉	1.34E+08	-0.02308
27	oar3_OAR〉	1.34E+08	0.04878
27	oar3_OAR〉	1.34E+08	-0.02575
27	oar3_OAR〉	1.34E+08	0.028916
27	oar3_OAR〉	1.34E+08	-0.00776
27	oar3_OAR〉	1.34E+08	0.02374
27	oar3_OAR〉	1.34E+08	0.074647
27	oar3_OAR〉	1.34E+08	0.044724
27	oar3_OAR〉	1.34E+08	0.923967
27	oar3_OAR〉	1.34E+08	0.020399
27	oar3_OAR〉	1.34E+08	-0.01086
27	oar3_OAR〉	1.34E+08	0.059631
27	oar3_OAR〉	1.34E+08	0.074647
27	oar3_OAR〉	1.34E+08	-0.0163
27	oar3_OAR〉	1.34E+08	-0.02444
27	oar3_OAR〉	1.34E+08	-0.02444
27	oar3_OAR〉	1.34E+08	0.103251
27	oar3_OAR〉	1.34E+08	0.044724
27	oar3_OAR〉	1.34E+08	-0.00957
27	oar3_OAR〉	1.34E+08	0.003593
27	oar3_OAR〉	1.34E+08	0.00172
27	oar3_OAR〉	1.34E+08	0.000399
27	oar3_OAR〉	1.34E+08	0.014461
27	oar3_OAR〉	1.34E+08	-0.00269
27	oar3_OAR〉	1.34E+08	0.037433
27	oar3_OAR〉	1.34E+08	-0.00269
27	oar3_OAR〉	1.34E+08	0.011067
27	oar3_OAR〉	1.34E+08	0.024621
27	oar3_OAR〉	1.34E+08	0.003593
27	oar3_OAR〉	1.34E+08	0.007039
27	oar3_OAR〉	1.34E+08	0.057333
27	oar3_OAR〉	1.34E+08	0.00481
27	oar3_OAR〉	1.34E+08	0.018271
27	oar3_OAR〉	1.34E+08	-0.00065
27	oar3_OAR〉	1.34E+08	-0.02316
27	oar3_OAR〉	1.34E+08	0.040818
27	oar3_OAR〉	1.34E+08	-0.01745
27	oar3_OAR〉	1.34E+08	0.044724
27	oar3_OAR〉	1.34E+08	-0.0038
27	oar3_OAR〉	1.34E+08	-0.01834
27	oar3_OAR〉	1.34E+08	0.030065
27	oar3_OAR〉	1.34E+08	0.073272
27	oar3_OAR〉	1.34E+08	0.039481
27	oar3_OAR〉	1.34E+08	0.029789
27	oar3_OAR〉	1.34E+08	0.007497
27	oar3_OAR〉	1.34E+08	0.013016

27	oar3_OAR⟩	1.34E+08	0.007497
27	oar3_OAR⟩	1.34E+08	-0.01745
27	oar3_OAR⟩	1.34E+08	-0.00829
27	oar3_OAR⟩	1.34E+08	-0.02184
27	oar3_OAR⟩	1.34E+08	0.032582
27	oar3_OAR⟩	1.34E+08	0.012017
27	oar3_OAR⟩	1.34E+08	0.038795
27	oar3_OAR⟩	1.34E+08	-0.02597
27	oar3_OAR⟩	1.34E+08	0.100161
27	oar3_OAR⟩	1.34E+08	0.079499
27	oar3_OAR⟩	1.34E+08	-0.03679
27	oar3_OAR⟩	1.34E+08	-0.01248
27	oar3_OAR⟩	1.34E+08	0.035652
27	oar3_OAR⟩	1.34E+08	0.010604
27	oar3_OAR⟩	1.34E+08	0.149033
27	oar3_OAR⟩	1.34E+08	-0.00953
27	oar3_OAR⟩	1.34E+08	0.021037
27	oar3_OAR⟩	1.34E+08	0.009888
27	oar3_OAR⟩	1.34E+08	-0.01737
27	oar3_OAR⟩	1.34E+08	-0.00846
27	oar3_OAR⟩	1.34E+08	0.033799
27	oar3_OAR⟩	1.34E+08	0.097696
27	oar3_OAR⟩	1.34E+08	0.097696
27	oar3_OAR⟩	1.34E+08	0.007039
27	oar3_OAR⟩	1.34E+08	-0.01737
27	oar3_OAR⟩	1.34E+08	0.071155
27	oar3_OAR⟩	1.34E+08	0.079423
27	oar3_OAR⟩	1.34E+08	0.018166
27	oar3_OAR⟩	1.34E+08	-0.01954
27	oar3_OAR⟩	1.34E+08	-0.01745
27	oar3_OAR⟩	1.34E+08	0.020207
27	oar3_OAR⟩	1.34E+08	0.011067
27	oar3_OAR⟩	1.34E+08	0.005921
27	oar3_OAR⟩	1.34E+08	-0.00706
27	oar3_OAR⟩	1.34E+08	0.002133
27	oar3_OAR⟩	1.34E+08	0.025086
27	oar3_OAR⟩	1.34E+08	-0.02424
27	oar3_OAR⟩	1.34E+08	-0.00606
27	oar3_OAR⟩	1.34E+08	0.137798
27	oar3_OAR⟩	1.34E+08	0.01898
27	oar3_OAR⟩	1.34E+08	-0.02424
27	oar3_OAR⟩	1.34E+08	-0.00606
27	oar3_OAR⟩	1.34E+08	0.023589
27	oar3_OAR⟩	1.34E+08	0.137798
27	oar3_OAR⟩	1.34E+08	-0.00606
27	oar3_OAR⟩	1.34E+08	0.011067
27	oar3_OAR⟩	1.34E+08	-0.02064
27	oar3_OAR⟩	1.34E+08	-0.02064
27	oar3_OAR⟩	1.34E+08	0.064177
27	oar3_OAR⟩	1.34E+08	0.004943
27	oar3_OAR⟩	1.34E+08	-0.01934
27	oar3_OAR⟩	1.34E+08	0.022352
27	oar3_OAR⟩	1.34E+08	0.085087
27	oar3_OAR⟩	1.34E+08	0.085087

27	oar3_OAR〉	1.34E+08	0.085087
27	oar3_OAR〉	1.34E+08	0.021106
27	oar3_OAR〉	1.34E+08	0.00837
27	oar3_OAR〉	1.34E+08	-0.01263
27	oar3_OAR〉	1.34E+08	0.065146
27	oar3_OAR〉	1.35E+08	-0.02448
27	oar3_OAR〉	1.35E+08	-0.02024
27	oar3_OAR〉	1.35E+08	-0.03401
27	oar3_OAR〉	1.35E+08	-0.02448
27	oar3_OAR〉	1.35E+08	-0.03401
27	oar3_OAR〉	1.35E+08	-0.02448
27	oar3_OAR〉	1.35E+08	0.065146
27	oar3_OAR〉	1.35E+08	-0.02448
27	oar3_OAR〉	1.35E+08	-0.03111
27	oar3_OAR〉	1.35E+08	0.089452
27	oar3_OAR〉	1.35E+08	-0.00082
27	oar3_OAR〉	1.35E+08	0.089452
27	oar3_OAR〉	1.35E+08	-0.00082
27	oar3_OAR〉	1.35E+08	0.022924
27	oar3_OAR〉	1.35E+08	0.089452
27	oar3_OAR〉	1.35E+08	-0.00082
27	oar3_OAR〉	1.35E+08	0.089452
27	oar3_OAR〉	1.35E+08	0.089452
27	oar3_OAR〉	1.35E+08	-0.00082
27	oar3_OAR〉	1.35E+08	0.089452
27	oar3_OAR〉	1.35E+08	-0.02133
27	oar3_OAR〉	1.35E+08	0.003253
27	oar3_OAR〉	1.35E+08	0.003253
27	oar3_OAR〉	1.35E+08	-0.03431
27	oar3_OAR〉	1.35E+08	-0.02532
27	oar3_OAR〉	1.35E+08	0.1027
27	oar3_OAR〉	1.35E+08	0.148599
27	oar3_OAR〉	1.35E+08	0.032055
27	oar3_OAR〉	1.35E+08	0.02958
27	oar3_OAR〉	1.35E+08	0.054214
27	oar3_OAR〉	1.35E+08	0.027218
27	oar3_OAR〉	1.35E+08	0.102201
27	oar3_OAR〉	1.35E+08	0.008134
27	oar3_OAR〉	1.35E+08	0.008134
27	oar3_OAR〉	1.35E+08	-0.02788
27	oar3_OAR〉	1.35E+08	0.008134
27	oar3_OAR〉	1.35E+08	-0.02788
27	oar3_OAR〉	1.35E+08	-0.02788
27	oar3_OAR〉	1.35E+08	-0.02788
27	oar3_OAR〉	1.35E+08	-0.02788
27	oar3_OAR〉	1.35E+08	-0.0233
27	oar3_OAR〉	1.35E+08	0.026055
27	oar3_OAR〉	1.35E+08	0.048106
27	oar3_OAR〉	1.35E+08	0.009146
27	oar3_OAR〉	1.35E+08	-0.00181
27	oar3_OAR〉	1.35E+08	0.052252
27	oar3_OAR〉	1.35E+08	0.043995
27	oar3_OAR〉	1.35E+08	0.028715
27	oar3_OAR〉	1.35E+08	0.095663

27	oar3_OAR›	1.35E+08	0.037198
27	oar3_OAR›	1.35E+08	0.020321
27	oar3_OAR›	1.35E+08	0.012157
27	oar3_OAR›	1.35E+08	0.004747
27	oar3_OAR›	1.35E+08	0.042346
27	oar3_OAR›	1.35E+08	-0.02217
27	oar3_OAR›	1.35E+08	0.057704
27	oar3_OAR›	1.35E+08	-0.02049
27	oar3_OAR›	1.35E+08	-0.00952
27	oar3_OAR›	1.35E+08	0.057704
27	oar3_OAR›	1.35E+08	0.004747
27	oar3_OAR›	1.35E+08	0.042346
27	oar3_OAR›	1.35E+08	0.042346
27	oar3_OAR›	1.35E+08	0.078761
27	oar3_OAR›	1.35E+08	0.105518
27	oar3_OAR›	1.35E+08	0.061427
27	oar3_OAR›	1.35E+08	0.002694
27	oar3_OAR›	1.35E+08	0.085782
27	oar3_OAR›	1.35E+08	0.002173
27	oar3_OAR›	1.35E+08	0.060256
27	oar3_OAR›	1.35E+08	0.002173
27	oar3_OAR›	1.35E+08	0.061427
27	oar3_OAR›	1.35E+08	0.000889
27	oar3_OAR›	1.35E+08	0.034602
27	oar3_OAR›	1.35E+08	0.060256
27	oar3_OAR›	1.35E+08	-0.00404
27	oar3_OAR›	1.35E+08	0.029082
27	oar3_OAR›	1.35E+08	0.029082
27	oar3_OAR›	1.35E+08	0.029082
27	oar3_OAR›	1.35E+08	-0.00061
27	oar3_OAR›	1.35E+08	0.010985
27	oar3_OAR›	1.35E+08	0.070814
27	oar3_OAR›	1.35E+08	0.010985
27	oar3_OAR›	1.35E+08	0.070814
27	oar3_OAR›	1.35E+08	0.067788
27	oar3_OAR›	1.35E+08	0.030952
27	oar3_OAR›	1.35E+08	0.011228
27	oar3_OAR›	1.35E+08	0.080447
27	oar3_OAR›	1.35E+08	-0.005
27	oar3_OAR›	1.35E+08	0.016711
27	oar3_OAR›	1.35E+08	0.016711
27	oar3_OAR›	1.35E+08	0.019247
27	oar3_OAR›	1.35E+08	0.019247
27	oar3_OAR›	1.35E+08	0.019247
27	oar3_OAR›	1.35E+08	0.102493
27	oar3_OAR›	1.35E+08	0.019247
27	oar3_OAR›	1.35E+08	-0.01957
27	oar3_OAR›	1.35E+08	0.019247
27	oar3_OAR›	1.35E+08	0.012436
27	oar3_OAR›	1.35E+08	0.012436
27	oar3_OAR›	1.35E+08	0.102493
27	oar3_OAR›	1.35E+08	-0.00833
27	oar3_OAR›	1.35E+08	-0.02551
27	oar3_OAR›	1.35E+08	-0.02551

27	oar3_OAR〉	1.35E+08	0.012436
27	oar3_OAR〉	1.35E+08	-0.02551
27	oar3_OAR〉	1.35E+08	-0.02551
27	oar3_OAR〉	1.35E+08	0.012436
27	oar3_OAR〉	1.35E+08	0.029915
27	oar3_OAR〉	1.35E+08	0.042012
27	oar3_OAR〉	1.35E+08	0.029915
27	oar3_OAR〉	1.35E+08	0.042012
27	oar3_OAR〉	1.35E+08	0.012436
27	oar3_OAR〉	1.35E+08	0.003678
27	oar3_OAR〉	1.35E+08	0.029915
27	oar3_OAR〉	1.35E+08	0.012436
27	oar3_OAR〉	1.35E+08	0.029915
27	oar3_OAR〉	1.35E+08	0.003678
27	oar3_OAR〉	1.35E+08	0.012436
27	oar3_OAR〉	1.35E+08	0.029915
27	oar3_OAR〉	1.35E+08	0.012436
27	oar3_OAR〉	1.35E+08	0.003678
